# Iridium-Catalyzed Enantioselective Alkene Hydroalkylation via a Heteroaryl-Directed Enolization–Decarboxylation Sequence

**DOI:** 10.1021/jacs.3c10163

**Published:** 2023-10-25

**Authors:** Changcheng Jing, Wenbin Mao, John F. Bower

**Affiliations:** Department of Chemistry, University of Liverpool, Crown Street, Liverpool L69 7ZD, United Kingdom

## Abstract

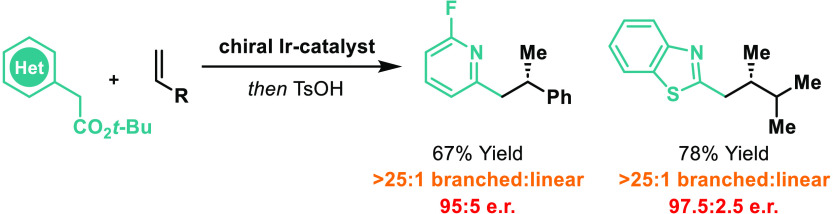

Upon exposure to a cationic Ir(I)-complex modified with the chiral diphosphine DuanPhos, hydroalkylations of styrenes and α-olefins with diverse heteroaryl *tert*-butyl acetates occur with complete branched selectivity and very high enantioselectivity. The initial adducts undergo acid promoted decarboxylation in situ to provide alkylated heteroarenes possessing defined β-stereocenters. The processes are postulated to proceed via a stereodefined chiral Ir-enolate, which arises upon heteroarene directed enolization of the heteroaryl acetate precursor. The method can be classified as an enantioselective decarboxylative C(sp^3^)–C(sp^3^) cross-coupling.

Cross-couplings that involve the enantioselective intermolecular addition of C–H bonds across alkenes are attractive because they (a) minimize prefunctionalization, (b) maximize step and atom economy, and (c) employ readily available feedstocks as a coupling partner (Scheme 1A).^1^ A promising framework for achieving such processes is via directed metal insertion into C–H bonds (i.e., C–H activation).^1−6^ This approach generally requires polarized alkenes because these (a) offer enhanced reactivity and (b) are electronically predisposed to regioselective functionalization. The latter is important for enforcing branched versus linear selectivity for processes involving monosubstituted alkenes (cf. Scheme 1A). Indeed, there are only a few enantioselective protocols that can accommodate minimally activated monosubstituted alkenes (i.e., styrenes and α-olefins), with Ir-based catalysts proving to be especially promising for hydroarylation and -alkenylation reactions (Scheme 1B). For example, our group developed highly branched and enantioselective Ir-catalyzed *hydroarylations* of styrenes and α-olefins with acetanilides (eq 1).^2a^ More recently, Li and co-workers reported branched and enantioselective *hydroalkenylation* reactions that use enamides (eq 2).^3a^ Here, hydrolysis of the initial hydroalkenylation products furnished ketones, thereby providing a two-step method for enantioselective alkene *hydroalkylation*. In a prior report, Dong and co-workers achieved promising levels of stereocontrol for a related method where an enamine is generated catalytically.^3b^ In this study, we outline a distinct *decarboxylative alkene hydroalkylation* process that is postulated to occur by directed enolization rather than via directed C–H activation (Scheme 1C).^6^ This cross-coupling process is enabled by the hitherto unreported Ir-catalyzed branched selective hydroalkylation of styrenes or α-olefins with 2-azaaryl acetates. The method offers high enantioselectivities and provides an attractive framework for the assembly of challenging C(sp^3^)–C(sp^3^) bonds.^7^

**Scheme 1:**
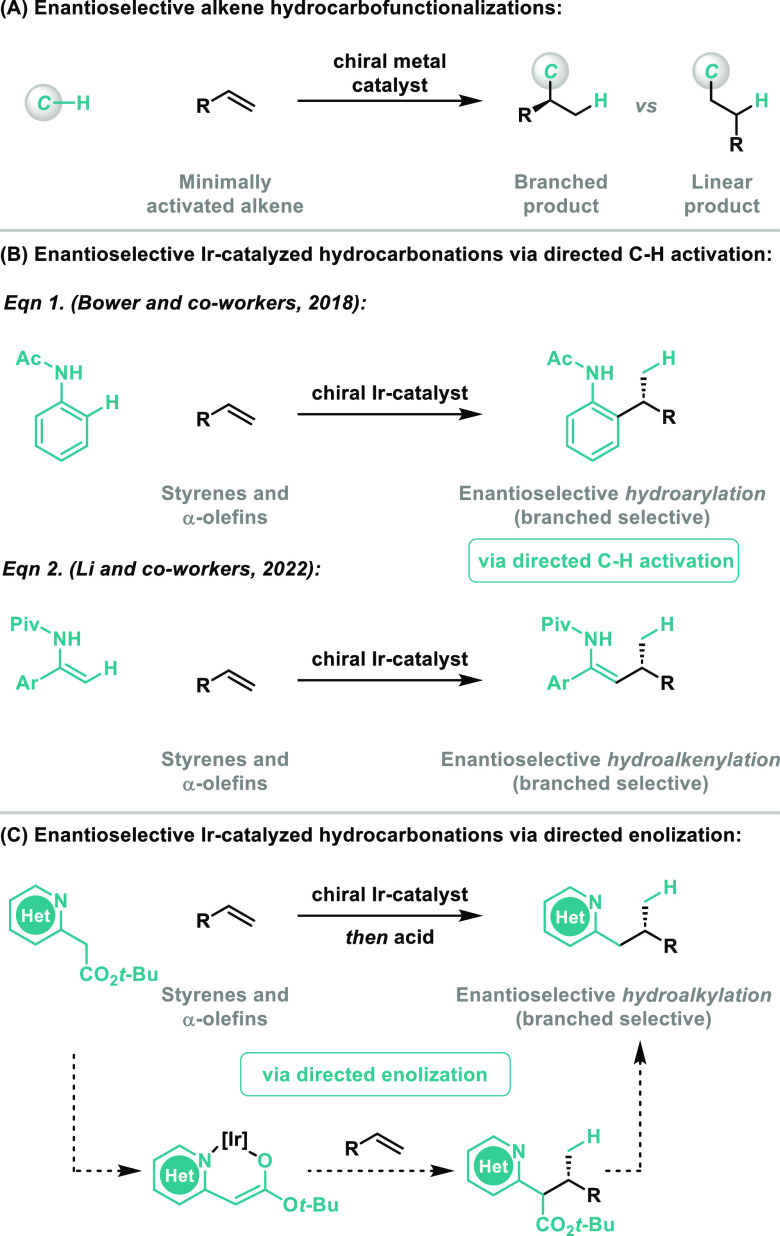
Introduction

Our studies were inspired by recent reports from Takeuchi and co-workers, who showed that cationic Ir(I)-systems are unusually effective for the hydroalkylation of minimally activated alkenes with 1,3-dicarbonyl compounds.^8^ So far, enantioselective variants of this chemistry have not been reported. Although the processes are mechanistically unclear, a possible reason for this is that transfer of chiral information from the metal-center may be challenging if C–C bond formation occurs via outer sphere attack of an enol onto an Ir-π-complex.^8a,9^ To address this, we considered redesigning the processes to enforce the Ir-catalyzed activation of the pronucleophile rather than the proelectrophile. Accordingly, we were drawn to the use of 2-azaaryl acetates; here, we envisaged that coordination of the Lewis basic N-center to the Ir-catalyst would trigger enolization (Scheme 1C).^10,11^ The resulting chiral nucleophile should then be able to transfer stereochemical information more efficiently during C–C bond formation with the alkene. A subsequent decarboxylation process would then provide direct access to medicinally valuable β-stereogenic aza-arenes.^12^

In early studies toward the envisaged process, we exposed 2-pyridyl *tert-*butyl acetate **1a** to styrene **2a** and [Ir(cod)_2_]BARF/**L1** (5 mol %) at 100 °C in PhMe (0.5 M) (Table 1, entry 1). Under these conditions, smooth conversion to **3aa** was observed; this material was formed with very high branched to linear selectivity (B:L > 25:1) and as an inconsequential mixture of diastereomers (3.7:1 dr). Decarboxylation of **3aa** was readily achieved by addition of *p*-TsOH (30 mol %), and this telescoped procedure provided target **4aa** in 81% yield and 56:44 er. Optimization studies focused primarily on the evaluation of a wide range of chiral diphosphine ligands; selected results are presented in Table 1, and further studies are summarized in SI. Other BINAP-like systems **L2** and **L3** offered good chemical efficiency and improvements in enantioselectivity (entries 2 and 3). Spirodiindane system **L4** improved enantioselectivity further, albeit at the expense of yield (entry 4). Ultimately, we explored more electron rich diphosphines **L5** and **L6** (entries 5 and 6), and found that the latter offers a good balance of yield and selectivity. Lower reaction temperatures offered no overall benefits (entry 7). For this pyridyl-based system, a *tert*-butyl ester (**1a**) was found to be critical: methyl and ethyl variants **1a′** and **1a′′** were ineffective for the initial alkene hydroalkylation step (entries 8 and 9). Using **1a** and **L6**, we validated that a reaction concentration of 0.5 M is optimal (entries 10 and 11). The use of a BARF counterion is important: replacement of [Ir(cod)_2_]BARF with, for example, [Ir(cod)_2_]BF_4_ or [Ir(cod)_2_]OTf resulted in no conversion or decomposition, respectively. PhMe can be replaced with a range of other solvents (e.g., 2-MeTHF, 1,4-dioxane, anisole), and yields and selectivities are maintained (see the SI). The absolute stereochemistry of **4aa** was determined by comparison of its specific rotation value to literature data for the antipode [**4aa**: [α]_D_^24^ = +76.7 (*c* 0.5, CH_2_Cl_2_), *ent*-**4aa**: (lit.) [α]_D_^25^ = −60.3 (*c* 0.5, CH_2_Cl_2_)^13^], and this provided the basis for the stereochemical assignment of other products described later.

**Table 1:**
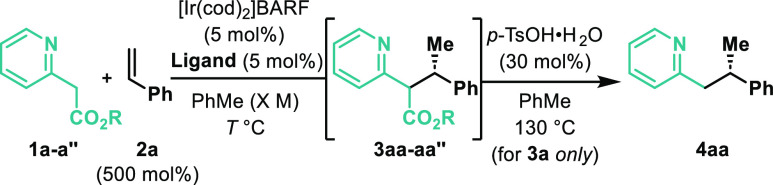

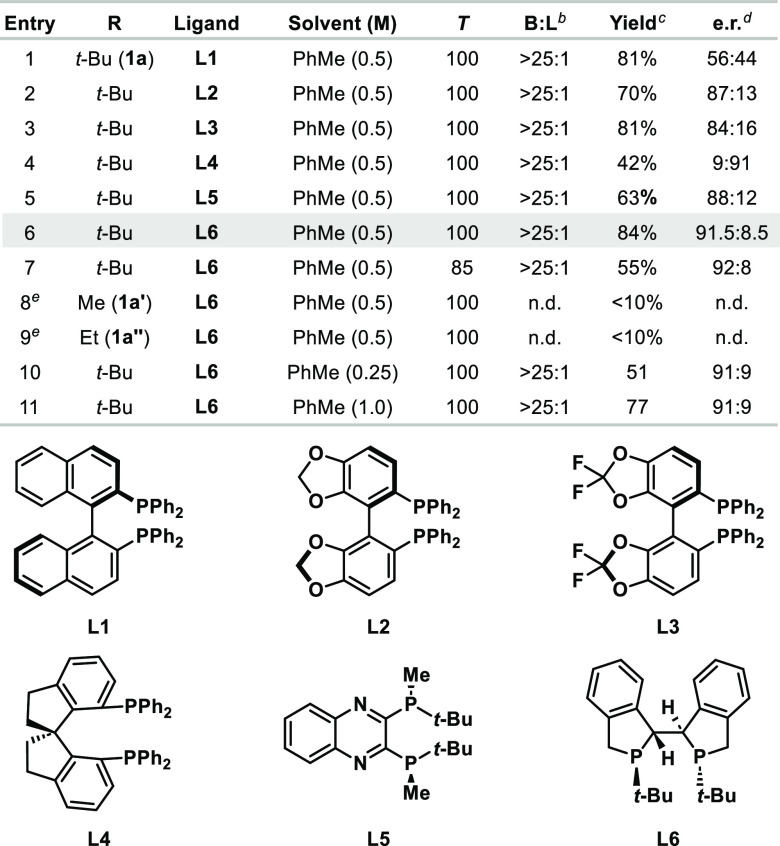
Reaction Development^a^

a Further optimization results, including the evaluation of other ligands, are given in the SI; BARF = tetrakis(3,5-bis(trifluoromethyl)phenyl)borate.

b Branched to linear (B:L) selectivities were determined by ^1^H NMR analysis of the crude mixture.

c Isolated yields are quoted.

d Determined by chiral SFC analysis.

e The results are from the first step, as confirmed by ^1^H NMR analysis of the crude mixture.

The conditions outlined in Table 1, entry 6, were used to evaluate the scope of the heteroaryl component in decarboxylative hydroalkylations of styrene **2a** (Table 2). The two-step telescoped protocol tolerates a range of electronically distinct and differentially substituted pyridines (**4ba–fa**), including those that possess potentially labile C(sp^2^)-halide bonds (**4ea**). In all cases, very high B:L selectivities were maintained, although enantioselectivity was compromised for **4fa**, which possesses a highly electron withdrawing CF_3_ unit. This may reflect a competing background reaction, wherein enolization occurs in the absence of the Ir catalyst (vide infra). Interestingly, for **3da**, we were unable to effect decarboxylation, with the *tert*-butyl ester remaining intact even upon prolonged and forcing exposure to *p*-TsOH. Note that **3da** was formed as a mixture of equally enantioenriched diastereomers; the mechanistic implications of this observation are discussed later. A variety of other N-heteroaromatic units also participate, including benzothiazoles (**4ga**, **4pa–sa**), thiazoles (**4ha–ia**), benzoxazoles (**4ja**), benzimidazoles (**4ka**), pyrazines (**4la**), triazines (**4ma**), isoquinolines (**4na**), and quinolines (**4oa**). For **4ka**, modified Krapcho-like decarboxylation conditions were required,^14^ because the *p*-TsOH-promoted protocol was not effective. Certain systems are ineffective: for example, **1t** and **1u** both decomposed under the reaction conditions.

**Table 2:**
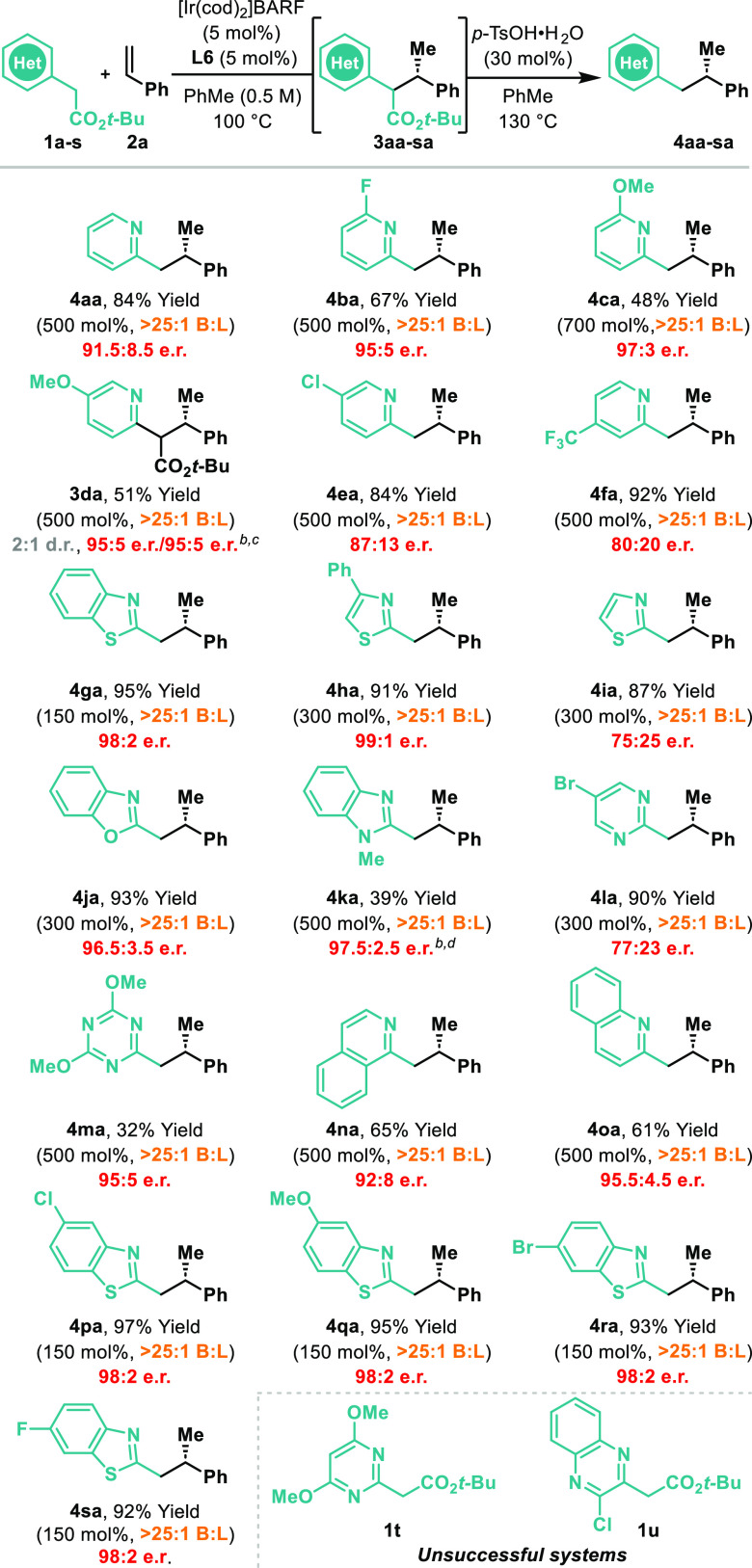
Scope of Heteroaryl Component^a^

a Alkene equivalents are specified in parentheses. Branched to linear (B:L) selectivities were determined by ^1^H NMR analysis of the crude mixture. Isolated yields are quoted and er values were determined by chiral SFC analysis of this material.

b Step 1 was performed at 120 °C.

c We were unable to promote decarboxylation to afford **4da**.

d Modified decarboxylation conditions were used: DMSO, 150 °C, 4 h.

Benzothiazole system **1g** was selected to explore the scope of the alkene reaction partner (Table 3). As with Table 2, some variance of the loading of this component was required for optimal efficiencies, but in most cases, just 150 mol % sufficed. Styrenes are excellent reaction partners, with **4gb–gi** all generated with excellent levels of regioselectivity and enantioselectivity. A bulky ferrocene-substituted alkene (**4gi**) participated, albeit with more modest levels of enantioinduction. Significantly, the process accommodates completely nonactivated alkyl-substituted alkenes. For example, decarboxylative hydroalkylation of hex-1-ene **2j** with **1g** provided **4gj** in >25:1 B:L selectivity and 93.5:6.5 er. Similar levels of efficiency were observed for other alkyl-substituted systems (**4gk–gp**). Interestingly, efficient reactivity is maintained regardless of whether the alkene α-carbon center is secondary, tertiary, or quaternary (cf. **4gk** vs **4gl** vs **4gm**). For **4gn**, potential isomerization of allyl benzene **2n** to β-methylstyrene was not observed as a significant competing process.^15^

**Table 3:**
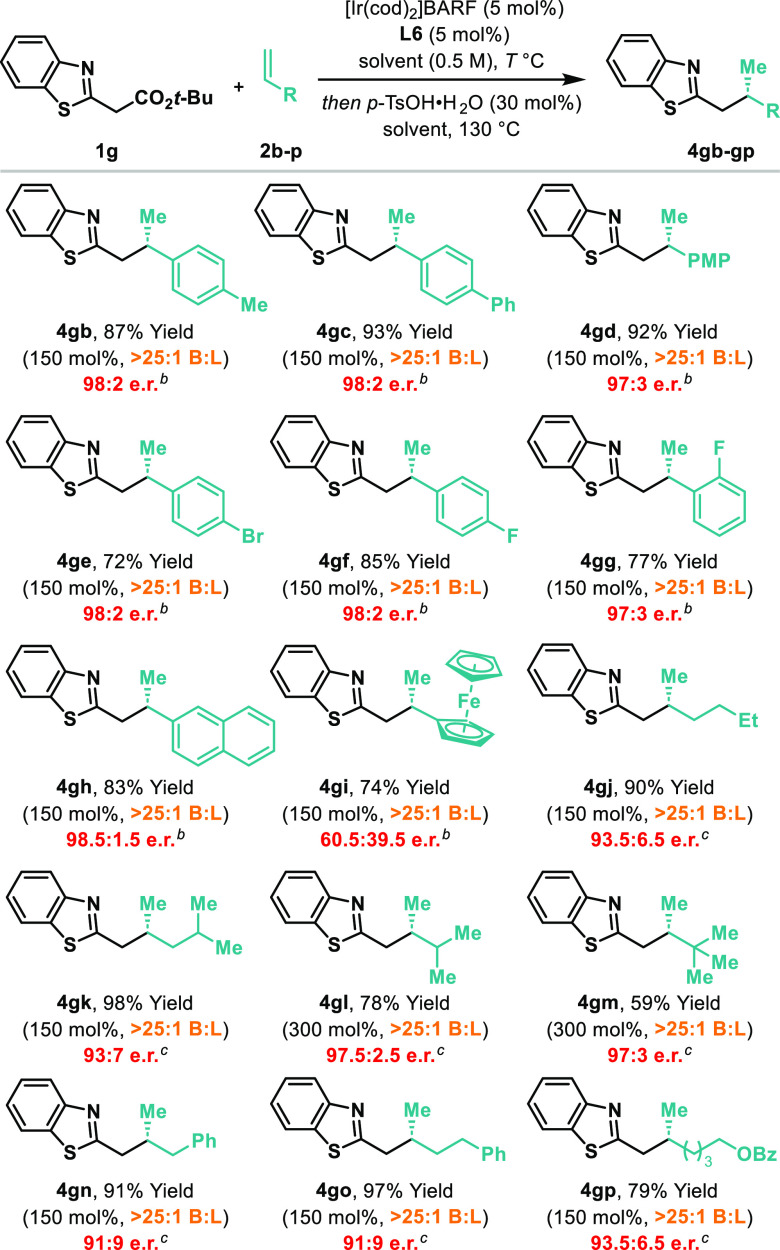
Scope of the Alkene^a^

a See Table 2 footnote; PMP = *p*-methoxyphenyl.

b The reaction was performed at 100 °C in PhMe.

c The reaction was performed at 70 °C in *m*-xylene.

The decarboxylative step in the sequence can be used to trigger further C–C bond formations. For example, hydroalkylation of ketone-containing alkene **2q** with **1g** provided **3gq** (Scheme 2A). Exposure of this to *p*-TsOH effected a decarboxylative aza-aldol-like condensation to provide chiral cyclopentene **5** in 49% yield and 91:9 er. Extension of this approach to the construction of other ring systems can be envisaged. The initial hydroalkylation products are also amenable to functionalization via the corresponding enolate (Scheme 2B). For example, formation of the lithium enolate from **3ga** allowed the stereocontrolled installation of fluoro (**6**) and methyl substituents (**7**).^16^ Again, further possibilities and applications are evident here.

**Scheme 2:**
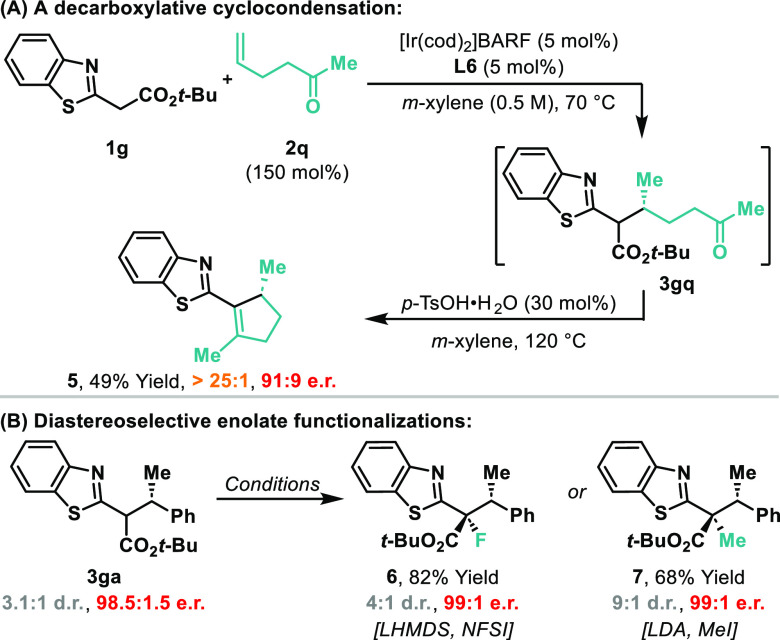
Further Utility of the Process NFSI = *N*-fluoro-*N*-(phenylsulfonyl)benzenesulfonamide.

Preliminary investigations have been undertaken to provide insight into the mechanism of the processes (Scheme 3). Using *rac*-BINAP as ligand, attempted hydroalkylation of styrene **2a** with 2- vs 3- or 4-substituted pyridyl systems (**1a′′** vs **3***N*- and **4***N***-1a′′**) revealed that only the former participates, which confirms the importance of an appropriately positioned N-center (Scheme 3A). Exposure of **1g** to these conditions in the absence of an alkene but in the presence of D_2_O (1000 mol %) revealed substantial deuterium exchange at C2 (Scheme 3B, eq 1). Lower incorporation was observed in the absence of the Ir catalyst (0.50 vs 1.59 D in eq 1). Thus, both innate and Ir-catalyzed enolization pathways may be operative. When the hydroalkylation process was run in the presence of D_2_O, deuterium incorporation was observed at the C2, methyl, and methine positions of *deuterio*-**3gc** (eq 2). As supported by eq 3, deuterium exchange at the former position can occur prior to or after C–C bond formation, whereas exchange at the latter two sites occurs at the stage of alkene **2c**, most likely via reversible hydrometalation.^17^ An additional deuterium labeling experiment was undertaken to support this (see the SI). Graphical kinetic analysis revealed the order in catalyst ([Ir(cod)_2_]BARF/**L6**) to be approximately 1 (see the SI).^18^ Currently, we favor a carbometallative pathway to form **Int-II**.^19^ This could occur either via inner sphere alkene carbometalation from Ir-enolate **Int-I**, or via outer sphere attack of enol **Int-III** onto Ir-π-complex **Int-IV**. If the latter is operative, then other enolizable pronucleophiles would be expected to participate with high levels of enantioselectivity. However, this is not the case, with, for example, β-ketoesters delivering essentially racemic hydroalkylation product.^20^ For both options in Scheme 3C, branched selectivity can be attributed to either electronic effects and/or a preference for the Ir-center to move to the less hindered end of the alkene.

**Scheme 3:**
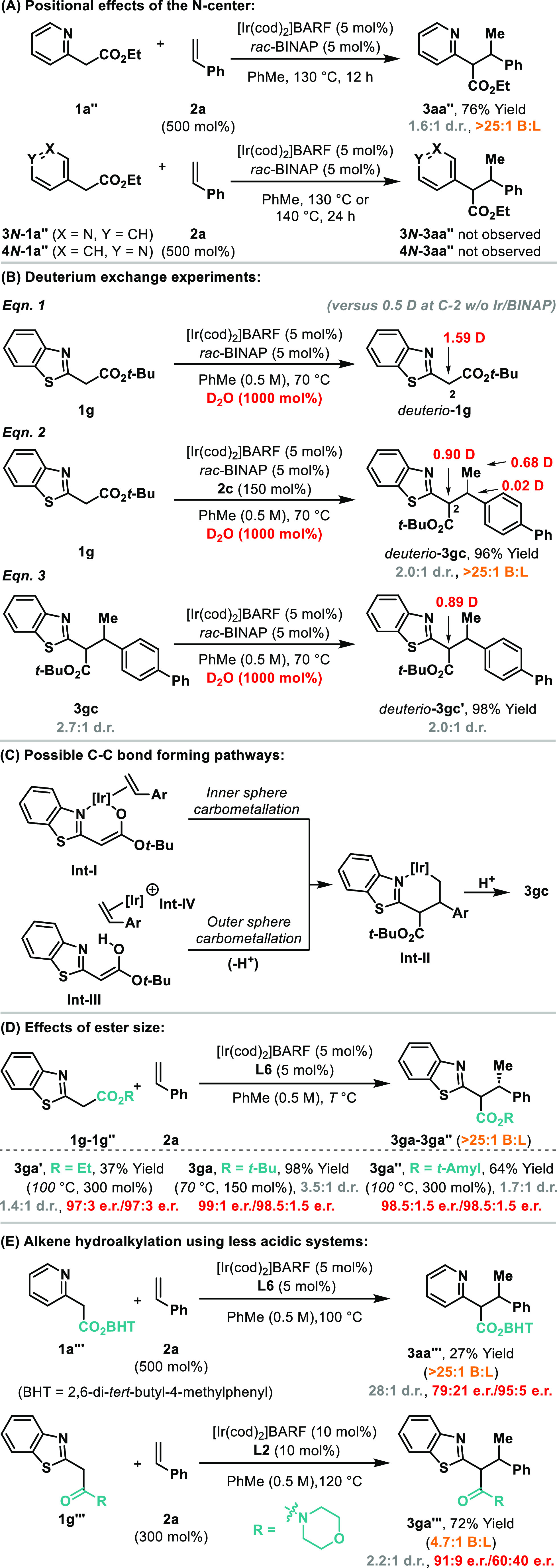
Mechanistic Studies

As outlined in Table 1, systems possessing *t*-butyl esters are especially suitable. To probe this, we compared the reaction of styrene **2a** with substrates **1g′**, **1g**, and **1g′′**, which are equipped with ethyl, *t*-butyl, and *t*-amyl esters respectively (Scheme 3D). **1g** offered the highest yield and allowed the mildest reaction conditions (70 °C vs 100 °C for **1g′** and **1g′′**). Interestingly, products **3ga–ga′′** were generated with similar levels of enantioenrichment, which shows that the structural features of the ester unit are important for reactivity but have minimal influence on enantioinduction.^21^ Note that both diastereomers of each product (**3ga–ga′′**) were isolated with the same er, which is expected given that the α-stereocenter epimerizes under the reactions conditions (cf. Scheme 3B, eq 3). Although not critical for the decarboxylative sequences described here, suppression of this equilibration process would offer insight into the degree of kinetic diastereocontrol achieved during alkene hydroalkylation. Accordingly, we evaluated the reaction of styrene with **1a′′′**, and this provided **3aa′′′** in 28:1 dr and with distinct enantioselectivities for the two diastereomers (Scheme 3E). The bulky ester unit should disfavor enolization of **3aa′′′** (to avoid A(1,3)-type strain), and so we suggest that this result reflects the kinetic outcome of the alkene hydroalkylation event. Distinct diastereomeric enantioselectivities were also observed using amide **1g′′′**, although in this case product **3ga′′′** was formed in low dr. In this process, we also observed significant leakage to the linear alkene hydroalkylation product (4.7:1 branched:linear); this demonstrates that this aspect is nontrivial, and underlines the excellent regioselectivities observed elsewhere.^22^ The high enantioselectivities observed in Tables 2 and 3 require high facial selectivity with respect to the alkene.^23^ For the proposed inner sphere pathway (**Int-I** to **Int-II**, Scheme 3C), this likely also requires high kinetic diastereoselectivity, so it is striking that the size of the ester unit in **3ga–ga′′** has minimal influence.

In summary, we demonstrate a unique Ir-catalyzed enolization–decarboxylation approach to the enantioselective hydroalkylation of minimally polarized alkenes. As outlined in the introduction, related C–H activation-based C(sp^2^)-H additions to minimally polarized alkenes have proven to be exceptionally difficult to execute because the catalyst system must promote both high branched regioselectivity and high enantioselectivity. The method described here offers a powerful alternative approach that is based on replacing the directed C–H activation step with a directed enolization process. This, in turn, enables highly demanding branched and enantioselective C(sp^3^)-H additions. The processes are mechanistically intriguing and set the stage for the exploration and development of numerous related C(sp^3^)–C(sp^3^) cross-couplings. For example, the results in Scheme 3E, which involve less acidic pronucleophiles, lay the groundwork for the development of methods for the stereocontrolled installation of contiguous stereocenters. This and other related objectives are an ongoing focus of our laboratory.

## Supporting Information Available

Experimental details, characterization data and crystallographic data (PDF)

## References

[ref1] aRajanBabu T. V. Asymmetric Hydrovinylation Reaction. Chem. Rev. 2003, 103, 2845–2860. 10.1021/cr020040g. 12914483

[ref2] aGrélaud S.; Cooper P.; Feron L. J.; Bower J. F. Branch-Selective and Enantioselective Iridium-Catalyzed Alkene Hydroarylation via Anilide-Directed C-H Oxidative Addition. J. Am. Chem. Soc. 2018, 140, 9351–9356. 10.1021/jacs.8b04627. 30024748

[ref3] aSun X.; Lin E.-Z.; Li B.-J. Iridium-Catalyzed Branch-Selective and Enantioselective Hydroalkenylation of α-Olefins through C–H Cleavage of Enamides. J. Am. Chem. Soc. 2022, 144, 17351–17358. 10.1021/jacs.2c07477. 36121772

[ref4] Zhang A.; Rajanbabu T. V. All-Carbon Quaternary Centers via Catalytic Asymmetric Hydrovinylation. New Approaches to the Exocyclic Side Chain Stereochemistry Problem. J. Am. Chem. Soc. 2006, 128, 5620–5621. 10.1021/ja060999b. 16637613

[ref5] aZhang C.; Santiago C. B.; Crawford J. M.; Sigman M. S. Enantioselective Dehydrogenative Heck Arylations of Trisubstituted Alkenes with Indoles to Construct Quaternary Stereocenters. J. Am. Chem. Soc. 2015, 137, 15668–15671. 10.1021/jacs.5b11335. 26624236 PMC5039010

[ref6] aYamauchi D.; Yamakawa K.; Nishimura T. Iridium-Catalyzed Enantioselective Direct α-C–H Alkylation of Saturated Cyclic Amines with Alkenes. Org. Lett. 2022, 24, 6828–6833. 10.1021/acs.orglett.2c02733. 36106719

[ref7] aKranthikumar R. Recent Advances in C(sp^3^)–C(sp^3^) Cross-Coupling Chemistry: A Dominant Performance of Nickel Catalysts. Organometallics 2022, 41, 667–679. 10.1021/acs.organomet.2c00032.

[ref8] aTakeuchi R.; Sagawa J.; Fujii M. Cationic Iridium Complex-Catalyzed Intermolecular Hydroalkylation of Unactivated Alkenes with 1,3-Diketones. Org. Lett. 2019, 21, 741–744. 10.1021/acs.orglett.8b03975. 30638390

[ref9] These processes are mechanistically unclear and various options have been presented in references (8a−8c).

[ref10] aJiang X.; Boehm P.; Hartwig J. F. Stereodivergent Allylation of Azaaryl Acetamides and Acetates by Synergistic Iridium and Copper Catalysis. J. Am. Chem. Soc. 2018, 140, 1239–1242. 10.1021/jacs.7b12824. 29319306 PMC5812688

[ref11] aTrost B. M.; Thaisrivongs D. A. Strategy for Employing Unstabilized Nucleophiles in Palladium-Catalyzed Asymmetric Allylic Alkylations. J. Am. Chem. Soc. 2008, 130, 14092–14093. 10.1021/ja806781u. 18826305

[ref12] Best D.; Lam H. W. C=N-Containing Azaarenes as Activating Groups in Enantioselective Catalysis. J. Org. Chem. 2014, 79, 831–845. 10.1021/jo402414k. 24341407

[ref13] Zhang C.; Gao A. Z.; Nie X.; Ye C.-X.; Ivlev S. I.; Chen S.; Meggers E. Catalytic α-Deracemization of Ketones Enabled by Photoredox Deprotonation and Enantioselective Protonation. J. Am. Chem. Soc. 2021, 143, 13393–13400. 10.1021/jacs.1c06637. 34392683

[ref14] Krapcho A. P.; Weimaster J. F.; Eldridge J. M.; Jahngen E. G. E. Jr; Lovey A. J.; Stephens W. P. Synthetic Applications and Mechanism Studies of the Decarbalkoxylations of Geminal Diesters and Related Systems Effected in Me_2_SO by Water and/or by Water with Added Salts. J. Org. Chem. 1978, 43, 138–147. 10.1021/jo00395a032.

[ref15] At the current level of development, more highly substituted alkenes are not suitable; for example, use of β-methyl styrene resulted in minimal hydroalkylation, whereas α-ethyl styrene did participate using *rac***-L1** (29% yield), but failed to react using **L6** (see the SI). For substrates that exhibited lower reactivity, higher yields and increased rates were observed using increased equivalents of alkene.

[ref16] The SI details the relative stereochemical assignment of **7**. The relative stereochemistry of **6** was assigned by analogy.

[ref17] For example, this might occur via the protonated form of **Int-I**.

[ref18] Burés J. A Simple Graphical Method to Determine the Order in Catalyst. Angew. Chem., Int. Ed. 2016, 55, 2028–2031. 10.1002/anie.201508983. PMC4797368 26749539

[ref19] Morris R. H. Brønsted–Lowry Acid Strength of Metal Hydride and Dihydrogen Complexes. Chem. Rev. 2016, 116, 8588–8654. 10.1021/acs.chemrev.5b00695. 26963836

[ref20] Using [Ir(cod)_2_]BARF/**L2** at 150 °C in PhMe, the hydroalkylation of styrene with ethyl 3-oxo-3-phenylpropanoate delivered a 2:3 ratio of branched to linear product in 77% yield after decarboxylation. The branched product was formed in < 5% e.e. For this process, **L6** was not chemically effective (see the SI for details).

[ref21] A possible explanation is that the larger ester substituent of **3ga** (vs **3ga′**) increases A(1,3)-type strain in the Ir-enolate, thereby decreasing its stability and increasing its reactivity. In this scenario, further increases in steric demand (i.e., **3ga′′**) may be detrimental to either the enolization or alkylation events.

[ref22] Acid catalyzed deamidation of **3ga′′′** provided **4ga** in 56% yield and 50% ee (see the SI).

[ref23] This interpretation assumes that alkene carbometalation is turnover limiting. For an Ir-catalyzed process involving reversible alkene carbometalation, see reference (2a).

