# Supplementary material for: Iridium-Catalyzed Enantioselective Alkene Hydroalkylation via a Heteroaryl-Directed Enolization–Decarboxylation Sequence
Source: J Am Chem Soc. 2023 Oct 25;145(44):23918–24. doi: 10.1021/jacs.3c10163 (PMC10636747; doi:10.1021/jacs.3c10163)
Supplement: Supplementary file 1 — ja3c10163_si_001.pdf [file ja3c10163_si_001.pdf]

Iridium-Catalyzed Enantioselective Alkene Hydroalkylation via a Heteroaryl-Directed Enolization-Decarboxylation Sequence

Changcheng Jing, Wenbin Mao, and John F. Bower*

Department of Chemistry, University of Liverpool, Crown Street, Liverpool, L69 7ZD, United Kingdom

<u>Supporting Information</u>

<u>Table of Contents</u>

General Information ……………………..………………………………………..……..……………S1

Experimental Procedures and Data …………………………………….………..…………..…..…S2

Reaction Development……………………………………………………….…………………S3

Substrate Synthesis and Catalysis……………………………………….…………..………S6

Further Utility of the Hydroalkylation Process ……………………...…..………..…..…..…S77

Mechanistic Studies ………………………………….……………………..………S87

Details of the Synthesis and Evaluation of Unsuccessful Substrates ……………………..S107

NMR Spectra for Novel Compounds……………..……………………………………..….S111

Notes and References …………..…..…………………………………….…………..………..S189

General Information

Reagents, Solvents and Reactions. Starting materials were purchased from commercial sources (Acros, Aldrich, Alfa Aesar, Fluorochem, TCI, Apollo Scientific) and used without further purification unless otherwise stated. Anhydrous solvents were obtained by passage through drying columns supplied by Anhydrous Engineering Ltd. The removal of solvents in vacuo was achieved using both a Büchi rotary evaporator (bath temperatures up to 40 °C) at a pressure of either 15 mmHg (diaphragm pump) or 0.1 mmHg (oil pump), as appropriate, and a high vacuum line at room temperature. Reactions requiring anhydrous conditions were run under a dry atmosphere of nitrogen or argon; glassware was either flame dried immediately prior to use or placed in an oven (200 °C) for at least 2 hours and allowed to cool either in a desiccator or under an atmosphere of nitrogen or argon; liquid reagents, solutions or solvents were added *via* syringe through rubber septa.

Chromatography. Flash column chromatography (FCC) was performed using Sigma-Aldrich silica gel (60 Å, 230-400 mesh, 40-63 μm). Thin-layer chromatography was performed using aluminium backed 60F254 silica plates. Visualisation was achieved by UV fluorescence or a basic $KMnO_4$ solution and heat.

Spectroscopy. NMR spectra were recorded on Bruker Nano 400 and Bruker Avance III HD 500 Cryo spectrometers. Chemical shifts (δ) are given in parts per million (ppm) and referenced to the appropriate residual solvent peak. Peaks are described as singlets (s), doublets (d), triplets (t), quartets (q), pentets (pent), sextets (sext), heptets (hept), multiplets (m) and broad (br). Coupling constants (J) are quoted to the nearest 0.5 Hz. Assignments of ^{1}H NMR and ^{13}C NMR signals were made, where possible, using COSY, HSQC, HMBC, and NOE experiments. *Numbering systems for NMR signal assignments are specified on the structure and are not related to those used for the compound names.* Infra-red (IR) spectra were recorded on a Perkin Elmer Spectrum Two FTIR spectrometer as either neat films or solids compressed on a diamond plate. Only selected absorption maxima (ν_{max}) are reported in wavenumbers (cm^{-1}). High resolution mass spectra (HRMS) were recorded on a VG Analytical Autospec spectrometer by Chemical Ionisation (CI) and a Bruker micrOTOF instrument or a 6200 series TOF/6500 series Q-TOF instrument by Electrospray Ionisation (ESI). Melting points were determined using a Stuart SMP30 melting point apparatus and temperature controller and are uncorrected. Optical rotation ($[\alpha]_D^T$) were measured using an ADP440+ polarimeter at the concentration and temperature stated. Enantiomeric excesses were determined using an Agilent 1290 Infinity chiral SFC as stated for each compound.

Naming of Compounds. Compound names are generated by ChemDraw 16.0 software (PerkinElmer), following IUPAC nomenclature.

Experimental Procedures and Data

General Procedure A: Synthesis of azaarylacetate from dibutyldicarbonate

To a solution of diisopropylamine (1.2 eq.) in dry THF (2.0 M) was added *n*-BuLi (2.5 M in hexane, 1.2 eq.) over a period of 15 min at –78 °C and the resulting solution was stirred at –78 °C for additional 30 min. To the above reaction mixture was added 2-methylazaarene (1.0 eq.) in dry THF (2.0 M) dropwise and the reaction mixture was stirred for another 1 h. Then dibutyldicarbonate (1.4 eq.) was added and the reaction mixture was allowed to warm to room temperature over a period of 2 h. The reaction was quenched with water (*approx.* 3 mL/mmol) and extracted with diethyl ether (*approx.* 3 × 5 mL/mmol). The combined organic phase was washed with brine (*approx.* 5 mL/mmol), dried over Na_2SO_4, filtered and concentrated *in vacuo*. The residue was purified by FCC under the conditions noted.

General Procedure B: Synthesis of azaarylacetamide from carbamoyl chloride

To a solution of 2-methylazaarene (1.0 eq.) in dry THF (3.5 M) was added *n*-BuLi (2.5 M in hexane, 2.6 eq.) over 5 min at –78 °C and the resulting mixture was stirred at –78 °C for 1 h. Then carbamoyl chloride (1.0 eq.) was added dropwise over 1 min and the reaction mixture was warmed to room temperature over 1 h and stirred for a further 17 h. The reaction was quenched with saturated aq. NH_4Cl solution (*approx.* 3 mL/mmol) and extracted with EtOAc (*approx.* 3 × 5 mL/mmol). The combined organic phase was washed with brine (*approx.* 5 mL/mmol), dried over Na_2SO_4, filtered and concentrated *in vacuo*. The residue was purified by FCC under the conditions noted.

General Procedure C: Monoarylation of *tert*-butyl acetate or *N,N*-dialkylacetamide

To a solution of heteroaryl halide (1.0 eq.) and *tert*-butyl acetate or *N*,*N*-dialkylacetamide (3.0 eq.) was added NaHMDS (0.6 M in toluene, 3.0 eq.) over 5 min at 0 °C. After stirring the mixture at 0 °C for 5 h, the reaction was warmed to room temperature and stirred for 18 h. The reaction was quenched with saturated aq. NH_4Cl solution (*approx.* 3 mL/mmol) and extracted with EtOAc (*approx.* 3 × 5 mL/mmol). The combined organic phase was washed with brine (*approx.* 5 mL/mmol), dried over Na_2SO_4, filtered and concentrated *in vacuo*. The residue was purified by FCC under the conditions noted.

General Procedure D: Pd-catalyzed monoarylation of *tert*-butyl acetate

t-BuXPhos palladium(II) phenethylamine chloride (0.5-1.0 mol%) was added to a Schlenk tube equipped with a magnetic stirrer. The Schlenk tube was sealed with a teflon septum-lined screw cap and evacuated/backfilled with argon. Heteroaryl halide (1.0 eq.), *tert*-butyl acetate and LiHMDS (1.0 M in toluene, 3.0 eq.) were added in succession *via* syringe at room temperature. The reaction mixture was allowed to stir for the time noted at room temperature. The reaction was quenched with saturated aq. NH_4Cl solution (*approx.* 3 mL/mmol) and extracted with EtOAc (*approx.* 3 × 5 mL/mmol). The

combined organic phase was washed with brine (*approx.* 5 mL/mmol), dried over Na_2SO_4, filtered and concentrated *in vacuo*. The residue was purified by FCC under the conditions noted.

<u>General Procedure E</u>: Iridium-catalyzed enantioselective alkene hydroalkylation

A Schlenk tube was charged with azaarylacetate or azaarylacetamide (0.10 mmol, 100 mol%), $[Ir(cod)_2]$BARF (0.005 mmol, 5 mol%), ligand **L6** or **L2** (0.005 mmol, 5 mol%) and alkene (if non-volatile 150-700 mol%). The Schlenk tube was evacuated/backfilled with N_2 (three cycles), then alkene (if volatile, 150-700 mol%) was added followed by toluene or *m*-xylene (0.20 mL, 0.5 M). The tube was sealed and heated at 100 °C unless otherwise stated for the time noted (**step a**). After cooling to room temperature, *p*-toluenesulfonic acid monohydrate (0.03 mmol, 30 mol%) was added and the resulting reaction mixture was heated at 130 °C unless otherwise stated for the time noted (**step b**). After cooling to room temperature, the solvent was removed under reduced pressure and the crude reaction mixture was purified by FCC under the conditions noted.

Note: All racemic compounds are synthesized according to the method outline above by using *rac*-BINAP (*rac*-**L1**).

Reaction Development

Ligands for optimization experiments

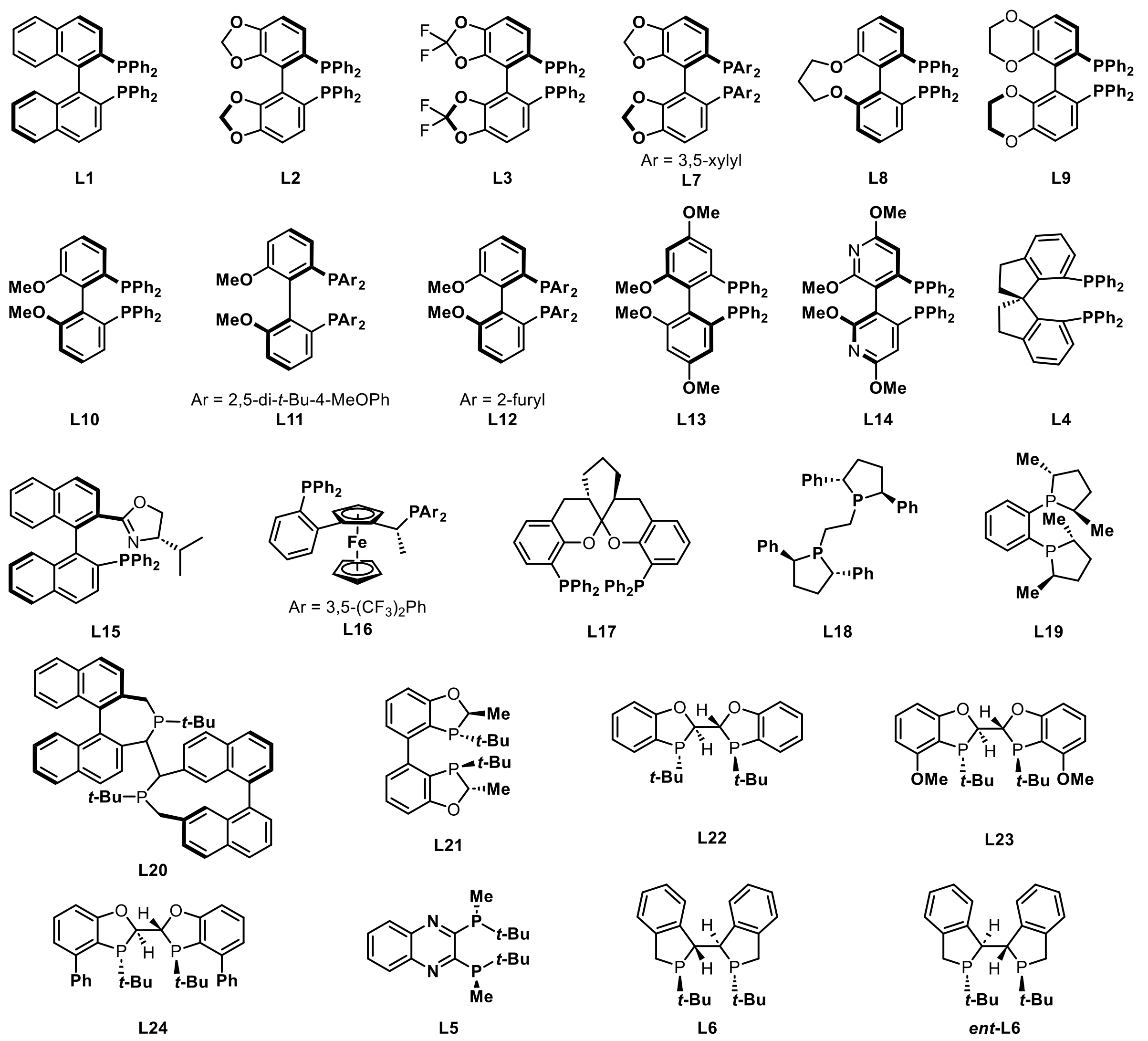

Selected additional optimization experiments for table 1 are presented below:

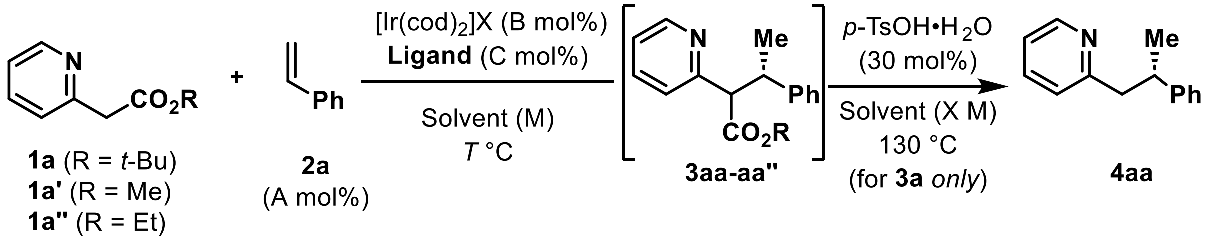

Entry	R	2a (A mol%)	X (B mol%)	Ligand (C mol%)	Solvent (M)	T (°C)	B:L[a]	Yield[b]	e.r.[c]
**1**	*t*-Bu	500	BARF (5)	**L1** (5)	toluene (0.5)	100	>25:1	81	56:44
**2**	*t*-Bu	500	BARF (5)	**L2** (5)	toluene (0.5)	100	>25:1	70	87:13

**3**	*t*-Bu	500	BARF (5)	**L3** (5)	toluene (0.5)	100	>25:1	81	84:16
**4**	*t*-Bu	500	BARF (5)	**L7** (5)	toluene (0.5)	100	>25:1	87	72:28
**5**	*t*-Bu	500	BARF (5)	**L8** (5)	toluene (0.5)	100	>25:1	88	58:42
**6**	*t*-Bu	500	BARF (5)	**L9** (5)	toluene (0.5)	100	>25:1	74	66:34
**7**	*t*-Bu	500	BARF (5)	**L10** (5)	toluene (0.5)	100	>25:1	83	83:17
**8**	*t*-Bu	500	BARF (5)	**L11** (5)	toluene (0.5)	100	n.d.	<10	n.d.
**9**	*t*-Bu	500	BARF (5)	**L12** (5)	toluene (0.5)	100	>25:1	47	79:21
**10**	*t*-Bu	500	BARF (5)	**L13** (5)	toluene (0.5)	100	n.d.	<10	n.d.
**11**	*t*-Bu	500	BARF (5)	**L14** (5)	toluene (0.5)	100	>25:1	61	86:14
**12**	*t*-Bu	500	BARF (5)	**L4** (5)	toluene (0.5)	100	>25:1	42	9:91
**13**	*t*-Bu	500	BARF (5)	**L15** (5)	toluene (0.5)	100	n.d.	<10	n.d.
**14**	*t*-Bu	500	BARF (5)	**L16** (5)	toluene (0.5)	100	n.d.	<10	n.d.
**15**	*t*-Bu	500	BARF (5)	**L17** (5)	toluene (0.5)	100	n.d.	<10	n.d.
**16**	*t*-Bu	500	BARF (5)	**L18** (5)	toluene (0.5)	100	n.d.	<10	n.d.
**17**	*t*-Bu	500	BARF (5)	**L19** (5)	toluene (0.5)	100	>25:1	56	63:37
**18**	*t*-Bu	500	BARF (5)	**L20** (5)	toluene (0.5)	100	n.d.	<10	n.d.
**19**	*t*-Bu	500	BARF (5)	**L21** (5)	toluene (0.5)	100	n.d.	<10	n.d.
**20**	*t*-Bu	500	BARF (5)	**L22** (5)	toluene (0.5)	100	>25:1	66	9:91
**21**	*t*-Bu	500	BARF (5)	**L23** (5)	toluene (0.5)	100	>25:1	45	10:90
**22**	*t*-Bu	500	BARF (5)	**L24** (5)	toluene (0.5)	100	>25:1	25	18:82
**23**	*t*-Bu	500	BARF (5)	**L5** (5)	toluene (0.5)	100	>25:1	63	88:12
**24**	*t*-Bu	500	BARF (5)	**L6** (5)	toluene (0.5)	100	>25:1	84	91.5:8.5
**25**	*t*-Bu	500	BF_4 (5)	**L6** (5)	toluene (0.5)	100	n.d.	<10	n.d.
**26**	*t*-Bu	500	OTf (5)	**L6** (5)	toluene (0.5)	100	n.d.	<10	n.d.
**27**	*t*-Bu	500	BARF (5)	**L6** (5)	1,4-dioxane (0.5)	100	>25:1	63	85:15
**28**	*t*-Bu	500	BARF (5)	**L6** (5)	2-MeTHF (0.5)	100	>25:1	71	87:13
**29**	*t*-Bu	500	BARF (5)	**L6** (5)	1,2-DCB (0.5)	100	n.d.	<10	n.d.

**30**	*t*-Bu	500	BARF (5)	**L6** (5)	*m*-xylene (0.5)	100	>25:1	57	92:8
**31**	*t*-Bu	500	BARF (5)	**L6** (5)	*o*-xylene (0.5)	100	>25:1	77	91:9
**32**	*t*-Bu	500	BARF (5)	**L6** (5)	xylenes (0.5)	100	>25:1	61	91:9
**33**	*t*-Bu	500	BARF (5)	**L6** (5)	mesitylene (0.5)	100	>25:1	66	91:9
**34**	*t*-Bu	500	BARF (5)	**L6** (5)	anisole (0.5)	100	>25:1	51	91:9
**35**	*t*-Bu	500	BARF (5)	**L6** (5)	toluene (1.0)	100	>25:1	77	91:9
**36**	*t*-Bu	500	BARF (5)	**L6** (5)	toluene (0.25)	100	>25:1	51	91:9
**37**	*t*-Bu	250	BARF (5)	**L6** (5)	toluene (0.5)	100	>25:1	25	91:9
**38**	*t*-Bu	500	BARF (3)	**L6** (3)	toluene (0.5)	100	>25:1	27	91:9
**39**[d]	Me	500	BARF (5)	**L6** (5)	toluene (0.5)	100	n.d.	<10	n.d.
**40**[d]	Et	500	BARF (5)	**L6** (5)	toluene (0.5)	100	n.d.	<10	n.d.
**41**	*t*-Bu	500	BARF (5)	**L6** (5)	toluene (0.5)	85	>25:1	55	92:8
**42**	*t*-Bu	500	BARF (5)	***ent*-L6** (5)	toluene (0.5)	100	>25:1	81	9:91

[a] Branched to linear (B:L) selectivities were determined by ^{1}H NMR analysis of the crude mixture. [b] Isolated yields are quoted. [c] Determined by chiral SFC analysis. [d] The results are from the first step, as confirmed by ^{1}H NMR analysis of the crude mixture.

Substrate Synthesis and Catalysis

***tert*-Butyl 2-(pyridin-2-yl)acetate (1a)**

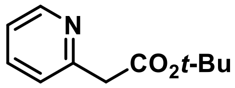

To a solution of 2-picoline (4.66 g, 4.93 mL, 50.0 mmol, 1.0 eq.) in dry THF (50.0 mL) was added *n*-BuLi (2.5 M in hexane, 24.0 mL, 60.0 mmol, 1.2 eq.) over a period of 15 min at 0 °C and the resulting solution was stirred at 0 °C for additional 30 min. Then dibutyldicarbonate (14.2 g, 65.0 mmol, 1.3 eq.) was added portionwise and the resulting reaction mixture was stirred at 0 °C for 3 h. The reaction was quenched with water (30 mL) and extracted with diethyl ether (3 × 30 mL). The combined organic phase was washed with brine (30 mL), dried over Na_2SO_4, filtered and concentrated *in vacuo*. The residue was purified by flash column chromatography (Hexane/EtOAc = 10/1 to 3/1) afforded the title compound (3.58 g, 37%) as a yellow oil. ^{1}H NMR (400 MHz, $CDCl_3$) δ_H = 8.55 (ddd, *J* = 4.9, 1.9, 1.0 Hz, 1H), 7.65 (ddd, *J* = 7.8, 7.7, 1.9 Hz, 1H), 7.28 (ddd, *J* = 7.8, 1.1, 1.0 Hz, 1H), 7.17 (ddd, *J* = 7.7, 4.9, 1.1 Hz, 1H), 3.76 (s, 2H), 1.45 (s, 9H); ^{13}C NMR (101 MHz, $CDCl_3$) δ_C = 170.1, 155.1, 149.4, 136.7, 124.0, 122.0, 81.3, 45.2, 28.2.

The spectroscopic properties were consistent with the data available in the literature.[1]

(*S*)-2-(2-Phenylpropyl)pyridine (4aa)

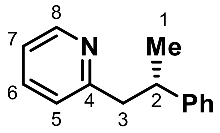

General procedure E: The preceding azaarylacetate (19.3 mg, 0.10 mmol) and styrene (52.1 mg, 57.5 μL, 0.50 mmol) were employed with [Ir(cod)$_2$]BARF(6.36 mg, 0.005 mmol) and **L6** (1.91 mg, 0.005 mmol) in toluene (0.20 mL). The reaction was stirred at 100 °C for 72 h. Then *p*-toluenesulfonic acid monohydrate (5.71 mg, 0.03 mmol) was added and the resulting reaction mixture was heated at 130 °C for 8 h. Purification by flash column chromatography (Hexane/EtOAc = 10/1 to 4/1) afforded the title compound (16.5 mg, 84%, B:L > 25:1, 91.5:8.5 e.r.) as a colorless oil. $[\alpha]_D^{25}$ = +76.7 (*c* 0.5, CH_2Cl_2).

SFC conditions: CHIRALPACK SC (25 cm), 97:3 CO_2:*i*-PrOH, 2.0 mL/min, 254 nm; *Retention times*: t_{minor} = 5.4 min, t_{major} = 5.9 min.

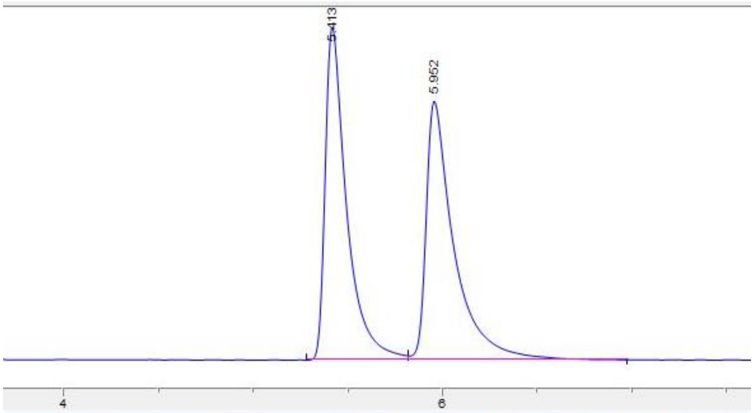

#	Time	Area	Height	Width	Area%	Symmetry
1	5.413	1853.3	235.3	0.1159	49.722	0.445
2	5.952	1874	182.9	0.1456	50.278	0.368

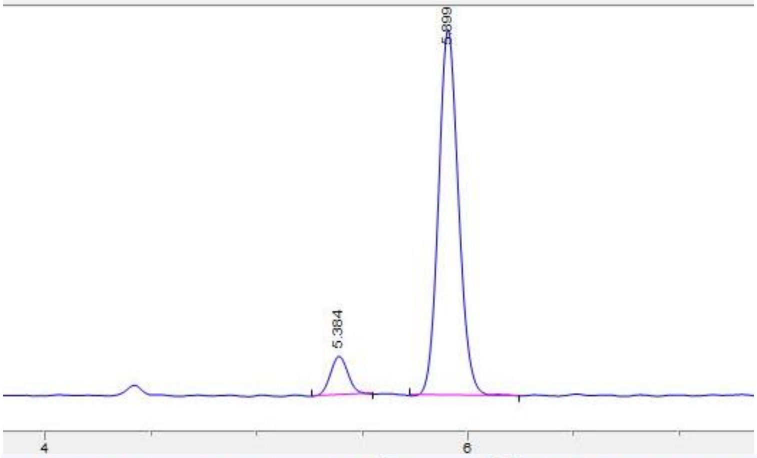

#	Time	Area	Height	Width	Area%	Symmetry
1	5.384	31	5.3	0.0924	8.618	0.916
2	5.899	329	49.8	0.103	91.382	0.856

(*R*)-2-(2-Phenylpropyl)pyridine (*ent*-4aa)

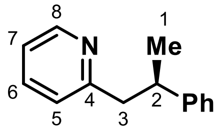

General procedure E: The preceding azaarylacetate (19.3 mg, 0.10 mmol) and styrene (52.1 mg, 57.5 μL, 0.50 mmol) were employed with [Ir(cod)$_2$]BARF (6.36 mg, 0.005 mmol) and ***ent*-L6** (1.91 mg, 0.005 mmol) in toluene (0.20 mL). The reaction was stirred at 100 °C for 72 h. Then *p*-toluenesulfonic acid monohydrate (5.71 mg, 0.03 mmol) was added and the resulting reaction mixture was heated at 130 °C for 8 h. Purification by flash column chromatography (Hexane/EtOAc = 10/1 to 4/1) afforded the title compound (15.9 mg, 81%, B:L > 25:1, 9:91 e.r.) as a colorless oil. $[\alpha]_D^{24}$ = -81.7 (*c* 0.5, CH_2Cl_2).

SFC conditions: CHIRALPACK SC (25 cm), 97:3 CO_2:*i*-PrOH, 2.0 mL/min, 254 nm; *Retention times*: t_{major} = 5.4 min, t_{minor} = 5.9 min.

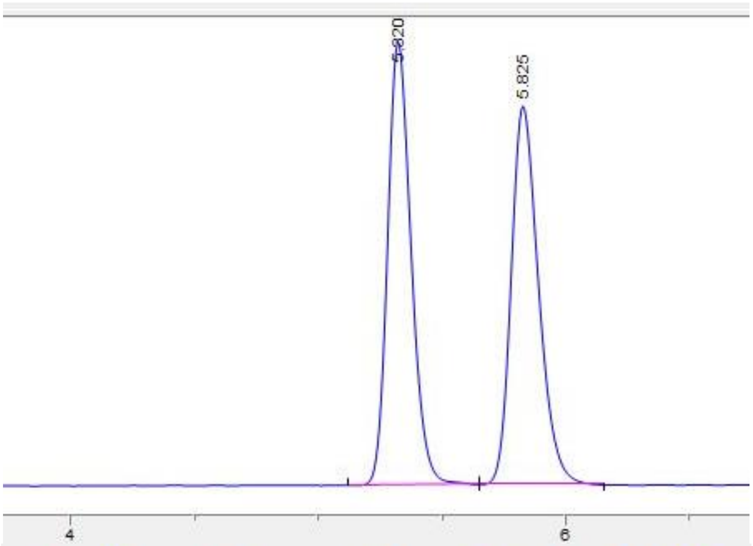

#	Time	Area	Height	Width	Area%	Symmetry
1	5.32	1384.9	210	0.1009	50.151	0.757
2	5.825	1376.6	179	0.1177	49.849	0.71

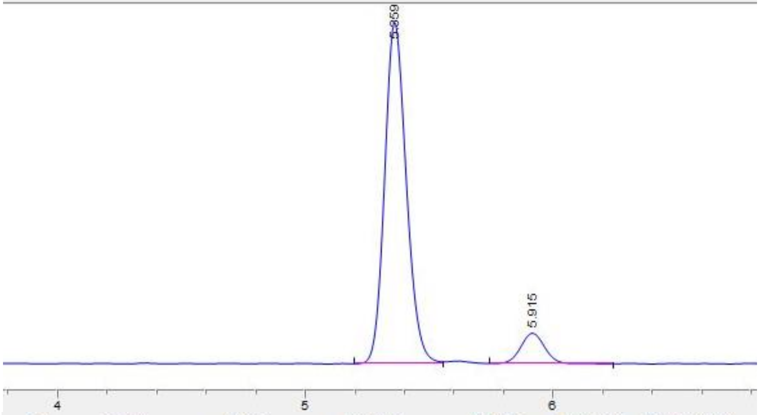

#	Time	Area	Height	Width	Area%	Symmetry
1	5.359	752	123.6	0.095	90.896	0.806
2	5.915	75.3	11.1	0.1055	9.104	0.92

(*S*)-2-(2-Phenylpropyl)pyridine and (*R*)-2-(2-Phenylpropyl)pyridine: IR (thin film) ν_{max}/cm^{-1}: 2960, 2926, 1589, 1434, 1148, 761. ^{1}H NMR (400 MHz, CDCl$_3$) δ_H = 8.56 (ddd, *J* = 4.9, 1.9, 1.0 Hz, 1H, C**8**-H), 7.51 (ddd, *J* = 7.8, 7.6, 1.9 Hz, 1H, C**6**-H), 7.31 – 7.25 (m, 2H, Ph ArCH), 7.24 – 7.14 (m, 3H, Ph ArCH), 7.09 (ddd, *J* = 7.6, 4.9, 1.2 Hz, 1H, C**7**-H), 6.95 (ddd, *J* = 7.8, 1.2, 1.0 Hz, 1H, C**5**-H), 3.31 (ddq, *J* = 8.1, 7.0, 7.0 Hz, 1H, C**2**-H), 3.10 (dd, *J* = 13.3, 7.0 Hz, 1H, C**3**-H), 3.00 (dd, *J* = 13.3, 8.1 Hz, 1H, C**3**-H'), 1.29 (d, *J* = 7.0 Hz, 3H, C**1**-H$_3$); ^{13}C NMR (101 MHz, CDCl$_3$) δ_C = 160.7 (C**4**), 149.3 (C**8**), 146.8 (Ph ArC), 136.2 (C**6**), 128.4 (Ph ArCH), 127.1 (Ph ArCH), 126.2 (Ph ArCH), 123.8 (C**5**), 121.2 (C**7**), 47.3 (C**3**), 40.5 (C**2**), 21.5 (C**1**). HRMS (ESI$^+$) calculated for $C_{14}H_{16}N$ [M+H]$^+$ = 198.1277, found 198.1283.

***tert*-Butyl 2-(6-fluoropyridin-2-yl)acetate (1b)**

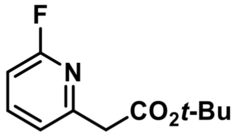

General procedure A: 2-Fluoro-6-methylpyridine (1.11 g, 10.0 mmol) was employed with diisopropylamine (1.68 mL, 12.0 mmol), *n*-BuLi (2.5 M in hexane, 4.80 mL, 12.0 mmol) and dibutyldicarbonate (2.62 g, 12.0 mmol). Purification by flash column chromatography (Hexane/EtOAc = 100/0 to 30/1) afforded the title compound (890 mg, 42%) as a colorless oil. ^{1}H NMR (500 MHz, CDCl$_3$) δ_H = 7.80 – 7.69 (m, 1H), 7.17 (dd, *J* = 7.4, 2.5 Hz, 1H), 6.82 (dd, *J* = 8.2, 2.5 Hz, 1H), 3.70 (s, 2H), 1.45 (s, 9H); ^{13}C NMR (125 MHz, CDCl$_3$) δ_C = 169.5, 163.2 (d, $^{1}J_{CF}$ = 239.2 Hz), 154.0 (d, $^{3}J_{CF}$ = 13.4 Hz), 141.5 (d, $^{3}J_{CF}$ = 7.7 Hz), 121.3 (d, $^{4}J_{CF}$ = 4.4 Hz), 107.8 (d, $^{2}J_{CF}$ = 36.7 Hz), 81.7, 44.4, 28.2.

The spectroscopic properties were consistent with the data available in the literature.[2]

(*S*)-2-Fluoro-6-(2-phenylpropyl)pyridine (4ba)

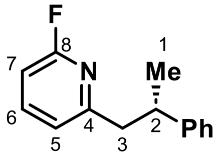

General procedure E: The preceding azaarylacetate (21.1 mg, 0.10 mmol) and styrene (52.1 mg, 57.5 µL, 0.50 mmol) were employed with [Ir(cod)$_2$]BARF (6.36 mg, 0.005 mmol) and **L6** (1.91 mg, 0.005 mmol) in toluene (0.20 mL). The reaction was stirred at 100 °C for 120 h. Then *p*-toluenesulfonic acid monohydrate (5.71 mg, 0.03 mmol) was added and the resulting reaction mixture was heated at 130 °C for 2 h. Purification by flash column chromatography (Hexane/Toluene = 9/1 to 4/1) afforded the title compound (14.4 mg, 67%, B:L > 25:1, 95:5 e.r.) as a colorless oil. $[\alpha]_D^{23}$ = +92.6 (*c* 0.5, CH_2Cl_2). IR (thin film) ν_{max}/cm^{-1}: 2962, 2926, 1606, 1452, 1264, 969, 783. ^{1}H NMR (500 MHz, CDCl$_3$) δ_H = 7.62 – 7.55 (m, 1H, C**6**-H), 7.30 – 7.26 (m, 2H, Ph ArCH), 7.22 – 7.16 (m, 3H, Ph ArCH), 6.81 (dd, *J* = 7.3, 2.6 Hz, 1H, C**5**-H), 6.72 (dd, *J* = 8.1, 2.6 Hz, 1H, C**7**-H), 3.31 (ddq, *J* = 7.9, 7.3, 6.9 Hz, 1H, C**2**-H),

3.03 (dd, J = 13.4, 7.3 Hz, 1H, C**3**-H), 2.95 (dd, J = 13.4, 7.9 Hz, 1H, C**3**-H'), 1.29 (d, J = 6.9 Hz, 3H, C**1**-H_3); ^{13}C NMR (125 MHz, $CDCl_3$) δ_C = 163.3 (d, $^1J_{CF}$ = 238.0 Hz, C**8**), 159.9 (d, $^3J_{CF}$ = 12.9 Hz, C**4**), 146.4 (Ph ArC), 140.9 (d, $^3J_{CF}$ = 7.7 Hz, C**6**), 128.5 (Ph ArCH), 127.1 (Ph ArCH), 126.3 (Ph ArCH), 120.9 (d, $^4J_{CF}$ = 4.1 Hz, C**5**), 106.7 (d, $^2J_{CF}$ = 37.2 Hz, C**7**), 46.4 (C**3**), 40.1 (C**2**), 21.6 (C**1**); ^{19}F NMR (471 MHz, $CDCl_3$) δ_F = -67.6 – -67.8 (m, 1F). HRMS (ESI$^+$) calculated for $C_{14}H_{15}FN$ $[M+H]^+$ = 216.1183, found 216.1181.

SFC conditions: CHIRALPACK SB (25 cm), 98.5:1.5 CO_2:MeOH, 1.0 mL/min, 254 nm; *Retention times*: t_{minor} = 10.4 min, t_{major} = 10.9 min.

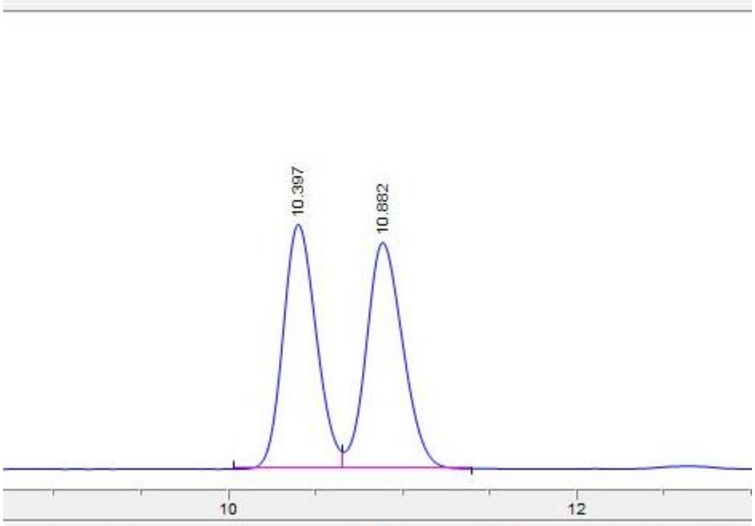

#	Time	Area	Height	Width	Area%	Symmetry
1	10.397	7815.5	589.5	0.2067	49.580	0.811
2	10.882	7947.9	545.2	0.2261	50.420	0.787

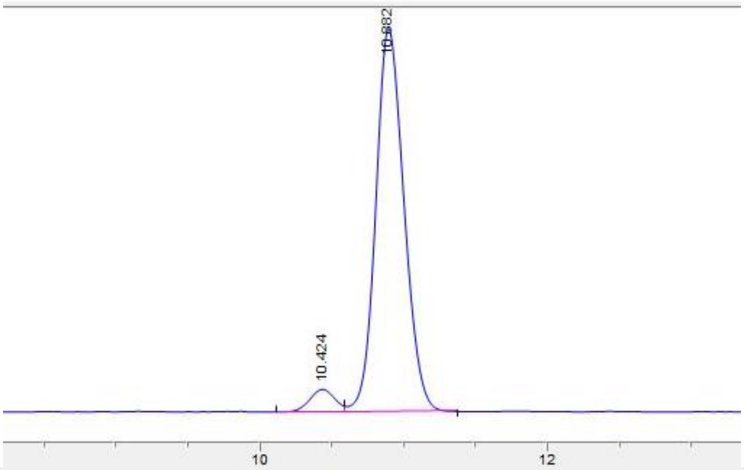

#	Time	Area	Height	Width	Area%	Symmetry
1	10.424	349.9	28.4	0.1876	5.054	1.034
2	10.882	6573.1	482	0.2091	94.946	0.804

tert-Butyl 2-(6-methoxypyridin-2-yl)acetate (1c)

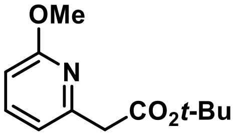

<u>General procedure D</u>: 2-Chloro-6-methoxypyridine (574 mg, 4.00 mmol) was employed with *t*-BuXPhos palladium(II) phenethylamine chloride (13.7 mg, 0.02 mmol), *tert*-butyl acetate (697 mg, 804 μL, 6.00 mmol) and LiHMDS (1.0 M in toluene, 12.0 mL, 12.0 mmol). The reaction was stirred for 2 h. Purification by flash column chromatography (Hexane/EtOAc = 15/1 to 9/1) afforded the title compound (485 mg, 54%) as a colorless oil. ^{1}H NMR (500 MHz, $CDCl_3$) δ_H = 7.50 (dd, J = 8.2, 7.3 Hz, 1H), 6.81 (d, J = 7.3 Hz, 1H), 6.60 (d, J = 8.2 Hz, 1H), 3.90 (s, 3H), 3.64 (s, 2H), 1.46 (s, 9H); ^{13}C NMR (125 MHz, $CDCl_3$) δ_C = 170.2, 163.8, 152.8, 139.0, 116.3, 108.8, 81.0, 53.4, 45.0, 28.2.

The spectroscopic properties were consistent with the data available in the literature.[3]

(*S*)-2-Methoxy-6-(2-phenylpropyl)pyridine (4ca)

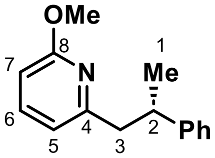

<u>General procedure E</u>: The preceding azaarylacetate (22.3 mg, 0.10 mmol) and styrene (72.9 mg, 80.2 μL, 0.70 mmol) were employed with [Ir(cod)$_2$]BARF (6.36 mg, 0.005 mmol) and **L6** (1.91 mg, 0.005 mmol) in toluene (0.20 mL). The reaction was stirred at 100 °C for 144 h. Then *p*-toluenesulfonic acid monohydrate (5.71 mg, 0.03 mmol) was added and the resulting reaction mixture was heated at 130 °C for 6 h. Purification by flash column chromatography (Hexane/EtOAc = 20/1 to 9/1) afforded the title compound (10.8 mg, 48%, B:L > 25:1, 97:3 e.r.) as a colorless oil. $[\alpha]_D^{26}$ = +99.7 (c 0.2, CH_2Cl_2). IR (thin film) ν_{max}/cm^{-1}: 2922, 2851, 1578, 1465, 1037, 798. ^{1}H NMR (500 MHz, $CDCl_3$) δ_H = 7.39 (dd, J = 8.2, 7.2 Hz, 1H, C**6**-<u>H</u>), 7.30 – 7.26 (m, 2H, Ph ArC<u>H</u>), 7.24 – 7.20 (m, 2H, Ph ArC<u>H</u>), 7.19 – 7.14 (m, 1H, Ph ArC<u>H</u>), 6.56 (d, J = 7.2 Hz, 1H, C**7**-<u>H</u>), 6.52 (d, J = 8.2 Hz, 1H, C**5**-<u>H</u>), 3.92 (s, 3H, OC<u>H$_3$</u>), 3.34 (dqd, J = 8.1, 7.0, 6.9 Hz, 1H, C**2**-<u>H</u>), 3.00 (dd, J = 13.4, 6.9 Hz, 1H, C**3**-<u>H</u>), 2.89 (dd, J = 13.4, 8.1 Hz, 1H, C**3**-<u>H’</u>), 1.28 (d, J = 7.0 Hz, 3H, C**1**-<u>H$_3$</u>); ^{13}C NMR (125 MHz, $CDCl_3$) δ_C = 163.7 (C**8**), 158.5 (C**4**), 147.2 (Ph Ar<u>C</u>), 138.6 (C**6**), 128.4 (Ph Ar<u>C</u>H), 127.2 (Ph Ar<u>C</u>H), 126.0 (Ph Ar<u>C</u>H), 116.2 (C**7**), 107.5 (C**5**), 53.3 (O<u>C</u>H$_3$), 46.6 (C**3**), 39.9 (C**2**), 21.7 (C**1**). HRMS (ESI$^+$) calculated for $C_{15}H_{18}NO$ $[M+H]^+$ = 228.1383, found 228.1389.

SFC conditions: CHIRALPACK OJ-H (25 cm), 98:2 CO_2:*i*-PrOH, 1.0 mL/min, 254 nm; *Retention times*: t_{minor} = 15.2 min, t_{major} = 17.0 min.

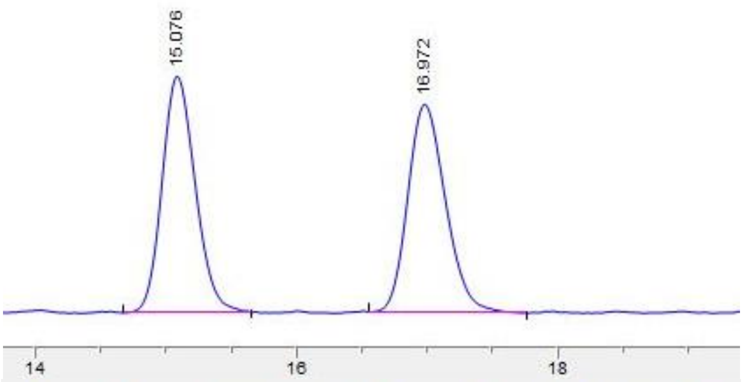

#	Time	Area	Height	Width	Area%	Symmetry
1	15.076	2591.4	145.4	0.2699	49.897	0.798
2	16.972	2602.1	128.3	0.3066	50.103	0.779

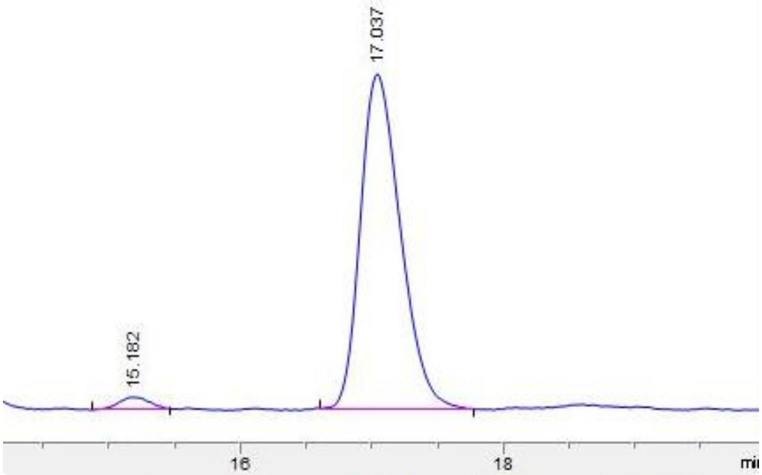

#	Time	Area	Height	Width	Area%	Symmetry
1	15.182	126.2	7.5	0.2015	2.810	0.899
2	17.037	4364.7	204.9	0.3206	97.190	0.702

***tert*-Butyl 2-(5-methoxypyridin-2-yl)acetate (1d)**

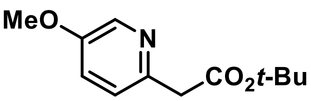

<u>**General procedure D**</u>: 2-Chloro-5-methoxypyridine (287 mg, 2.00 mmol) was employed with *t*-BuXPhos palladium(II) phenethylamine chloride (6.87 mg, 0.01 mmol), *tert*-butyl acetate (349 mg, 402 µL, 3.00 mmol) and LiHMDS (1.0 M in toluene, 6.00 mL, 6.00 mmol). The reaction was stirred for 6 h. Purification by flash column chromatography (Hexane/EtOAc = 6/1 to 3/1) afforded the title compound (77.1 mg, 17%) as a yellow oil. IR (thin film) ν_{max}/cm^{-1}: 2978, 2931, 1595, 1143, 1039, 842. ^{1}H NMR (500 MHz, $CDCl_3$) δ_H = 8.36 (d, J = 5.8 Hz, 1H), 6.81 (d, J = 2.4 Hz, 1H), 6.71 (dd, J = 5.8,

2.4 Hz, 1H), 3.84 (s, 3H), 3.71 (s, 2H), 1.45 (s, 9H); ^{13}C NMR (125 MHz, $CDCl_3$) δ_C = 170.0, 166.3, 156.6, 150.5, 109.8, 108.5, 81.3, 55.2, 45.2, 28.2. HRMS (ESI$^+$) calculated for $C_{12}H_{17}NNaO_3$ [M+Na]$^+$ = 246.1101, found 246.1097.

***tert*-Butyl (3*S*)-2-(5-methoxypyridin-2-yl)-3-phenylbutanoate (3da)**

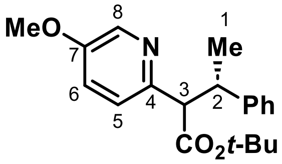

General procedure E: The preceding azaarylacetate (22.3 mg, 0.10 mmol) and styrene (52.1 mg, 57.5 μL, 0.50 mmol) were employed with [Ir(cod)$_2$]BARF (6.36 mg, 0.005 mmol) and **L6** (1.91 mg, 0.005 mmol) in toluene (0.20 mL). The reaction was stirred at 120 °C for 72 h. Purification by flash column chromatography (Hexane/EtOAc = 9/1 to 4/1) afforded the title compounds (16.7 mg, 51%, B:L > 25:1, 2:1 d.r., 95:5 e.r./95:5 e.r.) as colorless oils. IR (thin film) ν_{max}/cm^{-1}: 2924, 2851, 1594, 1453, 1146, 699. Diastereomer 1: ^{1}H NMR (500 MHz, $CDCl_3$) δ_H = 8.41 (d, *J* = 5.8 Hz, 1H, C**8**-H), 7.34 – 7.28 (m, 2H, Ph ArCH), 7.22 – 7.18 (m, 1H, Ph ArCH), 7.14 – 7.02 (m, 2H, Ph ArCH), 7.12 (d, *J* = 2.5 Hz, 1H, C**5**-H), 6.75 (dd, *J* = 5.8, 2.5 Hz, 1H, C**6**-H), 3.92 (d, *J* = 11.5 Hz, 1H, C**3**-H), 3.88 (s, 3H, OCH_3), 3.47 (dq, *J* = 11.5, 6.9 Hz, 1H, C**2**-H), 1.08 (s, 9H, $C(CH_3)_3$), 1.04 (d, *J* = 6.9 Hz, 3H, C**1**-H_3); ^{13}C NMR (125 MHz, $CDCl_3$) δ_C = 171.3 (C=O), 166.4 (C**7**), 159.9 (C**4**), 150.4 (C**8**), 144.8 (Ph ArC), 128.4 (Ph ArCH), 127.9 (Ph ArCH), 126.6 (Ph ArCH), 109.0 (C**5**), 108.5 (C**6**), 80.8 ($C(CH_3)_3$), 62.3 (C**3**), 55.3 (OCH_3), 44.0 (C**2**), 27.7 ($C(CH_3)_3$), 20.6 (C**1**). Diastereomer 2: ^{1}H NMR (500 MHz, $CDCl_3$) δ_H = 8.19 (d, *J* = 5.8 Hz, 1H, C**8**-H), 7.34 – 7.28 (m, 2H, Ph ArCH), 7.14 – 7.02 (m, 3H, Ph ArCH), 6.66 (d, *J* = 2.4 Hz, 1H, C**5**-H), 6.52 (dd, *J* = 5.8, 2.4 Hz, 1H, C**6**-H), 3.89 (d, *J* = 10.9 Hz, 1H, C**3**-H), 3.70 (s, 3H, OCH_3), 3.57 (dq, *J* = 10.9, 6.9 Hz, 1H, C**2**-H), 1.43 (s, 9H, $C(CH_3)_3$), 1.41 (d, *J* = 6.9 Hz, 3H, C**1**-H_3); ^{13}C NMR (125 MHz, $CDCl_3$) δ_C = 171.8 (C=O), 165.7 (C**7**), 159.5 (C**4**), 150.0 (C**8**), 144.0 (Ph ArC), 128.2 (Ph ArCH), 127.8 (Ph ArCH), 126.2 (Ph ArCH), 109.1 (C**5**), 108.4 (C**6**), 81.3 ($C(CH_3)_3$), 62.0 (C**3**), 55.1 (OCH_3), 43.0 (C**2**), 28.2 ($C(CH_3)_3$), 21.0 (C**1**). HRMS (ESI$^+$) calculated for $C_{20}H_{26}NO_3$ [M+H]$^+$ = 328.1907, found 328.1904.

SFC conditions: CHIRALPACK IC (25 cm), 95:5 CO_2:*i*-PrOH, 2.0 mL/min, 210 nm; *Retention times*: *Diastereomer 1:* t_{minor} = 7.0 min, t_{major} = 8.4 min; *Diastereomer 2:* t_{major} = 10.0 min, t_{minor} = 16.4 min.

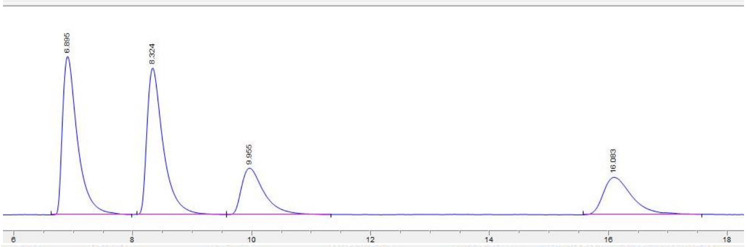

#	Time	Area	Height	Width	Area%	Symmetry
1	6.895	3930.8	223.8	0.2629	34.667	0.471
2	8.324	3920.8	206.9	0.2871	34.579	0.49
3	9.955	1725.3	65.6	0.3909	15.216	0.513
4	16.083	1761.9	52.9	0.4369	15.539	0.558

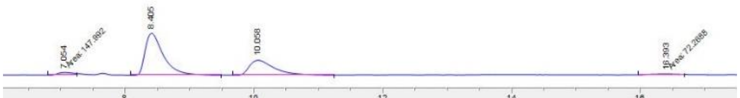

#	Time	Area	Height	Width	Area%	Symmetry
1	7.054	148	8.9	0.278	3.478	0.7
2	8.405	2724.4	138.7	0.2911	64.027	0.507
3	10.058	1310.4	48.9	0.389	30.797	0.495
4	16.393	72.3	2.6	0.4609	1.698	1.255

***tert*-Butyl 2-(5-chloropyridin-2-yl)acetate (1e)**

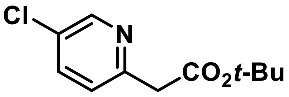

__General procedure D__: 2-Bromo-5-chloropyridine (192 mg, 1.00 mmol) was employed with *t*-BuXPhos palladium(II) phenethylamine chloride (6.87 mg, 0.01 mmol), *tert*-butyl acetate (174 mg, 201 µL, 1.50 mmol) and LiHMDS (1.0 M in toluene, 3.00 mL, 3.00 mmol). The reaction was stirred for 2 h. Purification by flash column chromatography (Hexane/EtOAc = 10/1 to 5/1) afforded the title compound (193 mg, 85%) as a pale-yellow oil. IR (thin film) ν_{max}/cm^{-1}: 2979, 2939, 1726, 1368, 1142, 1014, 823. ^{1}H NMR (400 MHz, $CDCl_3$) δ_H = 8.51 (d, *J* = 2.5 Hz, 1H), 7.63 (dd, *J* = 8.4, 2.5 Hz, 1H), 7.25 (d, *J* = 8.4 Hz, 1H), 3.74 (s, 2H), 1.45 (s, 9H); ^{13}C NMR (101 MHz, $CDCl_3$) δ_C = 169.7, 153.3, 148.3, 136.4, 130.7, 124.8, 81.7, 44.5, 28.2. HRMS (ESI$^+$) calculated for $C_{11}H_{15}ClNO_2$ $[M+H]^+$ = 228.0785, found 228.0780.

(*S*)-5-Chloro-2-(2-phenylpropyl)pyridine (4ea)

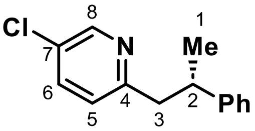

__General procedure E__: The preceding azaarylacetate (22.8 mg, 0.10 mmol) and styrene (52.1 mg, 57.5 µL, 0.50 mmol) were employed with $[Ir(cod)_2]$BARF (6.36 mg, 0.005 mmol) and **L6** (1.91 mg, 0.005 mmol) in toluene (0.20 mL). The reaction was stirred at 100 °C for 24 h. Then *p*-toluenesulfonic acid monohydrate (5.71 mg, 0.03 mmol) was added and the resulting reaction mixture was heated at 130 °C for 3 h. Purification by flash column chromatography (Hexane/EtOAc = 12/1 to 6/1) afforded the title compound (19.5 mg, 84%, B:L > 25:1, 87:13 e.r.) as a colorless oil. $[\alpha]_D^{24}$ = +102.1 (*c* 0.5, CH_2Cl_2). IR (thin film) ν_{max}/cm^{-1}: 2925, 2851, 1460, 1079, 639. ^{1}H NMR (500 MHz, $CDCl_3$) δ_H = 8.50 (d, *J* = 2.5 Hz, 1H, C**8**-H), 7.46 (dd, *J* = 8.2, 2.5 Hz, 1H, C**6**-H), 7.30 – 7.26 (m, 2H, Ph ArCH), 7.21 – 7.16 (m, 3H, Ph ArCH), 6.86 (d, *J* = 8.2 Hz, 1H, C**5**-H), 3.26 (ddq, *J* = 7.7, 7.4, 6.9 Hz, 1H, C**2**-H), 3.06 (dd, *J* = 13.4, 7.4 Hz, 1H, C**3**-H), 3.00 (dd, *J* = 13.4, 7.7 Hz, 1H, C**3**-H'), 1.30 (d, *J* = 6.9 Hz, 3H, C**1**-H_3); ^{13}C NMR (125 MHz, $CDCl_3$) δ_C = 158.9 (C**8**), 148.2 (C**4**), 146.3 (Ph ArC), 135.8 (C**6**), 129.5 (C**7**), 128.5

(Ph Ar<u>C</u>H), 127.1 (Ph Ar<u>C</u>H), 126.3 (Ph Ar<u>C</u>H), 124.5 (C**5**), 46.5 (C**3**), 40.6 (C**2**), 21.6 (C**1**). HRMS (ESI$^+$) calculated for $C_{14}H_{15}ClN$ $[M+H]^+$ = 232.0888, found 232.0887.

SFC conditions: CHIRALPACK OJ-H (25 cm), 97:3 CO_2:*i*-PrOH, 2.0 mL/min, 254 nm; *Retention times*: t_{minor} = 6.8 min, t_{major} = 7.2 min.

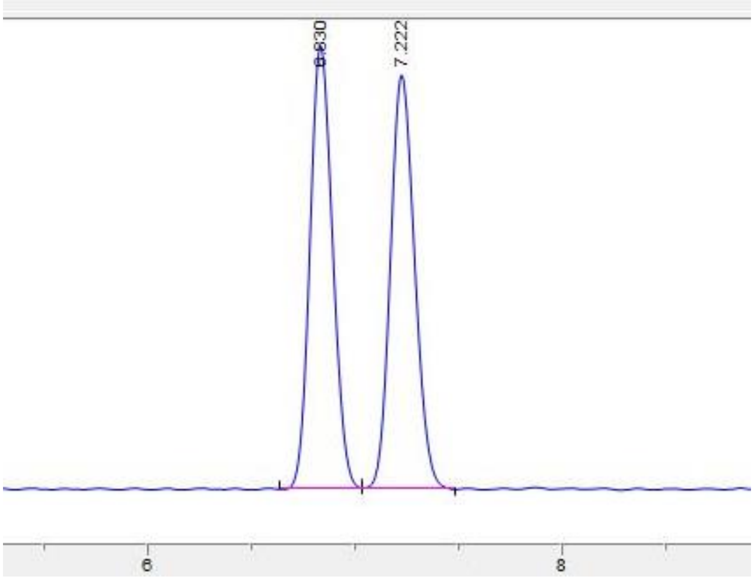

#	Time	Area	Height	Width	Area%	Symmetry
1	6.83	707.8	93.1	0.1187	49.932	0.833
2	7.222	709.8	87	0.1272	50.068	0.828

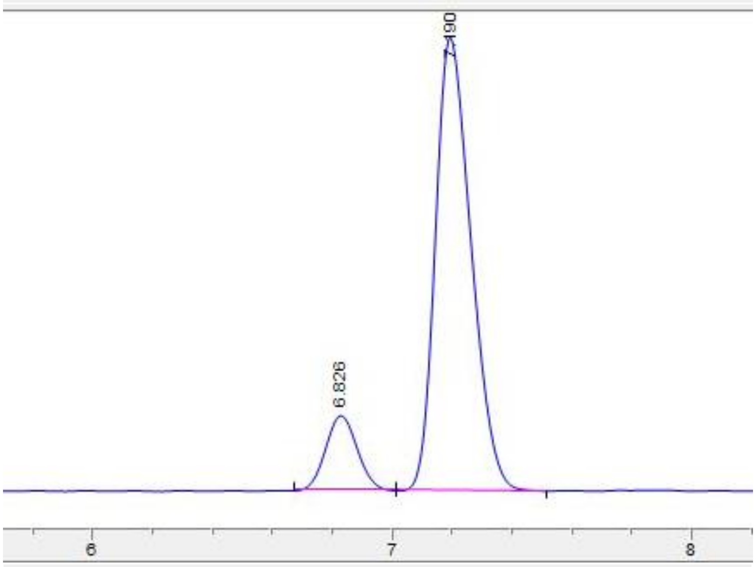

#	Time	Area	Height	Width	Area%	Symmetry
1	6.826	254.9	34.5	0.1163	12.917	0.903
2	7.19	1718.7	207.8	0.1285	87.083	0.671

***tert*-Butyl 2-(4-(trifluoromethyl)pyridin-2-yl)acetate (1f)**

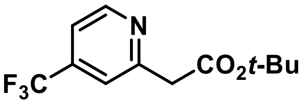

General procedure D: 2-Chloro-4-(trifluoromethyl)pyridine (363 mg, 2.00 mmol) was employed with *t*-BuXPhos palladium(II) phenethylamine chloride (6.87 mg, 0.01 mmol), *tert*-butyl acetate (348 mg, 402 µL, 3.00 mmol) and LiHMDS (1.0 M in toluene, 6.00 mL, 6.00 mmol). The reaction was stirred for 2 h. Purification by flash column chromatography (Hexane/EtOAc = 10/1 to 4/1) afforded the title compound (384 mg, 73%) as a yellow oil. IR (thin film) ν_{max}/cm^{-1}: 2981, 2933, 1730, 1332, 1131, 1038, 843. ^{1}H NMR (400 MHz, $CDCl_3$) δ_H = 8.74 (d, *J* = 5.2 Hz, 1H), 7.53 (s, 1H), 7.41 (d, *J* = 5.2 Hz, 1H), 3.85 (s, 2H), 1.46 (s, 9H); ^{13}C NMR (101 MHz, $CDCl_3$) δ_C = 169.2, 156.7, 150.4, 138.9 (d, $^{2}J_{CF}$ = 34.1 Hz), 122.9 (d, $^{1}J_{CF}$ = 273.2 Hz), 119.8 (q, $^{3}J_{CF}$ = 3.6 Hz), 117.8 (q, $^{3}J_{CF}$ = 3.5 Hz), 81.9, 45.1, 28.2; ^{19}F NMR (376 MHz, $CDCl_3$) δ_F = -64.8 (s, 3F). HRMS (ESI$^+$) calculated for $C_{12}H_{14}F_3NO_2$ [M+H]$^+$ = 262.1049, found 262.1049.

(*S*)-2-(2-Phenylpropyl)-4-(trifluoromethyl)pyridine (4fa)

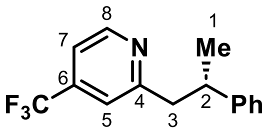

General procedure E: The preceding azaarylacetate (26.1 mg, 0.10 mmol) and styrene (52.1 mg, 57.5 µL, 0.50 mmol) were employed with [Ir(cod)$_2$]BARF (6.36 mg, 0.005 mmol) and **L6** (1.91 mg, 0.005 mmol) in toluene (0.20 mL). The reaction was stirred at 100 °C for 24 h. Then *p*-toluenesulfonic acid monohydrate (5.71 mg, 0.03 mmol) was added and the resulting reaction mixture was heated at 130 °C for 3 h. Purification by flash column chromatography (Hexane/EtOAc = 12/1 to 5/1) afforded the title compound (24.3 mg, 92%, B:L > 25:1, 80:20 e.r.) as a colorless oil. $[\alpha]_D^{23}$ = +69.4 (*c* 0.5, CH_2Cl_2). IR (thin film) ν_{max}/cm^{-1}: 2925, 2851, 1460, 1079, 639. ^{1}H NMR (500 MHz, $CDCl_3$) δ_H = 8.72 (d, *J* = 5.1 Hz, 1H, C**8**-H), 7.30 (d, *J* = 5.1 Hz, 1H, C**7**-H), 7.29 – 7.26 (m, 2H, Ph ArCH), 7.21 – 7.15 (m, 3H, Ph ArCH), 7.10 (s, 1H, C**5**-H), 3.31 (ddq, *J* = 7.9, 7.2, 6.9 Hz, 1H, C**2**-H), 3.15 (dd, *J* = 13.4, 7.2 Hz, 1H, C**3**-H), 3.10 (dd, *J* = 13.4, 7.9 Hz, 1H, C**3**-H’), 1.31 (d, *J* = 6.9 Hz, 3H, C**1**-H_3); ^{13}C NMR (125 MHz, $CDCl_3$) δ_C = 162.4 (C**4**), 150.3 (C**8**), 146.0 (Ph ArC), 138.3 (q, $^{2}J_{CF}$ = 33.8 Hz, C**6**), 128.6 (Ph ArCH), 127.1 (Ph ArCH), 126.5 (Ph ArCH), 122.9 (d, $^{1}J_{CF}$ = 273.0 Hz, CF_3), 119.3 (q, $^{3}J_{CF}$ = 3.6 Hz, C**7**), 116.8 (q, $^{3}J_{CF}$ = 3.5 Hz, C**5**), 47.3 (C**3**), 40.5 (C**2**), 21.5 (C**1**); ^{19}F NMR (471 MHz, $CDCl_3$) δ_F = -64.9 (s, 3F). HRMS (ESI$^+$) calculated for $C_{15}H_{15}F_3N$ [M+H]$^+$ = 266.1151, found 266.1153.

SFC conditions: CHIRALPACK OJ-H (25 cm), 98:2 CO_2:*i*-PrOH, 1.0 mL/min, 254 nm; *Retention times*: t_{minor} = 6.4 min, t_{major} = 7.1 min.

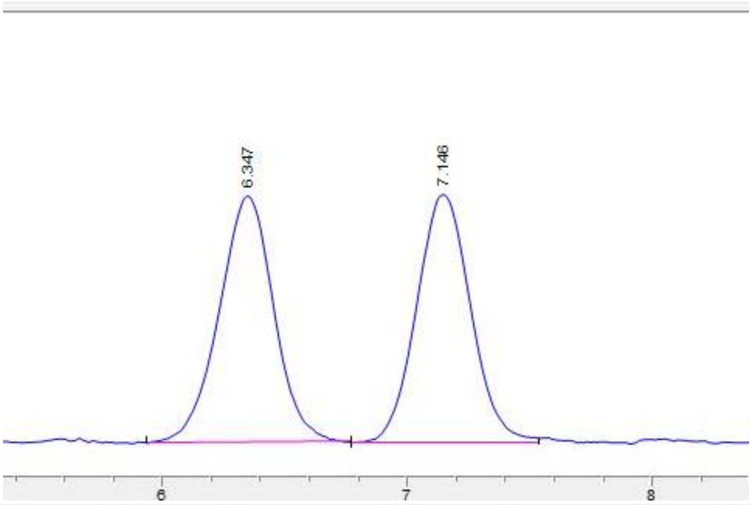

#	Time	Area	Height	Width	Area%	Symmetry
1	6.347	1090.7	69.7	0.2286	50.341	1.071
2	7.146	1075.9	70	0.2234	49.659	0.938

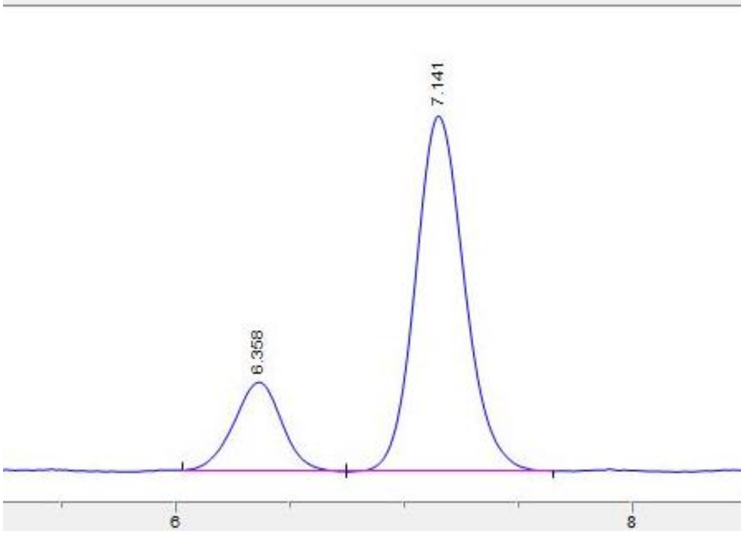

#	Time	Area	Height	Width	Area%	Symmetry
1	6.358	870.6	58.3	0.2202	20.132	1.107
2	7.141	3453.9	231.6	0.226	79.868	0.858

***tert*-Butyl 2-(benzo[*d*]thiazol-2-yl)acetate (1g)**

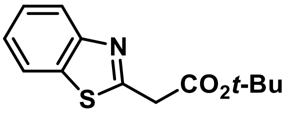

General procedure C: 2-Chlorobenzothiazole (2.04 g, 12.0 mmol) was employed with *tert*-butyl acetate (4.18 g, 4.83 mL, 36.0 mmol) and NaHMDS (0.6 M in toluene, 60.0 mL, 36.0 mmol). Purification by flash column chromatography (Hexane/EtOAc = 20/1 to 9/1) afforded the title compound (2.98 g, 99%) as a yellow solid. ^{1}H NMR (500 MHz, $CDCl_3$) δ_H = 8.01 (d, J = 8.1 Hz, 1H), 7.87 (d, J = 7.8 Hz, 1H), 7.47 (ddd, J = 8.1, 7.4, 1.2 Hz, 1H), 7.38 (ddd, J = 7.8, 7.4, 1.1 Hz, 1H), 4.10 (s, 2H), 1.50 (s, 9H); ^{13}C NMR (125 MHz, $CDCl_3$) δ_C = 167.7, 163.6, 152.6, 135.9, 126.2, 125.3, 123.0, 121.6, 82.7, 41.3, 28.1.

The spectroscopic properties were consistent with the data available in the literature.[4]

(*S*)-2-(2-Phenylpropyl)benzo[*d*]thiazole (4ga)

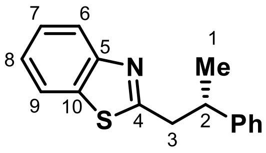

<u>**General procedure E**</u>: The preceding azaarylacetate (24.9 mg, 0.10 mmol) and styrene (15.6 mg, 17.2 μL, 0.15 mmol) were employed with $[Ir(cod)_2]BARF$ (6.36 mg, 0.005 mmol) and **L6** (1.91 mg, 0.005 mmol) in toluene (0.20 mL). The reaction was stirred at 100 °C for 12 h. Then *p*-toluenesulfonic acid monohydrate (5.71 mg, 0.03 mmol) was added and the resulting reaction mixture was heated at 130 °C for 3 h. Purification by flash column chromatography (Hexane/EtOAc = 20/1 to 9/1) afforded the title compound (24.0 mg, 95%, B:L > 25:1, 98:2 e.r.) as a colorless oil. $[\alpha]_D^{25}$ = +106.8 (*c* 0.5, CH_2Cl_2). IR (thin film) ν_{max}/cm^{-1}: 2962, 2926, 1516, 1435, 1125, 759. ^1H NMR (500 MHz, $CDCl_3$) δ_H = 7.99 (d, *J* = 8.2 Hz, 1H, C**6**-<u>H</u>), 7.80 (dd, *J* = 7.9, 1.2 Hz, 1H, C**9**-<u>H</u>), 7.45 (ddd, *J* = 8.2, 7.2, 1.2 Hz, 1H, C**7**-<u>H</u>), 7.36 – 7.33 (m, 1H, C**8**-<u>H</u>), 7.32 – 7.27 (m, 4H, Ph ArC<u>H</u>), 7.25 – 7.19 (m, 1H, Ph ArC<u>H</u>), 3.45 – 3.34 (m, 3H, C**2**-<u>H</u> + C**3**-<u>H</u> + C**3**-<u>H’</u>), 1.39 (d, *J* = 6.6 Hz, 3H, C**1**-$\underline{H_3}$); ^{13}C NMR (125 MHz, $CDCl_3$) δ_C = 170.5 (C**4**), 153.2 (C**5**), 145.6 (Ph Ar<u>C</u>), 135.3 (C**10**), 128.7 (Ph Ar<u>C</u>H), 127.1 (Ph Ar<u>C</u>H), 126.7 (Ph Ar<u>C</u>H), 126.0 (C**7**), 124.8 (C**8**), 122.7 (C**6**), 121.6 (C**9**), 42.8 (C**3**), 40.7 (C**2**), 21.9 (C**1**). HRMS (ESI$^+$) calculated for $C_{16}H_{15}NNaS$ $[M+Na]^+$ = 276.0817, found 276.0813.

SFC conditions: CHIRALPACK SB (25 cm), 95:5 CO_2:*i*-PrOH, 2.0 mL/min, 254 nm; *Retention times*: t_{major} = 9.9 min, t_{minor} = 10.5 min.

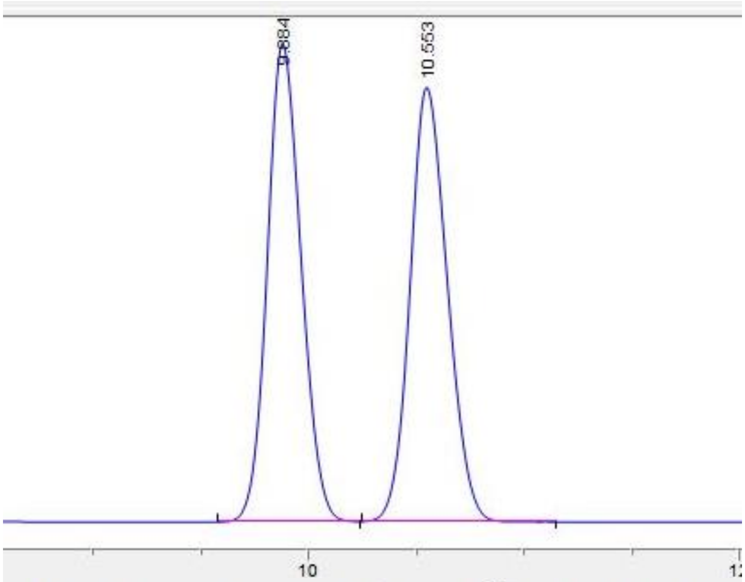

#	Time	Area	Height	Width	Area%	Symmetry
1	9.884	14819.6	1358.9	0.1692	49.915	0.806
2	10.553	14870	1234.1	0.1846	50.085	0.783

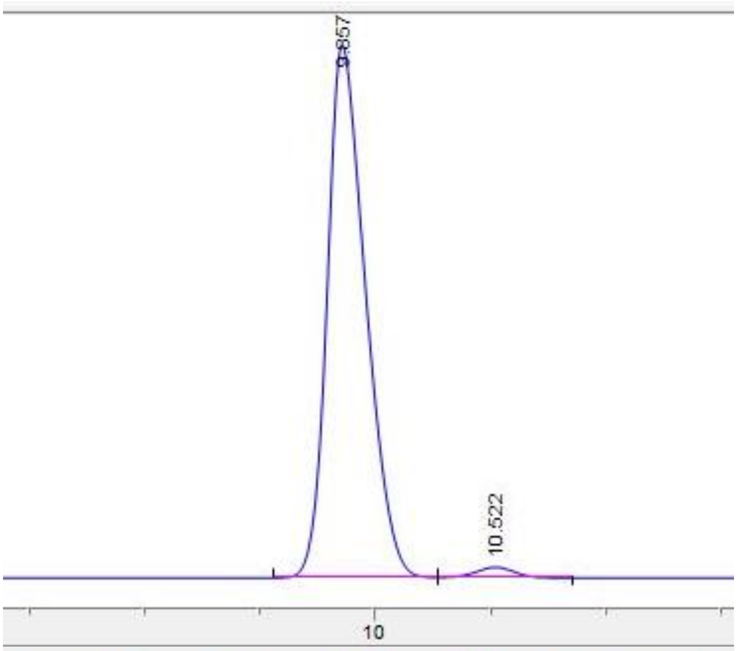

#	Time	Area	Height	Width	Area%	Symmetry
1	9.857	12400.4	1072.9	0.1768	98.007	0.622
2	10.522	252.1	21.2	0.1845	1.993	1.053

***tert*-Butyl 2-(4-phenylthiazol-2-yl)acetate (1h)**

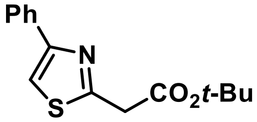

__General procedure D__: 2-Chloro-4-phenylthiazole (587 mg, 3.00 mmol) was employed with *t*-BuXPhos palladium(II) phenethylamine chloride (20.4 mg, 0.03 mmol), *tert*-butyl acetate (523 mg, 603 μL, 4.50 mmol) and LiHMDS (1.0 M in toluene, 9.00 mL, 9.00 mmol). The reaction was stirred for 4 h. Purification by flash column chromatography (Hexane/EtOAc = 9/1 to 4/1) afforded the title compound (138 mg, 17%) as a colorless oil. IR (thin film) ν_{max}/cm^{-1}: 2979, 2925, 1731, 1368, 1151, 736. ^{1}H NMR (500 MHz, $CDCl_3$) δ_H = 7.94 – 7.84 (m, 2H), 7.44 (s, 1H), 7.43 – 7.38 (m, 2H), 7.36 – 7.30 (m, 1H), 4.05 (s, 2H), 1.51 (s, 9H); ^{13}C NMR (125 MHz, $CDCl_3$) δ_C = 168.3, 162.4, 155.0, 134.5, 128.8, 128.1, 126.4, 113.8, 82.3, 40.4, 28.1. HRMS (ESI$^+$) calculated for $C_{15}H_{18}NO_2S$ [M+H]$^+$ = 276.1052, found 276.1048**.**

(*S*)-4-Phenyl-2-(2-phenylpropyl)thiazole (4ha)

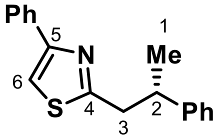

__General procedure E__: The preceding azaarylacetate (27.5 mg, 0.10 mmol) and styrene (31.2 mg, 34.4 μL, 0.30 mmol) were employed with [Ir(cod)$_2$]BARF (6.36 mg, 0.005 mmol) and **L6** (1.91 mg, 0.005 mmol) in toluene (0.20 mL). The reaction was stirred at 100 °C for 72 h. Then *p*-toluenesulfonic acid

monohydrate (5.71 mg, 0.03 mmol) was added and the resulting reaction mixture was heated at 130 °C for 3 h. Purification by flash column chromatography (Hexane/Toluene = 10/1, then Hexane/EtOAc = 10/1 to 5/1) afforded the title compound (25.4 mg, 91%, B:L > 25:1, 99:1 e.r.) as a colorless oil. $[\alpha]_D^{24}$ = +86.3 (c 0.5, CH_2Cl_2). IR (thin film) ν_{max}/cm^{-1}: 2921, 2850, 1494, 1445, 1026, 731. ^{1}H NMR (500 MHz, $CDCl_3$) δ_H = 7.93 – 7.86 (m, 2H, Aza-Ph ArCH), 7.46 – 7.40 (m, 2H, Aza-Ph ArCH), 7.39 – 7.30 (m, 3H, Aza-Ph ArCH + 2 × Ph ArCH), 7.29 – 7.26 (m, 2H, Ph ArCH), 7.28 (s, 1H, C**6**-H), 7.25 – 7.20 (m, 1H, Ph ArCH), 3.40 – 3.32 (m, 3H, C**2**-H + C**3**-H + C**3**-H'), 1.40 (d, J = 6.3 Hz, 3H, C**1**-H_3); ^{13}C NMR (125 MHz, $CDCl_3$) δ_C = 169.3 (C**4**), 155.1 (C**5**), 145.8 (Ph ArC), 134.8 (Aza-Ph ArC), 128.8 (Aza-Ph ArCH), 128.6 (Ph ArCH), 128.1 (Aza-Ph ArCH), 127.2 (Ph ArCH), 126.6 (Ph ArCH), 126.5 (Aza-Ph ArCH), 112.3 (C**6**), 42.1 (C**3**), 40.9 (C**2**), 21.9 (C**1**). HRMS (ESI$^+$) calculated for $C_{18}H_{17}NNaS$ $[M+Na]^+$ = 302.0974, found 302.0976.

SFC conditions: CHIRALPACK SB (25 cm), 95:5 CO_2:*i*-PrOH, 2.0 mL/min, 254 nm; *Retention times*: t_{major} = 19.7 min, t_{minor} = 22.8 min.

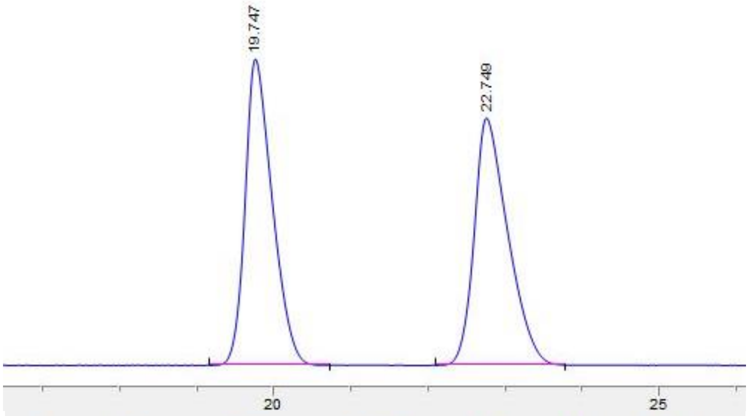

#	Time	Area	Height	Width	Area%	Symmetry
1	19.747	7094.4	288.5	0.3729	50.075	0.594
2	22.749	7073.2	232.7	0.4426	49.925	0.561

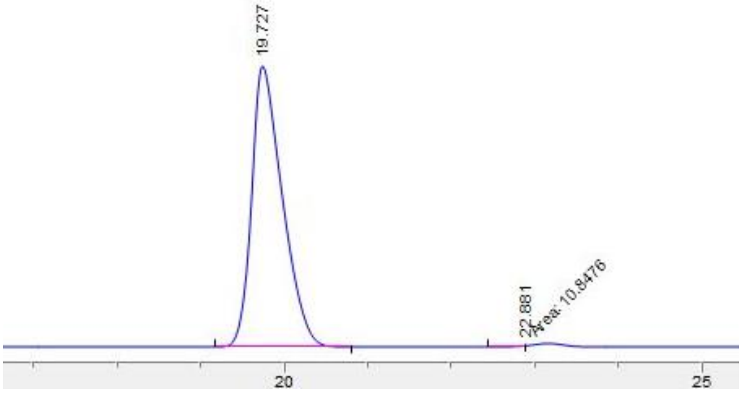

#	Time	Area	Height	Width	Area%	Symmetry
1	19.727	9233.3	360.8	0.3765	99.883	0.543
2	22.881	10.8	1.4	0.1247	0.117	1680.09

tert-Butyl 2-(thiazol-2-yl)acetate (1i)

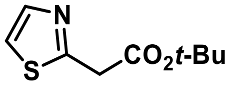

<u>General procedure C</u>: 2-Chlorobenzothiazole (478 mg, 4.0 mmol) was employed with *tert*-butyl acetate (1.39 g, 1.61 mL, 12.0 mmol) and NaHMDS (0.6 M in toluene, 20.0 mL, 12.0 mmol). Purification by flash column chromatography (Hexane/EtOAc = 9/1 to 6/1) afforded the title compound (110 mg, 23%) as a yellow oil. IR (thin film) ν_{max}/cm^{-1}: 2979, 2925, 1731, 1368, 1151, 736. ^{1}H NMR (500 MHz, $CDCl_3$) δ_H = 7.76 (d, J = 3.4 Hz, 1H), 7.33 (d, J = 3.4 Hz, 1H), 4.03 (s, 2H), 1.50 (s, 9H); ^{13}C NMR (125 MHz, $CDCl_3$) δ_C = 168.3, 162.5, 142.2, 120.0, 82.3, 40.1, 28.1. HRMS (ESI$^+$) calculated for $C_9H_{13}NNaO_2S$ [M+Na]$^+$ = 222.0559, found 222.0555.

(*S*)-2-(2-Phenylpropyl)thiazole (4ia)

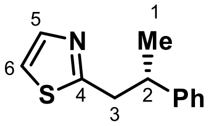

<u>General procedure E</u>: The preceding azaarylacetate (19.9 mg, 0.10 mmol) and styrene (31.2 mg, 34.4 µL, 0.30 mmol) were employed with [Ir(cod)$_2$]BARF (6.36 mg, 0.005 mmol) and **L6** (1.91 mg, 0.005 mmol) in toluene (0.20 mL). The reaction was stirred at 100 °C for 72 h. Then *p*-toluenesulfonic acid monohydrate (5.71 mg, 0.03 mmol) was added and the resulting reaction mixture was heated at 130 °C for 2 h. Purification by flash column chromatography (Hexane/EtOAc = 10/1 to 9/1) afforded the title compound (17.7 mg, 87%, B:L > 25:1, 75:25 e.r.) as a colorless oil. $[\alpha]_D^{24}$ = +37.8 (c 0.5, CH_2Cl_2). IR (thin film) ν_{max}/cm^{-1}: 2972, 2866, 1445, 1054, 664. ^{1}H NMR (500 MHz, $CDCl_3$) δ_H = 7.67 (d, J = 3.3 Hz, 1H, C**5**-<u>H</u>), 7.34 – 7.27 (m, 2H, Ph ArC<u>H</u>), 7.25 – 7.18 (m, 3H, Ph ArC<u>H</u>), 7.13 (d, J = 3.3 Hz, 1H, C**6**-<u>H</u>), 3.36 – 3.25 (m, 3H, C**2**-<u>H</u> + C**3**-<u>H</u> + C**3**-<u>H’</u>), 1.34 (d, J = 6.6 Hz, 3H, C**1**-<u>H$_3$</u>); ^{13}C NMR (125 MHz, $CDCl_3$) δ_C = 169.5 (C**4**), 145.7 (C**5**), 142.2 (Ph Ar<u>C</u>), 128.6 (Ph Ar<u>C</u>H), 127.1 (Ph Ar<u>C</u>H), 126.6 (Ph ArCH), 118.4 (C**6**), 41.8 (C**3**), 41.0 (C**2**), 21.7 (C**1**). HRMS (ESI$^+$) calculated for $C_{12}H_{14}NS$ [M+H]$^+$ = 204.0841, found 204.0845.

SFC conditions: CHIRALPACK SB (25 cm), 95:5 CO_2:*i*-PrOH, 2.0 mL/min, 254 nm; *Retention times*: t_{minor} = 4.7 min, t_{major} = 5.1 min.

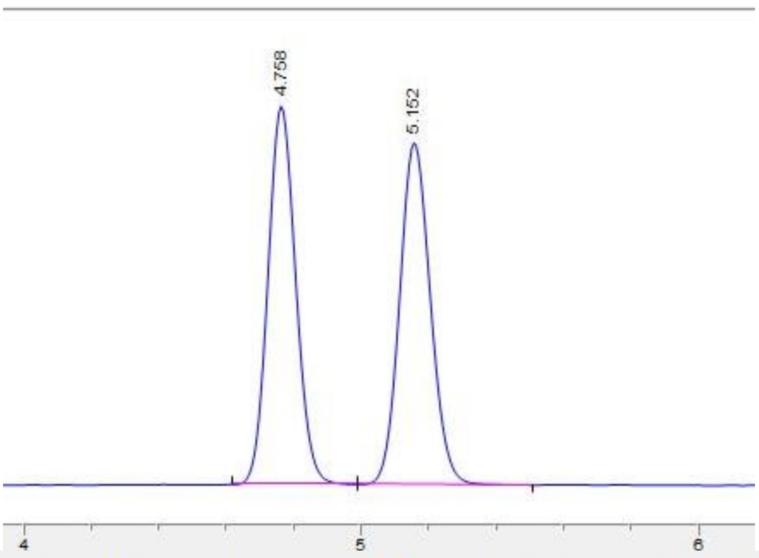

#	Time	Area	Height	Width	Area%	Symmetry
1	4.758	548.5	96.3	0.0884	49.876	0.88
2	5.152	551.2	87.1	0.0979	50.124	0.864

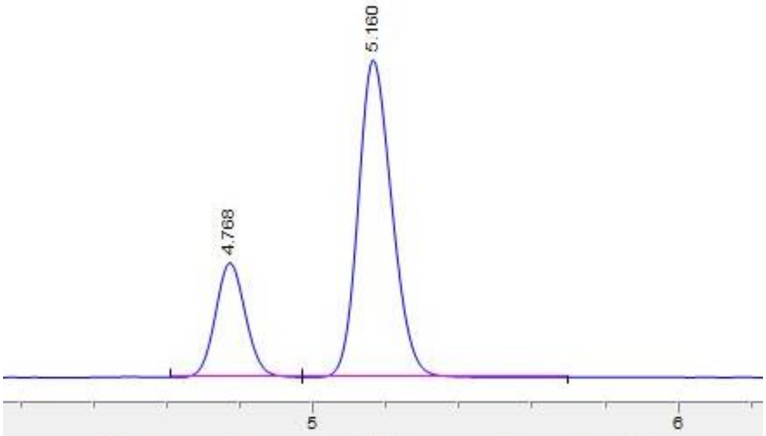

#	Time	Area	Height	Width	Area%	Symmetry
1	4.768	532.2	92.6	0.0891	24.476	0.891
2	5.16	1642.3	256.4	0.0987	75.524	0.767

***tert*-Butyl 2-(benzo[*d*]oxazol-2-yl)acetate (1j)**

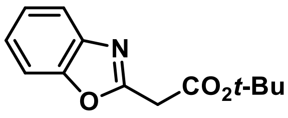

<u>**General procedure A**</u>: 2-Methylbenzo[*d*]oxazole (1.33 g, 10.0 mmol) was employed with diisopropylamine (1.68 mL, 12.0 mmol), *n*-BuLi (2.5 M in hexane, 4.80 mL, 12.0 mmol) and dibutyldicarbonate (2.62 g, 12.0 mmol). Purification by flash column chromatography (Hexane/EtOAc = 20/1 to 9/1) afforded the title compound (614 mg, 25%) as a colorless solid. m.p. 85 - 88 °C (EtOAc/hexane). IR (thin film) ν_{max}/cm^{-1}: 2984, 2937, 1723, 1578, 1455, 1148, 751. ^{1}H NMR (500 MHz, $CDCl_3$) δ_H = 7.79 – 7.65 (m, 1H), 7.58 – 7.47 (m, 1H), 7.37 – 7.28 (m, 2H), 3.94 (s, 2H), 1.47 (s,

9H); ^{13}C NMR (125 MHz, $CDCl_3$) δ_C = 166.3, 160.2, 151.3, 141.3, 125.2, 124.5, 120.1, 110.7, 82.8, 36.6, 28.1. HRMS (ESI$^+$) calculated for $C_{13}H_{15}NNaO_3$ [M+Na]$^+$ = 256.0944, found 256.0950.

(*S*)-2-(2-Phenylpropyl)benzo[*d*]oxazole (4ja)

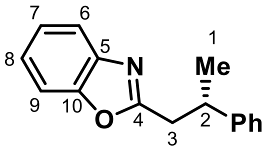

General procedure E: The preceding azaarylacetate (24.9 mg, 0.10 mmol) and styrene (31.2 mg, 34.4 μL, 0.30 mmol) were employed with [Ir(cod)$_2$]BARF (6.36 mg, 0.005 mmol) and **L6** (1.91 mg, 0.005 mmol) in toluene (0.20 mL). The reaction was stirred at 100 °C for 24 h. Then *p*-toluenesulfonic acid monohydrate (5.71 mg, 0.03 mmol) was added and the resulting reaction mixture was heated at 130 °C for 3 h. Purification by flash column chromatography (Hexane/EtOAc = 30/1 to 9/1) afforded the title compound (22.0 mg, 93%, B:L > 25:1, 96.5:3.5 e.r.) as a colorless oil. $[\alpha]_D^{24}$ = +83.4 (*c* 0.5, CH_2Cl_2). IR (thin film) ν_{max}/cm^{-1}: 2964, 2927, 1570, 1455, 1242, 744. ^{1}H NMR (500 MHz, $CDCl_3$) δ_H = 7.71 – 7.63 (m, 1H, C**6**-H), 7.49 – 7.45 (m, 1H, C**9**-H), 7.34 – 7.27 (m, 6H, C**7**-H + C**8**-H + 4 × Ph ArCH), 7.24 – 7.19 (m, 1H, Ph ArCH), 3.51 (dqd, *J* = 9.0, 6.9, 6.2 Hz, 1H, C**2**-H), 3.25 (dd, *J* = 15.0, 6.2 Hz, 1H, C**3**-H), 3.15 (dd, *J* = 15.0, 9.0 Hz, 1H, C**3**-H'), 1.38 (d, *J* = 6.9 Hz, 3H, C**1**-H_3); ^{13}C NMR (125 MHz, $CDCl_3$) δ_C = 165.9 (C**4**), 150.9 (C**5**), 145.5 (Ph ArC), 141.4 (C**10**), 128.8 (Ph ArCH), 126.8 (Ph ArCH), 126.7 (Ph ArCH), 124.6 (C**7**), 124.2 (C**8**), 119.7 (C**6**), 110.4 (C**9**), 38.3 (C**3**), 37.4 (C**2**), 21.7 (C**1**). HRMS (ESI$^+$) calculated for $C_{16}H_{16}NO$ [M+H]$^+$ = 238.1226, found 238.1227.

SFC conditions: CHIRALPACK SB (25 cm), 98:2 CO_2:MeOH, 1.5 mL/min, 254 nm; *Retention times*: t_{minor} = 9.3 min, t_{major} = 9.7 min.

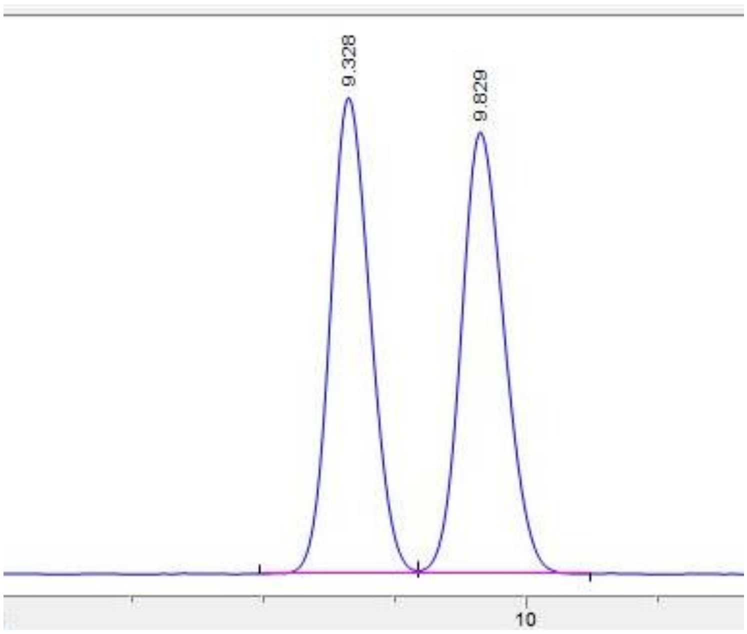

#	Time	Area	Height	Width	Area%	Symmetry
1	9.328	2263.8	215	0.1648	49.948	0.821
2	9.829	2268.5	199.5	0.1747	50.052	0.783

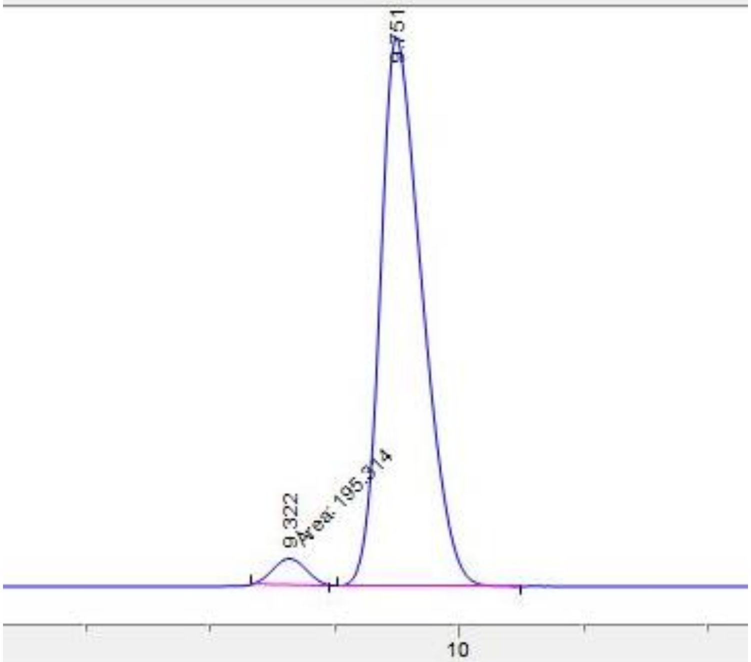

#	Time	Area	Height	Width	Area%	Symmetry
1	9.322	195.3	21	0.1553	3.655	0.916
2	9.751	5147.9	440.7	0.1783	96.345	0.601

***tert*-Butyl 2-(1*H*-benzo[*d*]imidazol-2-yl)acetate**

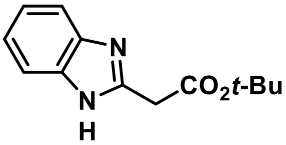

A solution of 3-*tert*-butoxy-3-oxopropanoic acid (3.20 g, 20.0 mmol) and 1,2-phenylenediamine (2.16 g, 20.0 mmol) in acetonitrile (20.0 mL) was added a solution of DCC (4.54 g, 22.0 mmol) in acetonitrile (10.0 mL), and the reaction was stirred for 20 min at room temperature. The white suspension was filtered and the filtrate was evaporated to leave a dark yellow oil which was purified by flash column chromatography (Hexane/EtOAc = 2/1 to 1/1) followed by recrystallization from a mixture of hexane/EtOAc (1/1) to give *tert*-butyl 3-(2-aminophenylamino)-3-oxopropanoate (2.63 g, 53%) as a white crystalline solid.

The preceding amide (2.63 g, 10.5 mmol) was dissolved in acetic acid (5.20 mL), and the resulting reaction mixture was heated to 90 °C for 1 h. After cooling to room temperature, the solvent was evaporated and the residue was recrystallized from a mixture of hexane/EtOAc to give the title compound (2.37 g, 97%) as a white crystalline solid. ^{1}H NMR (500 MHz, $CDCl_3$) δ_H = 7.58 (dd, J = 6.0, 3.2 Hz, 2H), 7.24 (dd, J = 6.0, 3.2 Hz, 2H), 6.49 (br s, 1H), 4.00 (s, 2H), 1.51 (s, 9H); ^{13}C NMR (125 MHz, $CDCl_3$) δ_C = 169.4, 147.7, 138.1, 122.7, 115.1, 83.1, 35.5, 28.2.

The spectroscopic properties were consistent with the data available in the literature.[5]

tert-Butyl 2-(1-methyl-1*H*-benzo[*d*]imidazol-2-yl)acetate (1k)

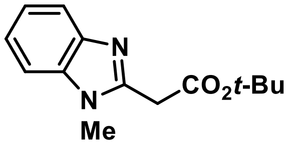

A mixture of *tert*-Butyl 2-(1*H*-benzo[*d*]imidazol-2-yl)acetate (584 mg, 2.52 mmol) and K_2CO_3 (522 mg, 3.78 mmol) in dry DMF (5.00 mL) was added MeI (537 mg, 235 µL, 3.78 mmol) dropwise at 0 °C. The reaction was stirred for 15 h at 20 °C. The reaction was quenched with water (25 mL) and extracted with EtOAc (4 × 25 mL). The combined organic phase was washed with brine (25 mL), dried over Na_2SO_4, filtered and concentrated *in vacuo*. The residue was purified by flash column chromatography (Hexane/EtOAc = 5/1 to 1/1) to give the title compound (300 mg, 48%) as a colorless solid. m.p. 94 - 96 °C (EtOAc/hexane). IR (thin film) ν_{max}/cm^{-1}: 2979, 2941, 1729, 1476, 1334, 1149, 743. ^{1}H NMR (500 MHz, $CDCl_3$) δ_H = 7.74 – 7.70 (m, 1H), 7.34 – 7.30 (m, 1H), 7.30 – 7.23 (m, 2H), 3.95 (s, 2H), 3.76 (s, 3H), 1.45 (s, 9H); ^{13}C NMR (125 MHz, $CDCl_3$) δ_C = 167.5, 148.5, 142.4, 136.1, 122.7, 122.2, 119.7, 109.4, 82.5, 35.8, 30.4, 28.1. HRMS (ESI$^+$) calculated for $C_{14}H_{18}N_2NaO_2$ $[M+Na]^+$ = 269.1260, found 269.1262.

tert-Butyl (3*S*)-2-(1-methyl-1*H*-benzo[*d*]imidazol-2-yl)-3-phenylbutanoate (3ka)

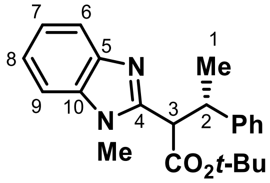

<u>**General procedure E**</u>: The preceding azaarylacetate (24.6 mg, 0.10 mmol) and styrene (52.1 mg, 57.5 µL, 0.50 mmol) were employed with $[Ir(cod)_2]$BARF (6.36 mg, 0.005 mmol) and **L6** (1.91 mg, 0.005 mmol) in toluene (0.20 mL). The reaction was stirred at 120 °C for 96 h. Purification by flash column chromatography (Hexane/EtOAc = 10/1 to 4/1) afforded the title compounds (1.5:1 d.r., B:L > 25:1; diastereomer 1: 9.20 mg, 26%, 98:2 e.r.; diastereomer 2: 6.00 mg, 17%, 98.5:1.5 e.r.) as colorless solids.

Diastereomer 1: $[\alpha]_D^{24}$ = +40.2 (*c* 0.2, CH_2Cl_2). m.p. 141 - 144 °C (EtOAc/hexane). IR (thin film) ν_{max}/cm^{-1}: 2973, 2921, 1728, 1462, 1139, 743. ^{1}H NMR (500 MHz, $CDCl_3$) δ_H = 7.86 (dd, *J* = 6.8, 2.1 Hz, 1H, C**9**-<u>H</u>), 7.42 – 7.27 (m, 7H, C**6**-<u>H</u> + C**7**-<u>H</u> + C**8**-<u>H</u> + 4 × Ph ArC<u>H</u>), 7.26 – 7.21 (m, 1H, Ph ArC<u>H</u>), 4.18 (d, *J* = 11.0 Hz, 1H, C**3**-<u>H</u>), 4.03 (dq, *J* = 11.0, 6.9 Hz, 1H, C**2**-<u>H</u>), 3.88 (s, 3H, NC<u>H₃</u>), 1.24 (d, *J* = 6.9 Hz, 3H, C**1**-<u>H₃</u>), 1.09 (s, 9H, C(C<u>H₃</u>)₃); ^{13}C NMR (125 MHz, $CDCl_3$) δ_C = 168.5 (<u>C</u>=O), 150.7 (C**4**), 144.2 (Ph Ar<u>C</u>), 142.5 (C**5**), 135.9 (C**10**), 128.5 (Ph Ar<u>C</u>H), 127.9 (Ph Ar<u>C</u>H), 126.9 (Ph Ar<u>C</u>H), 122.7 (C**8**), 122.3 (C**7**), 120.0 (C**9**), 109.4 (C**6**), 82.0 (<u>C</u>(CH₃)₃), 53.2 (C**3**), 41.2 (C**2**), 30.3 (N<u>C</u>H₃), 27.6 (C(<u>C</u>H₃)₃), 21.2 (C**1**). HRMS (ESI$^+$) calculated for $C_{22}H_{27}N_2O_2$ $[M+H]^+$ = 351.2067, found 351.2069.

SFC conditions: CHIRALPACK IC (25 cm), 90:10 CO_2:*i*-PrOH, 2.0 mL/min, 254 nm; *Retention times*: t_{minor} = 6.4 min, t_{major} = 8.6 min.

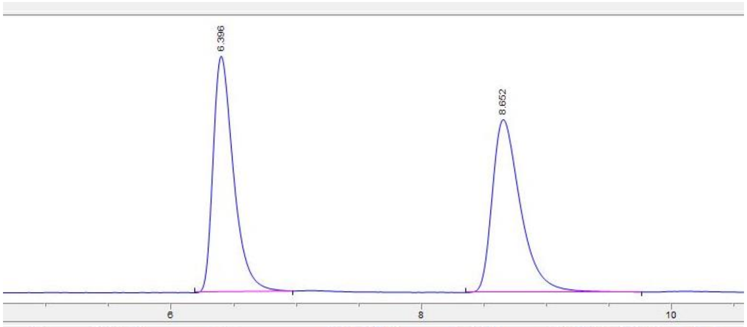

#	Time	Area	Height	Width	Area%	Symmetry
1	6.396	1266.1	111.2	0.1728	49.643	0.607
2	8.652	1284.3	81.4	0.24	50.357	0.586

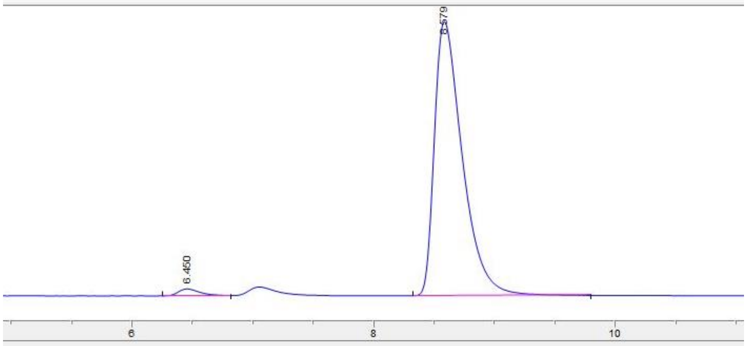

#	Time	Area	Height	Width	Area%	Symmetry
1	6.45	72.7	5.7	0.1937	1.985	0.596
2	8.579	3590.9	223.2	0.2457	98.015	0.508

Diastereomer 2: $[\alpha]_D^{24}$ = +82.4 (*c* 0.2, CH_2Cl_2). m.p. 110 - 112 °C (EtOAc/hexane). IR (thin film) ν_{max}/cm^{-1}: 2974, 2927, 1738, 1468, 1150, 745. ^{1}H NMR (500 MHz, $CDCl_3$) δ_H = 7.80 – 7.72 (m, 1H, C**9**-H), 7.23 – 7.16 (m, 2H, C**6**-H + C**8**-H), 7.16 – 7.10 (m, 3H, C**7**-H + 2 × Ph ArCH), 7.08 (m, 2H, Ph ArCH), 7.05 – 7.00 (m, 1H, Ph ArCH), 4.01 (d, *J* = 10.5 Hz, 1H, C**3**-H), 3.95 (dd, *J* = 10.5, 6.8 Hz, 1H, C**2**-H), 3.38 (s, 3H, NCH_3), 1.54 (d, *J* = 6.8 Hz, 3H, C**1**-H_3), 1.44 (s, 9H, $C(CH_3)_3$); ^{13}C NMR (125 MHz, $CDCl_3$) δ_C = 167.9 (C=O), 150.9 (C**4**), 146.2 (Ph ArC), 143.6 (C**5**), 131.0 (C**10**), 128.4 (Ph ArCH), 127.5 (Ph ArCH), 126.7 (Ph ArCH), 122.3 (C**8**), 122.0 (C**7**), 119.9 (C**9**), 109.2 (C**6**), 82.3($C(CH_3)_3$), 53.1 (C**3**), 41.4 (C**2**), 29.7 (NCH_3), 28.2 ($C(CH_3)_3$, 20.3 (C**1**). HRMS (ESI^+) calculated for $C_{22}H_{27}N_2O_2$ $[M+H]^+$ = 351.2067, found 351.2070.

SFC conditions: CHIRALPACK SC (25 cm), 90:10 CO_2:*i*-PrOH, 2.0 mL/min, 254 nm; *Retention times*: t_{major} = 6.9 min, t_{minor} = 10.0 min.

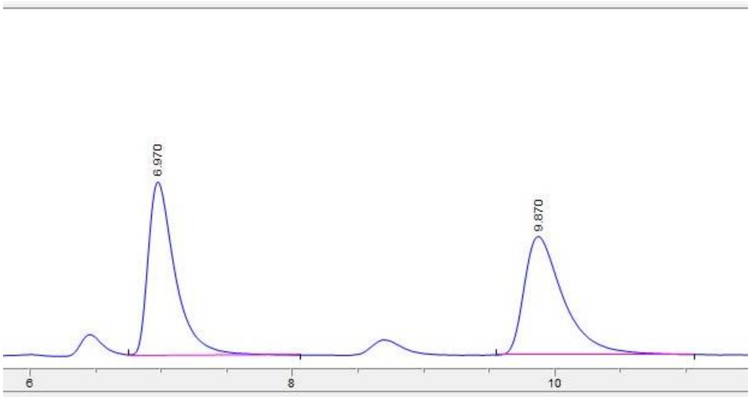

#	Time	Area	Height	Width	Area%	Symmetry
1	6.97	946.8	65.5	0.2146	50.408	0.513
2	9.87	931.5	44.6	0.3118	49.592	0.507

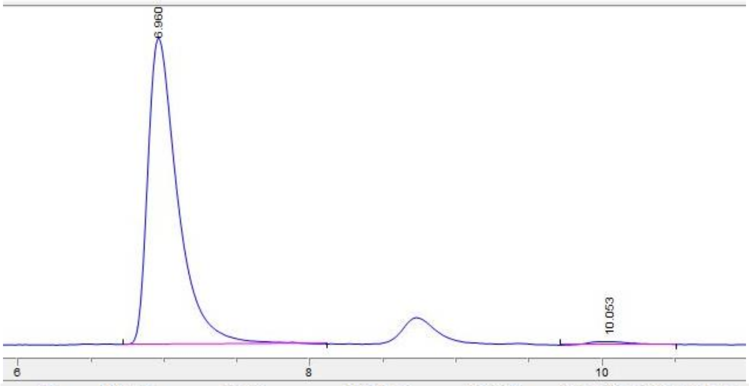

#	Time	Area	Height	Width	Area%	Symmetry
1	6.96	1563	109.3	0.2149	98.421	0.498
2	10.053	25.1	1.2	0.2637	1.579	0.902

(*S*)-1-Methyl-2-(2-phenylpropyl)-1*H*-benzo[*d*]imidazole (4ka)

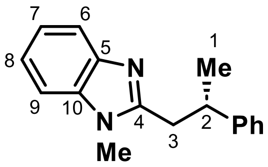

The preceding *tert*-butyl (3*S*)-2-(1-methyl-1*H*-benzo[*d*]imidazol-2-yl)-3-phenylbutanoate (10.0 mg, 28.6 µmol) was dissolved in DMSO (0.20 mL), and the resulting solution was stirred at 150 °C for 4 h. The solvent was removed under vacuum. The residue was purified by flash column chromatography (Hexane/EtOAc = 5/1 to 3/1) to give the title compound (6.40 mg, 90%, 97.5:2.5 e.r.) as a colorless oil. *The yield of the title compound was 39% (over two steps) starting from the corresponding azaarylacetate and styrene.* $[\alpha]_D^{25}$ = +126.0 (*c* 0.1, CH_2Cl_2). IR (thin film) ν_{max}/cm^{-1}: 2962, 2923, 1602, 1507, 1467, 1398, 1286, 742. ^{1}H NMR (500 MHz, $CDCl_3$) δ_H = 7.77 – 7.73 (m, 1H, C**9**-H), 7.30 – 7.17 (m, 8H, C**6**-H + C**7**-H + C**8**-H + 5 × Ph ArCH), 3.51 – 3.42 (m, 1H, C**2**-H), 3.39 (s, 3H, NCH_3), 3.17 (dd, *J* = 14.4, 7.8 Hz, 1H, C**3**-H), 3.09 (dd, *J* = 14.4, 6.7 Hz, 1H, C**3**-H’), 1.43 (d, *J* = 6.9 Hz, 3H, C**1**-H_3); ^{13}C NMR (125 MHz, $CDCl_3$) δ_C = 154.0 (C**4**), 145.9 (Ph ArC), 142.6 (C**5**), 135.6 (C**10**), 128.7 (Ph ArCH), 127.0 (Ph ArCH), 126.7 (Ph ArCH), 122.1 (C**8** or C**7**), 122.0 (C**7** or C**8**), 119.3 (C**9**), 109.2

(C**6**), 39.7 (C**2**), 36.7 (C**3**), 29.6 (N<u>C</u>H$_3$), 21.1 (C**1**). HRMS (ESI$^+$) calculated for $C_{17}H_{18}N_2Na$ [M+Na]$^+$ = 273.1362, found 273.1363.

SFC conditions: CHIRALPACK SB (25 cm), 85:15 CO_2:*i*-PrOH, 2.0 mL/min, 254 nm; *Retention times*: t_{major} = 4.5 min, t_{minor} = 4.9 min.

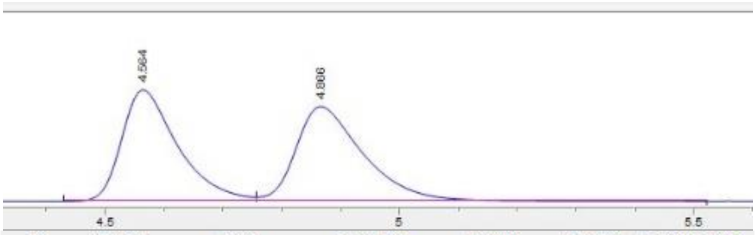

#	Time	Area	Height	Width	Area%	Symmetry
1	4.564	1191.1	180.1	0.0992	49.378	0.576
2	4.866	1221.1	153.1	0.1171	50.622	0.548

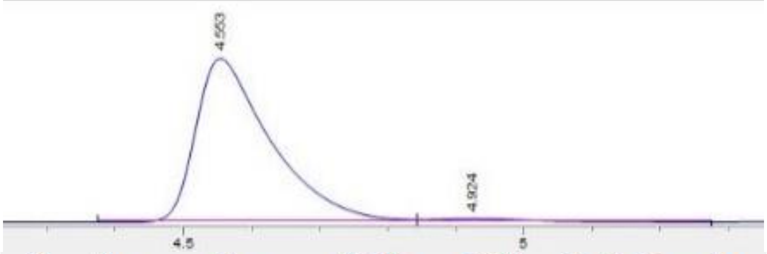

#	Time	Area	Height	Width	Area%	Symmetry
1	4.553	2880.3	374	0.1119	97.435	0.448
2	4.924	75.8	7.6	0.1461	2.565	0.613

***tert*-Butyl 2-(5-bromopyrimidin-2-yl)acetate (1l)**

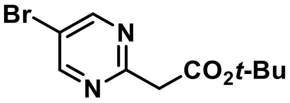

NaH (60% dispersion in mineral oil, 800 mg, 20.0 mmol) was added portion wise under N_2 to a stirred solution of *tert*-butyl methyl malonate (3.48 g, 3.38 mL, 20.0 mmol) in DMF (10.0 mL). The orange solution was stirred at room temperature for 15 min and 5-bromo-2-chloropyrimidine (1.93 g, 10.0 mmol) was added. The reaction mixture was heated at 80 °C overnight, then cooled to 10 °C and 20 mL of a saturated aq. NH_4Cl was added dropwise. The pH was adjusted to 3 by the addition of a 1 N aq. HCl solution and the aqueous phase was extracted with diethyl ether (3 × 80 mL). The combined organic phase was washed with brine (50 mL), dried over Na_2SO_4, filtered and concentrated *in vacuo.* Methyl *tert*-butyl 2-(5-bromopyrimidin-2-yl)propanedioate was afforded as an orange oil, which was used in the next step without any further purification.

A solution of crude methyl *tert*-butyl 2-(5-bromopyrimidin-2-yl)propanedioate (~10.0 mol) and NaOH (800 mg, 20.0 mmol) in a mixture of water (30 mL) and methanol (60 mL) was stirred at room temperature for 4 h. A 2 N HCl aq. solution was added until pH 5, and the mixture was washed with diethyl ether (3 × 50 mL). The combined organic phase was washed with brine (50 mL), dried over Na_2SO_4, filtered and concentrated *in vacuo.* The residue was purified by flash column chromatography

(Hexane/EtOAc = 12/1 to 9/1) to give the title compound (1.18 g, 43% over two steps) as a white solid. ^{1}H NMR (400 MHz, $CDCl_3$) δ_H = 8.76 (s, 2H), 3.91 (s, 2H), 1.46 (s, 9H); ^{13}C NMR (101 MHz, $CDCl_3$) δ_C = 168.7, 163.3, 158.1, 118.8, 81.9, 46.0, 28.2.

The spectroscopic properties were consistent with the data available in the literature.[6]

(*S*)-5-Bromo-2-(2-phenylpropyl)pyrimidine (4la)

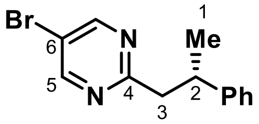

<u>General procedure E</u>: The preceding azaarylacetate (27.3 mg, 0.10 mmol) and styrene (31.2 mg, 34.4 μL, 0.30 mmol) were employed with $[Ir(cod)_2]$BARF (6.36 mg, 0.005 mmol) and **L6** (1.91 mg, 0.005 mmol) in toluene (0.20 mL). The reaction was stirred at 100 °C for 24 h. Then *p*-toluenesulfonic acid monohydrate (5.71 mg, 0.03 mmol) was added and the resulting reaction mixture was heated at 130 °C for 3 h. Purification by flash column chromatography (Hexane/EtOAc = 12/1 to 5/1) afforded the title compound (25.0 mg, 90%, B:L > 25:1, 77:23 e.r.) as a colorless solid. $[\alpha]_D^{22}$ = +79.2 (*c* 0.5, CH_2Cl_2). m.p. 70 - 72 °C (EtOAc/hexane). IR (thin film) ν_{max}/cm^{-1}: 2920, 2851, 1539, 1422, 1116, 699. ^{1}H NMR (500 MHz, $CDCl_3$) δ_H = 8.67 (s, 2H, C**5**-<u>H</u>), 7.29 – 7.21 (m, 4H, Ph ArC<u>H</u>), 7.19 – 7.14 (m, 1H, Ph ArC<u>H</u>), 3.46 (ddq, *J* = 8.2, 7.2, 6.9 Hz, 1H, C**2**-<u>H</u>), 3.24 (dd, *J* = 13.8, 7.2 Hz, 1H, C**3**-<u>H</u>), 3.15 (dd, *J* = 13.8, 8.2 Hz, 1H, C**3**-<u>H'</u>), 1.29 (d, *J* = 6.9 Hz, 3H, C**1**-<u>H$_3$</u>); ^{13}C NMR (125 MHz, $CDCl_3$) δ_C = 168.4 (C**4**), 157.6 (C**5**), 146.2 (Ph Ar<u>C</u>), 128.5 (Ph Ar<u>C</u>H), 127.0 (Ph Ar<u>C</u>H), 126.3 (Ph Ar<u>C</u>H), 117.8 (C**6**), 47.2 (C**3**), 39.5 (C**2**), 21.8 (C**1**). HRMS (ESI$^+$) calculated for $C_{13}H_{14}BrN_2$ $[M+H]^+$ = 277.0335, found 277.0331.

SFC conditions: CHIRALPACK SC (25 cm), 97:3 CO_2:*i*-PrOH, 2.0 mL/min, 254 nm; *Retention times*: t_{minor} = 5.6 min, t_{major} = 5.8 min.

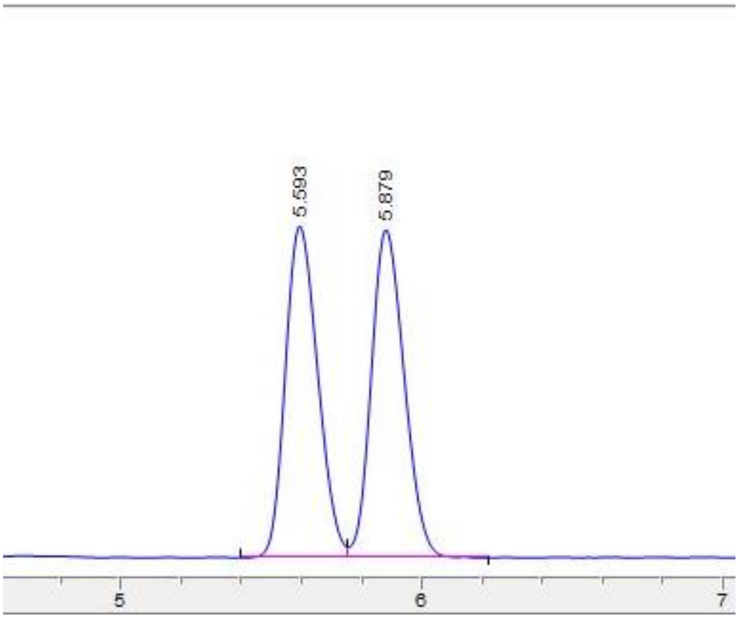

#	Time	Area	Height	Width	Area%	Symmetry
1	5.593	588.7	78.9	0.1171	49.859	0.764
2	5.879	592	78	0.1185	50.141	0.769

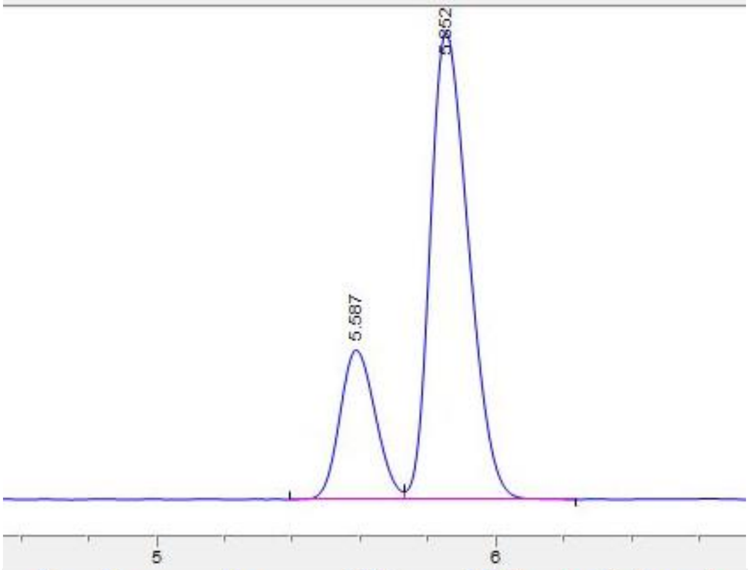

#	Time	Area	Height	Width	Area%	Symmetry
1	5.587	338.7	46.4	0.1151	22.965	0.82
2	5.852	1136.2	145.1	0.1234	77.035	0.664

***tert*-Butyl 2-(4,6-dimethoxy-1,3,5-triazin-2-yl)acetate (1m)**

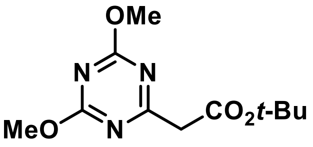

__General procedure C__: 2-Chloro-4,6-dimethoxy-1,3,5-triazine (702 mg, 4.00 mmol) was employed with *tert*-butyl acetate (1.39 g, 1.61 mL, 12.0 mmol) and NaHMDS (0.6 M in toluene, 20.0 mL, 12.0 mmol). Purification by flash column chromatography (Hexane/EtOAc = 8/1 to 4/1) afforded the title compound (459 mg, 45%) as a colorless oil. ^{1}H NMR (500 MHz, $CDCl_3$) δ_H = 4.03 (s, 6H), 3.69 (s, 2H), 1.46 (s, 9H); ^{13}C NMR (125 MHz, $CDCl_3$) δ_C = 176.8, 172.7, 167.7, 81.9, 55.4, 46.1, 28.2.

The spectroscopic properties were consistent with the data available in the literature.[7]

(*S*)-2,4-Dimethoxy-6-(2-phenylpropyl)-1,3,5-triazine (4ma)

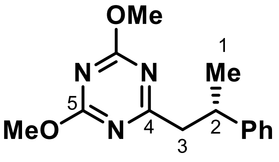

__General procedure E__: The preceding azaarylacetate (25.5 mg, 0.10 mmol) and styrene (52.1 mg, 57.5 μL, 0.50 mmol) were employed with [Ir(cod)$_2$]BARF (6.36 mg, 0.005 mmol) and **L6** (1.91 mg, 0.005 mmol) in toluene (0.20 mL). The reaction was stirred at 100 °C for 9 h. Then *p*-toluenesulfonic acid monohydrate (5.71 mg, 0.03 mmol) was added and the resulting reaction mixture was heated at 130 °C for 1 h. Purification by flash column chromatography (Hexane/EtOAc = 10/1 to 5/1) afforded the title

compound (8.20 mg, 32%, B:L > 25:1, 95:5 e.r.) as a colorless oil. $[\alpha]_D^{26}$ = +34.6 (c 0.2, CH_2Cl_2). IR (thin film) ν_{max}/cm^{-1}: 2915, 2851, 1553, 1464, 1054, 699. ^{1}H NMR (500 MHz, $CDCl_3$) δ_H = 7.31 – 7.23 (m, 4H, Ph ArCH), 7.22 – 7.12 (m, 1H, Ph ArCH), 4.00 (s, 6H, OCH_3), 3.51 (dqd, J = 8.3, 7.0, 6.9 Hz, 1H, C**2**-H), 3.07 (dd, J = 14.1, 6.9 Hz, 1H, C**3**-H), 2.96 (dd, J = 14.1, 8.3 Hz, 1H, C**3**-H'), 1.29 (d, J = 7.0 Hz, 3H, C**1**-H_3); ^{13}C NMR (125 MHz, $CDCl_3$) δ_C = 182.1 (C**4**), 172.5 (C**5**), 146.2 (Ph ArC), 128.5 (Ph ArCH), 127.1 (Ph ArCH), 126.3 (Ph ArCH), 55.2 (OCH_3), 46.8 (C**3**), 38.3 (C**2**), 22.0 (C**1**). HRMS (ESI^+) calculated for $C_{14}H_{18}N_3$ $[M+H]^+$ = 260.1394, found 260.1398.

SFC conditions: CHIRALPACK SB (25 cm), 98:2 CO_2:*i*-PrOH, 1.3 mL/min, 210 nm; *Retention times*: t_{minor} = 17.3 min, t_{major} = 17.9 min.

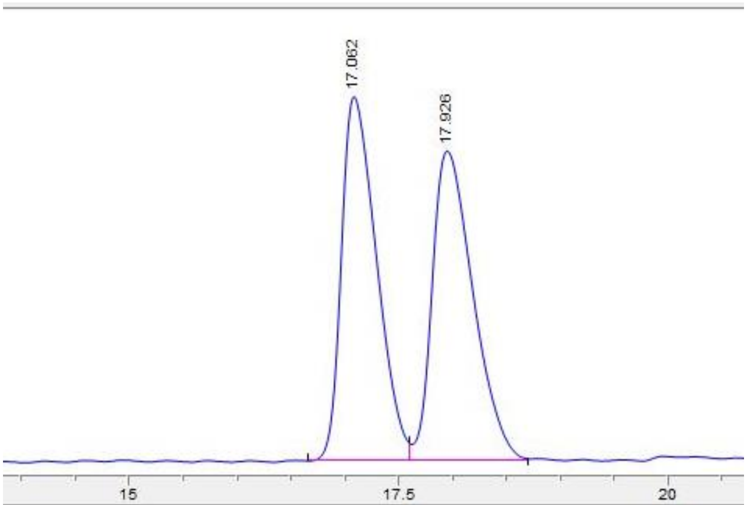

#	Time	Area	Height	Width	Area%	Symmetry
1	17.062	9123.4	402.9	0.3542	50.236	0.535
2	17.926	9037.8	342.9	0.3975	49.764	0.549

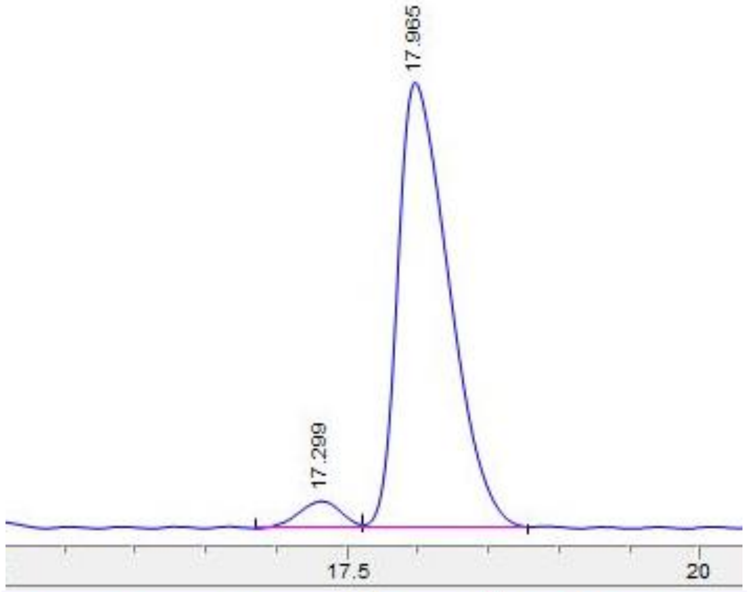

#	Time	Area	Height	Width	Area%	Symmetry
1	17.299	483.8	22.4	0.2696	4.949	1.084
2	17.965	9292.4	366.9	0.3755	95.051	0.493

***tert*-Butyl 2-(isoquinolin-1-yl)acetate (1n)**

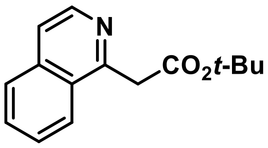

General procedure C: 1-Chloroisoquinoline (654 mg, 4.00 mmol) was employed with *tert*-butyl acetate (1.39 g, 1.61 mL, 12.0 mmol) and NaHMDS (0.6 M in toluene, 20.0 mL, 12.0 mmol). Purification by flash column chromatography (Hexane/EtOAc = 9/1 to 4/1) afforded the title compound (275 mg, 28%) as a yellow oil. ^{1}H NMR (500 MHz, $CDCl_3$) δ_H = 8.46 (d, J = 5.8 Hz, 1H), 8.12 – 8.03 (m, 1H), 7.83 (dd, J = 8.3, 1.1 Hz, 1H), 7.68 (ddd, J = 8.2, 6.8, 1.2 Hz, 1H), 7.64 – 7.57 (m, 2H), 4.28 (s, 2H), 1.41 (s, 9H); ^{13}C NMR (125 MHz, $CDCl_3$) δ_C = 169.8, 155.3, 141.9, 136.5, 130.2, 127.6, 127.6, 127.5, 125.5, 120.5, 81.6, 43.6, 28.1.

The spectroscopic properties were consistent with the data available in the literature.[8]

(*S*)-1-(2-Phenylpropyl)isoquinoline (4na)

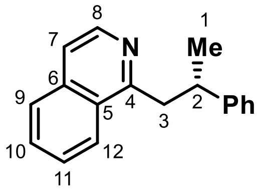

General procedure E: The preceding azaarylacetate (24.3 mg, 0.10 mmol) and styrene (52.1 mg, 57.5 µL, 0.50 mmol) were employed with [Ir(cod)$_2$]BARF (6.36 mg, 0.005 mmol) and **L6** (1.91 mg, 0.005 mmol) in toluene (0.20 mL). The reaction was stirred at 100 °C for 72 h. Then *p*-toluenesulfonic acid monohydrate (5.71 mg, 0.03 mmol) was added and the resulting reaction mixture was heated at 130 °C for 18 h. Purification by flash column chromatography (Hexane/EtOAc = 10/1 to 4/1) afforded the title compound (16.0 mg, 65%, B:L > 25:1, 92:8 e.r.) as a colorless oil. $[\alpha]_D^{25}$ = +25.1 (c 0.5, CH_2Cl_2). IR (thin film) ν_{max}/cm^{-1}: 2961, 2870, 1561, 1386, 1006, 699. ^{1}H NMR (500 MHz, $CDCl_3$) δ_H = 8.46 (d, J = 5.7 Hz, 1H, C**8**-H), 8.10 (d, J = 8.3 Hz, 1H, C**12**-H), 7.81 (d, J = 8.1 Hz, 1H, C**9**-H), 7.65 (ddd, J = 8.1, 6.8, 1.2 Hz, 1H, C**10**-H), 7.55 (ddd, J = 8.3, 6.8, 1.3 Hz, 1H, C**11**-H), 7.51 (d, J = 5.7 Hz, 1H, C**7**-H), 7.33 – 7.26 (m, 4H, Ph ArCH), 7.19 (tt, J = 5.6, 2.7 Hz, 1H, Ph ArCH), 3.68 – 3.60 (m, 1H, C**3**-H), 3.53 – 3.43 (m, 2H, C**2**-H + C**3**-H’), 1.30 (d, J = 6.5 Hz, 3H, C**1**-H_3); ^{13}C NMR (125 MHz, $CDCl_3$) δ_C = 160.7 (C**4**), 147.2 (Ph ArC), 141.9 (C**8**), 136.4 (C**6**), 129.9 (C**10**), 128.6 (Ph ArCH), 127.5 (C**5**), 127.5 (C**9**), 127.1 (C**11**), 127.0 (Ph ArCH), 126.3 (Ph ArCH), 125.5 (C**12**), 119.5 (C**7**), 43.8 (C**3**), 40.4 (C**2**), 21.4 (C**1**). HRMS (ESI$^+$) calculated for $C_{18}H_{18}N$ $[M+H]^+$ = 248.1436, found 248.1444.

SFC conditions: CHIRALPACK SB (25 cm), 96:4 CO_2:MeOH, 2.0 mL/min, 254 nm; *Retention times*: t_{minor} = 9.1 min, t_{major} = 9.8 min.

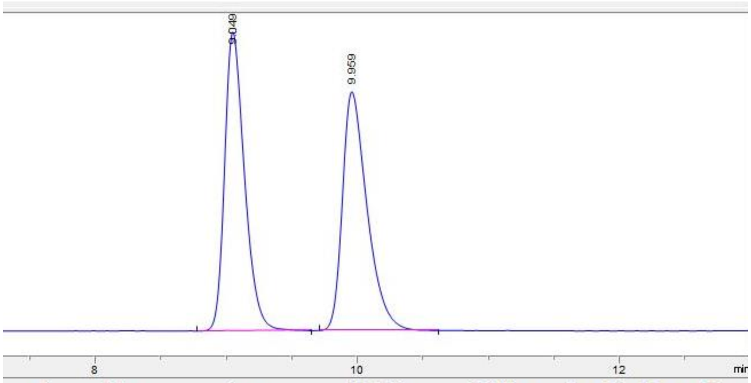

#	Time	Area	Height	Width	Area%	Symmetry
1	9.049	1877.7	177.3	0.1616	50.271	0.658
2	9.959	1857.5	141.6	0.197	49.729	0.58

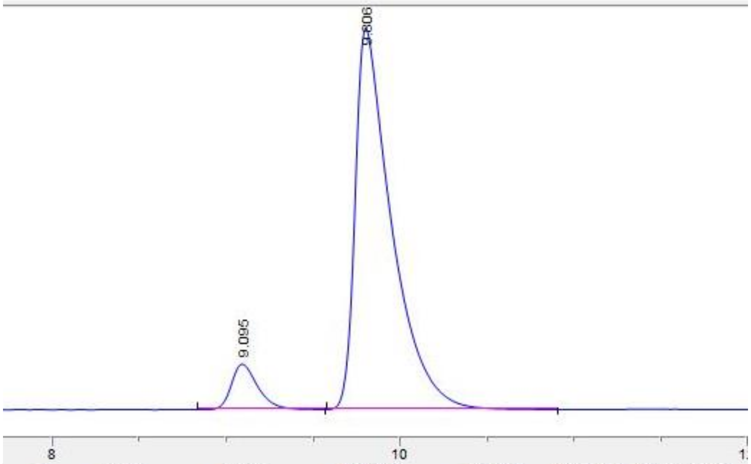

#	Time	Area	Height	Width	Area%	Symmetry
1	9.095	524.3	49.5	0.1617	7.986	0.651
2	9.806	6041.5	414.4	0.2101	92.014	0.376

***tert*-Butyl 2-(quinolin-2-yl)acetate (1o)**

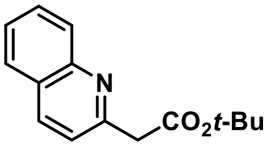

__**General procedure A**__: 2-Chloroquinoline (2.86 g, 20.0 mmol) was employed with diisopropylamine (3.36 mL, 24.0 mmol), *n*-BuLi (2.5 M in hexane, 9.60 mL, 24.0 mmol) and dibutyldicarbonate (5.67 g, 26.0 mmol). Purification by flash column chromatography (Hexane/EtOAc = 9/1 to 3/1) afforded the title compound (1.47 g, 30%) as a yellow solid. ^{1}H NMR (500 MHz, $CDCl_3$) δ_H = 8.13 (d, J = 8.5 Hz, 1H), 8.07 (d, J = 8.5 Hz, 1H), 7.80 (dd, J = 8.2, 1.3 Hz, 1H), 7.70 (ddd, J = 8.3, 6.8, 1.4 Hz, 1H), 7.56 – 7.49 (m, 1H), 7.43 (d, J = 8.4 Hz, 1H), 3.97 (s, 2H), 1.46 (s, 9H); ^{13}C NMR (125 MHz, $CDCl_3$) δ_C = 169.9, 155.5, 147.9, 136.6, 129.7, 129.1, 127.6, 127.1, 126.4, 121.9, 81.5, 46.1, 28.2.

The spectroscopic properties were consistent with the data available in the literature.[9]

(S)-2-(2-Phenylpropyl)quinoline (4oa)

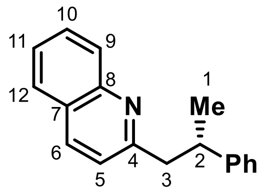

General procedure E: The preceding azaarylacetate (24.3 mg, 0.10 mmol) and styrene (52.1 mg, 57.5 μL, 0.50 mmol) were employed with $[Ir(cod)_2]BARF$ (6.36 mg, 0.005 mmol) and **L6** (1.91 mg, 0.005 mmol) in toluene (0.20 mL). The reaction was stirred at 100 °C for 120 h. Then *p*-toluenesulfonic acid monohydrate (5.71 mg, 0.03 mmol) was added and the resulting reaction mixture was heated at 130 °C for 18 h. Purification by flash column chromatography (Hexane/EtOAc = 10/1 to 4/1) afforded the title compound (15.0 mg, 61%, B:L > 25:1, 95.5:4.5 e.r.) as a colorless oil. $[\alpha]_D^{25}$ = +123.1 (*c* 0.5, CH_2Cl_2). IR (thin film) ν_{max}/cm^{-1}: 2960, 2925, 1504, 1426, 1015, 699. ^1H NMR (500 MHz, $CDCl_3$) δ_H = 8.07 (d, *J* = 8.4 Hz, 1H, C**9**-H), 7.96 (d, *J* = 8.4 Hz, 1H, C**6**-H), 7.75 (d, *J* = 8.1 Hz, 1H, C**12**-H), 7.69 (ddd, *J* = 8.4, 6.8, 1.4 Hz, 1H, C**10**-H), 7.48 (ddd, *J* = 8.1, 6.8, 1.1 Hz, 1H, C**11**-H), 7.30 – 7.22 (m, 4H, Ph ArCH), 7.21 – 7.14 (m, 1H, Ph ArCH), 7.06 (d, *J* = 8.4 Hz, 1H, C**5**-H), 3.40 (ddq, *J* = 8.1, 7.1, 6.9 Hz, 1H, C**2**-H), 3.28 (dd, *J* = 13.3, 7.1 Hz, 1H, C**3**-H), 3.21 (dd, *J* = 13.3, 8.1 Hz, 1H, C**3**-H'), 1.33 (d, *J* = 6.9 Hz, 3H, C**1**-H_3); ^{13}C NMR (125 MHz, $CDCl_3$) δ_C = 161.4 (C**4**), 148.0 (C**8**), 146.7 (Ph ArC), 136.0 (C**6**), 129.5 (C**10**), 129.0 (C**9**), 128.5 (Ph ArCH), 127.6 (C**12**), 127.2 (Ph ArCH), 126.9 (C**7**), 126.3 (Ph ArCH), 125.9 (C**11**), 122.2 (C**5**), 47.9 (C**3**), 40.7 (C**2**), 21.8 (C**1**). HRMS (ESI^+) calculated for $C_{18}H_{18}N$ $[M+H]^+$ = 248.1436, found 248.1434.

SFC conditions: CHIRALPACK SB (25 cm), 95:5 CO_2:*i*-PrOH, 2.0 mL/min, 254 nm; *Retention times*: t_{major} = 12.0 min, t_{minor} = 13.2 min.

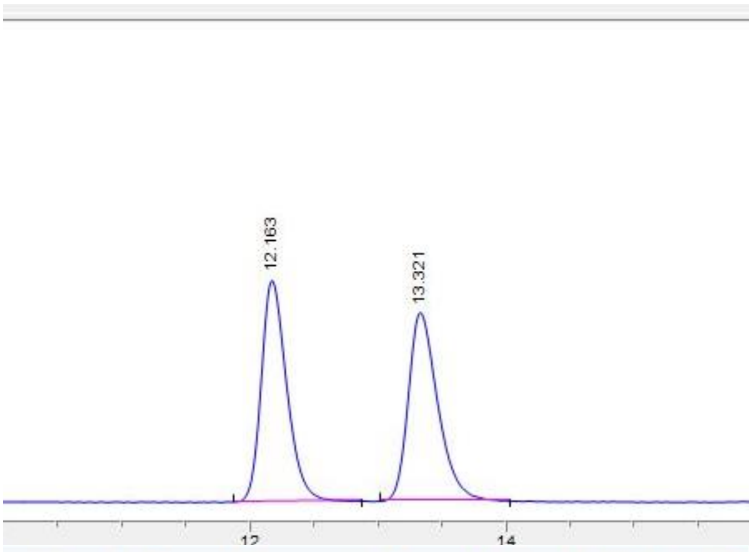

#	Time	Area	Height	Width	Area%	Symmetry
1	12.163	769.6	55.5	0.2118	50.238	0.693
2	13.321	762.4	47.3	0.244	49.762	0.642

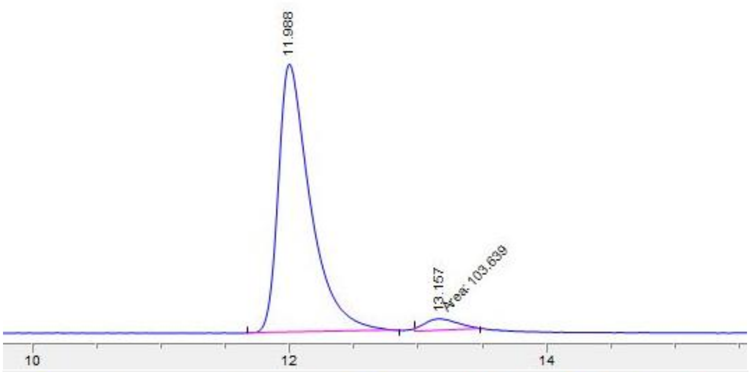

#	Time	Area	Height	Width	Area%	Symmetry
1	11.988	2288.9	131.9	0.2564	95.668	0.488
2	13.157	103.6	5.7	0.301	4.332	0.791

***tert*-Butyl 2-(5-chlorobenzo[*d*]thiazol-2-yl)acetate (1p)**

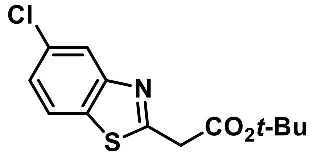

__**General procedure A**__: 5-Chloro-2-methylbenzo[*d*]thiazole (1.84 g, 10.0 mmol) was employed with diisopropylamine (1.68 mL, 12.0 mmol), *n*-BuLi (2.5 M in hexane, 4.80 mL, 12.0 mmol) and dibutyldicarbonate (2.62 g, 12.0 mmol). Purification by flash column chromatography (Hexane/EtOAc = 15/1 to 5/1) afforded the title compound (714 mg, 25%) as a colorless solid. m.p. 84 - 86 °C (EtOAc/hexane). IR (thin film) ν_{max}/cm^{-1}: 2980, 2936, 1500, 1389, 1163, 802. ^{1}H NMR (500 MHz, $CDCl_3$) δ_H = 7.99 (d, *J* = 2.0 Hz, 1H), 7.78 (d, *J* = 8.5 Hz, 1H), 7.36 (dd, *J* = 8.5, 2.0 Hz, 1H), 4.09 (s, 2H), 1.50 (s, 9H); ^{13}C NMR (125 MHz, $CDCl_3$) δ_C = 167.5, 165.6, 153.6, 134.2, 132.2, 125.8, 122.9, 122.4, 82.9, 41.3, 28.1. HRMS (ESI$^+$) calculated for $C_{13}H_{14}ClNNaO_2S$ $[M+Na]^+$ = 306.0326, found 306.0322.

(*S*)-5-Chloro-2-(2-phenylpropyl)benzo[*d*]thiazole (4pa)

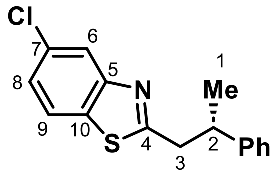

__**General procedure E**__: The preceding azaarylacetate (28.4 mg, 0.10 mmol) and styrene (15.6 mg, 17.2 μL, 0.15 mmol) were employed with [Ir(cod)$_2$]BARF (6.36 mg, 0.005 mmol) and **L6** (1.91 mg, 0.005 mmol) in toluene (0.20 mL). The reaction was stirred at 100 °C for 12 h. Then *p*-toluenesulfonic acid monohydrate (5.71 mg, 0.03 mmol) was added and the resulting reaction mixture was heated at 130 °C for 3 h. Purification by flash column chromatography (Hexane/EtOAc = 12/1 to 6/1) afforded the title compound (27.9 mg, 97%, B:L > 25:1, 98:2 e.r.) as a colorless oil. $[\alpha]_D^{23}$ = +113.7 (*c* 0.5, CH_2Cl_2). IR

(thin film) ν_{max}/cm^{-1}: 2978, 2948, 1725, 1060, 1007, 607. ^{1}H NMR (500 MHz, CDCl$_3$) δ_H = 7.91 (d, J = 2.0 Hz, 1H, C**6**-H), 7.64 (d, J = 8.5 Hz, 1H, C**9**-H), 7.28 – 7.23 (m, 3H, C**8**-H + 2 × Ph ArCH), 7.23 – 7.20 (m, 2H, Ph ArCH), 7.19 – 7.15 (m, 1H, Ph ArCH), 3.40 – 3.28 (m, 3H, C**2**-H + C**3**-H + C**3**-H'), 1.34 (d, J = 6.4 Hz, 3H, C**1**-H$_3$); ^{13}C NMR (125 MHz, CDCl$_3$) δ_C = 172.5 (C**4**), 154.1 (C**5**), 145.3 (Ph ArC), 133.6 (C**7**), 132.0 (C**10**), 128.8 (Ph ArCH), 127.1 (Ph ArCH), 126.8 (Ph ArCH), 125.3 (C**8**), 122.6 (C**6**), 122.3 (C**9**), 42.9 (C**3**), 40.7 (C**2**), 22.0 (C**1**). HRMS (ESI$^+$) calculated for $C_{16}H_{15}ClNS$ [M+H]$^+$ = 288.0608, found 288.0604.

SFC conditions: CHIRALPACK SB (25 cm), 94:6 CO_2:*i*-PrOH, 2.0 mL/min, 254 nm; *Retention times*: t_{major} = 9.3 min, t_{minor} = 9.7 min.

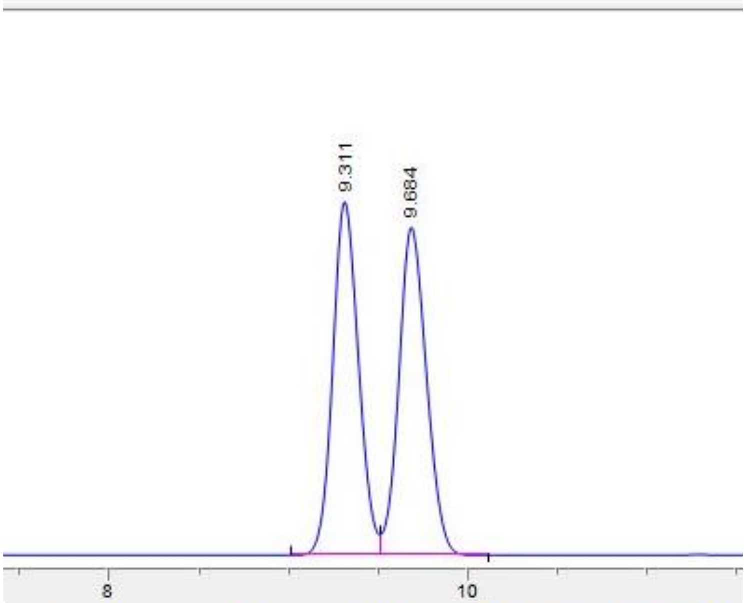

#	Time	Area	Height	Width	Area%	Symmetry
1	9.311	7820.2	783.1	0.1545	50.009	0.839
2	9.684	7817.3	726.6	0.1655	49.991	0.811

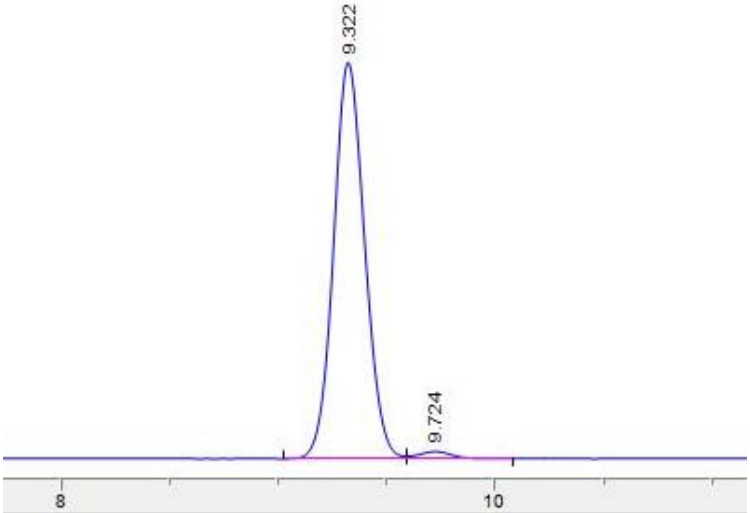

#	Time	Area	Height	Width	Area%	Symmetry
1	9.322	5860.6	589.2	0.1541	98.134	0.859
2	9.724	111.4	10.5	0.1598	1.866	0.981

tert-Butyl 2-(5-methoxybenzo[_d_]thiazol-2-yl)acetate (1q)

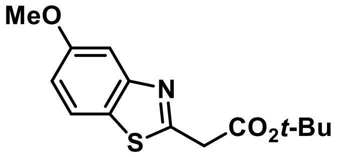

General procedure A: 5-Methoxy-2-methylbenzo[*d*]thiazole (1.79 g, 10.0 mmol) was employed with diisopropylamine (1.68 mL, 12.0 mmol), *n*-BuLi (2.5 M in hexane, 4.80 mL, 12.0 mmol) and dibutyldicarbonate (2.62 g, 12.0 mmol). Purification by flash column chromatography (Hexane/EtOAc = 15/1 to 4/1) afforded the title compound (800 mg, 29%) as a yellow solid. m.p. 38 - 41 °C (EtOAc/hexane). IR (thin film) ν_{max}/cm^{-1}: 2978, 2937, 1729, 1467, 1142, 841. ^{1}H NMR (500 MHz, $CDCl_3$) δ_H = 7.71 (d, *J* = 8.8 Hz, 1H), 7.49 (d, *J* = 2.5 Hz, 1H), 7.03 (dd, *J* = 8.8, 2.5 Hz, 1H), 4.07 (s, 2H), 3.88 (s, 3H), 1.49 (s, 9H); ^{13}C NMR (125 MHz, $CDCl_3$) δ_C = 167.7, 164.7, 159.0, 154.0, 127.7, 121.8, 115.5, 105.5, 82.6, 55.7, 41.3, 28.1. HRMS (ESI$^+$) calculated for $C_{14}H_{17}NNaO_3S$ $[M+Na]^+$ = 302.0821, found 302.0815.

(_S_)-5-Methoxy-2-(2-phenylpropyl)benzo[_d_]thiazole (4qa)

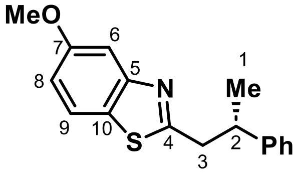

General procedure E: The preceding azaarylacetate (27.9 mg, 0.10 mmol) and styrene (15.6 mg, 17.2 μL, 0.15 mmol) were employed with $[Ir(cod)_2]BARF$ (6.36 mg, 0.005 mmol) and **L6** (1.91 mg, 0.005 mmol) in toluene (0.20 mL). The reaction was stirred at 100 °C for 12 h. Then *p*-toluenesulfonic acid monohydrate (5.71 mg, 0.03 mmol) was added and the resulting reaction mixture was heated at 130 °C for 3 h. Purification by flash column chromatography (Hexane/EtOAc = 10/1 to 4/1) afforded the title compound (27.0 mg, 95%, B:L > 25:1, 98:2 e.r.) as a colorless oil. $[\alpha]_D^{25}$ = +108.0 (*c* 0.5, CH_2Cl_2). IR (thin film) ν_{max}/cm^{-1}: 2921, 2861, 1406, 1157, 1033, 619. ^{1}H NMR (500 MHz, $CDCl_3$) δ_H = 7.61 (d, *J* = 8.8 Hz, 1H, C**9**-H), 7.46 (d, *J* = 2.5 Hz, 1H, C**6**-H), 7.32 – 7.22 (m, 4H, Ph ArCH), 7.22 – 7.14 (m, 1H, Ph ArCH), 6.96 (dd, *J* = 8.8, 2.5 Hz, 1H, C**8**-H), 3.85 (s, 3H, OCH_3), 3.43 – 3.33 (m, 2H, C**2**-H + C**3**-H), 3.33 – 3.27 (m, 1H, C**3**-H'), 1.36 (d, *J* = 6.4 Hz, 3H, C**1**-H_3); ^{13}C NMR (125 MHz, $CDCl_3$) δ_C = 171.8 (C**4**), 158.9 (C**7**), 154.4 (C**5**), 145.6 (Ph ArC), 128.7 (Ph ArCH), 127.1 (Ph ArCH), 127.1 (C**10**), 126.7 (Ph ArCH), 121.8 (C**9**), 114.9 (C**8**), 105.3 (C**6**), 55.7 (OCH_3), 42.9 (C**3**), 40.7 (C**2**), 21.9 (C**1**). HRMS (ESI$^+$) calculated for $C_{17}H_{17}NaNOS$ $[M+Na]^+$ = 306.0923, found 306.0922.

SFC conditions: CHIRALPACK SB (25 cm), 94:6 CO_2:*i*-PrOH, 2.0 mL/min, 254 nm; *Retention times*: t_{major} = 11.8 min, t_{minor} = 12.5 min.

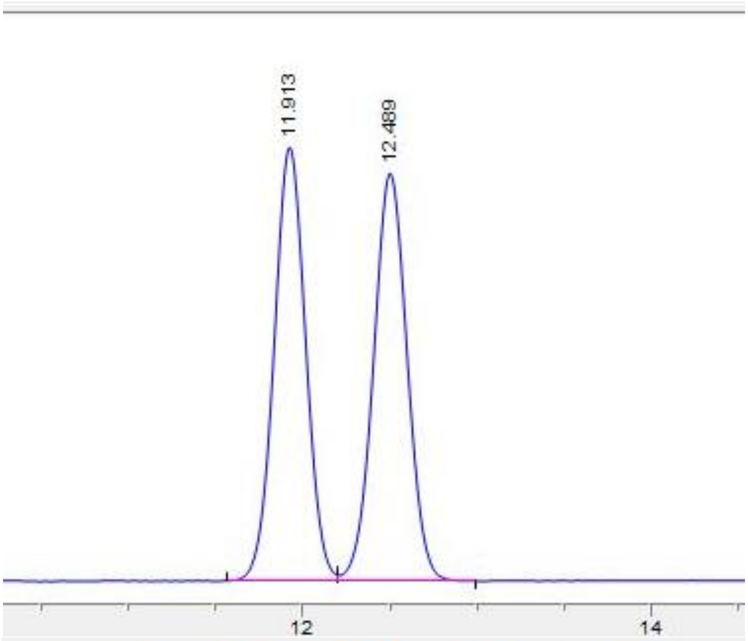

#	Time	Area	Height	Width	Area%	Symmetry
1	11.913	1156.2	92.1	0.1944	49.905	0.949
2	12.489	1160.6	86.7	0.2062	50.095	0.952

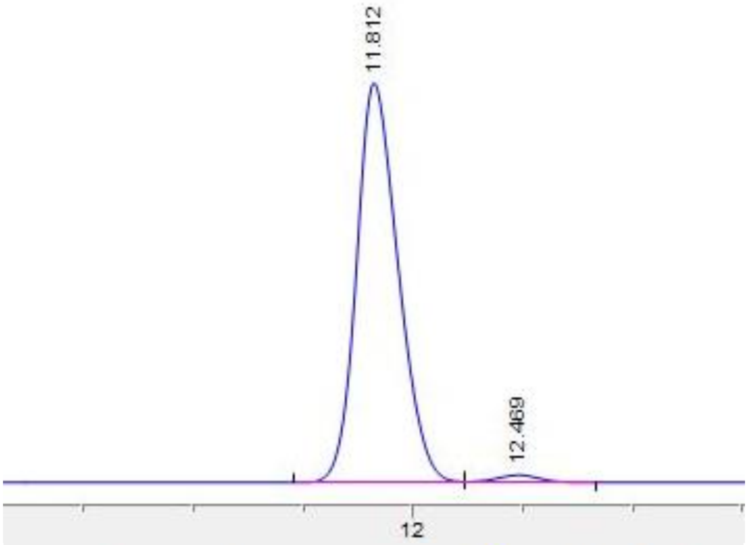

#	Time	Area	Height	Width	Area%	Symmetry
1	11.812	7013.2	531.9	0.1998	98.097	0.711
2	12.469	136	9.8	0.2051	1.903	1.035

***tert*-Butyl 2-(6-bromobenzo[*d*]thiazol-2-yl)acetate (1r)**

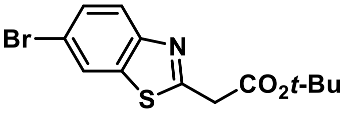

__**General procedure C**__: 6-Bromo-2-chlorobenzo[*d*]thiazole (994 mg, 4.00 mmol) was employed with *tert*-butyl acetate (1.39 g, 1.62 mL, 12.0 mmol) and NaHMDS (0.6 M in toluene, 20.0 mL, 12.0 mmol). Purification by flash column chromatography (Hexane/EtOAc = 10/1 to 4/1) afforded the title

compound (1.11 g, 85%) as a yellow solid. m.p. 57 - 59 °C (EtOAc/hexane). IR (thin film) ν_{max}/cm^{-1}: 2978, 2931, 1722, 1366, 1148, 1082, 822. ^{1}H NMR (500 MHz, $CDCl_3$) δ_H = 8.01 (d, J = 2.0 Hz, 1H), 7.85 (d, J = 8.7 Hz, 1H), 7.57 (dd, J = 8.7, 2.0 Hz, 1H), 4.08 (s, 2H), 1.50 (s, 9H); ^{13}C NMR (125 MHz, $CDCl_3$) δ_C = 167.5, 164.2, 151.6, 137.6, 129.7, 124.2, 124.2, 118.9, 82.9, 41.1, 28.1. HRMS (ESI$^+$) calculated for $C_{13}H_{14}BrNNaO_2S$ [M+Na]$^+$ = 349.9820, found 349.9812.

(*S*)-6-Bromo-2-(2-phenylpropyl)benzo[*d*]thiazole (4ra)

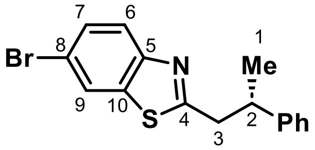

General procedure E: The preceding azaarylacetate (32.8 mg, 0.10 mmol) and styrene (15.6 mg, 17.2 μL, 0.15 mmol) were employed with [Ir(cod)$_2$]BARF (6.36 mg, 0.005 mmol) and **L6** (1.91 mg, 0.005 mmol) in toluene (0.20 mL). The reaction was stirred at 100 °C for 12 h. Then *p*-toluenesulfonic acid monohydrate (5.71 mg, 0.03 mmol) was added and the resulting reaction mixture was heated at 130 °C for 3 h. Purification by flash column chromatography (Hexane/EtOAc = 12/1 to 6/1) afforded the title compound (31.0 mg, 93%, B:L > 25:1, 98:2 e.r.) as a colorless oil. $[\alpha]_D^{24}$ = +102.7 (c 0.5, CH_2Cl_2). IR (thin film) ν_{max}/cm^{-1}: 2919, 2851, 1435, 1267, 1118, 699. ^{1}H NMR (500 MHz, $CDCl_3$) δ_H = 7.88 (d, J = 2.0 Hz, 1H, C**9**-H), 7.78 (d, J = 8.7 Hz, 1H, C**6**-H), 7.50 (dd, J = 8.7, 2.0 Hz, 1H, C**7**-H), 7.29 – 7.24 (m, 2H, Ph ArCH), 7.23 – 7.21 (m, 2H, Ph ArCH), 7.20 – 7.16 (m, 1H, Ph ArCH), 3.39 – 3.28 (m, 3H, C**2**-H + C**3**-H + C**3**-H’), 1.35 (d, J = 6.7 Hz, 3H, C**1**-H_3); ^{13}C NMR (125 MHz, $CDCl_3$) δ_C = 171.1 (C**4**), 152.1 (C**5**), 145.3 (Ph ArC), 137.0 (C**10**), 129.5 (C**7**), 128.8 (Ph ArCH), 127.1 (Ph ArCH), 126.8 (Ph ArCH), 124.1 (C**9**), 123.8 (C**6**), 118.4 (C**8**), 42.8 (C**3**), 40.6 (C**2**), 22.0 (C**1**). HRMS (ESI$^+$) calculated for $C_{16}H_{15}BrNS$ [M+H]$^+$ = 332.0103, found 332.0097.

SFC conditions: CHIRALPACK SB (25 cm), 92:8 CO_2:*i*-PrOH, 2.0 mL/min, 254 nm; *Retention times*: t_{major} = 14.3 min, t_{minor} = 19.1 min.

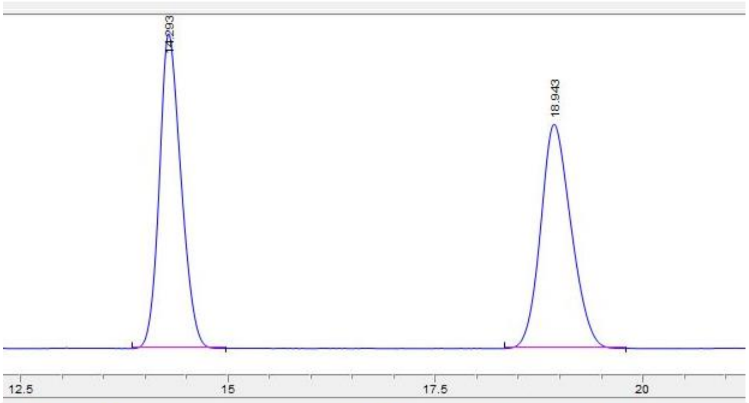

#	Time	Area	Height	Width	Area%	Symmetry
1	14.293	4225.2	232.1	0.2764	49.929	0.729
2	18.943	4237.2	165.3	0.391	50.071	0.768

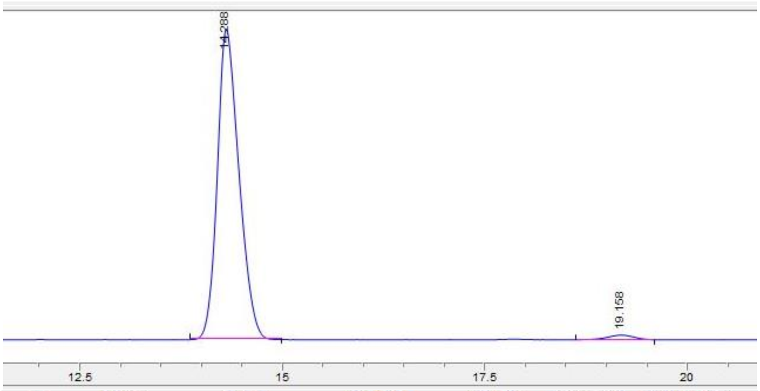

#	Time	Area	Height	Width	Area%	Symmetry
1	14.288	5197.3	279.9	0.2786	98.200	0.681
2	19.158	95.3	4.1	0.2772	1.800	1.063

***tert*-Butyl 2-(6-fluorobenzo[*d*]thiazol-2-yl)acetate (1s)**

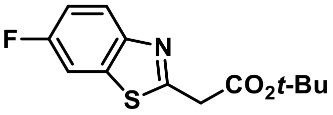

__General procedure C__: 2-Chloro-6-fluorobenzo[*d*]thiazole (563 mg, 3.00 mmol) was employed with *tert*-butyl acetate (1.05 g, 1.22 mL, 9.00 mmol) and NaHMDS (0.6 M in toluene, 15.0 mL, 9.00 mmol). Purification by flash column chromatography (Hexane/EtOAc = 10/1 to 4/1) afforded the title compound (358 mg, 45%) as a yellow solid. m.p. 70 - 73 °C (EtOAc/hexane). IR (thin film) ν_{max}/cm^{-1}: 2980, 2931, 1731, 1456, 1151, 849. ^{1}H NMR (500 MHz, $CDCl_3$) δ_H = 7.94 (dd, *J* = 8.9, 4.8 Hz, 1H), 7.55 (dd, *J* = 8.1, 2.6 Hz, 1H), 7.20 (ddd, *J* = 8.9, 8.9, 2.6 Hz, 1H), 4.07 (s, 2H), 1.50 (s, 9H); ^{13}C NMR (125 MHz, $CDCl_3$) δ_C = 167.5, 163.3 (d, $^4J_{CF}$ = 3.5 Hz), 160.6 (d, $^1J_{CF}$ = 245.5 Hz), 149.3, 136.9 (d, $^3J_{CF}$ = 11.0 Hz), 124.0 (d, $^3J_{CF}$ = 9.5 Hz), 114.8 (d, $^2J_{CF}$ = 24.8 Hz), 107.8 (d, $^2J_{CF}$ = 26.8 Hz), 82.8, 41.1, 28.1; ^{19}F NMR (471 MHz, $CDCl_3$) δ_F = -115.9 – -116.3 (m, 1F). HRMS (ESI$^+$) calculated for $C_{13}H_{14}FNNaO_2S$ [M+Na]$^+$ = 290.0621, found 290.0615.

(*S*)-6-Fluoro-2-(2-phenylpropyl)benzo[*d*]thiazole (4sa)

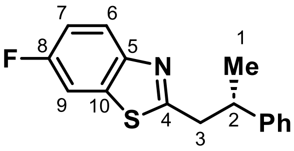

__General procedure E__: The preceding azaarylacetate (26.7 mg, 0.10 mmol) and styrene (15.6 mg, 17.2 µL, 0.15 mmol) were employed with [Ir(cod)$_2$]BARF (6.36 mg, 0.005 mmol) and **L6** (1.91 mg, 0.005 mmol) in toluene (0.20 mL). The reaction was stirred at 100 °C for 12 h. Then *p*-toluenesulfonic acid monohydrate (5.71 mg, 0.03 mmol) was added and the resulting reaction mixture was heated at 130 °C for 3 h. Purification by flash column chromatography (Hexane/EtOAc = 15/1 to 9/1) afforded the title compound (25.0 mg, 92%, B:L > 25:1, 98:2 e.r.) as a colorless oil. $[\alpha]_D^{24}$ = +103.5 (*c* 0.5, CH_2Cl_2). IR (thin film) ν_{max}/cm^{-1}: 2923, 2843, 1456, 1054, 1033, 610. ^{1}H NMR (500 MHz, $CDCl_3$) δ_H = 7.89 (dd, *J*

= 8.9, 4.8 Hz, 1H, C**6**-H), 7.45 (dd, J = 8.2, 2.6 Hz, 1H, C**9**-H), 7.33 – 7.27 (m, 2H, Ph ArCH), 7.27 – 7.24 (m, 2H, Ph ArCH), 7.23 – 7.19 (m, 1H, Ph ArCH), 7.16 (ddd, J = 8.9, 8.9, 2.6 Hz, 1H, C**7**-H), 3.41 – 3.31 (m, 3H, C**2**-H + C**3**-H + C**3**-H'), 1.37 (d, J = 6.2 Hz, 3H, C**1**-H_3); ^{13}C NMR (125 MHz, $CDCl_3$) δ_C = 170.2 (d, $^5J_{CF}$ = 3.3 Hz, C**4**), 160.3 (d, $^1J_{CF}$ = 244.7 Hz, C**8**), 149.8 (d, $^4J_{CF}$ = 1.8 Hz, C**5**), 145.4 (Ph ArC), 136.3 (d, $^3J_{CF}$ = 11.3 Hz, C**10**), 128.8 (Ph ArCH), 127.1 (Ph ArCH), 126.8 (Ph ArCH), 123.5 (d, $^3J_{CF}$ = 9.4 Hz, C**6**), 114.6 (d, $^2J_{CF}$ = 24.6 Hz, C**7**), 107.8 (d, $^2J_{CF}$ = 26.5 Hz, C**9**), 42.8 (C**3**), 40.6 (C**2**), 21.9 (C**1**); ^{19}F NMR (471 MHz, $CDCl_3$) δ_F = -116.6 – -117.0 (m, 1F). HRMS (ESI$^+$) calculated for $C_{16}H_{15}FNS$ $[M+H]^+$ = 272.0904, found 272.0904.

SFC conditions: CHIRALPACK SB (25 cm), 96:4 CO_2:*i*-PrOH, 1.5 mL/min, 254 nm; *Retention times*: t_{major} = 10.7 min, t_{minor} = 11.2 min.

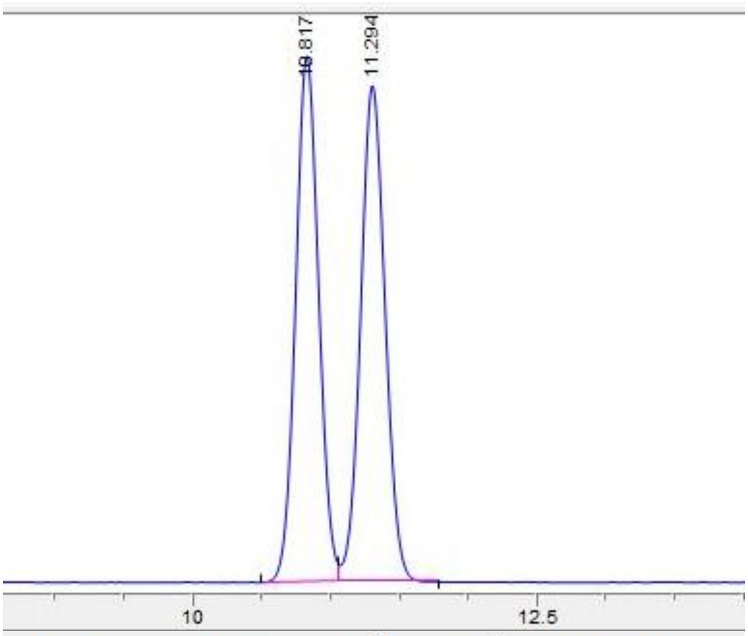

#	Time	Area	Height	Width	Area%	Symmetry
1	10.817	2712.5	232.9	0.1799	49.977	0.908
2	11.294	2715	219.6	0.1902	50.023	0.906

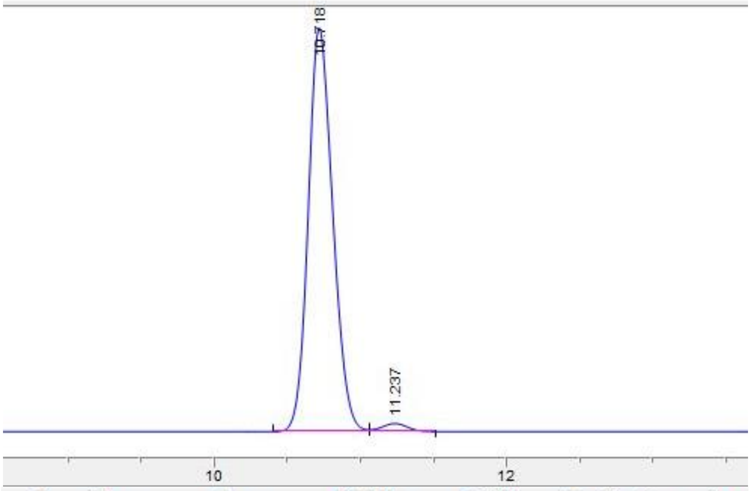

#	Time	Area	Height	Width	Area%	Symmetry
1	10.718	7521.9	644	0.1803	97.904	0.784
2	11.237	161	12.8	0.1863	2.096	1.038

(*S*)-2-(2-(*p*-Tolyl)propyl)benzo[*d*]thiazole (4gb)

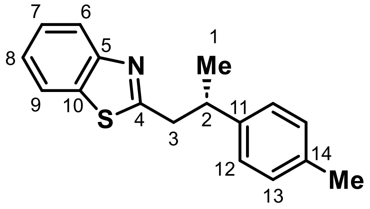

<u>**General procedure E**</u>: The preceding *tert*-butyl 2-(benzo[*d*]thiazol-2-yl)acetate (24.9 mg, 0.10 mmol) and 4-methylstyrene (23.6 mg, 19.7 μL, 0.20 mmol) were employed with $[Ir(cod)_2]$BARF (6.36 mg, 0.005 mmol) and **L6** (1.91 mg, 0.005 mmol) in toluene (0.20 mL). The reaction was stirred at 100 °C for 12 h. Then *p*-toluenesulfonic acid monohydrate (5.71 mg, 0.03 mmol) was added and the resulting reaction mixture was heated at 130 °C for 3 h. Purification by flash column chromatography (Hexane/EtOAc = 100/1 to 20/1) afforded the title compound (23.3 mg, 87%, B:L > 25:1, 98:2 e.r.) as a colorless oil. $[\alpha]_D^{25}$ = +84.2 (*c* 0.5, CH_2Cl_2). IR (thin film) ν_{max}/cm^{-1}: 2962, 2921, 1516, 1456, 1436, 1126, 1013, 759. ^{1}H NMR (500 MHz, $CDCl_3$) δ_H = 7.98 (d, *J* = 8.6 Hz, 1H, C**6**-<u>H</u>), 7.80 (d, *J* = 7.8 Hz, 1H, C**9**-<u>H</u>), 7.47 – 7.42 (m, 1H, C**7**-<u>H</u>), 7.36 – 7.31 (m, 1H, C**8**-<u>H</u>), 7.19 – 7.16 (m, 2H, C**12**-<u>H</u>), 7.14 – 7.10 (m, 2H, C**13**-<u>H</u>), 3.44 – 3.31 (m, 3H, C**2**-<u>H</u> + C**3**-<u>H</u> + C**3**-<u>H’</u>), 2.32 (s, 3H, ArC<u>H</u>$_3$), 1.36 (d, *J* = 6.2 Hz, 3H, C**1**-<u>H</u>$_3$); ^{13}C NMR (125 MHz, $CDCl_3$) δ_C = 170.7 (C**4**), 153.2 (C**5**), 142.6 (C**11**), 136.2 (C**14**), 135.4 (C**10**), 129.4 (C**13**), 127.0 (C**12**), 126.0 (C**7**), 124.8 (C**8**), 122.7 (C**6**), 121.6 (C**9**), 42.9 (C**3**), 40.3 (C**2**), 22.1 (C**1**), 21.2 (Ar<u>C</u>H$_3$). HRMS (ESI$^+$) calculated for $C_{17}H_{18}NS$ $[M+H]^+$ = 268.1154, found 268.1153.

SFC conditions: CHIRALPACK SB (25 cm), 98:2 CO_2:*i*-PrOH, 2.0 mL/min, 254 nm; *Retention times*: t_{major} = 20.6 min, t_{minor} = 23.7 min.

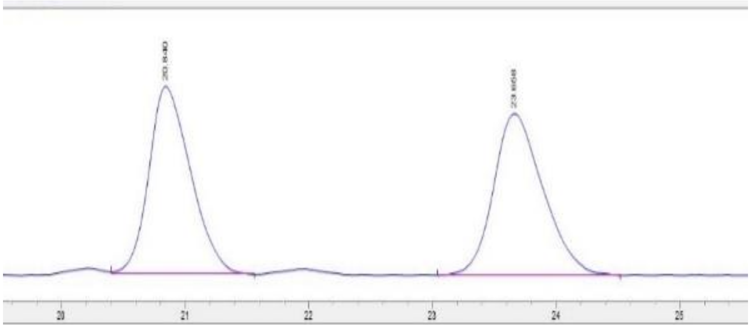

#	Time	Area	Height	Width	Area%	Symmetry
1	20.84	1687.6	71.3	0.3542	49.789	0.669
2	23.658	1701.8	61.2	0.3986	50.211	0.712

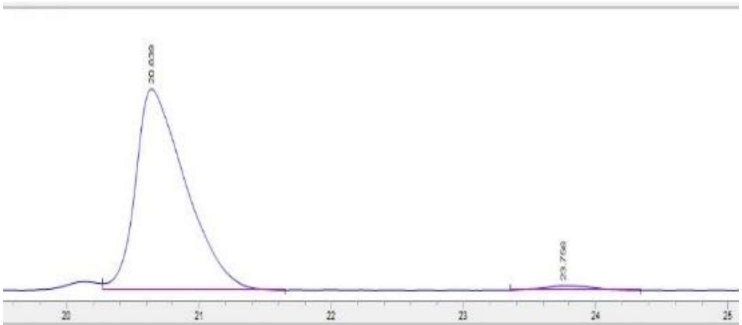

#	Time	Area	Height	Width	Area%	Symmetry
1	20.639	4741.4	179.3	0.3945	97.866	0.461
2	23.756	103.4	3.9	0.3158	2.134	0.679

(*S*)-2-(2-([1,1'-Biphenyl]-4-yl)propyl)benzo[*d*]thiazole (4gc)

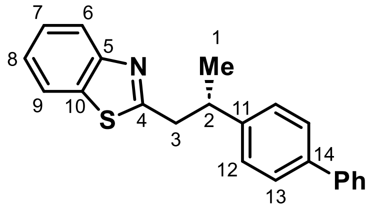

General procedure E: The preceding *tert*-butyl 2-(benzo[*d*]thiazol-2-yl)acetate (24.9 mg, 0.10 mmol) and 4-vinyl-1,1'-biphenyl (36.0 mg, 0.20 mmol) were employed with $[Ir(cod)_2]BARF$ (6.36 mg, 0.005 mmol) and **L6** (1.91 mg, 0.005 mmol) in toluene (0.20 mL). The reaction was stirred at 100 °C for 12 h. Then *p*-toluenesulfonic acid monohydrate (5.71 mg, 0.03 mmol) was added and the resulting reaction mixture was heated at 130 °C for 3 h. Purification by flash column chromatography (Hexane/EtOAc = 100/1 to 20/1) afforded the title compound (30.6 mg, 93%, B:L > 25:1, 98:2 e.r.) as a colorless oil. $[\alpha]_D^{19}$ = +145.0 (*c* 0.5, CH_2Cl_2). IR (thin film) ν_{max}/cm^{-1}: 2962, 2929, 1512, 1486, 1435, 1114, 1007, 836. ^1H NMR (500 MHz, $CDCl_3$) δ_H = 8.00 (d, *J* = 8.1 Hz, 1H, C**6**-H), 7.81 (d, *J* = 8.1 Hz, 1H, C**9**-H), 7.61 – 7.58 (m, 2H, Ph ArCH), 7.57 – 7.54 (m, 2H, C**13**-H), 7.48 – 7.41 (m, 3H, C**7**-H + 2 × Ph ArCH), 7.38 – 7.31 (m, 4H, C**8**-H + 2 × C**12**-H + Ph ArCH), 3.51 – 3.44 (m, 2H, C**2**-H + C**3**-H), 3.44 – 3.35 (m, 1H, C**3**-H’), 1.43 (d, *J* = 6.4 Hz, 3H, C**1**-H_3); ^{13}C NMR (125 MHz, $CDCl_3$) δ_C = 170.4 (C**4**), 153.2 (C**5**), 144.7 (C**11**), 141.0 (Ph ArC), 139.6 (C**14**), 135.4 (C**10**), 128.8 (Ph ArCH), 127.5 (C**12**), 127.4 (C**13**), 127.3 (Ph ArCH), 127.1 (Ph ArCH), 126.0 (C**7**), 124.9 (C**8**), 122.7 (**C6**), 121.6 (C**9**), 42.8 (C**3**), 40.3 (C**2**), 21.9 (C**1**). HRMS (ESI$^+$) calculated for $C_{22}H_{20}NS$ $[M+H]^+$ = 330.1311, found 330.1312.

SFC conditions: CHIRALPACK SB (25 cm), 90:10 CO_2:*i*-PrOH, 2.0 mL/min, 254 nm; *Retention times*: t_{major} = 10.8 min, t_{minor} = 11.5 min.

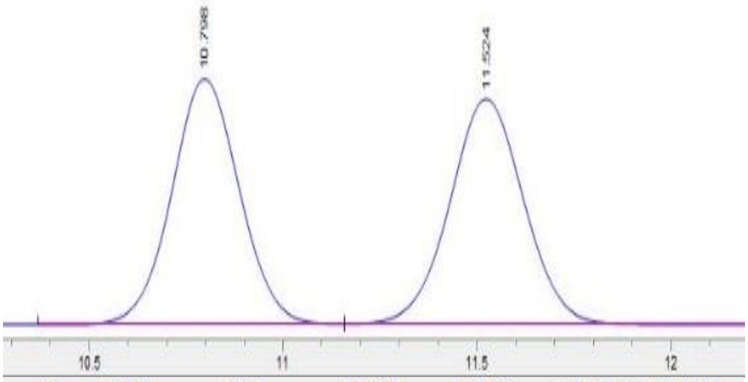

#	Time	Area	Height	Width	Area%	Symmetry
1	10.798	10385.3	854.1	0.1879	49.876	0.922
2	11.524	10437	784	0.2053	50.124	0.948

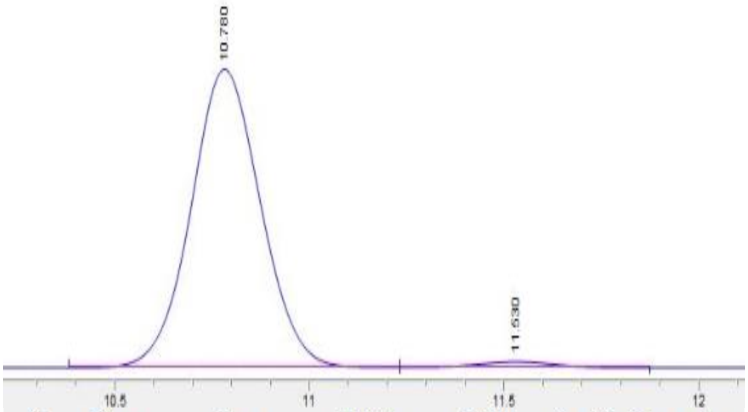

#	Time	Area	Height	Width	Area%	Symmetry
1	10.78	14509.4	1169	0.1908	98.038	0.903
2	11.53	290.3	21.6	0.2065	1.962	1.139

(*S*)-2-(2-(4-Methoxyphenyl)propyl)benzo[*d*]thiazole (4gd)

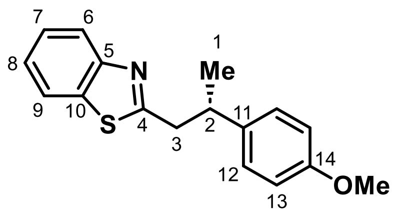

<u>**General procedure E**</u>: *tert*-Butyl 2-(benzo[*d*]thiazol-2-yl)acetate (24.9 mg, 0.10 mmol) and 1-methoxy-4-vinylbenzene (20.1 mg, 20.0 μL, 0.15 mmol) were employed with [Ir(cod)$_2$]BARF (6.36 mg, 0.005 mmol) and **L6** (1.91 mg, 0.005 mmol) in toluene (0.20 mL). The reaction was stirred at 100 °C for 12 h. Then *p*-toluenesulfonic acid monohydrate (5.71 mg, 0.03 mmol) was added and the resulting reaction mixture was heated at 130 °C for 3 h. Purification by flash column chromatography (Hexane/EtOAc = 15/1 to 7/1) afforded the title compound (26.0 mg, 92%, B:L > 25:1, 97:3 e.r.) as a

colorless oil. $[\alpha]_D^{23}$ = +108.4 (*c* 0.5, CH_2Cl_2). IR (thin film) ν_{max}/cm^{-1}: 2956, 2832, 1513, 1247, 1037, 757. ^1H NMR (500 MHz, $CDCl_3$) δ_H = 7.98 (dd, *J* = 8.3, 1.0 Hz, 1H, C**6**-H), 7.79 (dd, *J* = 8.2, 1.1 Hz, 1H, C**9**-H), 7.44 (ddd, *J* = 8.3, 7.2, 1.1 Hz, 1H, C**7**-H), 7.33 (ddd, *J* = 8.2, 7.2, 1.0 Hz, 1H, C**8**-H), 7.19 (d, *J* = 8.7 Hz, 2H, C**12**-H), 6.85 (d, *J* = 8.7 Hz, 2H, C**13**-H), 3.78 (s, 3H, OCH_3), 3.40 – 3.31 (m, 3H, C**2**-H + C**3**-H + C**3**-H'), 1.36 (d, *J* = 6.7 Hz, 3H, C**1**-H_3); ^{13}C NMR (125 MHz, $CDCl_3$) δ_C = 170.6 (C**4**), 158.3 (C**14**), 153.2 (C**5**), 137.6 (C**11**), 135.4 (C**10**), 128.0 (C**12**), 126.0 (C**7**), 124.8 (C**8**), 122.7 (C**6**), 121.6 (C**9**), 114.1 (C**13**), 55.3 (OCH_3), 43.1 (C**3**), 39.9 (C**2**), 22.2 (C**1**). HRMS (ESI^+) calculated for $C_{17}H_{18}NOS$ $[M+H]^+$ = 284.1103, found 284.1099.

SFC conditions: CHIRALPACK SB (25 cm), 90:10 CO_2:*i*-PrOH, 2.0 mL/min, 254 nm; *Retention times*: t_{major} = 7.9 min, t_{minor} = 8.9 min.

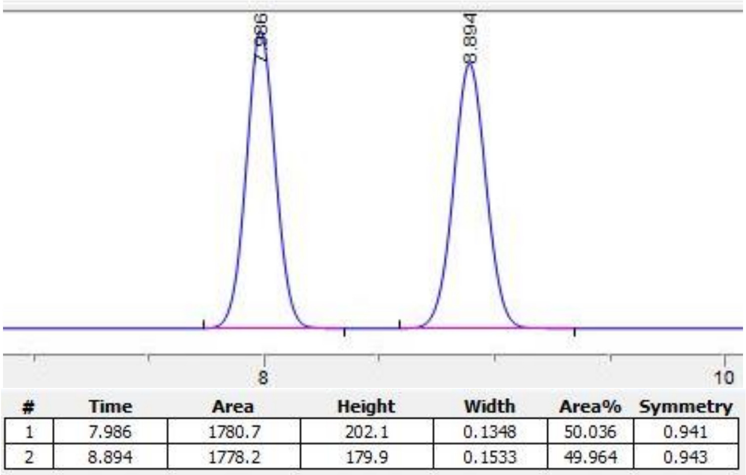

#	Time	Area	Height	Width	Area%	Symmetry
1	7.986	1780.7	202.1	0.1348	50.036	0.941
2	8.894	1778.2	179.9	0.1533	49.964	0.943

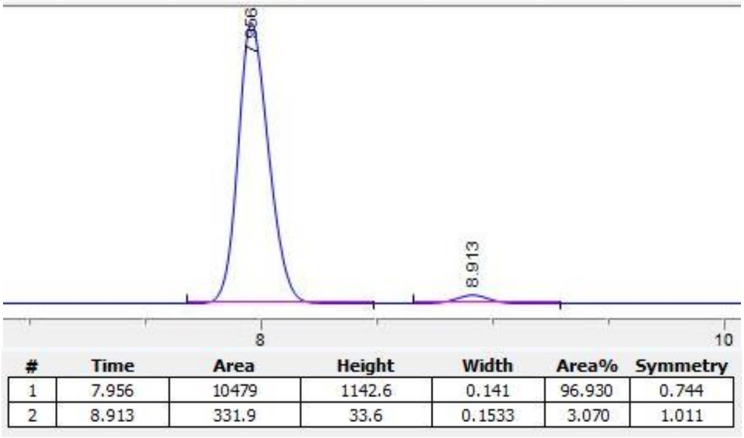

#	Time	Area	Height	Width	Area%	Symmetry
1	7.956	10479	1142.6	0.141	96.930	0.744
2	8.913	331.9	33.6	0.1533	3.070	1.011

(*S*)-2-(2-(4-Bromophenyl)propyl)benzo[*d*]thiazole (4ge)

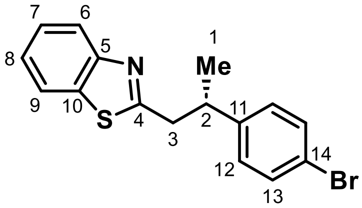

General procedure E: The preceding *tert*-butyl 2-(benzo[*d*]thiazol-2-yl)acetate (24.9 mg, 0.10 mmol) and 4-bromostyrene (36.6 mg, 19.6 μL, 0.20 mmol) were employed with $[Ir(cod)_2]BARF$ (6.36 mg, 0.005 mmol) and **L6** (1.91 mg, 0.005 mmol) in toluene (0.20 mL). The reaction was stirred at 100 °C for 12 h. Then *p*-toluenesulfonic acid monohydrate (5.71 mg, 0.03 mmol) was added and the resulting reaction mixture was heated at 130 °C for 3 h. Purification by flash column chromatography (Hexane/EtOAc = 100/1 to 20/1) afforded the title compound (24.0 mg, 72%, B:L > 25:1, 98:2 e.r.) as a yellow solid. m.p. 50 – 51 °C (EtOAc/hexane). $[\alpha]_D^{25}$ = +98.8 (*c* 0.5, CH_2Cl_2). IR (thin film) ν_{max}/cm^{-1}: 2962, 2925, 1518, 1489, 1435, 1126, 1009, 822. ^1H NMR (500 MHz, $CDCl_3$) δ_H = 7.97 (d, *J* = 8.2 Hz, 1H, C**6**-H), 7.79 (d, *J* = 7.6 Hz, 1H, C**9**-H), 7.46 – 7.39 (m, 3H, C**7**-H + 2 × C**13**-H), 7.35 – 7.31 (m, 1H, C**8**-H), 7.15 – 7.11 (d, *J* = 8.5 Hz, 2H, C**12**-H), 3.42 – 3.29 (m, 3H, C**2**-H + C**3**-H + C**3**-H'), 1.37 (d, *J* = 6.5 Hz, 3H, C**1**-H_3); ^{13}C NMR (125 MHz, $CDCl_3$) δ_C = 169.9 (C**4**), 153.2 (C**5**), 144.4 (C**11**), 135.2 (C**10**), 131.8 (C**13**), 128.9 (C**12**), 126.0 (C**7**), 124.9 (C**8**), 122.7 (**C6**), 121.6 (C**9**), 120.4 (C**14**), 42.6 (C**3**), 40.1 (C**2**), 21.9 (C**1**). HRMS (ESI^+) calculated for $C_{16}H_{15}BrNS$ $[M+H]^+$ = 332.0103, found 332.0097.

SFC conditions: CHIRALPACK SB (25 cm), 90:10 CO_2:*i*-PrOH, 2.0 mL/min, 254 nm; *Retention times*: t_{major} = 9.9 min, t_{minor} = 11.7 min.

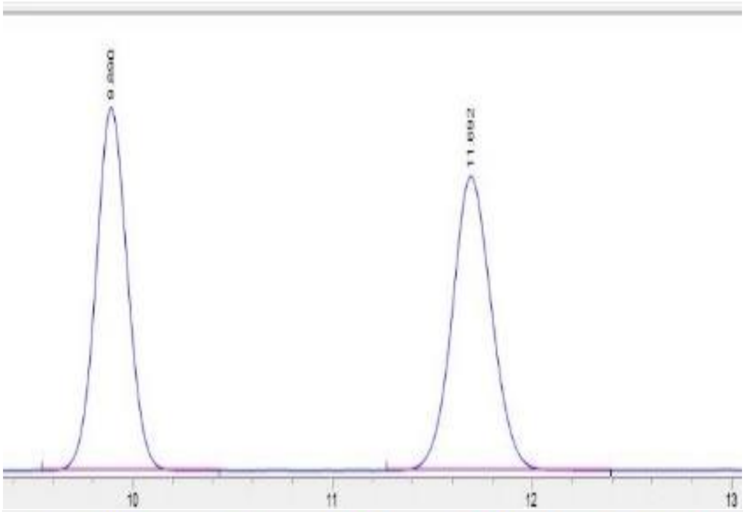

#	Time	Area	Height	Width	Area%	Symmetry
1	9.89	3054.4	278.5	0.17	49.511	0.921
2	11.692	3114.7	225.7	0.213	50.489	0.875

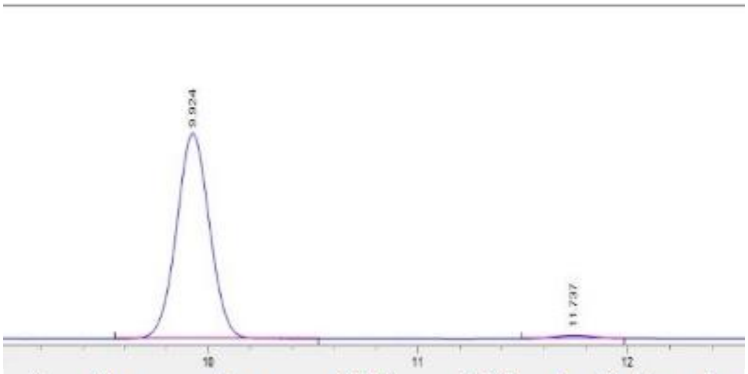

#	Time	Area	Height	Width	Area%	Symmetry
1	9.924	2035.7	185.3	0.1702	98.125	0.943
2	11.737	38.9	3.1	0.189	1.875	0.995

(*S*)-2-(2-(4-Fluorophenyl)propyl)benzo[*d*]thiazole (4gf)

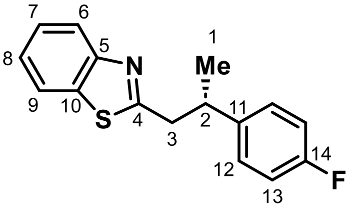

<u>General procedure E</u>: The preceding *tert*-butyl 2-(benzo[*d*]thiazol-2-yl)acetate (24.9 mg, 0.10 mmol) and 4-fluorostyrene (24.4 mg, 17.9 μL, 0.20 mmol) were employed with [Ir(cod)$_2$]BARF (6.36 mg, 0.005 mmol) and **L6** (1.91 mg, 0.005 mmol) in toluene (0.20 mL). The reaction was stirred at 100 °C for 12 h. Then *p*-toluenesulfonic acid monohydrate (5.71 mg, 0.03 mmol) was added and the resulting reaction mixture was heated at 130 °C for 3 h. Purification by flash column chromatography (Hexane/EtOAc = 100/1 to 20/1) afforded the title compound (23.1 mg, 85%, B:L > 25:1, 98:2 e.r.) as a colorless oil. $[\alpha]_D^{25}$ = +79.4 (*c* 0.5, CH_2Cl_2). IR (thin film) ν_{max}/cm^{-1}: 2960, 2927, 1603, 1510, 1434, 1223, 1161, 835. ^{1}H NMR (500 MHz, $CDCl_3$) δ_H = 7.97 (d, *J* = 8.3 Hz, 1H, C**6**-<u>H</u>), 7.80 (d, *J* = 8.0 Hz, 1H, C**9**-<u>H</u>), 7.47 – 7.42 (m, 1H, C**7**-<u>H</u>), 7.36 – 7.31 (m, 1H, C**8**-<u>H</u>), 7.23 – 7.19 (m, 2H, C**12**-<u>H</u>), 7.00 – 6.95 (m, 2H, C**13**-<u>H</u>), 3.44 – 3.30 (m, 3H, C**2**-<u>H</u> + C**3**-<u>H</u> + C**3**-<u>H’</u>), 1.37 (d, *J* = 6.6 Hz, 3H, C**1**-<u>H$_3$</u>); ^{13}C NMR (125 MHz, $CDCl_3$) δ_C = 170.1 (C**4**), 161.7 (d, $^1J_{CF}$ = 242.9 Hz, C**14**), 153.2 (C**5**), 141.1 (d, $^4J_{CF}$ = 3.1 Hz, C**11**), 135.3 (C**10**), 128.5 (d, $^3J_{CF}$ = 7.7 Hz, C**12**), 126.0 (C**7**), 124.9 (C**8**), 122.7 (C**6**), 121.6 (C**9**), 115.5 (d, $^2J_{CF}$ = 20.9 Hz, C**13**), 43.0 (C**3**), 40.0 (C**2**), 22.1 (C**1**); ^{19}F NMR (471 MHz, $CDCl_3$) δ_F = -116.5 – -116.6 (m, 1F). HRMS (ESI$^+$) calculated for $C_{16}H_{15}FNS$ $[M+H]^+$ = 272.0904, found 272.0914.

SFC conditions: CHIRALPACK SB (25 cm), 90:10 CO_2:*i*-PrOH, 2.0 mL/min, 254 nm; *Retention times*: t_{major} = 5.6 min, t_{minor} = 6.4 min.

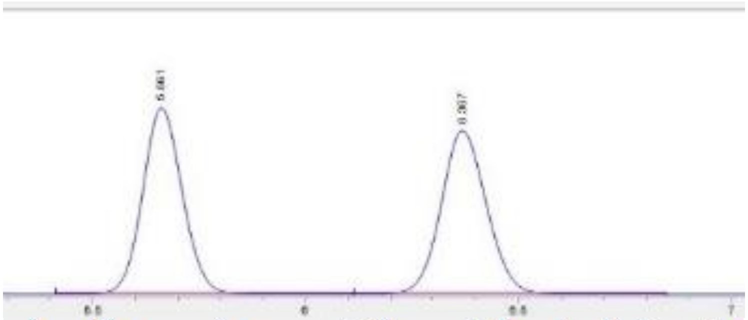

#	Time	Area	Height	Width	Area%	Symmetry
1	5.661	5148.7	797.1	0.1014	49.937	0.878
2	6.367	5161.7	699.1	0.1141	50.063	0.862

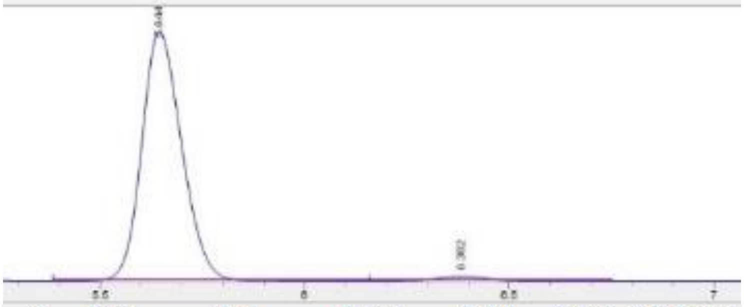

#	Time	Area	Height	Width	Area%	Symmetry
1	5.644	12969.7	1925.6	0.1046	98.101	0.757
2	6.382	251.1	34.3	0.1133	1.899	1.005

(*S*)-2-(2-(2-Fluorophenyl)propyl)benzo[*d*]thiazole (4gg)

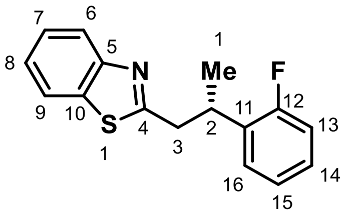

<u>General procedure E</u>: The preceding *tert*-butyl 2-(benzo[*d*]thiazol-2-yl)acetate (24.9 mg, 0.10 mmol) and 2-fluorostyrene (24.4 mg, 17.9 µL, 0.20 mmol) were employed with [Ir(cod)$_2$]BARF (6.36 mg, 0.005 mmol) and **L6** (1.91 mg, 0.005 mmol) in toluene (0.20 mL). The reaction was stirred at 100 °C for 12 h. Then *p*-toluenesulfonic acid monohydrate (5.71 mg, 0.03 mmol) was added and the resulting reaction mixture was heated at 130 °C for 3 h. Purification by flash column chromatography (Hexane/EtOAc = 100/1 to 20/1) afforded the title compound (21.0 mg, 77%, B:L > 25:1, 97:3 e.r.) as a yellow solid. m.p. 48 – 49 °C (EtOAc/hexane). $[\alpha]_D^{25}$ = +49.5 (*c* 0.5, CH_2Cl_2). IR (thin film) ν_{max}/cm^{-1}: 2967, 2927, 1583, 1517, 1491, 1220, 1087, 935. ^{1}H NMR (500 MHz, $CDCl_3$) δ_H = 7.98 (d, *J* = 8.1 Hz, 1H, C**6**-<u>H</u>), 7.80 (d, *J* = 8.0 Hz, 1H, C**9**-<u>H</u>), 7.46 – 7.42 (m, 1H, C**7**-<u>H</u>), 7.36 – 7.31 (m, 1H, C**8**-<u>H</u>), 7.30 – 7.25 (m, 1H, C**14**-<u>H</u> or C**16**-<u>H</u>), 7.22 – 7.16 (m, 1H, C**14**-<u>H</u> or C**16**-<u>H</u>), 7.11 – 7.06 (m, 1H, C**15**-<u>H</u>), 7.05 – 7.00 (m, 1H, C**13**-<u>H</u>), 3.75 – 3.66 (m, 1H, C**2**-<u>H</u>), 3.49 (dd, *J* = 14.6, 7.0 Hz, 1H, C**3**-<u>H</u>), 3.39 (dd, *J* = 14.6, 8.2 Hz, 1H, C**3**-<u>H'</u>), 1.40 (d, *J* = 7.0 Hz, 3H, C**1**-<u>H$_3$</u>); ^{13}C NMR (125 MHz, $CDCl_3$) δ_C = 170.2 (C**4**), 160.9 (d, $^1J_{CF}$ = 244.0 Hz, C**12**), 153.2 (C**5**), 135.4 (C**10**), 132.1 (d, $^2J_{CF}$ = 14.0 Hz, C**11**), 128.4 (d, $^3J_{CF}$ = 5.0 Hz, C**14** or C**16**), 128.2 (d, $^3J_{CF}$ = 8.3 Hz, C**14** or C**16**), 126.0 (C**7**), 124.8 (C**8**),

124.4 (d, $^4J_{CF}$ = 3.5 Hz, C**15**), 122.8 (C**6**), 121.6 (C**9**), 115.8 (d, $^2J_{CF}$ = 22.5 Hz, C**13**), 41.2 (d, $^4J_{CF}$ = 1.7 Hz, C**3**), 34.4 (d, $^3J_{CF}$ = 1.7 Hz, C**2**), 20.5 (C**1**); ^{19}F NMR (471 MHz, $CDCl_3$) δ_F = -117.9 – -118.0 (m, 1F). HRMS (ESI$^+$) calculated for $C_{16}H_{15}FNS$ $[M+H]^+$ = 272.0904, found 272.0906.

SFC conditions: CHIRALPACK SB (25 cm), 90:10 CO_2:*i*-PrOH, 2.0 mL/min, 254 nm; *Retention times*: t_{major} = 15.2 min, t_{minor} = 16.2 min.

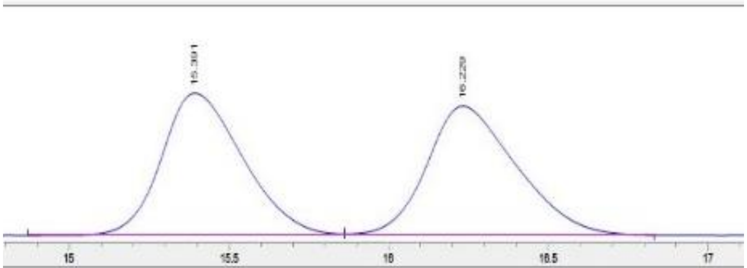

#	Time	Area	Height	Width	Area%	Symmetry
1	15.391	3340.8	188.5	0.2727	49.995	0.681
2	16.229	3341.4	171.3	0.2896	50.005	0.659

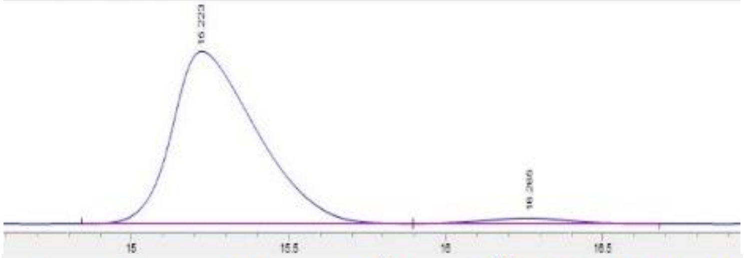

#	Time	Area	Height	Width	Area%	Symmetry
1	15.223	5808.5	309.2	0.2892	96.808	0.528
2	16.265	191.5	10.3	0.2503	3.192	1.125

(*S*)-2-(2-(Naphthalen-2-yl)propyl)benzo[*d*]thiazole (4gh)

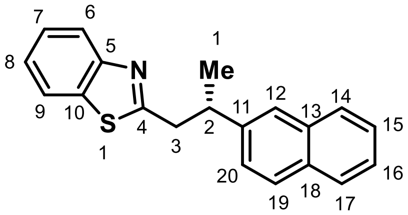

<u>**General procedure E**</u>: The preceding *tert*-butyl 2-(benzo[*d*]thiazol-2-yl)acetate (24.9 mg, 0.10 mmol) and 2-vinylnaphthalene (30.8 mg, 0.20 mmol) were employed with $[Ir(cod)_2]$BARF (6.36 mg, 0.005 mmol) and **L6** (1.91 mg, 0.005 mmol) in toluene (0.20 mL). The reaction was stirred at 100 °C for 12 h. Then *p*-toluenesulfonic acid monohydrate (5.71 mg, 0.03 mmol) was added and the resulting reaction mixture was heated at 130 °C for 3 h. Purification by flash column chromatography (Hexane/EtOAc = 100/1 to 20/1) afforded the title compound (25.2 mg, 83%, B:L > 25:1, 98.5:1.5 e.r.) as a yellow solid. m.p. 79 – 80 °C (EtOAc/hexane). $[\alpha]_D^{25}$ = +131.7 (*c* 0.5, CH_2Cl_2). IR (thin film) ν_{max}/cm^{-1}: 2961, 2923, 1600, 1509, 1435, 1125, 1057, 951. ^{1}H NMR (500 MHz, $CDCl_3$) δ_H = 7.99 (d, *J* = 8.1 Hz, 1H, C**6**-<u>H</u>), 7.84 – 7.75 (m, 4H, C**9**-<u>H</u> + 3 × ArC<u>H</u>), 7.72 (s, 1H, C**12**-<u>H</u>), 7.49 – 7.41 (m, 4H, C**7**-<u>H</u> + C**20**-<u>H</u> + 2 × ArC<u>H</u>), 7.35 – 7.30 (m, 1H, C**8**-<u>H</u>), 3.63 – 3.51 (m, 2H, C**2**-H + C**3**-<u>H</u>), 3.45 (dd, *J* = 14.1, 7.5 Hz, 1H,

C**3**-H̲’), 1.48 (d, J = 6.8 Hz, 3H, C**1**-$\underline{H_3}$); ^{13}C NMR (125 MHz, $CDCl_3$) δ_C = 170.4 (C**4**), 153.2 (C**5**), 143.0 (C**11**), 135.4 (C**10**), 133.7 (C**13** or C**18**), 132.5 (C**13** or C**18**), 128.4 (ArC̲H), 127.8 (ArC̲H), 127.7 (ArC̲H), 126.1 (ArC̲H), 126.0 (C**7**), 125.7 (ArC̲H), 125.6 (ArC̲H), 125.5 (ArC̲H), 124.8 (C**8**), 122.7 (C**6**), 121.6 (C**9**), 42.7 (C**3**), 40.8 (C**2**), 22.0 (C**1**). HRMS (ESI$^+$) calculated for $C_{20}H_{18}NS$ $[M+H]^+$ = 304.1155, found 304.1153.

SFC conditions: CHIRALPACK SB (25 cm), 90:10 CO_2:*i*-PrOH, 2.0 mL/min, 254 nm; *Retention times*: t_{major} = 16.9 min, t_{minor} = 17.7 min.

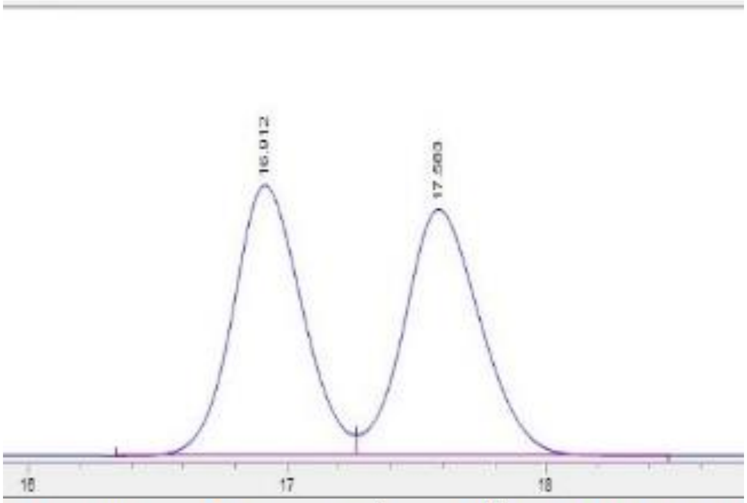

#	Time	Area	Height	Width	Area%	Symmetry
1	16.912	3942.4	211.6	0.2893	49.979	0.85
2	17.583	3945.7	192.9	0.3107	50.021	0.804

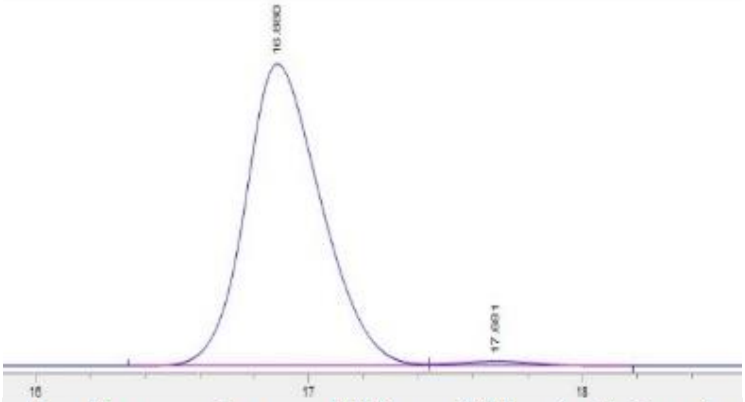

#	Time	Area	Height	Width	Area%	Symmetry
1	16.88	7981.3	412.1	0.296	98.406	0.721
2	17.681	129.3	6.5	0.2875	1.594	0.964

(*S*)-2-(2-(Ferrocenyl)propyl)benzo[*d*]thiazole (4gi)

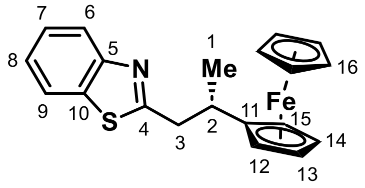

General procedure E: The preceding *tert*-butyl 2-(benzo[*d*]thiazol-2-yl)acetate (24.9 mg, 0.10 mmol) and vinylferrocene (42.4 mg, 0.20 mmol) were employed with [Ir(cod)$_2$]BARF (6.36 mg, 0.005 mmol) and **L6** (1.91 mg, 0.005 mmol) in toluene (0.20 mL). The reaction was stirred at 100 °C for 12 h. Then *p*-toluenesulfonic acid monohydrate (5.71 mg, 0.03 mmol) was added and the resulting reaction mixture was heated at 130 °C for 3 h. Purification by flash column chromatography (Hexane/EtOAc = 100/1 to 20/1) afforded the title compound (26.7 mg, 74%, B:L > 25:1, 60.5:39.5 e.r.) as a brown oil. $[\alpha]_D^{25}$ = -5.5 (*c* 0.5, CH_2Cl_2). IR (thin film) ν_{max}/cm^{-1}: 2962, 2925, 1518, 1454, 1435, 1312, 1105, 818. ^{1}H NMR (500 MHz, $CDCl_3$) δ_H = 8.00 (d, *J* = 8.2 Hz, 1H, C**6**-H), 7.84 (d, *J* = 8.5 Hz, 1H, C**9**-H), 7.49 – 7.44 (m, 1H, C**7**-H), 7.39 – 7.34 (m, 1H, C**8**-H), 4.16 (s, 5H, C**16**-H), 4.14 – 4.12 (m, 1H, C**12**-H), 4.12 – 4.10 (m, 1H, C**13**-H), 4.10 – 4.08 (m, 1H, C**14**-H), 4.08 – 4.05 (m, 1H, C**15**-H), 3.39 (dd, *J* = 13.8, 4.7 Hz, 1H, C**3**-H), 3.23 – 3.15 (m, 1H, C**2**-H), 3.12 (dd, *J* = 13.8, 9.2 Hz, 1H, C**3**-H'), 1.33 (d, *J* = 6.6 Hz, 3H, C**1**-H_3); ^{13}C NMR (125 MHz, $CDCl_3$) δ_C = 170.7 (C**4**), 153.4 (C**5**), 135.3 (C**10**), 126.0 (C**7**), 124.8 (C**8**), 122.7 (C**6**), 121.6 (C**9**), 94.2 (C**11**), 68.6 (C**16**), 67.5 (C**12**), 67.3 (C**13**), 67.1 (C**14**), 66.1 (C**15**), 43.4 (C**3**), 34.4 (C**2**), 20.4 (C**1**). HRMS (ESI$^+$) calculated for $C_{20}H_{20}FeNS$ $[M+H]^+$ = 362.0661, found 362.0649.

SFC conditions: CHIRALPACK SB (25 cm), 90:10 CO_2:*i*-PrOH, 2.0 mL/min, 254 nm; *Retention times*: t_{major} = 16.9 min, t_{minor} = 19.0 min.

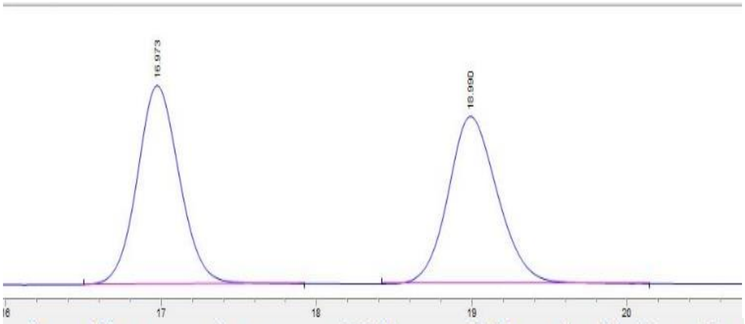

#	Time	Area	Height	Width	Area%	Symmetry
1	16.973	2725.8	144	0.2908	50.145	0.864
2	18.99	2710	121.4	0.3385	49.855	0.797

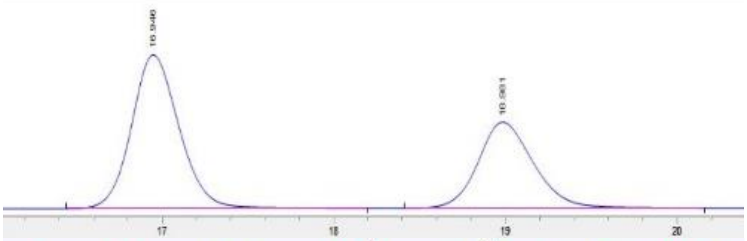

#	Time	Area	Height	Width	Area%	Symmetry
1	16.946	4232.4	219.9	0.2946	60.526	0.812
2	18.981	2760.3	123.3	0.3392	39.474	0.768

(*R*)-2-(2-Methylhexyl)benzo[*d*]thiazole (4gj)

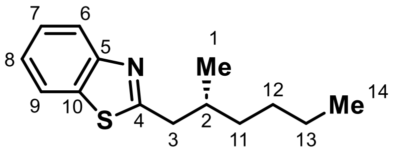

<u>General procedure E</u>: *tert*-Butyl 2-(benzo[*d*]thiazol-2-yl)acetate (24.9 mg, 0.10 mmol) and hex-1-ene (12.6 mg, 18.8 µL, 0.15 mmol) were employed with $[Ir(cod)_2]BARF$ (6.36 mg, 0.005 mmol) and **L6** (1.91 mg, 0.005 mmol) in *m*-xylene (0.20 mL). The reaction was stirred at 70 °C for 48 h. Then *p*-toluenesulfonic acid monohydrate (5.71 mg, 0.03 mmol) was added and the resulting reaction mixture was heated at 130 °C for 3 h. Purification by flash column chromatography (Hexane/EtOAc = 20/1 to 9/1) afforded the title compound (21.0 mg, 90%, B:L > 25:1, 93.5:6.5 e.r.) as a colorless oil. $[\alpha]_D^{25}$ = +8.03 (*c* 0.5, CH_2Cl_2). IR (thin film) ν_{max}/cm^{-1}: 2936, 2822, 1453, 1147, 1007, 857. ^1H NMR (500 MHz, $CDCl_3$) δ_H = 7.98 (dd, *J* = 8.4, 1.2 Hz, 1H, C**6**-<u>H</u>), 7.84 (dd, *J* = 8.2, 1.2 Hz, 1H, C**9**-<u>H</u>), 7.45 (ddd, *J* = 8.4, 7.2, 1.2 Hz, 1H, C**7**-<u>H</u>), 7.34 (ddd, *J* = 8.2, 7.2, 1.2 Hz, 1H, C**8**-<u>H</u>), 3.11 (dd, *J* = 14.4, 6.1 Hz, 1H, C**3**-<u>H</u>), 2.91 (dd, *J* = 14.4, 8.2 Hz, 1H, C**3**-<u>H’</u>), 2.12 – 2.03 (m, 1H, C**2**-<u>H</u>), 1.49 – 1.42 (m, 1H, C**11**-<u>H</u>), 1.42 – 1.36 (m, 1H, C**12**-<u>H</u>), 1.34 – 1.25 (m, 4H, C**11**-<u>H’</u> + C**12**-<u>H’</u> + C**13**-<u>H_2</u>), 1.00 (d, *J* = 6.6 Hz, 3H, C**1**-<u>H_3</u>), 0.89 (t, *J* = 7.1 Hz, 3H, C**14**-<u>H_3</u>); ^{13}C NMR (125 MHz, $CDCl_3$) δ_C = 171.7 (C**4**), 153.4 (C**5**), 135.4 (C**10**), 126.0 (C**7**), 124.7 (C**8**), 122.7 (C**6**), 121.6 (C**9**), 41.9 (C**3**), 36.5 (C**11**), 34.6 (C**2**), 29.3 (C**12**), 23.0 (C**13**), 19.7 (C**1**), 14.2 (C**14**). HRMS (ESI^+) calculated for $C_{14}H_{20}NS$ $[M+H]^+$ = 234.1311, found 234.1313.

SFC conditions: CHIRALPACK SC (25 cm), 98:2 CO_2:*i*-PrOH, 1.5 mL/min, 254 nm; *Retention times*: t_{minor} = 11.8 min, t_{major} = 12.5 min.

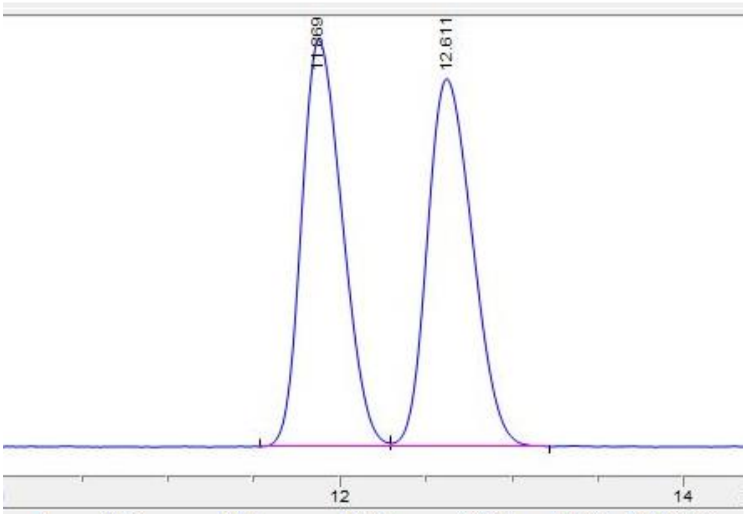

#	Time	Area	Height	Width	Area%	Symmetry
1	11.869	3438.6	209	0.2559	49.984	0.714
2	12.611	3440.8	189	0.2884	50.016	0.724

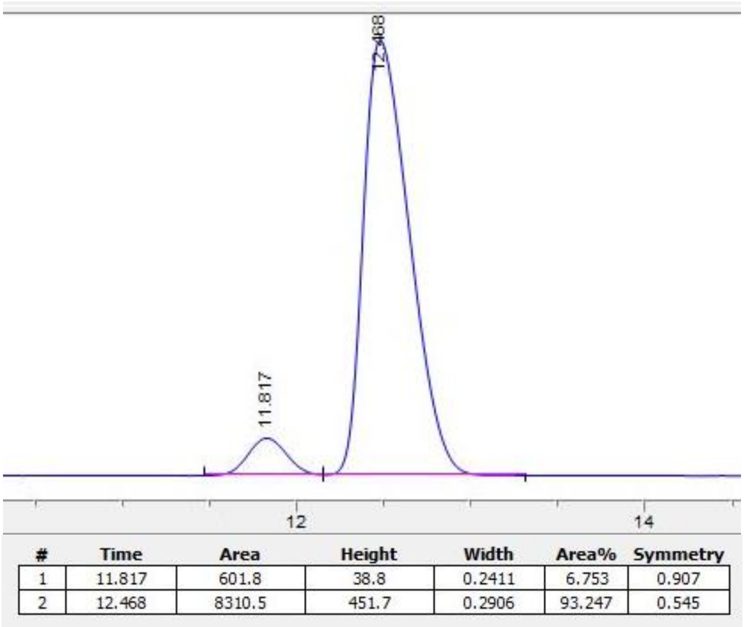

#	Time	Area	Height	Width	Area%	Symmetry
1	11.817	601.8	38.8	0.2411	6.753	0.907
2	12.468	8310.5	451.7	0.2906	93.247	0.545

(*R*)-2-(2,4-Dimethylpentyl)benzo[*d*]thiazole (4gk)

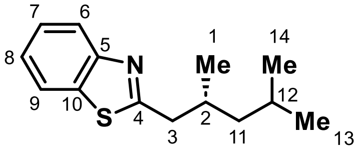

<u>General procedure E</u>: *tert*-Butyl 2-(benzo[*d*]thiazol-2-yl)acetate (24.9 mg, 0.10 mmol) and 4-methylpent-1-ene (12.6 mg, 19.0 µL, 0.15 mmol) were employed with $[Ir(cod)_2]$BARF (6.36 mg, 0.005 mmol) and **L6** (1.91 mg, 0.005 mmol) in *m*-xylene (0.20 mL). The reaction was stirred at 70 °C for 84 h. Then *p*-toluenesulfonic acid monohydrate (5.71 mg, 0.03 mmol) was added and the resulting reaction mixture was heated at 130 °C for 3 h. Purification by flash column chromatography (Hexane/EtOAc = 20/1 to 9/1) afforded the title compound (22.9 mg, 98%, B:L > 25:1, 93:7 e.r.) as a colorless oil. $[\alpha]_D^{23}$ = +12.5 (*c* 0.5, CH_2Cl_2). IR (thin film) ν_{max}/cm^{-1}: 2956, 2844, 1450, 1167, 1001, 757. ^{1}H NMR (500 MHz, $CDCl_3$) δ_H = 7.98 (d, *J* = 8.1 Hz, 1H, C**6**-<u>H</u>), 7.84 (dd, *J* = 8.0, 1.1 Hz, 1H, C**9**-<u>H</u>), 7.47 – 7.41 (m, 1H, C**7**-<u>H</u>), 7.38 – 7.31 (m, 1H, C**8**-<u>H</u>), 3.09 (dd, *J* = 14.4, 6.0 Hz, 1H, C**3**-<u>H</u>), 2.90 (dd, *J* = 14.4, 8.2 Hz, 1H, C**3**-<u>H’</u>), 2.19 – 2.10 (m, 1H, C**2**-<u>H</u>), 1.76 – 1.67 (m, 1H, C**12**-<u>H</u>), 1.26 (ddd, *J* = 13.9, 8.5, 5.6 Hz, 1H, C**11**-<u>H</u>), 1.18 (ddd, *J* = 13.9, 8.7, 5.9 Hz, 1H, C**11**-<u>H’</u>), 0.98 (d, *J* = 6.6 Hz, 3H, C**1**-<u>H$_3$</u>), 0.91 (d, *J* = 6.6 Hz, 3H, C**13**-<u>H$_3$</u>), 0.87 (d, *J* = 6.5 Hz, 3H, C**14**-<u>H$_3$</u>); ^{13}C NMR (125 MHz, $CDCl_3$) δ_C = 171.6 (C**4**), 153.4 (C**5**), 135.4 (C**10**), 126.0 (C**7**), 124.7 (C**8**), 122.7 (C**6**), 121.6 (C**9**), 46.3 (C**11**), 42.2 (C**3**), 32.3 (C**2**), 25.4 (C**12**), 23.5 (C**13**), 22.1 (C**14**), 19.7 (C**1**). HRMS (ESI$^+$) calculated for $C_{14}H_{20}NS$ $[M+H]^+$ = 234.1311, found 234.1318.

SFC conditions: CHIRALPACK IE (25 cm), 98.5:1.5 CO_2:*i*-PrOH, 1.0 mL/min, 254 nm; *Retention times*: t_{minor} = 23.5 min, t_{major} = 24.3 min.

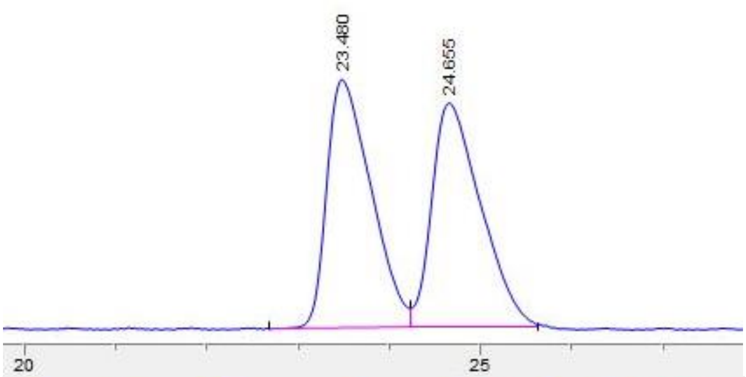

#	Time	Area	Height	Width	Area%	Symmetry
1	23.48	4503.7	133.1	0.4489	49.934	0.503
2	24.655	4515.7	120.2	0.5083	50.066	0.534

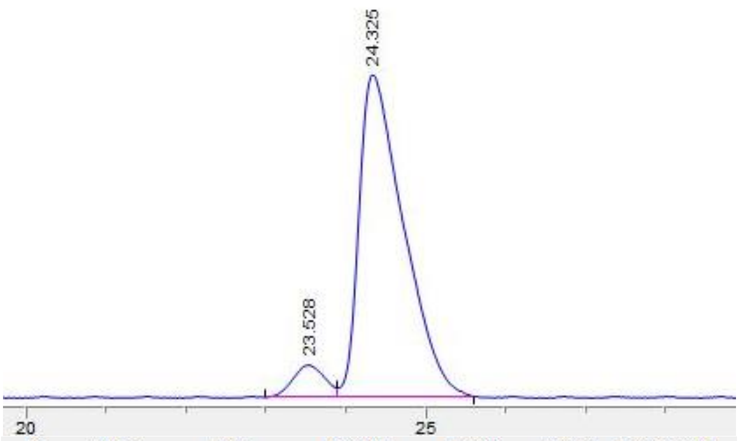

#	Time	Area	Height	Width	Area%	Symmetry
1	23.528	484.8	17.3	0.3334	6.791	0.97
2	24.325	6654.9	171.5	0.5067	93.209	0.401

(*S*)-2-(2,3-Dimethylbutyl)benzo[*d*]thiazole (4gl)

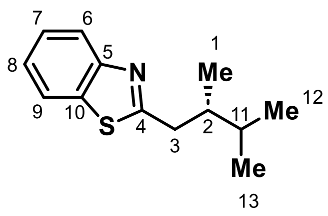

__General procedure E__: *tert*-Butyl 2-(benzo[*d*]thiazol-2-yl)acetate (24.9 mg, 0.10 mmol) and 3-methylbut-1-ene (21.0 mg, 33.6 μL, 0.30 mmol) were employed with [Ir(cod)$_2$]BARF (6.36 mg, 0.005 mmol) and **L6** (1.91 mg, 0.005 mmol) in *m*-xylene (0.20 mL). The reaction was stirred at 70 °C for 132 h. Then *p*-toluenesulfonic acid monohydrate (5.71 mg, 0.03 mmol) was added and the resulting reaction mixture was heated at 130 °C for 3 h. Purification by flash column chromatography (Hexane/EtOAc = 15/1 to 9/1) afforded the title compound (17.0 mg, 78%, B:L > 25:1, 97.5:2.5 e.r.) as a colorless oil.

$[\alpha]_D^{22}$ = -21.6 (*c* 0.5, CH_2Cl_2). IR (thin film) ν_{max}/cm^{-1}: 2958, 2873, 1436, 1142, 1001, 758. ^{1}H NMR (500 MHz, $CDCl_3$) δ_H = 7.97 (dd, *J* = 8.2, 1.1 Hz, 1H, C**6**-H), 7.84 (dd, *J* = 8.1, 1.1 Hz, 1H, C**9**-H), 7.44 (ddd, *J* = 8.2, 7.3, 1.1 Hz, 1H, C**7**-H), 7.34 (ddd, *J* = 8.1, 7.3, 1.1 Hz, 1H, C**8**-H), 3.16 (dd, *J* = 14.5, 5.4 Hz, 1H, C**3**-H), 2.89 (dd, *J* = 14.5, 9.4 Hz, 1H, C**3**-H'), 2.07 – 1.98 (m, 1H, C**2**-H), 1.76 – 1.70 (m, 1H, C**11**-H), 0.96 (d, *J* = 6.9 Hz, 3H, C**1**-H_3), 0.93 (d, *J* = 6.5 Hz, 3H, C**12**-H_3), 0.91 (d, *J* = 6.5 Hz, 3H, C**13**-H_3); ^{13}C NMR (125 MHz, $CDCl_3$) δ_C = 172.2 (C**4**), 153.4 (C**5**), 135.4 (C**10**), 126.0 (C**7**), 124.7 (C**8**), 122.6 (C**6**), 121.6 (C**9**), 40.3 (C**2**), 39.2 (C**3**), 32.1 (C**11**), 20.3 (C**1**), 18.0 (C**12**), 15.3 (C**13**). HRMS (ESI^+) calculated for $C_{13}H_{18}NS$ $[M+H]^+$ = 220.1154, found 220.1160.

SFC conditions: CHIRALPACK IE (25 cm), 98.5:1.5 CO_2:*i*-PrOH, 1.0 mL/min, 254 nm; *Retention times*: t_{minor} = 26.4 min, t_{major} = 27.2 min.

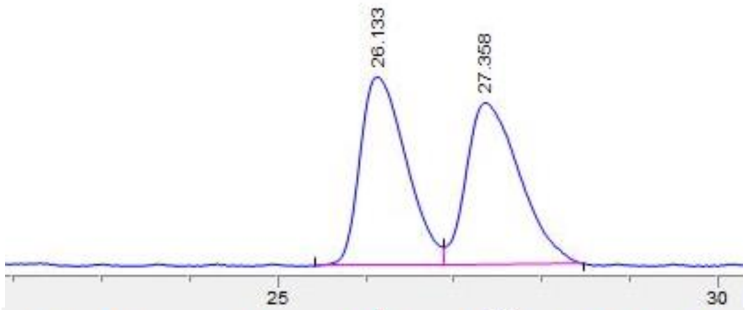

#	Time	Area	Height	Width	Area%	Symmetry
1	26.133	2836.5	77.2	0.4328	49.985	0.582
2	27.358	2838.2	66.6	0.5035	50.015	0.523

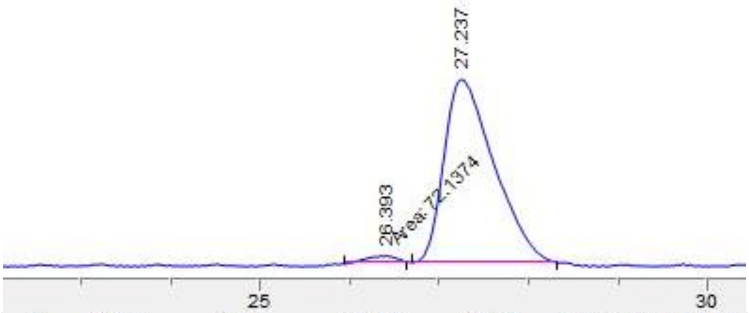

#	Time	Area	Height	Width	Area%	Symmetry
1	26.393	72.1	3.2	0.3808	2.459	1.713
2	27.237	2861.7	73.3	0.4693	97.541	0.53

(S)-2-(2,3,3-Trimethylbutyl)benzo[d]thiazole (4gm)

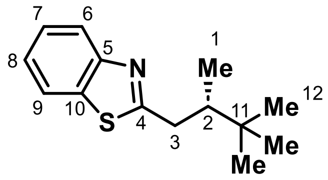

<u>General procedure E</u>: *tert*-Butyl 2-(benzo[*d*]thiazol-2-yl)acetate (24.9 mg, 0.10 mmol) and 3,3-dimethylbut-1-ene (25.2 mg, 38.6 μL, 0.30 mmol) were employed with [Ir(cod)$_2$]BARF (6.36 mg, 0.005 mmol) and **L6** (1.91 mg, 0.005 mmol) in *m*-xylene (0.20 mL). The reaction was stirred at 70 °C for 180 h. Then *p*-toluenesulfonic acid monohydrate (5.71 mg, 0.03 mmol) was added and the resulting reaction mixture was heated at 130 °C for 3 h. Purification by flash column chromatography (Hexane/EtOAc = 15/1 to 9/1) afforded the title compound (13.7 mg, 59%, B:L > 25:1, 97:3 e.r.) as a colorless oil. $[\alpha]_D^{22}$ = -46.9 (c 0.5, CH_2Cl_2). IR (thin film) ν_{max}/cm^{-1}: 2961, 2809, 1432, 1244, 1143, 758. ^{1}H NMR (500 MHz, $CDCl_3$) δ_H = 7.98 (d, J = 8.2 Hz, 1H, C**6**-<u>H</u>), 7.84 (dd, J = 8.2, 1.2 Hz, 1H, C**9**-<u>H</u>), 7.44 (ddd, J = 8.2, 7.2, 1.2 Hz, 1H, C**7**-<u>H</u>), 7.34 (ddd, J = 8.2, 7.2, 1.2 Hz, 1H, C**8**-<u>H</u>), 3.32 (dd, J = 14.3, 3.1 Hz, 1H, C**3**-<u>H</u>), 2.75 (dd, J = 14.3, 11.4 Hz, 1H, C**3**-<u>H'</u>), 1.95 – 1.85 (m, 1H, C**2**-<u>H</u>), 0.99 (s, 9H, 3 × C**12**-<u>H$_3$</u>), 0.90 (d, J = 6.7 Hz, 3H, C**1**-<u>H$_3$</u>); ^{13}C NMR (125 MHz, $CDCl_3$) δ_C = 173.0 (C**4**), 153.4 (C**5**), 135.4 (C**10**), 126.0 (C**7**), 124.7 (C**8**), 122.6 (C**6**), 121.6 (C**9**), 44.6 (C**3**), 37.2 (C**11**), 33.4 (C**2**), 27.4 (C**12**), 14.5 (C**1**). HRMS (ESI$^+$) calculated for $C_{14}H_{20}NS$ $[M+H]^+$ = 234.1311, found 234.1313.

SFC conditions: CHIRALPACK IE (25 cm), 98:2 CO_2:i-PrOH, 1.5 mL/min, 254 nm; *Retention times*: t_{minor} = 13.0 min, t_{major} = 14.3 min.

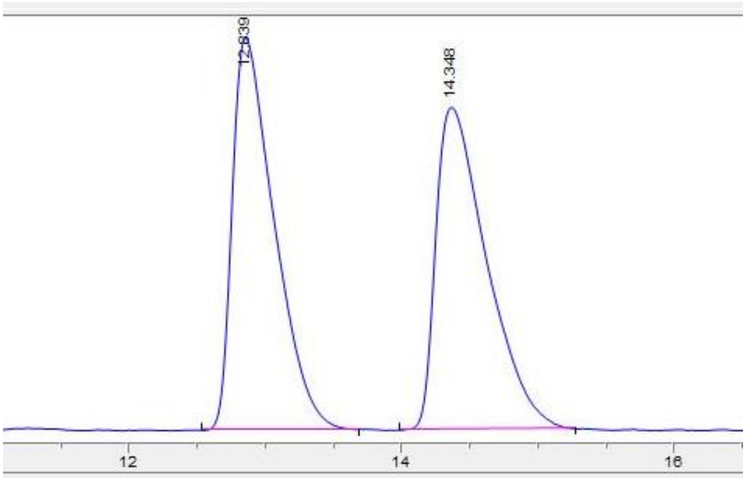

#	Time	Area	Height	Width	Area%	Symmetry
1	12.839	3274.6	152.9	0.3138	50.096	0.441
2	14.348	3262	125.7	0.3727	49.904	0.418

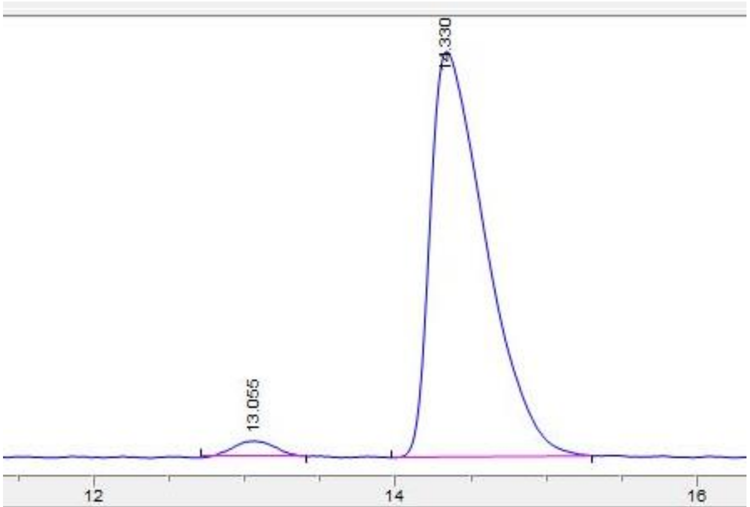

#	Time	Area	Height	Width	Area%	Symmetry
1	13.055	90.1	4.4	0.2488	3.056	1.003
2	14.33	2859.2	111.5	0.3572	96.944	0.398

(*R*)-2-(2-Methyl-3-phenylpropyl)benzo[*d*]thiazole (4gn)

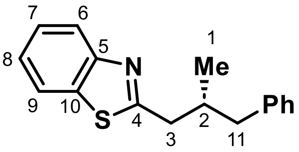

<u>**General procedure E**</u>: *tert*-Butyl 2-(benzo[*d*]thiazol-2-yl)acetate (24.9 mg, 0.10 mmol) and allylbenzene (17.7 mg, 19.9 µL, 0.15 mmol) were employed with [Ir(cod)$_2$]BARF (6.36 mg, 0.005 mmol) and **L6** (1.91 mg, 0.005 mmol) in *m*-xylene (0.20 mL). The reaction was stirred at 70 °C for 72 h. Then *p*-toluenesulfonic acid monohydrate (5.71 mg, 0.03 mmol) was added and the resulting reaction mixture was heated at 130 °C for 3 h. Purification by flash column chromatography (Hexane/EtOAc = 15/1 to 9/1) afforded the title compound (24.4 mg, 91%, B:L > 25:1, 91:9 e.r.) as a colorless oil. $[\alpha]_D^{23}$ = -6.51 (*c* 0.5, CH_2Cl_2). IR (thin film) ν_{max}/cm^{-1}: 2921, 2851, 1455, 1245, 1125, 758. ^{1}H NMR (500 MHz, $CDCl_3$) δ_H = 8.04 (d, *J* = 8.1 Hz, 1H, C**6**-<u>H</u>), 7.89 (d, *J* = 7.9 Hz, 1H, C**9**-<u>H</u>), 7.50 (dd, *J* = 8.1, 7.7 Hz, 1H, C**7**-<u>H</u>), 7.40 (dd, *J* = 7.9, 7.7 Hz, 1H, C**8**-<u>H</u>), 7.34 (dd, *J* = 7.5, 7.5 Hz, 2H, Ph ArC<u>H</u>), 7.28 – 7.23 (m, 3H, Ph ArC<u>H</u>), 3.21 (dd, *J* = 14.5, 6.1 Hz, 1H, C**3**-<u>H</u>), 3.01 (dd, *J* = 14.5, 8.2 Hz, 1H, C**3**-<u>H’</u>), 2.85 (dd, *J* = 13.5, 6.0 Hz, 1H, C**11**-<u>H</u>), 2.61 (dd, *J* = 13.5, 8.2 Hz, 1H, C**11**-<u>H’</u>), 2.53 – 2.44 (m, 1H, C**2**-<u>H</u>), 1.04 (d, *J* = 6.5 Hz, 3H, C**1**-<u>H$_3$</u>); ^{13}C NMR (125 MHz, $CDCl_3$) δ_C = 171.1 (C**4**), 153.4 (C**5**), 140.3 (Ph Ar<u>C</u>), 135.4 (C**10**), 129.4 (Ph Ar<u>C</u>H), 128.4 (Ph Ar<u>C</u>H), 126.2 (Ph Ar<u>C</u>H), 126.0 (C**7**), 124.8 (C**8**), 122.7 (C**6**), 121.6 (C**9**), 43.2 (C**11**), 41.3 (C**3**), 36.4 (C**2**), 19.4 (C**1**). HRMS (ESI$^+$) calculated for $C_{17}H_{18}NS$ [M+H]$^+$ = 268.1154, found 268.1156.

SFC conditions: CHIRALPACK SC (25 cm), 97:3 CO_2:*i*-PrOH, 1.5 mL/min, 254 nm; *Retention times*: t_{minor} = 16.1 min, t_{major} = 17.6 min.

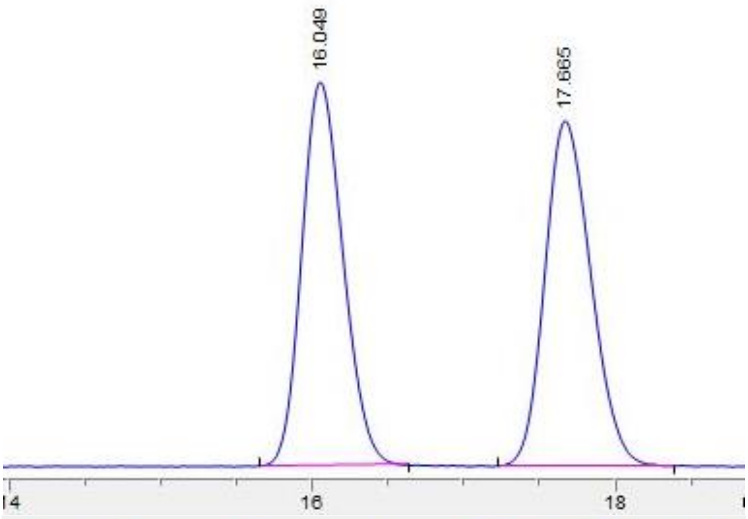

#	Time	Area	Height	Width	Area%	Symmetry
1	16.049	2824.1	149	0.2952	49.904	0.786
2	17.665	2835	134	0.3268	50.096	0.763

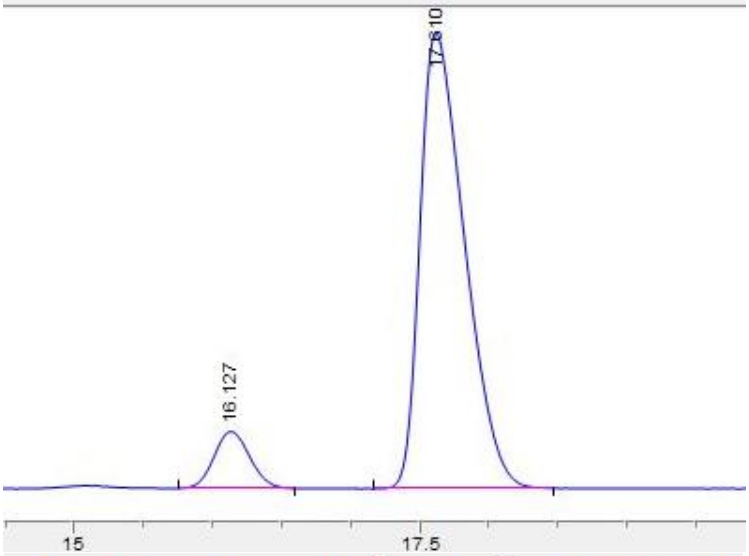

#	Time	Area	Height	Width	Area%	Symmetry
1	16.127	1024.5	56.8	0.2705	9.166	0.862
2	17.61	10152.4	454.1	0.3448	90.834	0.522

(*R*)-2-(2-Methyl-4-phenylbutyl)benzo[*d*]thiazole (4go)

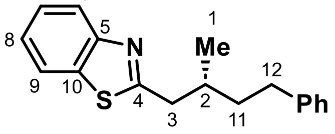

General procedure E: *tert*-Butyl 2-(benzo[*d*]thiazol-2-yl)acetate (24.9 mg, 0.10 mmol) and but-3-en-1-ylbenzene (19.8 mg, 22.5 μL, 0.15 mmol) were employed with [Ir(cod)$_2$]BARF (6.36 mg, 0.005 mmol) and **L6** (1.91 mg, 0.005 mmol) in *m*-xylene (0.20 mL). The reaction was stirred at 70 °C for 60 h. Then *p*-toluenesulfonic acid monohydrate (5.71 mg, 0.03 mmol) was added and the resulting reaction mixture was heated at 130 °C for 3 h. Purification by flash column chromatography (Hexane/EtOAc = 10/1 to 5/1) afforded the title compound (27.3 mg, 97%, B:L > 25:1, 91:9 e.r.) as a colorless oil. $[\alpha]_D^{23}$ = +31.2

(c 0.5, CH_2Cl_2). IR (thin film) ν_{max}/cm^{-1}: 2917, 2854, 1436, 1244, 1120, 759. ^{1}H NMR (500 MHz, $CDCl_3$) δ_H = 7.90 (d, J = 8.2 Hz, 1H, C**6**-H), 7.75 (dd, J = 8.0, 1.2 Hz, 1H, C**9**-H), 7.36 (ddd, J = 8.2, 7.4, 1.2 Hz, 1H, C**7**-H), 7.26 (ddd, J = 8.0, 7.4, 1.2 Hz, 1H, C**8**-H), 7.21 – 7.16 (m, 2H, Ph ArCH), 7.13 – 7.05 (m, 3H, Ph ArCH), 3.08 (dd, J = 14.5, 6.2 Hz, 1H, C**3**-H), 2.89 (dd, J = 14.5, 8.1 Hz, 1H, C**3**-H'), 2.67 (ddd, J = 13.7, 10.5, 5.5 Hz, 1H, C**12**-H), 2.56 (ddd, J = 13.7, 10.5, 6.0 Hz, 1H, C**12**-H'), 2.11 – 2.01 (m, 1H, C**2**-H), 1.74 – 1.68 (m, 1H, C**11**-H), 1.57 – 1.49 (m, 1H, C**11**-H'), 1.00 (d, J = 6.6 Hz, 3H, C**1**-H_3); ^{13}C NMR (125 MHz, $CDCl_3$) δ_C = 171.1 (C**4**), 153.4 (C**5**), 142.4 (Ph ArC), 135.4 (C**10**), 128.5 (Ph ArCH), 128.5 (Ph ArCH), 126.0 (C**7**), 125.9 (Ph ArCH), 124.8 (C**8**), 122.7 (C**6**), 121.6 (C**9**), 41.7 (C**3**), 38.6 (C**11**), 34.2 (C**2**), 33.4 (C**12**), 19.6 (C**1**). HRMS (ESI$^+$) calculated for $C_{18}H_{20}NS$ $[M+H]^+$ = 282.1311, found 282.1311.

SFC conditions: CHIRALPACK SC (25 cm), 97:3 CO_2:*i*-PrOH, 1.5 mL/min, 254 nm; *Retention times*: t_{minor} = 21.4 min, t_{major} = 29.4 min.

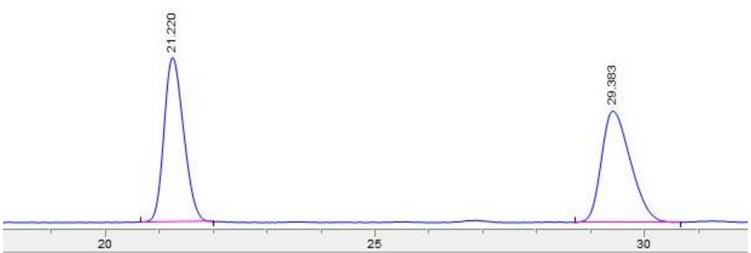

#	Time	Area	Height	Width	Area%	Symmetry
1	21.22	2969	117.2	0.3916	49.894	0.752
2	29.383	2981.6	79.5	0.5557	50.106	0.635

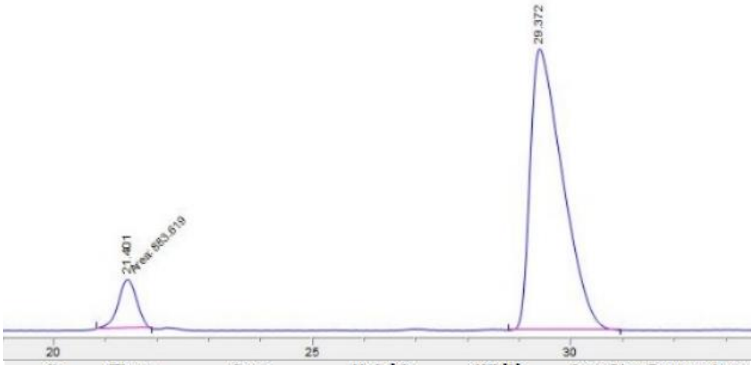

#	Time	Area	Height	Width	Area%	Symmetry
1	21.401	883.6	35.2	0.4183	9.055	1.002
2	29.372	8875.2	204	0.6355	90.945	0.394

(*R*)-6-(Benzo[*d*]thiazol-2-yl)-5-methylhexyl benzoate (4gp)

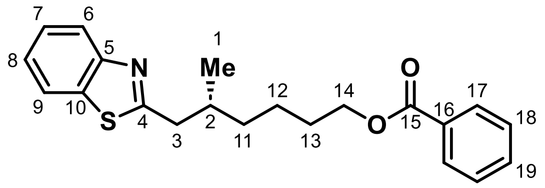

General procedure E: *tert*-Butyl 2-(benzo[*d*]thiazol-2-yl)acetate (24.9 mg, 0.10 mmol) and hex-5-en-1-yl benzoate (30.6 mg, 0.15 mmol) were employed with [Ir(cod)$_2$]BARF (6.36 mg, 0.005 mmol) and **L6** (1.91 mg, 0.005 mmol) in *m*-xylene (0.20 mL). The reaction was stirred at 70 °C for 96 h. Then *p*-toluenesulfonic acid monohydrate (5.71 mg, 0.03 mmol) was added and the resulting reaction mixture was heated at 130 °C for 3 h. Purification by flash column chromatography (Hexane/EtOAc = 10/1 to 5/1) afforded the title compound (27.9 mg, 79%, B:L > 25:1, 93.5:6.5 e.r.) as a colorless oil. $[\alpha]_D^{23}$ = +7.34 (*c* 0.5, CH_2Cl_2). IR (thin film) ν_{max}/cm^{-1}: 2937, 2873, 1448, 1274, 1113, 760. ^{1}H NMR (500 MHz, $CDCl_3$) δ_H = 8.11 – 8.00 (m, 2H, C**17**-H), 8.00 – 7.95 (m, 1H, C**6**-H), 7.86 – 7.79 (m, 1H, C**9**-H), 7.58 – 7.52 (m, 1H, C**19**-H), 7.47 – 7.39 (m, 3H, C**7**-H + 2 × C**18**-H), 7.34 (ddd, *J* = 8.2, 7.6, 1.2 Hz, 1H, C**8**-H), 4.32 (t, *J* = 6.7 Hz, 2H, C**14**-H_2), 3.11 (dd, *J* = 14.4, 6.3 Hz, 1H, C**3**-H), 2.94 (dd, *J* = 14.4, 8.0 Hz, 1H, C**3**-H'), 2.17 – 2.07 (m, 1H, C**2**-H), 1.78 (ddt, *J* = 15.2, 12.5, 6.7 Hz, 2H, C**13**-H_2), 1.61 – 1.46 (m, 3H, C**11**-H + C**12**-H + C**12**-H'), 1.40 – 1.32 (m, 1H, C**11**-H'), 1.02 (d, *J* = 6.6 Hz, 3H, C**1**-H_3); ^{13}C NMR (125 MHz, $CDCl_3$) δ_C = 171.3 (C**4**), 166.8 (C**15**), 153.3 (C**5**), 135.3 (C**10**), 132.9 (C**19**), 130.6 (C**16**), 129.7 (C**17**), 128.5 (C**18**), 126.0 (C**7**), 124.8 (C**8**), 122.7 (C**6**), 121.6 (C**9**), 65.0 (C**14**), 41.7 (C**3**), 36.3 (C**11**), 34.4 (C**2**), 29.0 (C**13**), 23.6 (C**12**), 19.6 (C**1**). HRMS (ESI$^+$) calculated for $C_{21}H_{24}NO_2S$ $[M+H]^+$ = 354.1522, found 354.1528.

SFC conditions: CHIRALPACK SC (25 cm), 85:15 CO_2:*i*-PrOH, 2.0 mL/min, 254 nm; *Retention times*: t_{major} = 11.8 min, t_{minor} = 12.9 min.

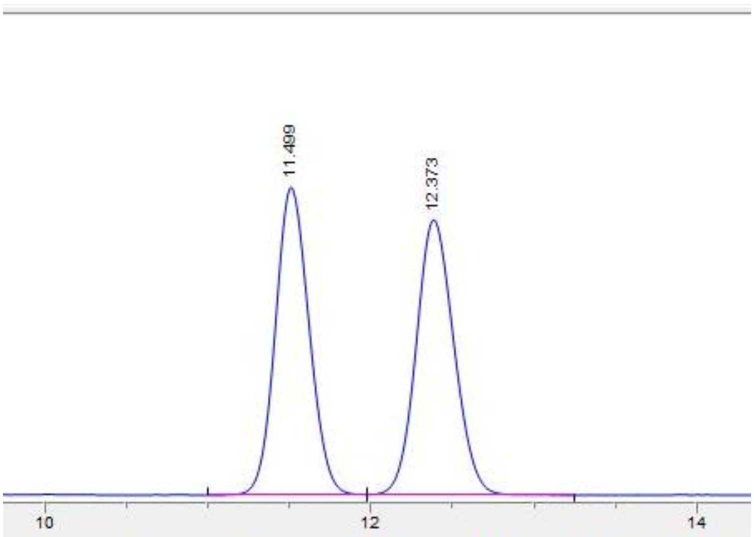

#	Time	Area	Height	Width	Area%	Symmetry
1	11.499	2768.3	192.9	0.2234	49.983	0.856
2	12.373	2770.1	172.5	0.2493	50.017	0.839

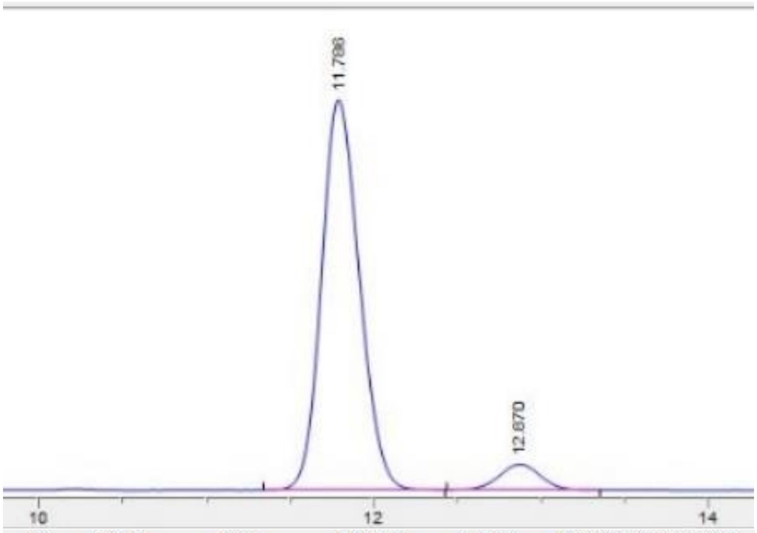

#	Time	Area	Height	Width	Area%	Symmetry
1	11.786	3834.2	244.5	0.2429	93.327	0.787
2	12.87	274.2	16.1	0.267	6.673	0.986

Ethyl (3*S*)-2-(benzo[*d*]thiazol-2-yl)-3-phenylbutanoate (3ga')

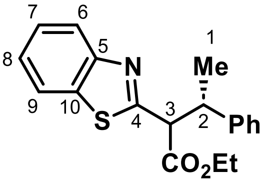

General procedure E: Ethyl 2-(benzo[*d*]thiazol-2-yl)acetate (22.1 mg, 0.10 mmol) and styrene (31.2 mg, 34.4 μL, 0.30 mmol) were employed with [Ir(cod)$_2$]BARF (6.36 mg, 0.005 mmol) and **L6** (1.91 mg, 0.005 mmol) in toluene (0.20 mL). The reaction was stirred at 100 °C for 120 h. Purification by flash column chromatography (Hexane/EtOAc = 15/1 to 9/1) afforded the title compounds (1.4:1 d.r., B:L > 25:1; diastereomer 1: 7.00 mg, 22%, 97:3 e.r.; diastereomer 2: 4.80 mg, 15%, 97:3 e.r.) as colorless oils.

Diastereomer 1: $[\alpha]_D^{24}$ = +8.65 (*c* 0.2, CH_2Cl_2). IR (thin film) ν_{max}/cm^{-1}: 2975, 2930, 1731, 1455, 1155, 760. ^{1}H NMR (500 MHz, CD_2Cl_2) δ_H = 8.04 (dd, *J* = 8.1, 1.1 Hz, 1H, C**6**-H), 7.94 (dd, *J* = 8.0, 1.1 Hz, 1H, C**9**-H), 7.50 (dd, *J* = 8.1, 8.0, 1.1 Hz, 1H, C**7**-H), 7.44 – 7.40 (m, 1H, C**8**-H), 7.35 – 7.32 (m, 4H, Ph ArCH), 7.28 – 7.22 (m, 1H, Ph ArCH), 4.39 (d, *J* = 11.1 Hz, 1H, C**3**-H), 3.92 – 3.81 (m, 2H, CH_2CH_3), 3.66 – 3.60 (m, 1H, C**2**-H), 1.22 (d, *J* = 6.9 Hz, 3H, C**1**-H_3), 0.93 (t, *J* = 7.1 Hz, 3H, CH_2CH_3); ^{13}C NMR (125 MHz, CD_2Cl_2) δ_C = 170.6 (C**4**), 167.5 (C=O), 153.1 (C**5**), 143.8 (Ph ArC), 136.1 (C**10**), 128.9 (Ph ArCH), 127.9 (Ph ArCH), 127.4 (Ph ArCH), 126.4 (C**7**), 125.6 (C**8**), 123.5 (C**6**), 122.1 (C**9**), 61.6 (CH_2CH_3), 59.2 (C**3**), 45.0 (C**2**), 20.2 (C**1**), 13.9 (CH_2CH_3). HRMS (ESI$^+$) calculated for $C_{19}H_{20}NO_2S$ $[M+H]^+$ = 326.1209, found 326.1213.

SFC conditions: CHIRALPACK IE (25 cm), 90:10 CO_2:MeOH, 2.0 mL/min, 254 nm; *Retention times*: t_{minor} = 6.6 min, t_{major} = 7.4 min.

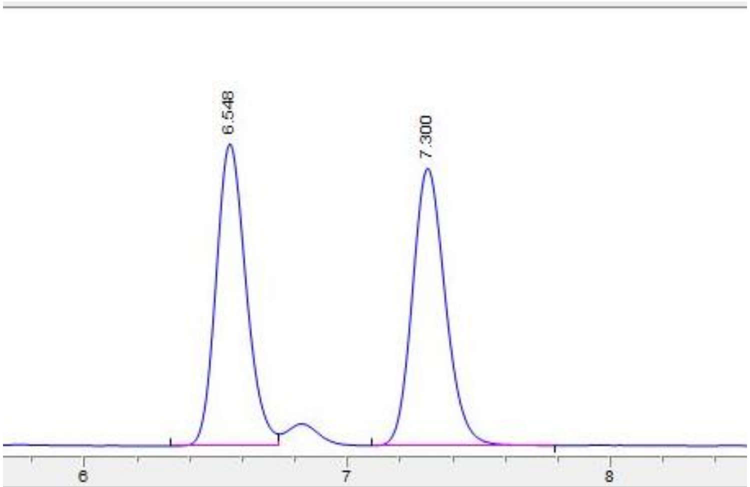

#	Time	Area	Height	Width	Area%	Symmetry
1	6.548	2212.4	278.7	0.1226	49.764	0.807
2	7.3	2233.3	256	0.1358	50.236	0.817

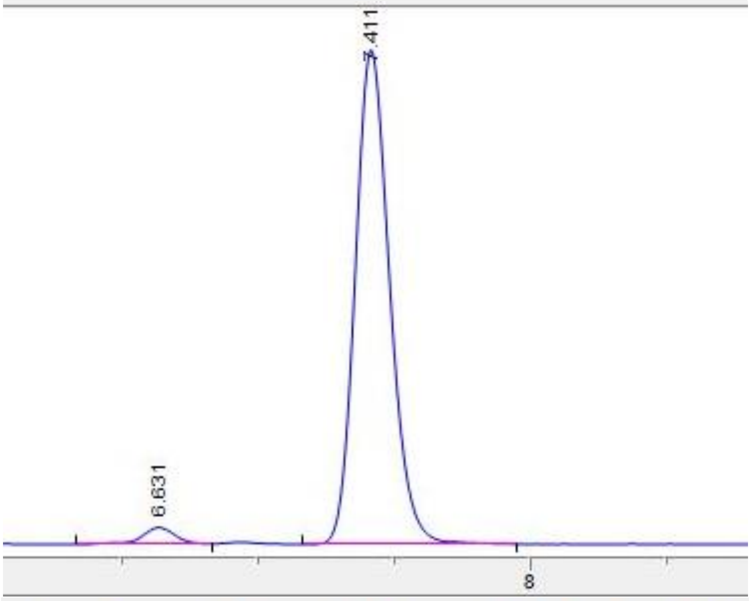

#	Time	Area	Height	Width	Area%	Symmetry
1	6.631	112.2	12.7	0.137	3.323	1.045
2	7.411	3264.6	371.5	0.1345	96.677	0.796

Diastereomer 2: $[\alpha]_D^{24}$ = +16.0 (*c* 0.2, CH_2Cl_2). IR (thin film) ν_{max}/cm^{-1}: 2977, 2935, 1731, 1435, 1156, 759. ^{1}H NMR (500 MHz, CD_2Cl_2) δ_H = 7.87 (dd, *J* = 8.3, 1.3 Hz, 1H, C**6**-H), 7.81 – 7.75 (dd, *J* = 8.2, 1.3 Hz, 1H, C**9**-H), 7.39 (ddd, *J* = 8.3, 7.1, 1.3 Hz, 1H, C**7**-H), 7.31 (ddd, *J* = 8.2, 7.1, 1.3 Hz, 1H, C**8**-H), 7.21 – 7.15 (m, 4H, Ph ArCH), 7.11 – 7.07 (m, 1H, Ph ArCH), 4.42 (d, *J* = 10.9 Hz, 1H, C**3**-H), 4.29 – 4.17 (m, 2H, CH_2CH_3), 3.69 (dt, *J* = 10.9, 6.9 Hz, 1H, C**2**-H), 1.44 (d, *J* = 6.9 Hz, 3H, C**1**-H_3), 1.28 (t, *J* = 7.1 Hz, 3H, CH_2CH_3); ^{13}C NMR (125 MHz, CD_2Cl_2) δ_C = 170.9 (C**4**), 167.3 (C=O), 152.8 (C**5**), 143.2 (Ph ArC), 135.8 (C**10**), 128.7 (Ph ArCH), 128.0 (Ph ArCH), 127.1 (Ph ArCH), 126.2 (C**7**), 125.4 (C**8**), 123.3 (C**6**), 121.9 (C**9**), 62.1 (CH_2CH_3), 58.5 (C**3**), 44.4 (C**2**), 21.1 (C**1**), 14.3 (CH_2CH_3). HRMS (ESI$^+$) calculated for $C_{19}H_{20}NO_2S$ $[M+H]^+$ = 326.1209, found 326.1208.

SFC conditions: CHIRALPACK IE (25 cm), 90:10 CO_2:MeOH, 2.0 mL/min, 254 nm; *Retention times*: t_{minor} = 6.2 min, t_{major} = 6.6 min.

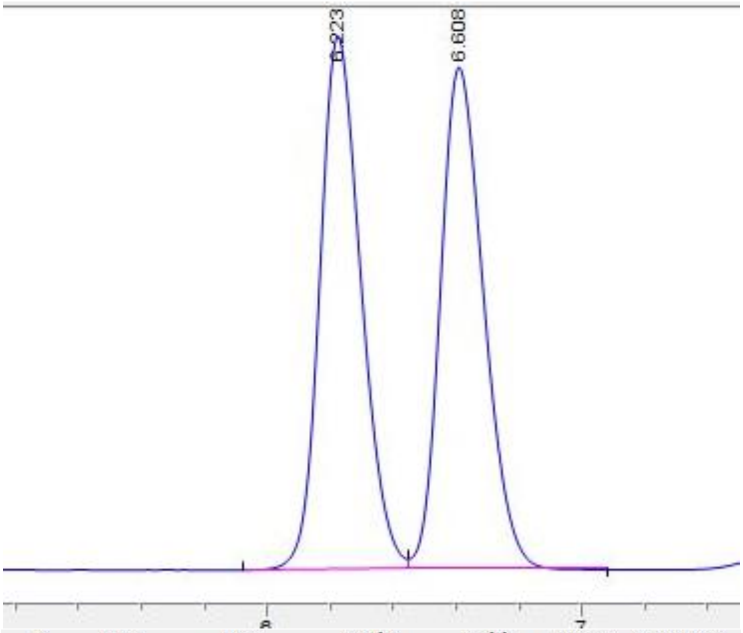

#	Time	Area	Height	Width	Area%	Symmetry
1	6.223	3513.9	385.5	0.1403	50.320	0.746
2	6.608	3469.2	363.1	0.1475	49.680	0.72

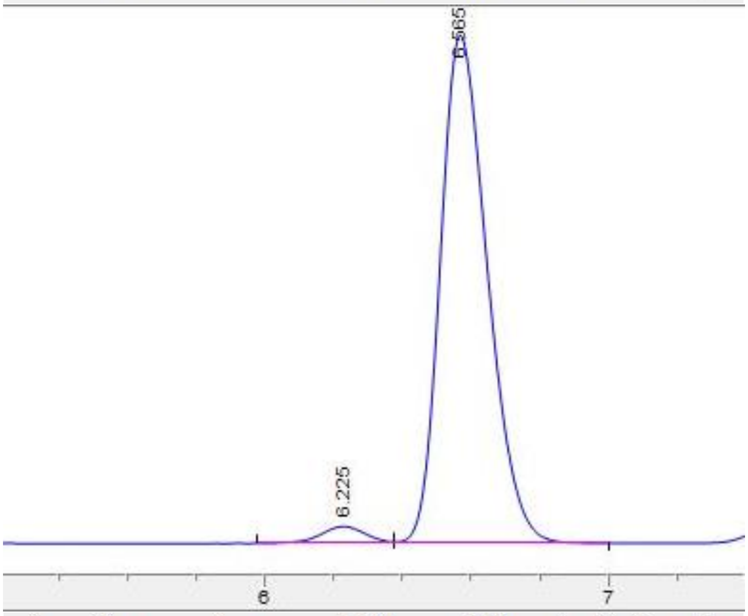

#	Time	Area	Height	Width	Area%	Symmetry
1	6.225	115.9	12.6	0.141	3.104	1.032
2	6.565	3617.1	380.4	0.1469	96.896	0.696

***tert*-Butyl (3*S*)-2-(benzo[*d*]thiazol-2-yl)-3-phenylbutanoate (3ga)**

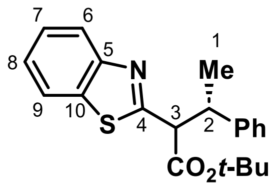

__General procedure E__: *tert*-Butyl 2-(benzo[*d*]thiazol-2-yl)acetate (24.9 mg, 0.10 mmol) and styrene (15.6 mg, 17.2 μL, 0.15 mmol) were employed with [Ir(cod)$_2$]BARF (6.36 mg, 0.005 mmol) and **L6** (1.91 mg, 0.005 mmol) in toluene (0.20 mL). The reaction was stirred at 70 °C for 48 h. Purification by

flash column chromatography (Hexane/EtOAc = 40/1 to 15/1) afforded the title compounds (3.5:1 d.r., B:L > 25:1; diastereomer 1: 26.5 mg, 75%, 99:1 e.r.; diastereomer 2: 8.00 mg, 23%, 98.5:1.5 e.r.) as colorless solids.

Diastereomer 1: m.p. 92 - 95 °C (EtOAc/hexane). $[\alpha]_D^{23}$ = +23.8 (*c* 0.5, CH_2Cl_2). IR (thin film) ν_{max}/cm^{-1}: 2976, 2931, 1726, 1456, 1148, 759. ^1H NMR (500 MHz, CD_2Cl_2) δ_H = 8.04 (dd, *J* = 8.2, 1.1 Hz, 1H, C**6**-H), 7.95 (dd, *J* = 8.0, 1.1 Hz, 1H, C**9**-H), 7.50 (ddd, *J* = 8.2, 7.2, 1.1 Hz, 1H, C**7**-H), 7.42 (ddd, *J* = 8.0, 7.2, 1.1 Hz, 1H, C**8**-H), 7.38 – 7.32 (m, 4H, Ph ArCH), 7.26 (tt, *J* = 5.5, 3.1 Hz, 1H, Ph ArCH), 4.32 (d, *J* = 11.3 Hz, 1H, C**3**-H), 3.54 (dq, *J* = 11.3, 7.0 Hz, 1H, C**2**-H), 1.19 (d, *J* = 7.0 Hz, 3H, C**1**-H_3), 1.14 (s, 9H, $C(CH_3)_3$); ^{13}C NMR (125 MHz, CD_2Cl_2) δ_C = 169.6 (C**4**), 168.2 (C=O), 153.1 (C**5**), 144.0 (Ph ArC), 136.1 (C**10**), 128.8 (Ph ArCH), 128.2 (Ph ArCH), 127.3 (Ph ArCH), 126.3 (C**7**), 125.5 (C**8**), 123.4 (C**6**), 122.1 (C**9**), 82.2 ($C(CH_3)_3$), 60.2 (C**3**), 45.5 (C**2**), 27.6 ($C(CH_3)_3$), 20.6 (C**1**). HRMS (ESI^+) calculated for $C_{21}H_{23}NNaO_2S$ $[M+Na]^+$ = 376.1342, found 376.1346.

SFC conditions: CHIRALPACK SC (25 cm), 95:5 CO_2:*i*-PrOH, 2.0 mL/min, 254 nm; *Retention times*: t_{minor} = 4.4 min, t_{major} = 6.2 min.

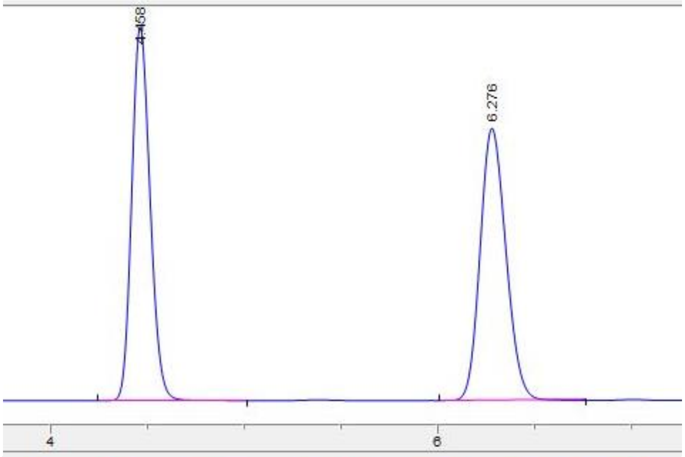

#	Time	Area	Height	Width	Area%	Symmetry
1	4.458	3521.7	535.9	0.1027	49.340	0.8
2	6.276	3615.9	389.6	0.1443	50.660	0.803

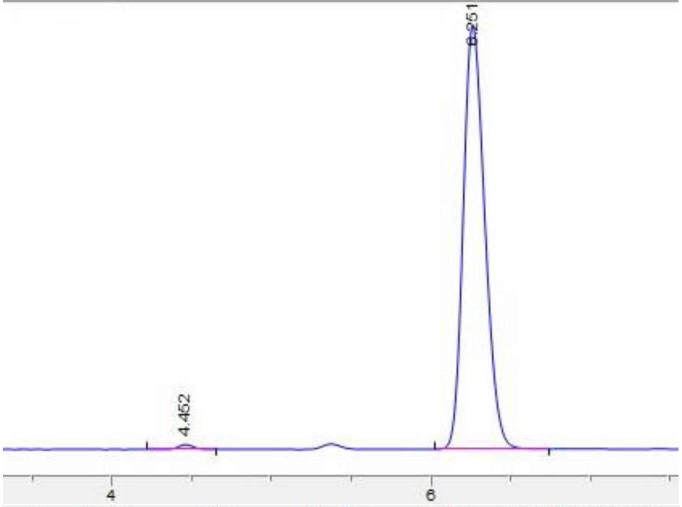

#	Time	Area	Height	Width	Area%	Symmetry
1	4.452	41.2	6.3	0.1029	0.784	0.878
2	6.251	5215.5	557.8	0.1451	99.216	0.741

Diastereomer 2: m.p. 91 - 93 °C (EtOAc/hexane). $[\alpha]_D^{23}$ = +84.7 (c 0.5, CH_2Cl_2). IR (thin film) ν_{max}/cm^{-1}: 2970, 2931, 1727, 1454, 1148, 759. ^{1}H NMR (500 MHz, CD_2Cl_2) δ_H = 7.84 (d, J = 8.2 Hz, 1H, C**6**-H), 7.79 (d, J = 8.0 Hz, 1H, C**9**-H), 7.38 (ddd, J = 8.2, 7.2, 1.3 Hz, 1H, C**7**-H), 7.30 (ddd, J = 8.0, 7.2, 1.2 Hz, 1H, C**8**-H), 7.19 – 7.13 (m, 4H, Ph ArCH), 7.11 – 7.05 (m, 1H, Ph ArCH), 4.30 (d, J = 11.0 Hz, 1H, C**3**-H), 3.63 (dq, J = 11.0, 6.8 Hz, 1H, C**2**-H), 1.48 (s, 9H, C(CH_3)$_3$), 1.44 (d, J = 6.8 Hz, 3H, C**1**-H_3); ^{13}C NMR (125 MHz, CD_2Cl_2) δ_C = 170.0 (C**4**), 167.9 (C=O), 152.8 (C**5**), 143.4 (Ph ArC), 135.8 (C**10**), 128.6 (Ph ArCH), 128.0 (Ph ArCH), 127.0 (Ph ArCH), 126.1 (C**7**), 125.2 (C**8**), 123.2 (C**6**), 121.8 (C**9**), 82.7 (C(CH_3)$_3$), 59.7 (C**3**), 44.6 (C**2**), 28.0 (C(CH_3)$_3$), 21.1 (C**1**). HRMS (ESI$^+$) calculated for $C_{21}H_{23}NNaO_2S$ $[M+Na]^+$ = 376.1342, found 376.1346.

SFC conditions: CHIRALPACK SC (25 cm), 95:5 CO_2:i-PrOH, 2.0 mL/min, 254 nm; *Retention times*: t_{minor} = 11.5 min, t_{major} = 18.3 min.

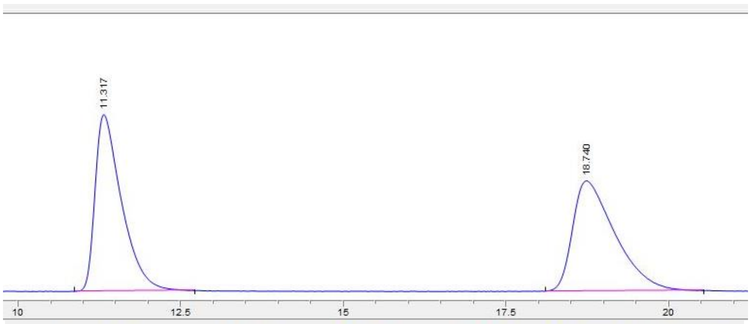

#	Time	Area	Height	Width	Area%	Symmetry
1	11.317	2729.6	96	0.4138	50.027	0.46
2	18.74	2726.7	59.9	0.6408	49.973	0.462

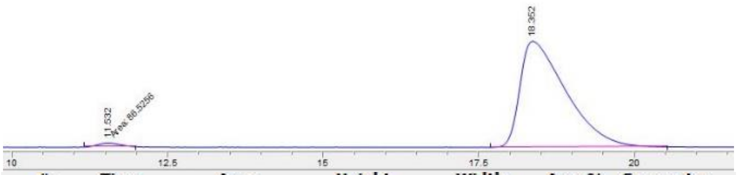

#	Time	Area	Height	Width	Area%	Symmetry
1	11.532	86.5	3.8	0.3753	1.408	0.926
2	18.352	6060.3	117.7	0.7109	98.592	0.365

***tert*-Pentyl 2-(benzo[*d*]thiazol-2-yl)acetate (1g'')**

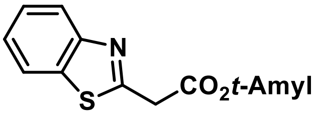

General procedure C: 2-Chlorobenzothiazole (339 mg, 2.00 mmol) was employed with *tert*-pentyl acetate (781 mg, 6.00 mmol) and NaHMDS (0.6 M in toluene, 10.0 mL, 6.00 mmol). Purification by flash column chromatography (Hexane/EtOAc = 20/1 to 9/1) afforded the title compound (510 mg, 97%) as a yellow oil. ^{1}H NMR (500 MHz, $CDCl_3$) δ_H = 8.00 (dd, J = 8.2, 1.0 Hz, 1H), 7.87 (dd, J = 8.0,

1.2 Hz, 1H), 7.46 (dd, J = 8.2, 7.4, 1.2 Hz, 1H), 7.38 (ddd, J = 8.0, 7.4, 1.0 Hz, 1H), 4.10 (s, 2H), 1.79 (q, J = 7.5 Hz, 2H), 1.46 (s, 6H), 0.87 (t, J = 7.5 Hz, 3H); ^{13}C NMR (125 MHz, $CDCl_3$) δ_C = 167.6, 163.5, 152.9, 136.0, 126.1, 125.2, 123.1, 121.6, 85.2, 41.3, 33.6, 25.6, 8.3. HRMS (ESI$^+$) calculated for $C_{14}H_{17}NNaO_2S$ [M+Na]$^+$ = 286.0872, found 286.0866.

***tert*-Pentyl (3*S*)-2-(benzo[*d*]thiazol-2-yl)-3-phenylbutanoate (3ga'')**

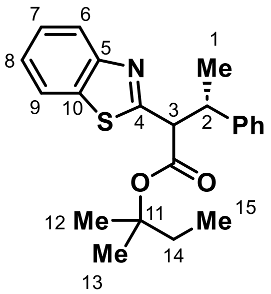

<u>General procedure E</u>: The preceding azaarylacetate (26.3 mg, 0.10 mmol) and styrene (31.2 mg, 34.4 μL, 0.30 mmol) were employed with [Ir(cod)$_2$]BARF (6.36 mg, 0.005 mmol) and **L6** (1.91 mg, 0.005 mmol) in toluene (0.20 mL). The reaction was stirred at 100 °C for 96 h. Purification by flash column chromatography (Hexane/EtOAc = 40/1 to 10/1) afforded the title compounds (1.7:1 d.r., B:L > 25:1; diastereomer 1: 15.2 mg, 41%, 98.5:1.5 e.r.; diastereomer 2: 8.50 mg, 23%, 98.5:1.5 e.r.) as colorless oils.

Diastereomer 1: $[\alpha]_D^{24}$ = +20.6 (c 0.5, CH_2Cl_2). IR (thin film) ν_{max}/cm^{-1}: 2974, 2934, 1725, 1455, 1146, 759. ^{1}H NMR (500 MHz, CD_2Cl_2) δ_H = 8.03 (dd, J = 8.2, 1.1 Hz, 1H, C**6**-<u>H</u>), 7.94 (dd, J = 8.0, 1.1 Hz, 1H, C**9**-<u>H</u>), 7.50 (ddd, J = 8.2, 7.2, 1.1 Hz, 1H, C**7**-<u>H</u>), 7.42 (ddd, J = 8.0, 7.2, 1.1 Hz 1H, C**8**-<u>H</u>), 7.37 – 7.32 (m, 4H, Ph ArC<u>H</u>), 7.25 (tt, J = 5.6, 2.7 Hz, 1H, Ph ArC<u>H</u>), 4.33 (d, J = 11.4 Hz, 1H, C**3**-<u>H</u>), 3.55 (dq, J = 11.4, 7.0 Hz, 1H, C**2**-<u>H</u>), 1.59 – 1.46 (m, 2H, C**14**-<u>H$_2$</u>), 1.18 (d, J = 7.0 Hz, 3H, C**1**-<u>H$_3$</u>), 1.11 (s, 3H, C**12**-<u>H$_3$</u>), 1.05 (s, 3H, C**13**-<u>H$_3$</u>), 0.64 (t, J = 7.5 Hz, 3H, C**15**-<u>H$_3$</u>); ^{13}C NMR (125 MHz, CD_2Cl_2) δ_C = 169.6 (C**4**), 168.2 (<u>C</u>=O), 153.1 (C**5**), 144.1 (Ph Ar<u>C</u>), 136.1 (C**10**), 128.8 (Ph Ar<u>C</u>H), 128.1 (Ph Ar<u>C</u>H), 127.3 (Ph Ar<u>C</u>H), 126.3 (C**7**), 125.5 (C**8**), 123.4 (C**6**), 122.1 (C**9**), 84.8 (C**11**), 60.2 (C**3**), 45.2 (C**2**), 33.9 (C**14**), 25.1 (C**12**), 24.7 (C**13**), 20.7 (C**1**), 8.1 (C**15**); HRMS (ESI$^+$) calculated for $C_{22}H_{25}NNaO_2S$ [M+Na]$^+$ = 390.1498, found 390.1503.

SFC conditions: CHIRALPACK IE (25 cm), 90:10 CO_2:MeOH, 2.0 mL/min, 254 nm; *Retention times*: t_{minor} = 5.4 min, t_{major} = 7.1 min.

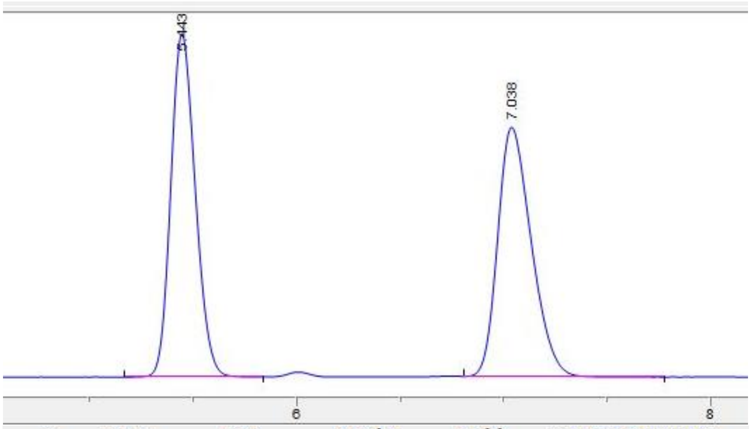

#	Time	Area	Height	Width	Area%	Symmetry
1	5.443	4632.8	562.6	0.1281	50.028	0.791
2	7.038	4627.6	409.1	0.174	49.972	0.677

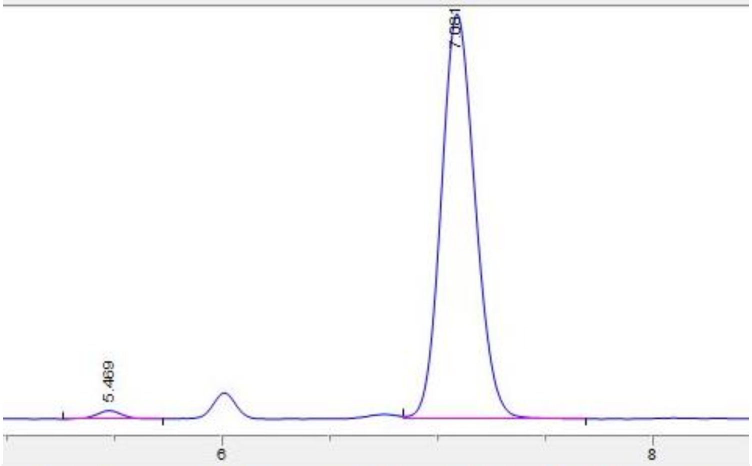

#	Time	Area	Height	Width	Area%	Symmetry
1	5.469	20.4	2.5	0.1252	1.549	0.969
2	7.081	1299	119	0.1713	98.451	0.837

Diastereomer 2: $[\alpha]_D^{24}$ = +89.0 (*c* 0.2, CH_2Cl_2). IR (thin film) ν_{max}/cm^{-1}: 2970, 2919, 1728, 1454, 1146, 759. ^{1}H NMR (500 MHz, CD_2Cl_2) δ_H = 7.84 (d, *J* = 8.1 Hz, 1H, C**6**-$\underline{H}$), 7.78 (d, *J* = 8.0 Hz, 1H, C**9**-$\underline{H}$), 7.38 (dd, *J* = 8.1, 7.6 Hz, 1H, C**7**-$\underline{H}$), 7.30 (dd, *J* = 8.0, 7.6 Hz, 1H, C**8**-$\underline{H}$), 7.20 – 7.13 (m, 4H, Ph ArC$\underline{H}$), 7.10 – 7.04 (m, 1H, Ph ArC$\underline{H}$), 4.32 (d, *J* = 11.1 Hz, 1H, C**3**-$\underline{H}$), 3.63 (dq, *J* = 11.1, 6.8 Hz, 1H, C**2**-$\underline{H}$), 1.83 – 1.76 (m, 2H, C**14**-$\underline{H_2}$), 1.45 (d, *J* = 6.8 Hz, C**1**-$\underline{H_3}$), 1.44 (s, 6H, C**12**-$\underline{H_3}$ + C**13**-$\underline{H_3}$), 0.84 (t, *J* = 7.5 Hz, 3H, C**15**-$\underline{H_3}$); ^{13}C NMR (125 MHz, CD_2Cl_2) δ_C = 169.9 (C**4**), 167.9 ($\underline{C}$=O), 152.8 (C**5**), 143.4 (Ph Ar$\underline{C}$), 135.8 (C**10**), 128.6 (Ph Ar$\underline{C}$H), 128.0 (Ph Ar$\underline{C}$H), 127.0 (Ph Ar$\underline{C}$H), 126.1 (C**7**), 125.2 (C**8**), 123.2 (C**6**), 121.8 (C**9**), 85.3 (C**11**), 59.7 (C**3**), 44.4 (C**2**), 33.9 (C**14**), 25.4 (C**12**), 25.4 (C**13**), 21.2 (C**1**), 8.3 (C**15**). HRMS (ESI$^+$) calculated for $C_{22}H_{25}NNaO_2S$ $[M+Na]^+$ = 390.1498, found 390.1494.

SFC conditions: CHIRALPACK IE (25 cm), 90:10 CO_2:MeOH, 2.0 mL/min, 254 nm; *Retention times*: t_{minor} = 5.3 min, t_{major} = 6.7 min.

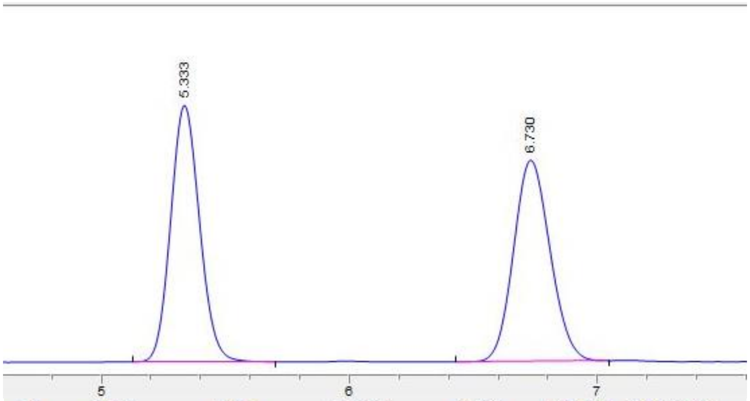

#	Time	Area	Height	Width	Area%	Symmetry
1	5.333	712.8	87.8	0.1267	50.300	0.87
2	6.73	704.3	68.8	0.1594	49.700	0.853

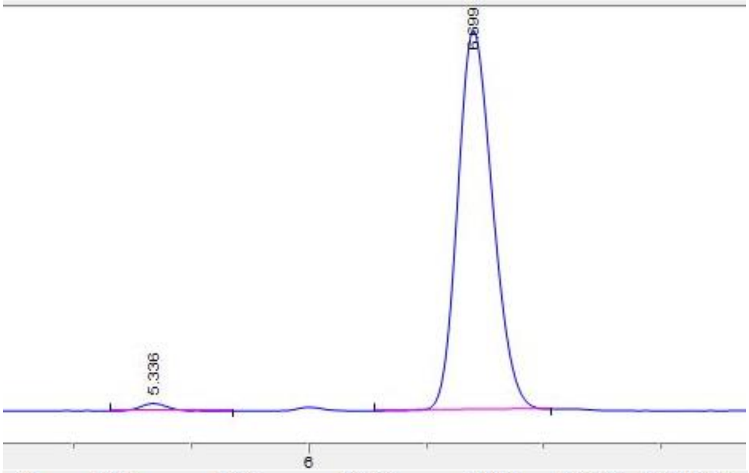

#	Time	Area	Height	Width	Area%	Symmetry
1	5.336	24	2.8	0.128	1.620	0.877
2	6.699	1458.8	140.6	0.163	98.380	0.778

(3*S*)-2-(Benzo[*d*]thiazol-2-yl)-1-morpholino-3-phenylbutan-1-one (3ga’’’)

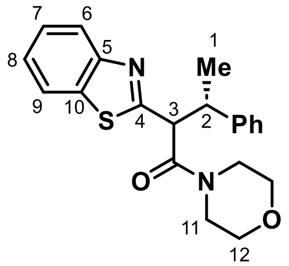

<u>**General procedure E**</u>: 2-(Benzo[*d*]thiazol-2-yl)-1-morpholinoethan-1-one (26.2 mg, 0.10 mmol) and styrene (31.2 mg, 34.5 μL, 0.30 mmol) were employed with [Ir(cod)$_2$]BARF (12.7 mg, 0.01 mmol) and **L2** (6.10 mg, 0.01 mmol) in toluene (0.20 mL). The reaction was stirred at 120 °C for 48 h. Purification by flash column chromatography (Hexane/EtOAc = 5/1 to 1/1) afforded the title compound (2.2:1 d.r., B:L = 4.7:1, diastereomer 1: 18.2 mg, 50%, 91:9 e.r.; diastereomer 2: 8.30 mg, 22%, 60:40 e.r.) as yellow solids.

Diastereomer 1: m.p. 125 – 126 °C (EtOAc/hexane). $[\alpha]_D^{25}$ = -18.7 (*c* 0.2, CH_2Cl_2). IR (thin film) ν_{max}/cm^{-1}: 2962, 2923, 2855, 1638, 1500, 1436, 1245, 1114, 848. ^{1}H NMR (500 MHz, $CDCl_3$) δ_H =

8.01 (d, J = 8.1 Hz, 1H, C**6**-H), 7.91 (d, J = 8.0 Hz, 1H, C**9**-H), 7.51 – 7.46 (m, 1H, C**7**-H), 7.42 – 7.37 (m, 1H, C**8**-H), 7.37 – 7.32 (m, 4H, Ph ArCH), 7.30 – 7.24 (m, 1H, Ph ArCH), 4.65 (d, J = 10.9 Hz, 1H, C**3**-H), 3.72 – 3.64 (m, 1H, C**2**-H), 3.53 – 3.43 (m, 2H, C**11**-H + C**12**-H), 3.41 – 3.31 (m, 2H, C**11'**-H + C**12'**-H), 3.30 – 3.24 (m, 1H, C**11**-H'), 3.21 – 3.14 (m, 2H, C**11'**-H' + C**12'**-H'), 2.84 – 2.77 (m, 1H, C**12**-H'), 1.21 (d, J = 7.0 Hz, 3H, C**1**-H_3); ^{13}C NMR (125 MHz, $CDCl_3$) δ_C = 169.3 (C=O), 169.2 (C**4**), 152.0 (C**5**), 143.6 (Ph ArC), 136.2 (C**10**), 128.8 (Ph ArCH), 127.7 (Ph ArCH), 127.4 (Ph ArCH), 126.1 (C**7**), 125.3 (C**8**), 123.0 (C**6**), 121.9 (C**9**), 66.6 (C**12**), 66.3 (C**12'**), 55.2 (C**3**), 46.5 (C**11**), 45.7 (C**2**), 42.5 (C**11'**), 19.1 (C**1**). HRMS (ESI$^+$) calculated for $C_{21}H_{22}N_2O_2SNa$ $[M+Na]^+$ = 389.1294, found 389.1298.

SFC conditions: CHIRALPACK SB (25 cm), 85:15 CO_2:*i*-PrOH, 2.0 mL/min, 254 nm; *Retention times*: t_{minor} = 5.1 min, t_{major} = 6.0 min.

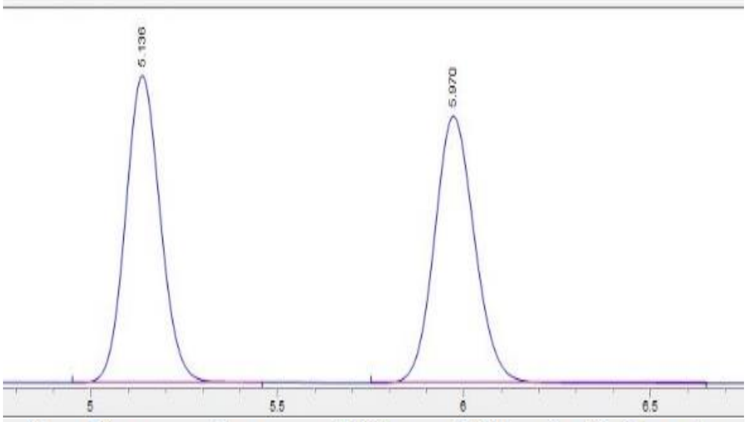

#	Time	Area	Height	Width	Area%	Symmetry
1	5.136	3239.8	498.4	0.0998	49.987	0.885
2	5.97	3241.6	433.2	0.1173	50.013	0.857

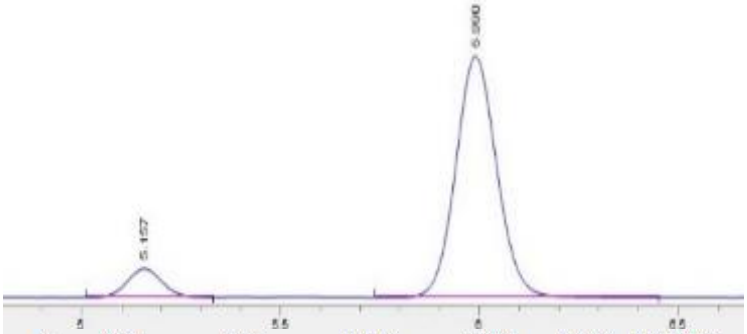

#	Time	Area	Height	Width	Area%	Symmetry
1	5.157	155.3	25.6	0.0947	9.071	0.935
2	5.99	1556.4	216.6	0.1118	90.929	0.891

Diastereomer 2: m.p. 148.6 – 149.7 °C (EtOAc/hexane). $[\alpha]_D^{25}$ = -13.9 (c 0.1, CH_2Cl_2). IR (thin film) ν_{max}/cm^{-1}: 2962, 2923, 2855, 1638, 1500, 1436, 1245, 1114, 848. ^{1}H NMR (500 MHz, $CDCl_3$) δ_H = 7.82 (d, J = 8.1 Hz, 1H, C**6**-H), 7.77 (d, J = 8.0 Hz, 1H, C**9**-H), 7.39 – 7.34 (m, 1H, C**7**-H), 7.32 – 7.27

(m, 1H, C**8**-H), 7.23 – 7.19 (m, 2H, Ph ArCH), 7.19 – 7.14 (m, 2H, Ph ArCH), 7.10 – 7.05 (m, 1H, Ph ArCH), 4.75 (d, J = 10.1 Hz, 1H, C**3**-H), 3.84 – 3.75 (m, 3H, C**2**-H + C**11**-H_2), 3.70 – 3.64 (m, 1H, C**12**-H), 3.64 – 3.51 (m, 4H, C**11'**-H_2 + C**12'**-H_2), 3.40 – 3.34 (m, 1H, C**12**-H'), 1.42 (d, J = 6.7 Hz, 3H, C**1**-H_3); ^{13}C NMR (125 MHz, $CDCl_3$) δ_C = 168.8 (C=O), 168.7 (C**4**), 152.1 (C**5**), 142.9 (Ph ArC), 135.6 (C**10**), 128.6 (Ph ArCH), 127.9 (Ph ArCH), 126.9 (Ph ArCH), 125.9 (C**7**), 125.1 (C**8**), 122.8 (C**6**), 121.7 (C**9**), 66.9 (C**12**), 66.7 (C**12'**), 54.1 (C**3**), 46.8 (C**11**), 44.8 (C**2**), 43.0 (C**11'**), 21.0 (C**1**). HRMS (ESI$^+$) calculated for $C_{21}H_{22}N_2O_2SNa$ [M+Na]$^+$ = 389.1294, found 389.1298.

SFC conditions: CHIRALPACK SB (25 cm), 92:8 CO_2:*i*-PrOH, 2.0 mL/min, 254 nm; *Retention times*: t_{minor} = 11.1 min, t_{major} = 11.6 min.

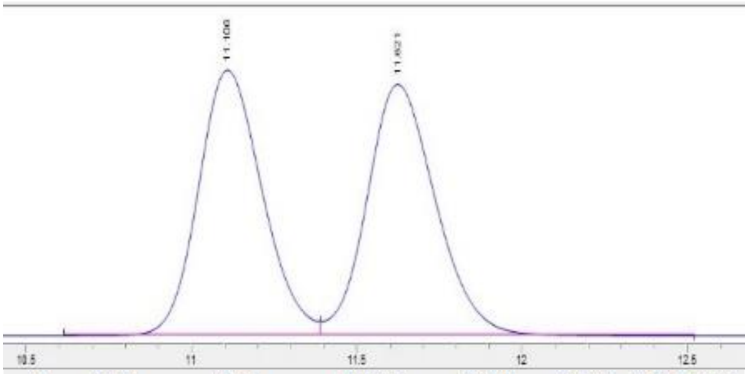

#	Time	Area	Height	Width	Area%	Symmetry
1	11.106	3864.2	286.8	0.2072	49.582	0.812
2	11.621	3929.3	271.5	0.2229	50.418	0.779

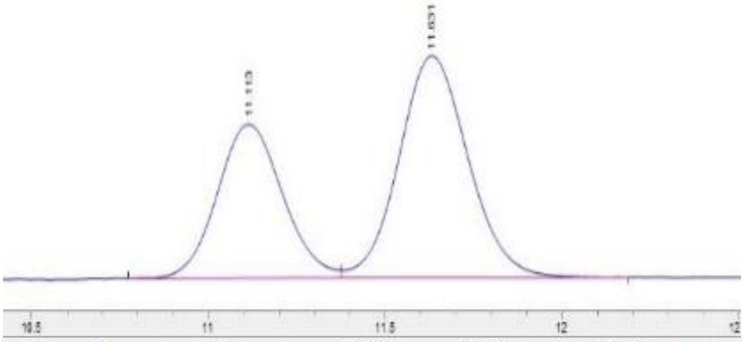

#	Time	Area	Height	Width	Area%	Symmetry
1	11.113	609.1	47.1	0.1989	39.715	0.893
2	11.631	924.6	67.8	0.2112	60.285	0.87

Deamidation of 3ga''' to (*S*)-2-(2-phenylpropyl)benzo[*d*]thiazole (4ga)

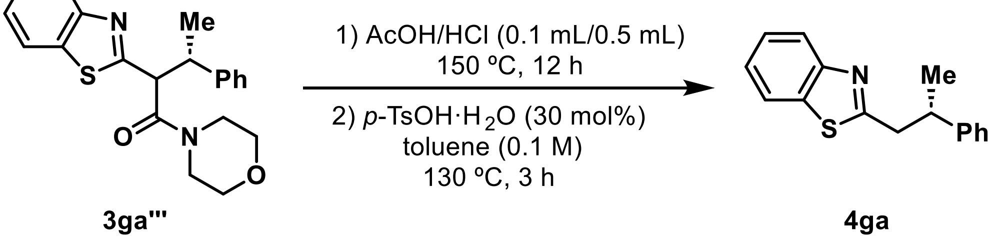

3ga'''
2.2:1 d.r., 91:9 e.r./60:40 e.r.

4ga
56%, 75:25 e.r.

The preceding (3*S*)-2-(benzo[*d*]thiazol-2-yl)-1-morpholino-3-phenylbutan-1-one (33.6 mg, 0.10 mmol) was subjected to a solution of AcOH/HCl (0.10 ml/0.50 mL). The reaction mixture was stirred at 150 °C for 12 h in a heating plate. Then, the resulting mixture was extracted with ethyl acetate (3 × 10 mL). The combined organic phase was dried through anhydrous $MgSO_4$, filtered and concentrated under reduced pressure to afford the crude mixture which was used directly in the next step. To the above crude mixture were added *p*-toluenesulfonic acid monohydrate (5.71 mg, 0.30 mmol) and toluene (0.20 mL). The reaction mixture was stirred at 110 °C for 3 h in a heating plate. Purification by flash column chromatography (Hexane/EtOAc = 100/1 to 20/1) afforded the title product (14.2 mg, 56%, 75:25 e.r.) as a colorless oil.

SFC conditions: CHIRALPACK SB (25 cm), 95:5 CO_2:*i*-PrOH, 2.0 mL/min, 250 nm; *Retention times*: t_{major} = 11.1 min, t_{minor} = 11.6 min.

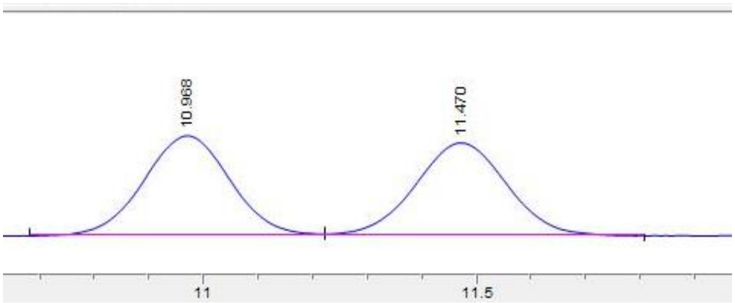

#	Time	Area	Height	Width	Area%	Symmetry
1	10.968	299.7	27	0.1736	50.500	0.984
2	11.47	293.8	25	0.181	49.500	0.984

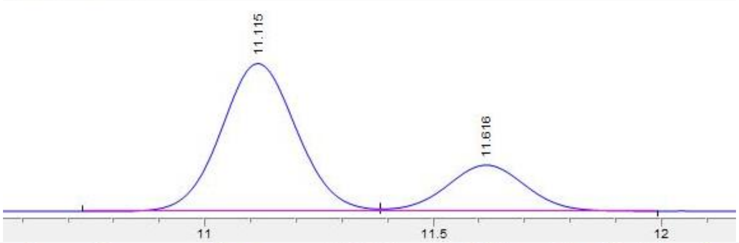

#	Time	Area	Height	Width	Area%	Symmetry
1	11.115	2453.9	212.4	0.1788	75.309	0.931
2	11.616	804.5	66.5	0.1872	24.691	0.985

Detailed Information for Reactivity Investigations of other Azaaryl Acetamides

2-(Benzo[*d*]thiazol-2-yl)-*N*,*N*-dimethylacetamide (S1g’’’’)

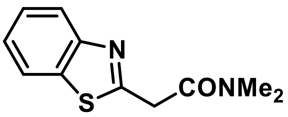

__General procedure C__: 2-Chlorobenzothiazole (679 mg, 4.00 mmol) was employed with *tert*-pentyl acetate (1.05 g, 1.12 mL, 12.0 mmol) and NaHMDS (0.6 M in toluene, 20.0 mL, 12.0 mmol). Purification by flash column chromatography (CH_2Cl_2/MeOH = 50/1 to 10/1) afforded the title compound (850 mg, 96%) as a yellow solid. ^1H NMR (500 MHz, $CDCl_3$) δ_H = 7.98 (dd, *J* = 8.3, 1.1 Hz, 1H), 7.87 (dd, *J* = 8.2, 1.2 Hz, 1H), 7.46 (ddd, *J* = 8.3, 7.2, 1.2 Hz, 1H), 7.37 (ddd, *J* = 8.2, 7.2, 1.1 Hz,

1H), 4.24 (s, 2H), 3.15 (s, 3H), 3.02 (s, 3H); ^{13}C NMR (125 MHz, $CDCl_3$) δ_C = 167.8, 164.8, 152.6, 136.0, 126.1, 125.2, 122.8, 121.7, 39.5, 38.0, 36.0.

The spectroscopic properties were consistent with the data available in the literature.[7]

(3*S*)-2-(Benzo[*d*]thiazol-2-yl)-*N*,*N*-dimethyl-3-phenylbutanamide (S3ga'''')

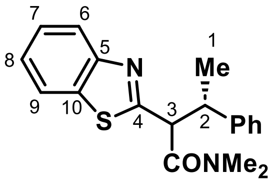

<u>General procedure E</u>: The preceding azaarylacetamide (22.0 mg, 0.10 mmol) and styrene (31.2 mg, 34.4 μL, 0.30 mmol) were employed with $[Ir(cod)_2]$BARF (6.36 mg, 0.005 mmol), **L2** (3.05 mg, 0.005 mmol) and toluene (0.20 mL). The reaction was stirred at 120 °C for 48 h. Purification by flash column chromatography (Hexane/EtOAc = 6/1 to 3/2) afforded the title compound (1.8:1 d.r., B:L = 6.6:1; diastereomer 1: 16.9 mg, 52%, 94:6 e.r.; diastereomer 2: 9.20 mg, 28%, 72:28 e.r.) as colorless solids.

Diastereomer 1: m.p. 125 - 129 °C (EtOAc/hexane). $[\alpha]_D^{24}$ = -35.0 (*c* 0.5, CH_2Cl_2). IR (thin film) ν_{max}/cm^{-1}: 2964, 2923, 1644, 1395, 1134, 763. ^{1}H NMR (500 MHz, $CDCl_3$) δ_H = 8.05 (d, *J* = 8.0 Hz, 1H, C**6**-<u>H</u>), 7.94 (dd, *J* = 8.1, 1.1 Hz, 1H, C**9**-<u>H</u>), 7.53 – 7.49 (ddd, *J* = 8.0, 7.1, 1.1 Hz, 1H, C**7**-<u>H</u>), 7.42 (ddd, *J* = 8.1, 7.1, 1.1 Hz, 1H, C**8**-<u>H</u>), 7.41 – 7.33 (m, 4H, Ph ArC<u>H</u>), 7.29 – 7.25 (m, 1H, Ph ArC<u>H</u>), 4.74 (d, *J* = 10.8 Hz, 1H, C**3**-<u>H</u>), 3.70 (dq, *J* = 10.8, 7.1 Hz, 1H, C**2**-<u>H</u>), 2.78 (s, 3H, NC<u>H</u>$_3$), 2.73 (s, 3H, NC<u>H</u>$_3$'), 1.23 (d, *J* = 7.1 Hz, 3H, C**1**-<u>H</u>$_3$); ^{13}C NMR (125 MHz, $CDCl_3$) δ_C = 170.5 (<u>C</u>=O), 169.6 (C**4**), 152.0 (C**5**), 143.8 (Ph Ar<u>C</u>), 136.3 (C**10**), 128.6 (Ph Ar<u>C</u>H), 127.6 (Ph Ar<u>C</u>H), 127.1 (Ph Ar<u>C</u>H), 126.0 (C**7**), 125.2 (C**8**), 122.9 (C**6**), 121.9 (C**9**), 55.8 (C**3**), 45.6 (C**2**), 37.5 (N<u>C</u>H$_3$), 35.9 (N<u>C</u>H$_3$'), 19.1 (C**1**). HRMS (CI$^+$) calculated for $C_{19}H_{21}N_2O_2S$ $[M+H]^+$ = 341.1318, found 341.1327.

SFC conditions: CHIRALPACK SB (25 cm), 90:10 CO_2:MeOH, 2.0 mL/min, 254 nm; *Retention times*: t_{minor} = 4.4 min, t_{major} = 5.4 min.

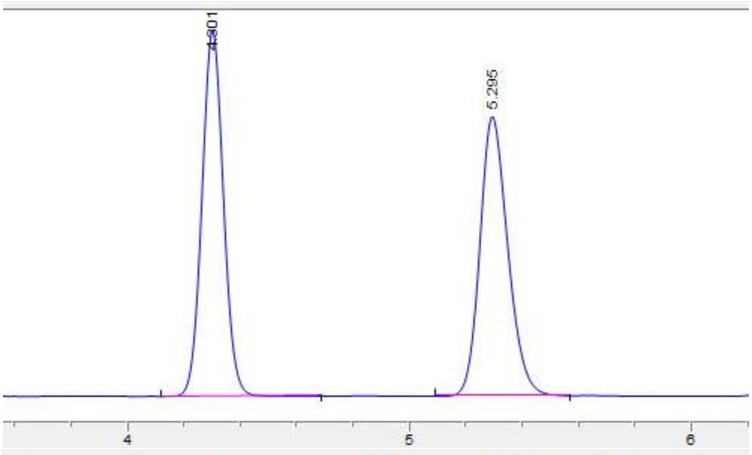

#	Time	Area	Height	Width	Area%	Symmetry
1	4.301	1537.1	291.2	0.0816	50.147	0.926
2	5.295	1528	222	0.1063	49.853	0.765

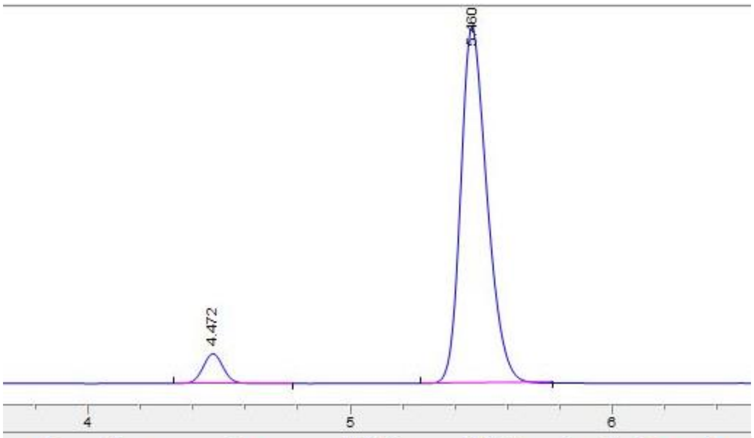

#	Time	Area	Height	Width	Area%	Symmetry
1	4.472	282.7	52.9	0.0824	6.009	0.937
2	5.46	4421.5	633.6	0.1074	93.991	0.699

Diastereomer 2: m.p. 136 - 140 °C (EtOAc/hexane). $[\alpha]_D^{24}$ = -51.4 (*c* 0.5, CH_2Cl_2). IR (thin film) ν_{max}/cm^{-1}: 2964, 2923, 1644, 1395, 1134, 763. ^{1}H NMR (500 MHz, $CDCl_3$) δ_H = 7.81 (d, *J* = 8.1 Hz, 1H, C**6**-H), 7.76 (d, *J* = 7.9 Hz, 1H, C**9**-H), 7.35 (dd, *J* = 8.1, 7.1 Hz, 1H, C**7**-H), 7.28 (dd, *J* = 7.9, 7.1 Hz, 1H, C**8**-H), 7.22 – 7.18 (m, 2H, Ph ArCH), 7.17 – 7.12 (m, 2H, Ph ArCH), 7.07 – 7.04 (m, 1H, Ph ArCH), 4.81 (d, *J* = 10.3 Hz, 1H, C**3**-H), 3.77 (dq, *J* = 10.3, 6.8 Hz, 1H, C**2**-H), 3.18 (s, 3H, NCH_3), 3.01 (s, 3H, NCH_3'), 1.41 (d, *J* = 6.8 Hz, 3H, C**1**-H_3); ^{13}C NMR (125 MHz, $CDCl_3$) δ_C = 170.4 (C=O), 169.2 (C**4**), 152.1 (C**5**), 143.0 (Ph ArC), 135.7 (C**10**), 128.5 (Ph ArCH), 127.8 (Ph ArCH), 126.8 (Ph ArCH), 125.8 (C**7**), 124.9 (C**8**), 122.7 (C**6**), 121.7 (C**9**), 54.5 (C**3**), 45.4 (C**2**), 38.0 (NCH_3), 36.4 (NCH_3'), 21.0 (C**1**). HRMS (CI$^+$) calculated for $C_{19}H_{21}N_2OS$ $[M+H]^+$ = 325.1369, found 325.1374.

SFC conditions: CHIRALPACK SB (25 cm), 90:10 CO_2:MeOH, 2.0 mL/min, 254 nm; *Retention times*: t_{minor} = 4.7 min, t_{major} = 5.1 min.

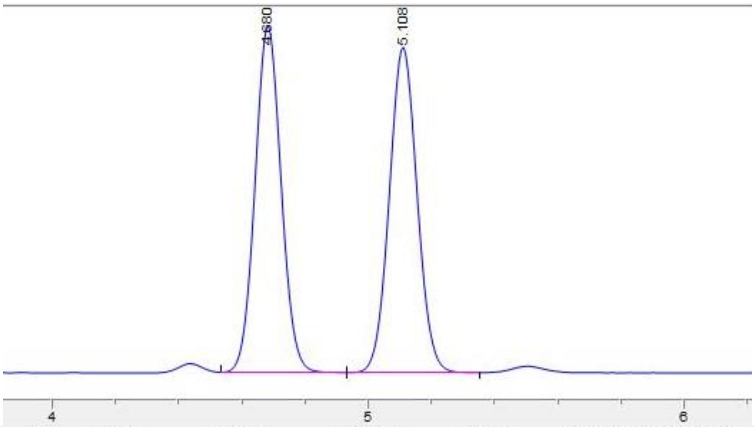

#	Time	Area	Height	Width	Area%	Symmetry
1	4.68	1218.1	212	0.089	50.021	0.92
2	5.108	1217.1	198.9	0.0954	49.979	0.937

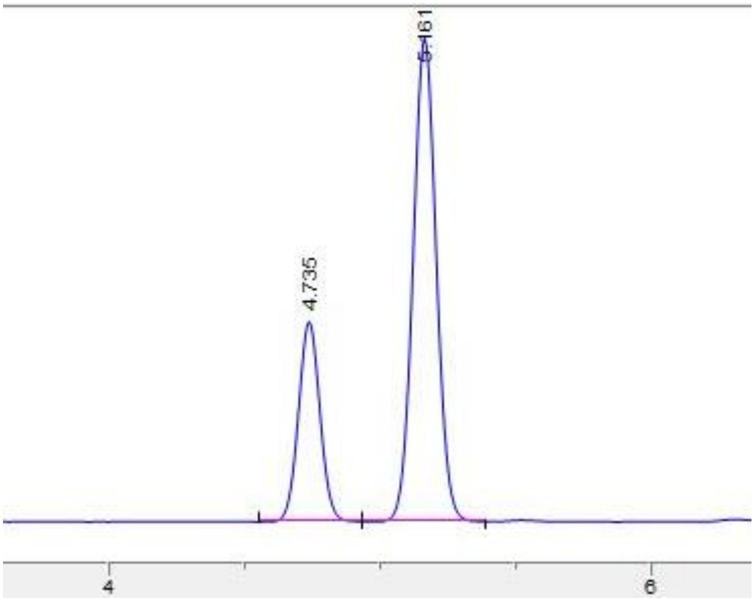

#	Time	Area	Height	Width	Area%	Symmetry
1	4.735	331.5	60	0.0864	27.826	0.943
2	5.161	859.8	145	0.0912	72.174	0.944

2-(Benzo[*d*]thiazol-2-yl)-*N*-methoxy-*N*-methylacetamide (S1g’’’’’)

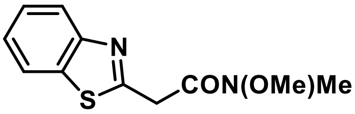

General procedure B: 2-Methylbenzo[*d*]thiazole (597 mg, 4.00 mmol) was employed with *N*-methyl-*N*-methoxycarbamoyl chloride (494 mg, 4.00 mmol) and *n*-BuLi (2.5 M in hexane, 4.00 mL, 10.0 mmol). Purification by flash column chromatography (Hexane/EtOAc = 3/1 to 1/1) afforded the title compound (455 mg, 48%) as a brown solid. ^{1}H NMR (500 MHz, $CDCl_3$) δ_H = 7.99 – 7.95 (m, 1H), 7.86 – 7.80 (m, 1H), 7.45 – 7.39 (m, 1H), 7.36 – 7.31 (m, 1H), 4.30 (s, 2H), 3.71 (s, 3H), 3.22 (s, 3H); ^{13}C NMR (125 MHz, $CDCl_3$) δ_C = 168.9, 164.0, 152.6, 135.9, 125.9, 125.0, 122.8, 121.5, 61.7, 37.7, 32.3.

The spectroscopic properties were consistent with the data available in the literature.[7]

(3*S*)-2-(Benzo[*d*]thiazol-2-yl)-*N*-methoxy-*N*-methyl-3-phenylbutanamide (S3ga’’’’’)

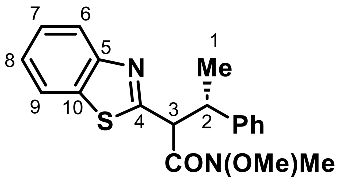

General procedure E: The preceding azaarylacetamide (23.6 mg, 0.10 mmol) and styrene (31.2 mg, 34.4 µL, 0.30 mmol) were employed with [Ir(cod)$_2$]BARF (6.36 mg, 0.005 mmol), **L2** (3.05 mg, 0.005 mmol) and toluene (0.20 mL). The reaction was stirred at 120 °C for 48 h. Purification by flash column chromatography (Hexane/EtOAc = 10/1 to 4/1) afforded the title compound (1.8:1 d.r., B:L = 5.7:1; diastereomer 1: 15.2 mg, 45%, 90.5:9.5 e.r.; diastereomer 2: 8.10 mg, 24%, 51.5:48.5 e.r.) as yellow oils.

Diastereomer 1: $[\alpha]_D^{24}$ = -22.6 (*c* 0.5, CH_2Cl_2). IR (thin film) ν_{max}/cm^{-1}: 2968, 2933, 1658, 1455, 1154, 760. ^{1}H NMR (500 MHz, $CDCl_3$) δ_H = 8.04 (d, *J* = 8.2 Hz, 1H, C**6**-H), 7.90 (dd, *J* = 8.0, 1.2 Hz, 1H, C**9**-H), 7.48 (ddd, *J* = 8.2, 7.1, 1.2 Hz, 1H, C**7**-H), 7.39 (ddd, *J* = 8.0, 7.1, 1.1 Hz, 1H, C**8**-H), 7.37 – 7.34 (m, 2H, Ph ArCH), 7.33 – 7.28 (m, 2H, Ph ArCH), 7.24 – 7.20 (m, 1H, Ph ArCH), 5.04 (d, *J* = 11.1 Hz, 1H, C**3**-H), 3.69 (dq, *J* = 11.1, 7.1 Hz, 1H, C**2**-H), 3.47 (s, 3H, $NOCH_3$), 2.91 (s, 3H, NCH_3), 1.21 (d, *J* = 7.1 Hz, 3H, C**1**-H_3); ^{13}C NMR (125 MHz, $CDCl_3$) δ_C = 171.2 (C=O), 169.0 (C**4**), 152.3 (C**5**), 143.7 (Ph ArC), 136.0 (C**10**), 128.5 (Ph ArCH), 127.8 (Ph ArCH), 127.0 (Ph ArCH), 126.0 (C**7**), 125.3 (C**8**), 123.1 (C**6**), 121.8 (C**9**), 61.9 ($NOCH_3$), 54.2 (C**3**), 44.8 (C**2**), 32.1 (NCH_3), 19.6 (C**1**). HRMS (CI^+) calculated for $C_{19}H_{21}N_2O_2S$ $[M+H]^+$ = 341.1318, found 341.1327.

SFC conditions: CHIRALPACK SB (25 cm), 85:15 CO_2:*i*-PrOH, 2.0 mL/min, 254 nm; *Retention times*: t_{minor} = 4.0 min, t_{major} = 4.4 min.

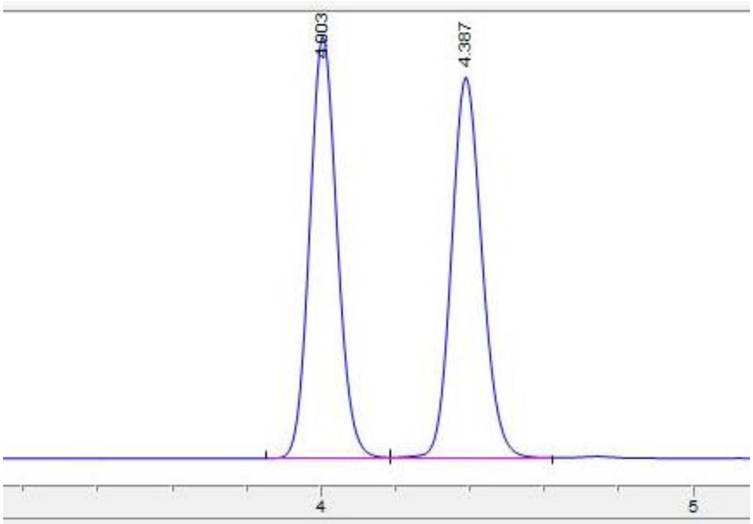

#	Time	Area	Height	Width	Area%	Symmetry
1	4.003	2954.4	577.2	0.0797	49.849	0.874
2	4.387	2972.3	521.2	0.0885	50.151	0.843

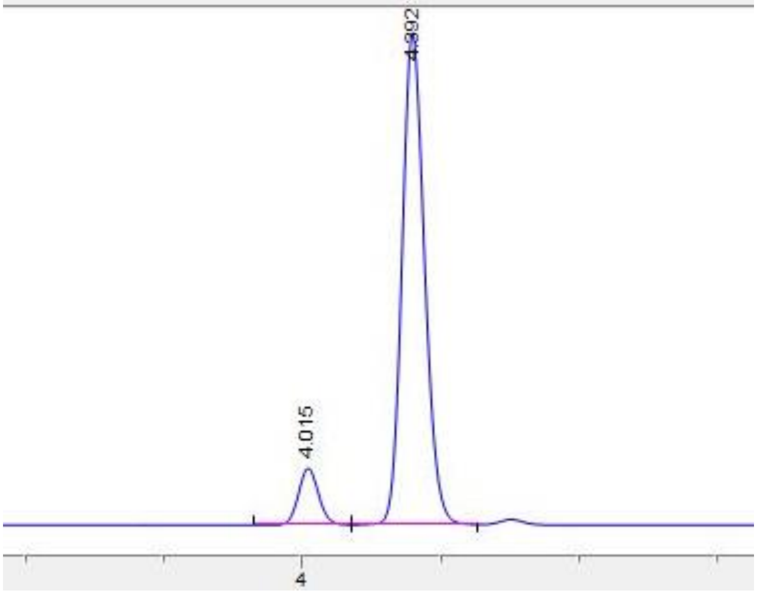

#	Time	Area	Height	Width	Area%	Symmetry
1	4.015	468.8	92.5	0.0792	9.371	0.895
2	4.392	4534.4	800.2	0.0881	90.629	0.816

Diastereomer 2: $[\alpha]_D^{25}$ = -2.08 (*c* 0.2, CH_2Cl_2). IR (thin film) ν_{max}/cm^{-1}: 2976, 2937, 1658, 1455, 1170, 761. ^1H NMR (500 MHz, $CDCl_3$) δ_H = 7.84 (d, *J* = 8.1 Hz, 1H, C**6**-H), 7.75 (d, *J* = 8.0 Hz, 1H, C**9**-H), 7.34 (dd, *J* = 8.1, 7.1 Hz, 1H, C**7**-H), 7.28 (dd, *J* = 8.0, 7.1 Hz, 1H, C**8**-H), 7.22 – 7.18 (m, 2H, Ph ArCH), 7.16 – 7.12 (m, 2H, Ph ArCH), 7.08 – 7.03 (m, 1H, Ph ArCH), 5.09 (d, *J* = 10.9 Hz, 1H, C**3**-H), 3.76 (s, 3H, $NOCH_3$), 3.72 (dq, *J* = 10.9, 6.8 Hz, 1H, C**2**-H), 3.27 (s, 3H, NCH_3), 1.42 (d, *J* = 6.8 Hz, 3H, C**1**-H_3); ^{13}C NMR (125 MHz, $CDCl_3$) δ_C = 171.5 (C=O), 168.6 (C**4**), 152.1 (C**5**), 142.9 (Ph ArC), 135.7 (C**10**), 128.5 (Ph ArCH), 127.8 (Ph ArCH), 126.8 (Ph ArCH), 125.7 (C**7**), 124.9 (C**8**), 123.0 (C**6**), 121.6 (C**9**), 62.2 ($NOCH_3$), 53.2 (C**3**), 44.9 (C**2**), 32.5 (NCH_3), 21.2 (C**1**). HRMS (CI^+) calculated for $C_{19}H_{21}N_2O_2S$ $[M+H]^+$ = 341.1318, found 341.1312.

SFC conditions: CHIRALPACK IB (25 cm), 85:15 CO_2:*i*-PrOH, 2.0 mL/min, 254 nm; *Retention times*: t_{minor} = 4.0 min, t_{major} = 4.4 min.

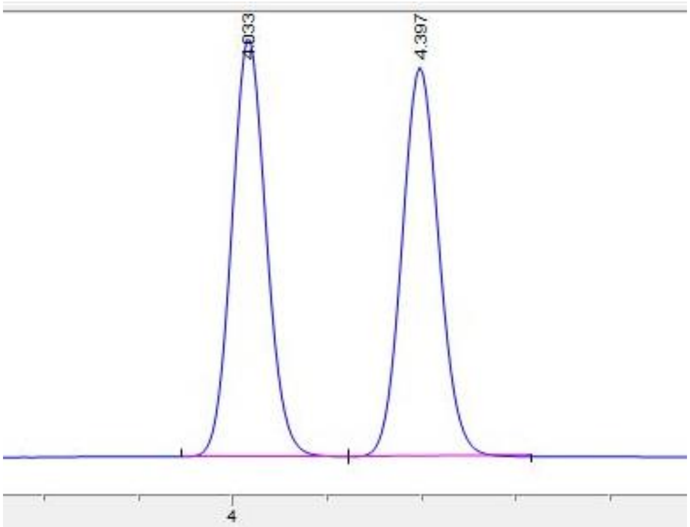

#	Time	Area	Height	Width	Area%	Symmetry
1	4.033	982.5	193.3	0.0793	50.031	0.891
2	4.397	981.3	179.7	0.0857	49.969	0.896

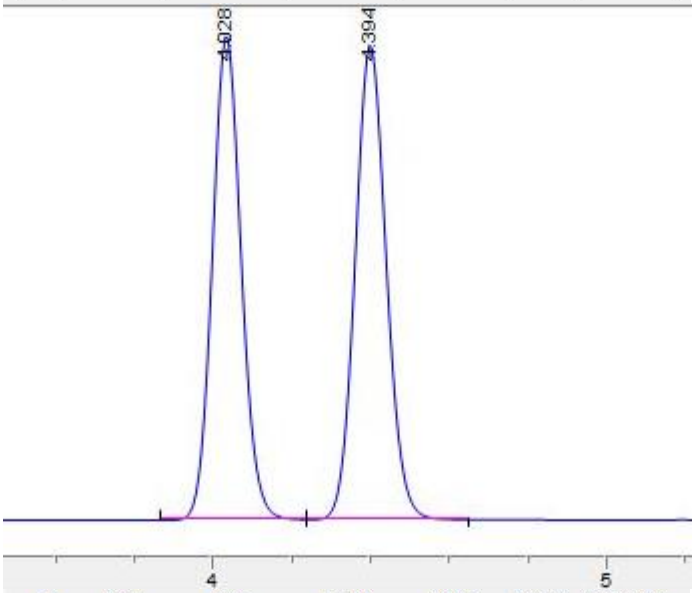

#	Time	Area	Height	Width	Area%	Symmetry
1	4.028	1639.5	322.1	0.0794	48.658	0.887
2	4.394	1730	315.8	0.0839	51.342	0.889

Further Utility of the Hydroalkylation Process

(*R*)-2-(2,5-Dimethylcyclopent-1-en-1-yl)benzo[*d*]thiazole (5)

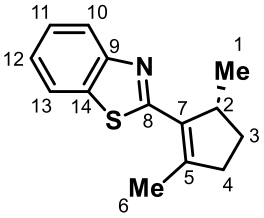

A Schlenk tube was charged with *tert*-Butyl 2-(benzo[*d*]thiazol-2-yl)acetate (24.9 mg, 0.10 mmol), [Ir(cod)$_2$]BARF (6.36 mg, 0.005 mmol) and **L6** (1.91 mg, 0.005 mmol). The Schlenk tube was evacuated/backfilled with N_2 (three cycles), then hex-5-en-2-one (14.7 mg, 17.4 µL, 0.15 mmol) was added followed by *m*-xylene (0.20 mL). The tube was sealed and heated at 70 °C for 84 h. After cooling to room temperature, the crude mixture was filtered through a short pad of silica gel to remove Ir/Ligand complex and washed with *m*-xylene (0.10 mL). The filtrate was transferred to a Schlenk tube and *p*-toluenesulfonic acid monohydrate (5.71 mg, 0.03 mmol) was added. The resulting reaction mixture was heated at 120 °C for 3 h. After cooling to room temperature, the solvent was removed under reduced pressure and the crude reaction mixture was purified by flash column chromatography (Hexane/EtOAc = 30/1 to 15/1) to give the title compound (11.3 mg, 49%, B:L > 25:1, 91:9 e.r.) as a colorless oil. $[\alpha]_D^{24}$ = +34.9 (*c* 0.5, CH_2Cl_2). IR (thin film) ν_{max}/cm^{-1}: 2953, 2869, 1630, 1434, 1017, 761. ^{1}H NMR (500 MHz, $CDCl_3$) δ_H = 8.03 (d, *J* = 8.2 Hz, 1H, C**10**-H), 7.87 (dd, *J* = 7.9, 1.2 Hz, 1H, C**13**-H), 7.45 (ddd, *J* = 8.2, 7.3, 1.2 Hz, 1H, C**11**-H), 7.36 – 7.31 (m, 1H, C**12**-H), 3.47 – 3.37 (m, 1H, C**2**-H), 2.78 – 2.66 (m, 1H, C**4**-H), 2.47 (ddd, *J* = 17.6, 9.5, 3.7 Hz, 1H, C**4**-H’), 2.27 – 2.20 (m, 1H, C**3**-H), 2.19 (s, 3H, C**6**-H_3), 1.64 – 1.57 (m, 1H, C**3**-H’), 1.22 (d, *J* = 6.9 Hz, 3H, C**1**-H_3); ^{13}C NMR (125 MHz, $CDCl_3$) δ_C = 164.7 (C**8**), 153.5 (C**9**), 145.9 (C**5**), 135.2 (C**14**), 134.9 (C**7**), 126.0 (C**11**), 124.6 (C**12**), 122.9 (C**10**), 121.3 (C**13**), 43.5 (C**2**), 39.2 (C**4**), 31.2 (C**3**), 20.2 (C**1**), 16.7 (C**6**). HRMS (ESI$^+$) calculated for $C_{14}H_{16}NS$ $[M+H]^+$ = 230.0998, found 230.1003.

SFC conditions: CHIRALPACK SC (25 cm), 97:3 CO_2:*i*-PrOH, 1.5 mL/min, 254 nm; *Retention times*: t_{major} = 11.6 min, t_{minor} = 14.2 min.

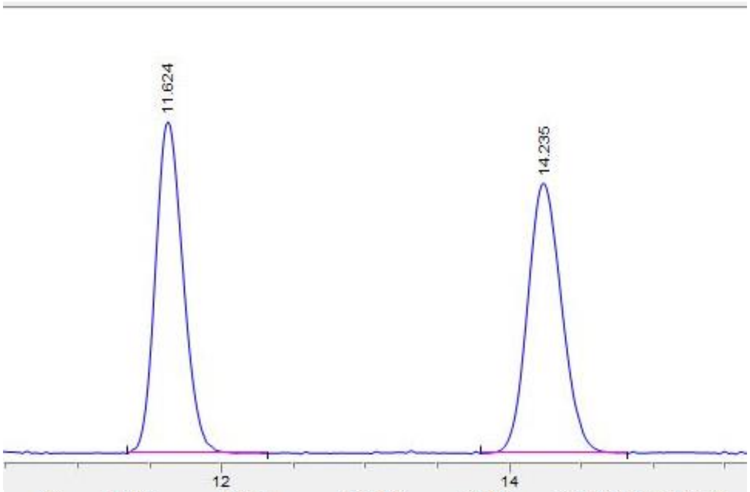

#	Time	Area	Height	Width	Area%	Symmetry
1	11.624	2307.7	171.9	0.2046	49.921	0.805
2	14.235	2315	140	0.2589	50.079	0.84

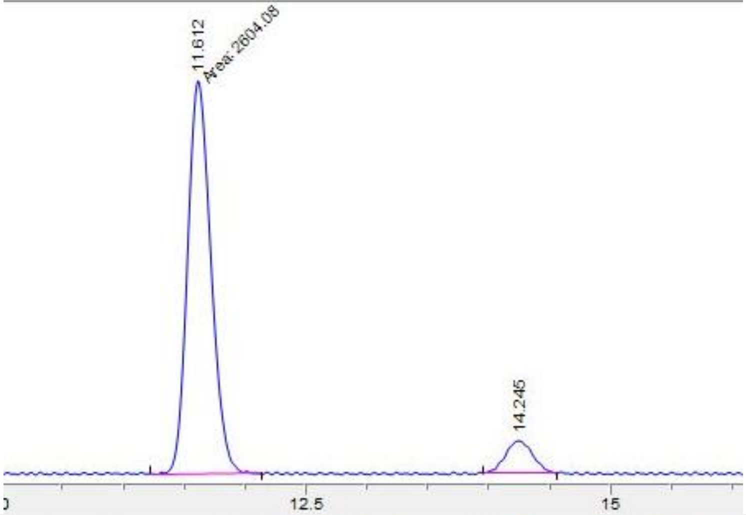

#	Time	Area	Height	Width	Area%	Symmetry
1	11.612	2604.1	193	0.2249	90.797	0.812
2	14.245	263.9	16.3	0.2175	9.203	0.948

tert-Butyl (3*R*)-2-(benzo[*d*]thiazol-2-yl)-2-fluoro-3-phenylbutanoate (6)

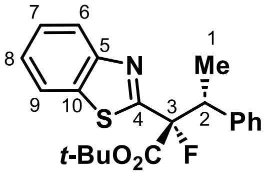

Major diastereomer

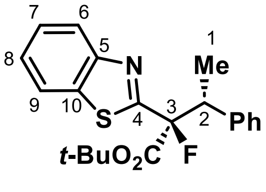

Minor diastereomer

A solution of *tert*-butyl (3*S*)-2-(benzo[*d*]thiazol-2-yl)-3-phenylbutanoate (47.7 mg, 0.135 mmol; 3.1:1 d.r., 98.5:1.5 e.r./98.5:1.5 e.r.) in dry THF (0.41 mL) at –78 °C was added LiHMDS (1.0 M in THF, 202 µL, 0.202 mmol) slowly and the resulting solution was stirred at –78 °C for 5 min before warming to 0 °C for another 1 h. Then, NFSI (63.8 mg, 0.202 mmol) was added one portion at –78 °C and the reaction mixture was stirred at –78 °C for 5 min before allowing to warm to –5 °C – 0 °C for additional 1 h. The reaction was quenched with water (1 mL). The reaction mixture was extracted with diethyl

ether (3 × 2 mL). The combined organic phase was washed with brine (2 mL), dried over Na_2SO_4, filtered and concentrated *in vacuo*. The residue was purified by flash column chromatography (Hexane/EtOAc = 40/1 to 19/1) to give the title compounds (4:1 d.r.; major diastereomer: 33.0 mg, 66%, 99:1 e.r.; minor diastereomer 2: 8.30 mg, 16%, 99:1 e.r.) as colorless oils.

Major diastereomer: $[\alpha]_D^{23}$ = +78.4 (*c* 0.5, CH_2Cl_2). IR (thin film) ν_{max}/cm^{-1}: 2980, 2933, 1751, 1456, 1161, 760. ^{1}H NMR (500 MHz, $CDCl_3$) δ_H = 8.16 (dd, *J* = 8.3, 1.1 Hz, 1H, C**6**-H), 7.94 (dd, *J* = 8.0, 1.1 Hz, 1H, C**9**-H), 7.53 (ddd, *J* = 8.3, 7.2, 1.1 Hz, 1H, C**7**-H), 7.49 – 7.41 (m, 3H, C**8**-H + 2 × Ph ArCH), 7.32 (dd, *J* = 8.2, 6.6 Hz, 2H, Ph ArCH), 7.29 – 7.25 (m, 1H, Ph ArCH), 4.18 (dq, *J* = 33.0, 7.2 Hz, 1H, C**2**-H), 1.32 (d, *J* = 7.2 Hz, 3H, C**1**-H_3), 1.14 (s, 9H, C(CH_3)$_3$); ^{13}C NMR (125 MHz, $CDCl_3$) δ_C = 167.5 (d, $^2J_{CF}$ = 31.3 Hz, C=O), 165.5 (d, $^2J_{CF}$ = 26.6 Hz, C**4**), 153.3 (C**5**), 140.6 (d, $^3J_{CF}$ = 1.5 Hz, Ph ArC), 135.4 (d, $^4J_{CF}$ = 2.1 Hz, C**10**), 129.2 (d, $^4J_{CF}$ = 2.5 Hz, Ph ArCH), 128.4 (Ph ArCH), 127.5 (Ph ArCH), 126.4 (C**7**), 125.7 (C**8**), 124.0 (C**6**), 121.9 (C**9**), 99.3 (d, $^1J_{CF}$ = 196.3 Hz, C**3**), 83.9 (C(CH_3)$_3$), 46.9 (d, $^2J_{CF}$ = 19.0 Hz, C**2**), 27.5 (C**1**), 16.4 (d, $^5J_{CF}$ = 5.9 Hz, C(CH_3)$_3$); ^{19}F NMR (471 MHz, $CDCl_3$) δ_F = -164.2 (d, *J* = 33.0 Hz, 1F). HRMS (ESI$^+$) calculated for $C_{21}H_{22}FNNaO_2S$ [M+Na]$^+$ = 394.1247, found 394.1251.

SFC conditions: CHIRALPACK SC (25 cm), 95:5 CO_2:*i*-PrOH, 2.0 mL/min, 254 nm; *Retention times*: t_{minor} = 5.0 min, t_{major} = 8.5 min.

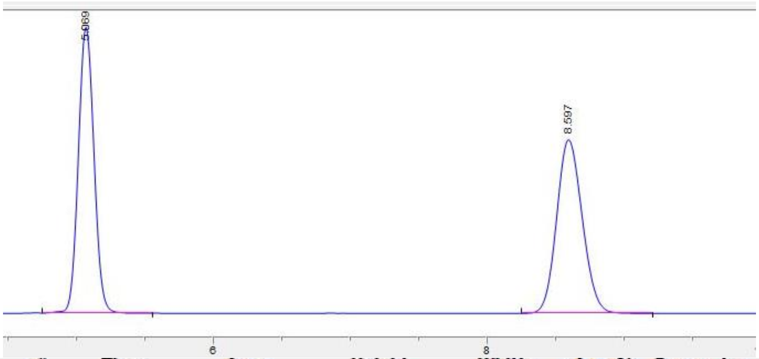

#	Time	Area	Height	Width	Area%	Symmetry
1	5.069	1970.3	247.9	0.1267	50.147	0.912
2	8.597	1958.8	150.6	0.2017	49.853	0.84

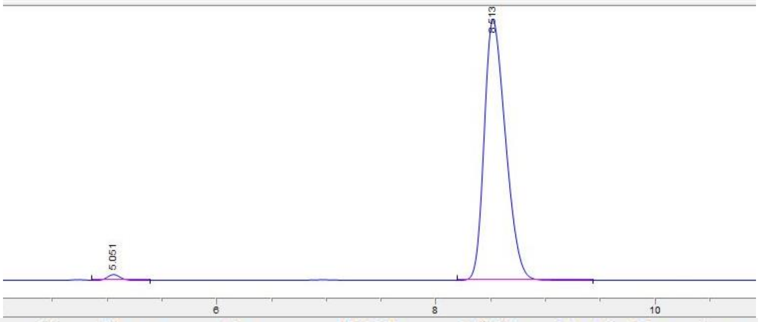

#	Time	Area	Height	Width	Area%	Symmetry
1	5.051	78.2	9.7	0.1285	1.294	0.886
2	8.513	5965	440.1	0.2101	98.706	0.664

Minor diastereomer: $[\alpha]_D^{22}$ = +79.5 (c 0.5, CH_2Cl_2). IR (thin film) ν_{max}/cm^{-1}: 2921, 2853, 1749, 1456, 1157, 762. ^{1}H NMR (500 MHz, $CDCl_3$) δ_H = 8.05 (d, J = 8.2 Hz, 1H, C**6**-H), 7.75 (d, J = 8.0 Hz, 1H, C**9**-H), 7.44 (dd, J = 8.2, 7.2 Hz, 1H, C**7**-H), 7.33 (dd, J = 8.0, 7.2 Hz, 1H, C**8**-H), 7.24 – 7.19 (d, J = 7.2 Hz, 2H, Ph ArCH), 7.13 – 7.06 (m, 3H, Ph ArCH), 4.13 (dq, J = 32.8, 7.3 Hz, 1H, C**2**-H), 1.55 (d, J = 7.3 Hz, 3H, C**1**-H_3), 1.53 (s, 9H, C(CH_3)$_3$); ^{13}C NMR (125 MHz, $CDCl_3$) δ_C = 167.4 (d, $^2J_{CF}$ = 31.4 Hz, C=O), 166.1 (d, $^2J_{CF}$ = 25.2 Hz, C**4**), 153.0 (C**5**), 139.4 (Ph ArC), 135.1 (d, $^4J_{CF}$ = 1.8 Hz, C**10**), 129.4 (d, $^4J_{CF}$ = 2.3 Hz, Ph ArCH), 128.1 (Ph ArCH), 127.2 (Ph ArCH), 126.1 (C**7**), 125.4 (C**8**), 123.8 (C**6**), 121.6 (C**9**), 99.2 (d, $^1J_{CF}$ = 196.1 Hz, C**3**), 84.4 (C(CH_3)$_3$), 46.7 (d, $^2J_{CF}$ = 19.9 Hz, C**2**), 28.0 (C**1**), 16.1 (d, $^5J_{CF}$ = 4.6 Hz, C(CH_3)$_3$); ^{19}F NMR (471 MHz, $CDCl_3$) δ_F = -165.7 (d, J = 32.8 Hz, 1F). HRMS (ESI$^+$) calculated for $C_{21}H_{22}FNNaO_2S$ $[M+Na]^+$ = 394.1247, found 394.1241.

SFC conditions: CHIRALPACK SC (25 cm), 95:5 CO_2:*i*-PrOH, 2.0 mL/min, 254 nm; *Retention times*: t_{major} = 4.7 min, t_{minor} = 13.1 min.

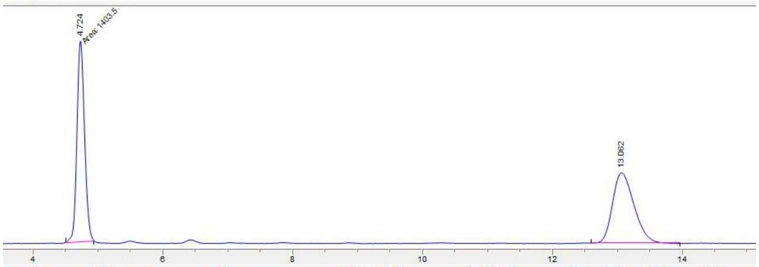

#	Time	Area	Height	Width	Area%	Symmetry
1	4.724	1403.5	171.3	0.1365	50.375	0.85
2	13.062	1382.6	60	0.3589	49.625	0.696

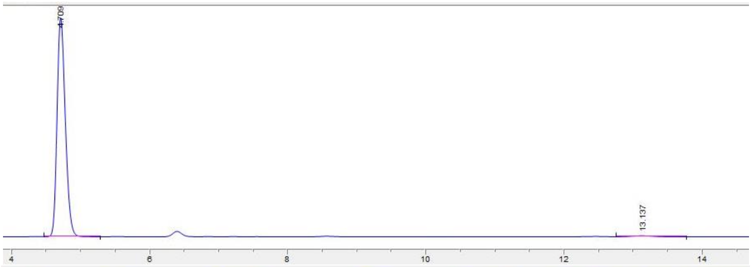

#	Time	Area	Height	Width	Area%	Symmetry
1	4.709	11349.4	1363.4	0.1291	98.869	0.737
2	13.137	129.9	6	0.2631	1.131	0.914

***tert*-Butyl (3*R*)-2-(benzo[*d*]thiazol-2-yl)-2-methyl-3-phenylbutanoate (7)**

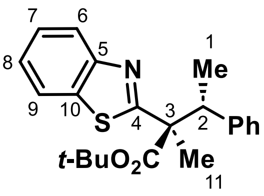

Major diastereomer

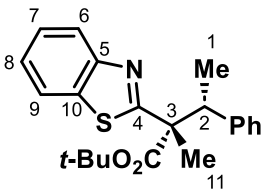

Minor diastereomer

A solution of diisopropylamine (65.3 mg, 90.6 µL, 0.645 mmol) in dry THF (0.13 mL) at –78 °C was added *n*-BuLi (2.5 M in hexane, 0.26 mL, 0.645 mmol) over a period of 15 min and the resulting solution was stirred at –78 °C for additional 30 min. To the above reaction mixture was added *tert*-butyl

(3*S*)-2-(benzo[*d*]thiazol-2-yl)-3-phenylbutanoate (45.7 mg, 0.129 mmol; 3.1:1 d.r., 98.5:1.5 e.r./98.5:1.5 e.r.) in dry THF (0.13 mL) dropwise and the reaction mixture was stirred for another 1 h at –78 °C. Then, iodomethane (128 mg, 56.2 µL, 0.903 mmol) was added and the reaction mixture was stirred for another 15 min at –78 °C. After that, the reaction mixture was allowed to warm to room temperature and stirred for 3 h. The reaction was quenched with water (1 mL). The reaction mixture was extracted with diethyl ether (3 × 2 mL). The combined organic phase was washed with brine (2 mL), dried over Na_2SO_4, filtered and concentrated *in vacuo*. The residue was purified by flash column chromatography (Hexane/EtOAc = 30/1 to 15/1) to give the title compounds (32.3 mg, 68%, 9:1 d.r., 99:1 e.r./99:1 e.r.) as colorless oils. IR (thin film) ν_{max}/cm^{-1}: 2977, 2929, 1732, 1455, 1126, 760. Major diastereomer: ^{1}H NMR (500 MHz, $CDCl_3$) δ_H = 8.06 (d, *J* = 8.0 Hz, 1H, C**6**-H), 7.89 (d, *J* = 7.6 Hz, 1H, C**9**-H), 7.50 – 7.45 (m, 1H, C**7**-H), 7.41 – 7.36 (m, 1H, C**8**-H), 7.32 – 7.20 (m, 5H, Ph ArCH), 3.97 (q, *J* = 7.2 Hz, 1H, C**2**-H), 1.74 (s, 3H, C**11**-H_3), 1.32 (s, 9H, C(CH_3)$_3$), 1.23 (d, *J* = 7.2 Hz, 3H, C**1**-H_3); ^{13}C NMR (125 MHz, $CDCl_3$) δ_C = 173.4 (C=O), 171.9 (C**4**), 152.5 (C**5**), 141.7 (Ph ArC), 135.9 (C**10**), 129.6 (Ph ArCH), 128.0 (Ph ArCH), 127.1 (Ph ArCH), 125.9 (C**7**), 125.1 (C**8**), 123.4 (C**6**), 121.5 (C**9**), 82.4 (C(CH_3)$_3$), 58.4 (C**3**), 48.3 (C**2**), 27.9 (C(CH_3)$_3$), 17.6 (C**11**), 16.4 (C**1**). Minor diastereomer: ^{1}H NMR (500 MHz, $CDCl_3$) δ_H = 7.93 (d, *J* = 8.1 Hz, 1H, C**6**-H), 7.82 (d, *J* = 7.9 Hz, 1H, C**9**-H), 7.44 – 7.39 (m, 1H, C**7**-H), 7.36 – 7.31 (m, 1H, C**8**-H), 7.11 – 7.06 (m, 3H, Ph ArCH), 7.04 – 7.00 (m, 2H, Ph ArCH), 3.94 (q, *J* = 7.2 Hz, 1H, C**2**-H), 1.65 (s, 3H, C**11**-H_3), 1.53 (s, 9H, C(CH_3)$_3$), 1.46 (d, *J* = 7.2 Hz, 3H, C**1**-H_3); ^{13}C NMR (125 MHz, $CDCl_3$) δ_C = 173.4 (C=O), 172.1 (C**4**), 152.5 (C**5**), 141.4 (Ph ArC), 135.4 (C**10**), 129.1 (Ph ArCH), 127.8 (Ph ArCH), 126.9 (Ph ArCH), 125.8 (C**7**), 124.9 (C**8**), 123.2 (C**6**), 121.4 (C**9**), 82.6 (C(CH_3)$_3$), 58.1 (C**3**), 48.5 (C**2**), 28.1 (C(CH_3)$_3$), 18.7 (C**11**), 16.9 (C**1**). HRMS (ESI^{+}) calculated for $C_{22}H_{25}NNaO_2S$ [M+Na]$^{+}$ = 390.1498, found 390.1499.

SFC conditions: CHIRALPACK SC (25 cm), 95:5 CO_2:*i*-PrOH, 2.0 mL/min, 254 nm; *Retention times*: major diastereomer: t_{minor} = 5.2 min, t_{major} = 6.6 min; minor diastereomer: t_{major} = 4.6 min, t_{minor} = 6.2 min.

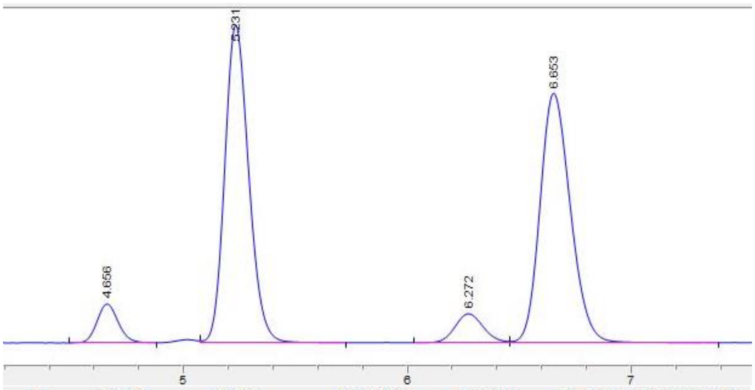

#	Time	Area	Height	Width	Area%	Symmetry
1	4.656	297.1	45	0.101	4.907	0.855
2	5.231	2737.5	368.2	0.1167	45.215	0.801
3	6.272	294.4	33.5	0.1364	4.862	0.88
4	6.653	2725.5	288.9	0.1461	45.016	0.778

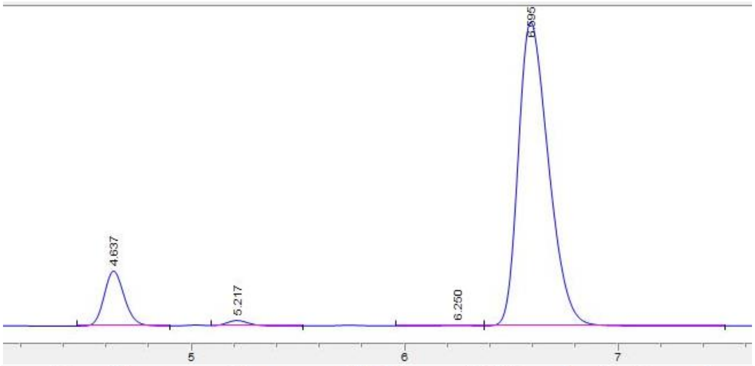

#	Time	Area	Height	Width	Area%	Symmetry
1	4.637	1304.7	198.2	0.1028	10.639	0.829
2	5.217	145.9	19.8	0.1119	1.190	0.857
3	6.25	19.7	2	0.1407	0.161	1.187
4	6.595	10793	1096.9	0.1528	88.010	0.656

Studies on the Relative Stereochemical Assignment of 7

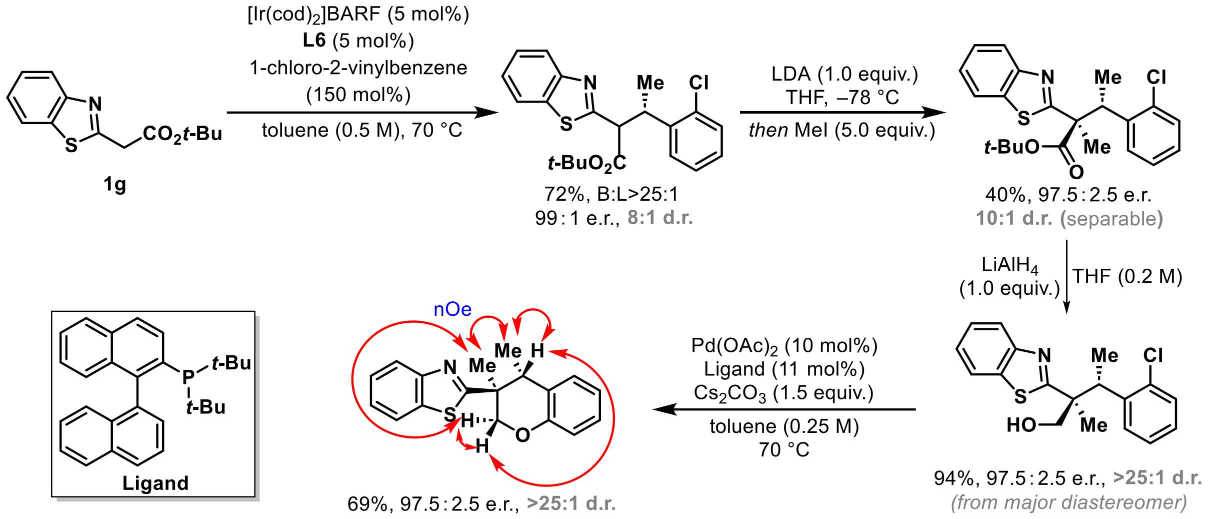

The relative stereochemistries of **6** and **7** were assigned by analogy.

***tert*-Butyl (3*S*)-2-(benzo[*d*]thiazol-2-yl)-3-(2-chlorophenyl)butanoate**

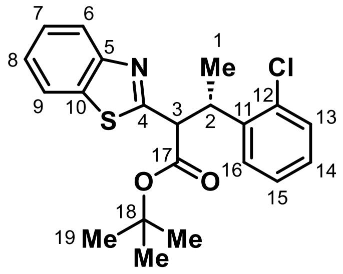

__General procedure E__: *tert*-Butyl 2-(benzo[*d*]thiazol-2-yl)acetate (24.9 mg, 0.10 mmol) and 1-chloro-2-vinylbenzene (20.8 mg, 19.2 μL, 0.15 mmol) were employed with $[Ir(cod)_2]BARF$ (6.36 mg, 0.005 mmol) and **L6** (1.91 mg, 0.005 mmol) in toluene (0.20 mL). The reaction was stirred at 70 °C for 48 h. Purification by flash column chromatography (Hexane/EtOAc = 100/1 to 20/1) afforded the title compound (8:1 d.r., B:L > 25:1; diastereomer 1: 24.8 mg, 64%, 99:1 e.r.; diastereomer 2: 3.1 mg, 8%) as colorless solids.

Diastereomer 1 (Major, 25:1): m.p. 89 – 90 °C (EtOAc/hexane). $[\alpha]_D^{25}$ = –8.0 (c 0.5, CH_2Cl_2). IR (thin film) ν_{max}/cm^{-1}: 2967, 2931, 1727, 1477, 1368, 755. ^{1}H NMR (400 MHz, $CDCl_3$) δ_H = 8.06 (d, J = 8.2 Hz, 1H, C**6**-H), 7.91 (d, J = 8.0 Hz, 1H, C**9**-H), 7.52 – 7.46 (m, 1H, C**7**-H), 7.43 – 7.36 (m, 3H, C**8**-H, C**16**-H + C**13**-H or C**15**-H), 7.29 – 7.24 (m, 1H, C**15**-H or C**13**-H), 7.20 – 7.14 (m, 1H, C**14**-H), 4.53 (d, J = 11.4 Hz, 1H, C**3**-H), 4.19 – 4.09 (m, 1H, C**2**-H), 1.47 (s, 0.36H, 3×C**19**-H_3 for minor), 1.42 (d, J = 6.8 Hz, 0.12H, C**1**-H_3 for minor), 1.17 (d, J = 7.0 Hz, 3H, C**1**-H_3), 1.16 (s, 9H, 3×C**19**-H_3); ^{13}C NMR (101 MHz, $CDCl_3$) δ_C = 168.9 (C**4** or C**17**), 167.8 (C**4** or C**17**), 152.5 (C**5**), 141.0 (C**11**), 135.8 (C**10**), 133.9 (C**12**), 129.9 (C**13** or C**15**), 128.3 (C**16**), 128.0 (C**14**), 127.2 (C**15** or C**13**), 126.1 (C**7**), 125.4 (C**8**), 123.3 (C**6**), 121.8 (C**9**), 82.3 (C**18**), 58.7 (C**3**), 41.1 (C**2**), 28.0 (C**19** for the minor), 27.5 (C**19**), 19.5 (C**1**). HRMS (ESI$^+$) calculated for $C_{21}H_{23}ClNO_2S$ $[M+H]^+$ = 388.1133, found 388.1132.

SFC conditions: CHIRALPACK SC (25 cm), 98:2 CO_2:MeOH, 1.5 mL/min, 250 nm; *Retention times*: t_{minor} = 10.8 min, t_{major} = 14.7 min.

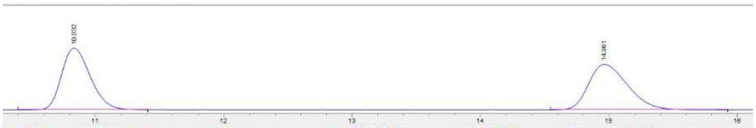

#	Time	Area	Height	Width	Area%	Symmetry
1	10.832	2822	181.2	0.2437	49.987	0.729
2	14.961	2823.4	133.5	0.3228	50.013	0.655

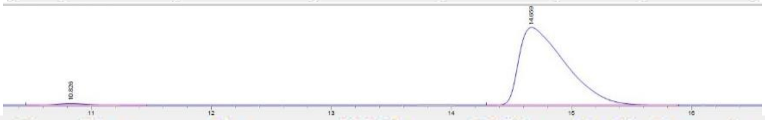

#	Time	Area	Height	Width	Area%	Symmetry
1	10.826	213.3	12.2	0.2524	1.128	0.825
2	14.659	18697.8	700.3	0.4135	98.872	0.348

***tert*-Butyl (2*S*,3*S*)-2-(benzo[*d*]thiazol-2-yl)-3-(2-chlorophenyl)-2-methylbutanoate**

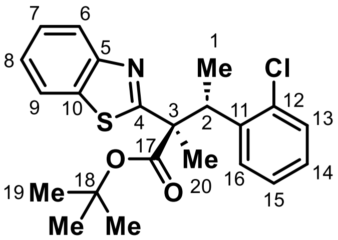

A solution of diisopropylamine (81.0 mg, 113 µL, 0.80 mmol) in dry THF (0.21 mL) at –78 °C was added *n*-BuLi (2.5 M in hexane, 0.32 mL, 0.80 mmol) over a period of 15 min and the resulting solution was stirred at –78 °C for additional 30 min. To the above reaction mixture was added *tert*-butyl (3*S*)-2-(benzo[*d*]thiazol-2-yl)-3-(2-chlorophenyl)butanoate (310 mg, 0.80 mmol) in dry THF (0.21 mL) dropwise and the reaction mixture was stirred for another 1 h at –78 °C. Then, iodomethane (568 mg, 249 µL, 4.0 mmol) was added and the reaction mixture was stirred for another 15 min at –78 °C. After that, the reaction mixture was allowed to warm to room temperature and stirred for 3 h. The reaction was quenched with water (1 mL). The reaction mixture was extracted with diethyl ether (3 × 2 mL).

The combined organic phase was washed with brine (2 mL), dried over Na_2SO_4, filtered and concentrated *in vacuo*. The residue was purified by flash column chromatography (Hexane/EtOAc = 30/1 to 15/1) to give the title compounds (128.6 mg, 40%, 10:1 d.r; diastereomer 1: 116.9 mg, 36%, 97.5:2.5 e.r.; diastereomer 2: 11.7 mg, 4%) as colorless solids.

Diastereomer 1 (Major): m.p. 71 – 72 °C (EtOAc/hexane). $[\alpha]_D^{24}$ = –3.5 (*c* 0.5, CH_2Cl_2). IR (thin film) ν_{max}/cm^{-1}: 2969, 2932, 1731, 1436, 1367, 753. ^{1}H NMR (400 MHz, $CDCl_3$) δ_H = 8.05 (d, *J* = 8.2 Hz, 1H, C**6**-H), 7.89 (d, *J* = 8.0 Hz, 1H, C**9**-H), 7.50 – 7.45 (m, 1H, C**7**-H), 7.42 – 7.34 (m, 2H, C**8**-H + C**13**-H or C**15**-H), 7.15 – 7.10 (m, 1H, C**15**-H or C**13**-H), 7.10 – 7.01 (m, 2H, C**14**-H + C**16**-H), 4.54 (q, *J* = 7.2 Hz, 1H, C**2**-H), 1.89 (s, 3H, C**20**-H_3), 1.37 (d, *J* = 7.2 Hz, 3H, C**1**-H_3), 1.30 (s, 9H, 3×C**19**-H_3); ^{13}C NMR (101 MHz, $CDCl_3$) δ_C = 172.7 (C**17** or C**4**), 171.9 (C**4** or C**17**), 152.4 (C**5**), 140.5 (C**11**), 135.8 (C**10**), 134.9 (C**12**), 129.7 (C**13** or C**15**), 129.2 (C**16**), 127.9 (C**14**), 126.7 (C**15** or C**13**), 125.8 (C**7**), 125.1 (C**8**), 123.4 (C**6**), 121.4 (C**9**), 82.3 (C**18**), 57.7 (C**3**), 42.9 (C**2**), 27.7 (C**19**), 21.0 (C**20**), 18.0 (C**1**). HRMS (ESI$^+$) calculated for $C_{22}H_{25}ClNO_2S$ $[M+H]^+$ = 402.1289, found 402.1290.

SFC conditions: CHIRALPACK SC (25 cm), 95:5 CO_2: *i*-PrOH, 2.0 mL/min, 250 nm; *Retention times*: t_{minor} = 5.4 min, t_{major} = 6.9 min.

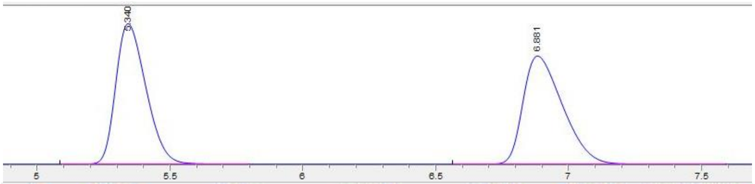

#	Time	Area	Height	Width	Area%	Symmetry
1	5.34	12703.1	1611.3	0.122	49.876	0.656
2	6.881	12766.1	1238.9	0.1602	50.124	0.556

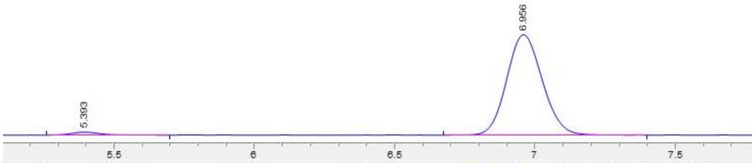

#	Time	Area	Height	Width	Area%	Symmetry
1	5.393	26.3	3.7	0.1069	2.317	0.818
2	6.956	1107.5	125.3	0.1391	97.683	0.858

(2*S*,3*S*)-2-(Benzo[*d*]thiazol-2-yl)-3-(2-chlorophenyl)-2-methylbutan-1-ol

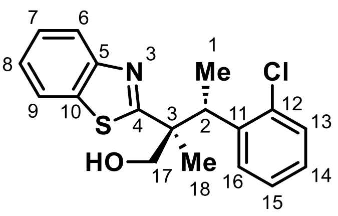

A solution of *tert*-Butyl (2*S*,3*S*)-2-(benzo[*d*]thiazol-2-yl)-3-(2-chlorophenyl)-2-methylbutanoate (80.4 mg, 0.20 mmol) in dry THF (1.00 mL) was added $LiAlH_4$ (2.0 M in THF, 0.1 mL, 0.20 mmol) dropwise

at 0 °C. The reaction mixture was stirred at 0 °C for 30 min. The reaction was quenched with water (1 mL). The reaction mixture was extracted with ethyl acetate (3 × 10 mL). The combined organic phase was dried by anhydrous $MgSO_4$, filtered and concentrated under reduced pressure. The residue was purified by flash column chromatography (Hexane/EtOAc = 9/1) to give the title compounds (62.6 mg, 94%, 97.5:2.5 e.r.) as a colorless oil. $[\alpha]_D^{24}$ = +30.7 (*c* 0.5, CH_2Cl_2). IR (thin film) ν_{max}/cm^{-1}: 3404, 2976, 2878, 1735, 1495, 1436, 755. ^1H NMR (400 MHz, $CDCl_3$) δ_H = 8.05 (d, *J* = 8.0 Hz, 1H, C**6**-H), 7.88 (d, *J* = 8.0 Hz, 1H, C**9**-H), 7.52 – 7.46 (m, 1H, C**7**-H), 7.42 – 7.36 (m, 2H, C**8**-H + C**13**-H or C**15**-H), 7.18 – 7.13 (m, 3H, C**14**-H + C**16**-H + C**15**-H or C**13**-H), 4.24 – 4.15 (m, 1H, C**17**-H), 4.13 (q, *J* = 7.2 Hz, 1H, C**2**-H), 3.60 (dd, *J* = 11.0, 6.7 Hz, 1H, C**17**-H'), 2.61 (s, 1H, OH), 1.54 (s, 3H, C**18**-H_3), 1.26 (d, *J* = 7.2 Hz, 3H, C**1**-H_3); ^{13}C NMR (100 MHz, $CDCl_3$) δ_C = 177.6 (C**4**), 153.0 (C**5**), 140.4 (C**11**), 134.9 (C**10**), 134.8 (C**12**), 129.9 (C**13** or C**15**), 129.8 (C**16**), 127.9 (C**14**), 126.6 (C**15** or C**13**), 126.1 (C**7**), 125.0 (C**8**), 123.2 (C**6**), 121.6 (C**9**), 70.4 (C**17**), 51.2 (C**3**), 41.3 (C**2**), 19.1 (C**18**), 17.3 (C**1**). HRMS (ESI$^+$) calculated for $C_{18}H_{19}ClNOS$ $[M+H]^+$ = 354.0696, found 354.0689.

SFC conditions: CHIRALPACK SC (25 cm), 70:30 CO_2:MeOH, 2.0 mL/min, 250 nm; *Retention times*: t_{minor} = 3.1 min, t_{major} = 3.9 min.

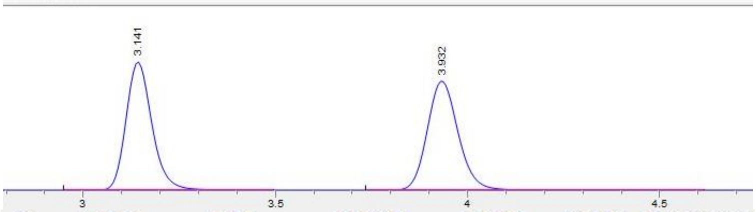

#	Time	Area	Height	Width	Area%	Symmetry
1	3.141	9978.3	2128.2	0.0727	49.690	0.785
2	3.932	10102.7	1811.6	0.0851	50.310	0.834

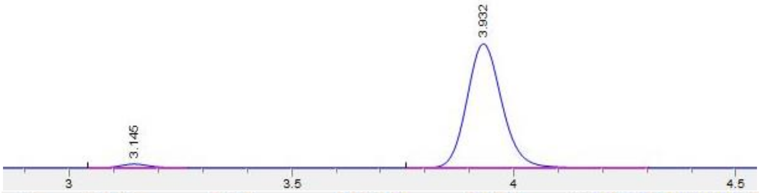

#	Time	Area	Height	Width	Area%	Symmetry
1	3.145	153.6	35	0.0731	2.338	0.846
2	3.932	6417.5	1175.9	0.091	97.662	0.865

2-((3*S*,4*R*)-3,4-Dimethylchroman-3-yl)benzo[*d*]thiazole

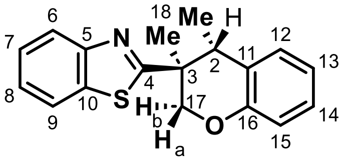

A flame-dried Schlenk was charged with $Pd(OAc)_2$ (2.25 mg, 0.01 mmol), (±)-2-(di-*t*-butylphosphino)-1,1'-binaphthyl (4.38 mg, 0.01 mmol) and Cs_2CO_3 (48.9 mg, 0.15 mmol). The Schlenk tube was

evacuated and backfilled with N_2 (3 times), and then a solution of (2*S*,3*S*)-2-(benzo[*d*]thiazol-2-yl)-3-(2-chlorophenyl)-2-methylbutan-1-ol (80.4 mg, 0.20 mmol) in dry toluene (0.40 mL) was added. The reaction mixture was stirred at 70 °C for 24 h in a heating plate. Purification by flash column chromatography (Hexane/EtOAc = 100/1 to 19/1) afforded the title compounds (20.4 mg, 69%, 97.5:2.5 e.r.) as a yellow solid. m.p. 59 – 60 °C (EtOAc/hexane). $[\alpha]_D^{24}$ = –55.8 (*c* 0.5, CH_2Cl_2). IR (thin film) ν_{max}/cm^{-1}: 3034, 2969, 2871, 1580, 1488, 1227, 752. ^1H NMR (400 MHz, $CDCl_3$) δ_H = 8.03 (d, *J* = 8.1 Hz, 1H, C**6**-H), 7.87 (d, *J* = 8.0 Hz, 1H, C**9**-H), 7.51 – 7.45 (m, 1H, C**7**-H), 7.40 – 7.35 (m, 1H, C**8**-H), 7.29 – 7.24 (m, 1H, C**12**-H), 7.17 – 7.11 (m, 1H, C**14**-H), 6.97 – 6.91 (m, 1H, C**13**-H), 6.88 – 6.83 (m, 1H, C**15**-H), 4.45 (d, *J* = 10.8 Hz, 1H, C**17**-H_a), 4.17 (d, *J* = 10.8 Hz, 1H, C**17**-H_b), 3.79 (q, *J* = 7.0 Hz, 1H, C**2**-H), 1.53 (s, 3H, C**18**-H_3), 1.32 (d, *J* = 7.0 Hz, 3H, C**1**-H_3); ^{13}C NMR (101 MHz, $CDCl_3$) δ_C = 176.4 (C**4**), 153.4 (C**5**), 153.0 (C**16**), 134.9 (C**10**), 128.4 (C**12**), 127.6 (C**14**), 126.2 (C**7**), 126.2 (C**11**), 125.0 (C**8**), 123.1 (C**6**), 121.7 (C**9**), 121.1 (C**13**), 116.6 (C**15**), 73.6 (C**17**), 43.1 (C**3**), 39.4 (C**2**), 18.2 (C**18**), 16.0 (C**1**). HRMS (ESI^+) calculated for $C_{18}H_{18}NOS$ $[M+H]^+$ = 296.1104, found 296.1110.

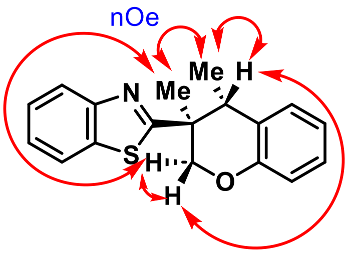

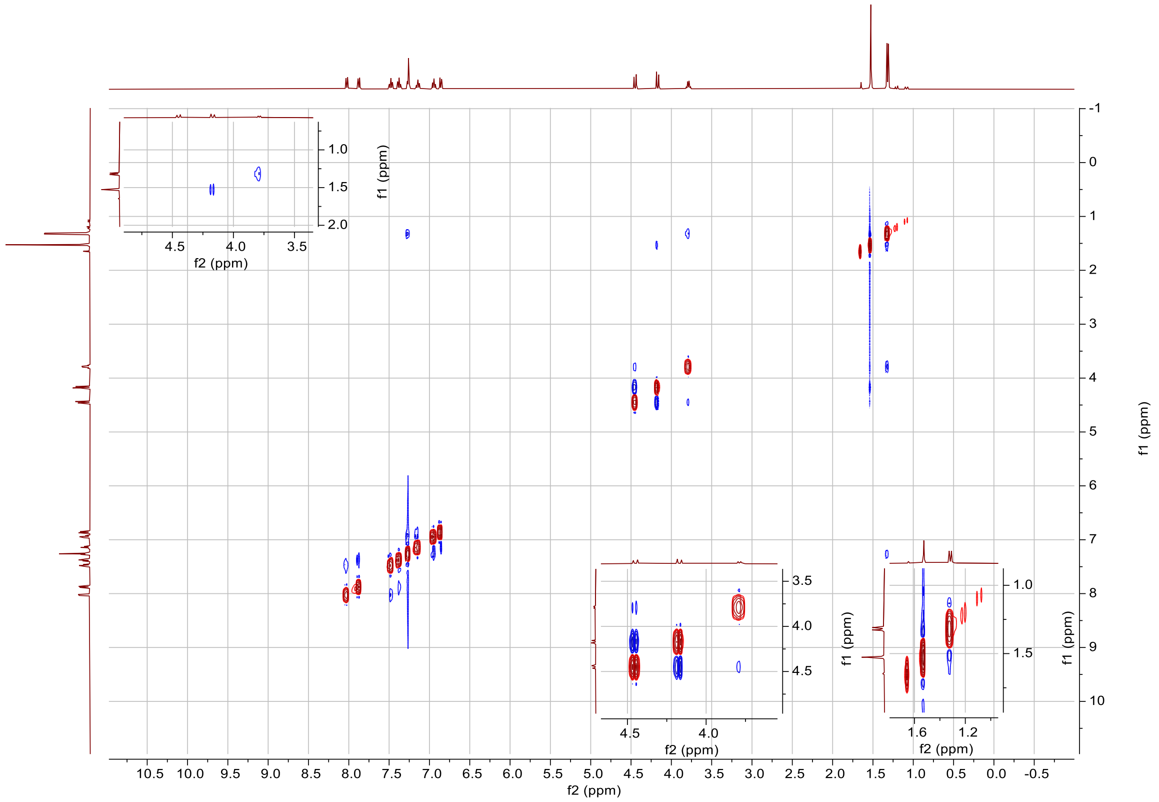

SFC conditions: CHIRALPACK SC (25 cm), 95:5 CO_2:MeOH, 2.0 mL/min, 250 nm; *Retention times*: t_{major} = 6.7 min, t_{minor} = 8.6 min.

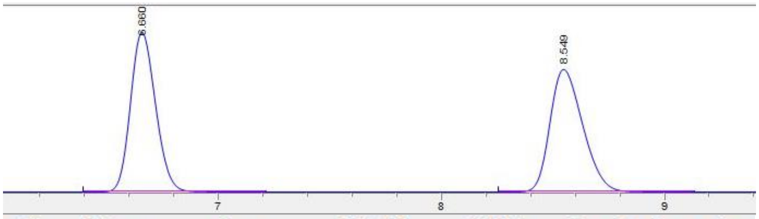

#	Time	Area	Height	Width	Area%	Symmetry
1	6.66	10195.3	1307.9	0.1229	49.943	0.806
2	8.549	10218.7	1002	0.159	50.057	0.683

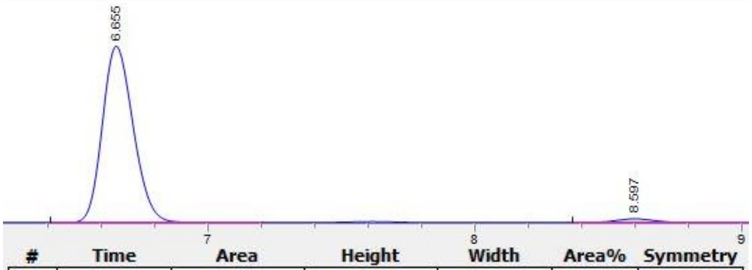

#	Time	Area	Height	Width	Area%	Symmetry
1	6.655	12679.9	1582.4	0.1255	97.501	0.78
2	8.597	325	32.9	0.1532	2.499	0.898

Mechanistic Studies

1. Positional effects of the *N*-center

a. The reaction of ethyl 2-(pyridin-2-yl)acetate and styrene

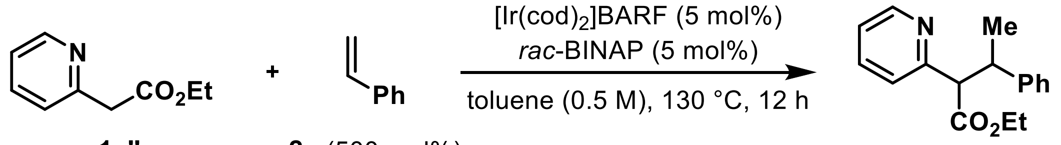

1a'' **2a** (500 mol%) **3aa''**, 76%, B:L>25:1, 1.6:1 d.r.

General procedure E: Ethyl 2-(pyridin-2-yl)acetate (16.5 mg, 0.10 mmol) and styrene (52.1 mg, 57.5 μL, 0.50 mmol) were employed with $[Ir(cod)_2]BARF$ (6.36 mg, 0.005 mmol) and *rac*-BINAP (3.11 mg, 0.005 mmol) in toluene (0.20 mL). The reaction was stirred at 130 °C for 12 h. Purification by flash column chromatography (Hexane/EtOAc = 10/1 to 4/1) afforded the title compounds (20.5 mg, 76%, B:L > 25:1, 1.6:1 d.r.) as colorless oils.

Ethyl 3-phenyl-2-(pyridin-2-yl)butanoate (3aa'')

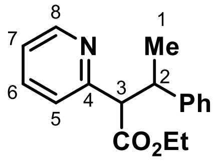

IR (thin film) ν_{max}/cm^{-1}: 2969, 2929, 1727, 1434, 1148, 761. Diastereomer 1: ^{1}H NMR (500 MHz, $CDCl_3$) δ_H = 8.44 – 8.38 (m, 1H, C**8**-H), 7.41 (ddd, *J* = 7.7, 7.7, 1.9 Hz, 1H, C**6**-H), 7.12 – 7.07 (m, 3H, C**7**-H + 2 × Ph ArCH), 7.06 – 7.00 (m, 3H, Ph ArCH), 6.98 (ddd, *J* = 7.7, 4.9, 1.2 Hz, 1H, C**5**-H), 4.26 – 4.13 (m, 2H, C$H_2$$CH_3$), 4.01 (d, *J* = 11.1 Hz, 1H, C**3**-H), 3.70 (dq, *J* = 11.1, 6.8 Hz, 1H, C**2**-H), 1.42

(d, J = 6.8 Hz, 3H, C**1**-H_3), 1.23 (t, J = 7.1 Hz, 3H, CH_2CH_3); ^{13}C NMR (125 MHz, $CDCl_3$) δ_C = 172.1 (C=O), 157.6 (C**4**), 149.6 (C**8**), 144.6 (Ph ArC), 136.9 (C**6**), 128.5 (Ph ArCH), 127.6 (Ph ArCH), 126.7 (Ph ArCH), 123.3 (C**5**), 122.6 (C**7**), 61.7 (CH_2CH_3), 60.6 (C**3**), 43.1 (C**2**), 20.2 (C**1**), 13.9 (CH_2CH_3). Diastereomer 2: ^{1}H NMR (500 MHz, $CDCl_3$) δ_H = 8.65 – 8.58 (m, 1H, C**8**-H), 7.69 (ddd, J = 7.8, 7.7, 1.8 Hz, 1H, C**6**-H), 7.53 (d, J = 7.8 Hz, 1H, C**5**-H), 7.36 – 7.28 (m, 4H, C**7**-H + 3 × Ph ArCH), 7.24 – 7.17 (m, 2H, Ph ArCH), 4.03 (d, J = 11.4 Hz, 1H, C**3**-H), 3.90 – 3.77 (m, 2H, CH_2CH_3), 3.62 (dq, J = 11.4, 7.0 Hz, 1H, C**2**-H), 1.04 (d, J = 7.0 Hz, 3H, C**1**-H_3), 0.87 (t, J = 7.1 Hz, 3H, CH_2CH_3); ^{13}C NMR (125 MHz, $CDCl_3$) δ_C = 172.6 (C=O), 157.4 (C**4**), 149.2 (C**8**), 143.7 (Ph ArC), 136.2 (C**6**), 128.2 (Ph ArCH), 127.7 (Ph ArCH), 126.3 (Ph ArCH), 123.5 (C**5**), 122.0 (C**7**), 61.4 (CH_2CH_3), 61.1 (C**3**), 42.8 (C**2**), 21.0 (C**1**), 14.3 (CH_2CH_3). HRMS (CI$^+$) calculated for $C_{17}H_{20}NO_2$ [M+H]$^+$ = 270.1489, found 270.1496.

b. The reaction of ethyl 2-(pyridin-3-yl)acetate and styrene

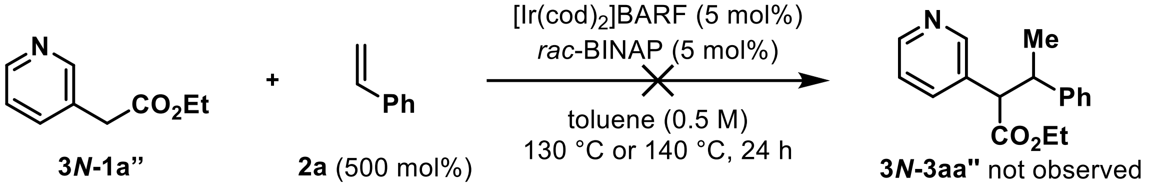

General procedure E: Ethyl 2-(pyridin-3-yl)acetate (16.5 mg, 0.10 mmol) and styrene (52.1 mg, 57.5 µL, 0.50 mmol) were employed with [Ir(cod)$_2$]BARF (6.36 mg, 0.005 mmol) and *rac*-BINAP (3.11 mg, 0.005 mmol) in toluene (0.20 mL). The reaction was stirred at 130 °C or 140 °C for 24 h. The reaction mixture was concentrated in *vacuo*. The ^{1}H NMR spectrum of the crude mixture indicated the results shown above.

c. The reaction of ethyl 2-(pyridin-4-yl)acetate and styrene

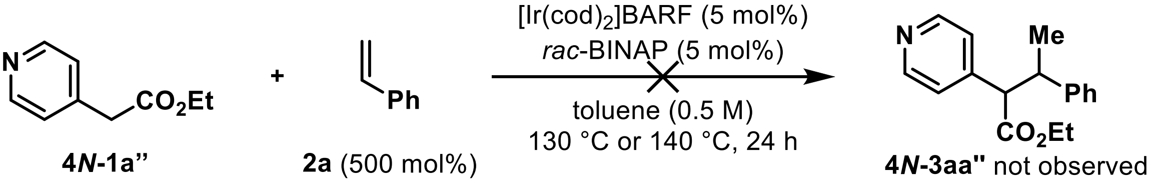

General procedure E: Ethyl 2-(pyridin-4-yl)acetate (16.5 mg, 0.10 mmol) and styrene (52.1 mg, 57.5 µL, 0.50 mmol) were employed with [Ir(cod)$_2$]BARF (6.36 mg, 0.005 mmol) and *rac*-BINAP (3.11 mg, 0.005 mmol) in toluene (0.20 mL). The reaction was stirred at 130 °C or 140 °C for 24 h. The reaction mixture was concentrated in *vacuo*. The ^{1}H NMR spectrum of the crude mixture indicated the results shown above.

2. Mechanistic studies based on *tert*-butyl 2-(benzo[*d*]thiazol-2-yl)acetate and 4-vinyl-1,1'- biphenyl

a. The reaction of *tert*-butyl 2-(benzo[*d*]thiazol-2-yl)acetate and 4-vinyl-1,1'-biphenyl

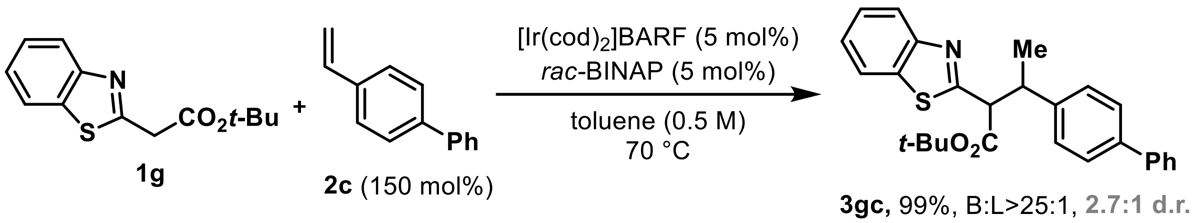

General procedure E: *tert*-Butyl 2-(benzo[*d*]thiazol-2-yl)acetate (74.7 mg, 0.30 mmol) and 4-vinyl-1,1'- biphenyl (82.0 mg, 0.45 mmol) were employed with $[Ir(cod)_2]BARF$ (19.1 mg, 0.015 mmol) and *rac*-BINAP (9.33 mg, 0.015 mmol) in toluene (0.60 mL). The reaction was stirred at 70 °C for 48 h. Purification by flash column chromatography (Hexane/EtOAc = 12/1 to 6/1) afforded the title compounds (127.8 mg, 99%, B:L > 25:1, 2.7:1 d.r.) as colorless solids.

***tert*-Butyl 3-([1,1'-biphenyl]-4-yl)-2-(benzo[*d*]thiazol-2-yl)butanoate (3gc)**

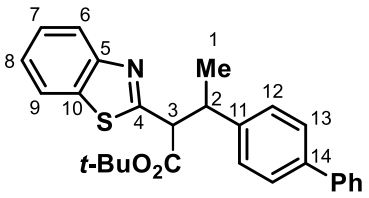

IR (thin film) ν_{max}/cm^{-1}: 2974, 2927, 1725, 1368, 1146, 760. Diastereomer 1: ^1H NMR (500 MHz, CD_2Cl_2) δ_H = 8.08 (d, J = 8.1 Hz, 1H, C**6**-H), 7.99 (d, J = 7.9 Hz, 1H, C**9**-H), 7.68 – 7.63 (m, 4H, 2 × C**12**-H + 2 × Ph ArCH), 7.57 – 7.52 (m, 1H, C**7**-H), 7.52 – 7.36 (m, 5H, C**8**-H + 2 × C**13**-H + 2 × Ph ArCH), 7.36 – 7.28 (m, 1H, Ph ArCH), 4.38 (d, J = 11.3 Hz, 1H, C**3**-H), 3.63 (dq, J = 11.3, 7.0 Hz, 1H, C**2**-H), 1.27 (d, J = 7.0 Hz, 3H, C**1**-H_3), 1.19 (s, 9H, $C(CH_3)_3$); ^{13}C NMR (125 MHz, CD_2Cl_2) δ_C = 169.8 (C=O), 168.3 (C**4**), 153.2 (C**5**), 143.3 (C**11**), 141.4 (C**14**), 140.4 (Ph ArC), 136.3 (C**10**), 129.3 (Ph ArCH), 128.8 (C**12**), 127.8 (Ph ArCH), 127.6 (Ph ArCH), 127.5 (C**13**), 126.5 (C**7**), 125.7 (C**8**), 123.6 (C**6**), 122.2 (C**9**), 82.5 ($C(CH_3)_3$), 60.3 (C**3**), 45.3 (C**2**), 27.8 ($C(CH_3)_3$), 20.7 (C**1**). Diastereomer 2: ^1H NMR (500 MHz, CD_2Cl_2) δ_H = 7.91 (d, J = 8.1 Hz, 1H, C**6**-H), 7.85 (d, J = 8.0 Hz, 1H, C**9**-H), 7.57 – 7.52 (m, 2H, C**7**-H + C**8**-H), 7.52 – 7.36 (m, 8H, 2 × C**12**-H + 2 × C**13**-H + 4 × Ph ArCH), 7.36 – 7.28 (m, 1H, Ph ArCH), 4.41 (d, J = 11.7 Hz, 1H, C**3**-H), 3.75 (dq, J = 11.7, 6.7 Hz, 1H, C**2**-H), 1.53 (s, 9H, $C(CH_3)_3$), 1.53 (d, J = 6.7 Hz, 3H, C**1**-H_3); ^{13}C NMR (125 MHz, CD_2Cl_2) δ_C = 170.2 (C=O), 168.0 (C**4**), 153.0 (C**5**), 142.8 (C**11**), 141.1 (C**14**), 139.8 (Ph ArC), 136.0 (C**10**), 129.2 (Ph ArCH), 128.6 (C**12**), 127.7 (Ph ArCH), 127.4 (Ph ArCH), 127.3 (C**13**), 126.3 (C**7**), 125.4 (C**8**), 123.4 (C**6**), 122.0 (C**9**), 83.0 ($C(CH_3)_3$), 59.8 (C**3**), 44.4 (C**2**), 28.2 ($C(CH_3)_3$), 21.3 (C**1**). HRMS (ESI^+) calculated for $C_{27}H_{27}NNaO_2S$ $[M+Na]^+$ = 452.1655, found 452.1654.

b. Deuterium exchange experiments

Deuterium incorporation was calculated by integration of 1H and/or 2D NMR spectra

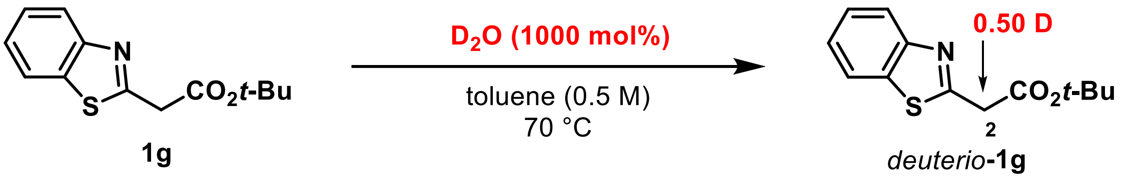

A Schlenk tube was charged with *tert*-butyl 2-(benzo[*d*]thiazol-2-yl)acetate (24.9 mg, 0.10 mmol), and evacuated/backfilled with N_2 (three cycles). Then D_2O (20.0 mg, 1.00 mmol) was added followed by toluene (0.20 mL). The tube was sealed and heated at 70 °C under stirring for 48 h. After cooling to room temperature, the solvent was removed under reduced pressure and the crude reaction mixture was analyzed by ^1H NMR spectroscopy using $CDCl_3$ as solvent.

^1H NMR-compound ***deuterio*-1g** (w/o Ir/BINAP)

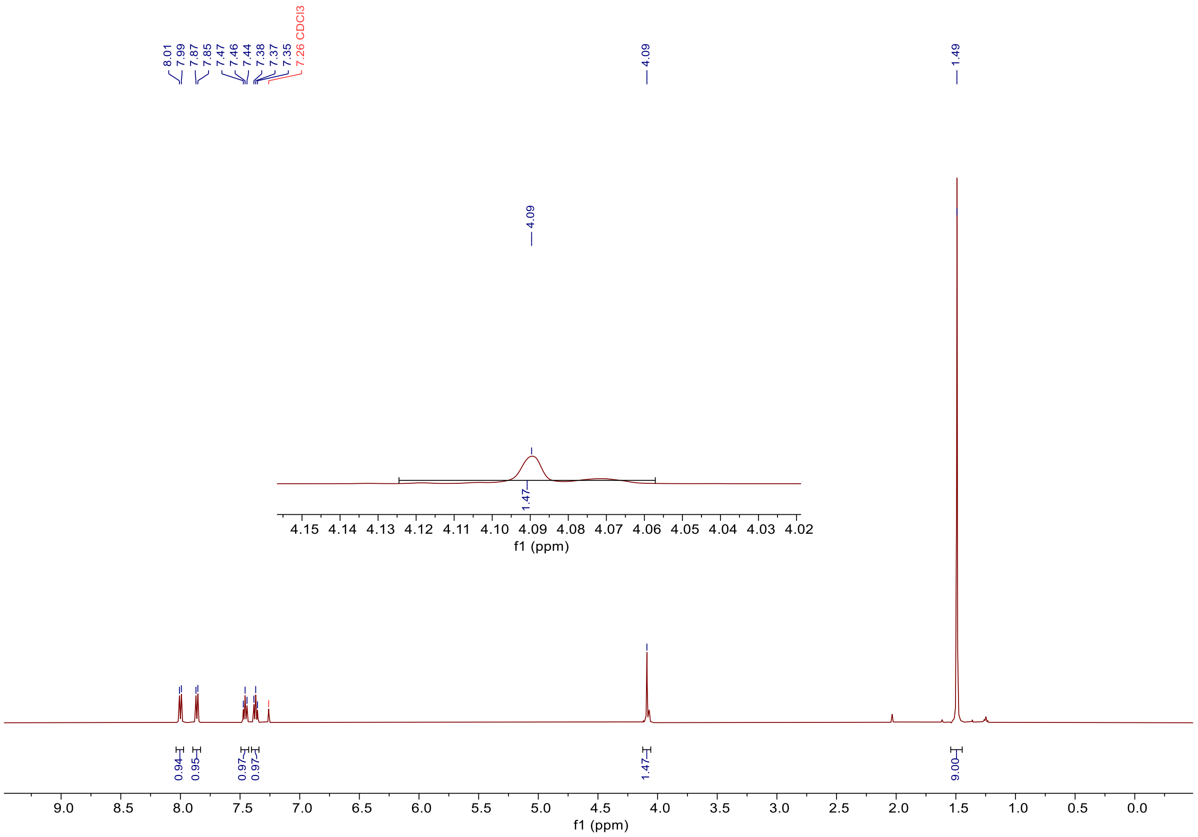

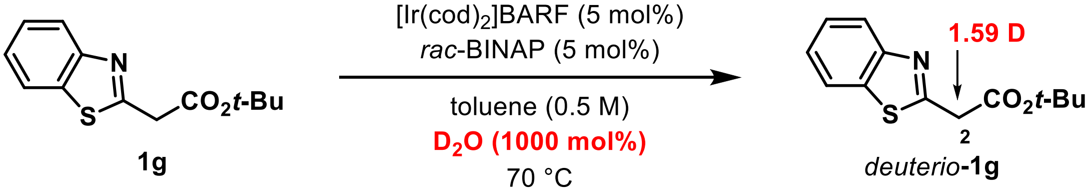

A Schlenk tube was charged with *tert*-butyl 2-(benzo[*d*]thiazol-2-yl)acetate (24.9 mg, 0.10 mmol), [Ir(cod)$_2$]BARF (6.36 mg, 0.005 mmol) and *rac*-BINAP (3.11 mg, 0.005 mmol). The Schlenk tube was evacuated/backfilled with N_2 (three cycles), then D_2O (20.0 mg, 1.00 mmol) was added followed by toluene (0.20 mL). The tube was sealed and heated at 70 °C under stirring for 48 h. After cooling to room temperature, the solvent was removed under reduced pressure and the crude reaction mixture was analyzed by ^1H NMR and ^2D NMR spectroscopy using CD_2Cl_2 as solvent.

^{1}H NMR-compound ***deuterio*-1g**

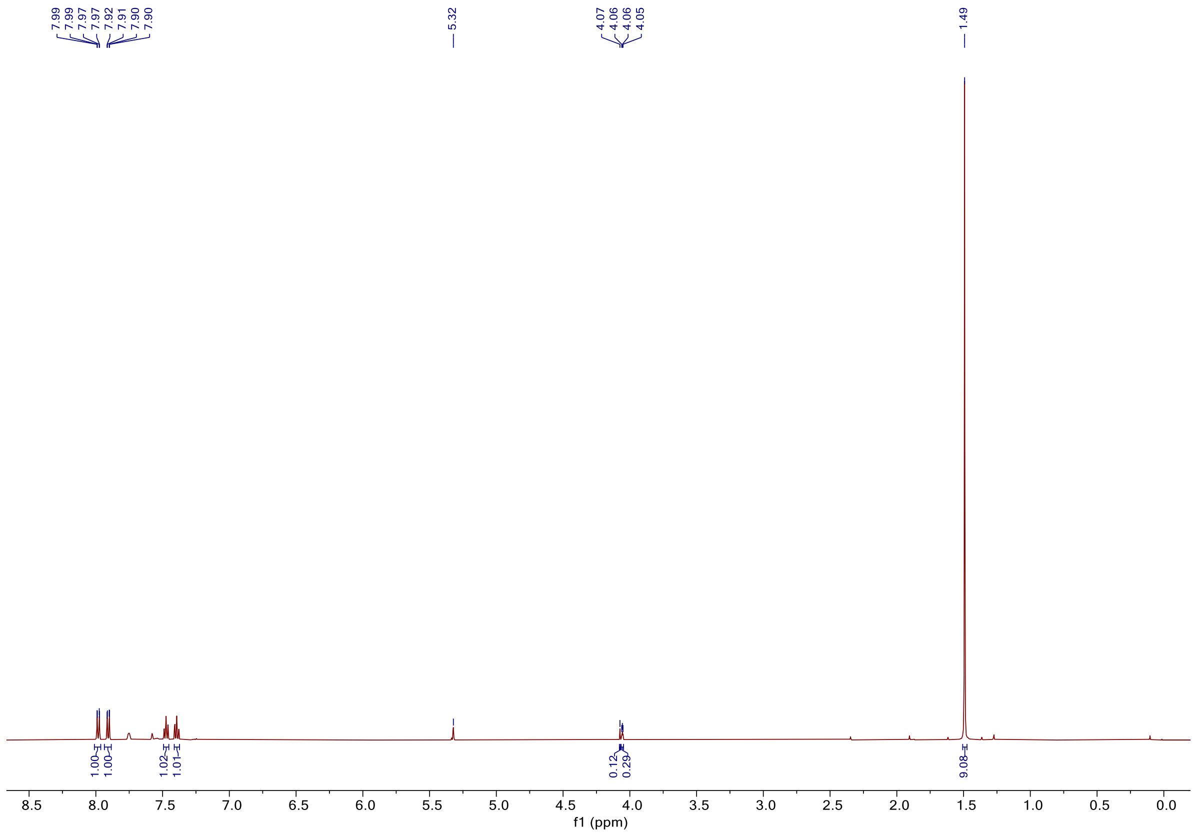

^{2}D NMR-compound ***deuterio*-1g**

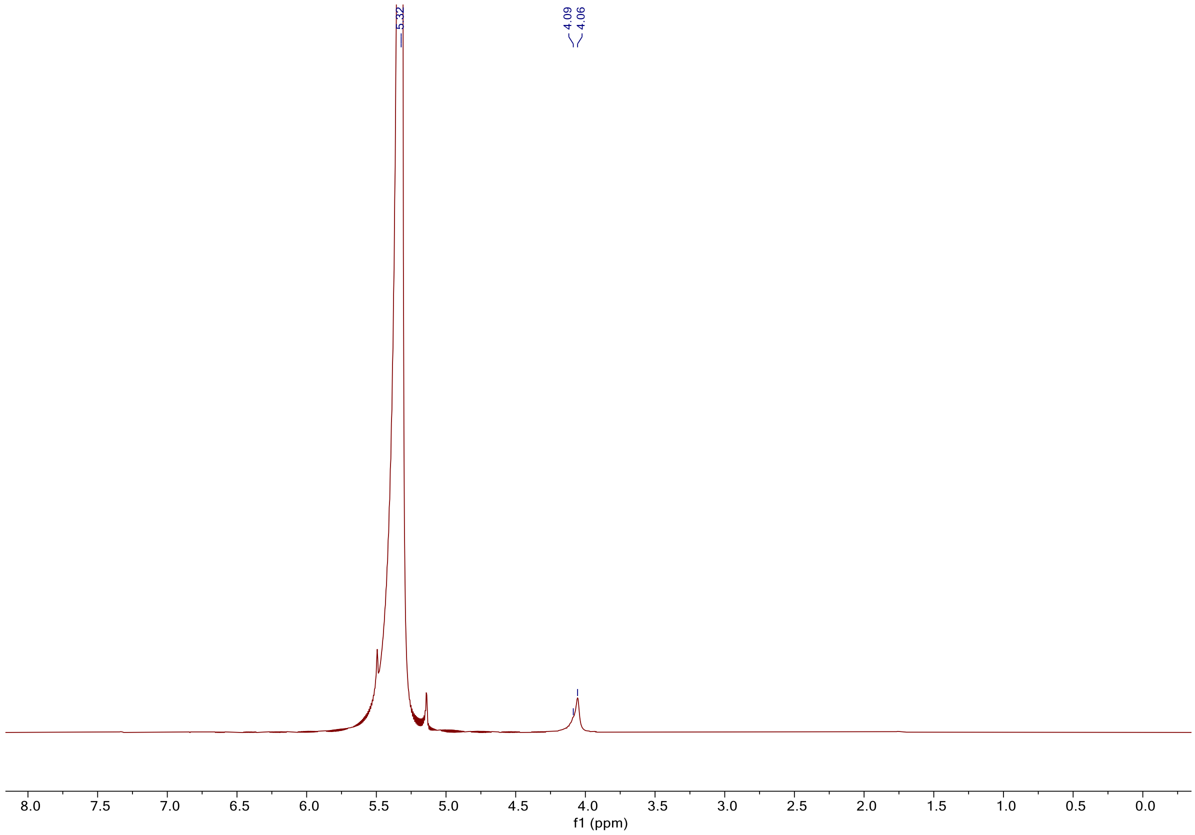

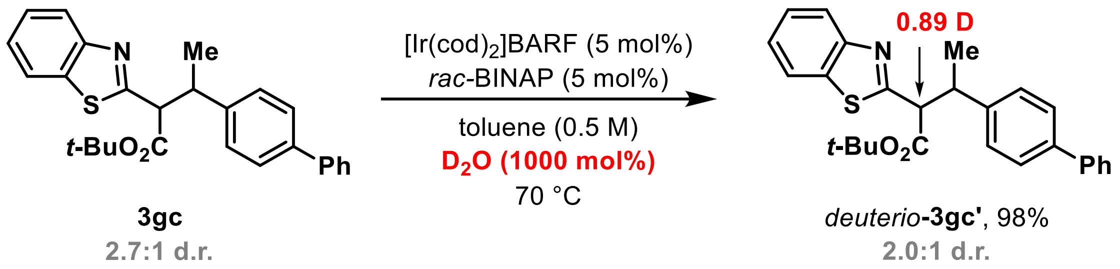

A Schlenk tube was charged with *tert*-Butyl 3-([1,1'-biphenyl]-4-yl)-2-(benzo[*d*]thiazol-2-yl)butanoate (21.5 mg, 0.05 mmol), [Ir(cod)$_2$]BARF (3.18 mg, 0.0025 mmol) and *rac*-BINAP (1.56 mg, 0.0025 mmol). The Schlenk tube was evacuated/backfilled with N_2 (three cycles), then D_2O (10.0 mg, 0.50 mmol) was added followed by toluene (0.10 mL). The tube was sealed and heated at 70 °C under stirring for 48 h. After cooling to room temperature, the solvent was removed under reduced pressure and the crude reaction mixture was analyzed by ^{1}H NMR and ^{2}D NMR spectroscopy using CD_2Cl_2 as solvent.

^{1}H NMR-compound **3gc**

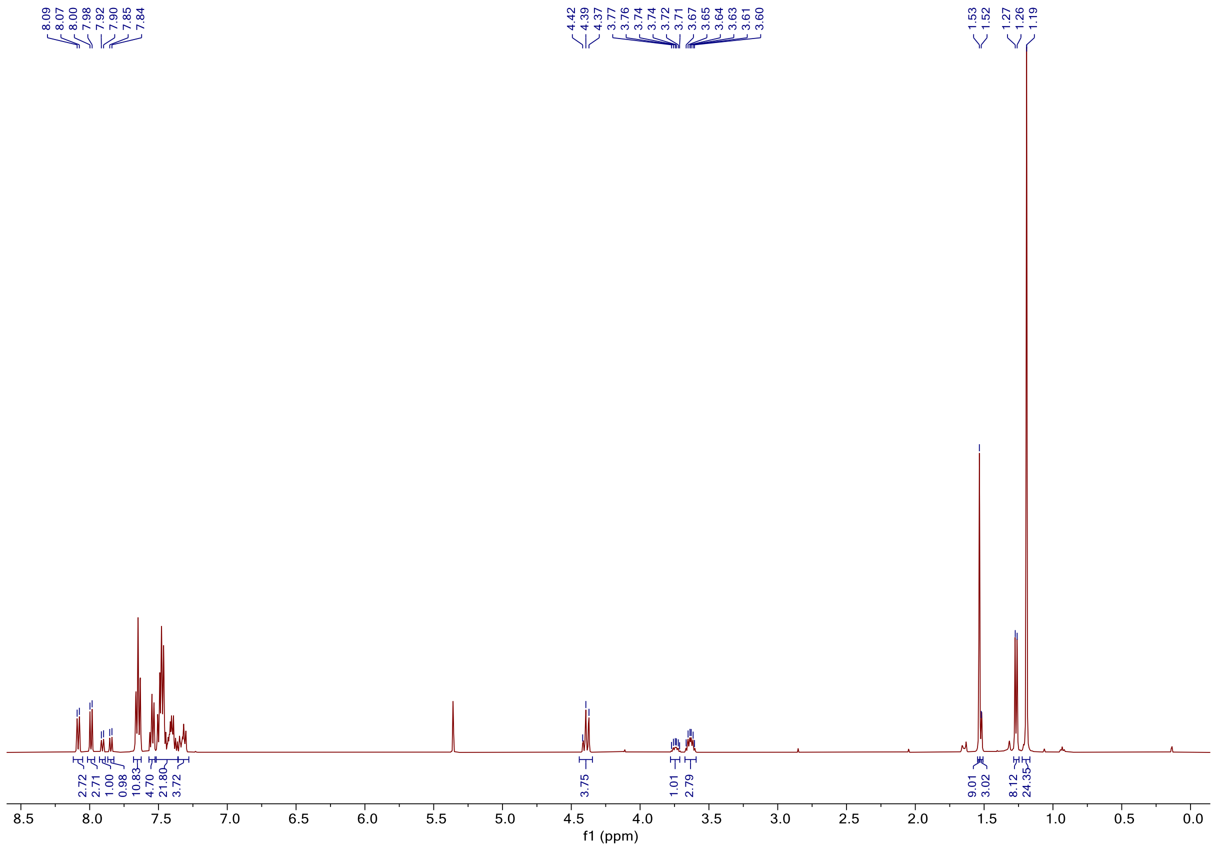

^{1}H NMR-compound ***deuterio*-3gc'**

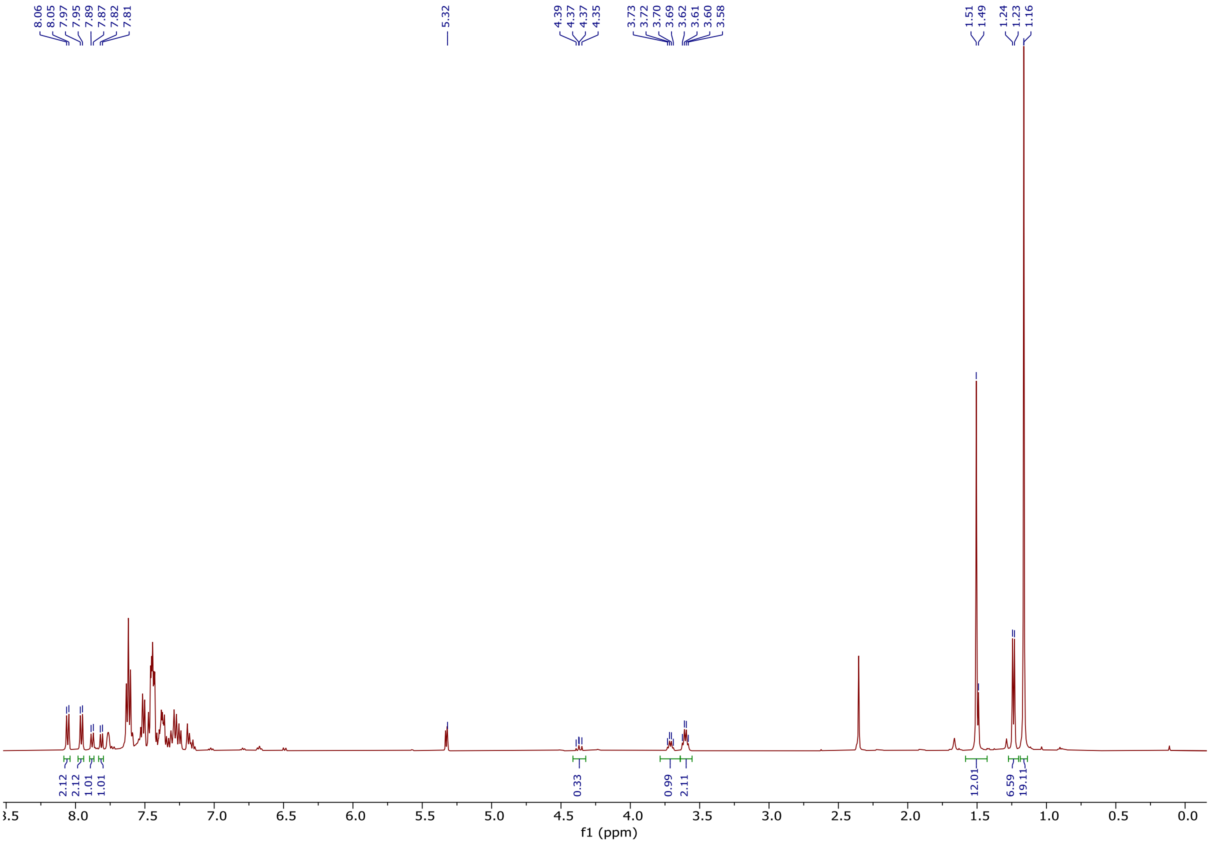

^{2}D NMR-compound ***deuterio*-3gc'**

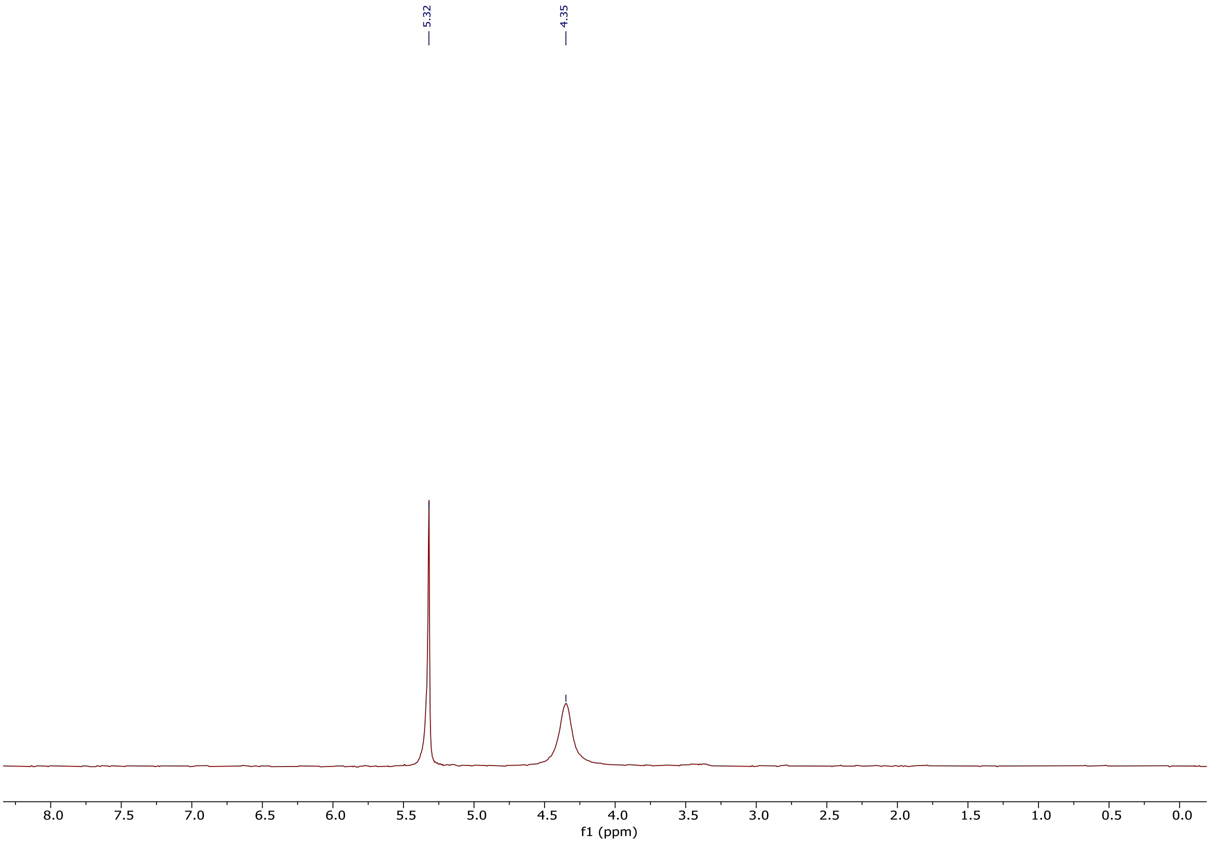

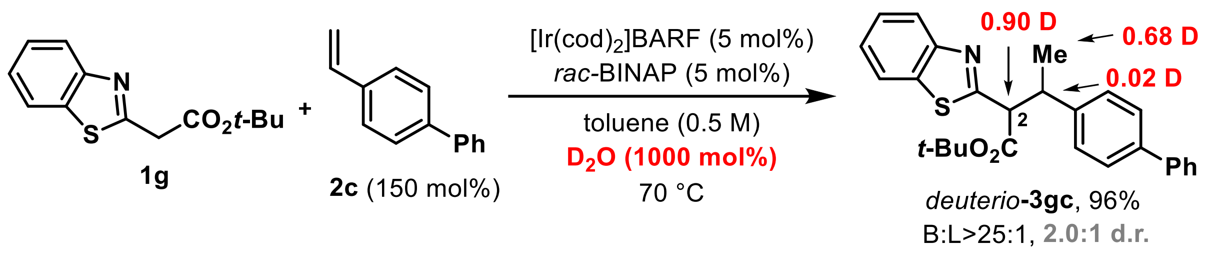

A Schlenk tube was charged with *tert*-butyl 2-(benzo[*d*]thiazol-2-yl)acetate (24.9 mg, 0.10 mmol), 4-vinyl-1,1'- biphenyl (27.3 mg, 0.15 mmol), [Ir(cod)$_2$]BARF (6.36 mg, 0.005 mmol) and *rac*-BINAP (3.11 mg, 0.005 mmol). The Schlenk tube was evacuated/backfilled with N_2 (three cycles), then D_2O (20.0 mg, 1.00 mmol) was added followed by toluene (0.20 mL). The tube was sealed and heated at 70 °C under stirring for 72 h. After cooling to room temperature, the solvent was removed under reduced pressure and the crude reaction mixture was purified by flash column chromatography (Hexane/EtOAc = 12/1 to 6/1) to give ***deuterio*-3gc** (42.1 mg, 96%, B:L > 25:1, 2.0:1 d.r.) as colorless solids. The obtained products were analyzed by ^{1}H NMR and ^{2}D NMR spectroscopy using CD_2Cl_2 as solvent.

^{1}H NMR-compound ***deuterio*-3gc**

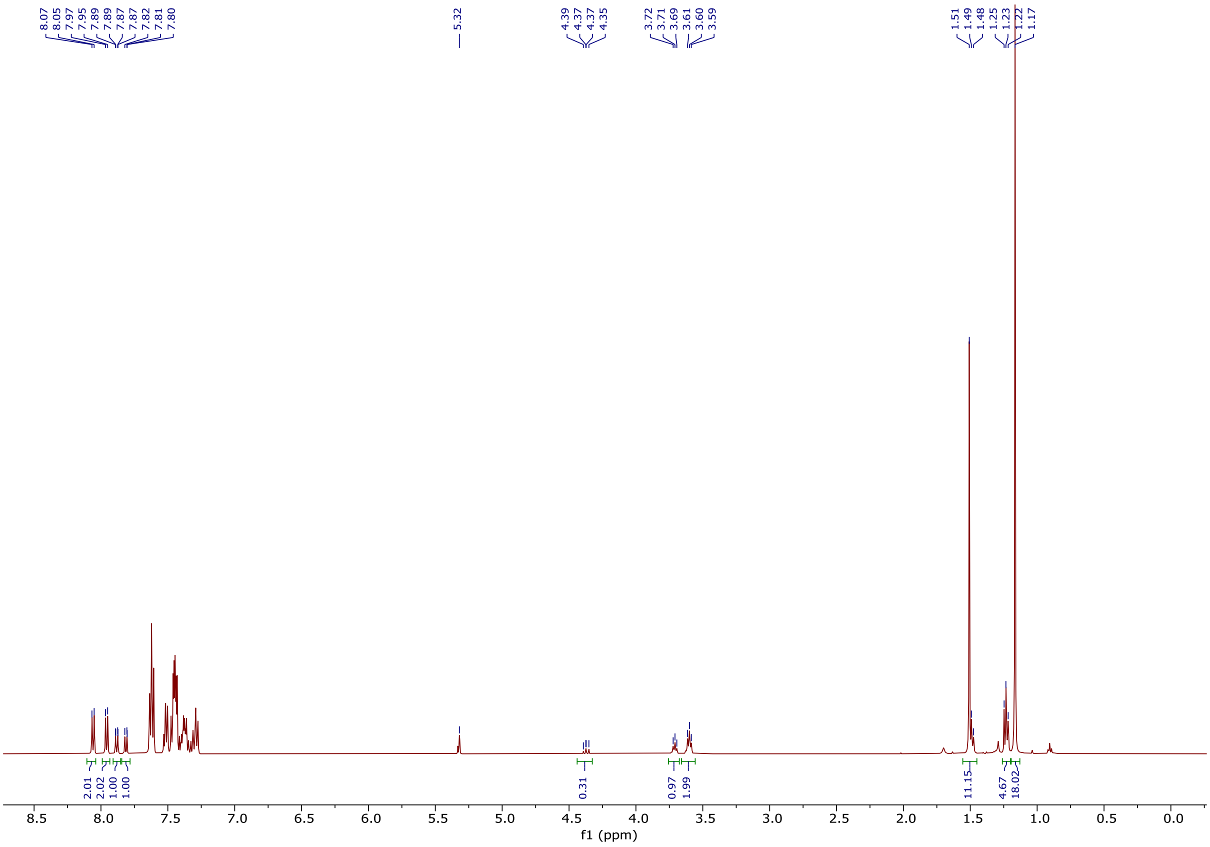

[2]D NMR-compound ***deuterio*-3gc**

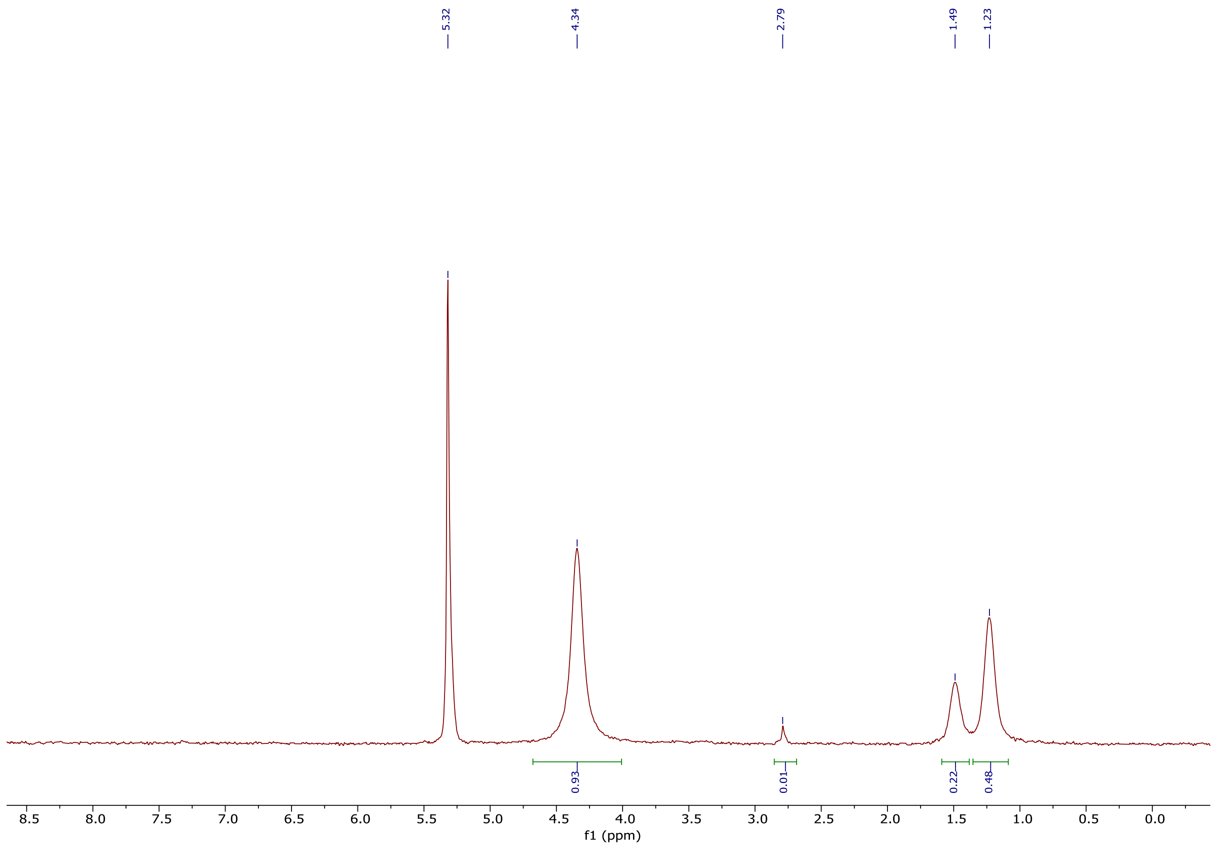

c. Deuterium labelling experiment: use of a labelled alkene partner

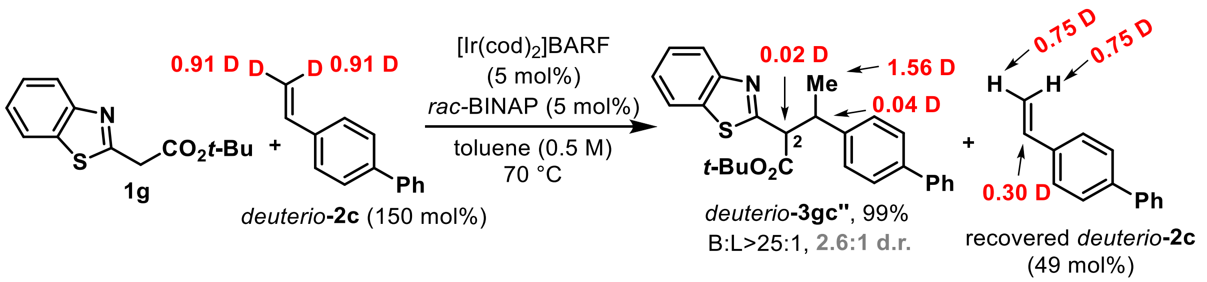

A Schlenk tube was charged with *tert*-butyl 2-(benzo[*d*]thiazol-2-yl)acetate (24.9 mg, 0.10 mmol), ***deuterio*-2c** (27.3 mg, 0.15 mmol), $[Ir(cod)_2]$BARF (6.36 mg, 0.005 mmol) and *rac*-BINAP (3.11 mg, 0.005 mmol). The Schlenk tube was evacuated/backfilled with N_2 (three cycles) followed by the adding of toluene (0.20 mL). The tube was sealed and heated at 70 °C under stirring for 48 h. After cooling to room temperature, the solvent was removed under reduced pressure and the crude reaction mixture was purified by flash column chromatography (Hexane) to give recovered ***deuterio*-2c** (9.00 mg, 0.049 mmol, 49 mol%) as a colorless solid, then flash column chromatography (Hexane/EtOAc = 12/1 to 6/1) to give ***deuterio*-3gc''** (42.4 mg, 99%, B:L > 25:1, 2.6:1 d.r.) as colorless solids. ***Deuterio*-2c**, ***deuterio*-3gc''** and recovered ***deuterio*-2c** were analyzed by ^{1}H NMR and/or ^{2}D NMR spectroscopy using CD_2Cl_2 as solvent.

^{1}H NMR-compound ***deuterio*-2c**

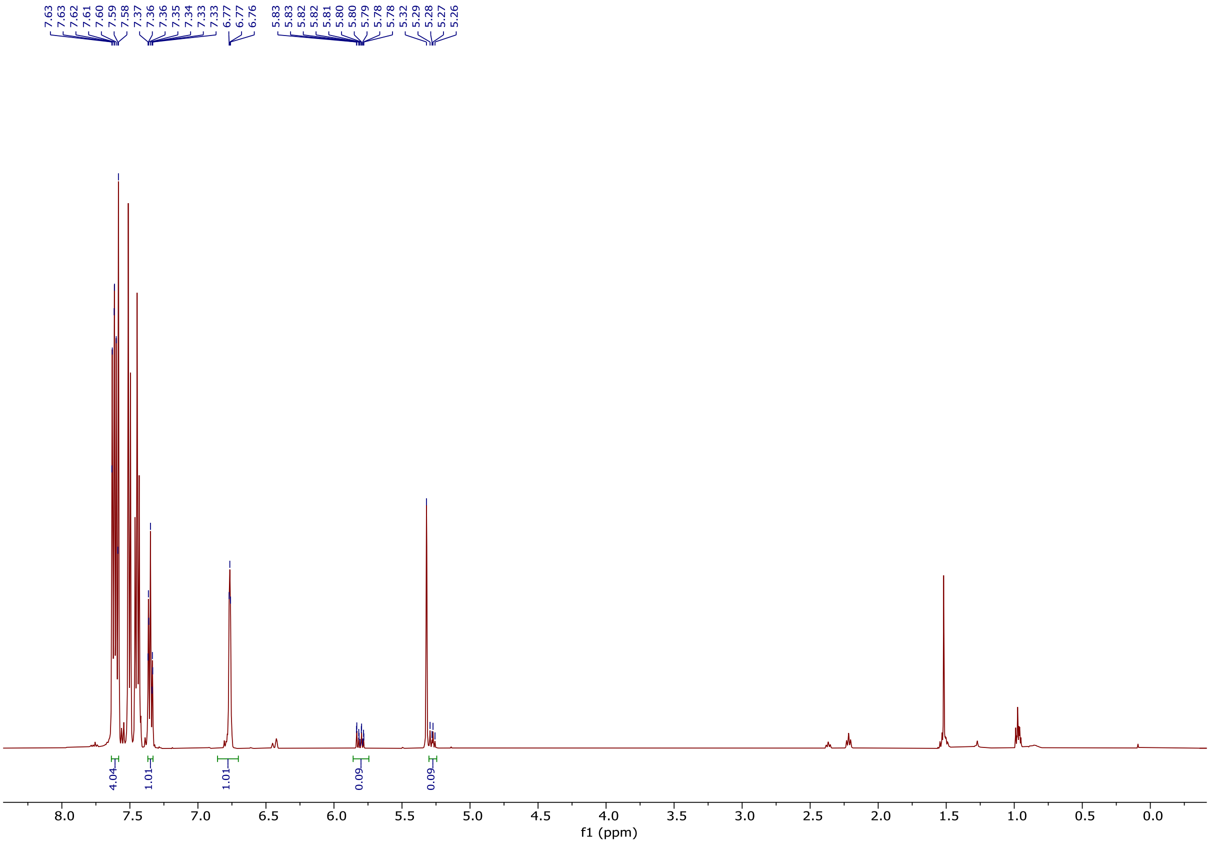

^{1}H NMR-compound recovered ***deuterio*-2c**

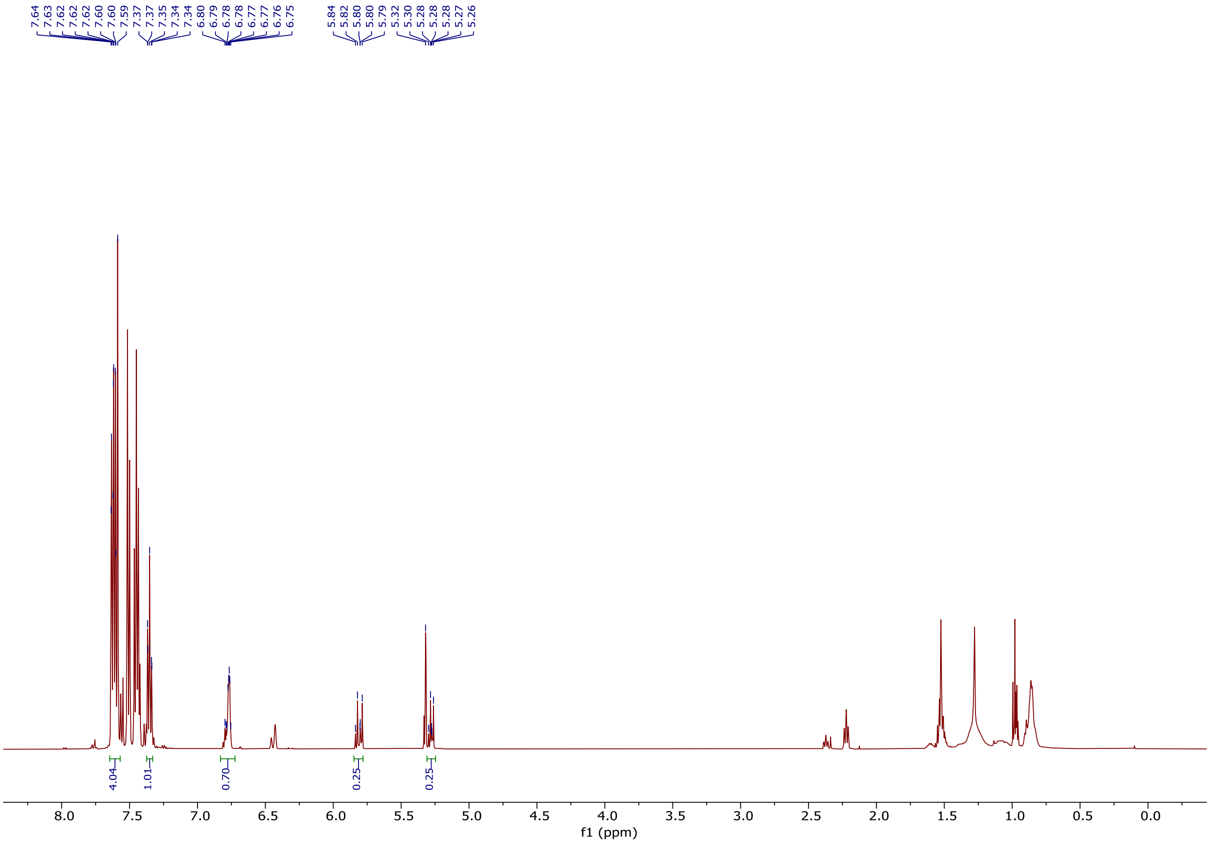

^{1}H NMR-compound ***deuterio*-3gc''**

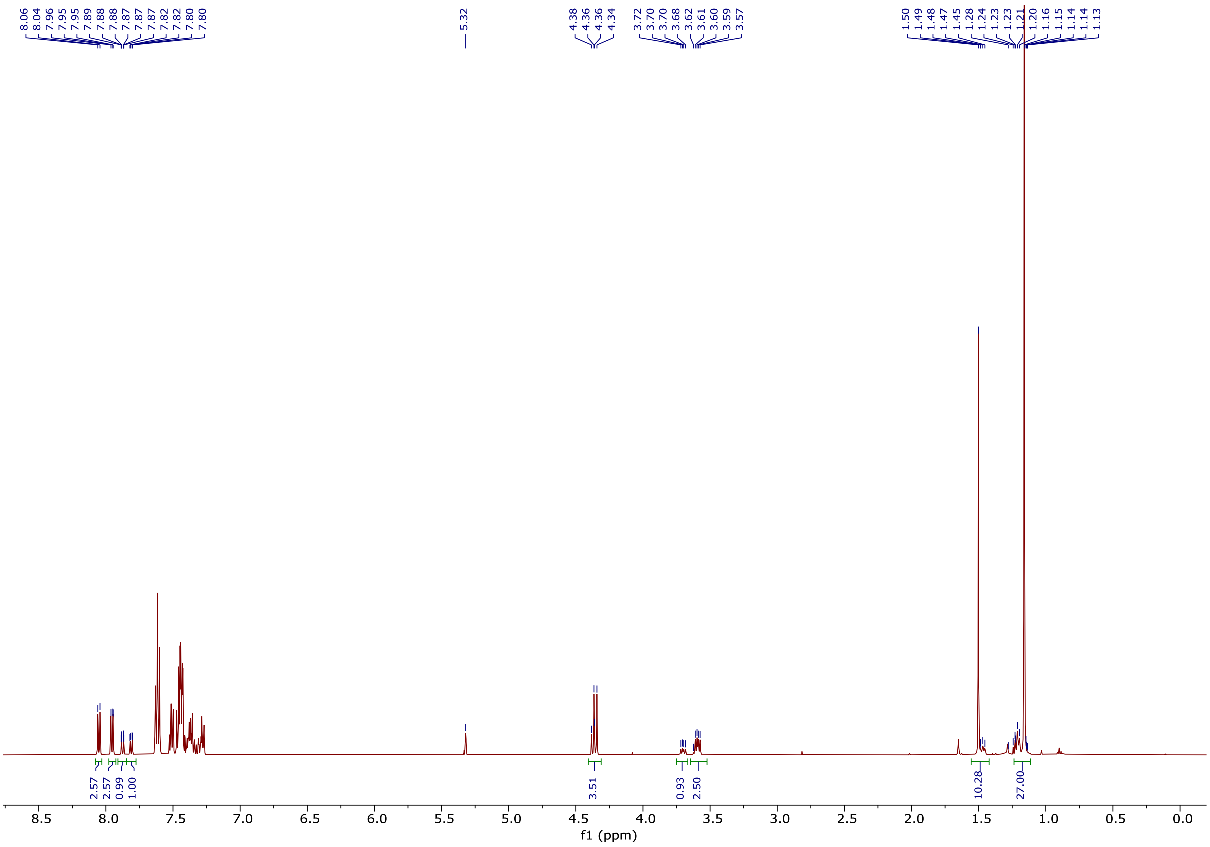

^{2}D NMR-compound ***deuterio*-3gc''**

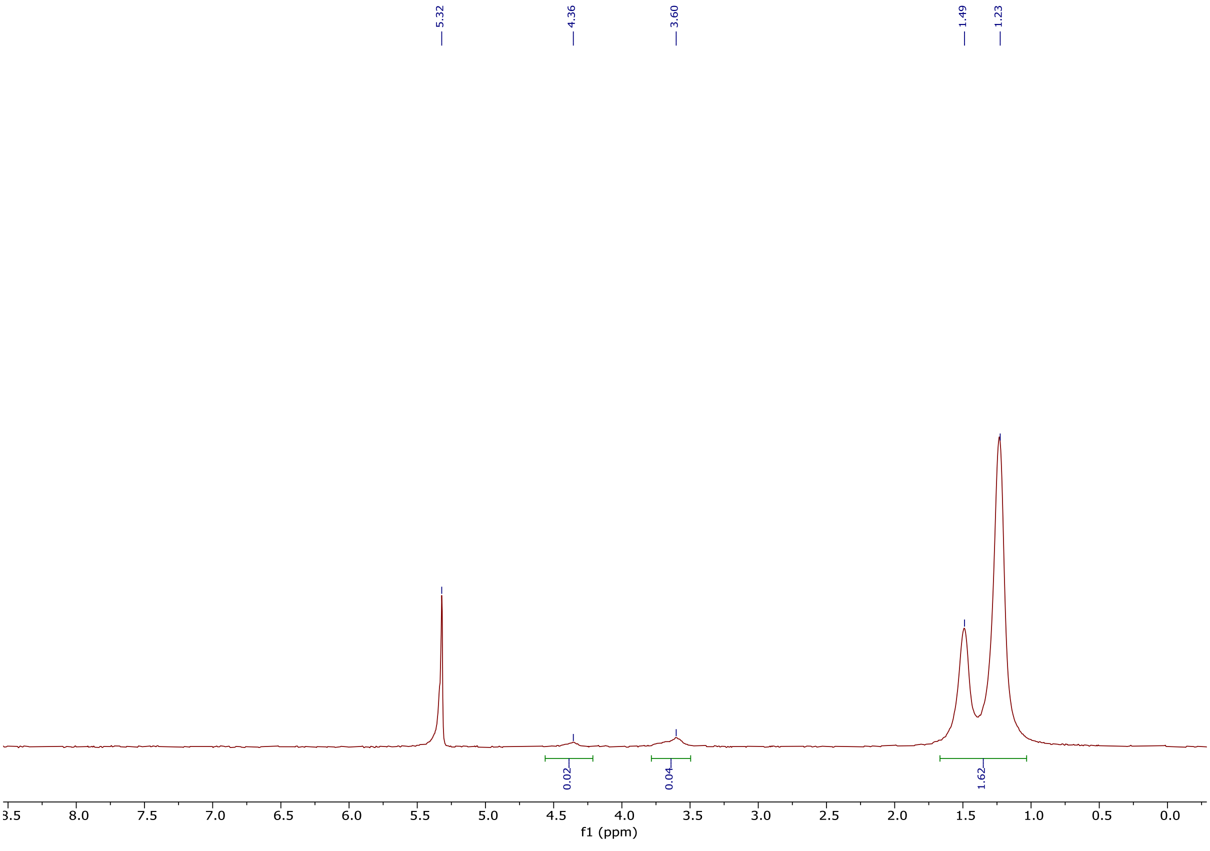

3. Graphical kinetic analysis

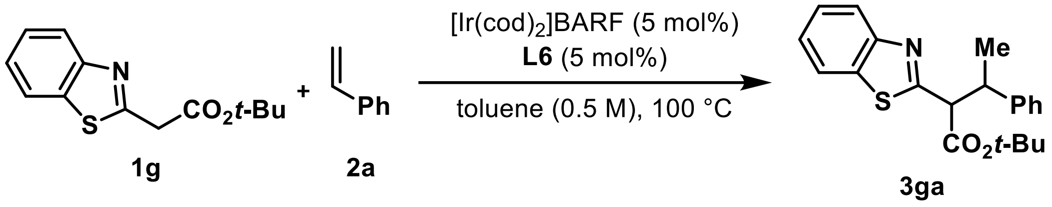

1g **2a** **3ga**

<u>**General procedure E**</u>: *The reaction kinetic was determined using* ***Burés**' normalized time scale method.*[10] Five parallel experiments of *tert*-butyl 2-(benzo[*d*]thiazol-2-yl)acetate (24.9 mg, 0.10 mmol) and styrene (15.6 mg, 17.2 µL, 0.15 mmol) were conducted using [Ir(cod)$_2$]BARF (6.36 mg, 0.005 mmol) and **L6** (1.91 mg, 0.005 mmol) in toluene (0.20 mL). The concentrations of *tert*-butyl 2-(benzo[*d*]-thiazol-2-yl)acetate at 0 h, 0.5 h, 0.75 h, 1 h and 1.5 h were determined by ^{1}H NMR analysis using 1,3,5-trimethoxybenzene as the internal standard. Another five parallel experiments used [Ir(cod)$_2$]BARF (3.82 mg, 0.003 mmol) and **L6** (1.15 mg, 0.003 mmol) in toluene (0.20 mL). The concentrations of *tert*-butyl 2-(benzo[*d*]-thiazol-2-yl)acetate at 0 h, 0.5 h, 1.5 h, 2 h and 3 h were determined by ^{1}H NMR analysis using 1,3,5-trimethoxybenzene as the internal standard:

For reactions using 5 mol% [Ir(cod)$_2$]BARF, concentration of catalyst is 0.022 mol/L

Time (h)	Concentration (mol/L)
0	0.47
0.5	0.425
1.5	0.317
2	0.262
3	0.0939

For reactions using 3 mol% [Ir(cod)$_2$]BARF, concentration of catalyst is 0.013 mol/L

Time (h)	Concentration (mol/L)
0	0.468
0.5	0.387
0.75	0.355
1	0.278
1.5	0.138

Graphical kinetic analysis: the order in catalyst ([Ir(cod)$_2$]BARF/**L6**) is approximately 1. This result should be treated with caution: each data point was determined from an individual experiment because the optimized reaction set-up uses sealed tubes, so errors are large. Further kinetic analyses are ongoing and will be reported as part of a full study.

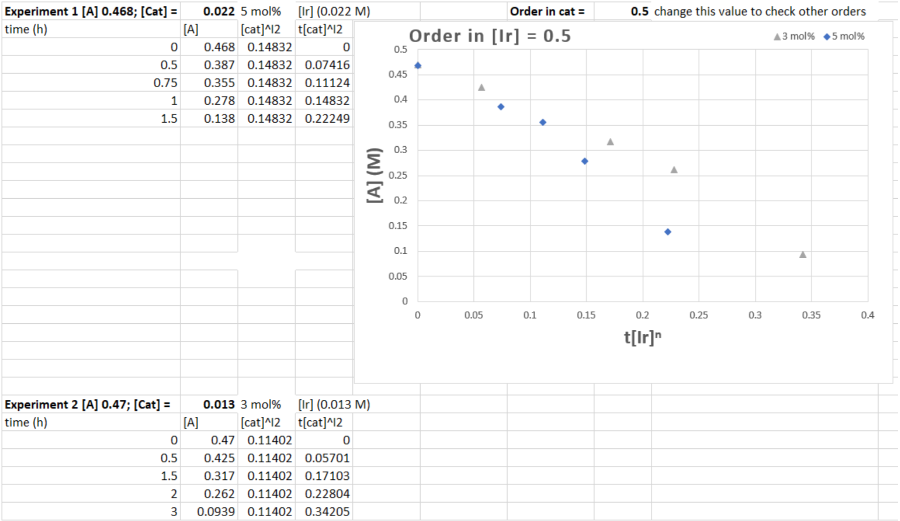

Experiment 1 [A] 0.468; [Cat] =	0.022	5 mol%	[Ir] (0.022 M)	Order in cat =	0.5	change this value to check other orders
time (h)	[A]	[cat]^I2	t[cat]^I2			
0	0.468	0.14832	0			
0.5	0.387	0.14832	0.07416			
0.75	0.355	0.14832	0.11124			
1	0.278	0.14832	0.14832			
1.5	0.138	0.14832	0.22249			

Experiment 2 [A] 0.47; [Cat] =	0.013	3 mol%	[Ir] (0.013 M)
time (h)	[A]	[cat]^I2	t[cat]^I2
0	0.47	0.11402	0
0.5	0.425	0.11402	0.05701
1.5	0.317	0.11402	0.17103
2	0.262	0.11402	0.22804
3	0.0939	0.11402	0.34205

Experiment 1 [A] 0.468; [Cat] =	0.022	5 mol%	[Ir] (0.022 M)	Order in cat =	1 change this value to check other orders
time (h)	[A]	[cat]^I2	t[cat]^I2		
0	0.468	0.022	0		
0.5	0.387	0.022	0.011		
0.75	0.355	0.022	0.0165		
1	0.278	0.022	0.022		
1.5	0.138	0.022	0.033		

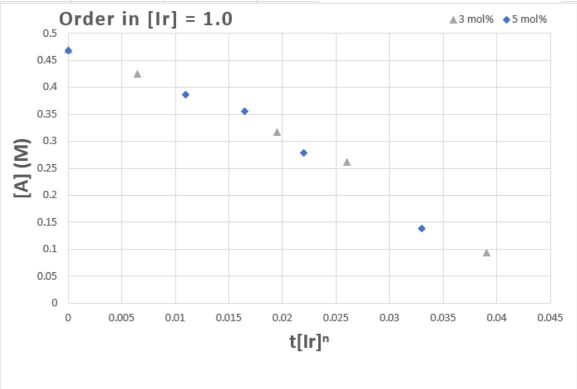

Experiment 2 [A] 0.47; [Cat] =	0.013	3 mol%	[Ir] (0.013 M)
time (h)	[A]	[cat]^I2	t[cat]^I2
0	0.47	0.013	0
0.5	0.425	0.013	0.0065
1.5	0.317	0.013	0.0195
2	0.262	0.013	0.026
3	0.0939	0.013	0.039

Experiment 1 [A] 0.468; [Cat] =	0.022	5 mol%	[Ir] (0.022 M)	Order in cat =	1.5 change this value to check other orders
time (h)	[A]	[cat]^I2	t[cat]^I2		
0	0.468	0.00326	0		
0.5	0.387	0.00326	0.00163		
0.75	0.355	0.00326	0.00245		
1	0.278	0.00326	0.00326		
1.5	0.138	0.00326	0.00489		

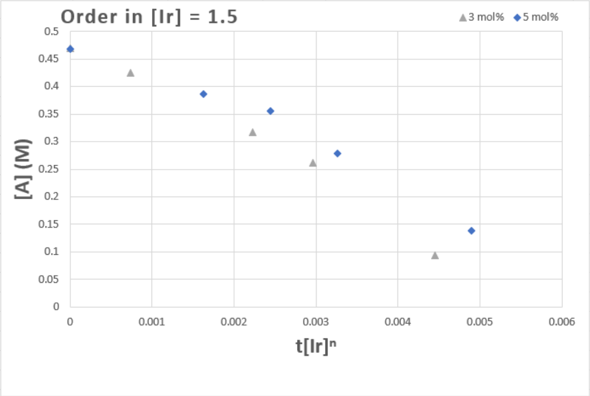

Experiment 2 [A] 0.47; [Cat] =	0.013	3 mol%	[Ir] (0.013 M)
time (h)	[A]	[cat]^I2	t[cat]^I2
0	0.47	0.00148	0
0.5	0.425	0.00148	0.00074
1.5	0.317	0.00148	0.00222
2	0.262	0.00148	0.00296
3	0.0939	0.00148	0.00445

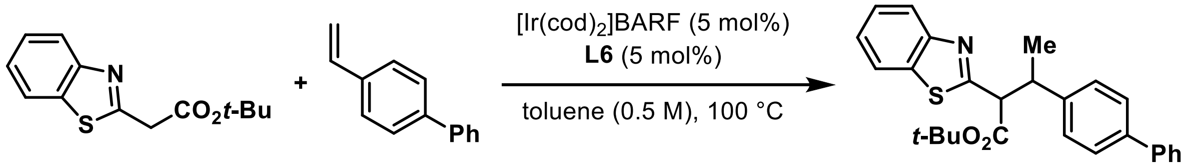

1g **2c** **3gc**

__General procedure E__: *The reaction kinetic was determined using **Burés**' normalized time scale method.*[10] Five parallel experiments of *tert*-butyl 2-(benzo[*d*]thiazol-2-yl)acetate (**1g**, 24.9 mg, 0.10 mmol) and 4-vinylbiphenyl (**2c**, 27.0 mg, 0.15 mmol) were conducted using [Ir(cod)$_2$]BARF (6.36 mg, 5.0 μmol) and **L6** (1.91 mg, 5.0 μmol) in toluene (0.20 mL). The concentrations of *tert*-butyl 2-(benzo[*d*]-thiazol-2-yl)acetate (**1g**) and 4-vinylbiphenyl (**2c**) at 0 min, 15 min, 30 min, 40 min, 60 min and 80 min were determined by ^{1}H NMR analysis using 1,3,5-trimethoxybenzene as the internal standard. Another five parallel experiments used *tert*-butyl 2-(benzo[*d*]thiazol-2-yl)acetate (**1g**, 18.7

mg, 75 μmol) and 4-vinylbiphenyl (**2c**, 27.0 mg, 0.15 mmol) were conducted using [Ir(cod)$_2$]BARF (6.36 mg, 5.0 μmol) and **L6** (1.91 mg, 5.0 μmol) in toluene (0.20 mL). The concentrations of *tert*-butyl 2-(benzo[*d*]-thiazol-2-yl)acetate (**1g**) and 4-vinylbiphenyl (**2c**) at 0 min, 20 min, 45 min, 55 min, 70 min and 80 min were determined by ^{1}H NMR analysis using 1,3,5-trimethoxybenzene as the internal standard. The results are listed as below:

For reactions using 0.1 mmol **1g**

Time (min)	Concentration (**1g**, mol/L)	Concentration (**2c**, mol/L)
0	0.435	0.652
15	0.390	0.620
30	0.383	0.610
40	0.311	0.555
60	0.224	0.469
80	0.184	0.443

For reactions using 0.075 mmol **1g**

Time (min)	Concentration (**1g**, mol/L)	Concentration (**2c**, mol/L)
0	0.326	0.652
20	0.311	0.626
45	0.278	0.600
55	0.233	0.564
70	0.186	0.512
80	0.160	0.476

Graphical kinetic analysis: there is a positive order in *tert*-butyl 2-(benzo[*d*]thiazol-2-yl)acetate (**1g**) (approximately 1). This result should be treated with caution: each data point was determined from an individual experiment because the optimized reaction set-up uses sealed tubes, so errors are large. Further kinetic analyses are ongoing and will be reported as part of a full study.

		Experiment 1				Experiment 2			
order in 1g	0.5	t (min)	$\Sigma[\mathbf{1g}]^n\Delta t$	[**1g**] (M)	[**2c**] (M)	t (min)	$\Sigma[\mathbf{1g}]^n\Delta t$	[**1g**] (M)	[**2c**] (M)
		0.00	0.00	0.435	0.652	0.00	0.00	0.326	0.652
		15.00	9.63	0.390	0.620	20.00	11.29	0.311	0.626
		30.00	18.96	0.383	0.610	45.00	24.85	0.278	0.600
		40.00	24.85	0.311	0.555	55.00	29.91	0.233	0.564
		60.00	35.19	0.224	0.469	70.00	36.77	0.186	0.512
		80.00	44.23	0.184	0.443	80.00	40.93	0.160	0.476

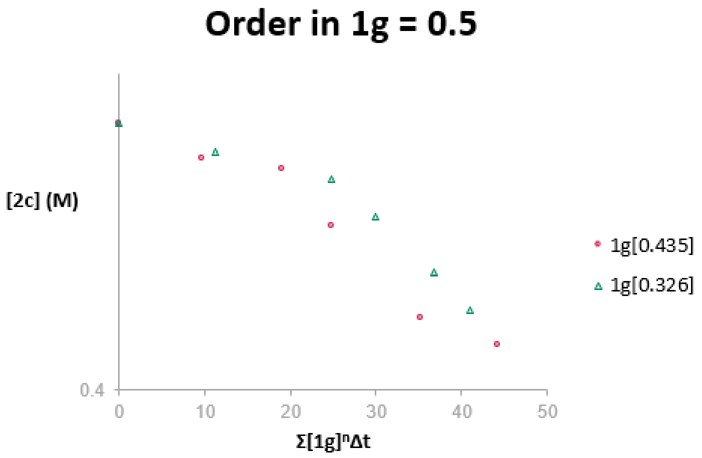

[1g]o	0.435 M
[2c]o	0.652 M
[Ir]o	0.0217 M
[1g]o	0.326 M
[2c]o	0.652 M
[Ir]o	0.0217 M

		Experiment 1				Experiment 2			
order in 1g	1	t (min)	Σ[**1g**]^{n}Δt	[**1g**] (M)	[**2c**] (M)	t (min)	Σ[**1g**]^{n}Δt	[**1g**] (M)	[**2c**] (M)
		0.00	0.00	0.435	0.652	0.00	0.00	0.326	0.652
		15.00	6.19	0.390	0.620	20.00	6.37	0.311	0.626
		30.00	11.99	0.383	0.610	45.00	13.73	0.278	0.600
		40.00	15.46	0.311	0.555	55.00	16.29	0.233	0.564
		60.00	20.81	0.224	0.469	70.00	19.43	0.186	0.512
		80.00	24.89	0.184	0.443	80.00	21.16	0.160	0.476

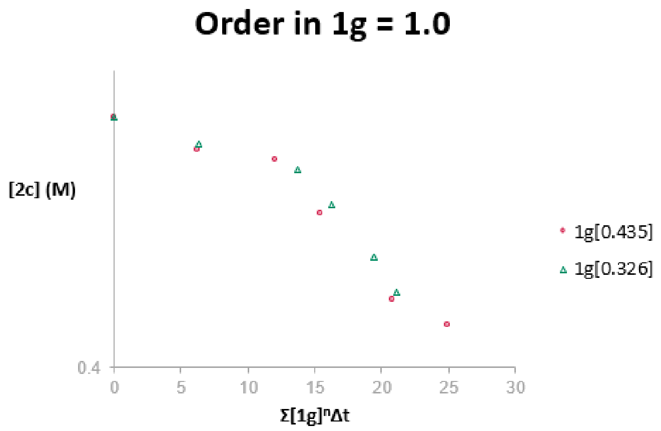

[1g]o	0.435 M
[2c]o	0.652 M
[Ir]o	0.0217 M
[1g]o	0.326 M
[2c]o	0.652 M
[Ir]o	0.0217 M

		Experiment 1				Experiment 2			
order in 1g	1.5	t (min)	Σ[**1g**]^{n}Δt	[**1g**] (M)	[**2c**] (M)	t (min)	Σ[**1g**]^{n}Δt	[**1g**] (M)	[**2c**] (M)
		0.00	0.00	0.435	0.652	0.00	0.00	0.326	0.652
		15.00	3.97	0.390	0.620	20.00	3.59	0.311	0.626
		30.00	7.58	0.383	0.610	45.00	7.59	0.278	0.600
		40.00	9.62	0.311	0.555	55.00	8.88	0.233	0.564
		60.00	12.39	0.224	0.469	70.00	10.32	0.186	0.512
		80.00	14.23	0.184	0.443	80.00	11.04	0.160	0.476

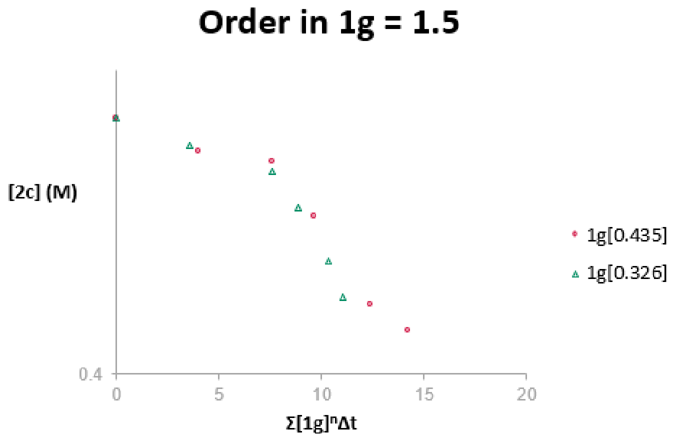

[1g]o	0.435 M
[2c]o	0.652 M
[Ir]o	0.0217 M
[1g]o	0.326 M
[2c]o	0.652 M
[Ir]o	0.0217 M

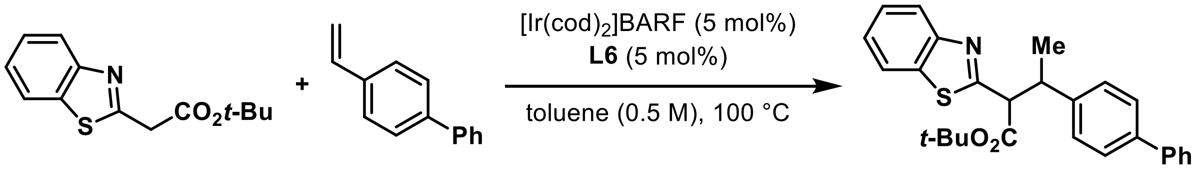

1g **2c** **3gc**

__**General procedure E**__: *The reaction kinetic was determined using **Burés**' normalized time scale method.*[10] Five parallel experiments of *tert*-butyl 2-(benzo[*d*]thiazol-2-yl)acetate (**1g**, 24.9 mg, 0.10

mmol) and 4-vinylbiphenyl (**2c**, 18.0 mg, 0.10 mmol) were conducted using [Ir(cod)$_2$]BARF (6.36 mg, 5.0 μmol) and **L6** (1.91 mg, 5.0 μmol) in toluene (0.20 mL). The concentrations of *tert*-butyl 2-(benzo[*d*]-thiazol-2-yl)acetate (**1g**) and 4-vinylbiphenyl (**2c**) at 0 min, 15 min, 30 min, 45 min, 60 min and 90 min were determined by ^{1}H NMR analysis using 1,3,5-trimethoxybenzene as the internal standard. The results are listed as below:

For reactions using 0.1 mmol **1g**

Time (min)	Concentration (**1g**, mol/L)	Concentration (**2c**, mol/L)
0	0.435	0.652
15	0.390	0.620
30	0.383	0.610
40	0.311	0.555
60	0.224	0.469
80	0.184	0.443

For reactions using 0.075 mmol **1g**

Time (min)	Concentration (**1g**, mol/L)	Concentration (**2c**, mol/L)
0	0.435	0.435
15	0.430	0.427
30	0.380	0.407
45	0.366	0.391
60	0.327	0.346
90	0.194	0.228

Graphical kinetic analysis: there is a positive order in 4-vinylbiphenyl (**2c**) (approximately 1). This result should be treated with caution: each data point was determined from an individual experiment because the optimized reaction set-up uses sealed tubes, so errors are large. Further kinetic analyses are ongoing and will be reported as part of a full study.

		Experiment 1				Experiment 3			
		t (min)	$\Sigma[\mathbf{2c}]^n\Delta t$	[**1g**] (M)	[**2c**] (M)	t (min)	$\Sigma[\mathbf{2c}]^n\Delta t$	[**1g**] (M)	[**2c**] (M)
order in 2c	0.5	0.00	0.00	0.435	0.652	0.00	0.00	0.435	0.435
		15.00	11.96	0.390	0.620	15.00	9.85	0.430	0.427
		30.00	23.72	0.383	0.610	30.00	19.53	0.380	0.407
		40.00	31.35	0.311	0.555	45.00	29.01	0.366	0.391
		60.00	45.66	0.224	0.469	60.00	38.11	0.327	0.346
		80.00	59.17	0.184	0.443	90.00	54.19	0.194	0.228

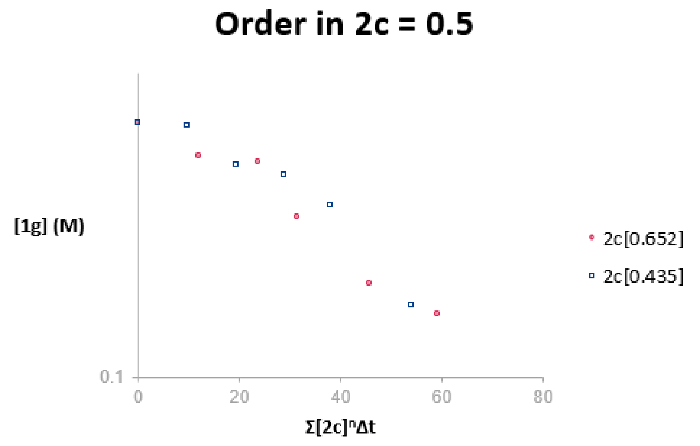

[1g]o	0.435 M
[2c]o	0.652 M
[Ir]o	0.0217 M
[1g]o	0.435 M
[2c]o	0.435 M
[Ir]o	0.0217 M

		Experiment 1				Experiment 3			
		t (min)	Σ[**2c**]^{n}Δt	[**1g**] (M)	[**2c**] (M)	t (min)	Σ[**2c**]^{n}Δt	[**1g**] (M)	[**2c**] (M)
**order in 2c**	**1**	0.00	0.00	0.435	0.652	0.00	0.00	0.435	0.435
		15.00	9.54	0.390	0.620	15.00	6.47	0.430	0.427
		30.00	18.76	0.383	0.610	30.00	12.72	0.380	0.407
		40.00	24.58	0.311	0.555	45.00	18.71	0.366	0.391
		60.00	34.82	0.224	0.469	60.00	24.23	0.327	0.346
		80.00	43.94	0.184	0.443	90.00	32.84	0.194	0.228

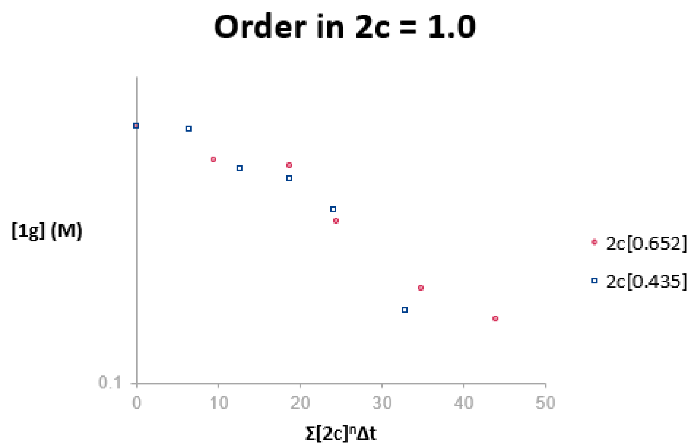

[1g]o	0.435 M
[2c]o	0.652 M
[Ir]o	0.0217 M
[1g]o	0.435 M
[2c]o	0.435 M
[Ir]o	0.0217 M

		Experiment 1				Experiment 3			
		t (min)	Σ[**2c**]^{n}Δt	[**1g**] (M)	[**2c**] (M)	t (min)	Σ[**2c**]^{n}Δt	[**1g**] (M)	[**2c**] (M)
**order in 2c**	**1.5**	0.00	0.00	0.435	0.652	0.00	0.00	0.435	0.435
		15.00	7.60	0.390	0.620	15.00	4.24	0.430	0.427
		30.00	14.83	0.383	0.610	30.00	8.28	0.380	0.407
		40.00	19.28	0.311	0.555	45.00	12.06	0.366	0.391
		60.00	26.60	0.224	0.469	60.00	15.42	0.327	0.346
		80.00	32.76	0.184	0.443	90.00	20.03	0.194	0.228

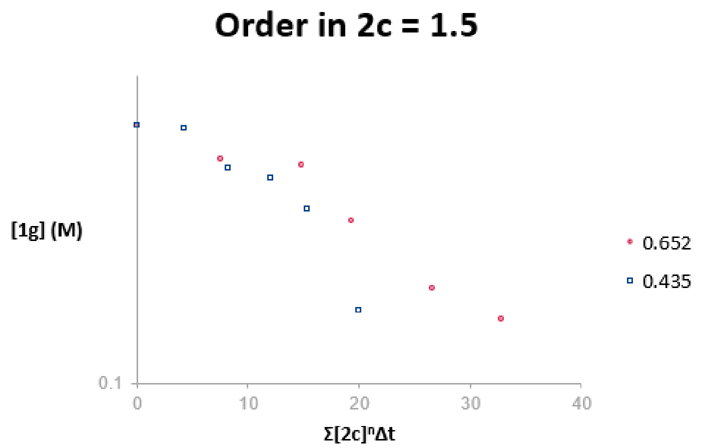

[A]o	0.435 M
[B]o	0.652 M
[cat]o	0.0217 M
[A]o	0.435 M
[B]o	0.435 M
[cat]o	0.0217 M

4. The reaction of β-ketoester and styrene to give racemic hydroalkylation product

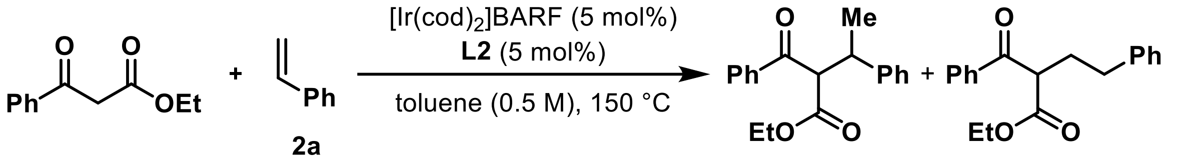

General procedure E: Ethyl 3-oxo-3-phenylpropanoate (19.2 mg, 0.10 mmol) and styrene (26.0 mg, 28.7 µL, 0.25 mmol) were employed with [Ir(cod)$_2$]BARF (6.36 mg, 0.005 mmol) and **L2** (3.01 mg, 0.005 mmol) in toluene (0.20 mL). The reaction was stirred at 150 °C for 12 h. Purification by flash column chromatography (Hexane/EtOAc = 100/1 to 20/1) afforded a mixture of regioisomers which was used in the next step.

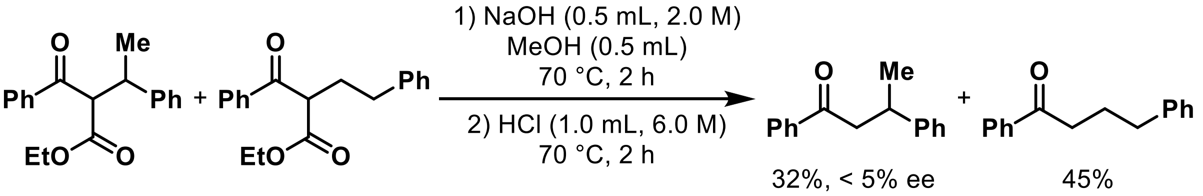

The preceding mixture of regioisomers was added methanol (0.50 mL) and aq. NaOH (0.50 mL, 2.0 M). The reaction mixture was stirred at 70 °C for 2 h in a heating plate. After cooling to room temperature, aq. HCl (1.00 mL, 6.0 M) was added and then stirred at 70 °C for 2 h in a heating plate. The reaction mixture was extracted by ethyl acetate (3 × 10 mL). The combined organic phase was dried by anhydrous MgSO4, filtered and concentrated under reduced pressure. Purification by flash column chromatography (Hexane/EtOAc = 100/1 to 20/1) afforded the titled product (7.18 mg, 32%, B:L = 1:1.4, 51:49 e.r.) as a colorless oil.

1,3-Diphenylbutan-1-one

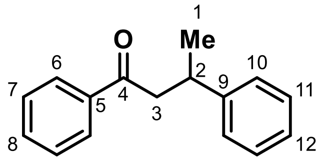

^{1}H NMR (400 MHz, CDCl$_3$) δ_H = 7.94 (d, J = 7.8 Hz, 2H, C**6**-H), 7.58 – 7.53 (m, 1H, C**8**-H), 7.48 – 7.42 (m, 2H, C**7**-H), 7.35 – 7.27 (m, 4H, 2×C**10**-H + 2×C**11**-H), 7.24 – 7.18 (m, 1H, C**12**-H), 3.58 – 3.48 (m, 1H, C**2**-H), 3.32 (dd, J = 16.4, 5.7 Hz, 1H, C**3**-H), 3.20 (d, J = 16.4, 8.2 Hz, 1H, C**3**-H’), 1.36 (d, J = 7.0 Hz, 3H, C**1**-H); ^{13}C NMR (101 MHz, CDCl$_3$) δ_C = 199.1 (**C4**), 146.6 (C**9**), 137.2 (C**5**), 133.0 (C**8**), 128.6 (C**7**), 128.5 (C**11**), 128.1 (C**6**), 126.9 (C**10**), 126.3 (C**12**), 47.0 (C**3**), 35.6 (C**2**), 21.9 (C**1**).

The spectroscopic properties were consistent with the data available in the literature.[11]

SFC conditions: CHIRALPACK SB (25 cm), 99:1 CO_2:MeOH, 1.5 mL/min, 250 nm; *Retention times*: t_{major} = 14.2 min, t_{minor} = 15.1 min.

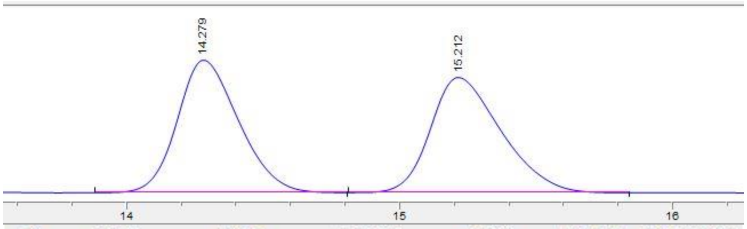

#	Time	Area	Height	Width	Area%	Symmetry
1	14.279	2219.8	141.8	0.2367	50.008	0.734
2	15.212	2219.1	123.3	0.272	49.992	0.633

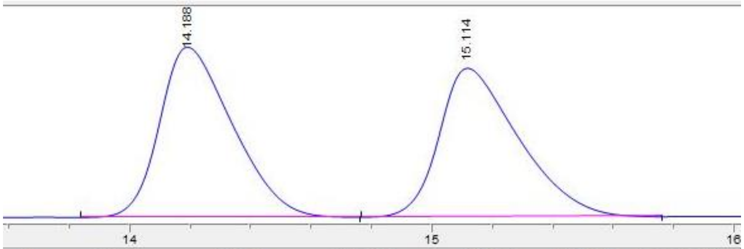

#	Time	Area	Height	Width	Area%	Symmetry
1	14.188	4134	246.1	0.254	51.183	0.6
2	15.114	3942.8	215.1	0.2738	48.817	0.535

5. The reaction of 2,6-di-*tert*-butyl-4-methylphenyl 2-(pyridin-2-yl)acetate and styrene

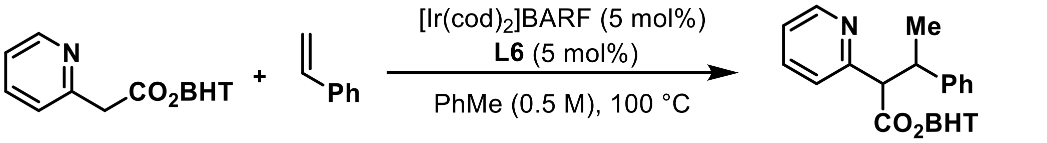

1a''' **2a** (500 mol%) **3aa'''**, 27%, B:L>25:1

28:1 d.r., 79:21 e.r./95:5 e.r.

(BHT = 2,6-di-*tert*-butyl-4-methylphenyl)

2,6-di-*tert*-Butyl-4-methylphenyl 2-(pyridin-2-yl)acetate (1a''')

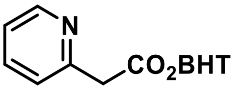

A suspension of 2-pyridylacetic acid hydrochloride (521 mg, 3.00 mmol, 1.0 eq.) and 2,6-di-*tert*-butylphenol (694 mg, 3.15 mmol, 1.05 eq.) in dichloromethane (7.5 mL) were added *N*,*N*-diisopropyl ethyl amine (DIPEA) (1.55 g, 2.09 mL, 12.0 mmol, 4.0 eq.), *N*-(3-dimethylaminopropyl)-*N*'-ethylcarbodiimide hydrochloride (EDC·HCl) (864 mg, 4.50 mmol, 1.5 eq.) and 4-(dimethylamino)pyridine (DMAP) (55.0 mg, 0.45 mmol, 0.15 eq.) at 0 °C. The reaction mixture was allowed to stir at rt for 16 h. The reaction mixture was diluted with ethyl acetate (10 mL) and extracted with 2M HCl (3 × 5 mL). The combined aqueous layers were neutralized with solid $NaHCO_3$ and extracted with ethyl acetate (3 × 10 mL). The combined organic phase was washed with brine (30 mL), dried over Na_2SO_4, filtered and concentrated *in vacuo*. The residue was purified by flash column

chromatography (Hexane/EtOAc = 10/1 to 4/1) afforded the title compound (278 mg, 27%) as a yellow oil. IR (thin film) ν_{max}/cm^{-1}: 2962, 2921, 1758, 1592, 1127, 767. ^{1}H NMR (400 MHz, $CDCl_3$) δ_H = 8.60 (dd, *J* = 4.9, 0.9 Hz, 1H), 7.72 – 7.63 (m, 1H), 7.45 (d, *J* = 7.8 Hz, 1H), 7.22 (ddd, *J* = 7.5, 4.9, 0.9 Hz, 1H), 7.09 (s, 2H), 4.16 (s, 2H), 2.30 (s, 3H), 1.28 (s, 18H); ^{13}C NMR (101 MHz, $CDCl_3$) δ_C = 170.7, 153.5, 149.6, 146.0, 142.1, 136.6, 134.7, 127.2, 124.8, 122.5, 45.6, 35.3, 31.6, 21.6. HRMS (ESI$^+$) calculated for $C_{27}H_{27}NNaO_2S$ [M+Na]$^+$ = 362.2091, found 362.2094.

2,6-di-*tert*-Butyl-4-methylphenyl 3-phenyl-2-(pyridin-2-yl)butanoate (3aa''')

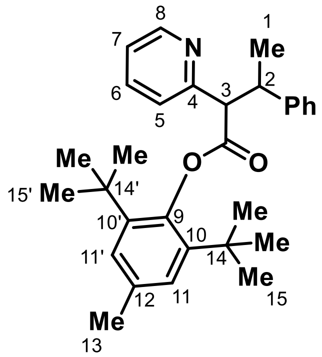

<u>General procedure E</u>: The preceding azaarylacetate (33.9 mg, 0.10 mmol) and styrene (52.1 mg, 57.5 μL, 0.50 mmol) were employed with [Ir(cod)$_2$]BARF (6.36 mg, 0.005 mmol) and **L6** (1.91 mg, 0.005 mmol) in toluene (0.20 mL). The reaction was stirred at 100 °C for 84 h. Purification by flash column chromatography (Hexane/EtOAc = 10/1 to 4/1) afforded the title compounds (12.1 mg, 27%, B:L > 25:1, 28:1 d.r., 79:21 e.r./95:5 e.r.) as colorless oils. IR (thin film) ν_{max}/cm^{-1}: 2963, 2927, 2869, 1758, 1588, 1100, 762. Diastereomer 1 (Major): ^{1}H NMR (500 MHz, $CDCl_3$) δ_H = 8.74 – 8.66 (m, 1H, C**8**-<u>H</u>), 7.75 (ddd, *J* = 7.7, 7.5, 1.2 Hz, 1H, C**6**-<u>H</u>), 7.54 – 7.48 (m, 1H, C**7**-<u>H</u>), 7.42 (d, *J* = 7.5 Hz, 2H, Ph ArC<u>H</u>), 7.30 – 7.24 (m, 3H, Ph ArC<u>H</u>), 7.17 (d, *J* = 7.5 Hz, 1H, C**5**-<u>H</u>), 6.96 (s, 1H, C**11**-<u>H</u>), 6.95 (s, 1H, C**11**'-<u>H</u>), 4.31 (d, *J* = 10.8 Hz, 1H, C**3**-<u>H</u>), 3.93 – 3.87 (m, 1H, C**2**-<u>H</u>), 2.21 (s, 3H, C**13**-<u>H$_3$</u>), 1.02 (d, *J* = 7.1 Hz, 3H, C**1**-<u>H$_3$</u>), 0.91 (s, 9H, C**15**-<u>H$_3$</u>), 0.82 (s, 9H, C**15'**-<u>H$_3$</u>); ^{13}C NMR (125 MHz, $CDCl_3$) δ_C = 170.4 (<u>C</u>=O), 156.4 (C**4**), 149.9 (C**8**), 146.3 (Ph Ar<u>C</u>), 145.0 (C**9**), 142.5 (C**10'**), 142.2 (C**10**), 137.0 (C**6**), 134.4 (C**12**), 128.8 (Ph Ar<u>C</u>H), 128.4 (Ph Ar<u>C</u>H), 127.2 (Ph Ar<u>C</u>H), 126.8 (C**11**), 126.7 (C**11'**), 125.7 (C**5**), 122.8 (C**7**), 62.4 (C**3**), 41.1 (C**2**), 34.8 (C**14'**), 34.7 (C**14**), 31.2 (C**15**), 30.7 (C**15'**), 21.4 (C**13**), 20.1 (C**1**). HRMS (CI$^+$) calculated for $C_{30}H_{38}NO_2$ [M+H]$^+$ = 444.2897, found 444.2905.

SFC conditions: CHIRALPACK SB (25 cm), 97:3 CO_2:MeOH, 2.0 mL/min, 210 nm; *Retention times*: *Diastereomer 1:* t_{minor} = 9.7 min, t_{major} = 11.7 min; *Diastereomer 2:* t_{minor} = 7.3 min, t_{major} = 7.8 min.

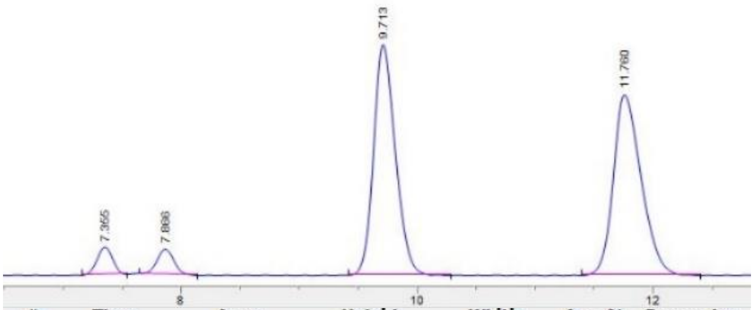

#	Time	Area	Height	Width	Area%	Symmetry
1	7.355	112.4	12.2	0.1455	3.923	1.022
2	7.866	109.5	11.1	0.1528	3.824	0.909
3	9.713	1318.9	102.8	0.1957	46.046	0.745
4	11.76	1323.6	80.5	0.2498	46.207	0.651

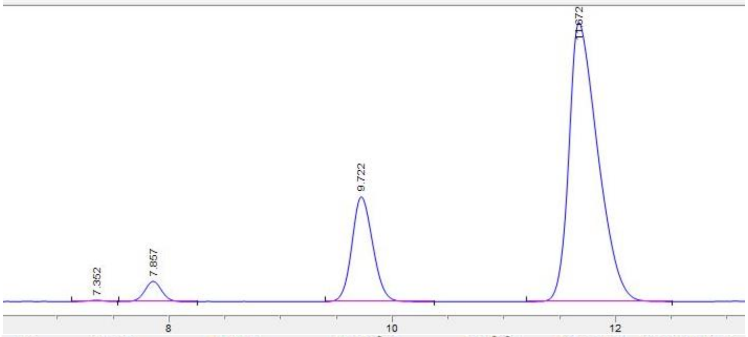

#	Time	Area	Height	Width	Area%	Symmetry
1	7.352	45.8	4.9	0.1493	0.182	1.302
2	7.857	809.4	76.8	0.1629	3.213	0.96
3	9.722	5202.1	398.5	0.2023	20.651	0.802
4	11.672	19132.7	1061.1	0.2764	75.954	0.514

Details of the Synthesis and Evaluation of Unsuccessful Substrates

1. The reaction of *tert*-Butyl 2-(4,6-dimethoxypyrimidin-2-yl)acetate and styrene

tert-Butyl 2-(4,6-dimethoxypyrimidin-2-yl)acetate (1t)

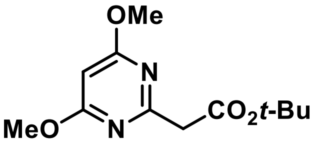

General procedure C: 2-Chloro-4,6-dimethoxypyrimidine (698 mg, 4.00 mmol) was employed with *tert*-butyl acetate (1.39 g, 1.61 mL, 12.0 mmol) and NaHMDS (0.6 M in toluene, 20.0 mL, 12.0 mmol). Purification by flash column chromatography (Hexane/EtOAc = 8/1 to 4/1) afforded the title compound (737 mg, 72%) as a colorless oil. IR (thin film) ν_{max}/cm^{-1}: 2979, 2953, 1733, 1589, 1140, 832. ^{1}H NMR (500 MHz, $CDCl_3$) δ_H = 5.90 (s, 1H), 3.91 (s, 6H), 3.72 (s, 2H), 1.47 (s, 9H); ^{13}C NMR (125 MHz, $CDCl_3$) δ_C = 171.6, 169.0, 164.2, 87.8, 81.1, 54.2, 46.6, 28.2. HRMS (ESI$^+$) calculated for $C_{12}H_{18}N_2NaO_4$ [M+Na]$^+$ = 277.1159, found 277.1157.

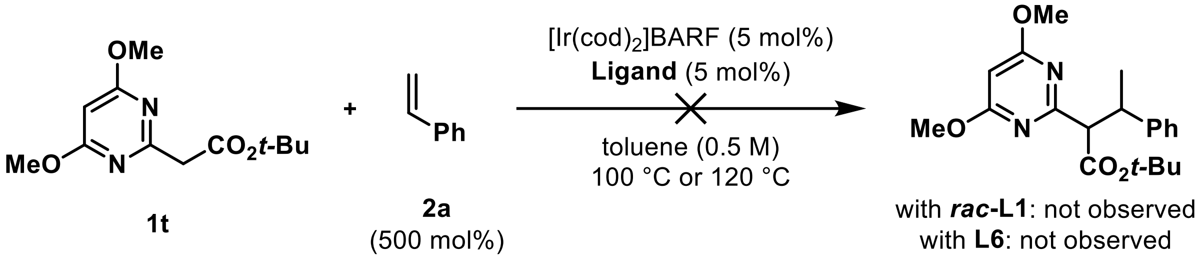

General procedure E: The preceding *tert*-butyl 2-(4,6-dimethoxypyrimidin-2-yl)acetate (25.4 mg, 0.10 mmol) and styrene (52.1 mg, 57.5 μL, 0.50 mmol) were employed with [Ir(cod)$_2$]BARF (6.36 mg, 0.005 mmol) and ***rac*-L1** (3.11 mg, 0.005 mmol) or **L6** (1.91 mg, 0.005 mmol) in toluene (0.20 mL). The reaction was stirred at 100 °C or 120 °C for 24 h. The reaction mixture was concentrated in *vacuo*. The ^{1}H NMR spectrum of the crude mixture indicated the results shown above.

2. The reaction of *tert*-butyl 2-(3-chloroquinoxalin-2-yl)acetate and styrene

***tert*-Butyl 2-(3-chloroquinoxalin-2-yl)acetate (1u)**

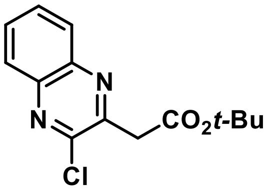

General procedure C: 2,3-Dichloroquinoxaline (796 mg, 4.00 mmol) was employed with *tert*-butyl acetate (1.39 g, 1.61 mL, 12.0 mmol) and NaHMDS (0.6 M in toluene, 20.0 mL, 12.0 mmol). Purification by flash column chromatography (Hexane/EtOAc = 15/1 to 9/1) afforded the title compound (374 mg, 38%) as an orange solid. m.p. 92 – 95 °C (EtOAc/hexane). IR (thin film) ν_{max}/cm^{-1}: 2978, 2935, 1727, 1154, 1044, 775. ^{1}H NMR (500 MHz, CDCl$_3$) δ_H = 8.09 – 8.05 (m, 1H), 8.03 – 7.99 (m, 1H), 7.79 – 7.75 (m, 2H), 4.13 (s, 2H), 1.47 (s, 9H); ^{13}C NMR (125 MHz, CDCl$_3$) δ_C = 168.1, 149.7, 147.8, 141.4, 140.9, 130.8, 130.4, 129.0, 128.3, 82.4, 43.8, 28.1. HRMS (ESI$^+$) calculated for $C_{14}H_{16}ClN_2O_2$ [M+H]$^+$ = 279.0895, found 279.0900.

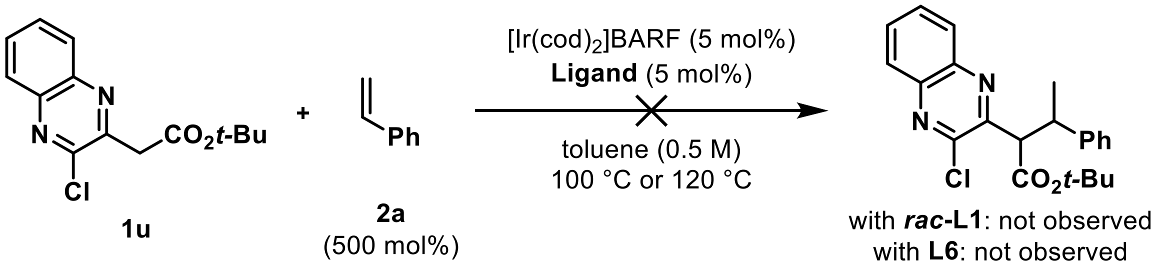

General procedure E: The preceding *tert*-butyl 2-(3-chloroquinoxalin-2-yl)acetate (24.4 mg, 0.10 mmol) and styrene (52.1 mg, 57.5 μL, 0.50 mmol) were employed with [Ir(cod)$_2$]BARF (6.36 mg, 0.005 mmol) and ***rac*-L1** (3.11 mg, 0.005 mmol) or **L6** (1.91 mg, 0.005 mmol) in toluene (0.20 mL). The

reaction was stirred at 100 °C or 120 °C for 24 h. The reaction mixture was concentrated in *vacuo*. The ^{1}H NMR spectrum of the crude mixture indicated the results shown above.

3. The reaction of *tert*-butyl 2-(benzo[*d*]thiazol-2-yl)acetate and α-ethyl styrene

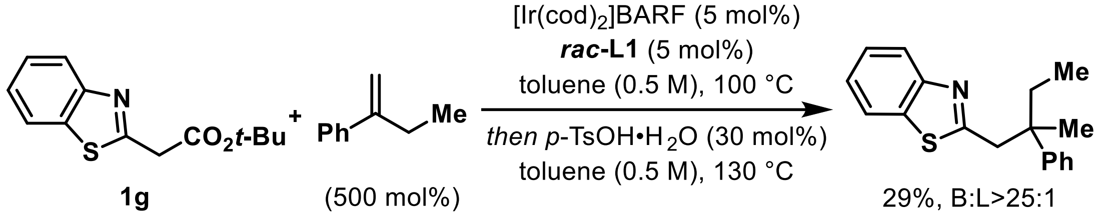

2-(2-Methyl-2-phenylbutyl)benzo[*d*]thiazole

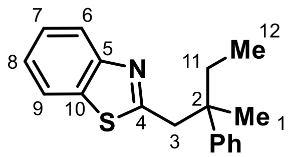

<u>General procedure E</u>: *tert*-Butyl 2-(benzo[*d*]thiazol-2-yl)acetate (24.9 mg, 0.10 mmol) and styrene (66.1 mg, 75.0 μL, 0.50 mmol) were employed with [Ir(cod)$_2$]BARF (6.36 mg, 0.005 mmol) and ***rac*-L1** (3.11 mg, 0.005 mmol) in toluene (0.20 mL). The reaction was stirred at 100 °C for 24 h. Then *p*-toluenesulfonic acid monohydrate (5.71 mg, 0.03 mmol) was added and the resulting reaction mixture was heated at 130 °C for 3 h. Purification by flash column chromatography (Hexane/EtOAc = 15/1 to 9/1) afforded the title compound (8.00 mg, 29%, B:L > 25:1) as a colorless oil. IR (thin film) ν_{max}/cm^{-1}: 2962, 2931, 1660, 1512, 1441, 1013, 759. ^{1}H NMR (500 MHz, CDCl$_3$) δ_H = 7.92 (d, *J* = 8.2 Hz, 1H, C**6**-<u>H</u>), 7.64 (d, *J* = 8.0 Hz, 1H, C**9**-<u>H</u>), 7.39 – 7.36 (m, 1H, C**7**-<u>H</u>), 7.35 – 7.28 (m, 4H, Ph ArC<u>H</u>), 7.26 – 7.21 (m, 2H, C**8**-<u>H</u> + Ph ArC<u>H</u>), 3.48 (d, *J* = 14.1 Hz, 1H, C**3**-<u>H</u>), 3.40 (d, *J* = 14.1 Hz, 1H, C**3**-<u>H’</u>), 1.95 (dq, *J* = 14.7, 7.4 Hz, 1H, C**11**-<u>H</u>), 1.72 (dq, *J* = 14.7, 7.4 Hz, 1H, C**11**-<u>H’</u>), 1.36 (s, 3H, C**1**-<u>H$_3$</u>), 0.72 (dd, *J* = 7.4, 7.4 Hz, 3H, C**12**-<u>H$_3$</u>); ^{13}C NMR (125 MHz, CDCl$_3$) δ_C = 169.0 (C**4**), 152.4 (C**5**), 145.7 (Ph Ar<u>C</u>), 135.7 (C**10**), 128.5 (Ph Ar<u>C</u>H), 127.1 (Ph Ar<u>C</u>H), 126.4 (Ph Ar<u>C</u>H), 125.7 (C**7**), 124.7 (C**8**), 122.6 (C**6**), 121.4 (C**9**), 48.4 (C**3**), 42.5 (C**2**), 35.9 (C**11**), 22.9 (C**1**), 8.8 (C**12**). HRMS (CI$^+$) calculated for $C_{18}H_{20}NS$ [M+H]$^+$ = 282.1310, found 282.1302.

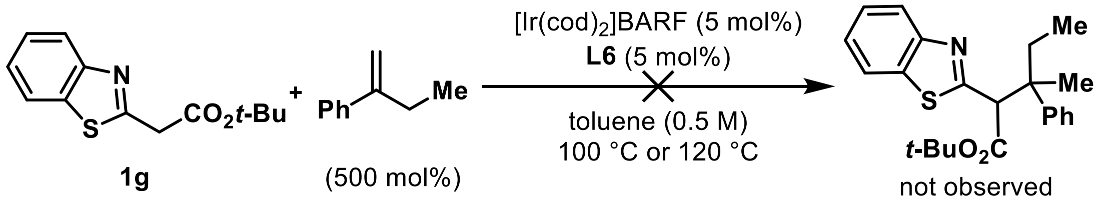

<u>General procedure E</u>: *tert*-Butyl 2-(benzo[*d*]thiazol-2-yl)acetate (24.9 mg, 0.10 mmol) and styrene (66.1 mg, 75.0 μL, 0.50 mmol) were employed with [Ir(cod)$_2$]BARF (6.36 mg, 0.005 mmol) and **L6** (1.91 mg, 0.005 mmol) in toluene (0.20 mL). The reaction was stirred at 100 °C or 120 °C for 48 h. The reaction mixture was concentrated in *vacuo*. The ^{1}H NMR spectrum of the crude mixture indicated the results shown above.

NMR Spectra for Novel Compounds

(*S*)-2-(2-Phenylpropyl)pyridine (4aa)

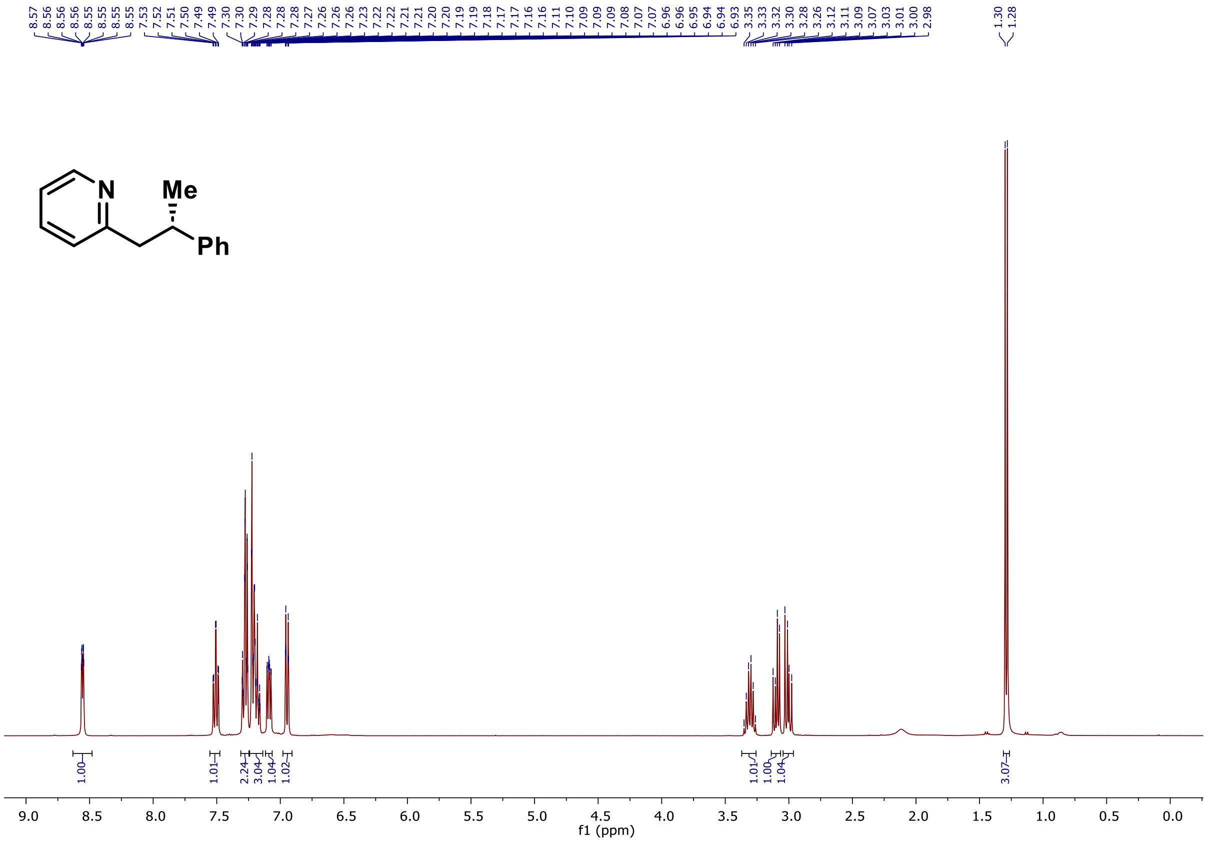

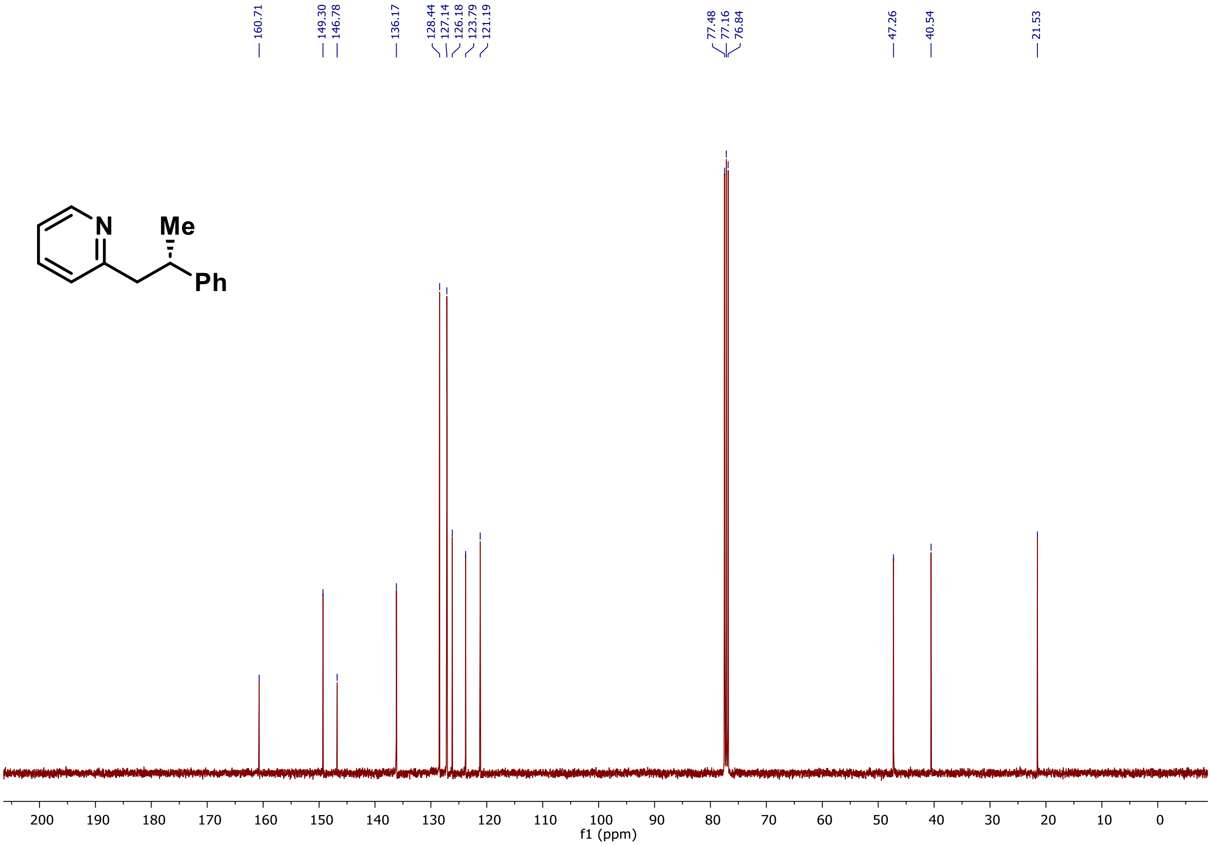

(*S*)-2-Fluoro-6-(2-phenylpropyl)pyridine (4ba)

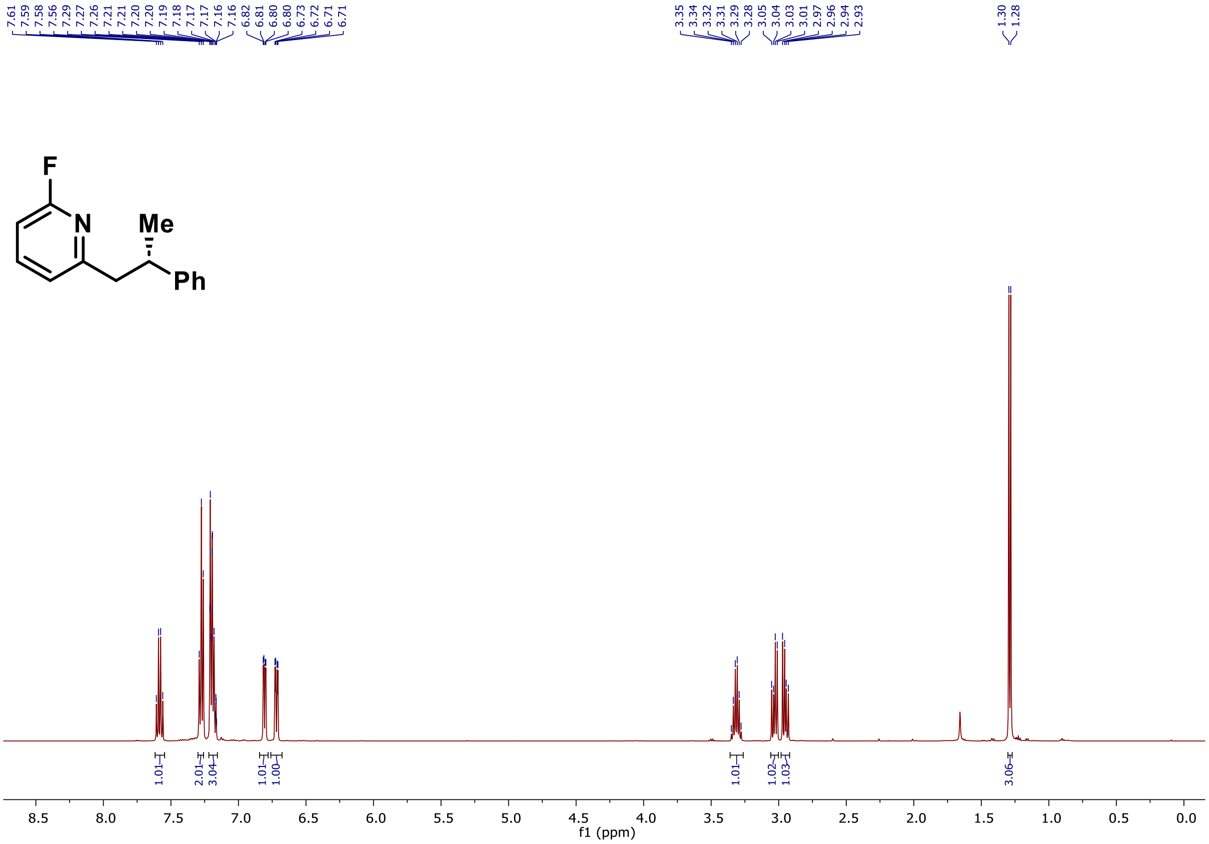

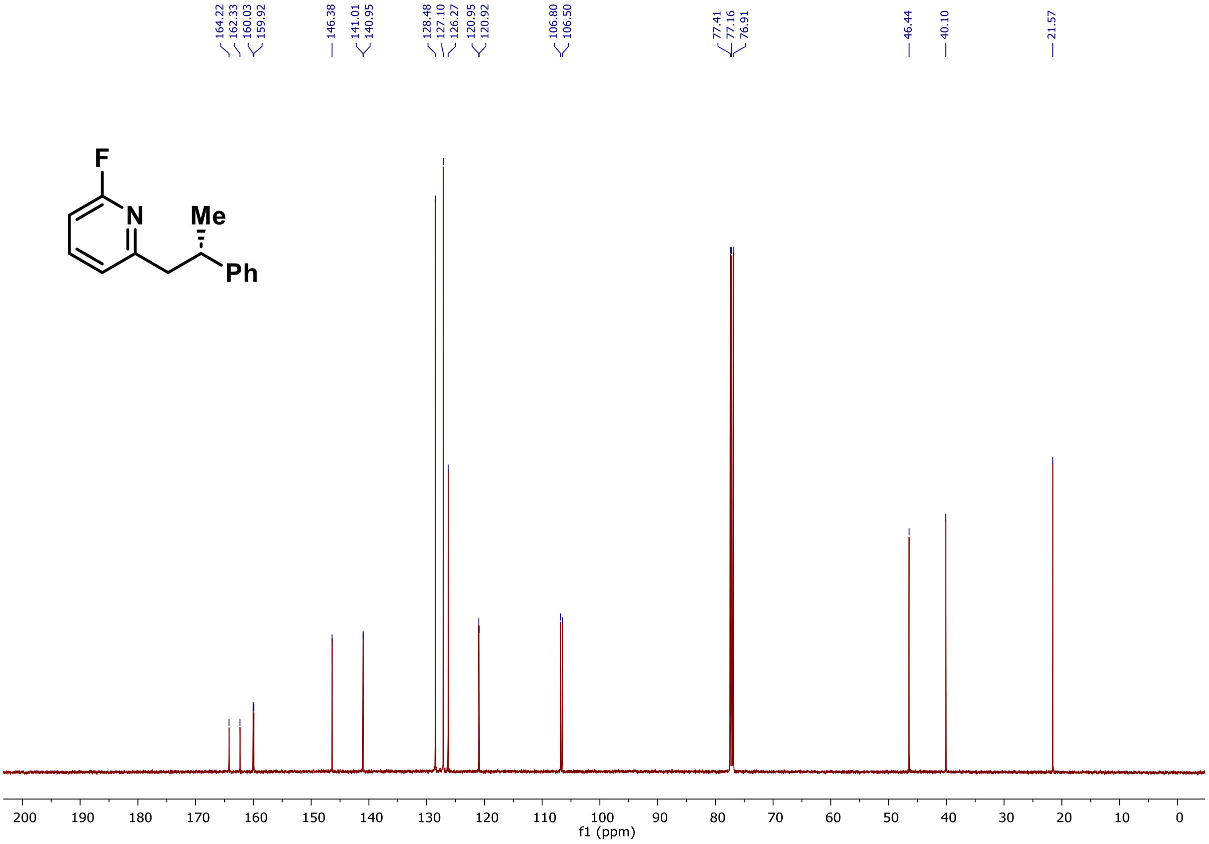

tert-Butyl 2-(6-methoxypyridin-2-yl)acetate (1c)

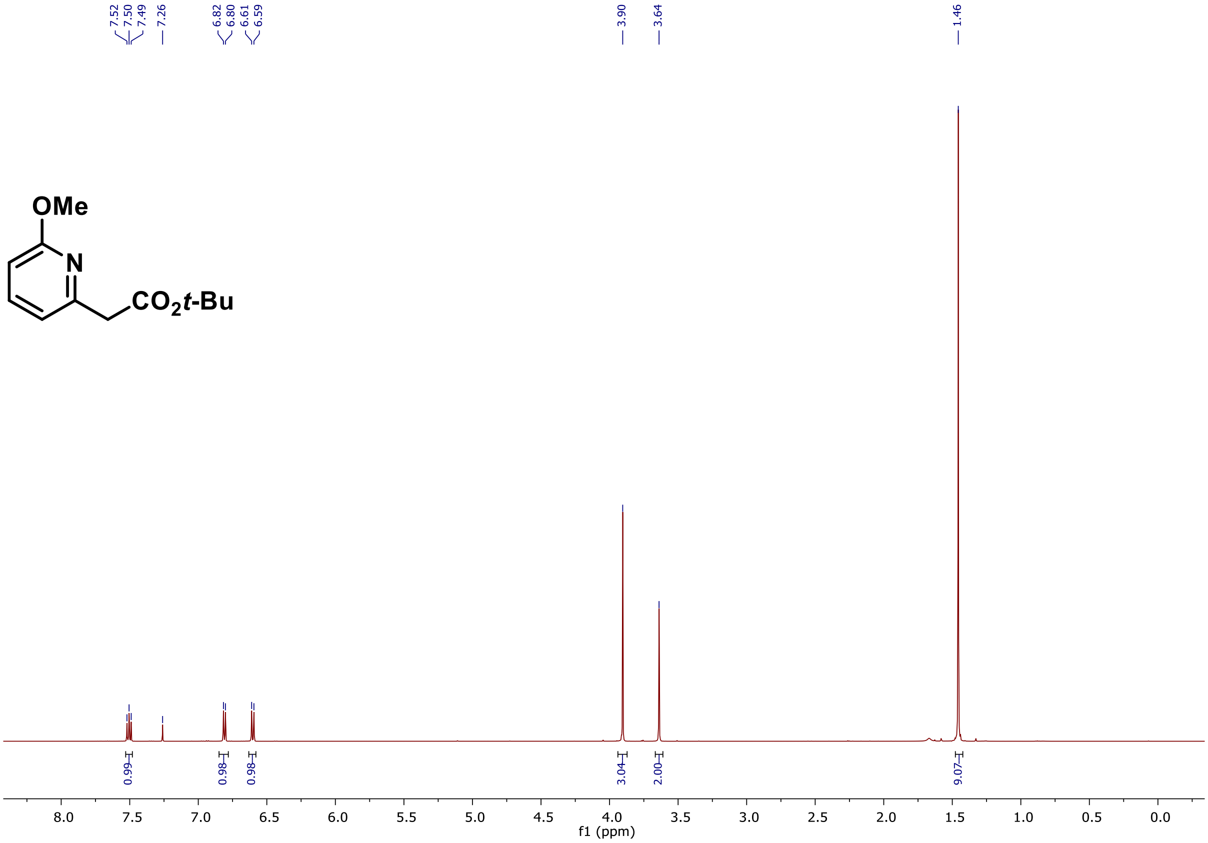

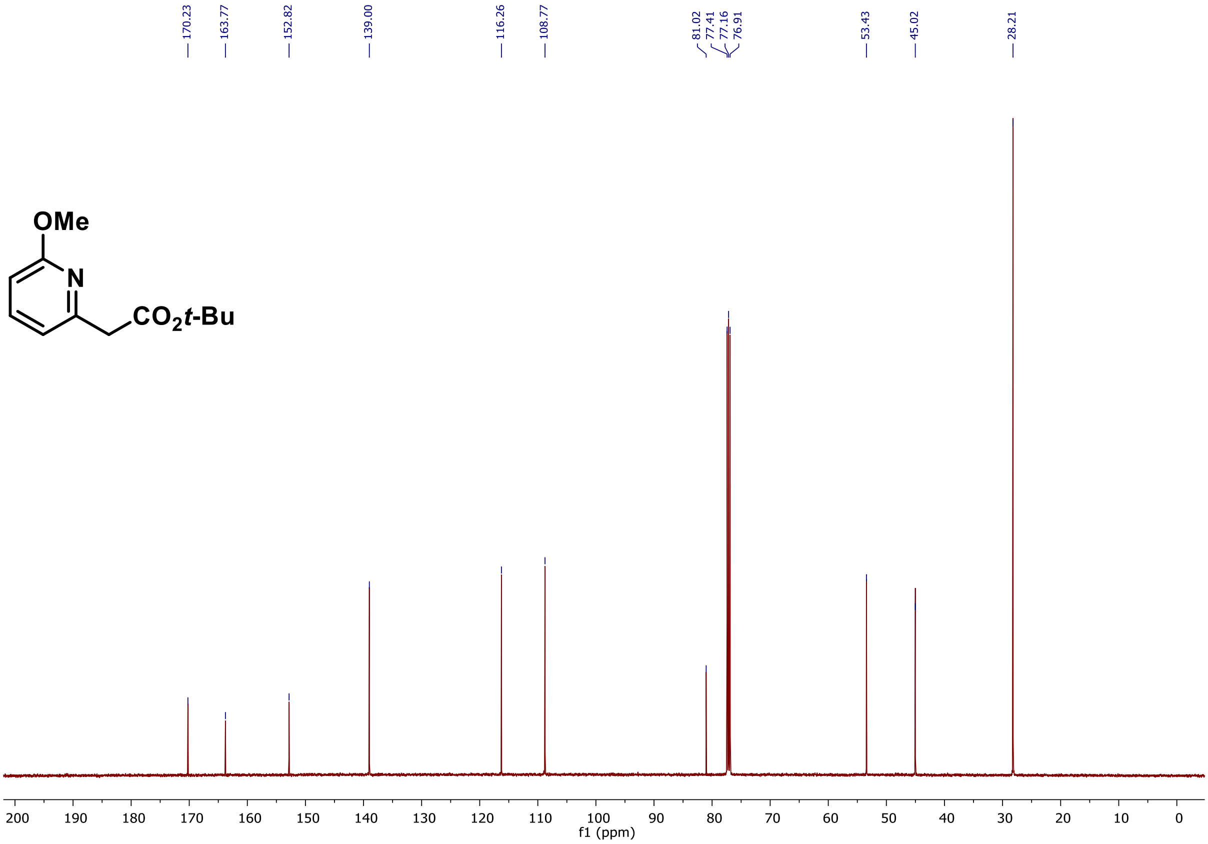

(*S*)-2-Methoxy-6-(2-phenylpropyl)pyridine (4ca)

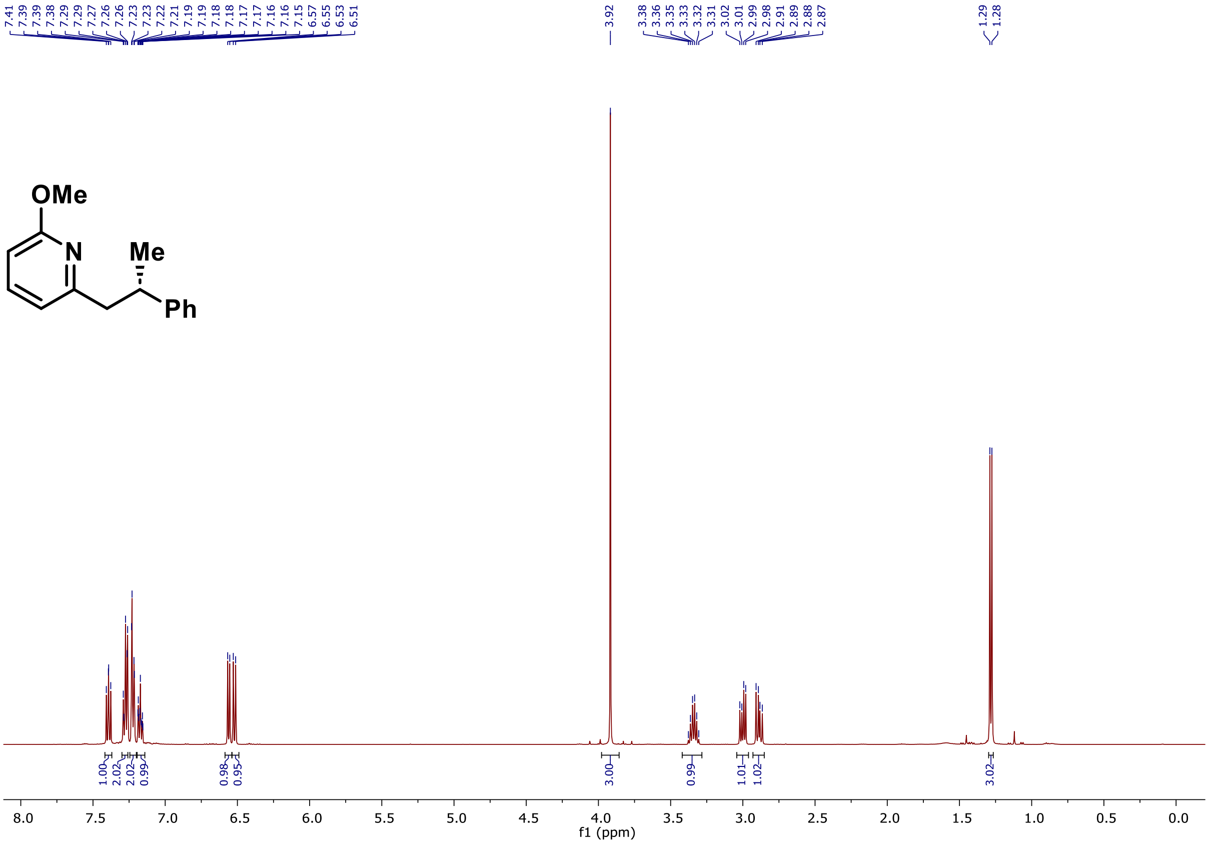

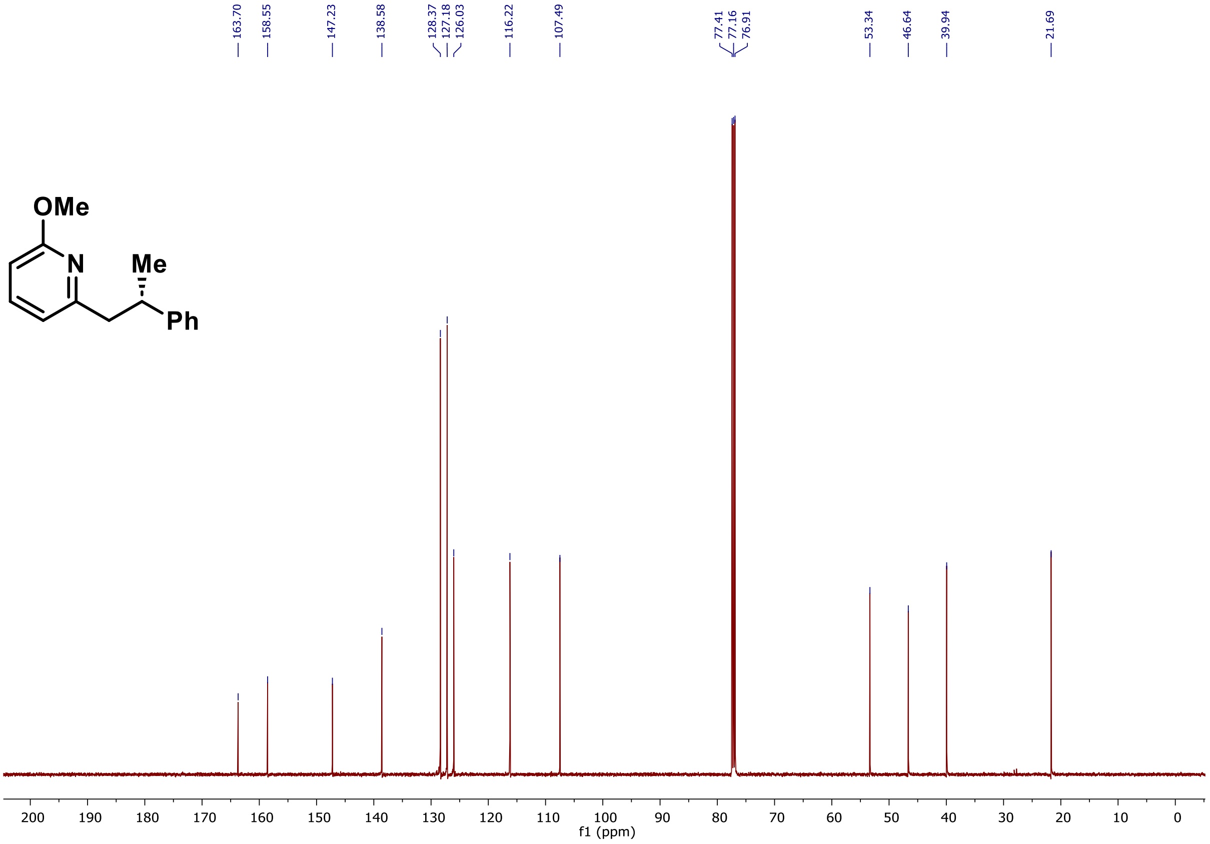

***tert*-Butyl 2-(5-methoxypyridin-2-yl)acetate (1d)**

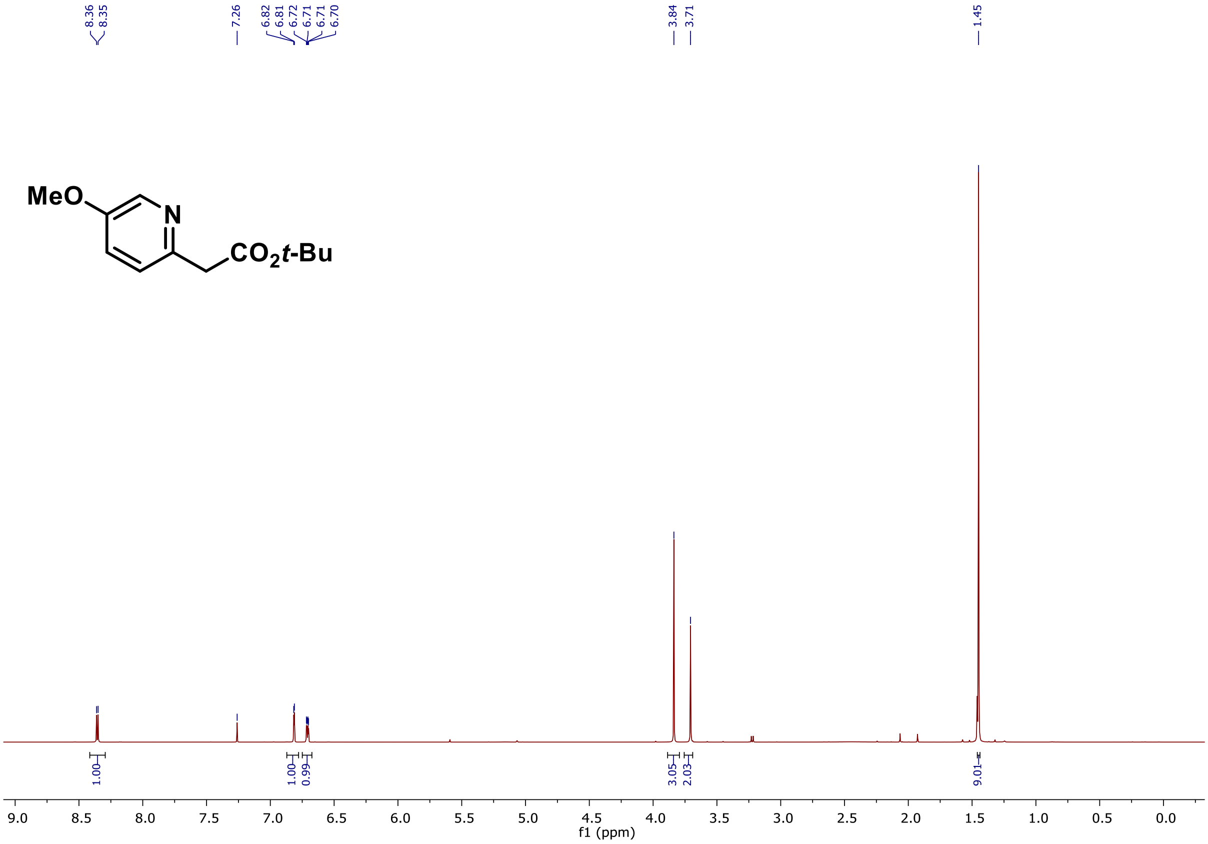

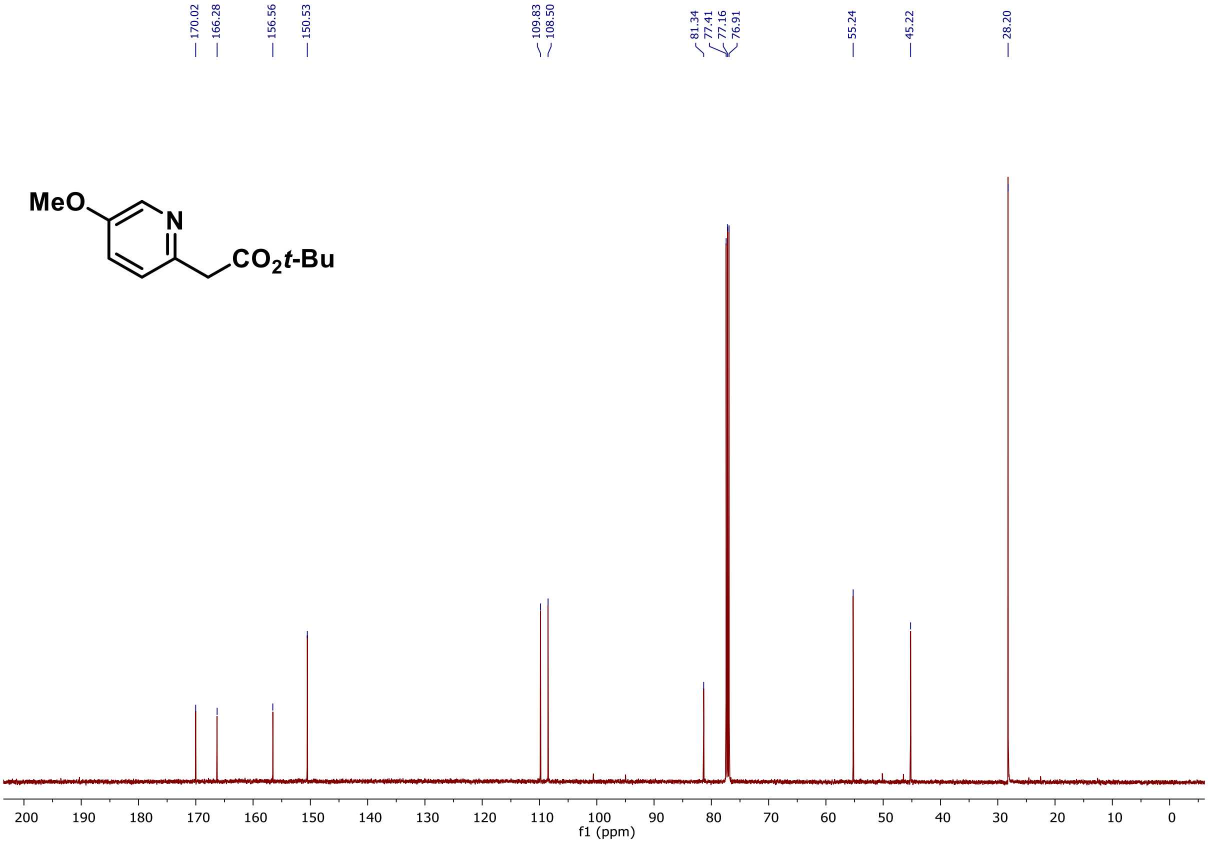

tert-Butyl (3S)-2-(5-methoxypyridin-2-yl)-3-phenylbutanoate (3da)

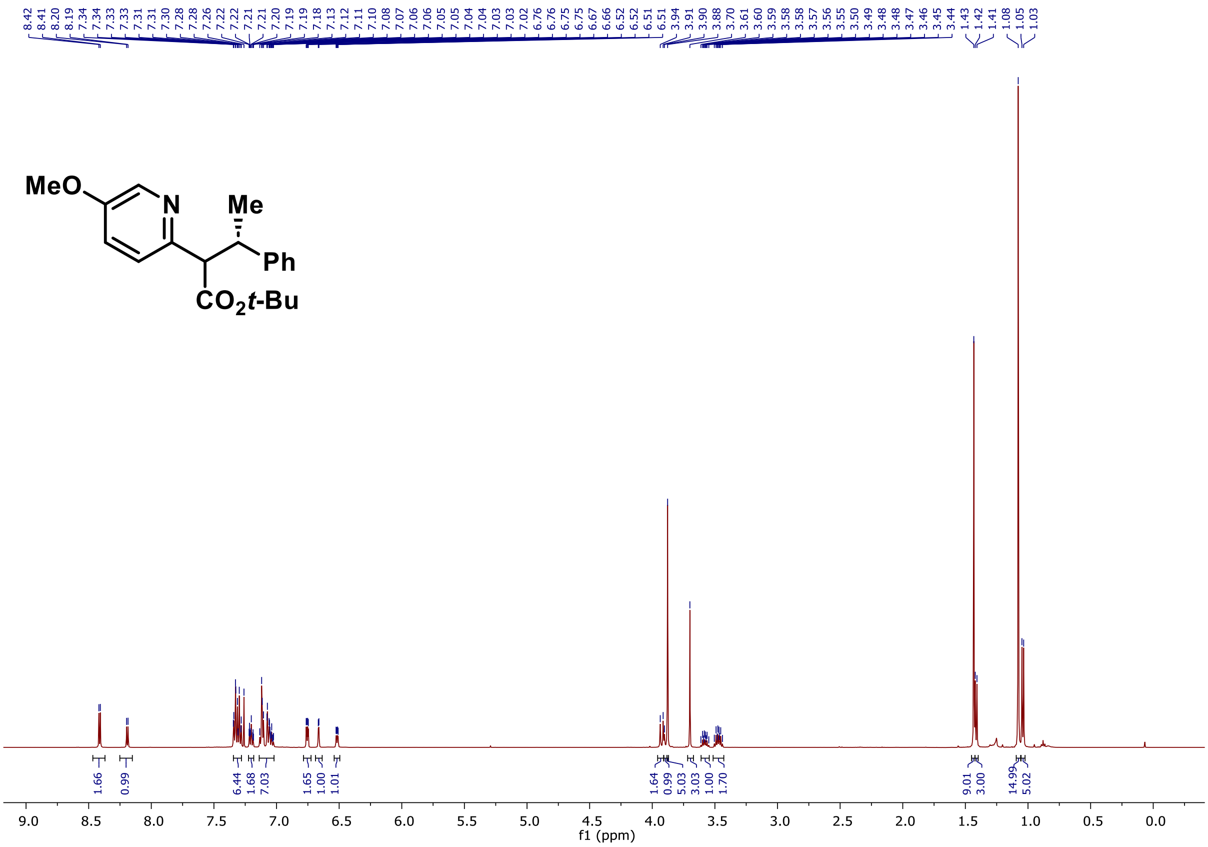

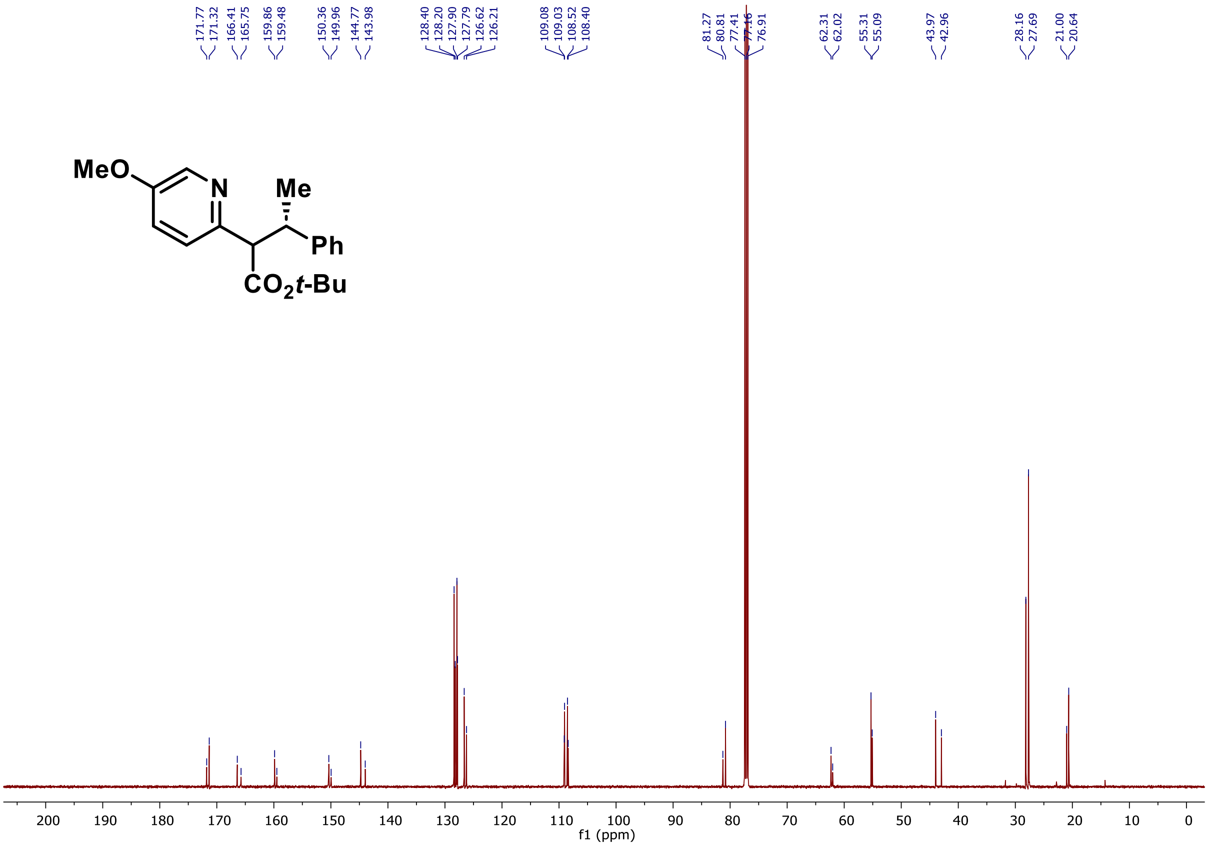

***tert*-Butyl 2-(5-chloropyridin-2-yl)acetate (1e)**

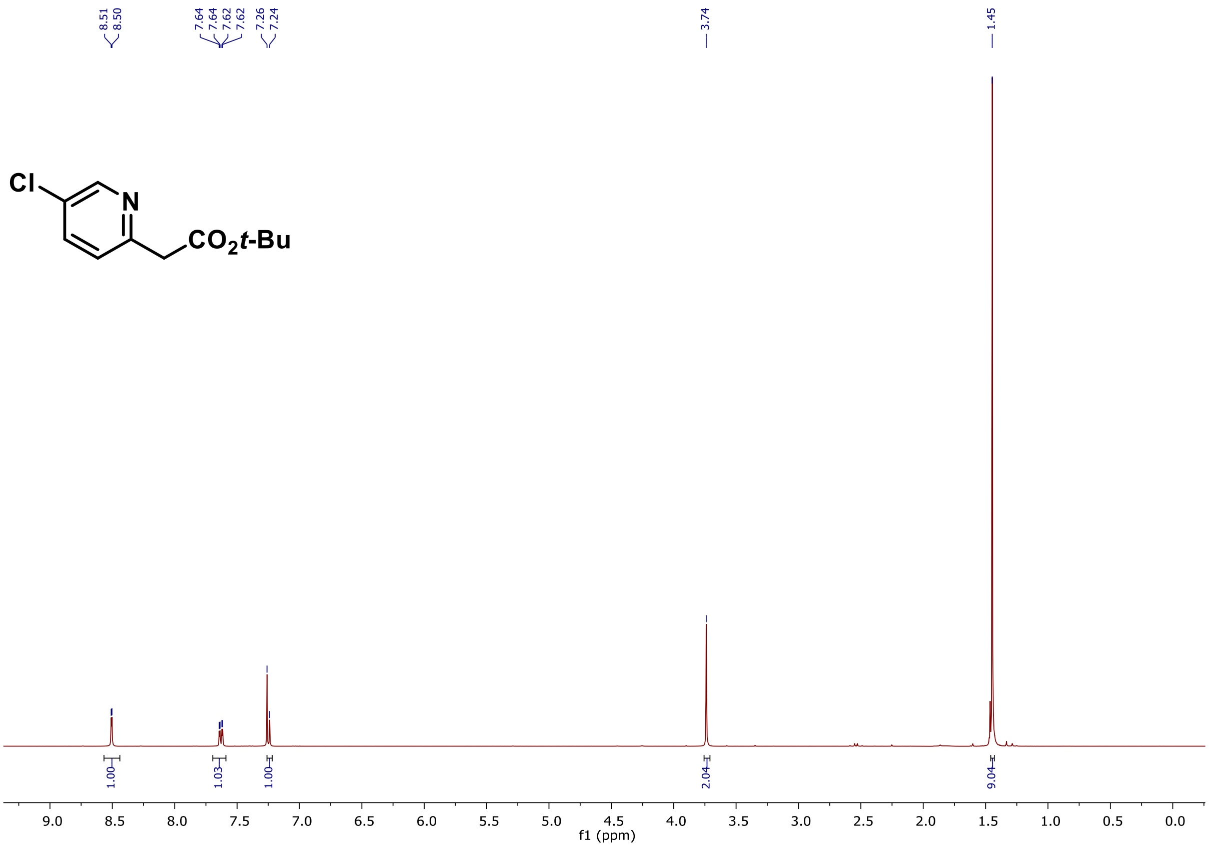

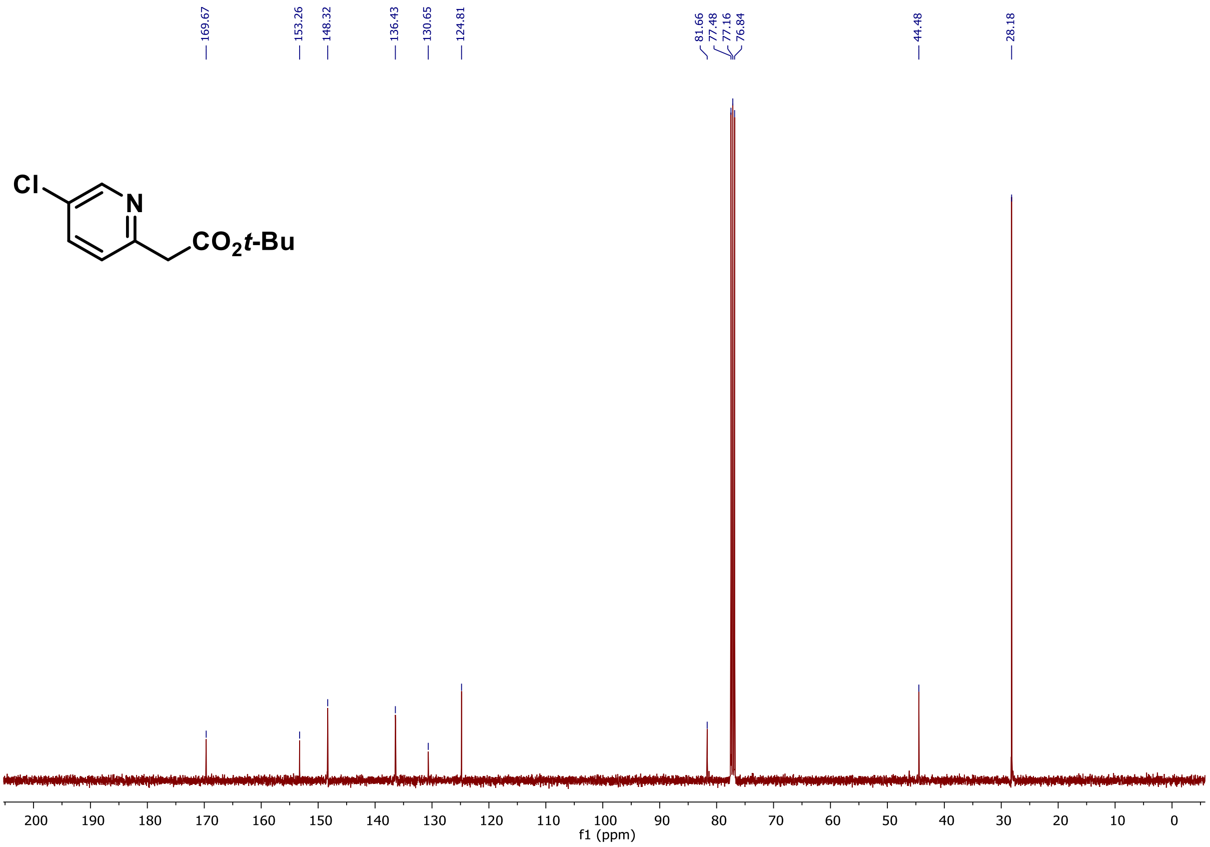

(*S*)-5-Chloro-2-(2-phenylpropyl)pyridine (4ea)

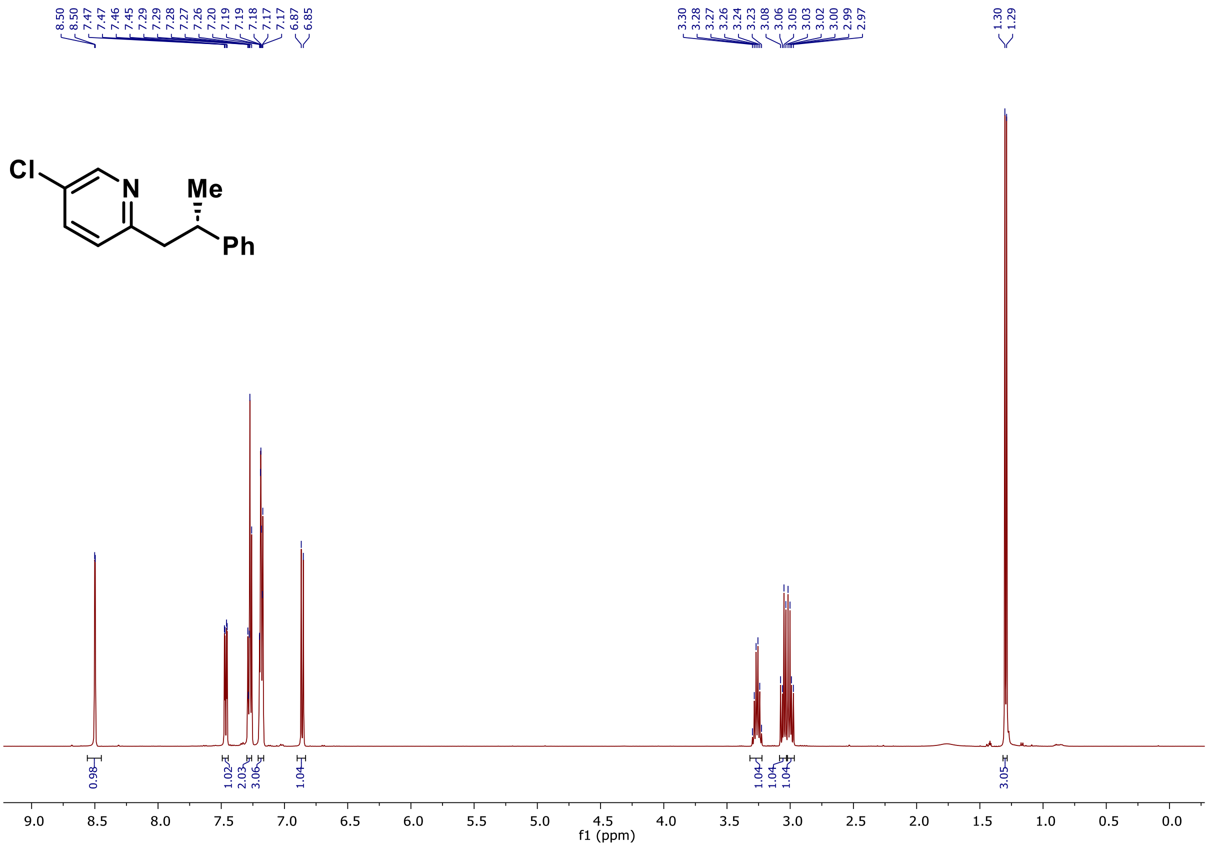

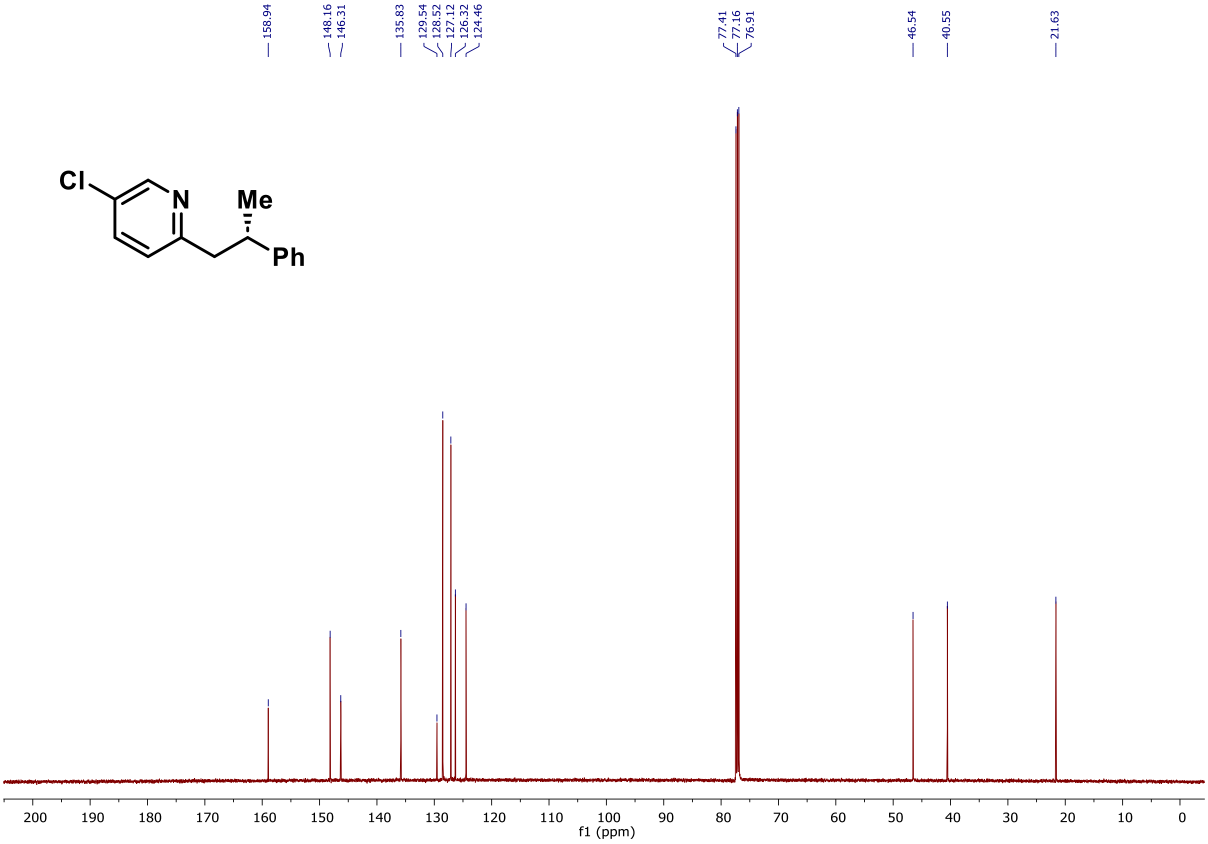

***tert*-Butyl 2-(4-(trifluoromethyl)pyridin-2-yl)acetate (1f)**

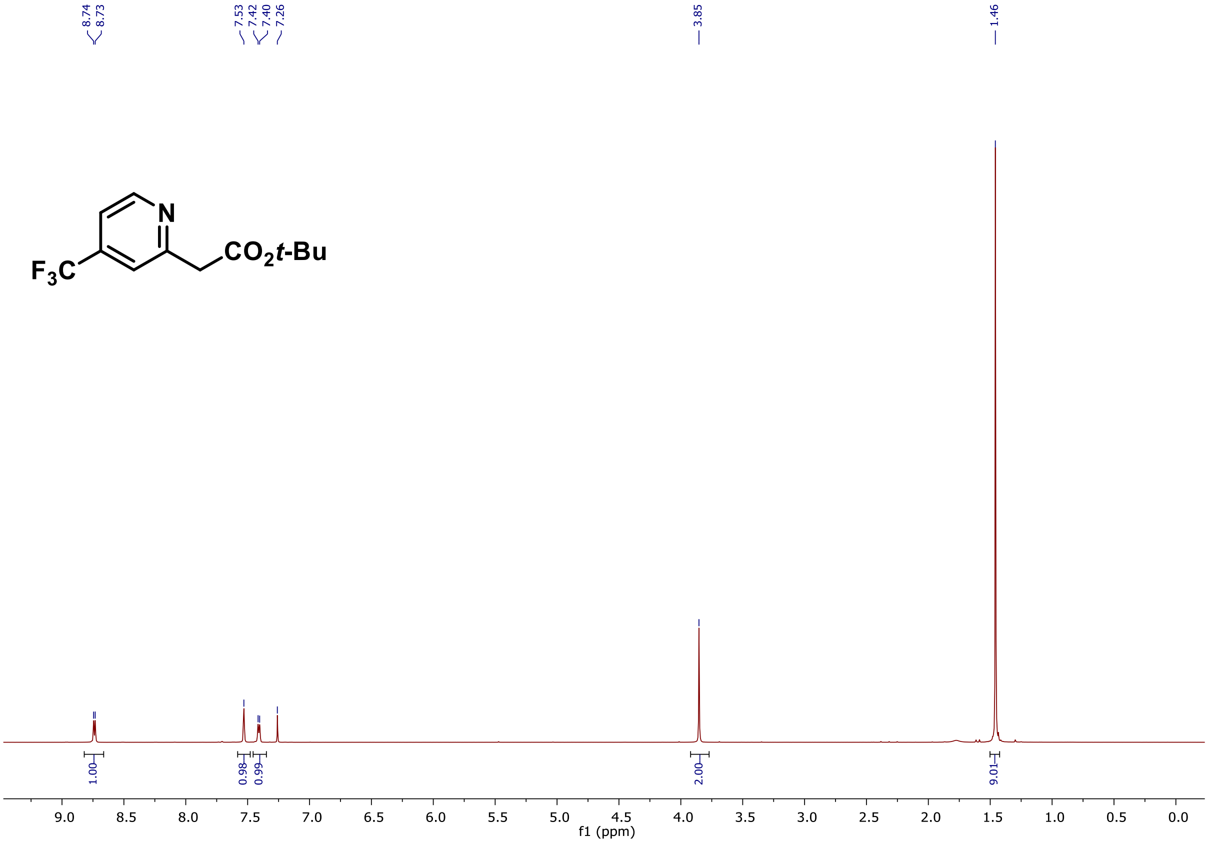

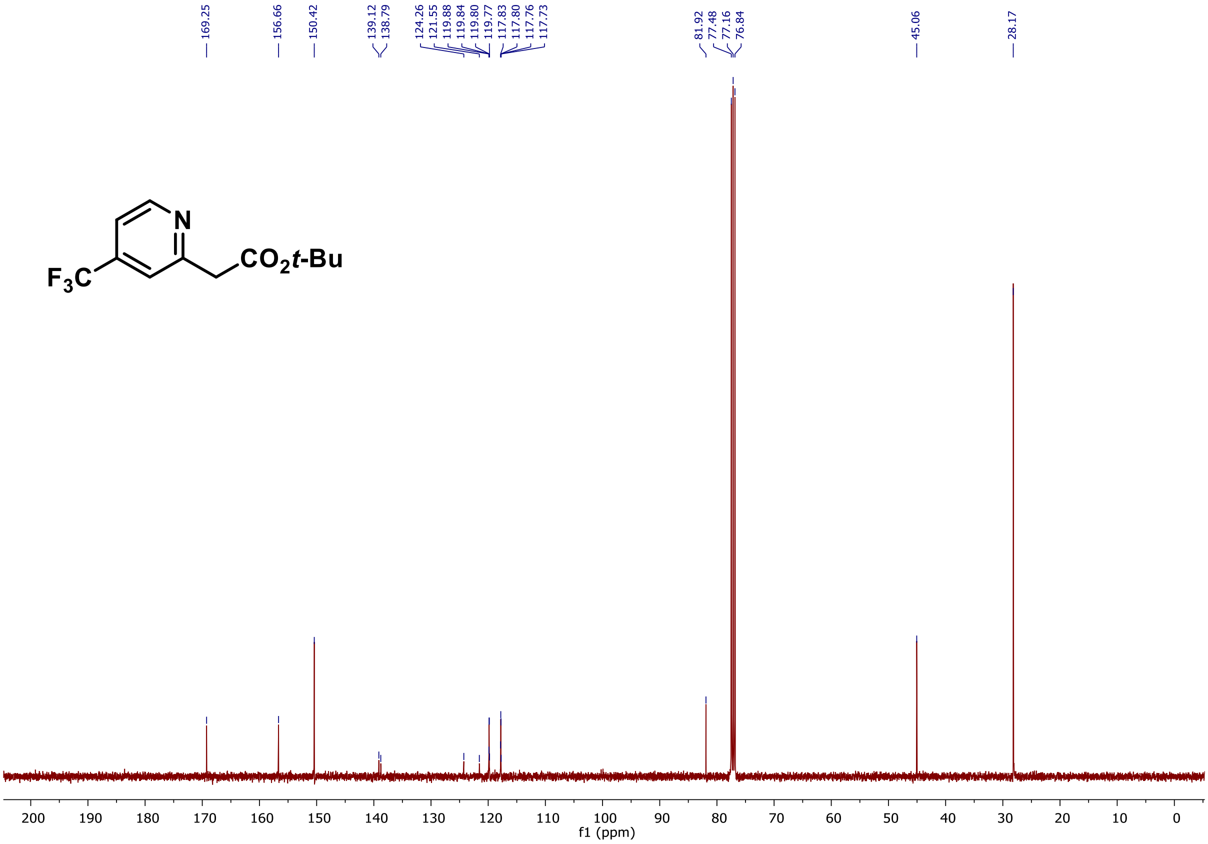

(*S*)-2-(2-Phenylpropyl)-4-(trifluoromethyl)pyridine (4fa)

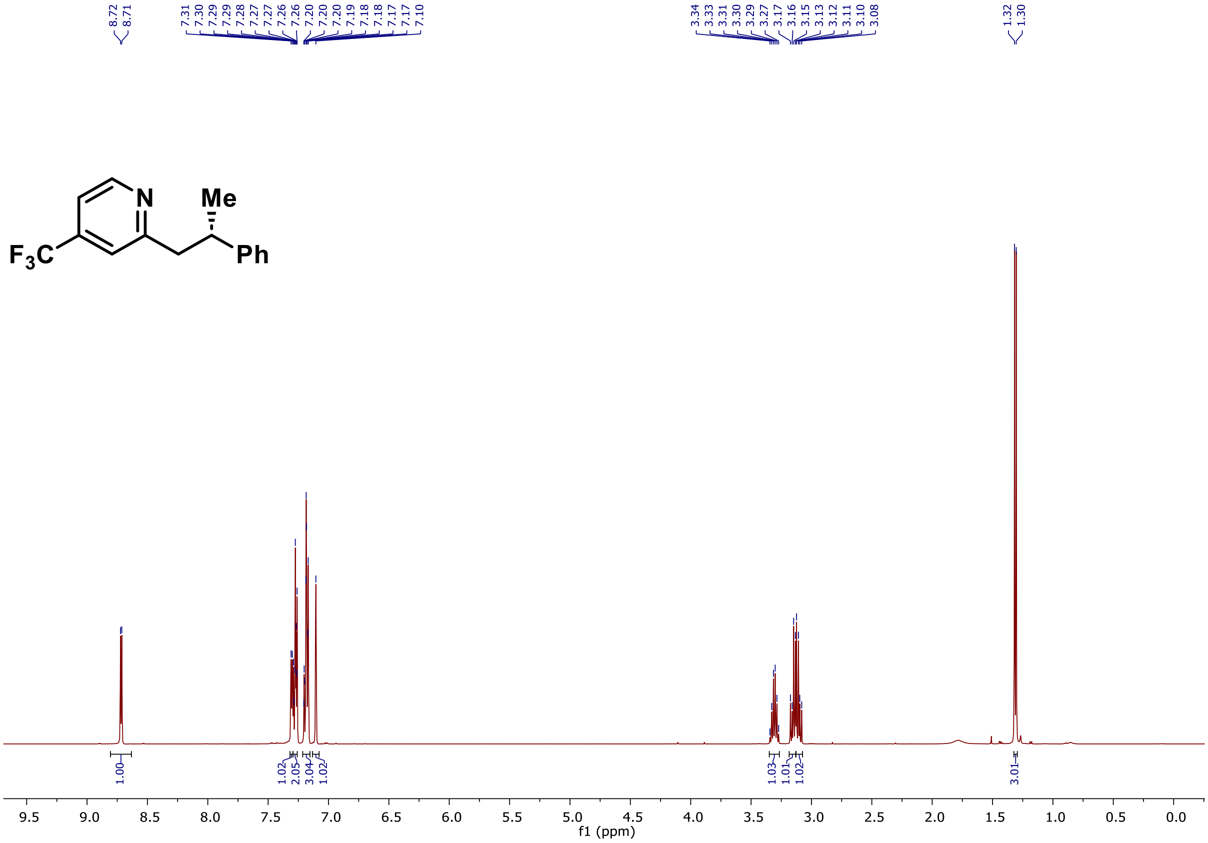

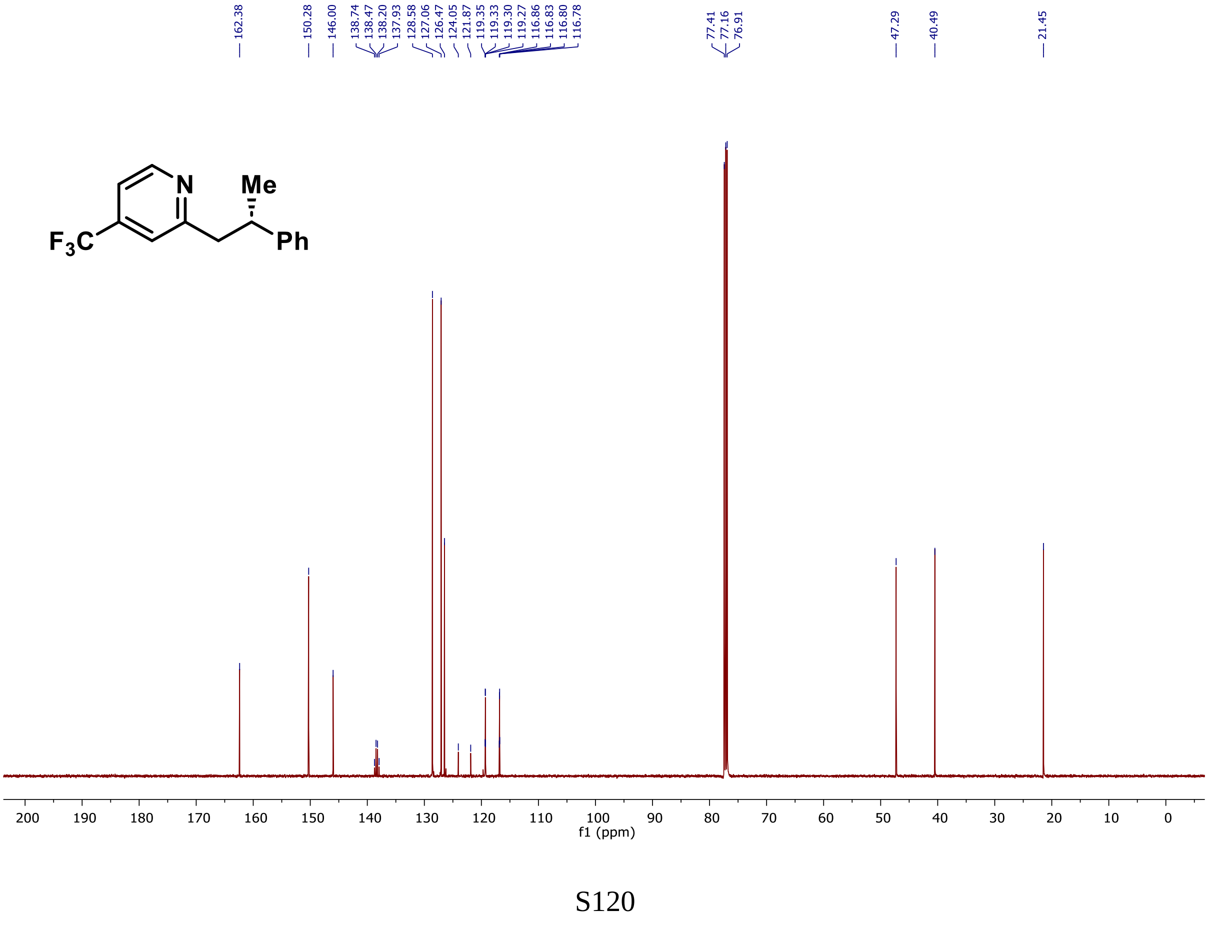

(*S*)-2-(2-Phenylpropyl)benzo[*d*]thiazole (4ga)

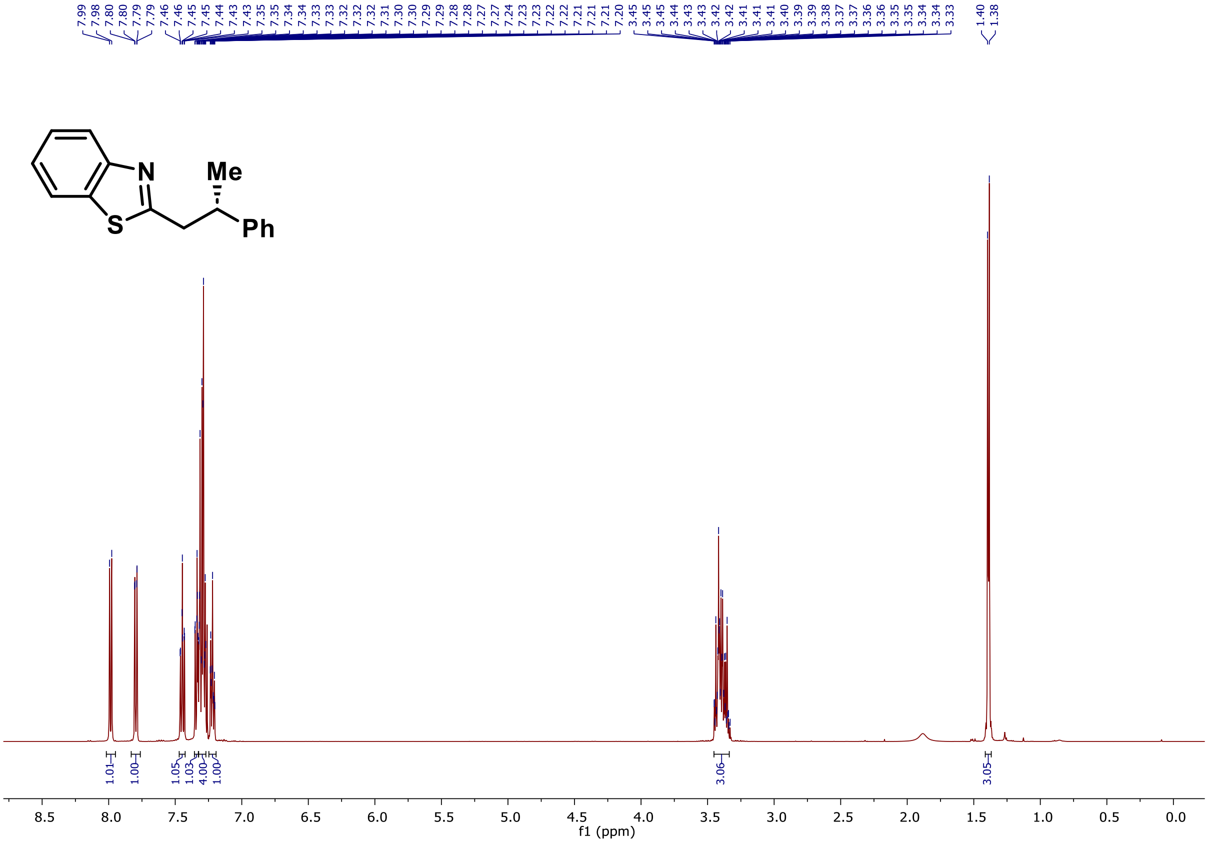

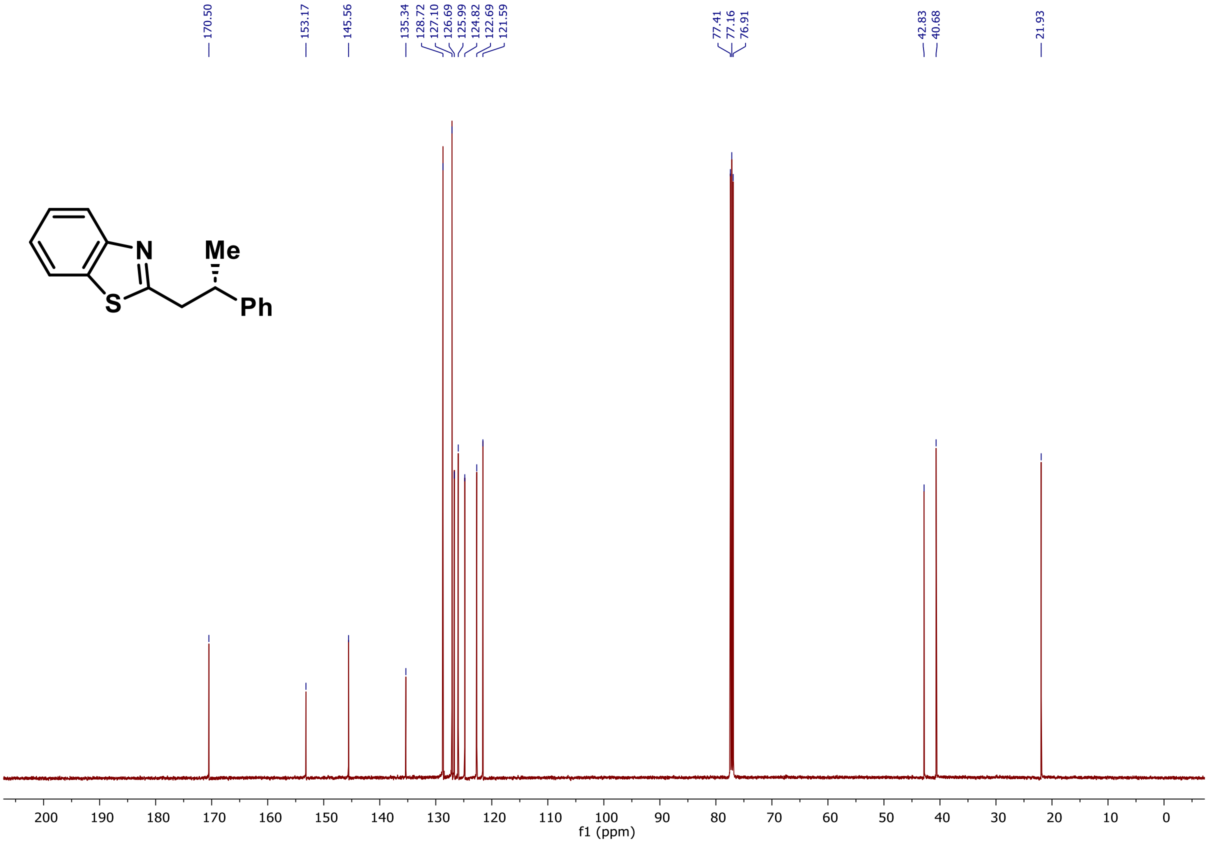

***tert*-Butyl 2-(4-phenylthiazol-2-yl)acetate (1h)**

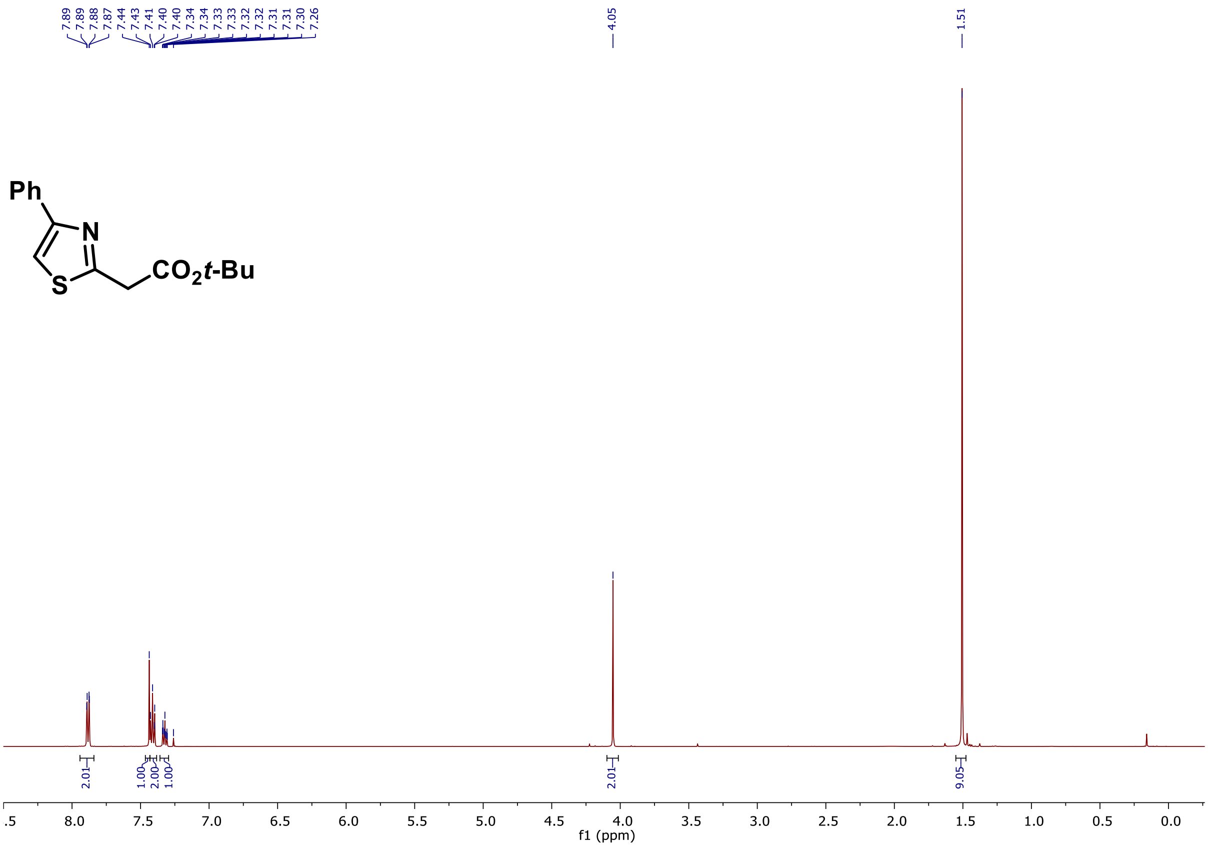

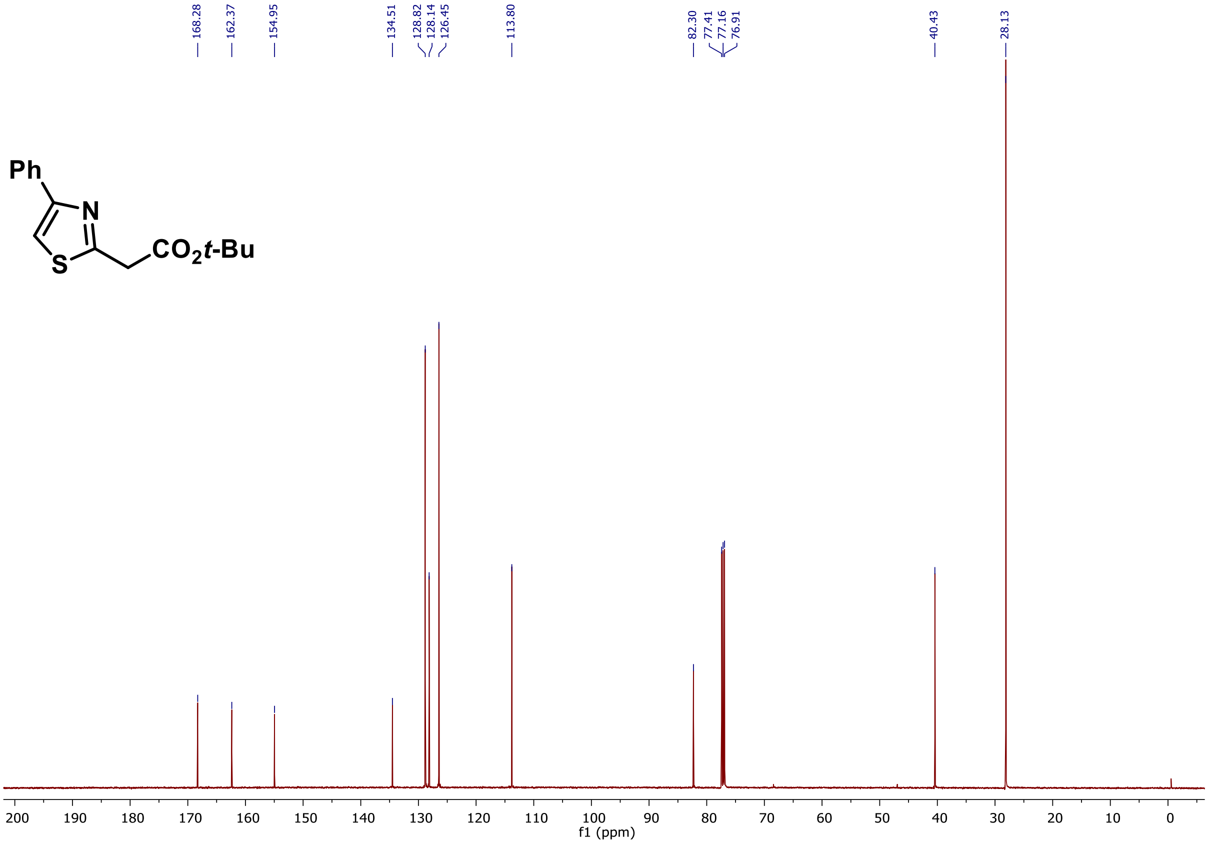

(*S*)-4-Phenyl-2-(2-phenylpropyl)thiazole (4ha)

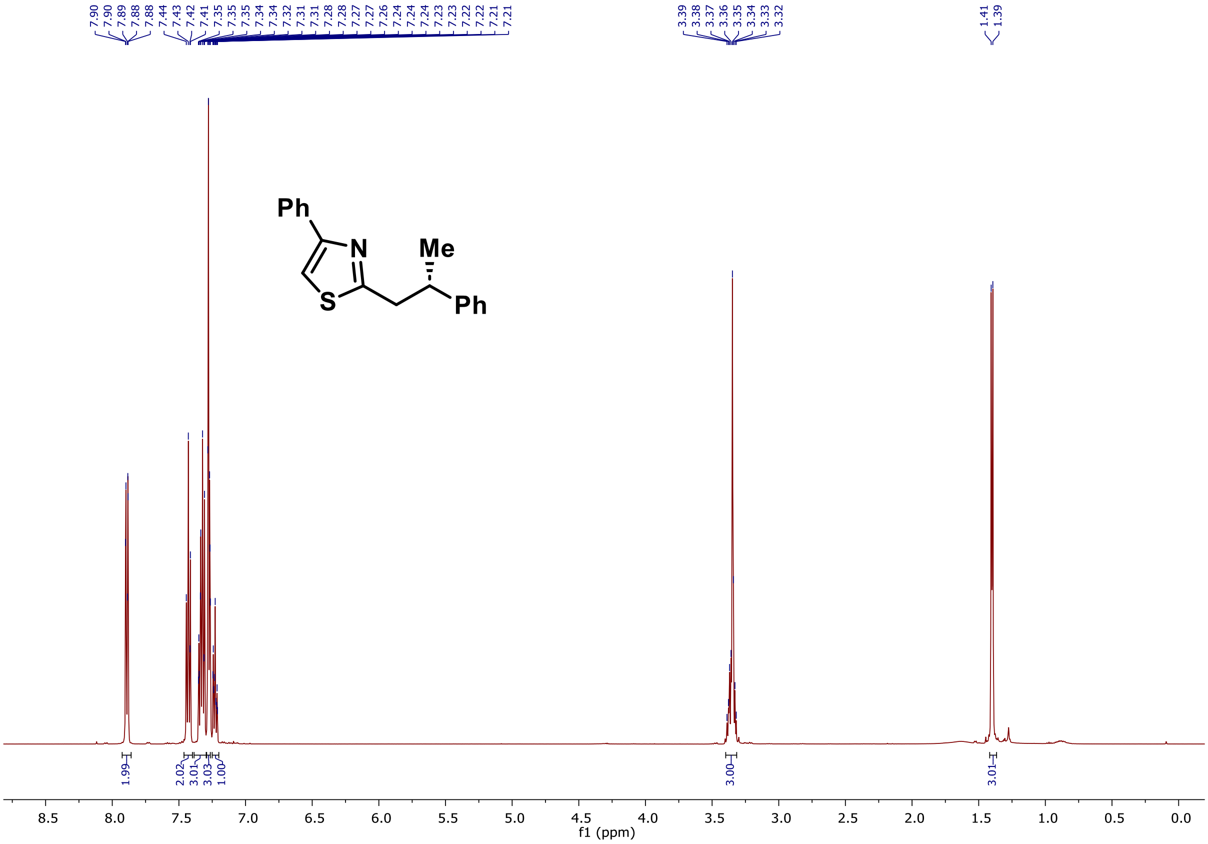

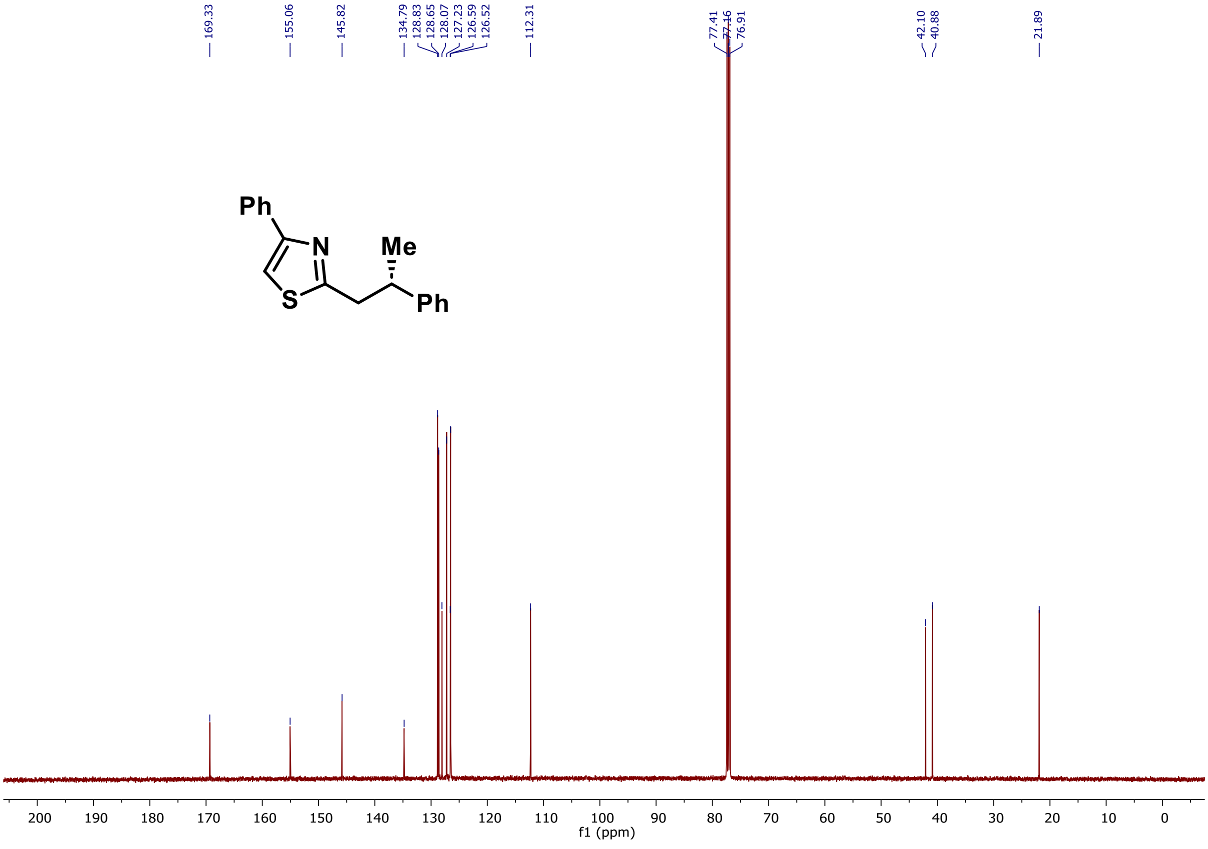

tert-Butyl 2-(thiazol-2-yl)acetate (1i)

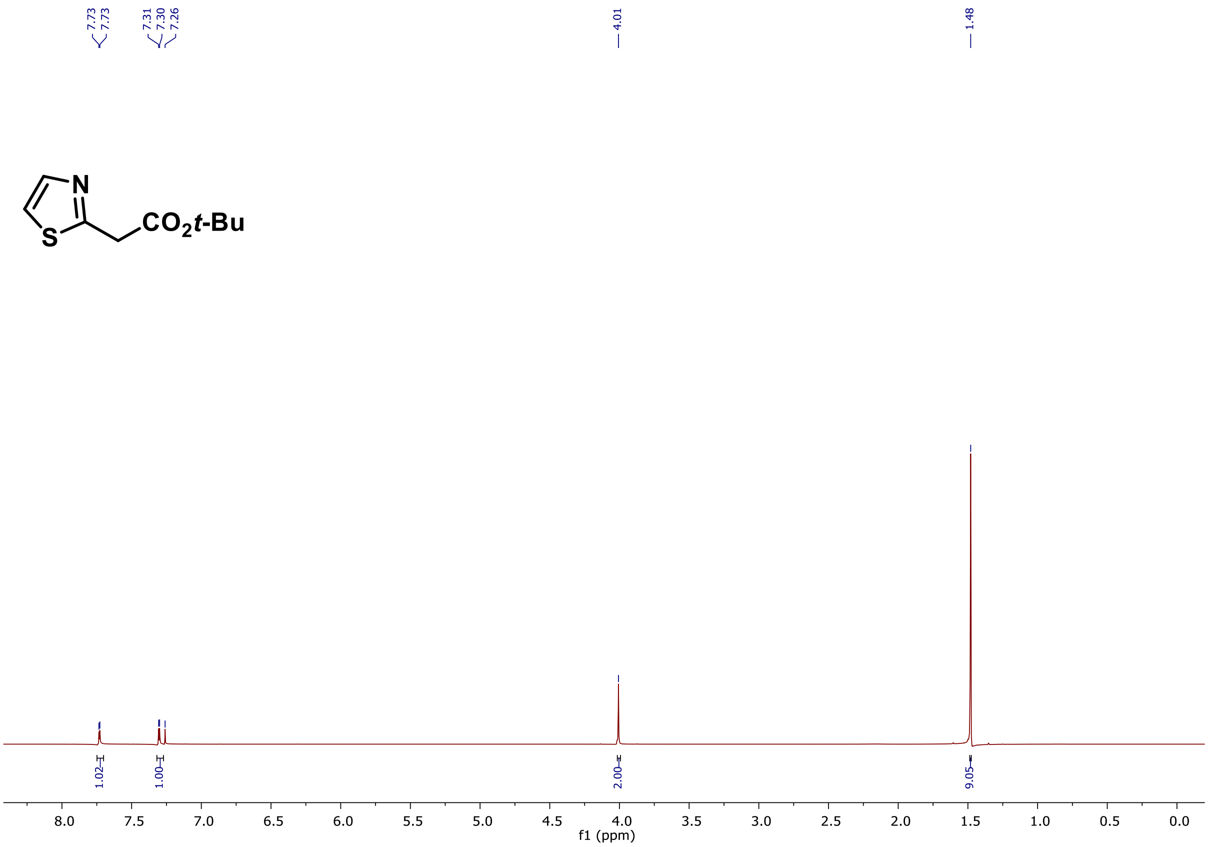

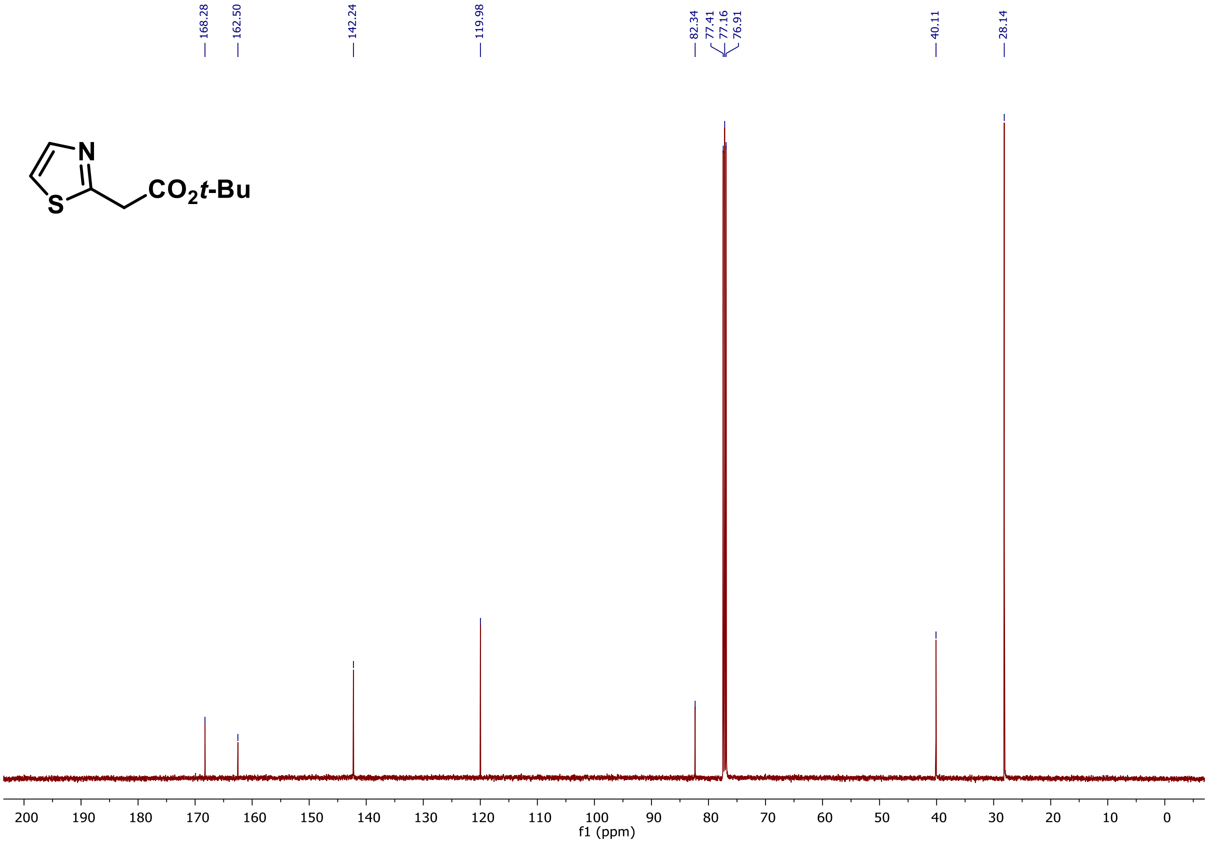

(*S*)-2-(2-Phenylpropyl)thiazole (4ia)

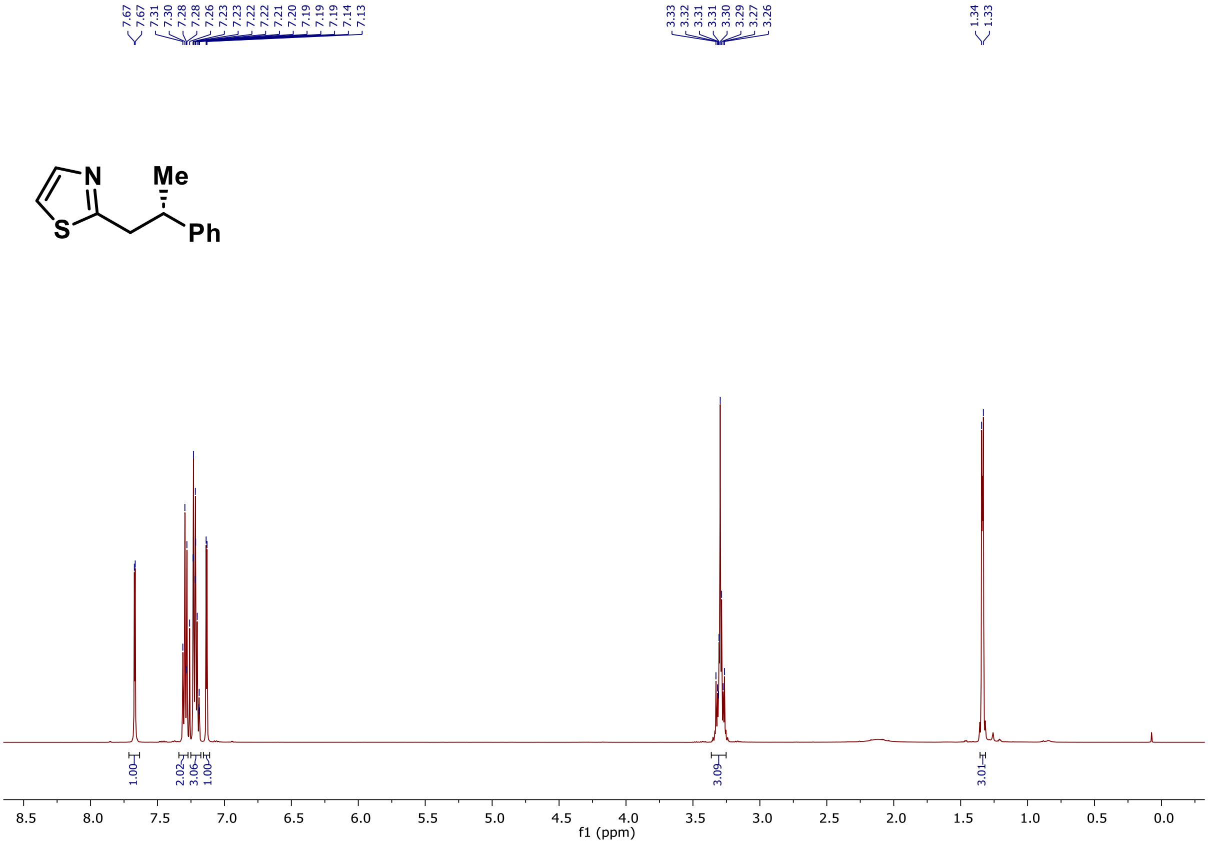

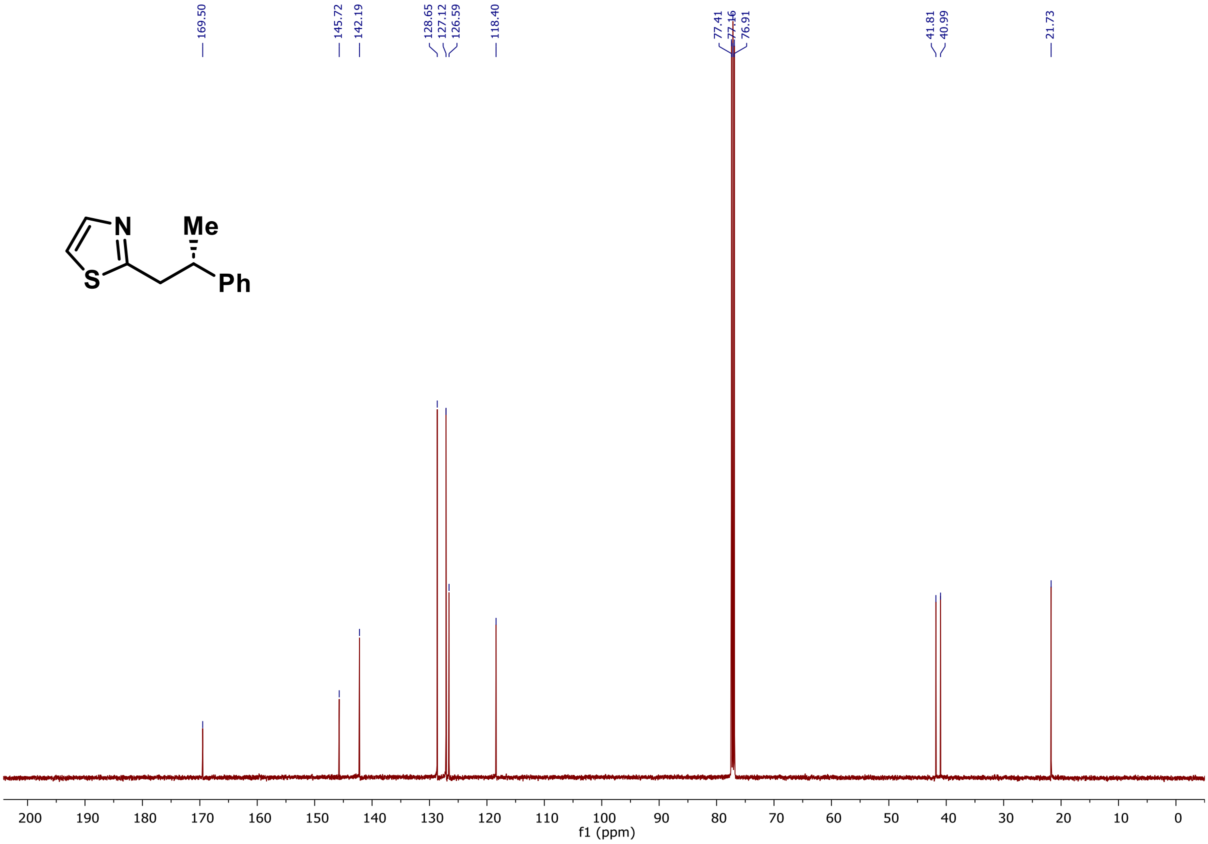

tert-Butyl 2-(benzo[*d*]oxazol-2-yl)acetate (1j)

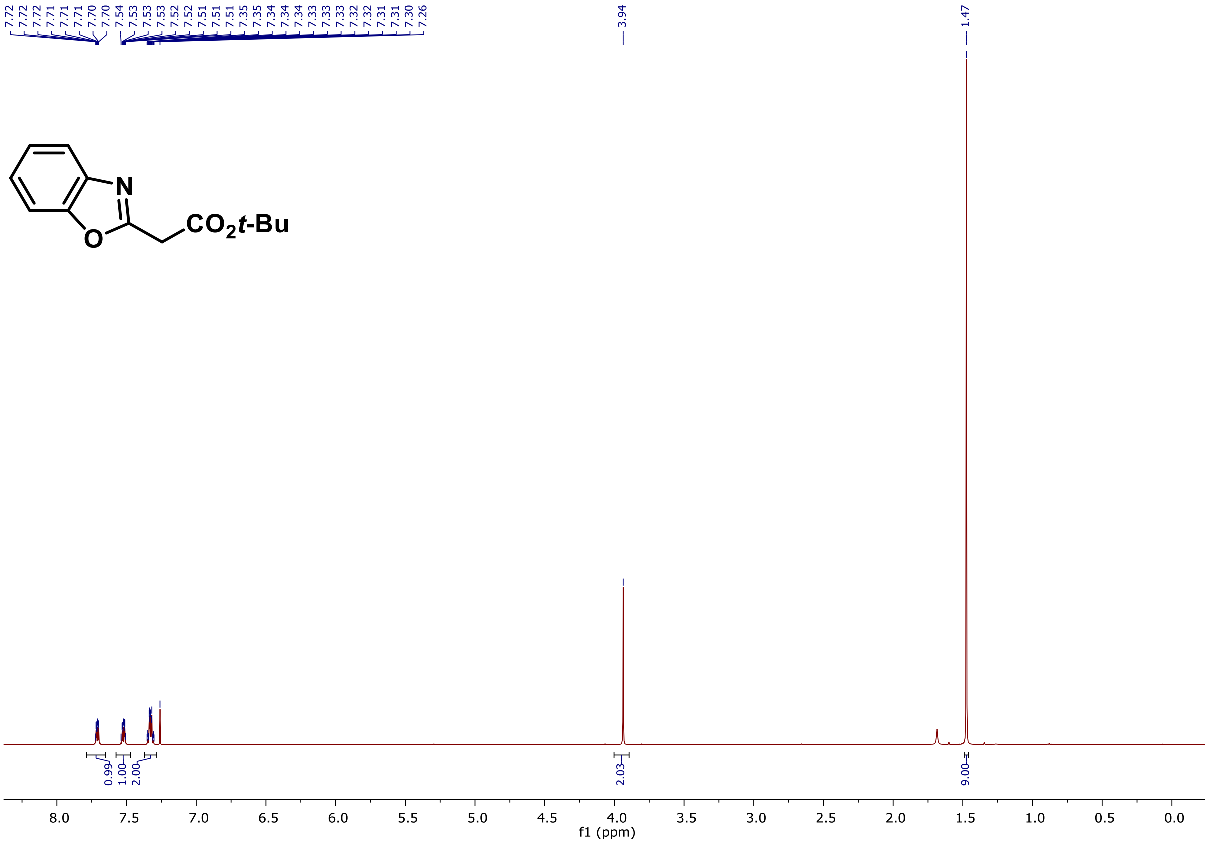

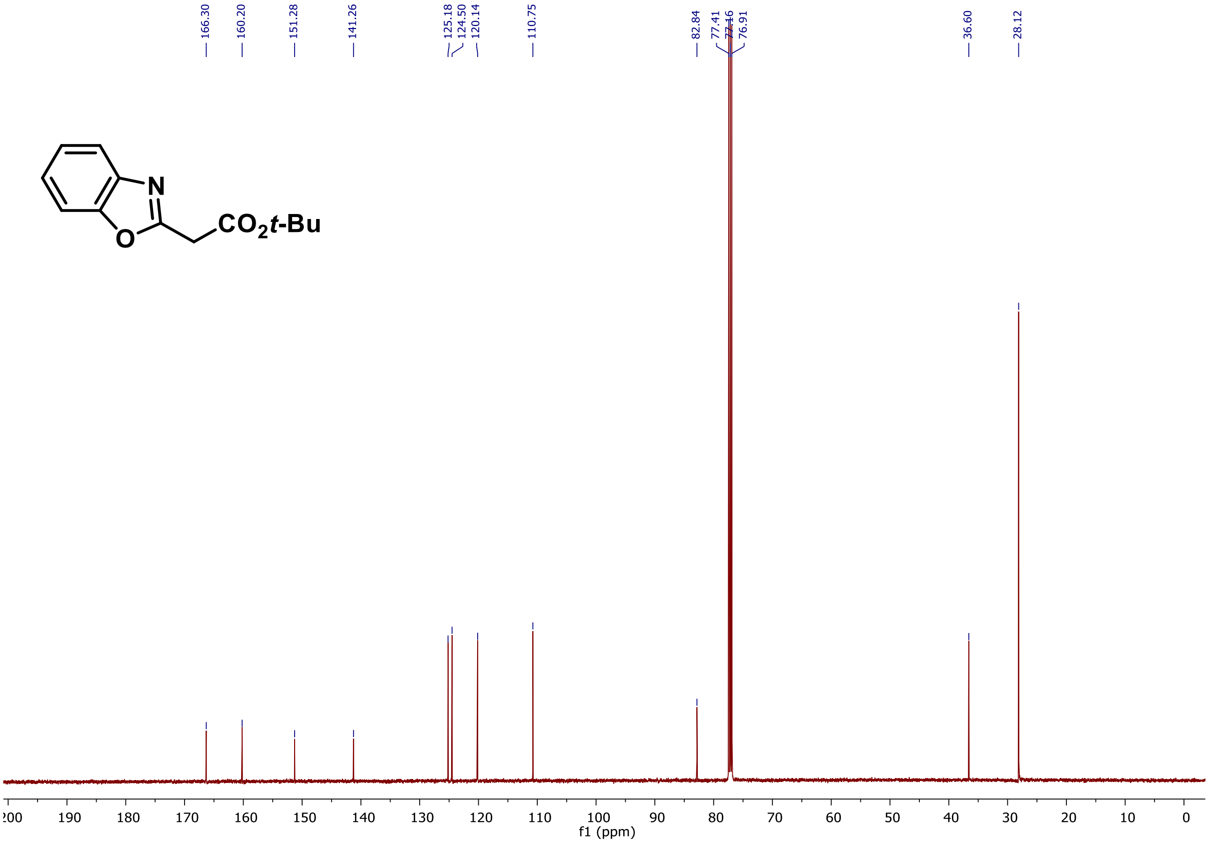

(*S*)-2-(2-Phenylpropyl)benzo[*d*]oxazole (4ja)

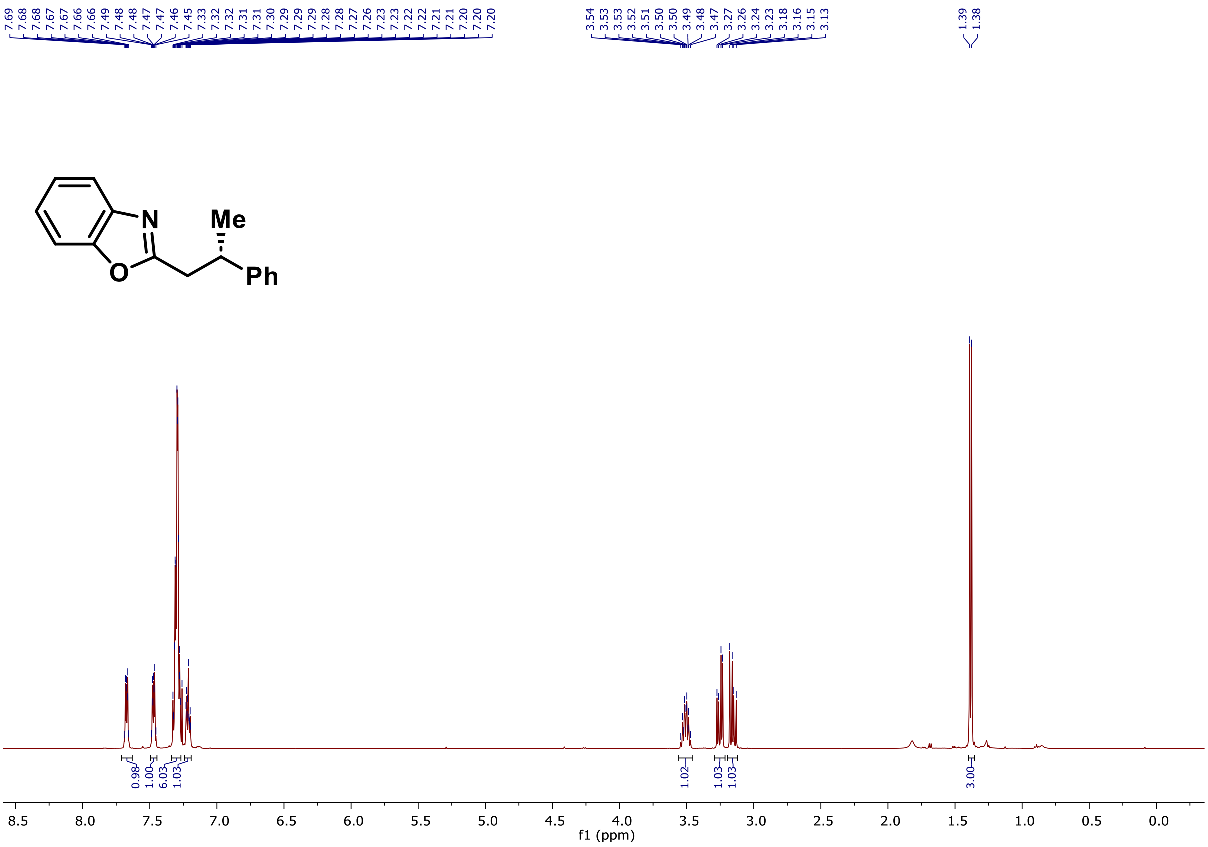

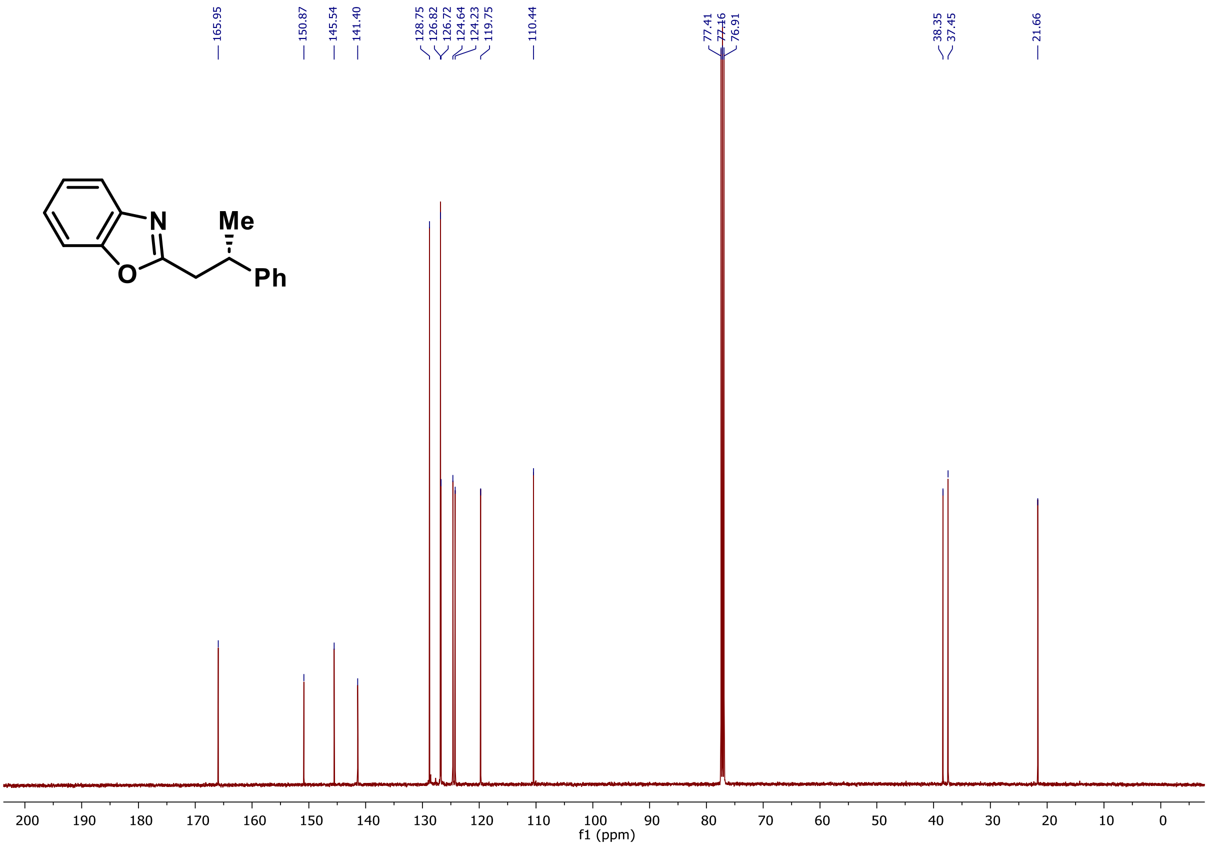

***tert*-Butyl 2-(1-methyl-1*H*-benzo[*d*]imidazol-2-yl)acetate (1k)**

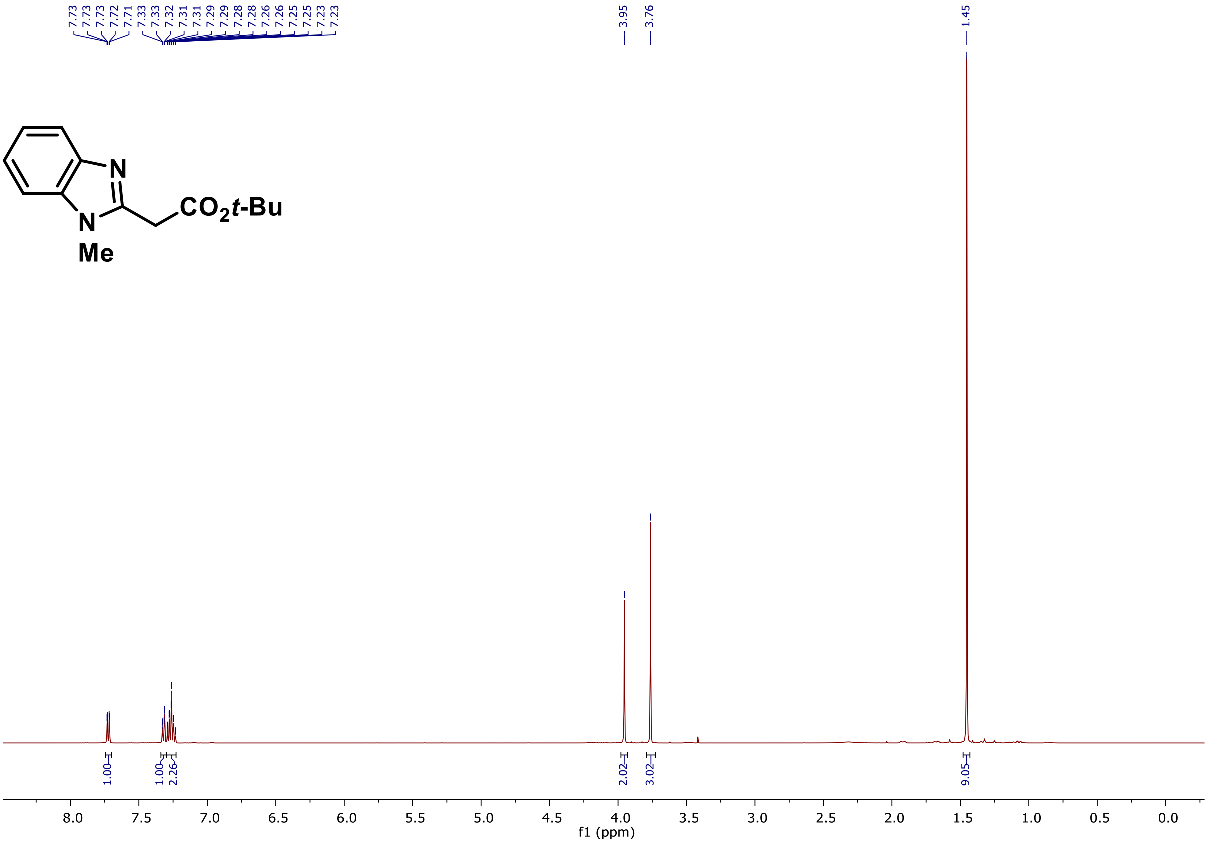

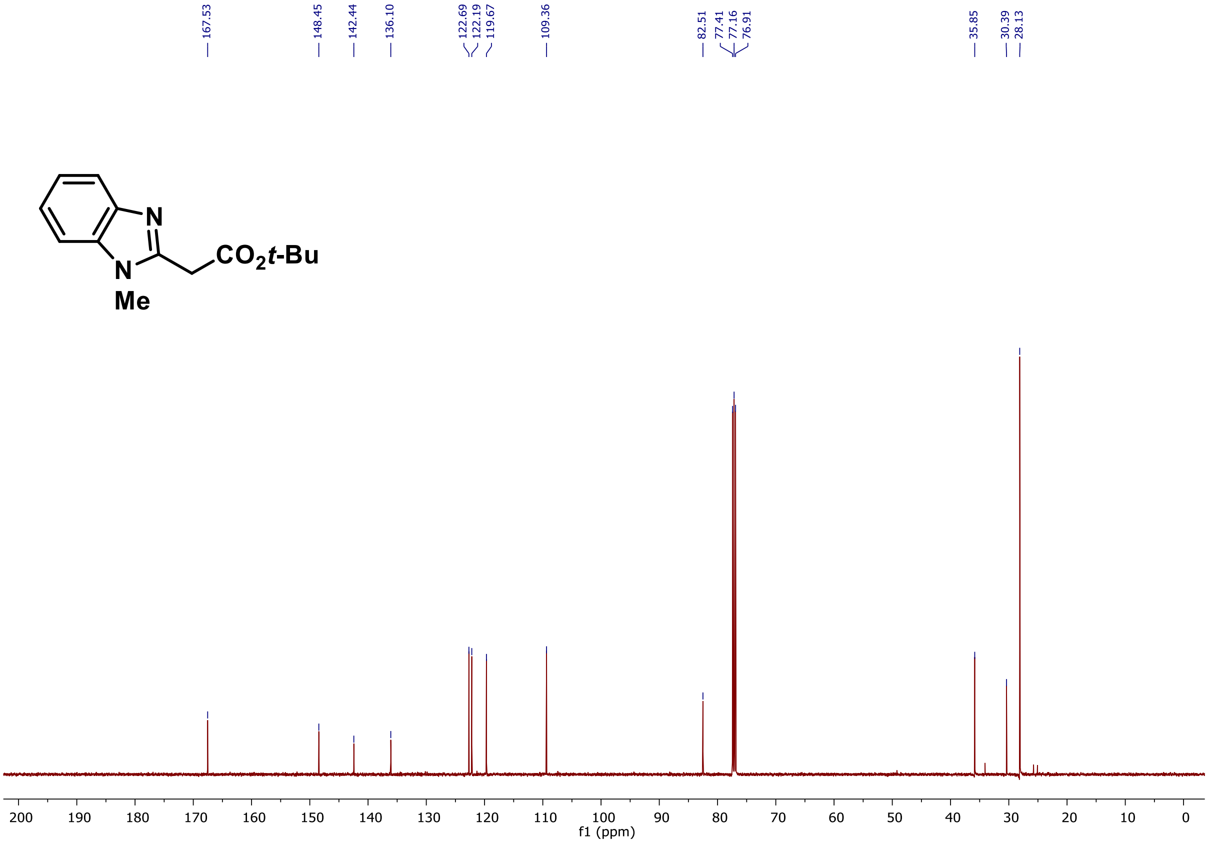

tert-Butyl (3_S_)-2-(1-methyl-1_H_-benzo[_d_]imidazol-2-yl)-3-phenylbutanoate (3ka)

Diastereomer 1

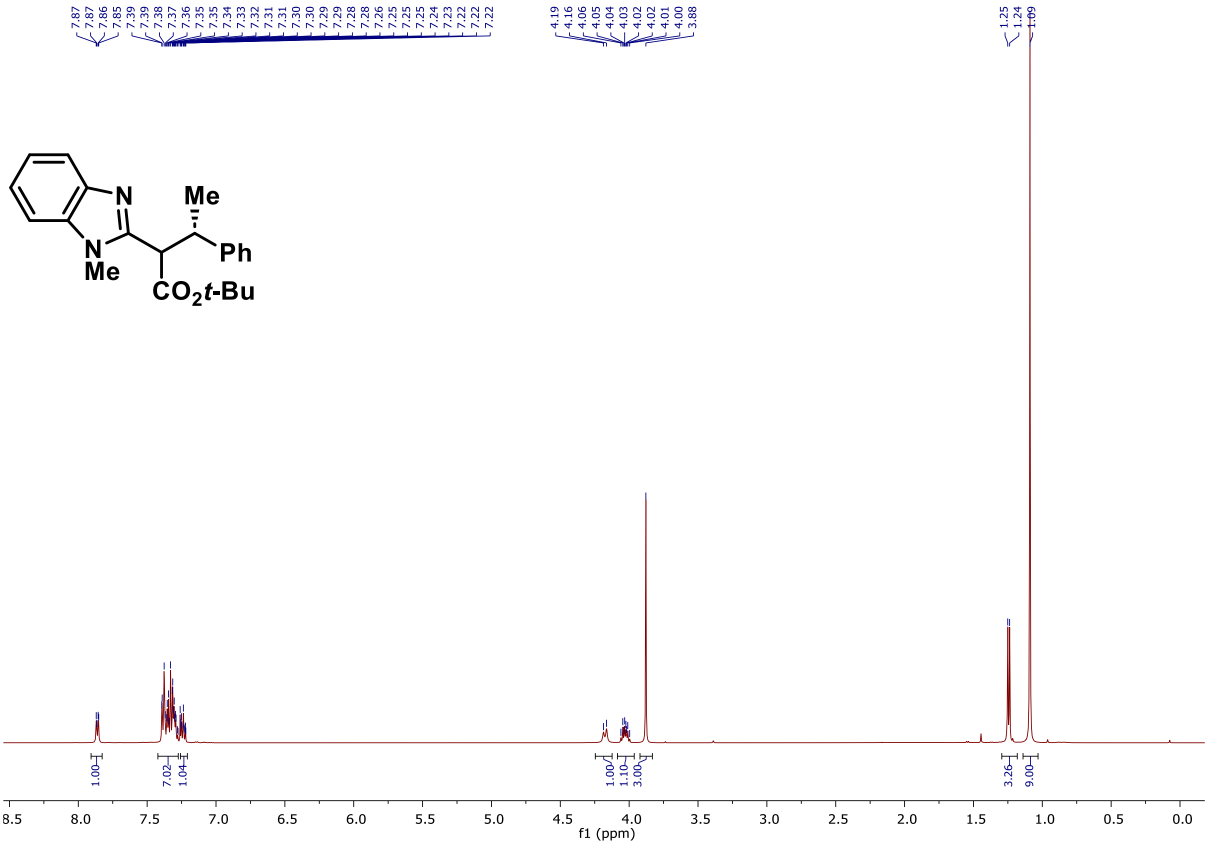

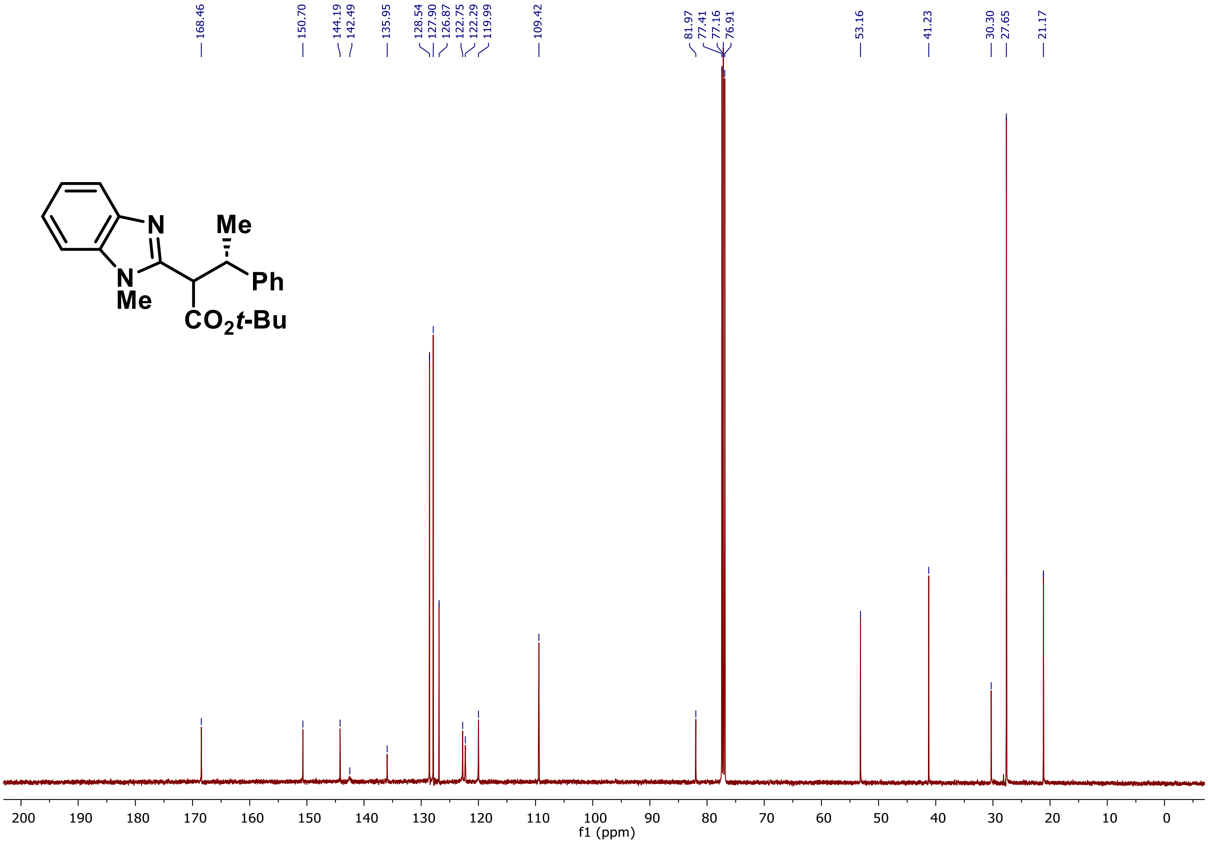

Diastereomer 2

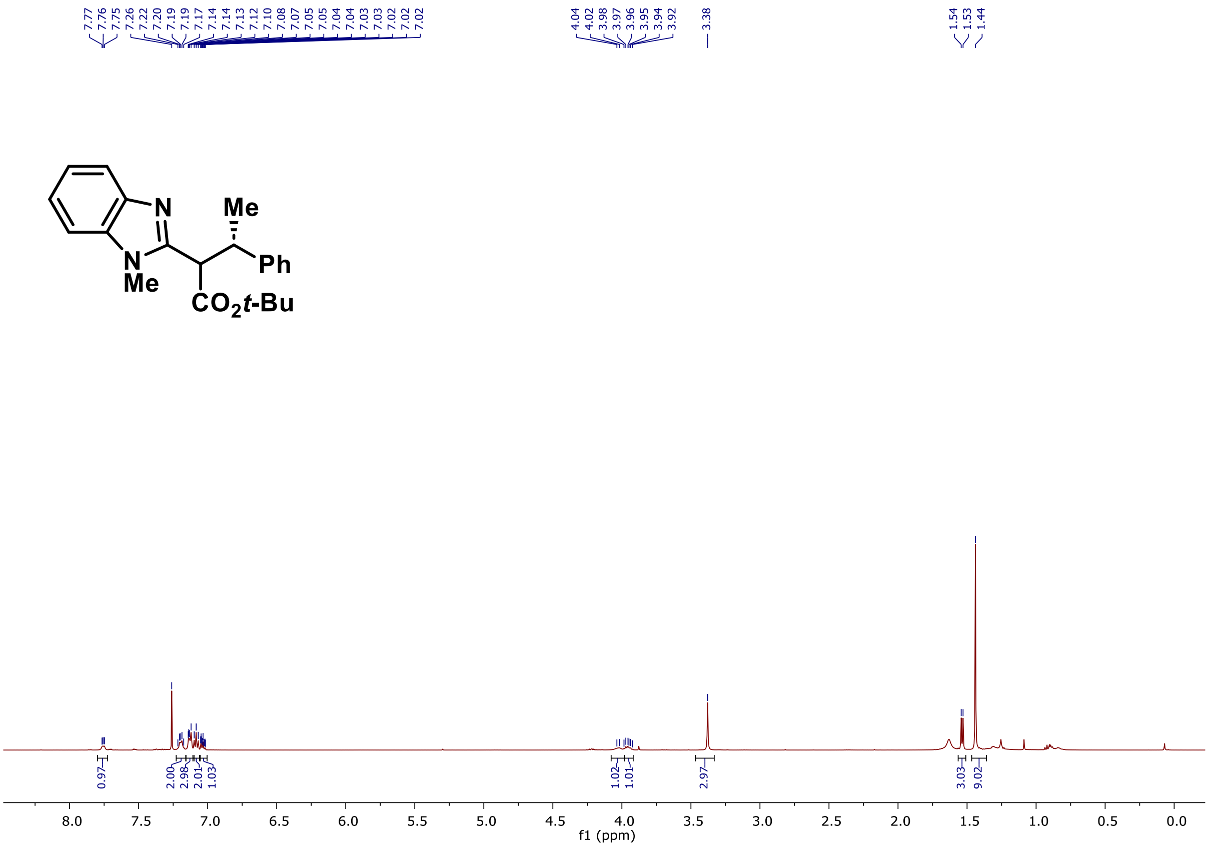

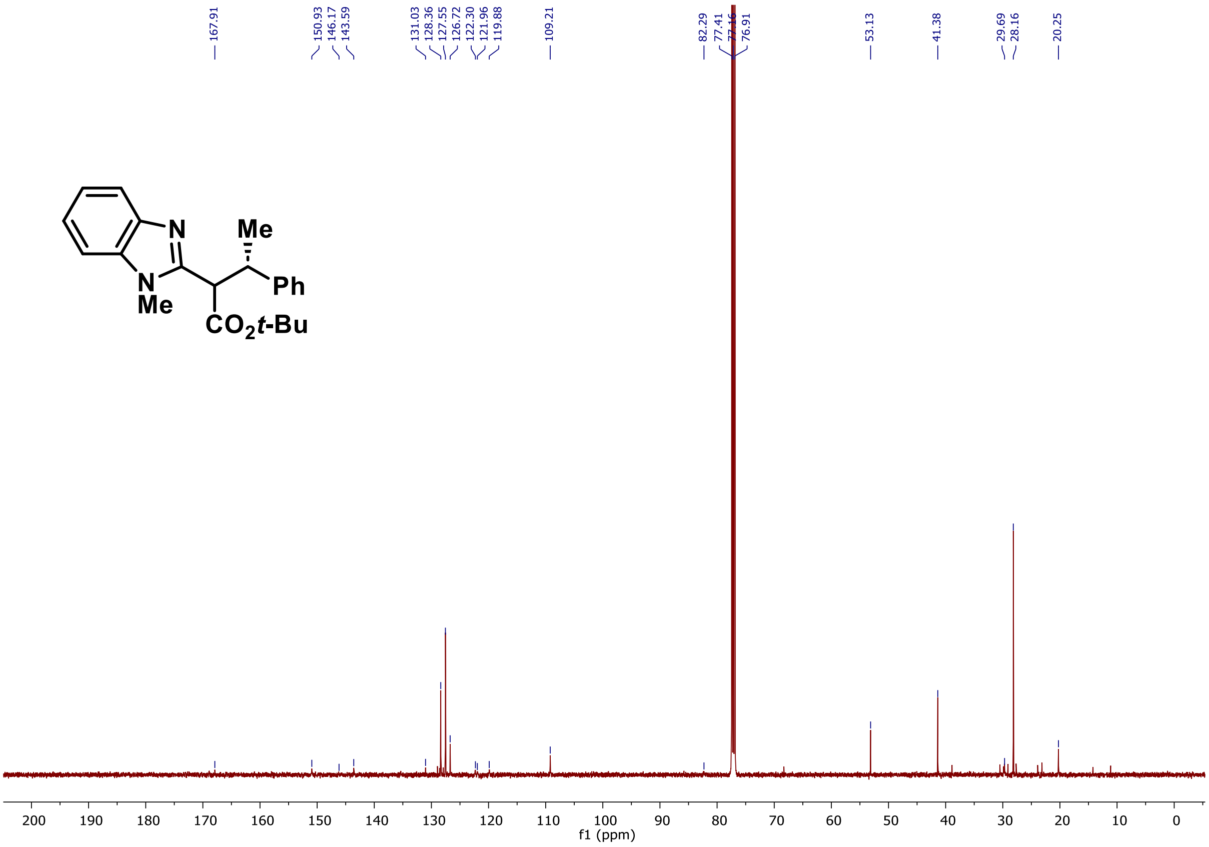

(*S*)-1-Methyl-2-(2-phenylpropyl)-1*H*-benzo[*d*]imidazole (4ka)

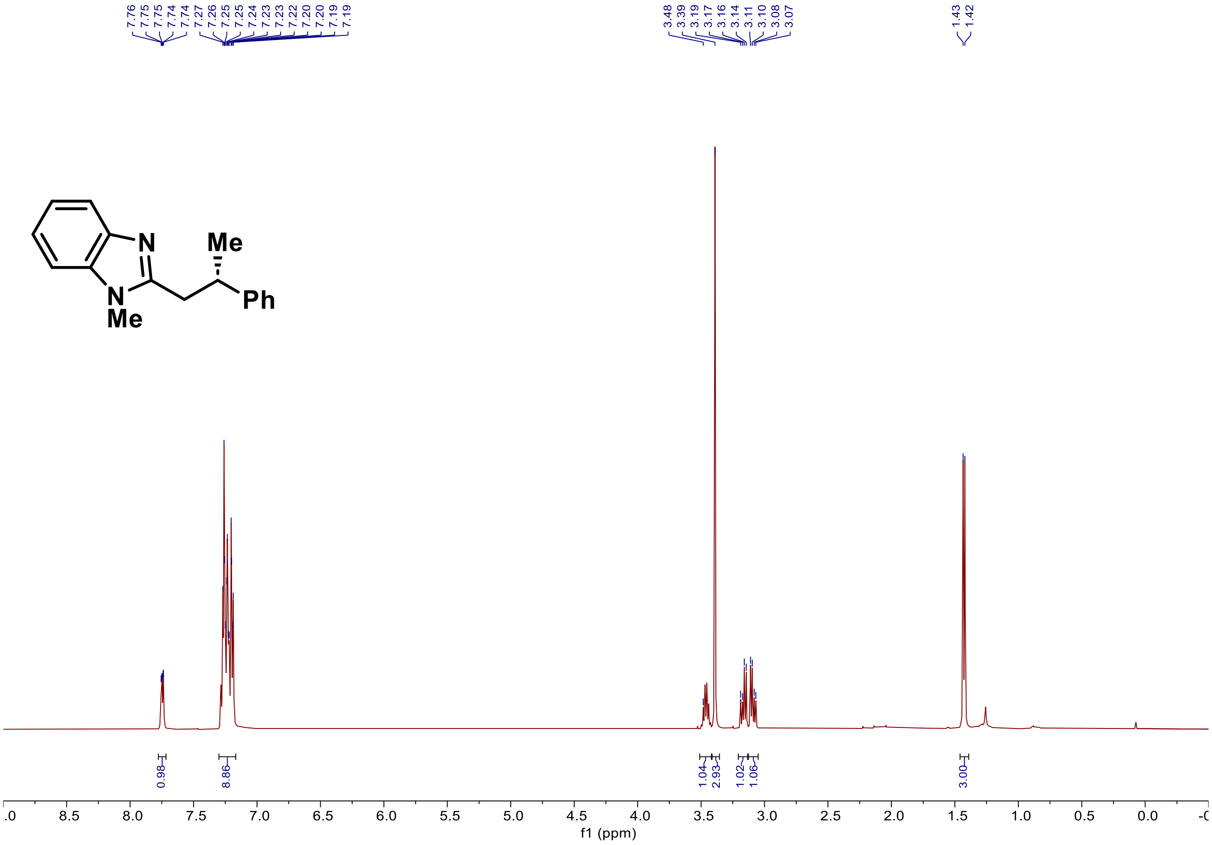

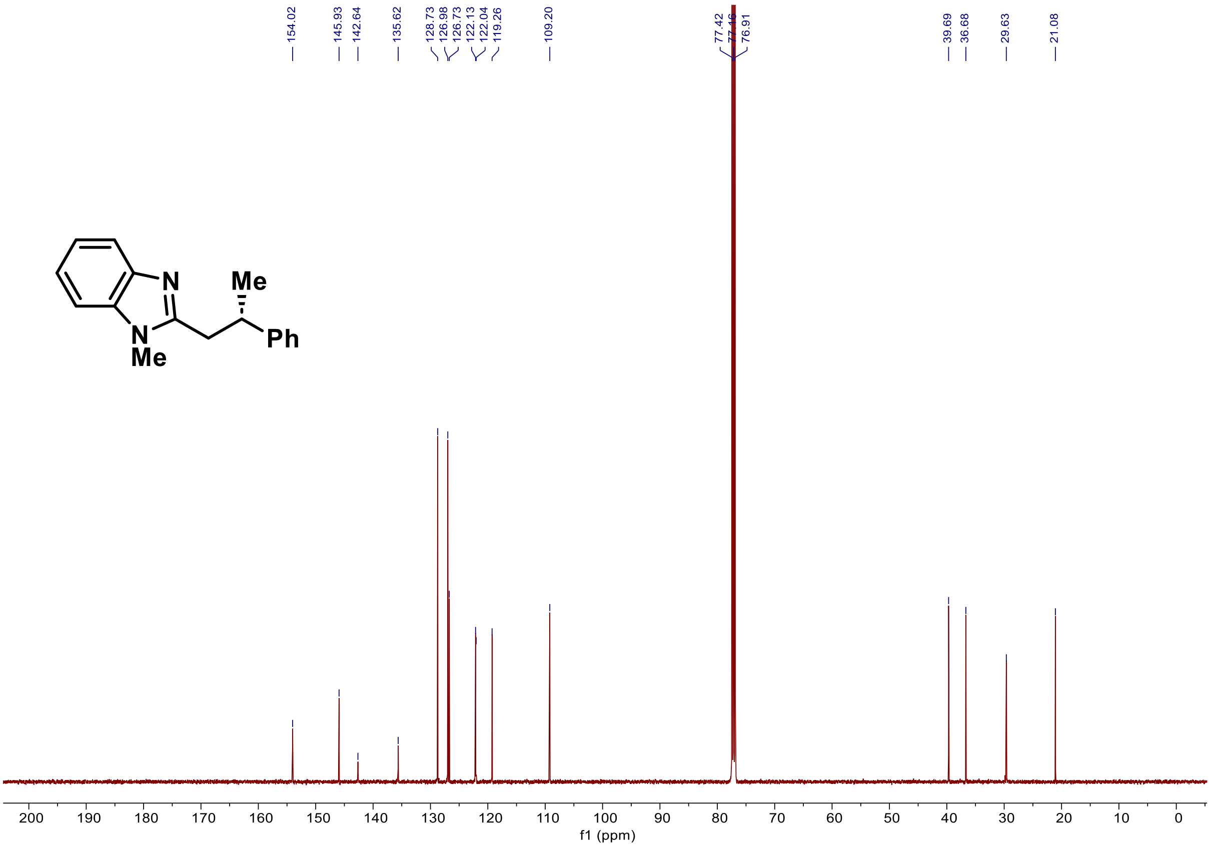

(*S*)-5-Bromo-2-(2-phenylpropyl)pyrimidine (4la)

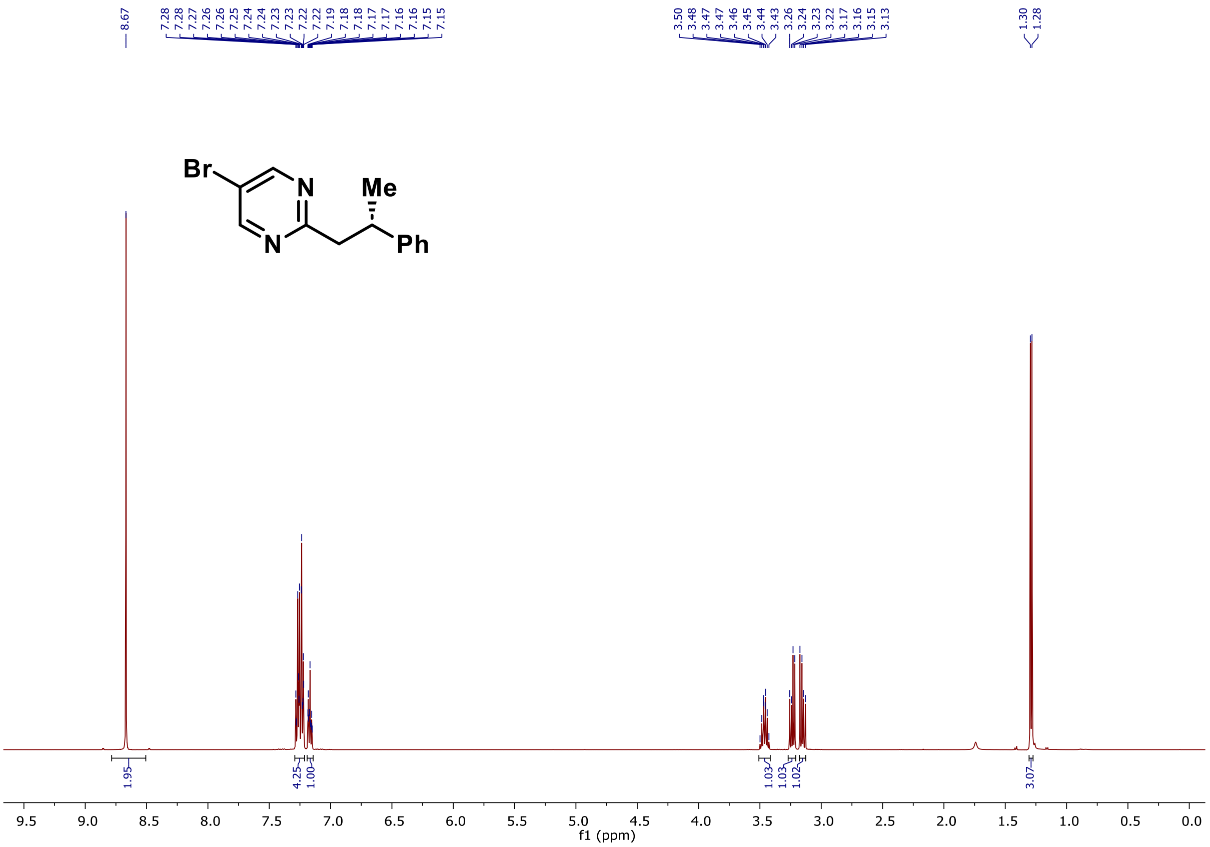

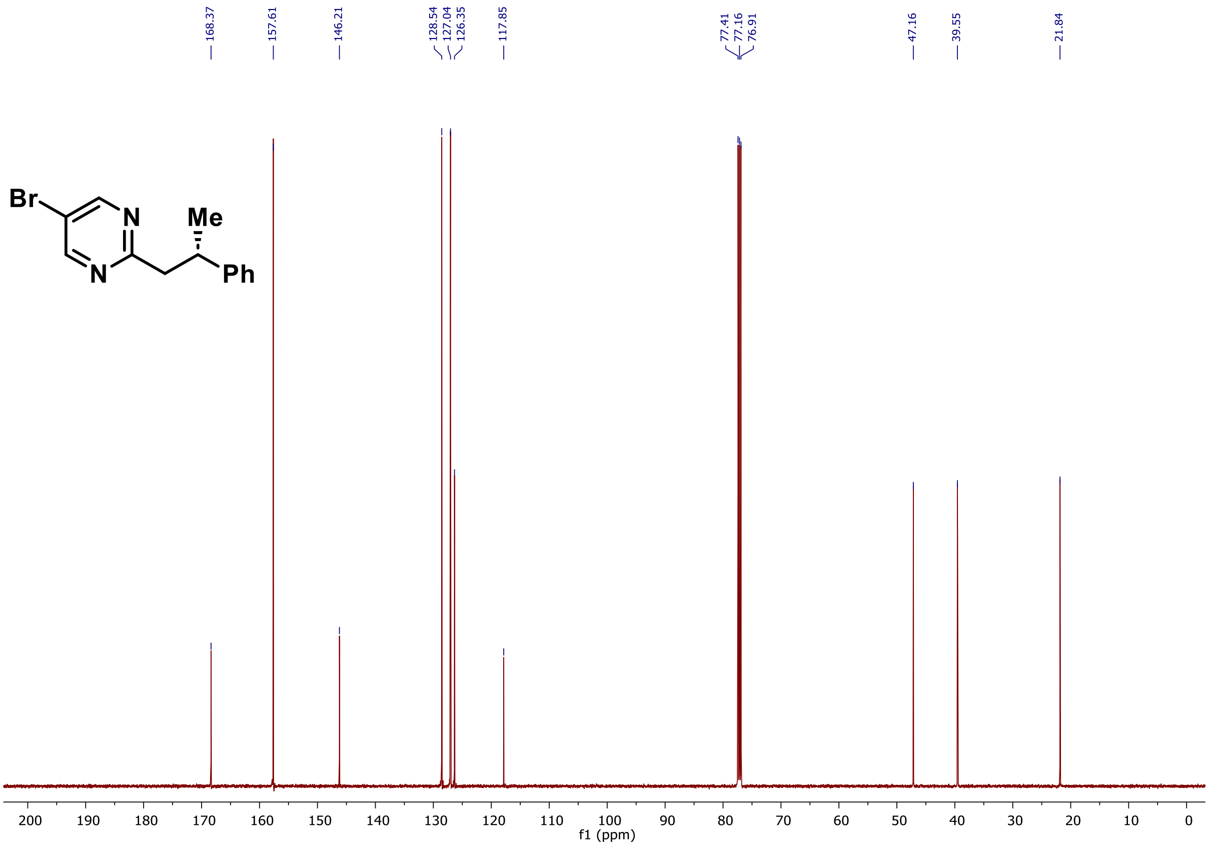

(*S*)-2,4-Dimethoxy-6-(2-phenylpropyl)-1,3,5-triazine (4ma)

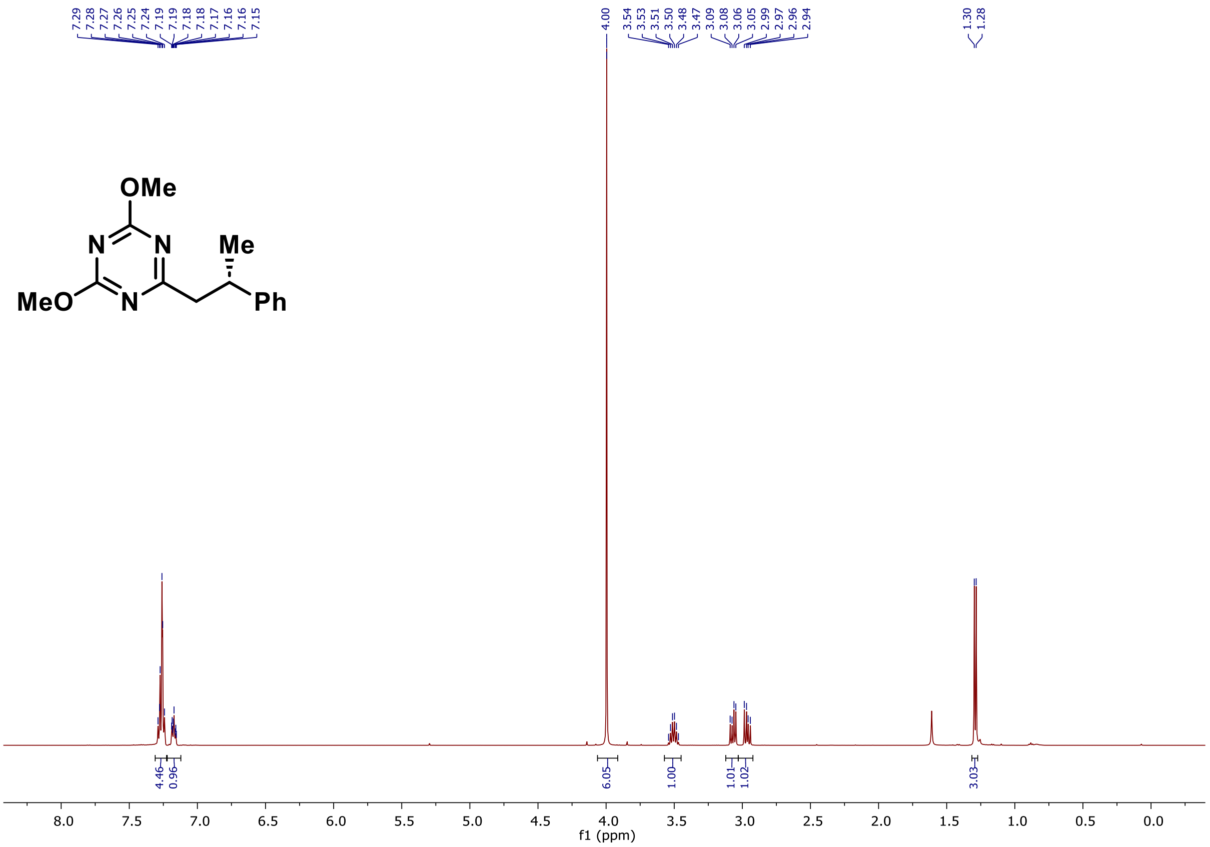

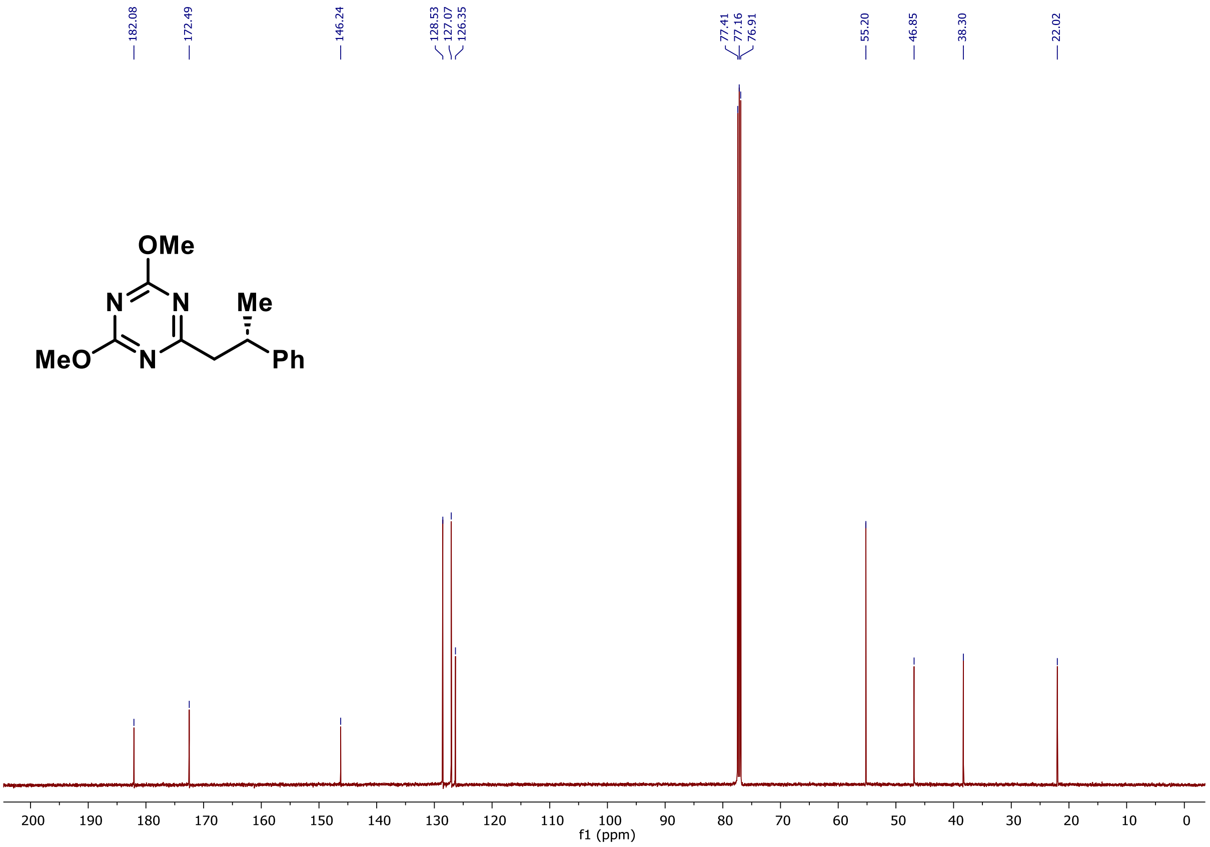

(*S*)-1-(2-Phenylpropyl)isoquinoline (4na)

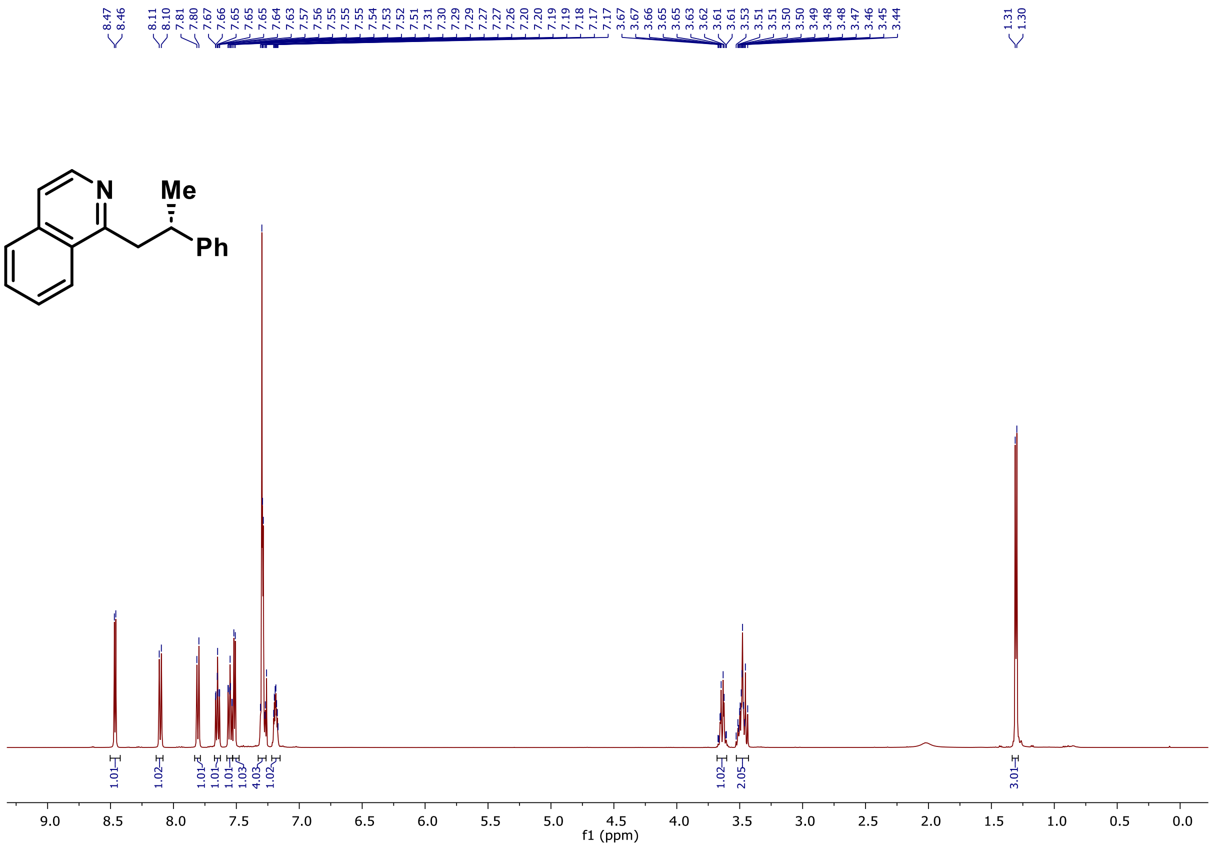

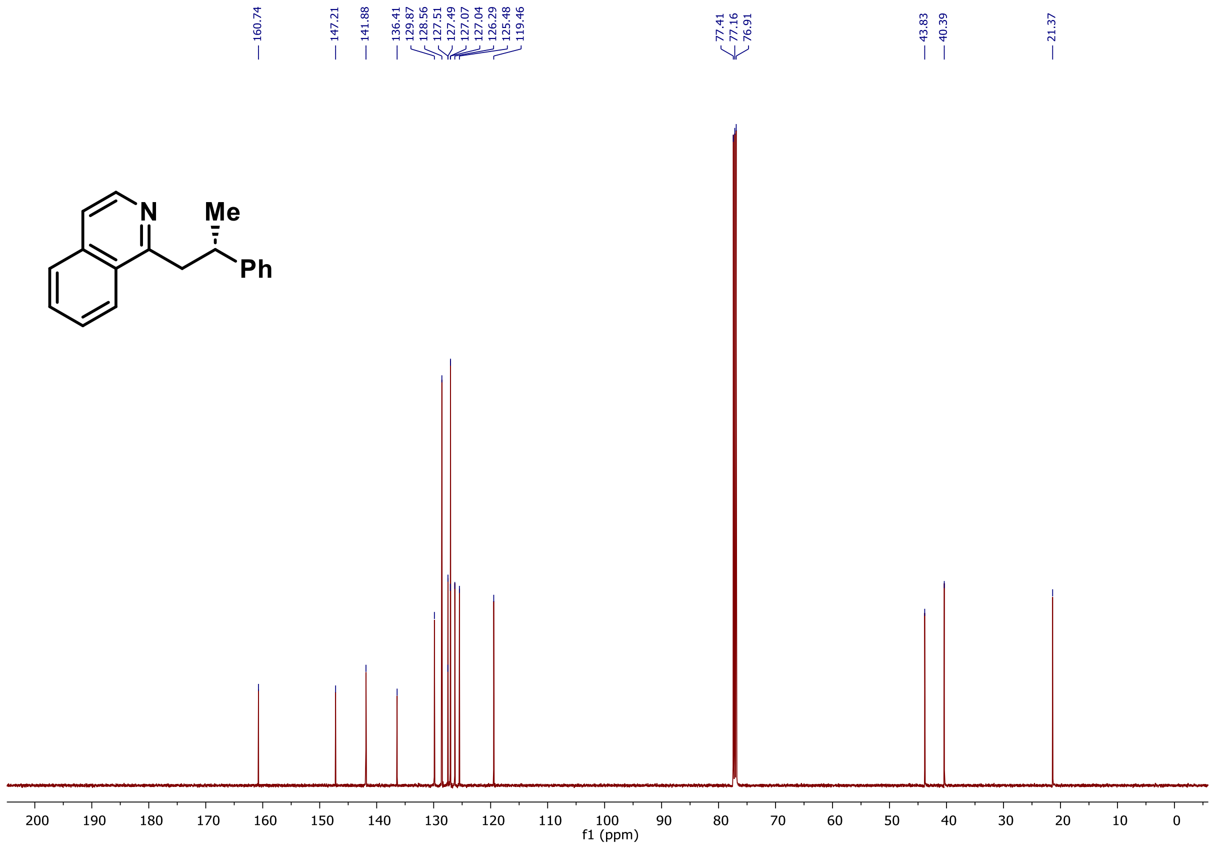

(S)-2-(2-Phenylpropyl)quinoline (4oa)

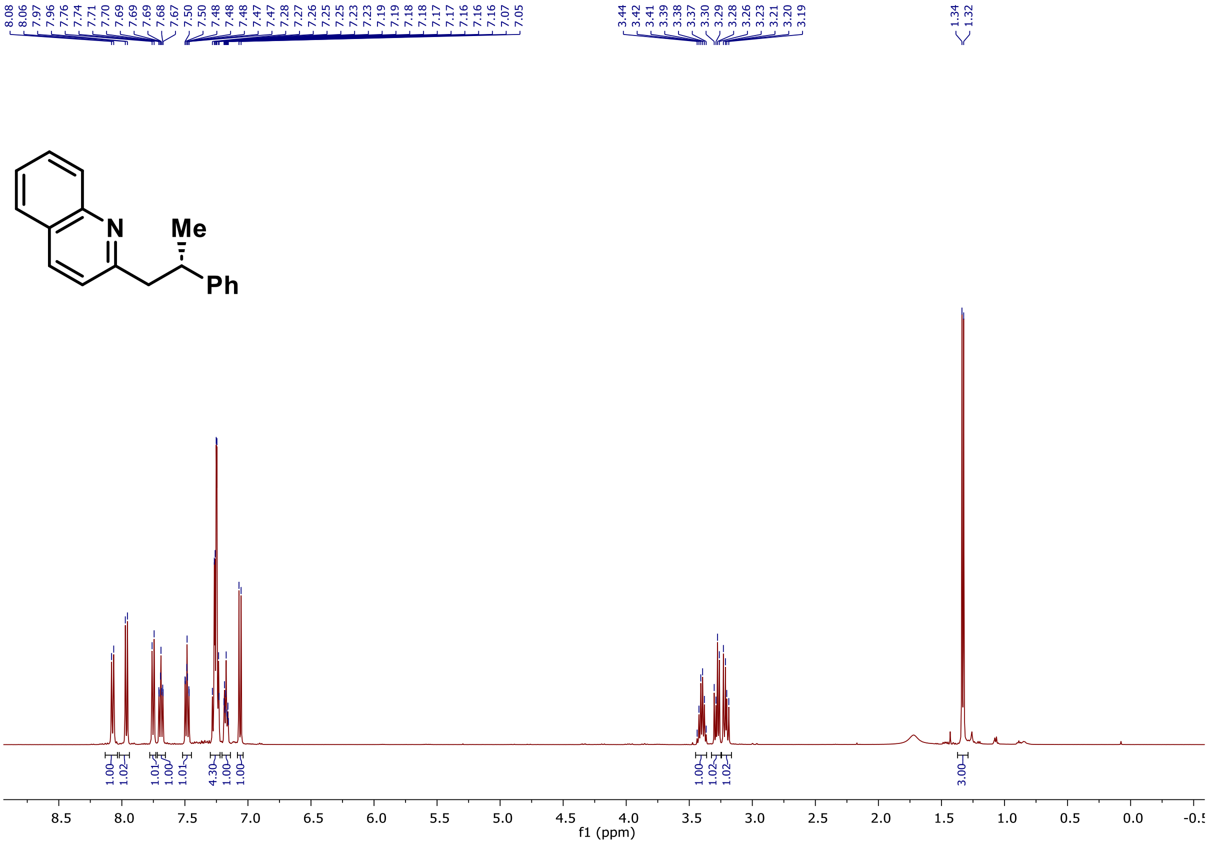

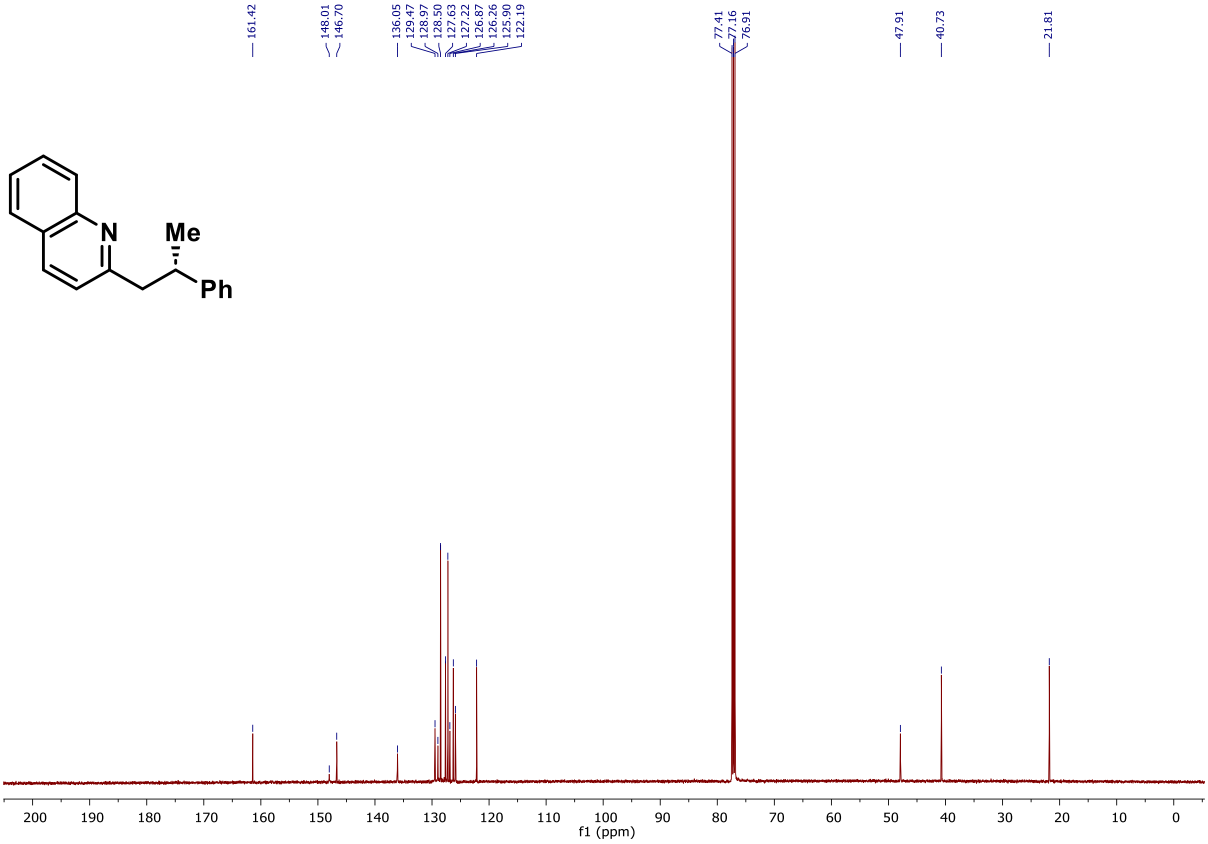

***tert*-Butyl 2-(5-chlorobenzo[*d*]thiazol-2-yl)acetate (1p)**

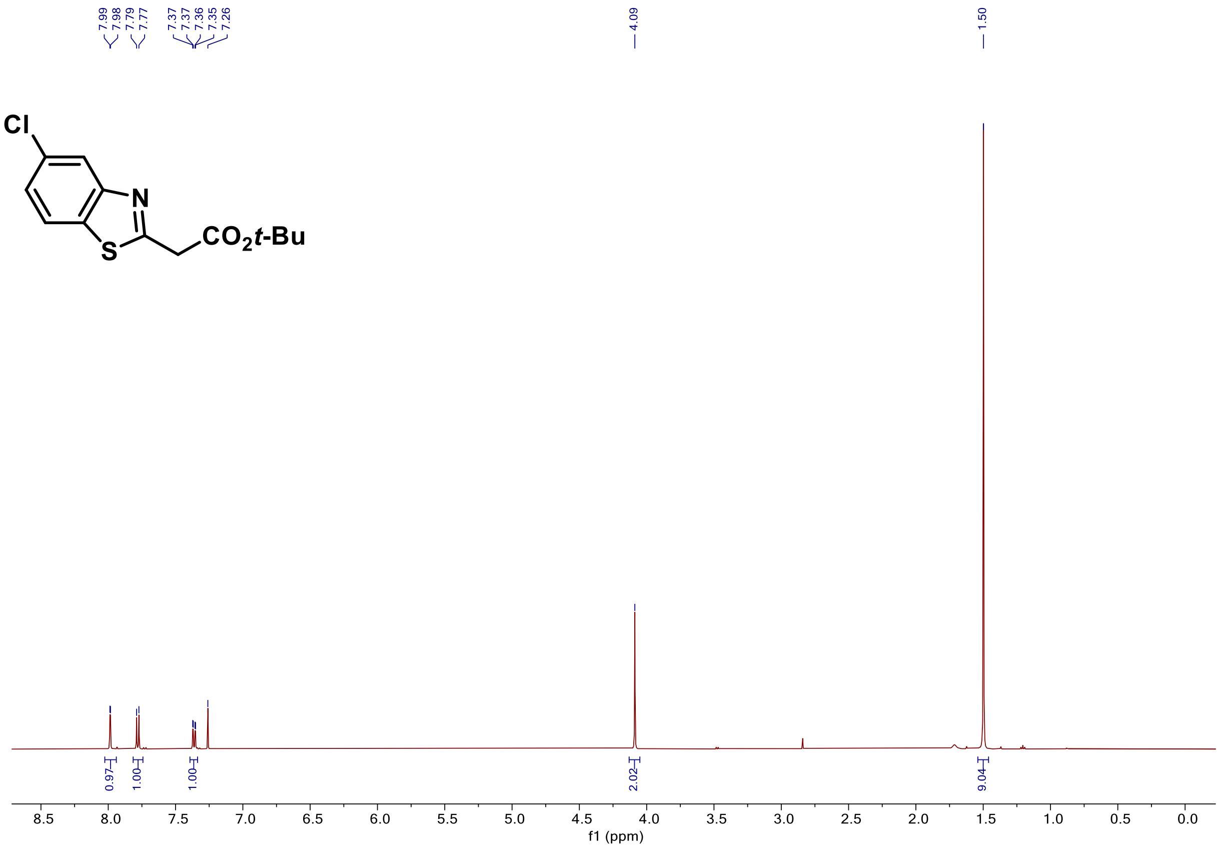

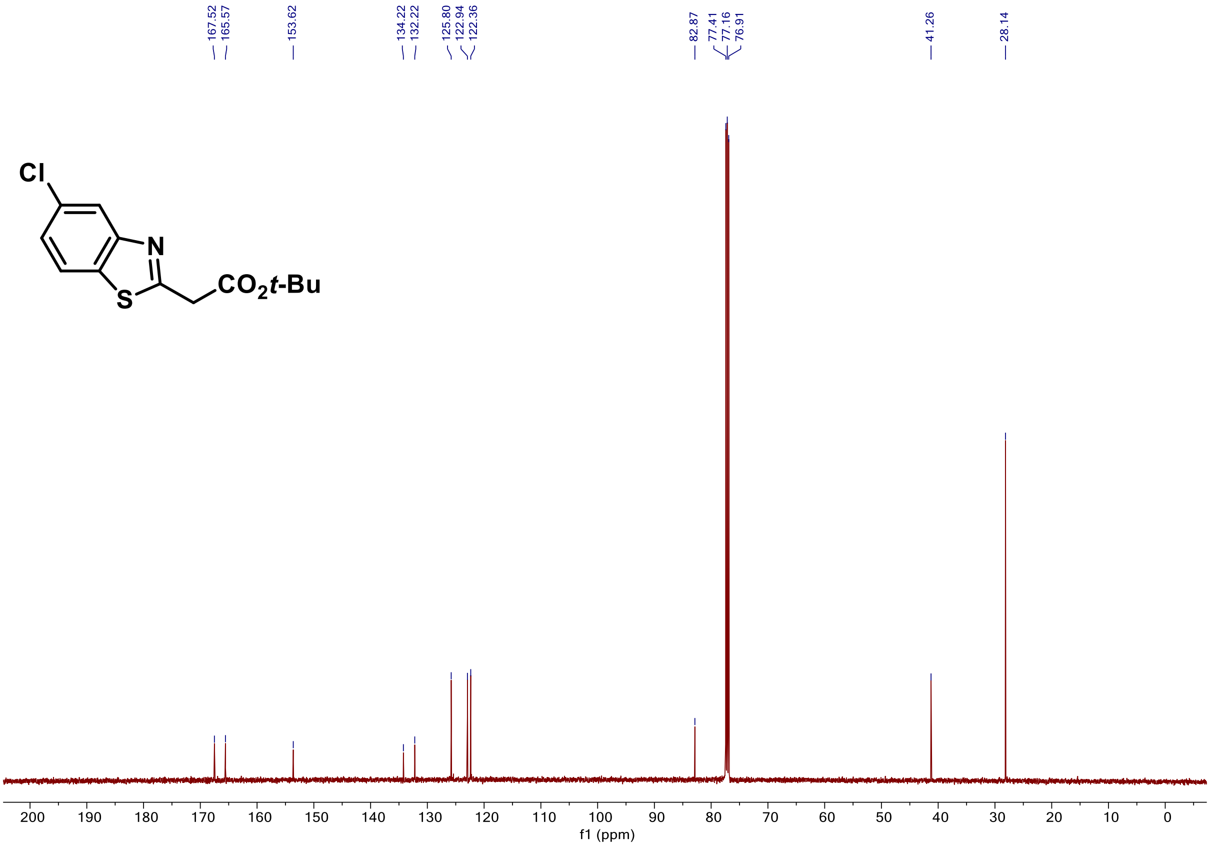

(*S*)-5-Chloro-2-(2-phenylpropyl)benzo[*d*]thiazole (4pa)

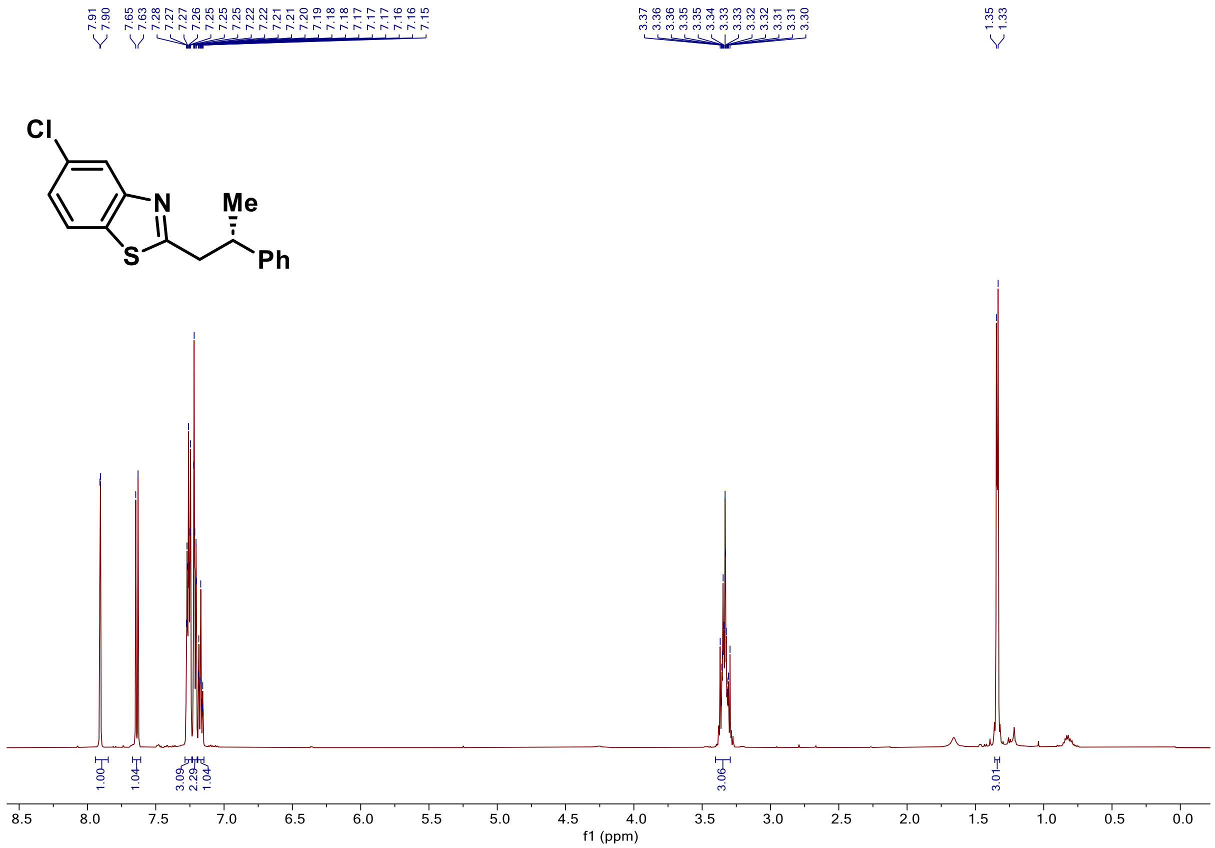

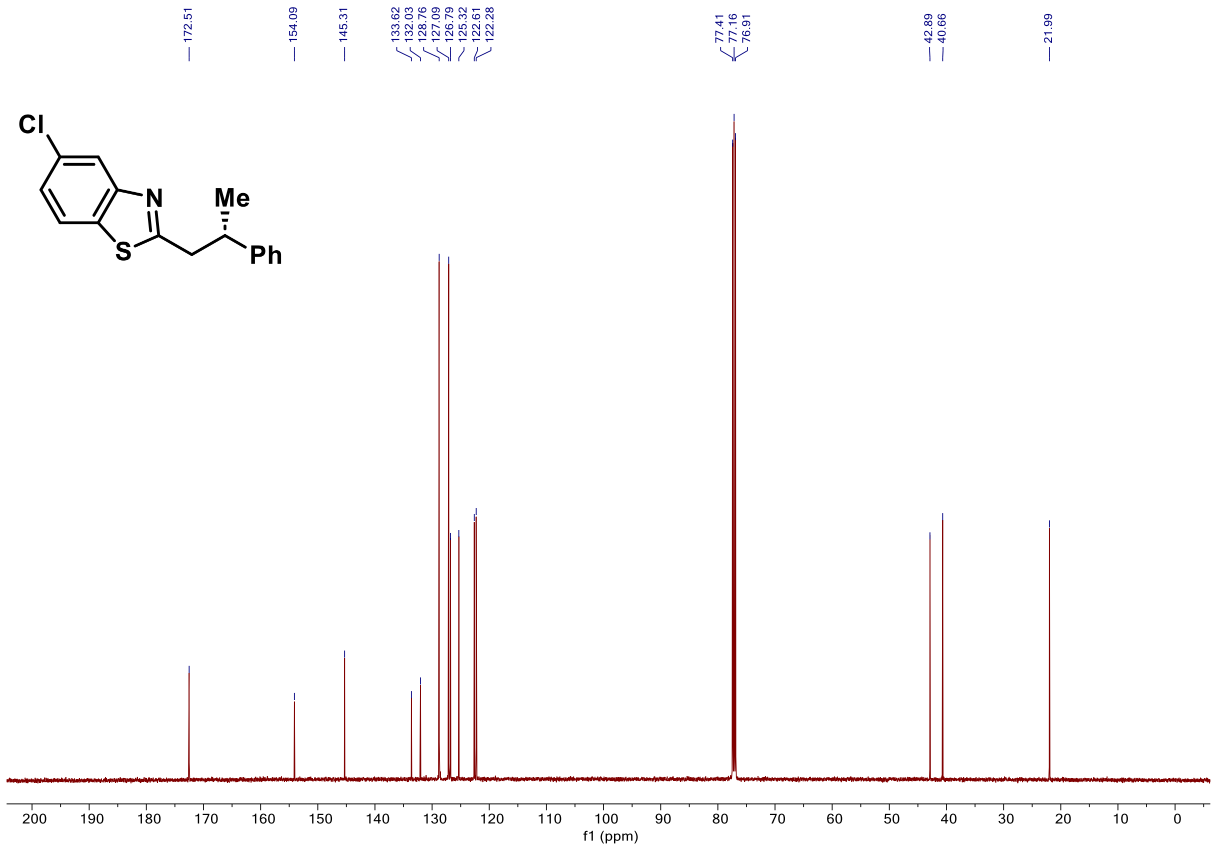

***tert*-Butyl 2-(5-methoxybenzo[*d*]thiazol-2-yl)acetate (1q)**

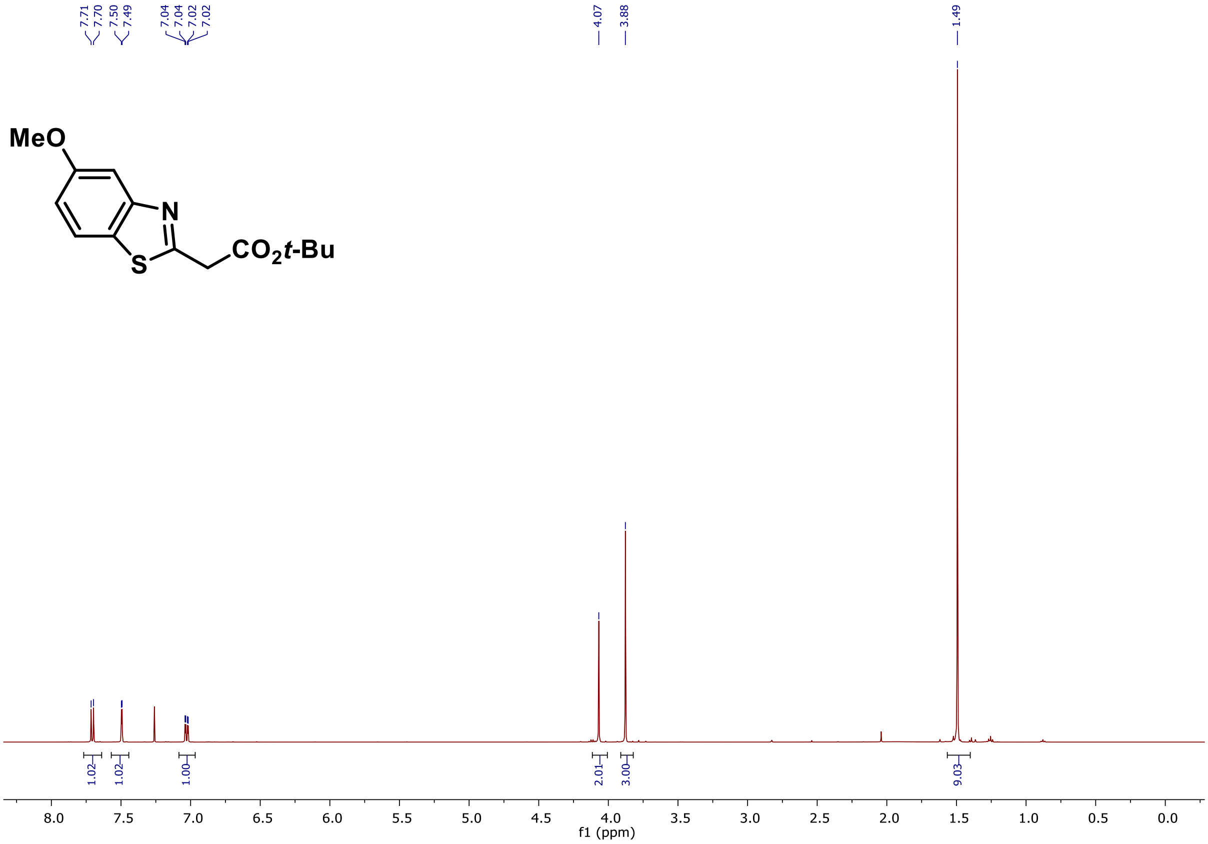

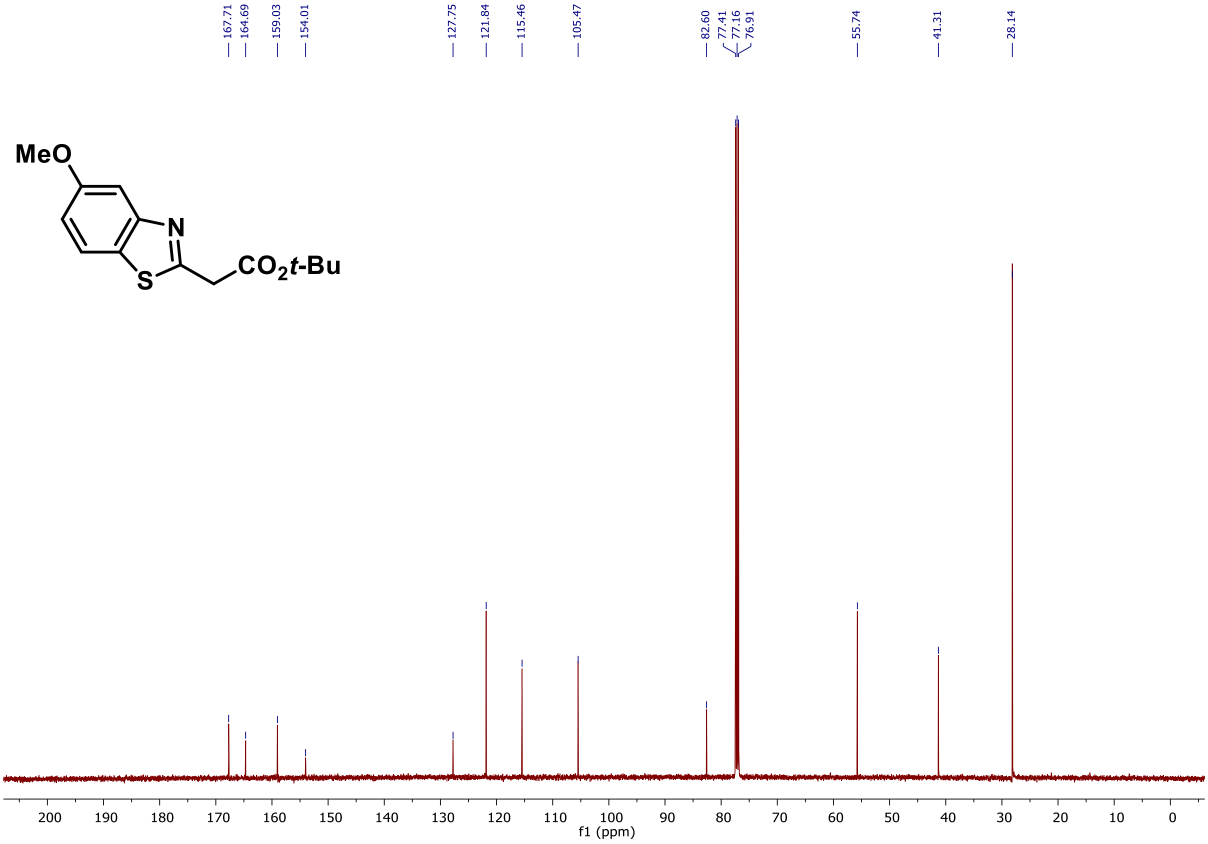

(*S*)-5-Methoxy-2-(2-phenylpropyl)benzo[*d*]thiazole (4qa)

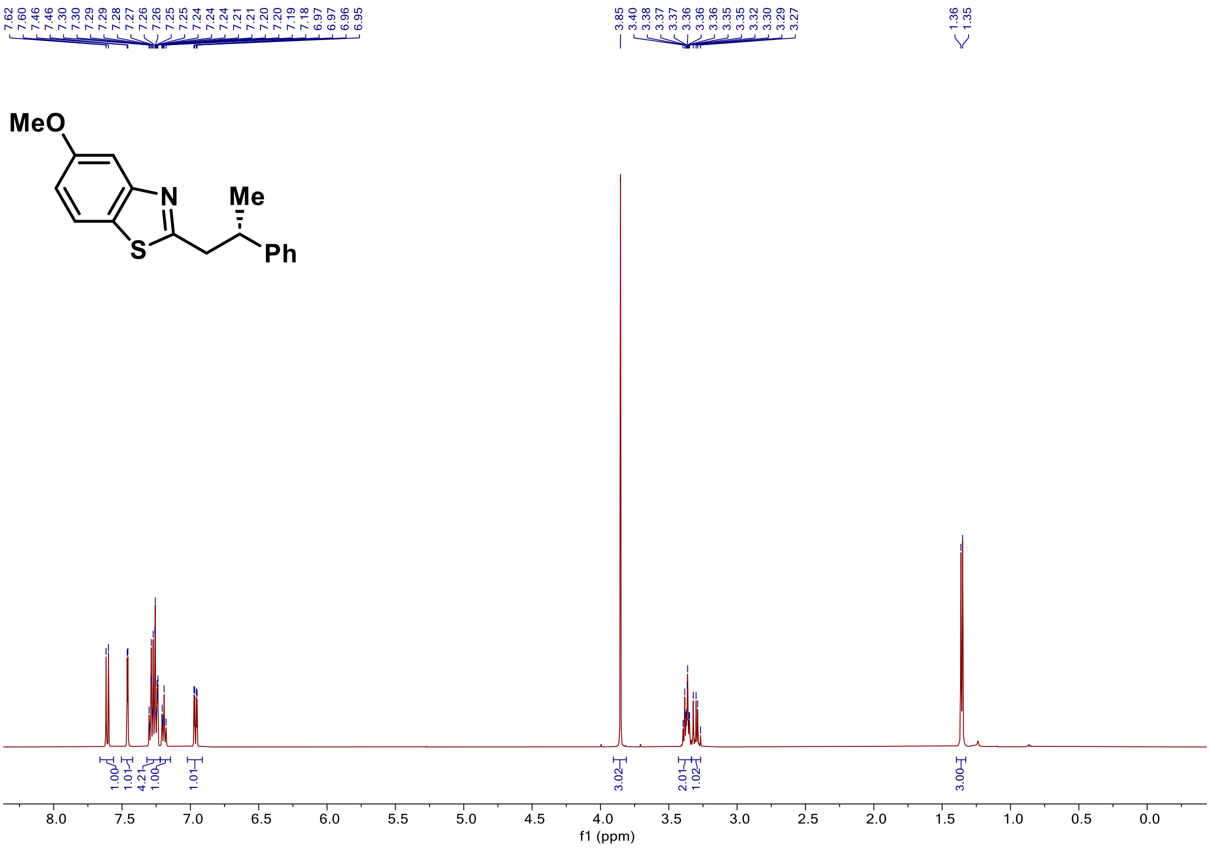

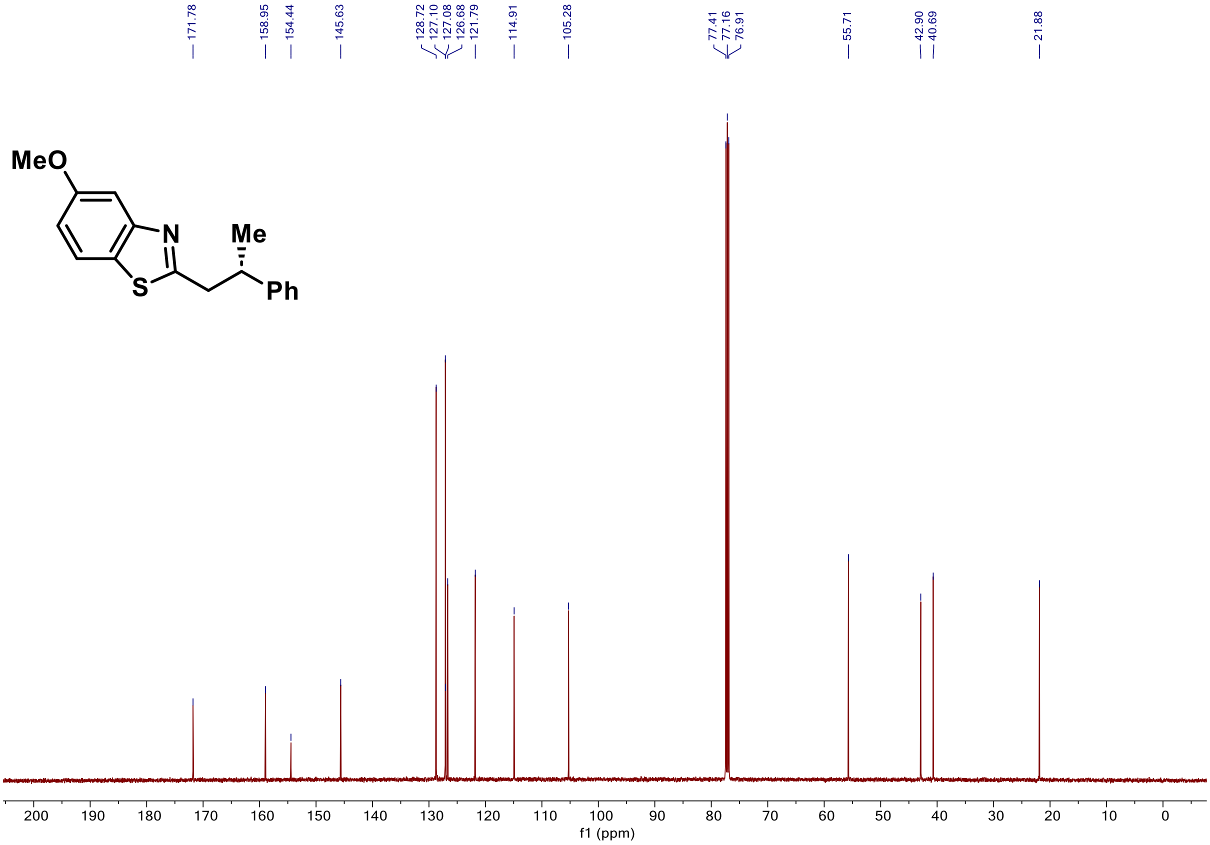

***tert*-Butyl 2-(6-bromobenzo[*d*]thiazol-2-yl)acetate (1r)**

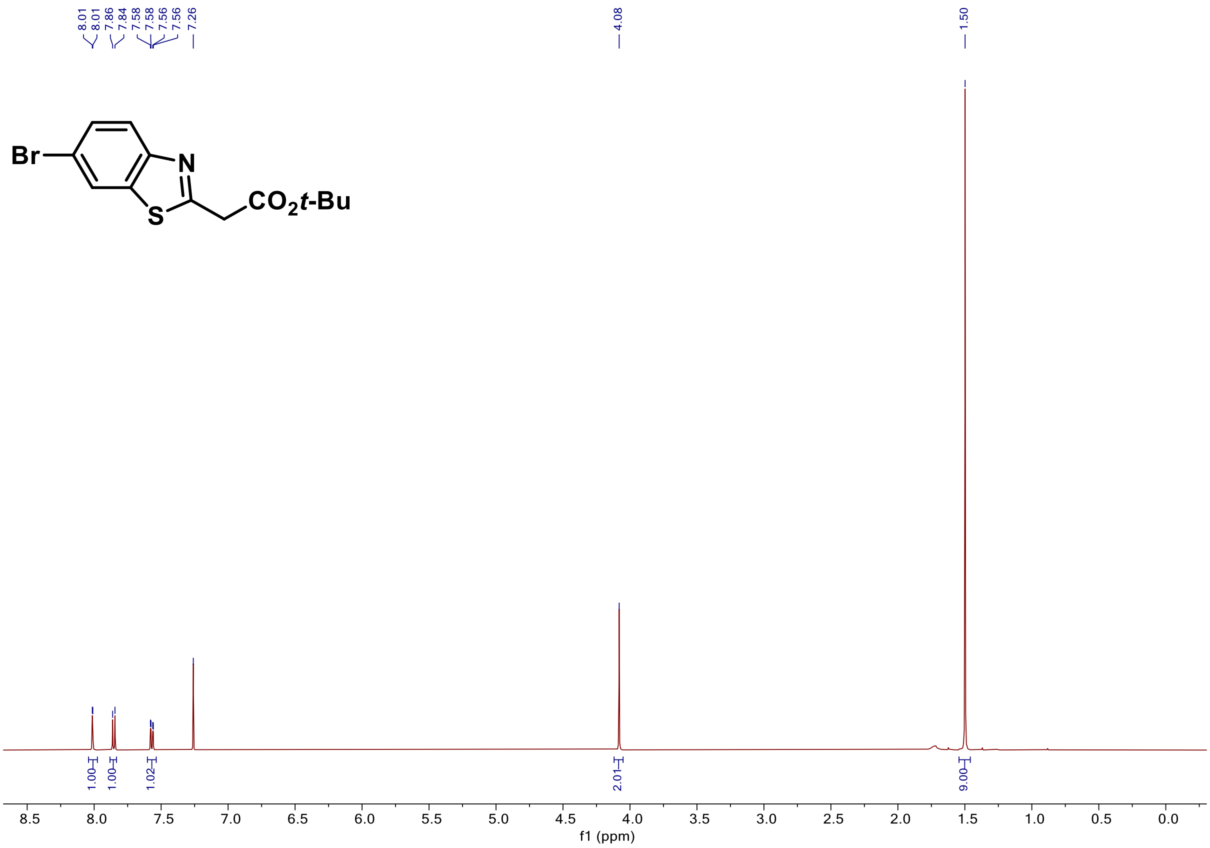

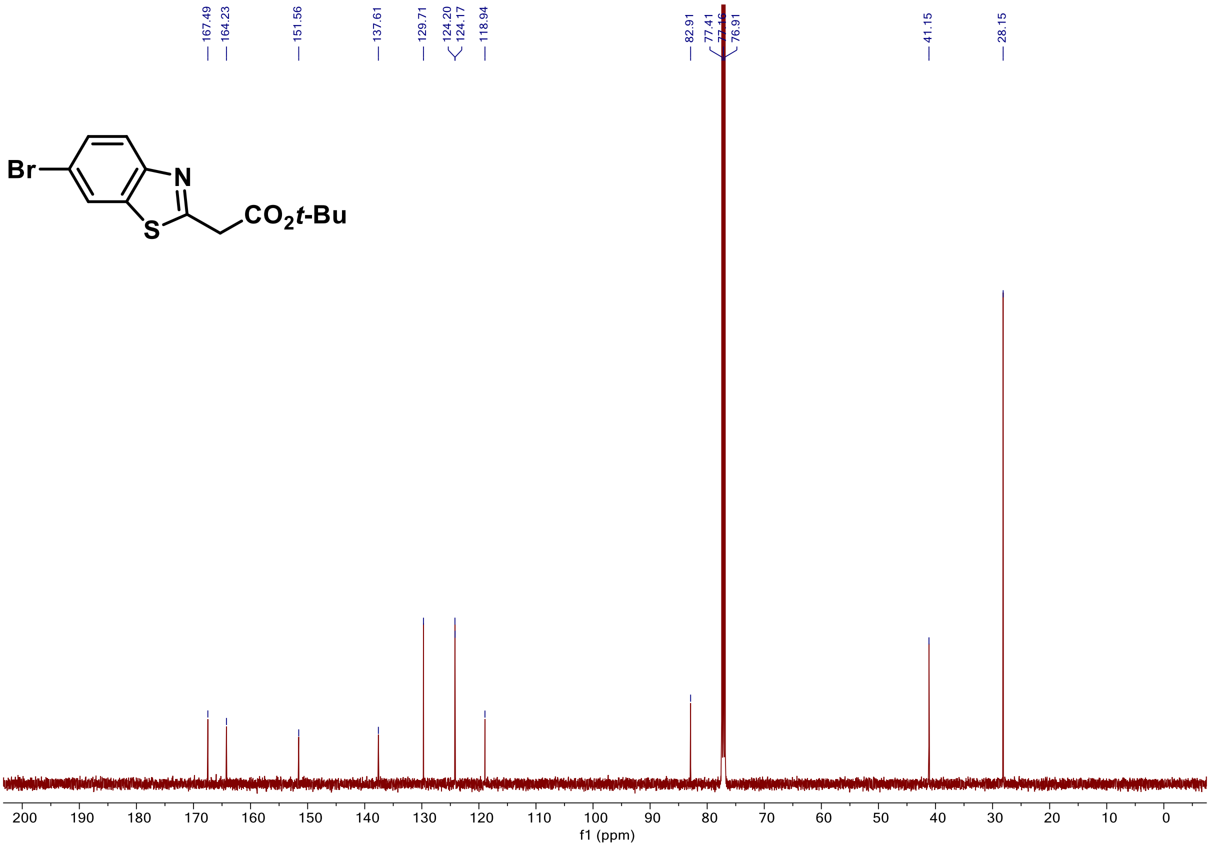

(*S*)-6-Bromo-2-(2-phenylpropyl)benzo[*d*]thiazole (4ra)

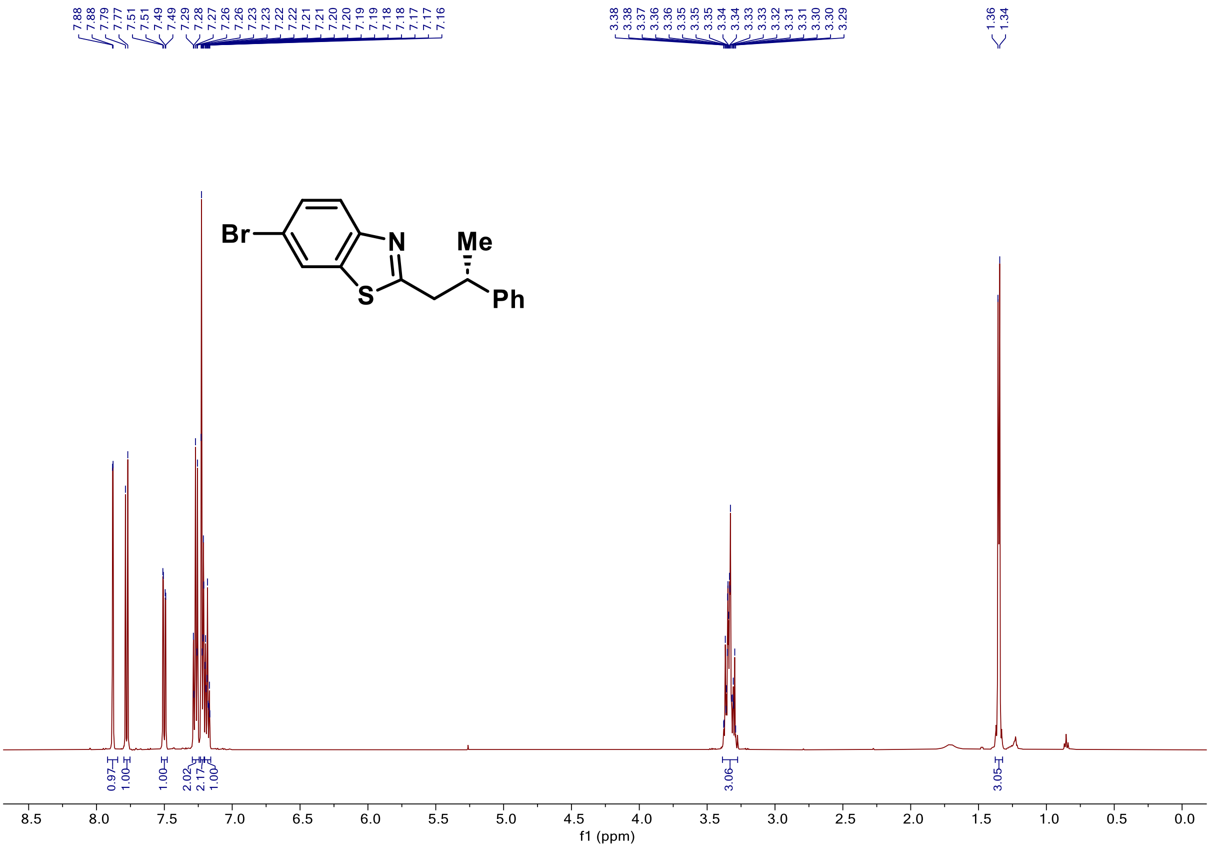

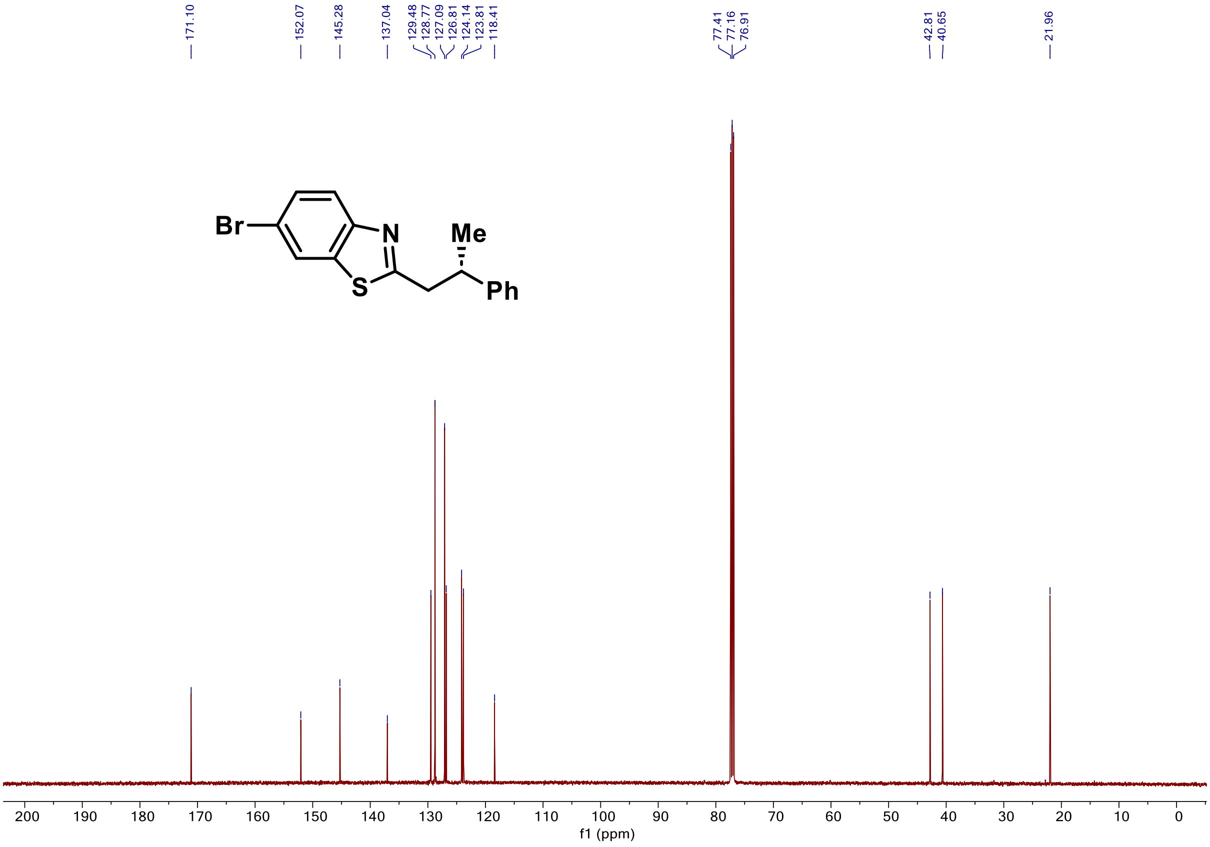

***tert*-Butyl 2-(6-fluorobenzo[*d*]thiazol-2-yl)acetate (1s)**

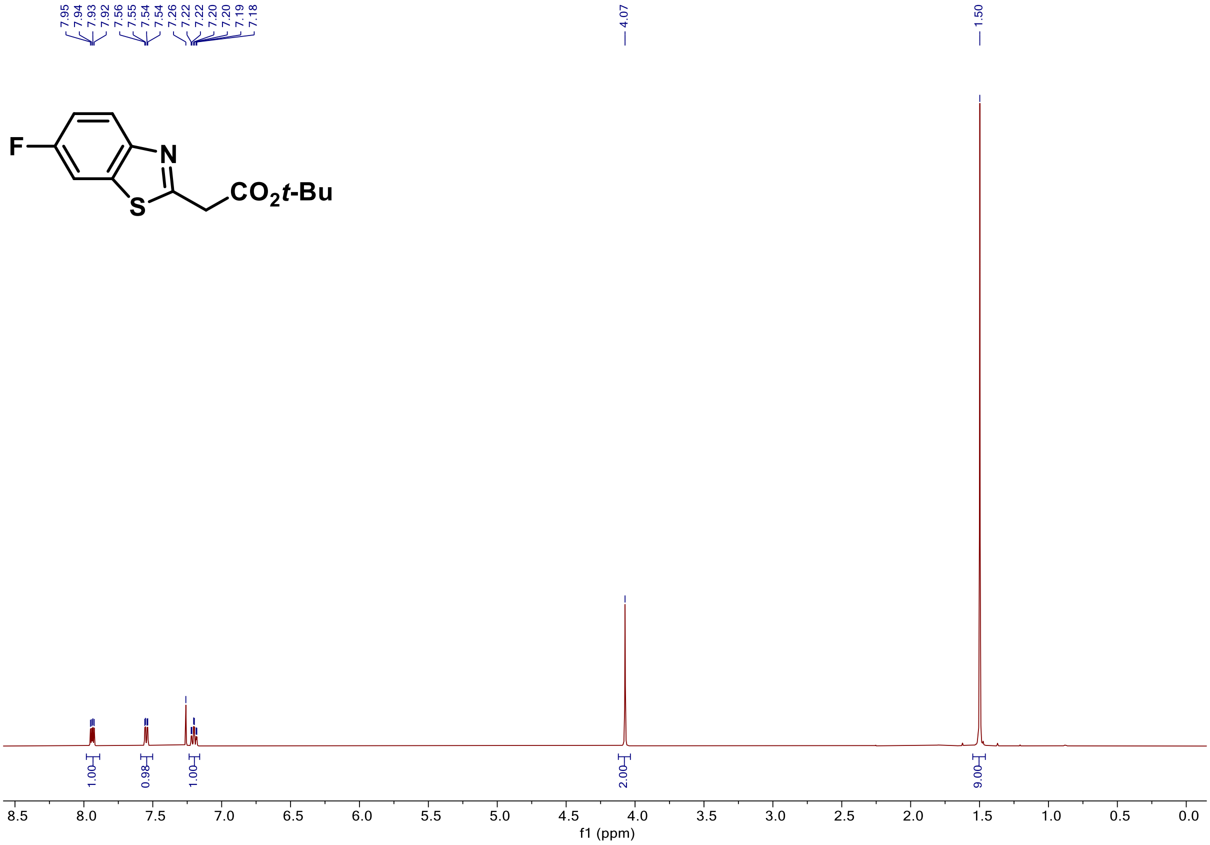

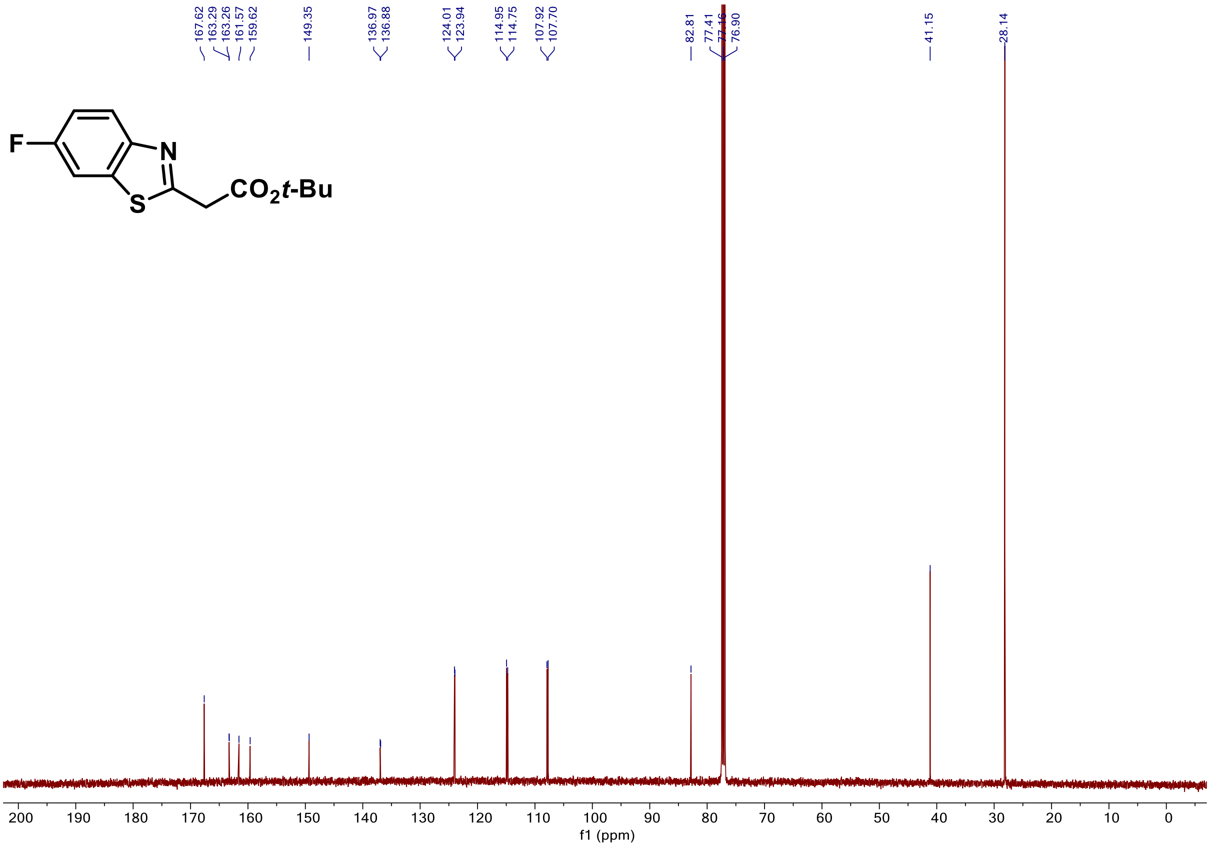

(*S*)-6-Fluoro-2-(2-phenylpropyl)benzo[*d*]thiazole (4sa)

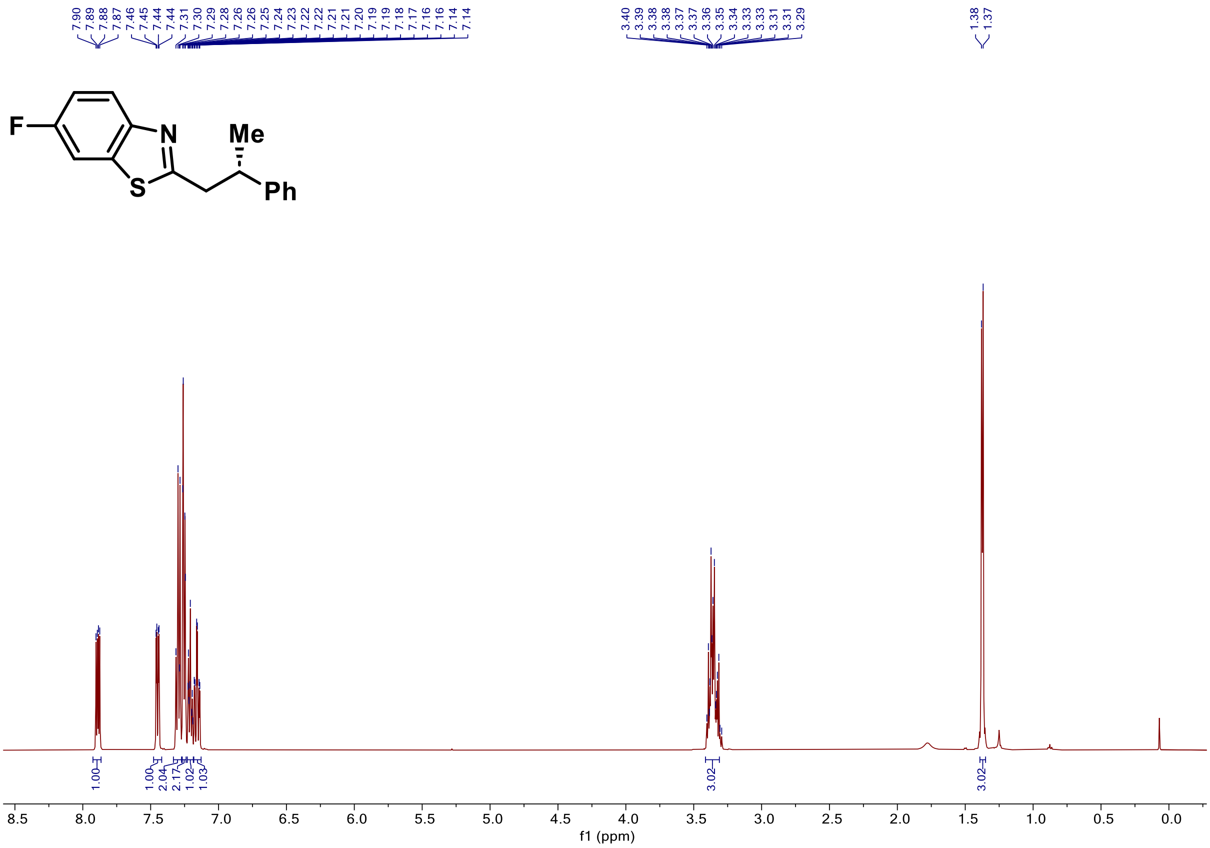

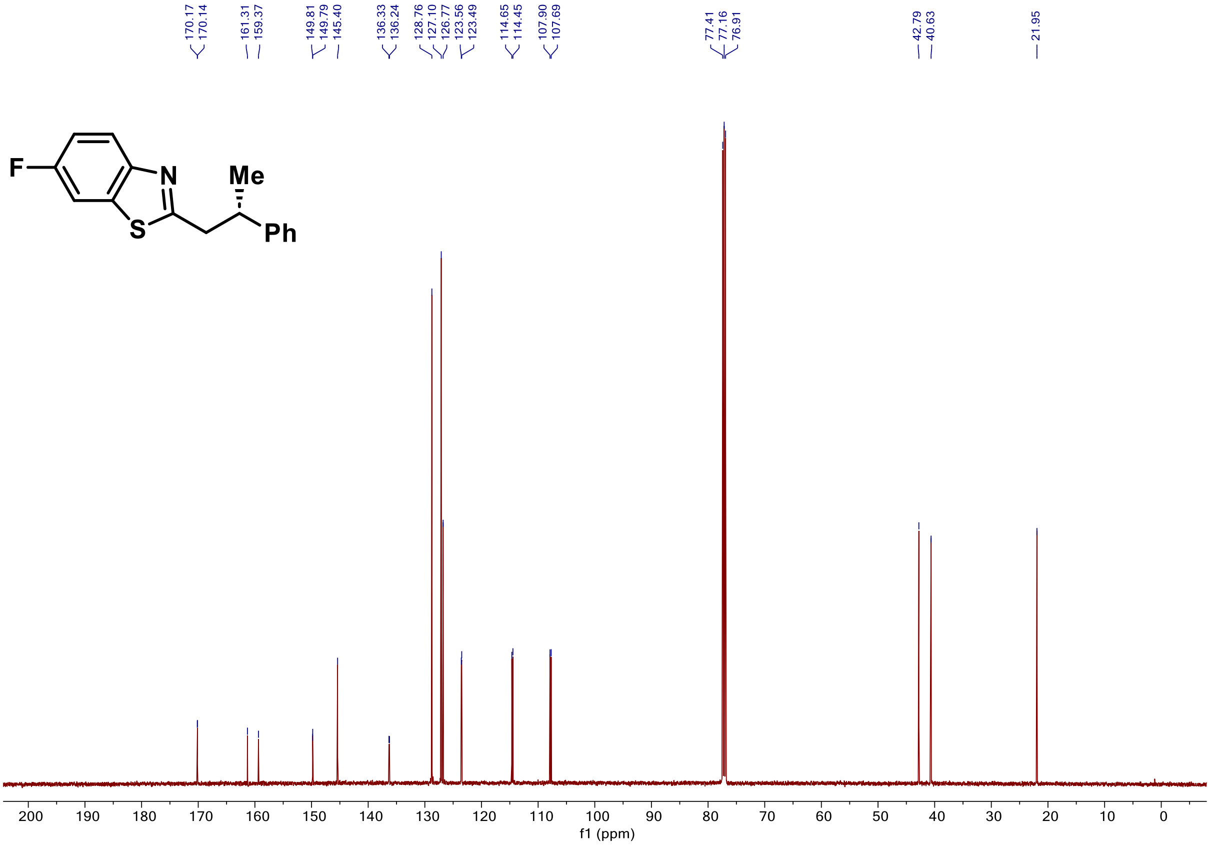

(*S*)-2-(2-(*p*-Tolyl)propyl)benzo[*d*]thiazole (4gb)

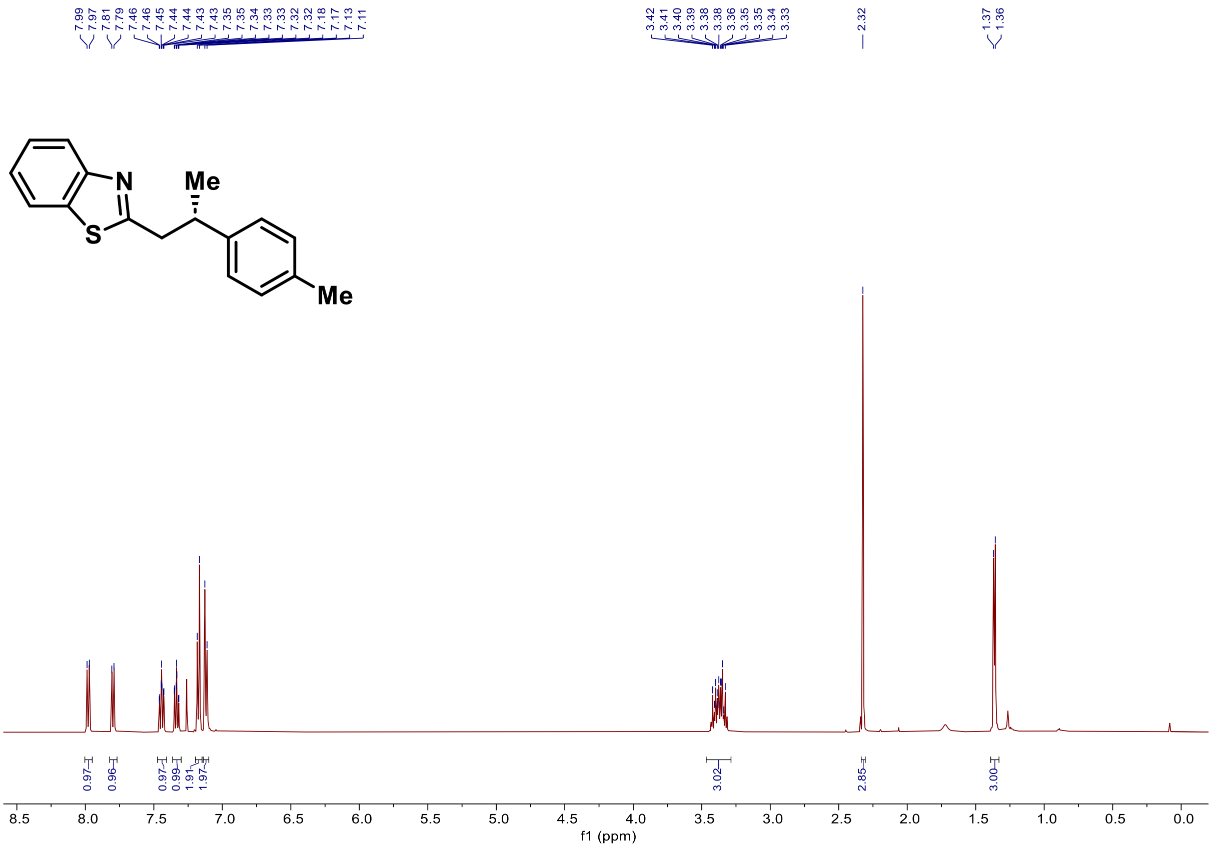

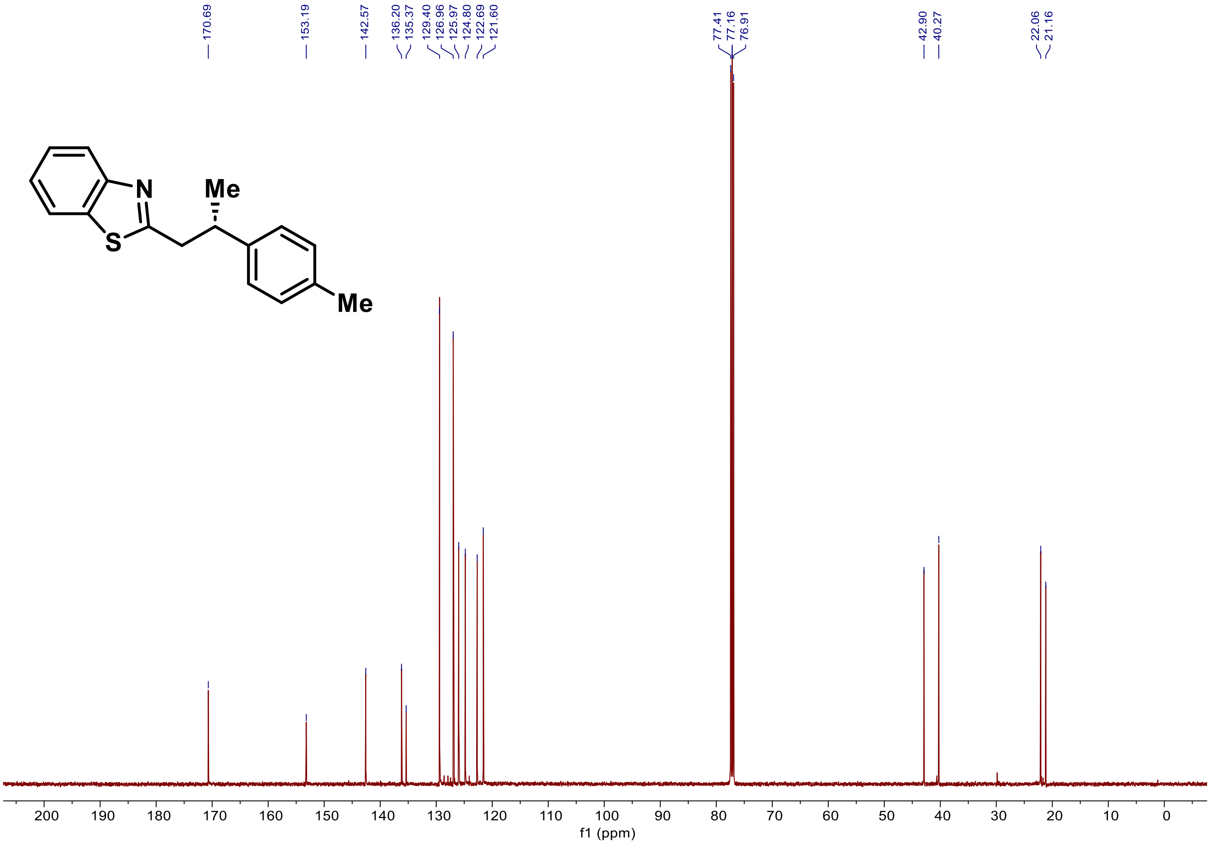

(*S*)-2-(2-([1,1'-Biphenyl]-4-yl)propyl)benzo[*d*]thiazole (4gc)

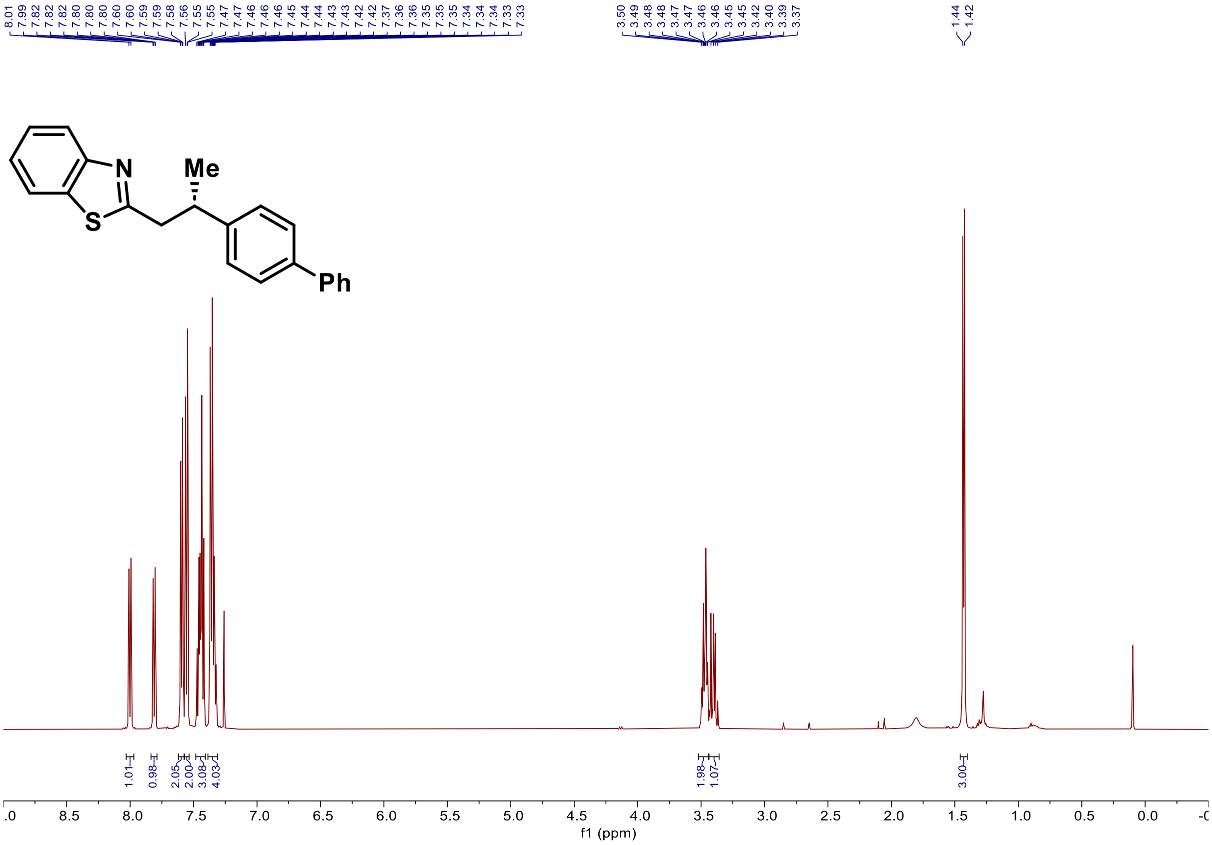

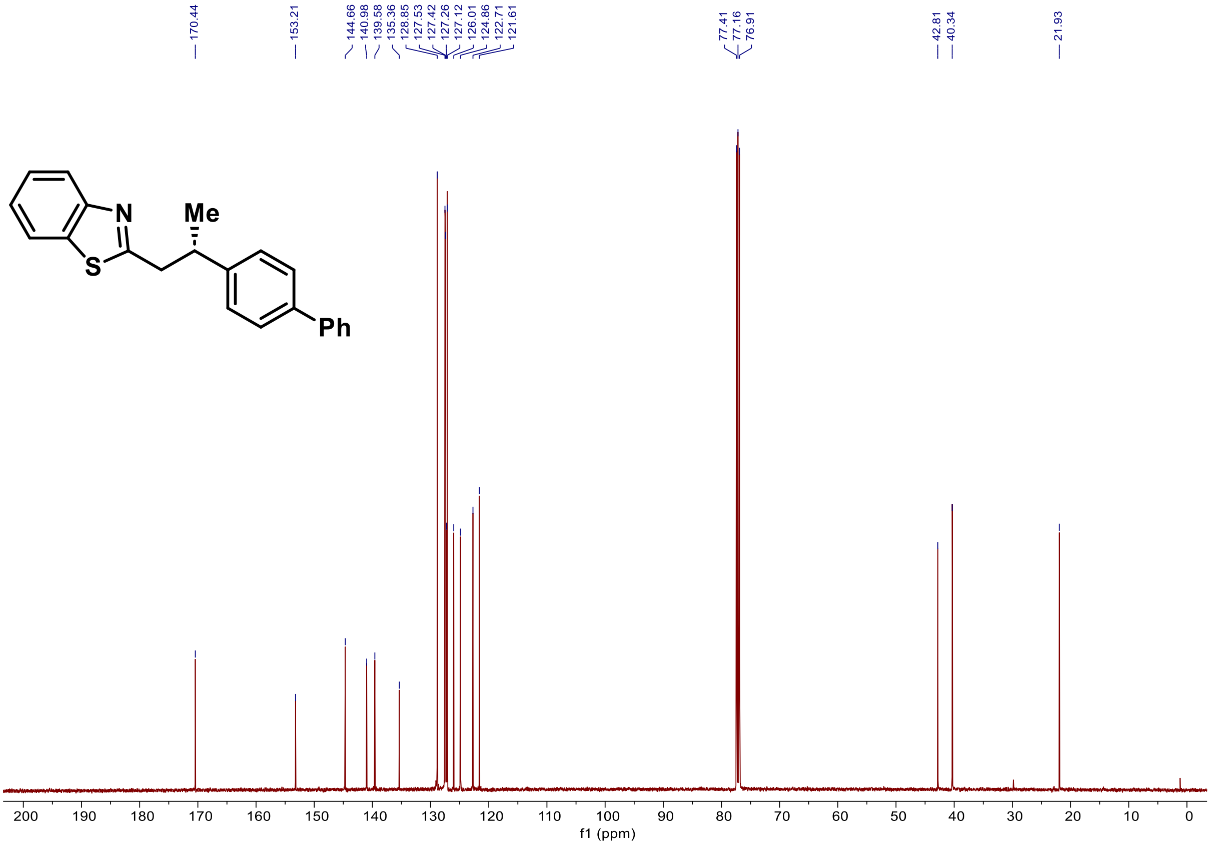

(*S*)-2-(2-(4-Methoxyphenyl)propyl)benzo[*d*]thiazole (4gd)

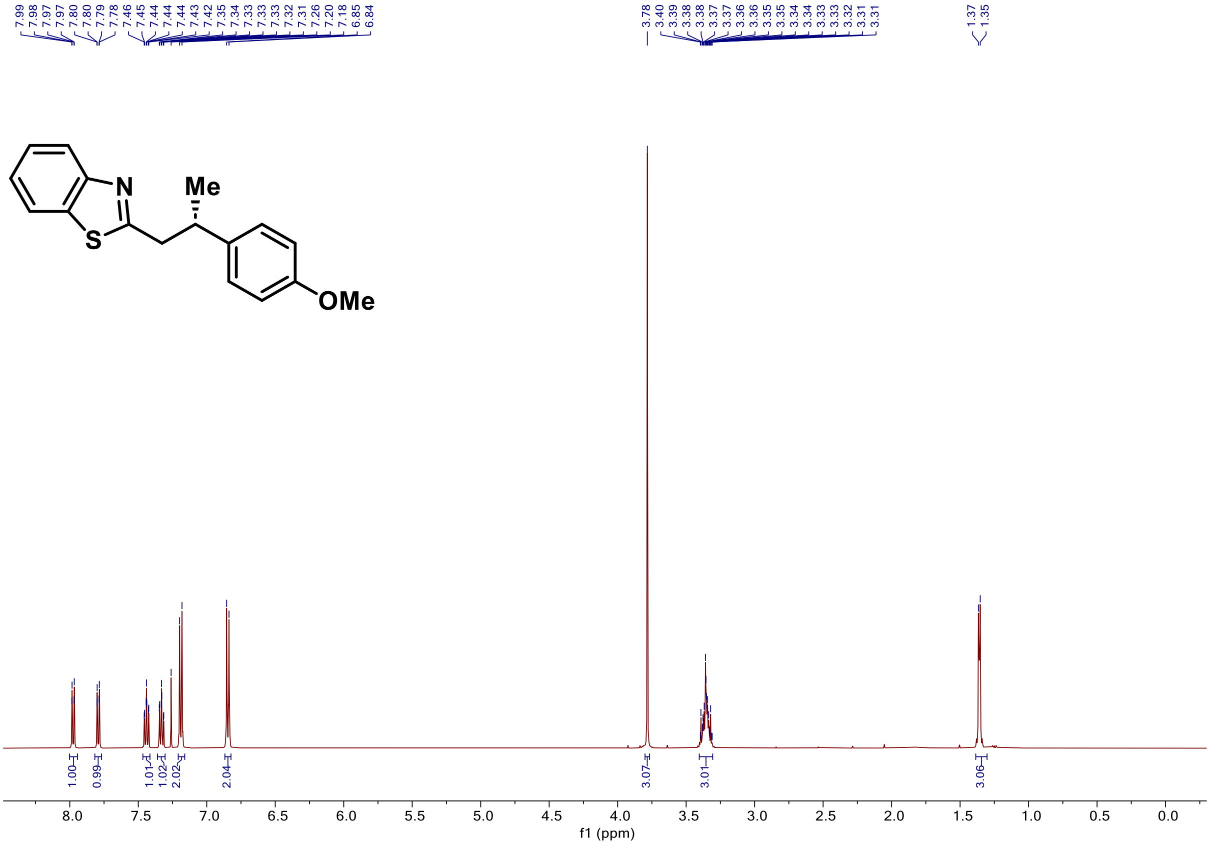

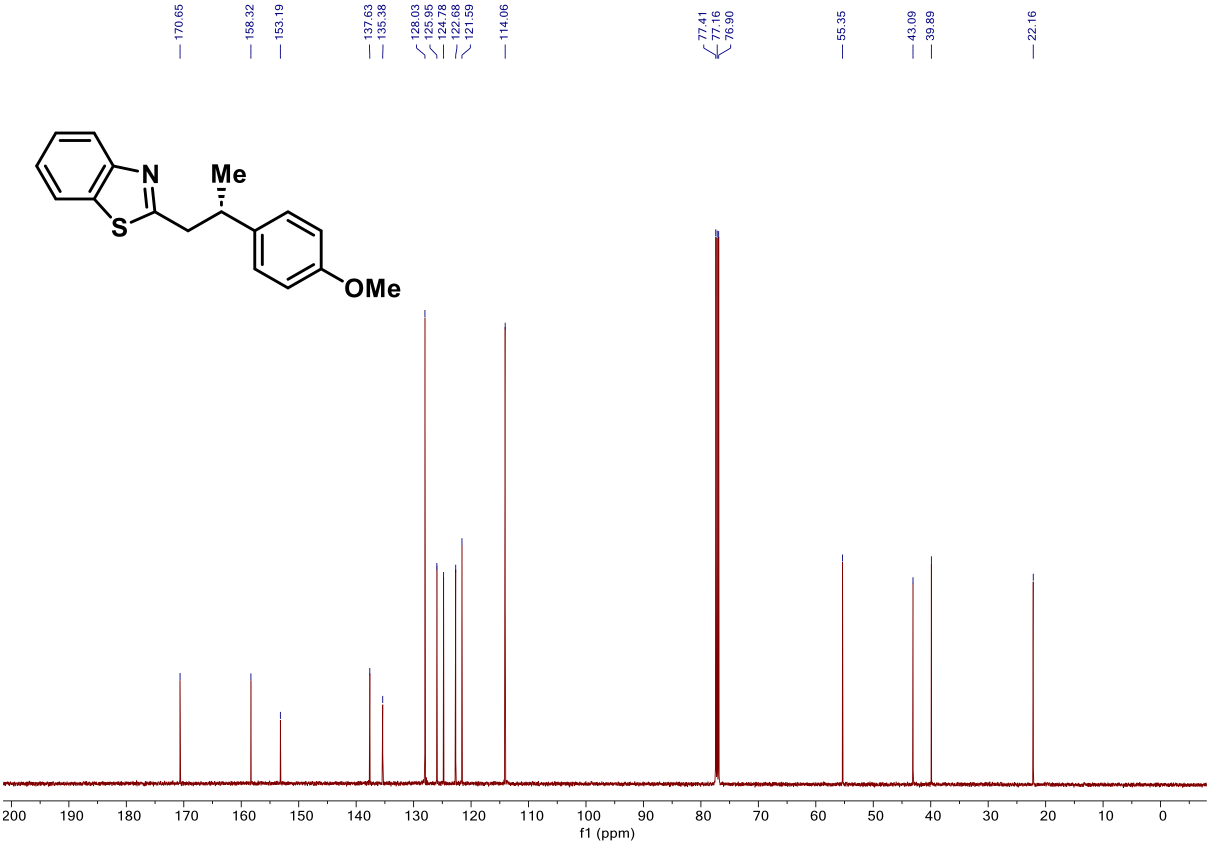

(*S*)-2-(2-(4-Bromophenyl)propyl)benzo[*d*]thiazole (4ge)

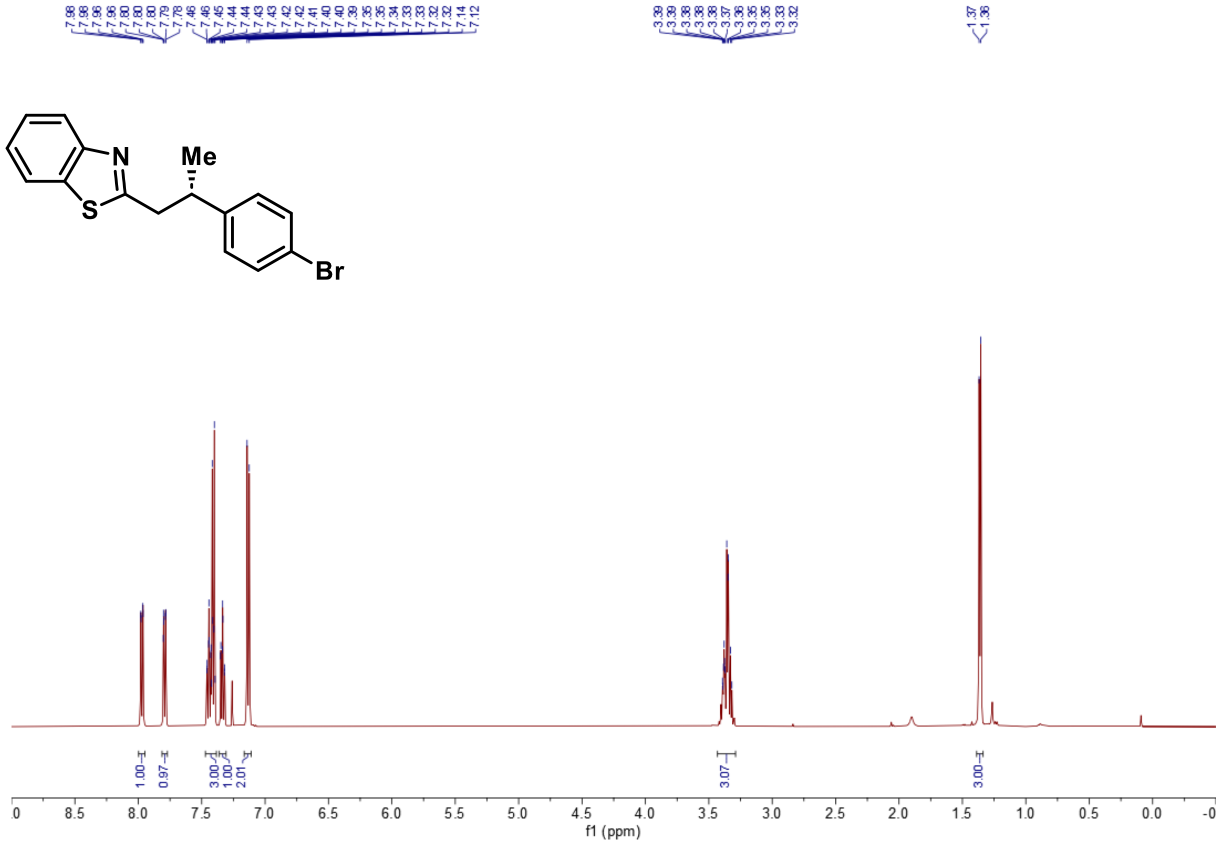

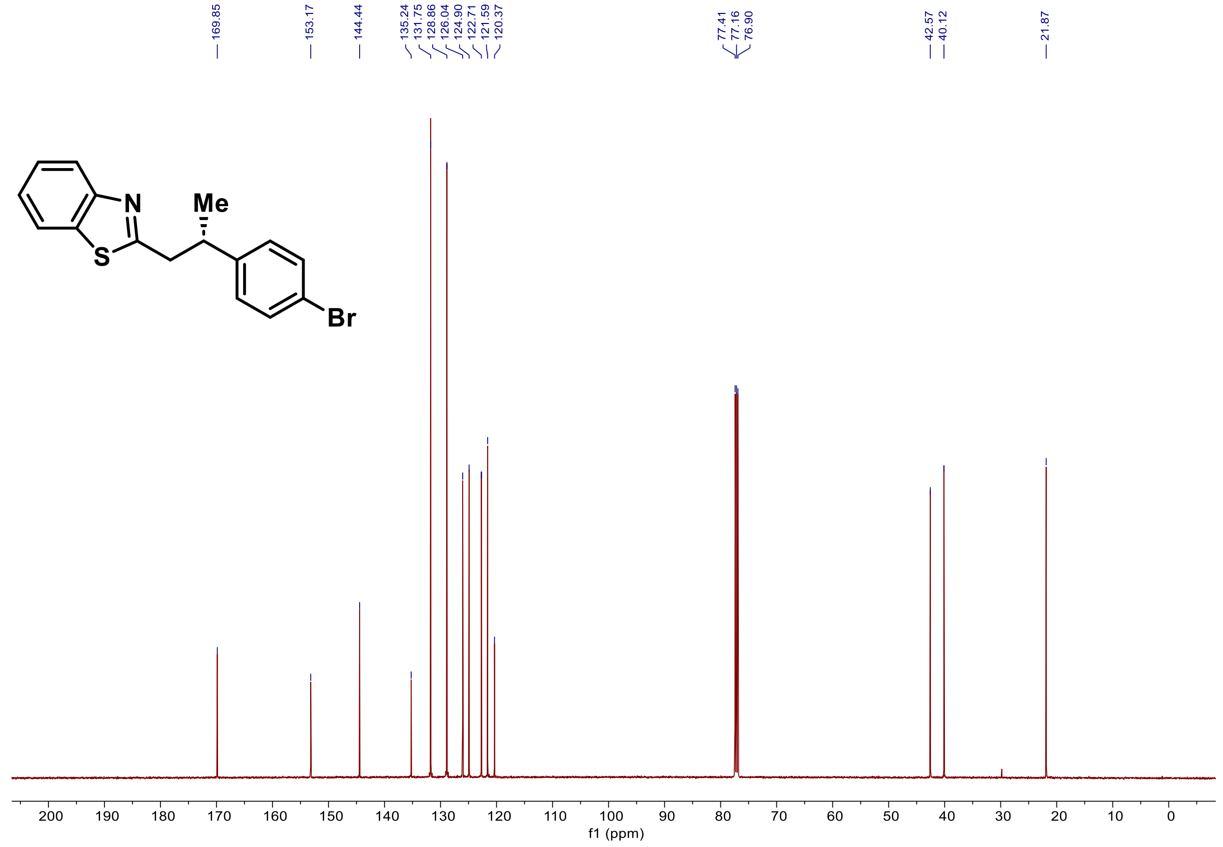

(*S*)-2-(2-(4-Fluorophenyl)propyl)benzo[*d*]thiazole (4gf)

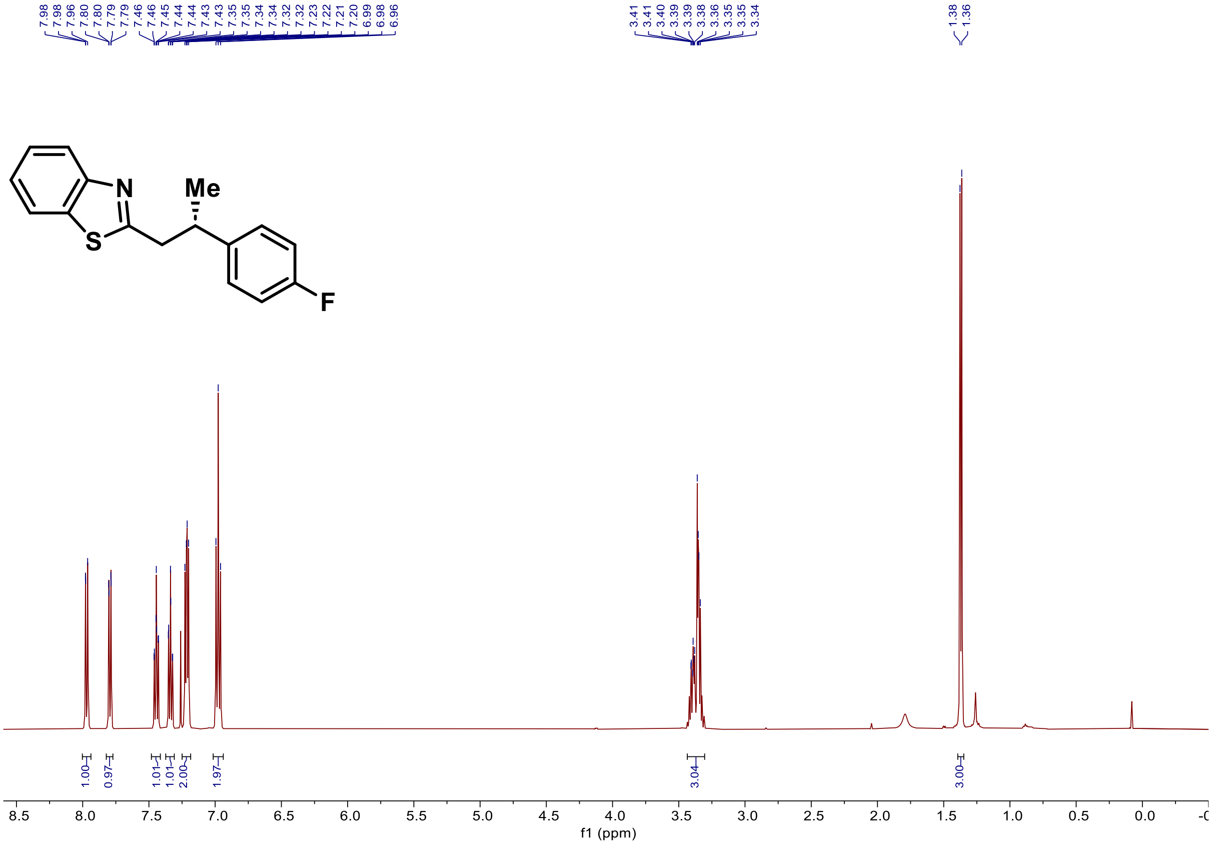

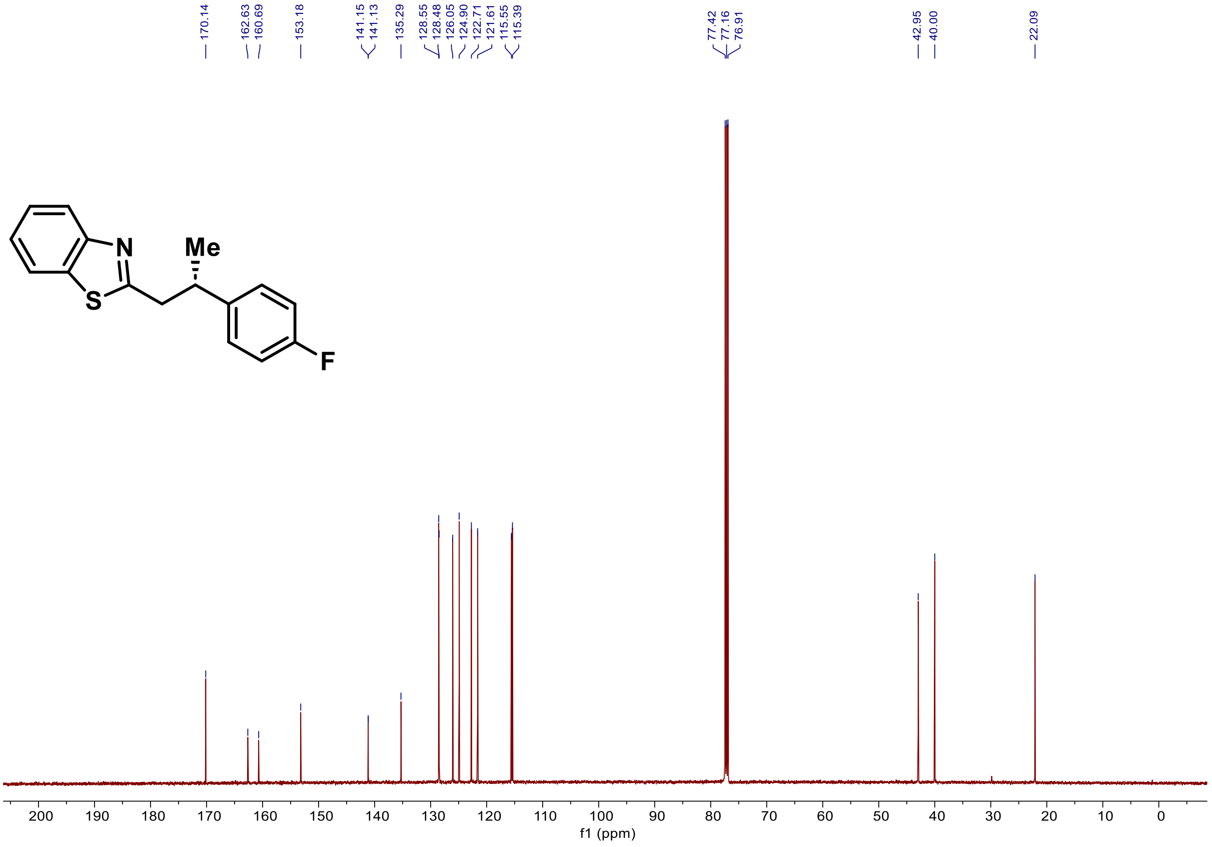

(*S*)-2-(2-(2-Fluorophenyl)propyl)benzo[*d*]thiazole (4gg)

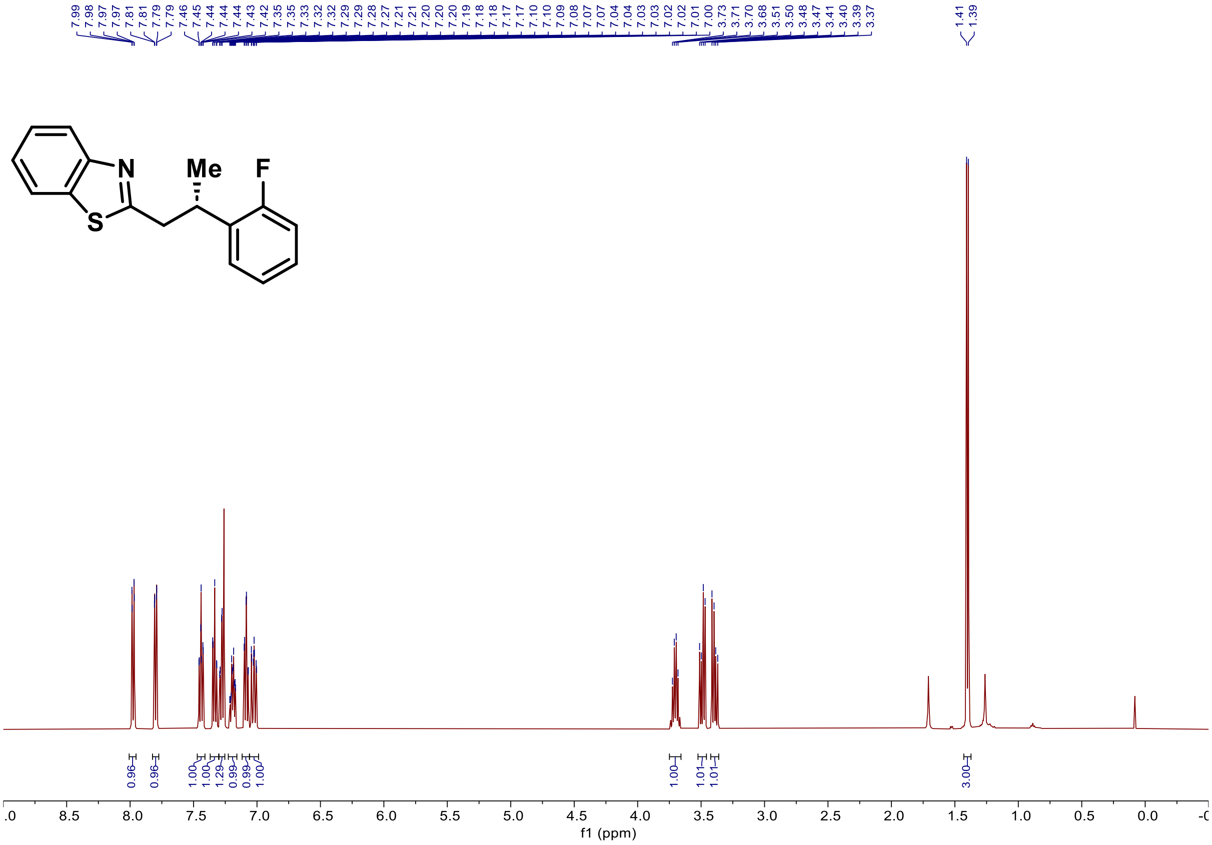

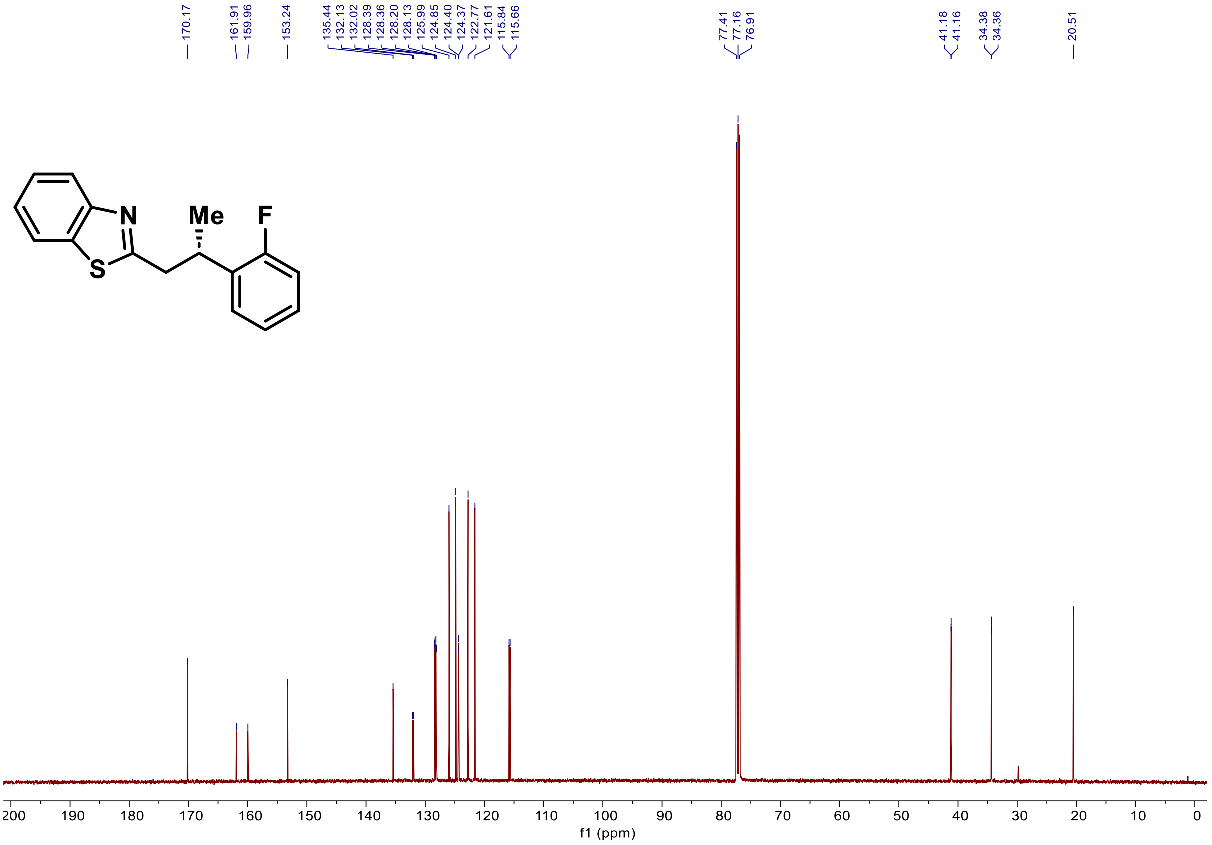

(*S*)-2-(2-(Naphthalen-2-yl)propyl)benzo[*d*]thiazole (4gh)

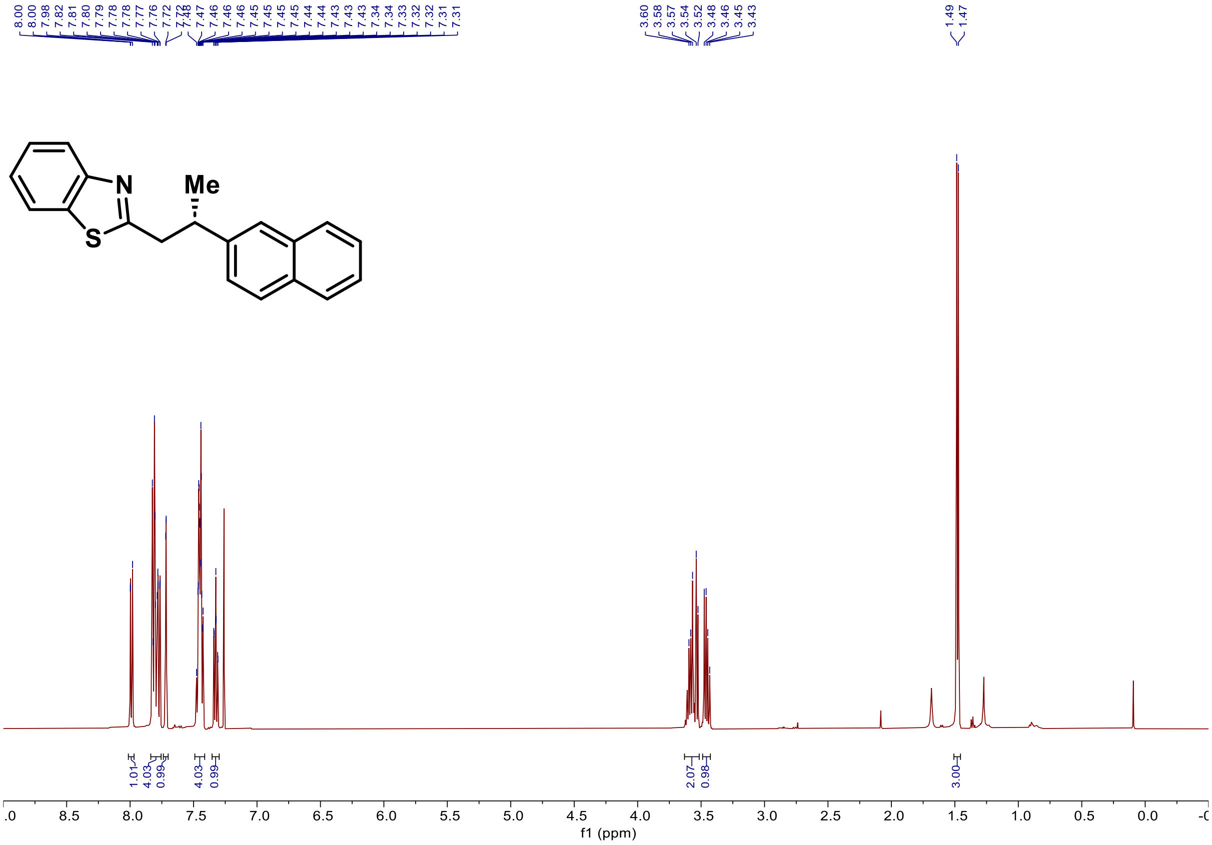

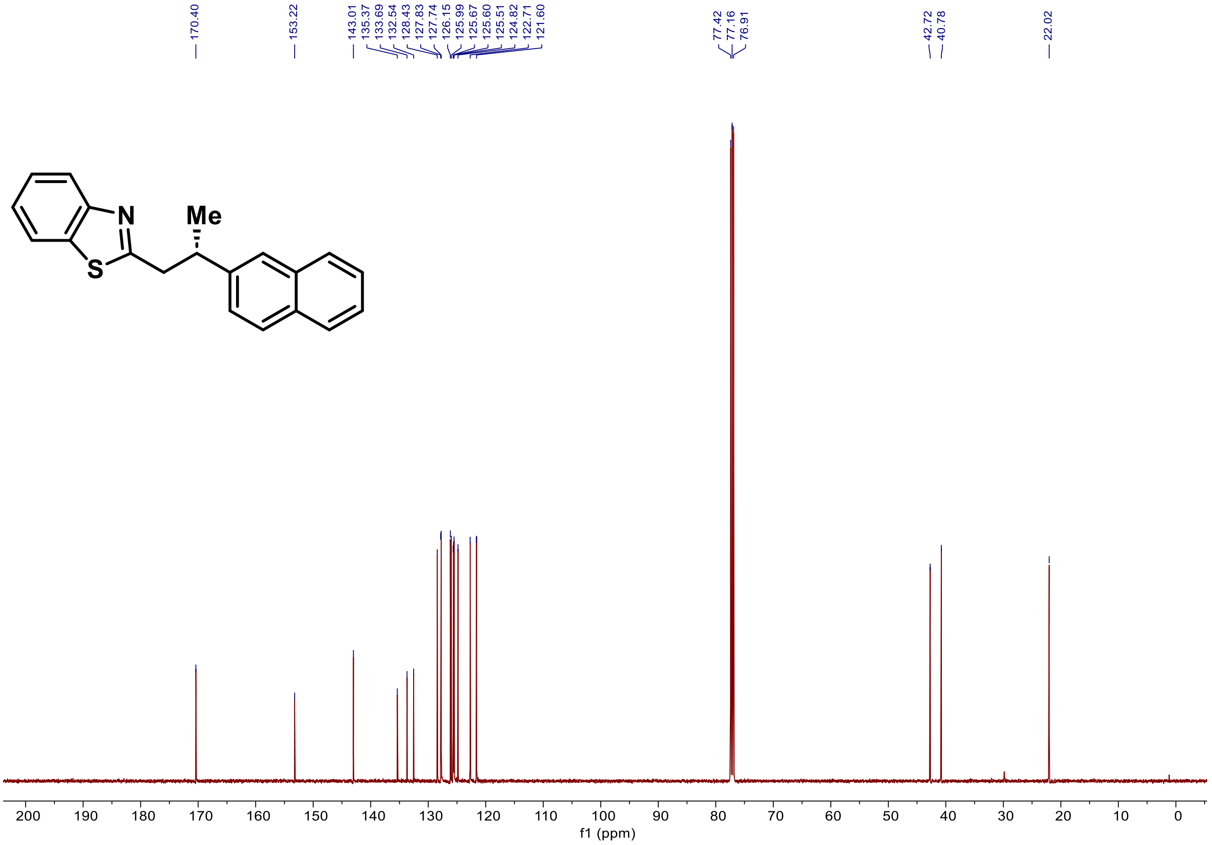

(*S*)-2-(2-(Ferrocenyl)propyl)benzo[*d*]thiazole (4gi)

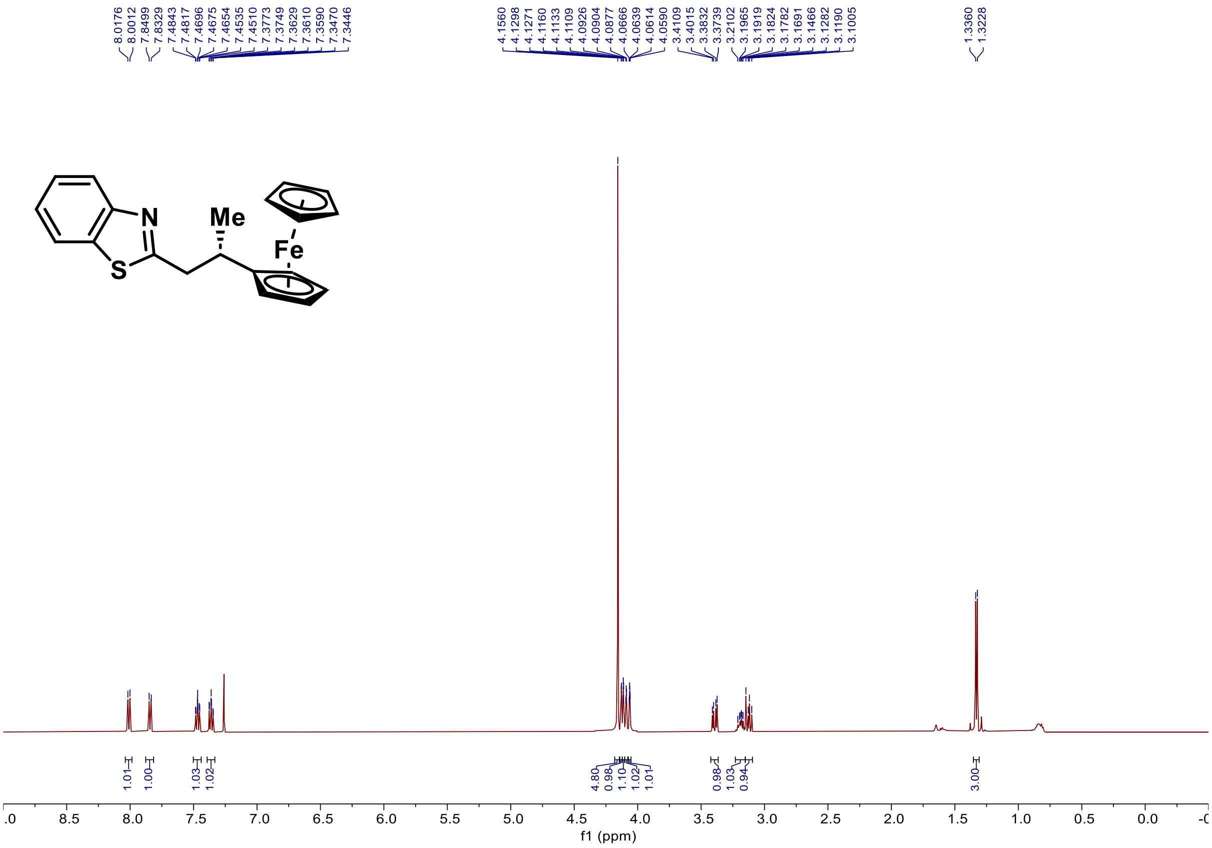

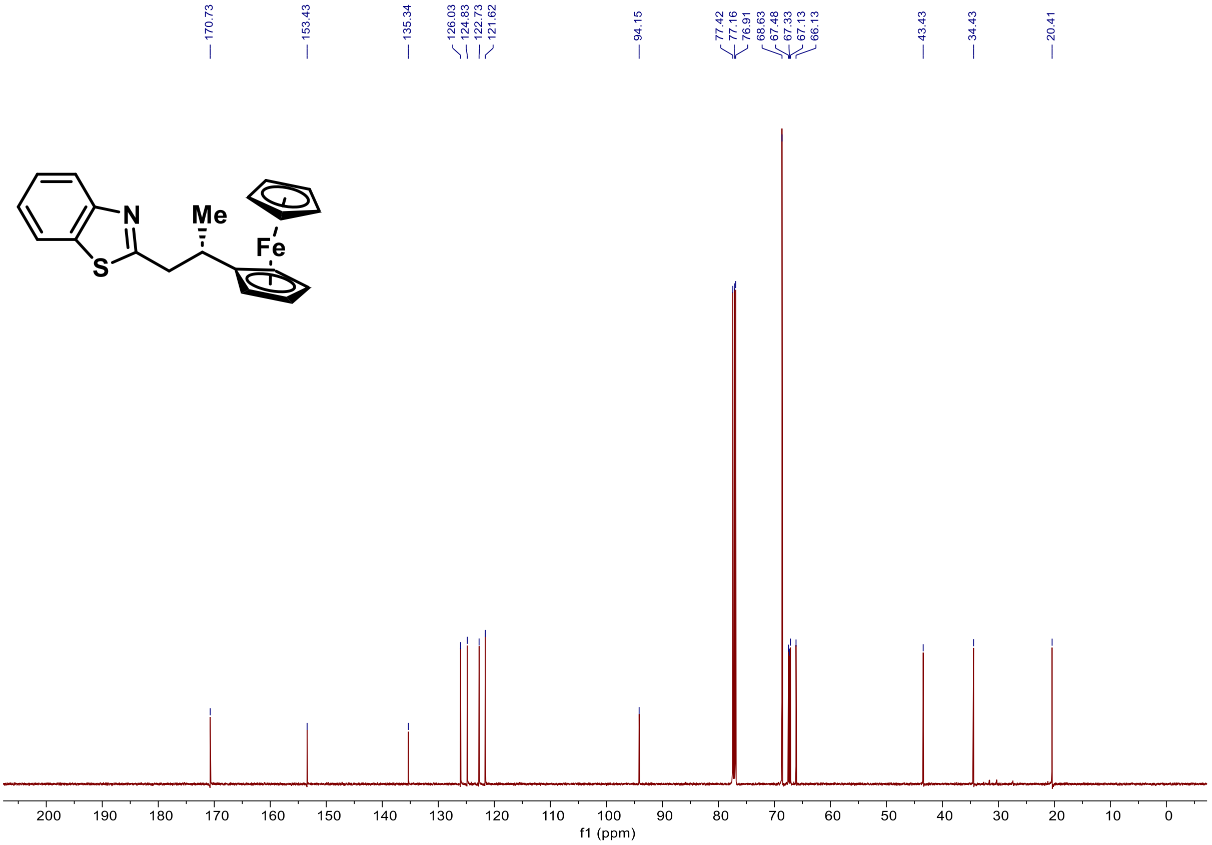

(*R*)-2-(2-Methylhexyl)benzo[*d*]thiazole (4gj)

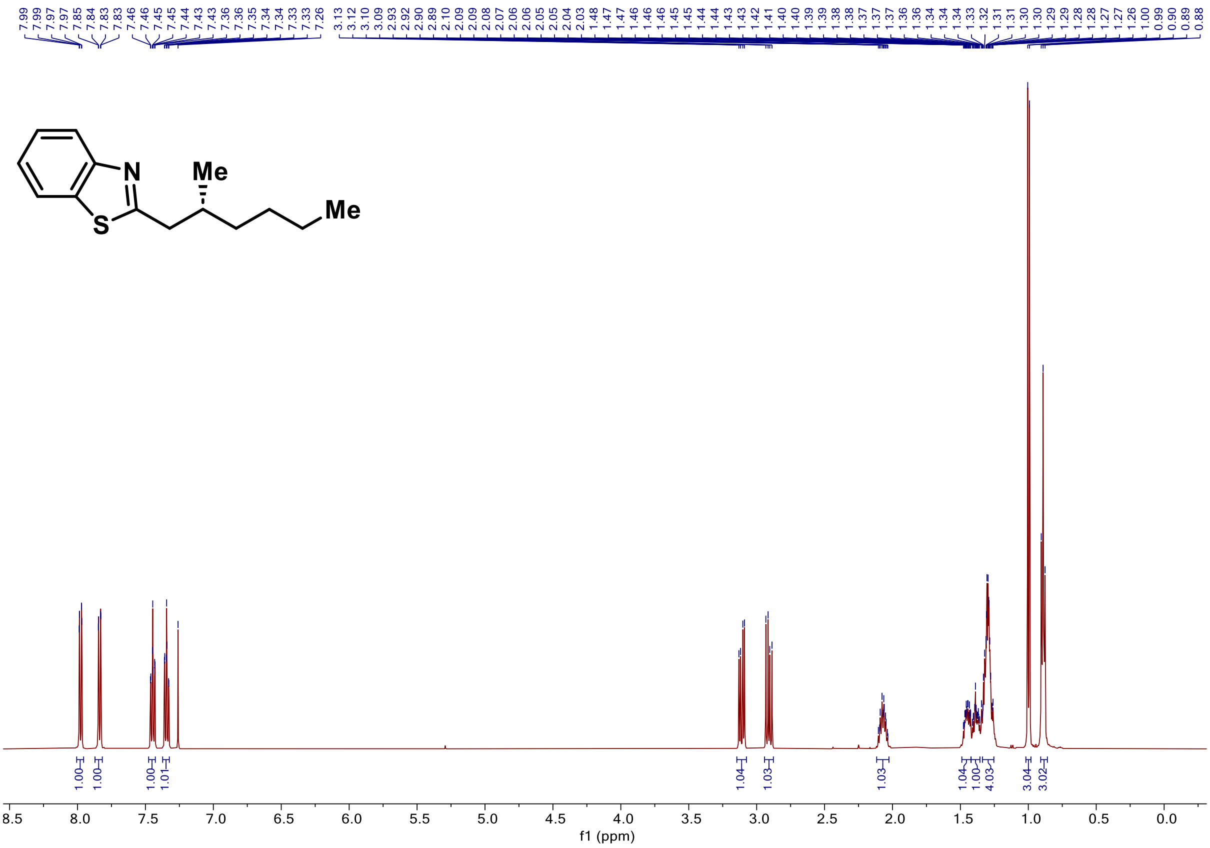

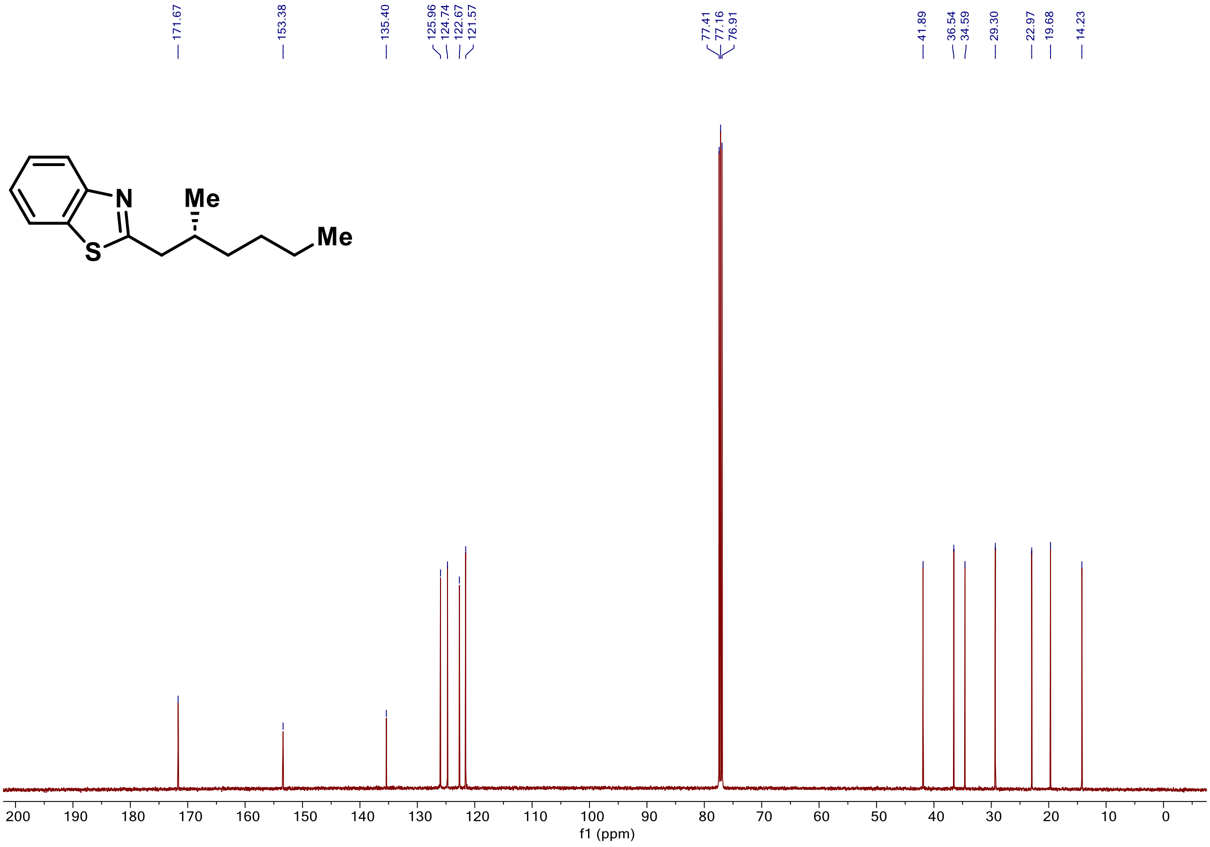

(*R*)-2-(2,4-Dimethylpentyl)benzo[*d*]thiazole (4gk)

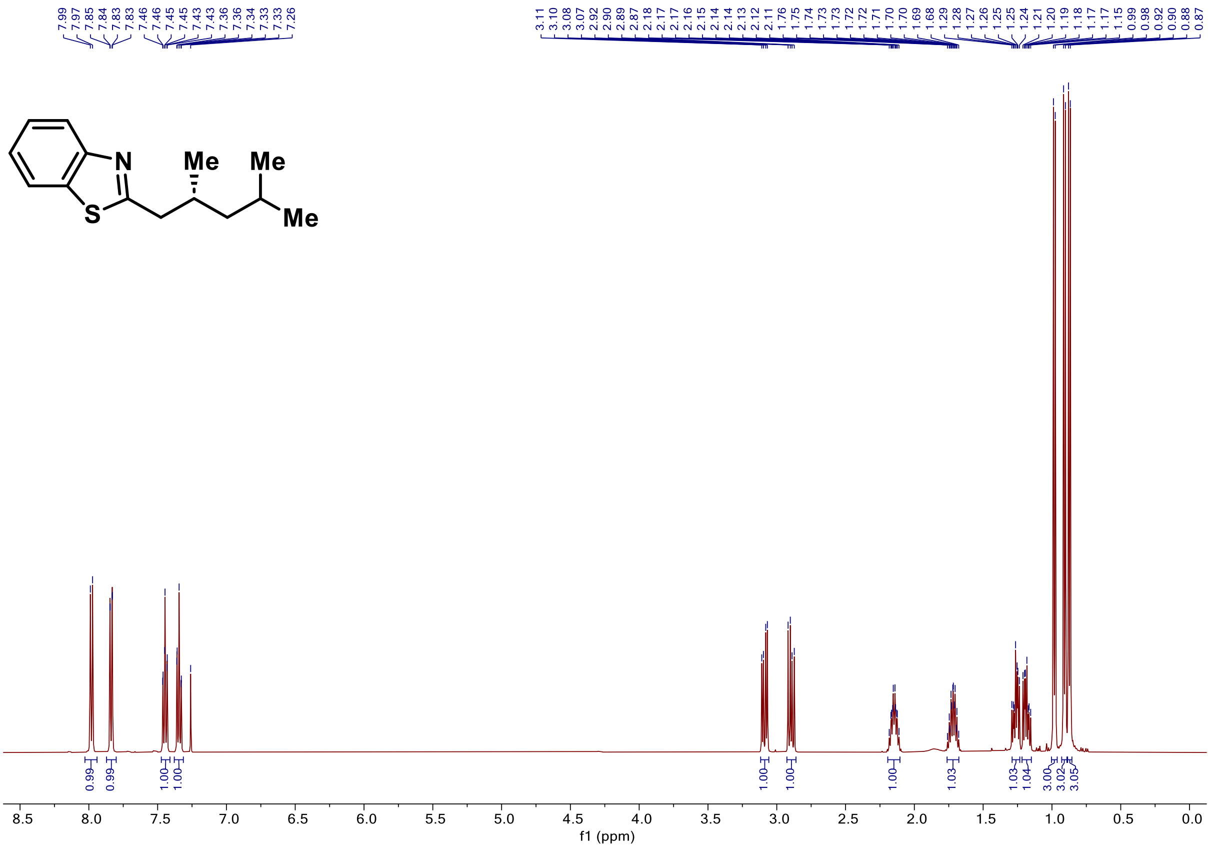

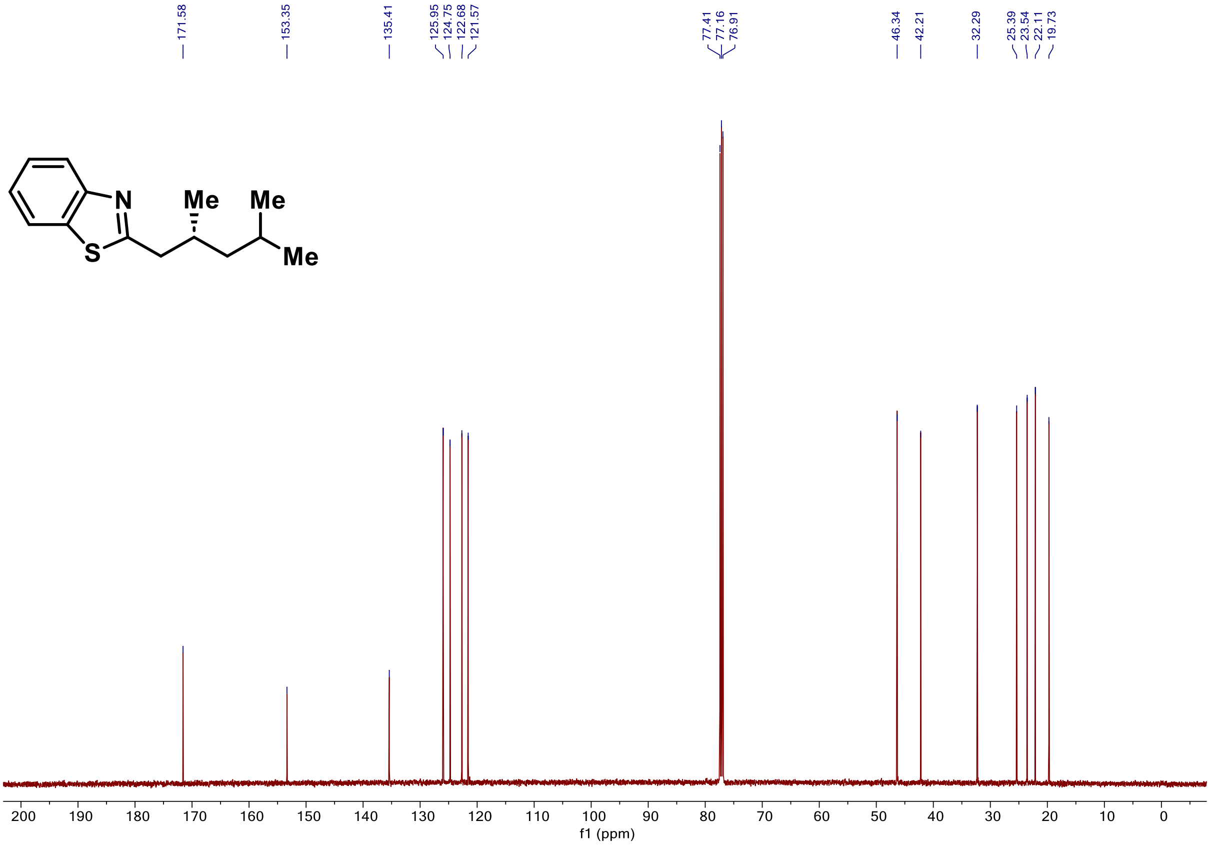

(*S*)-2-(2,3-Dimethylbutyl)benzo[*d*]thiazole (4gl)

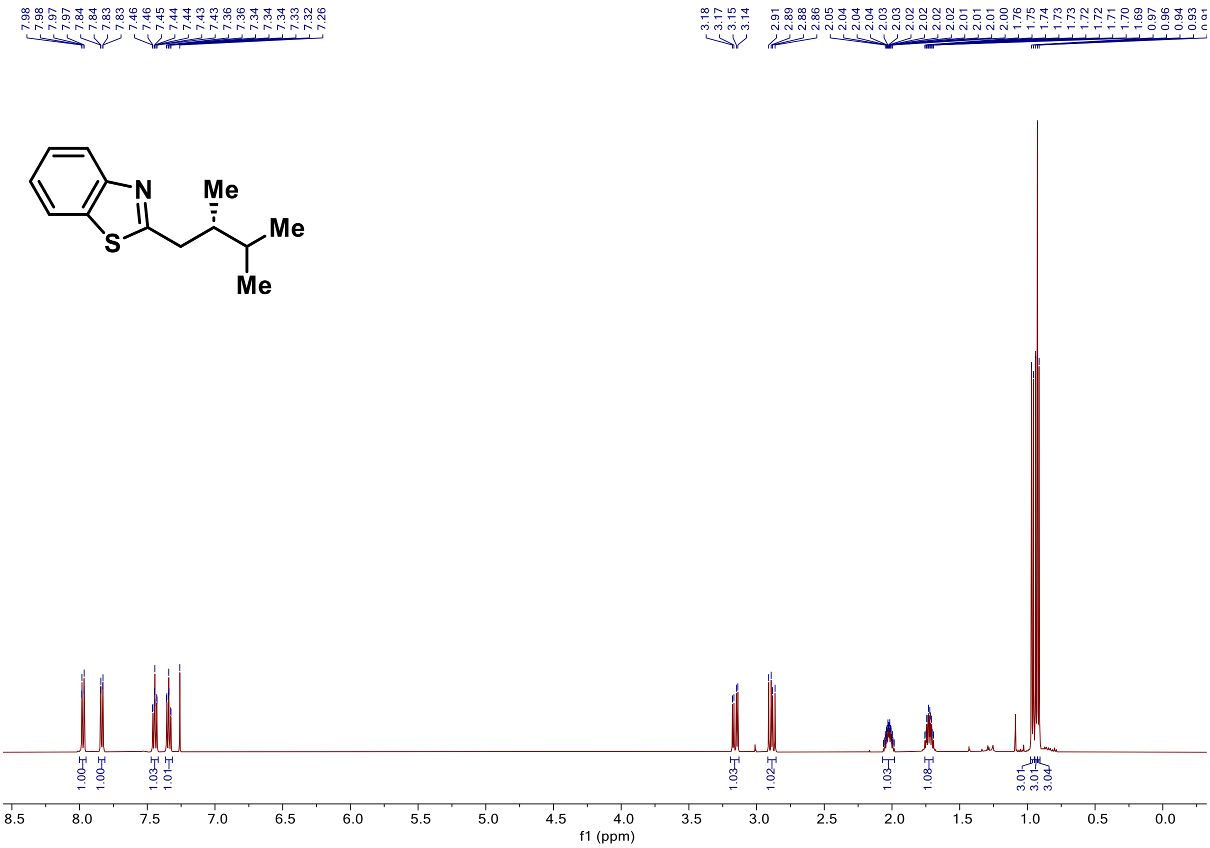

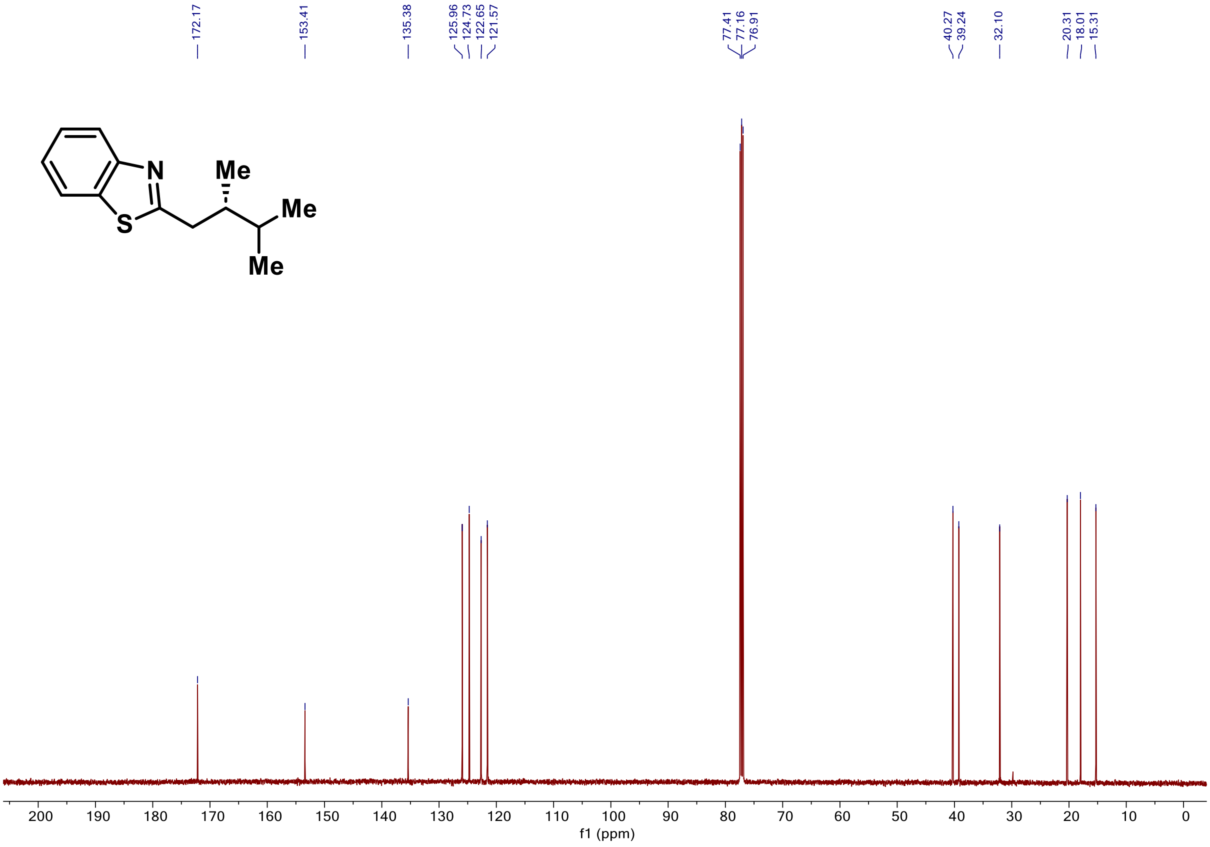

(*S*)-2-(2,3,3-Trimethylbutyl)benzo[*d*]thiazole (4gm)

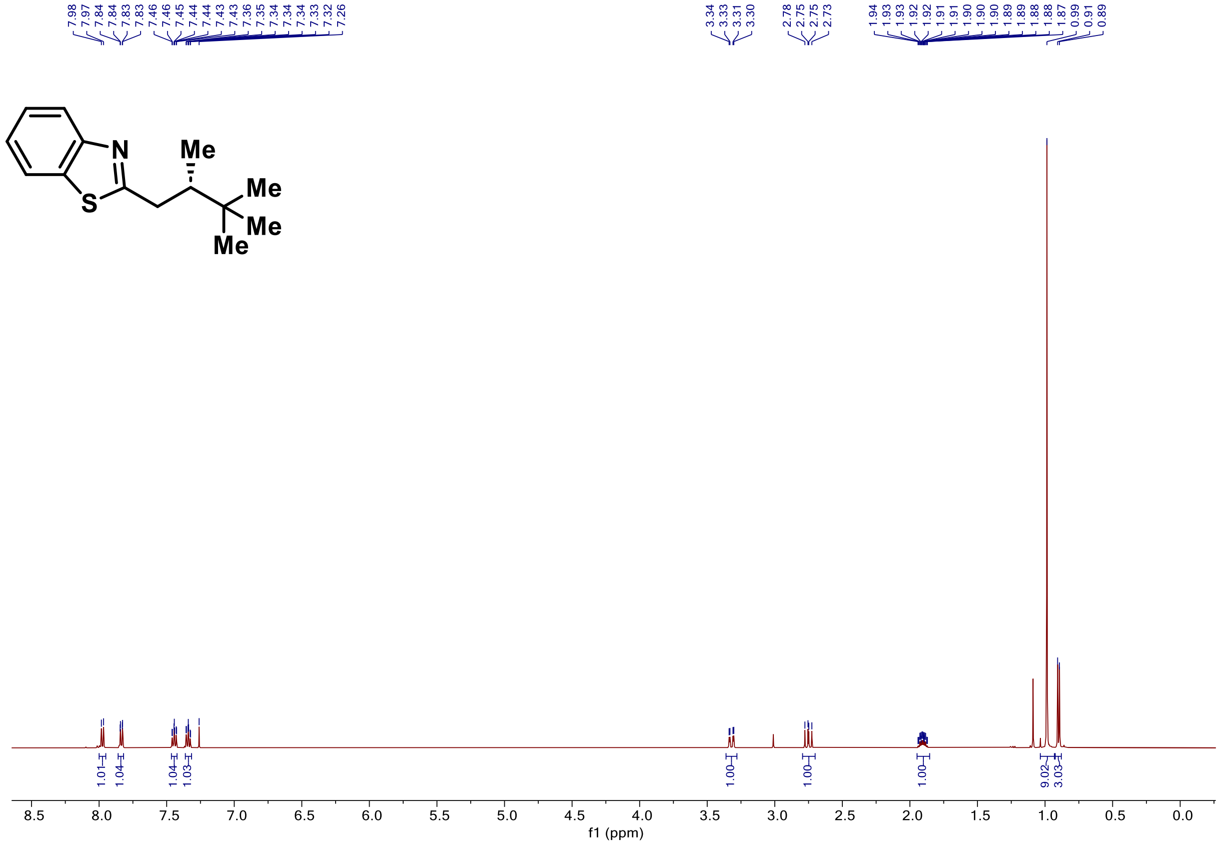

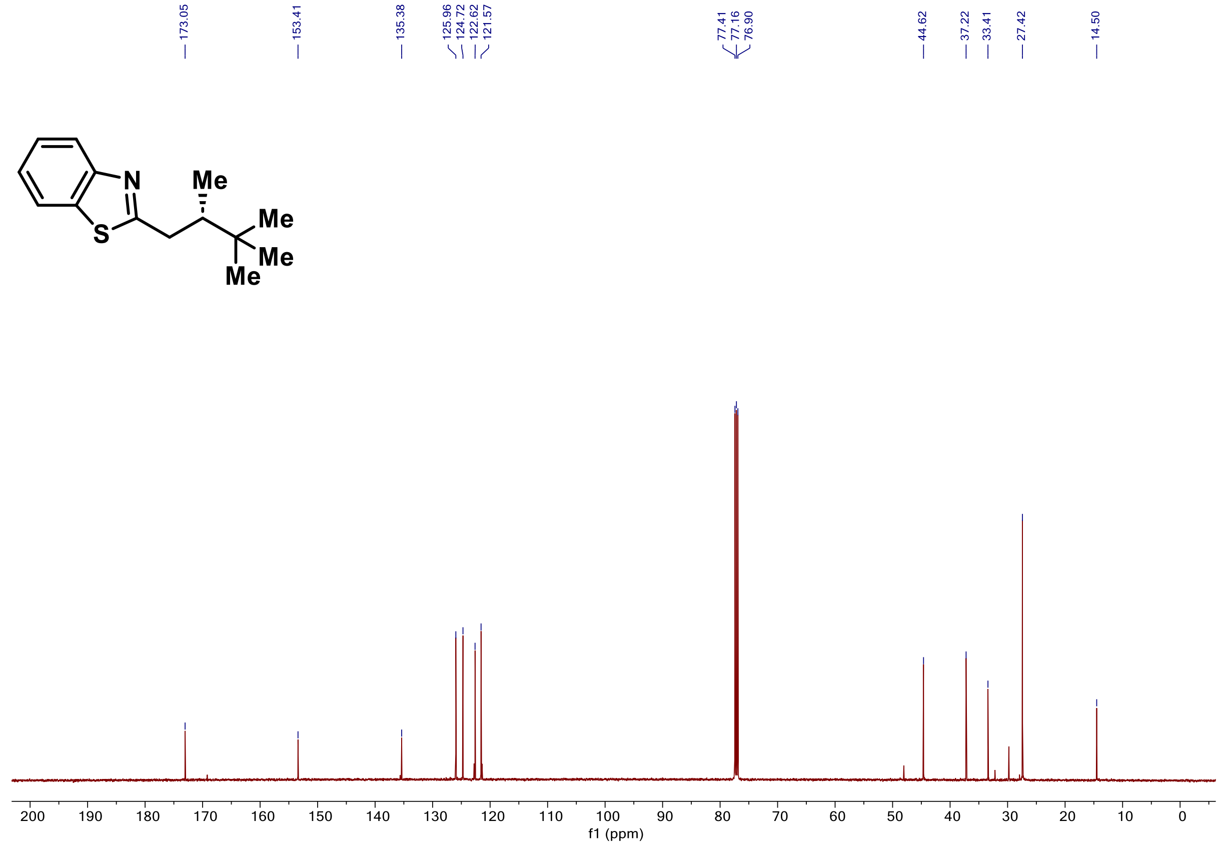

(*R*)-2-(2-Methyl-3-phenylpropyl)benzo[*d*]thiazole (4gn)

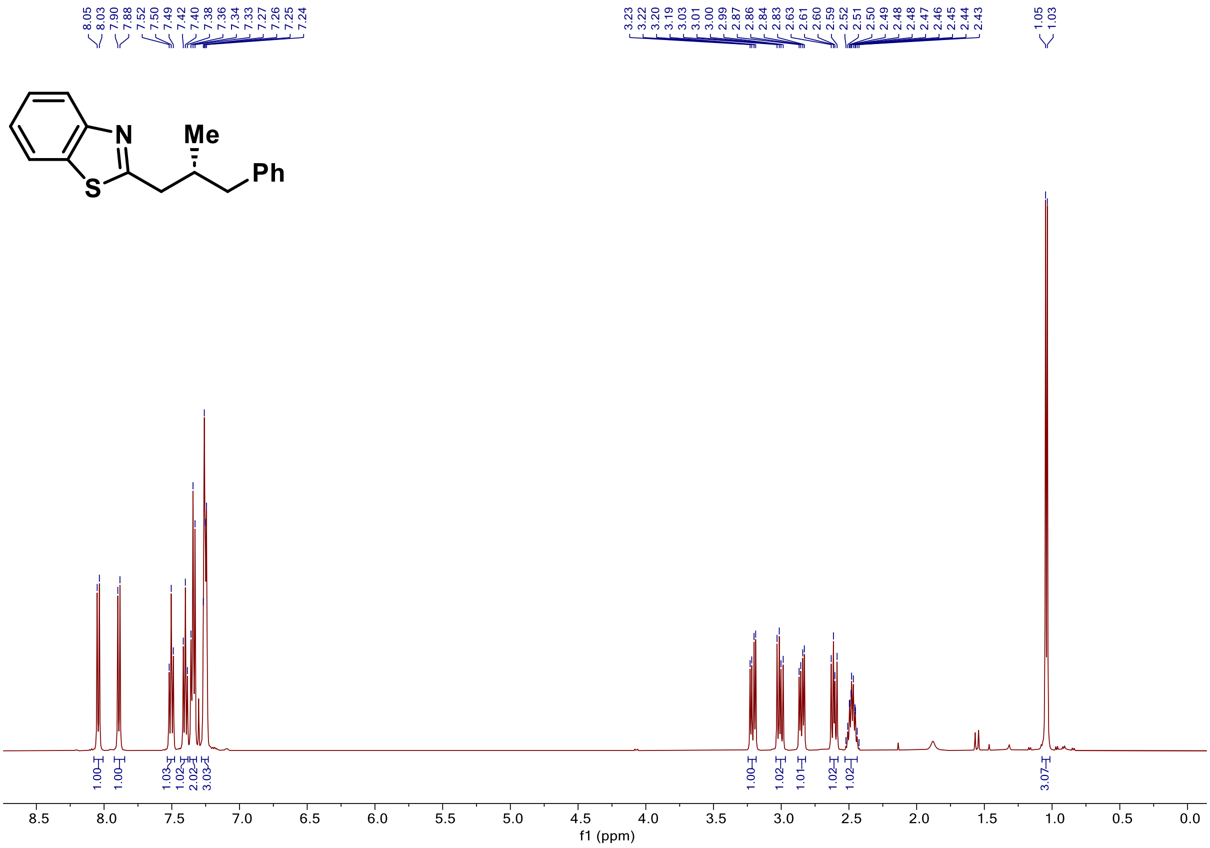

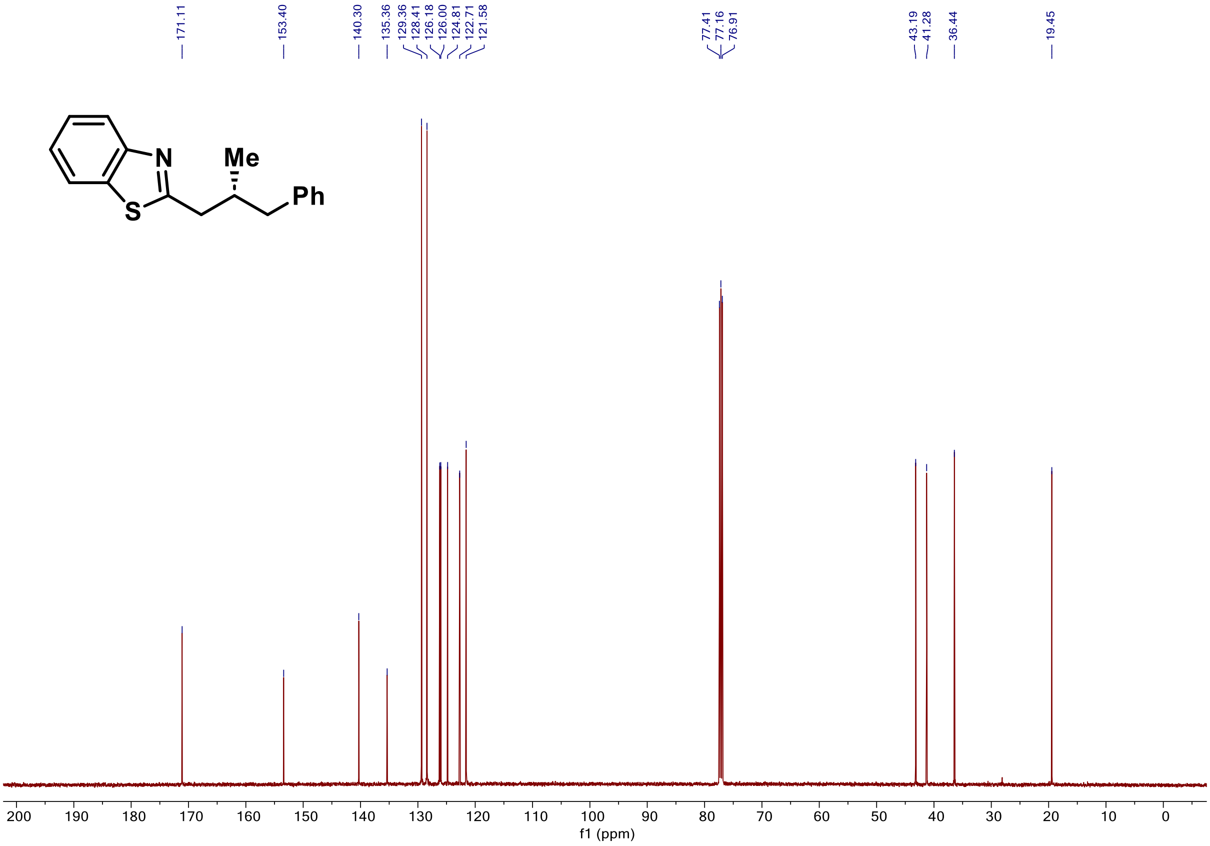

(*R*)-2-(2-Methyl-4-phenylbutyl)benzo[*d*]thiazole (4go)

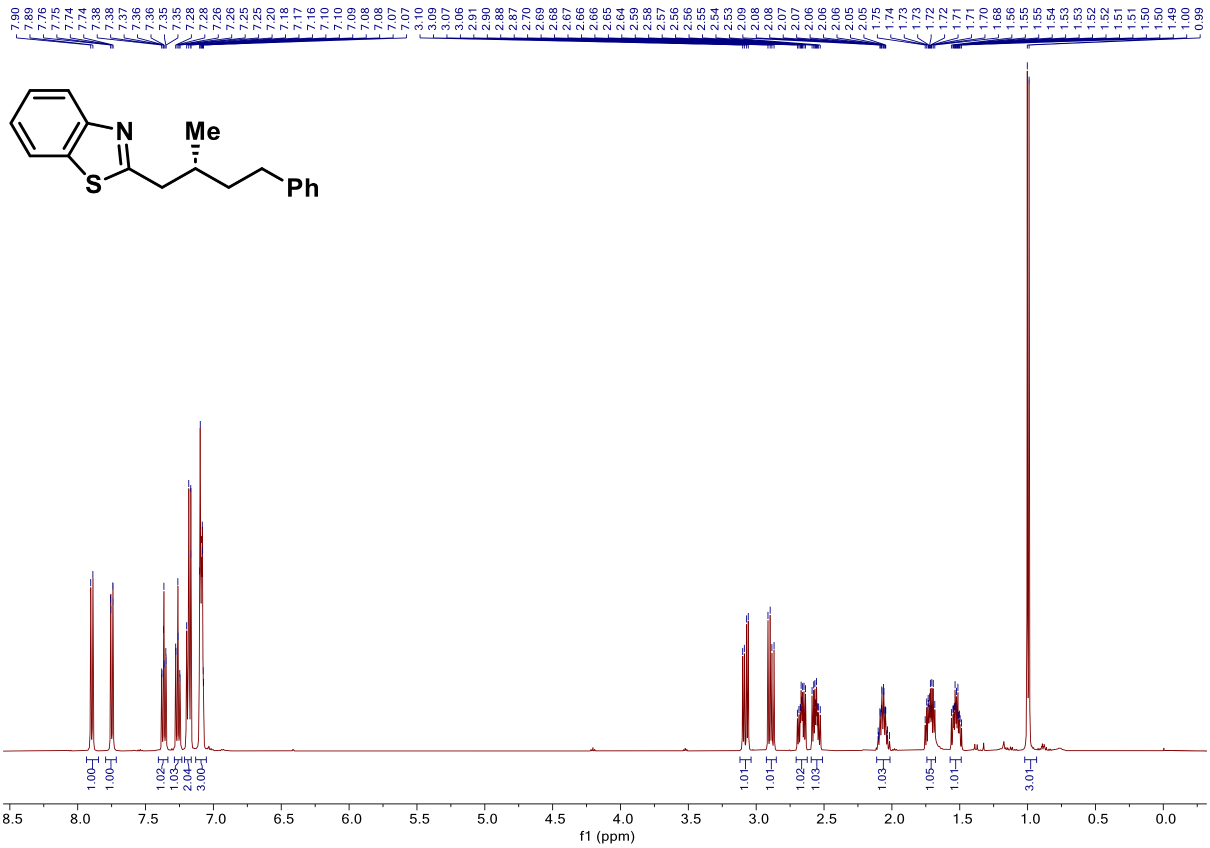

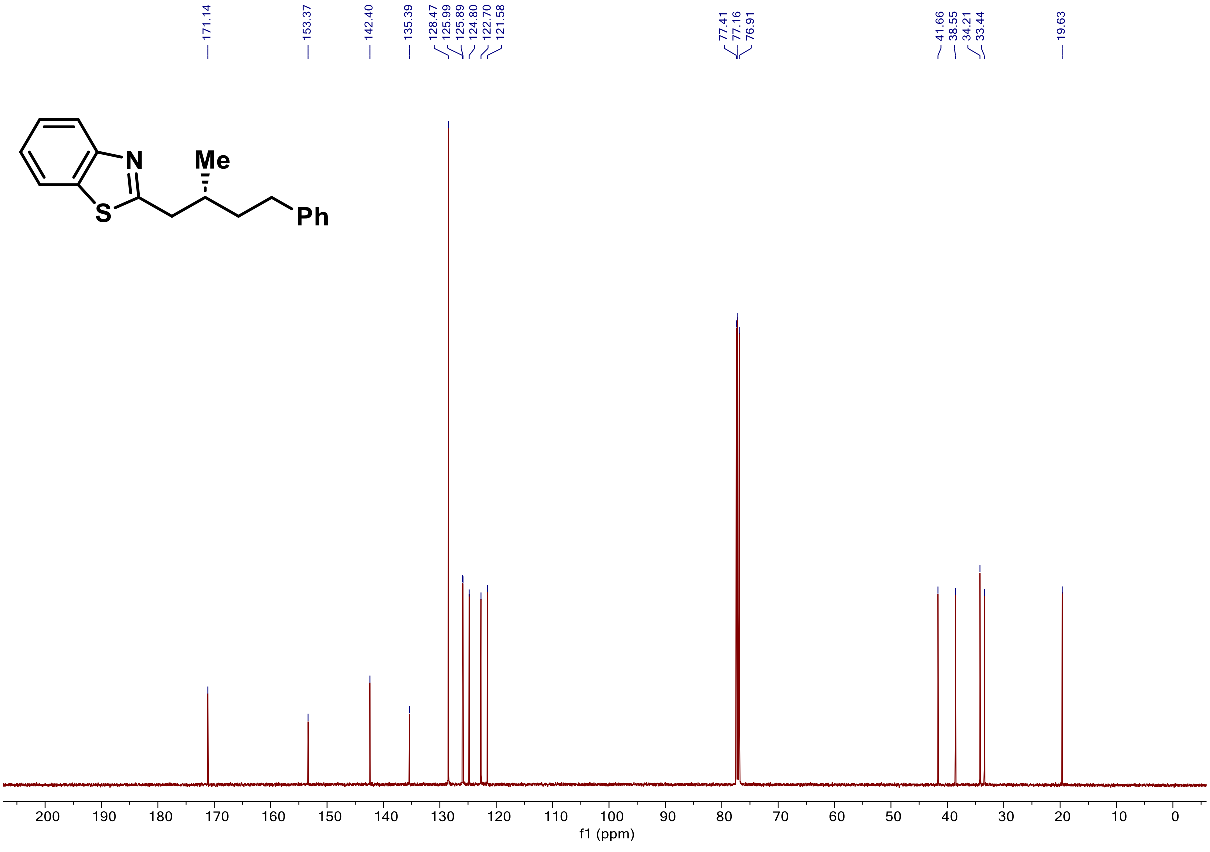

(*R*)-6-(Benzo[*d*]thiazol-2-yl)-5-methylhexyl benzoate (4gp)

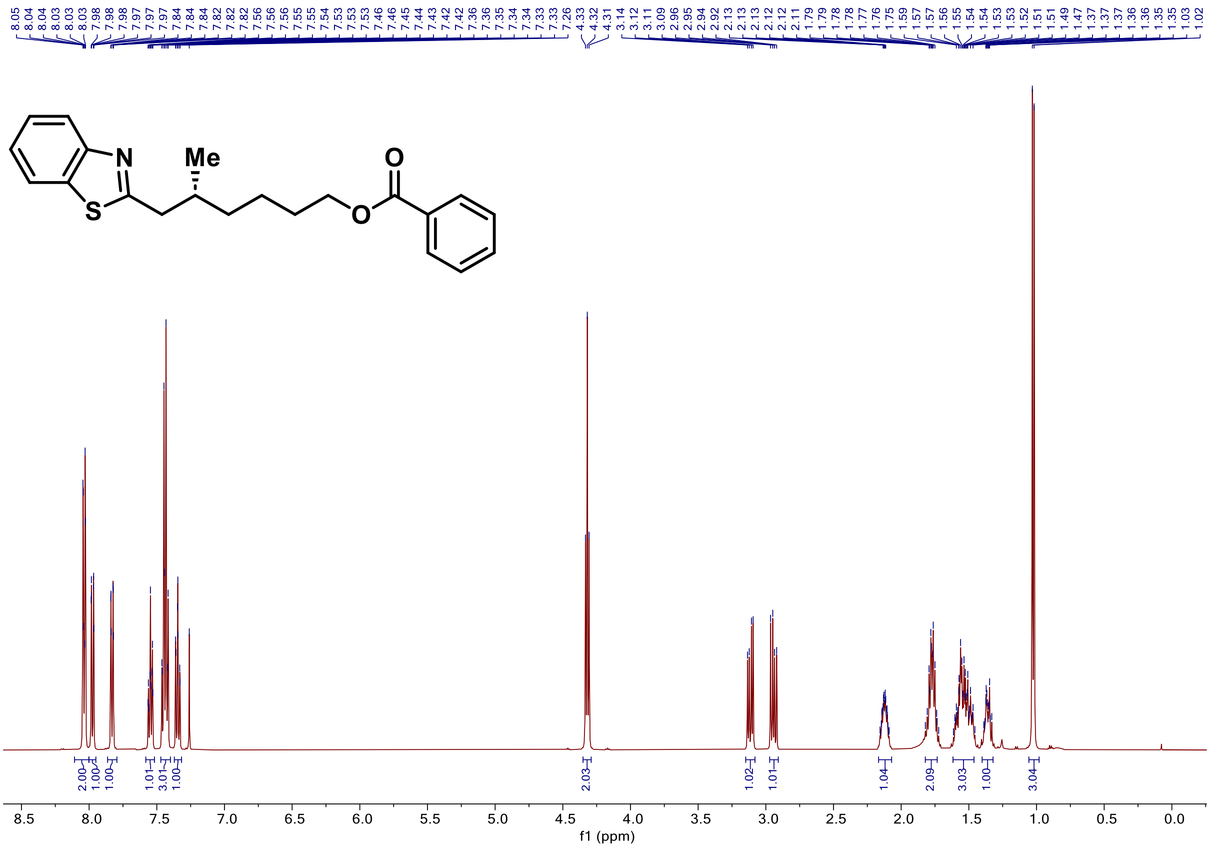

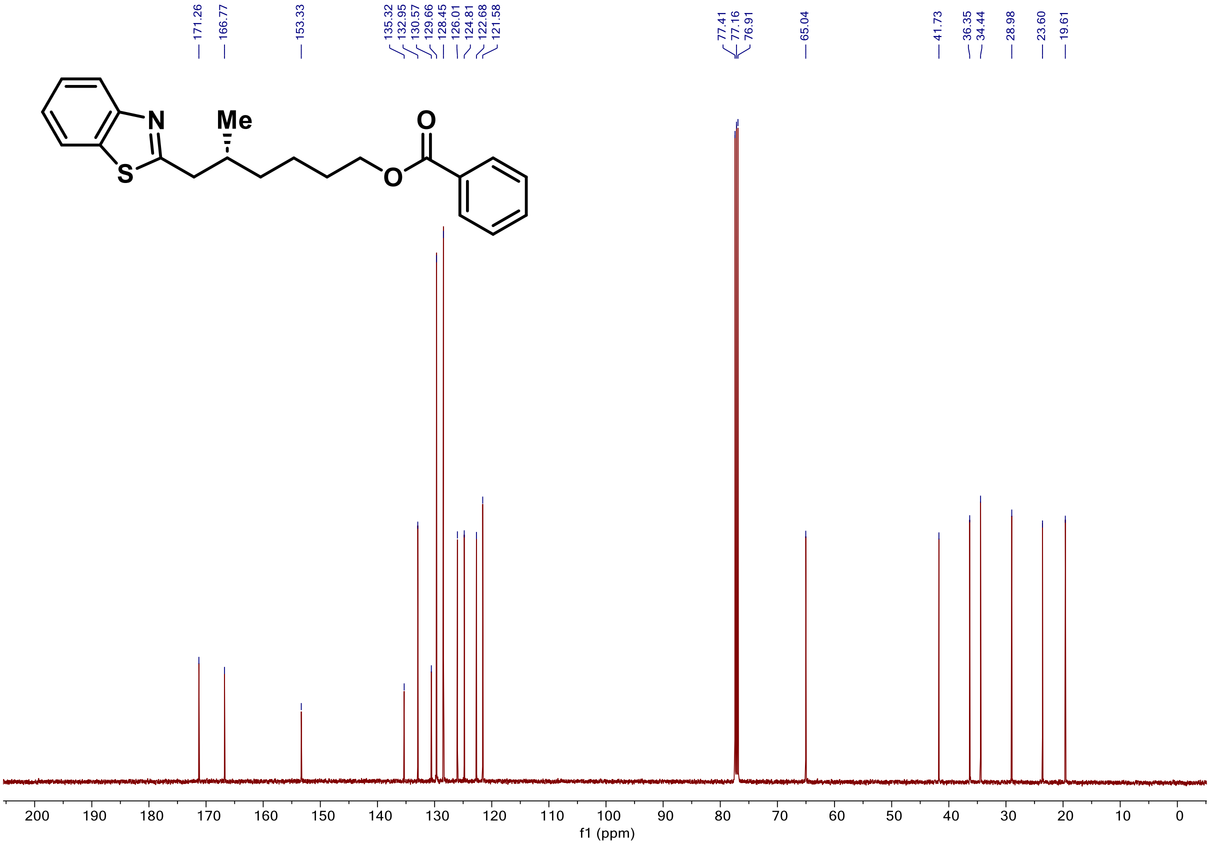

Ethyl (3*S*)-2-(benzo[*d*]thiazol-2-yl)-3-phenylbutanoate (3ga')

Diastereomer 1

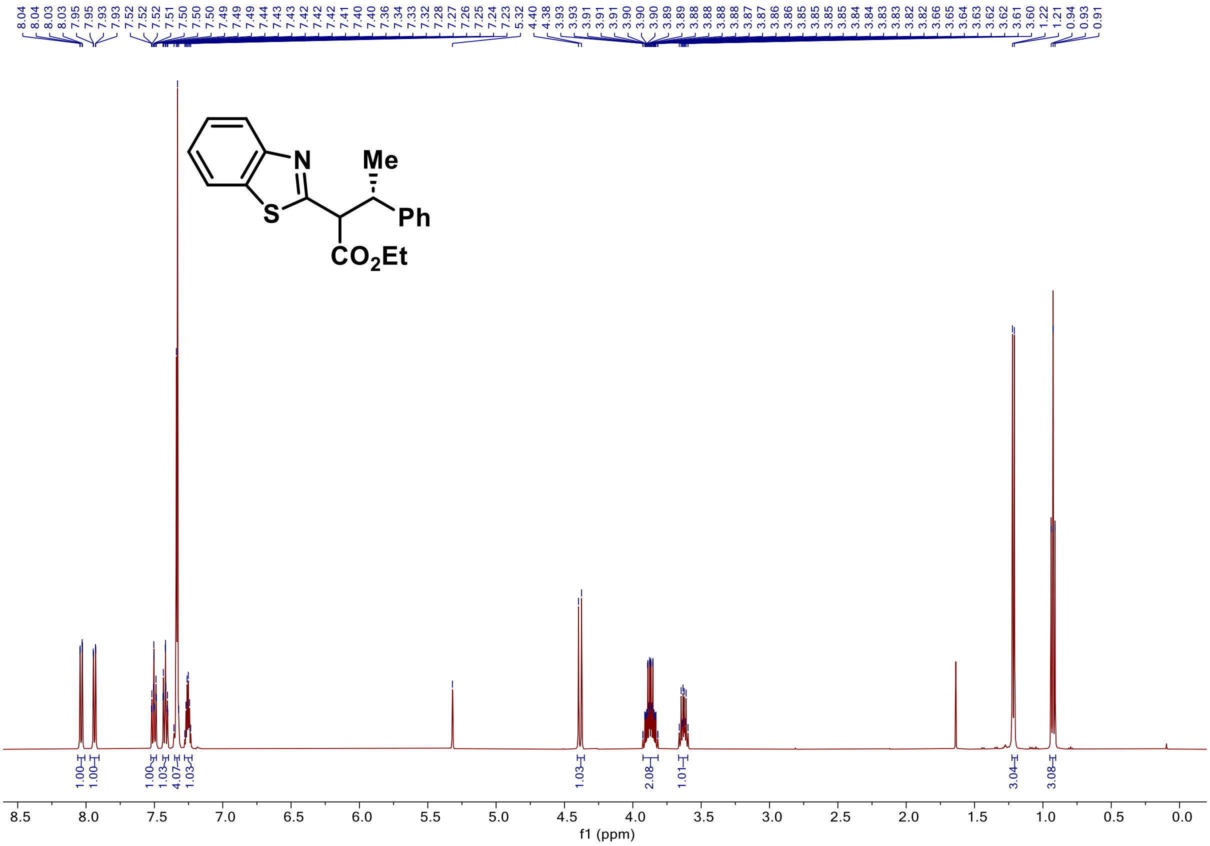

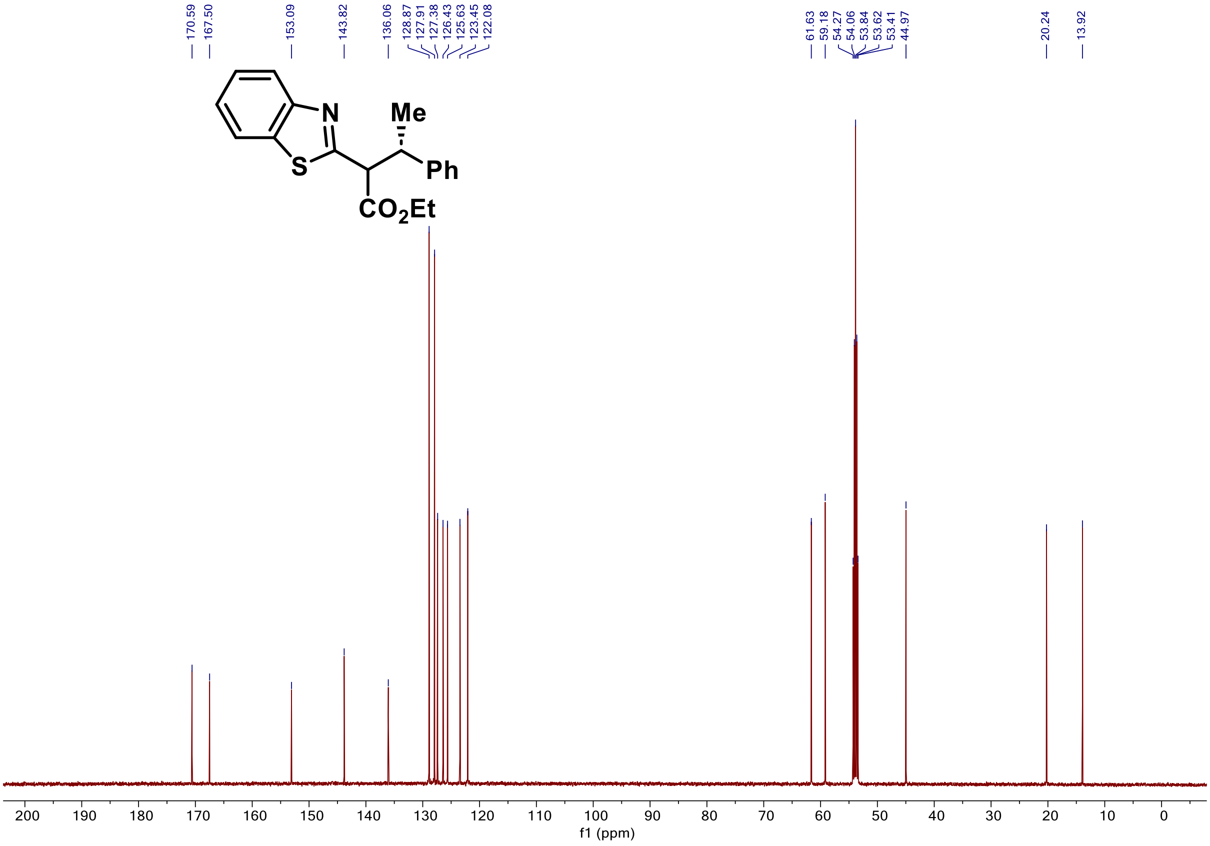

Diastereomer 2

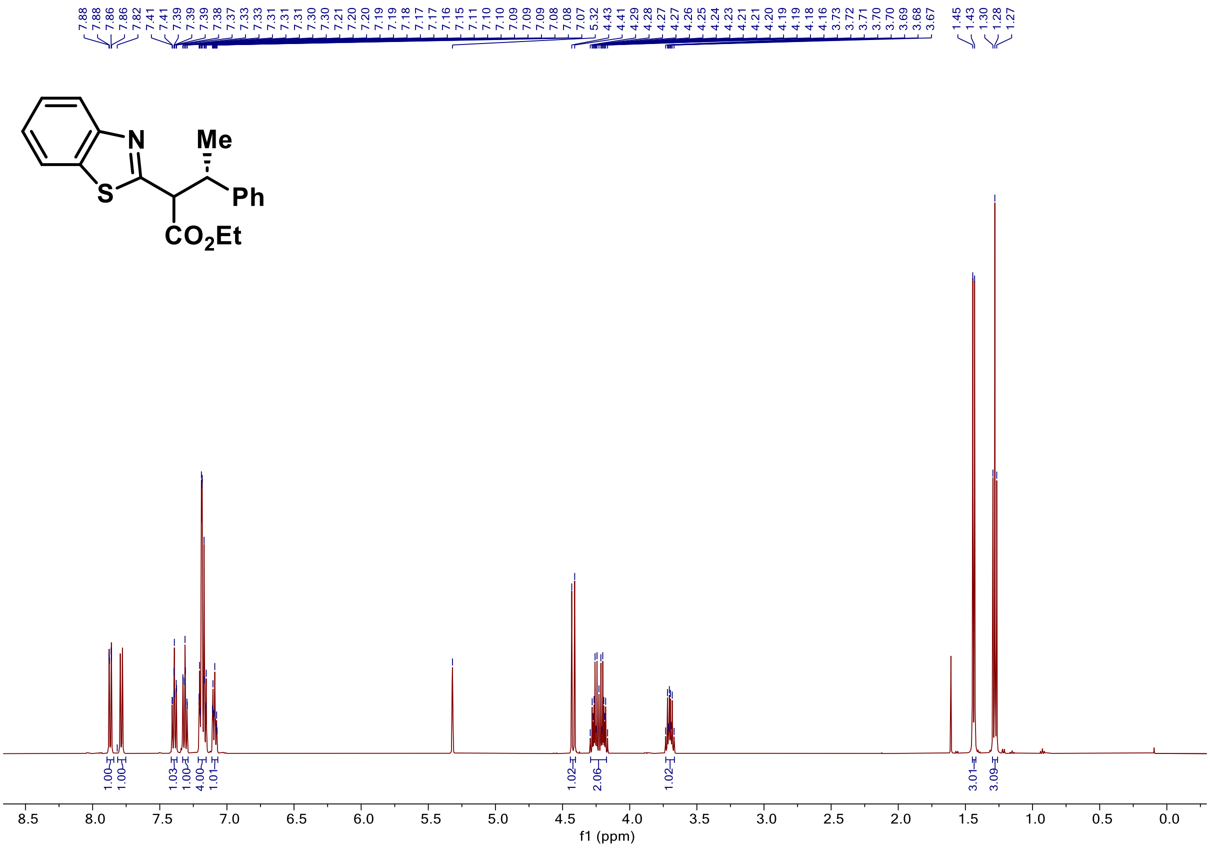

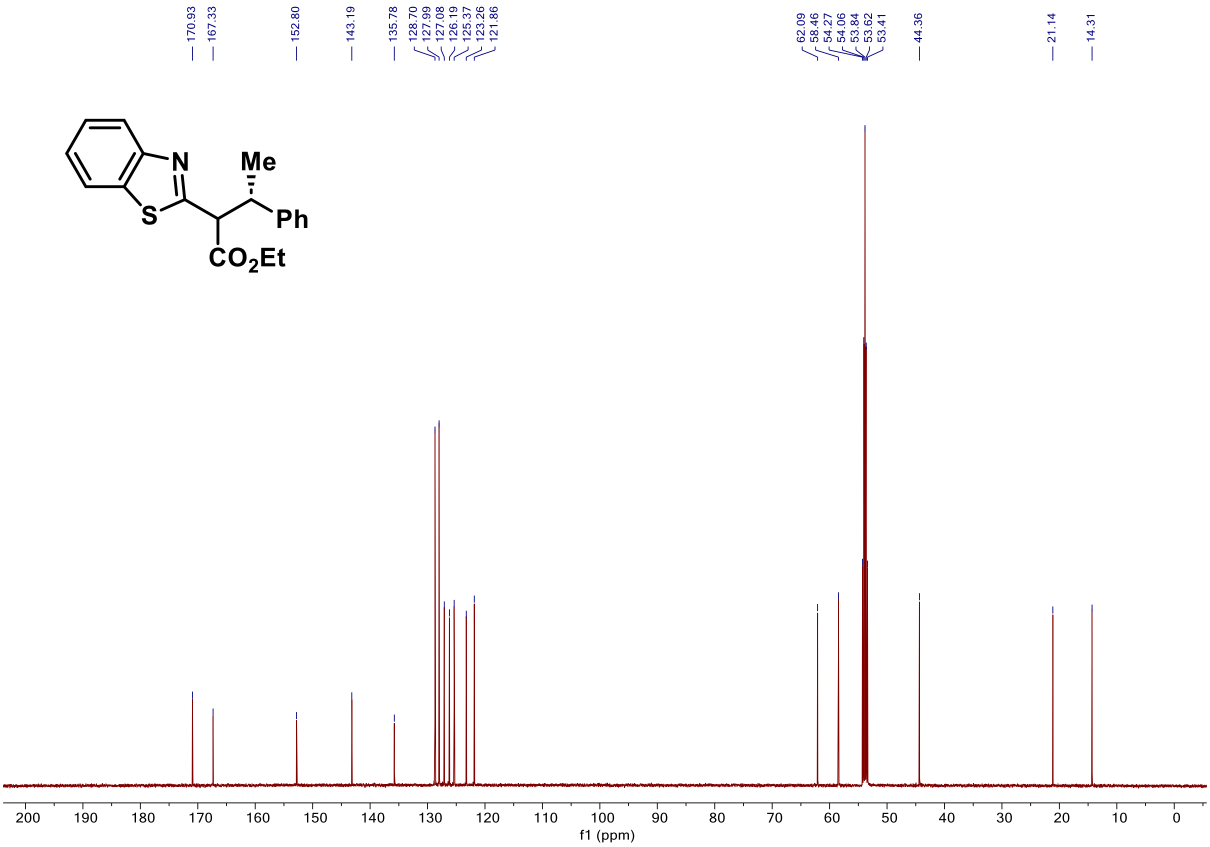

***tert*-Butyl (3*S*)-2-(benzo[*d*]thiazol-2-yl)-3-phenylbutanoate (3ga)**

Diastereomer 1

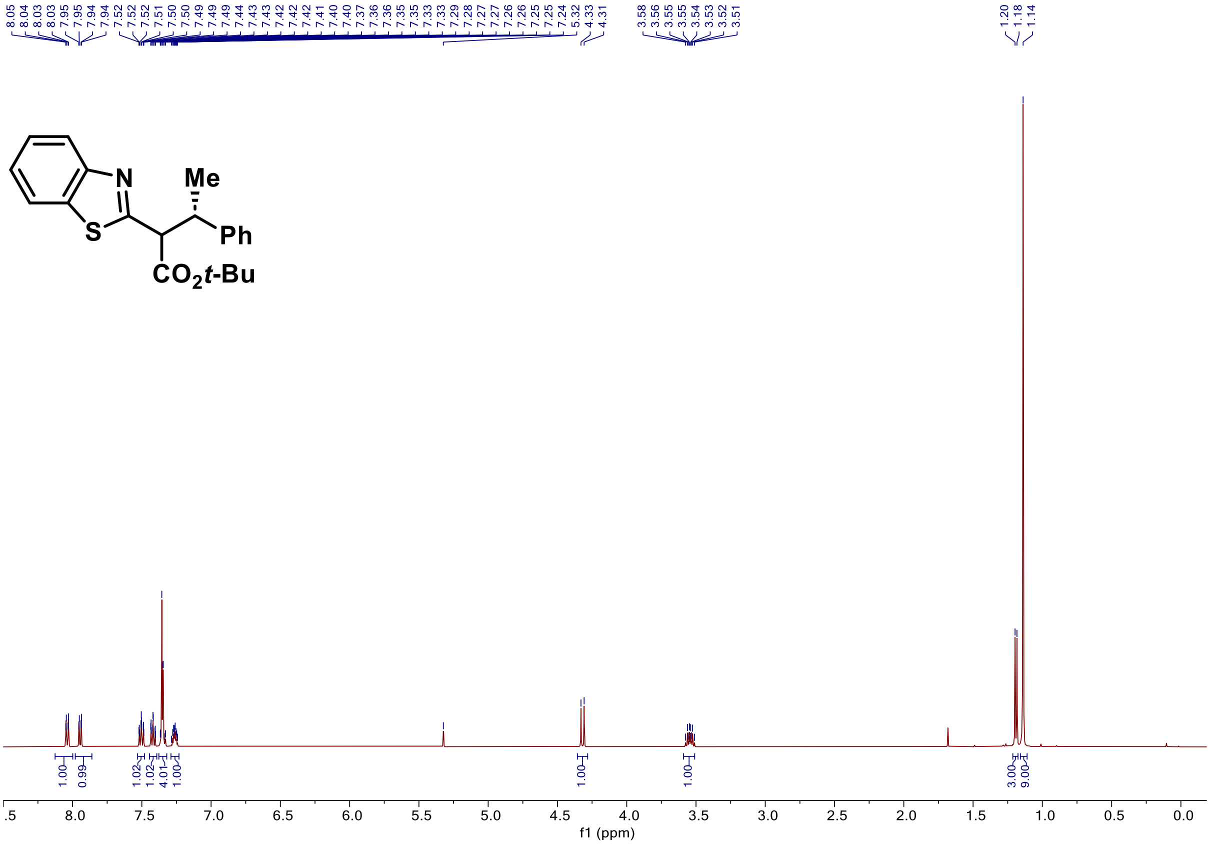

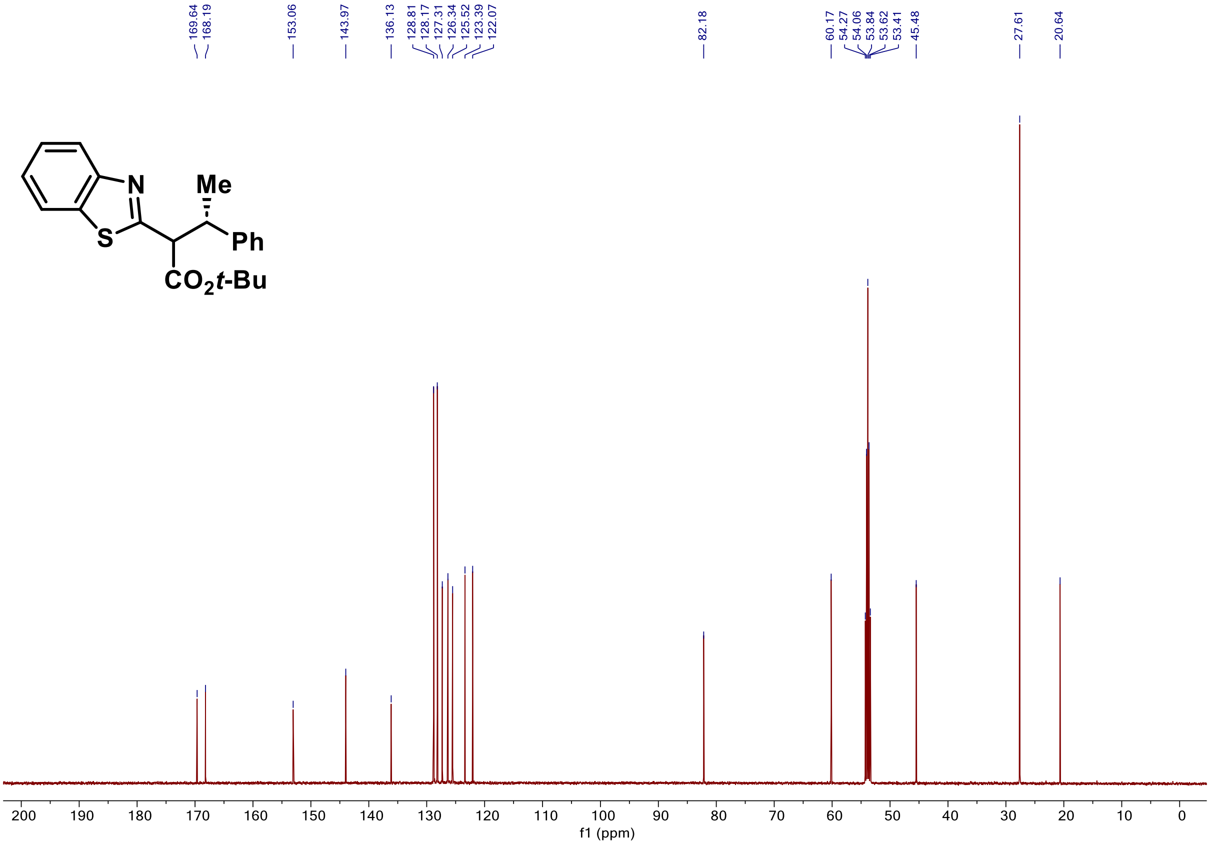

Diastereomer 2

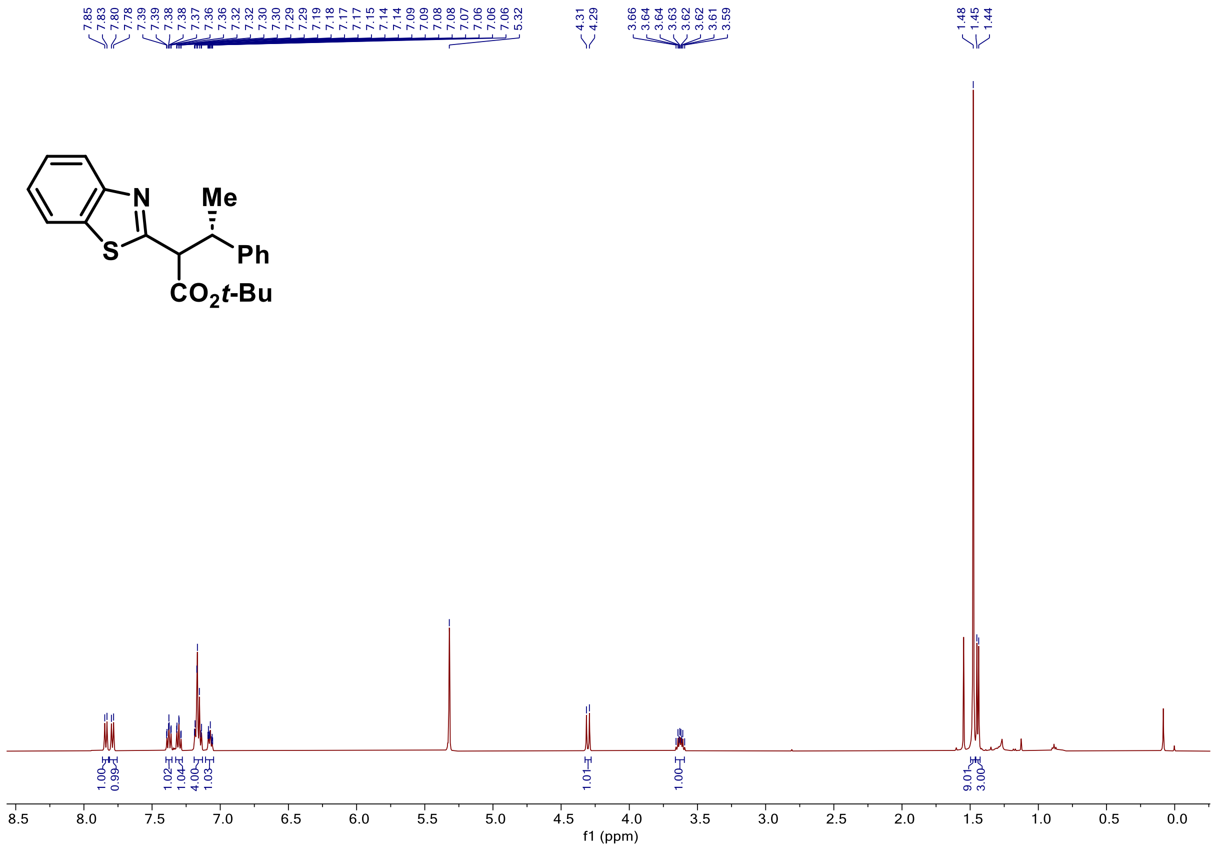

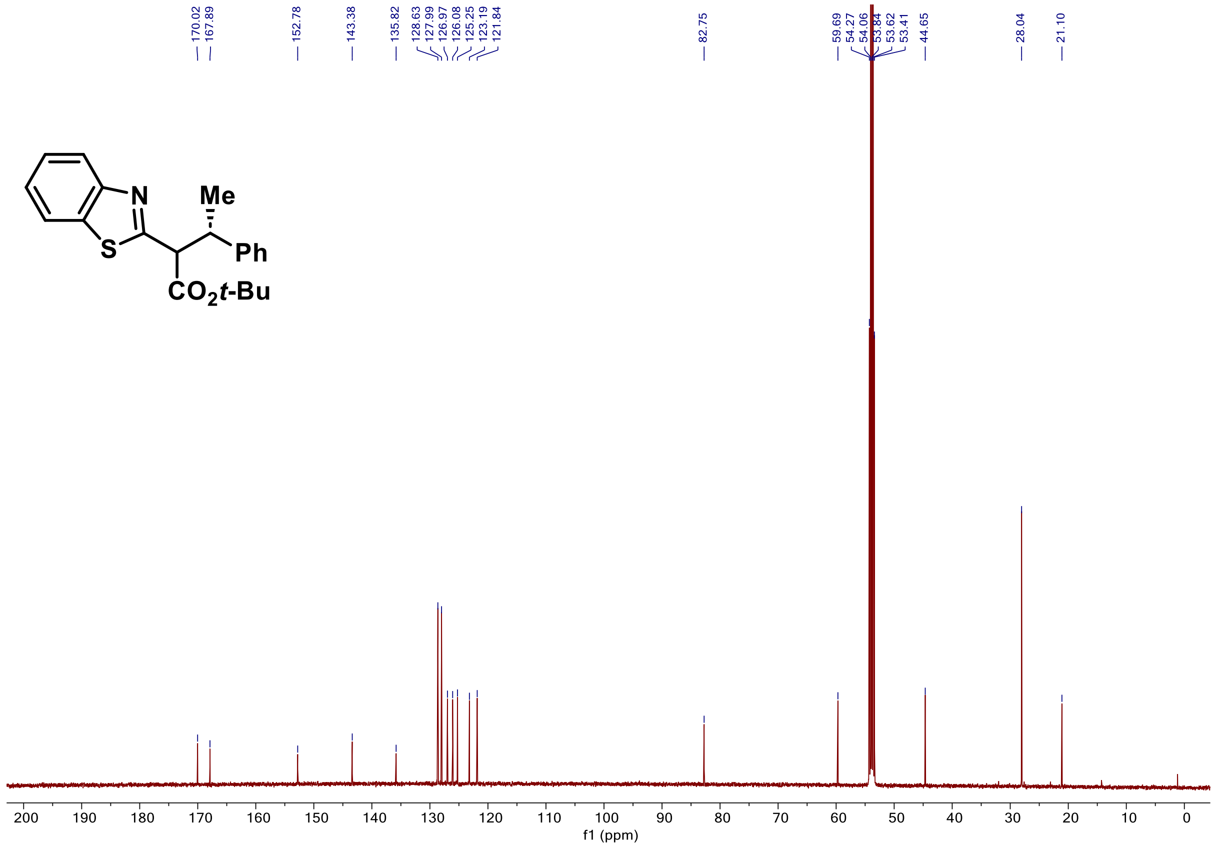

tert-Pentyl 2-(benzo[_d_]thiazol-2-yl)acetate (1g'')

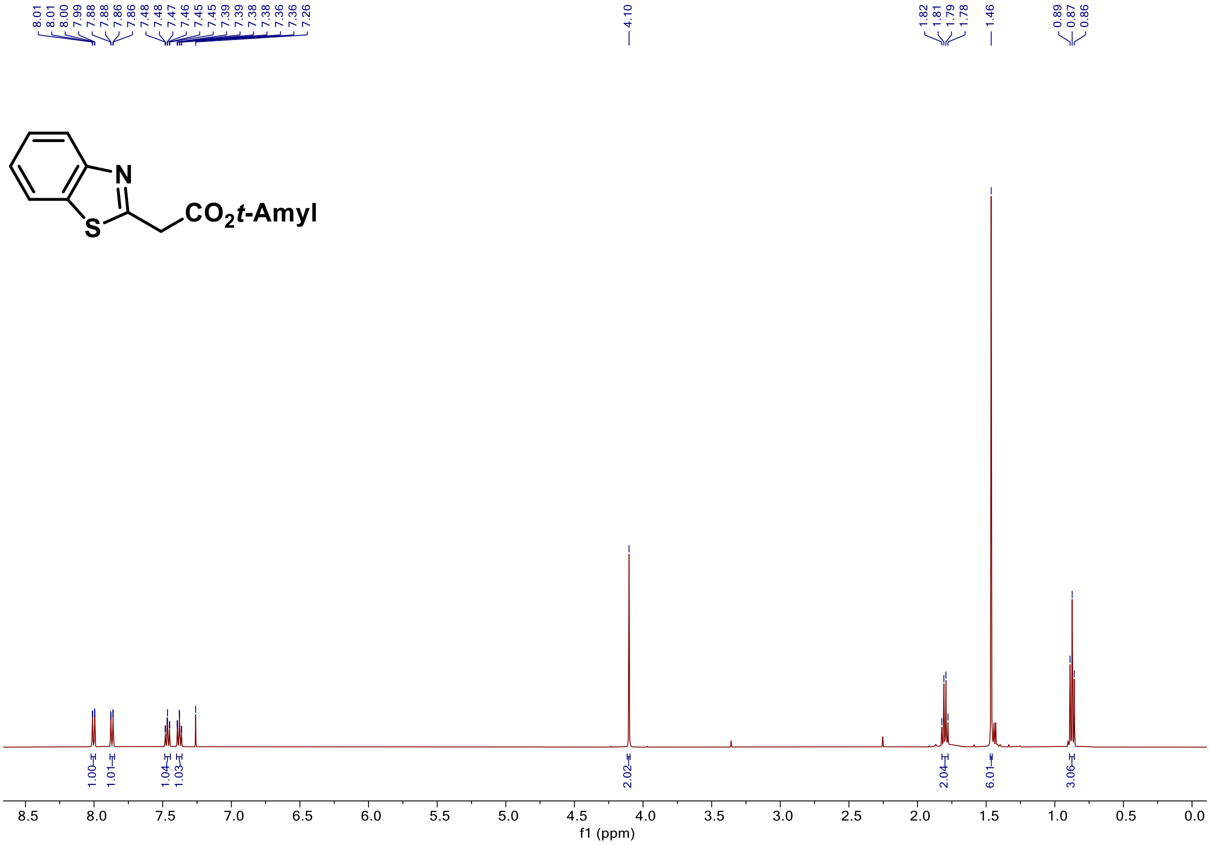

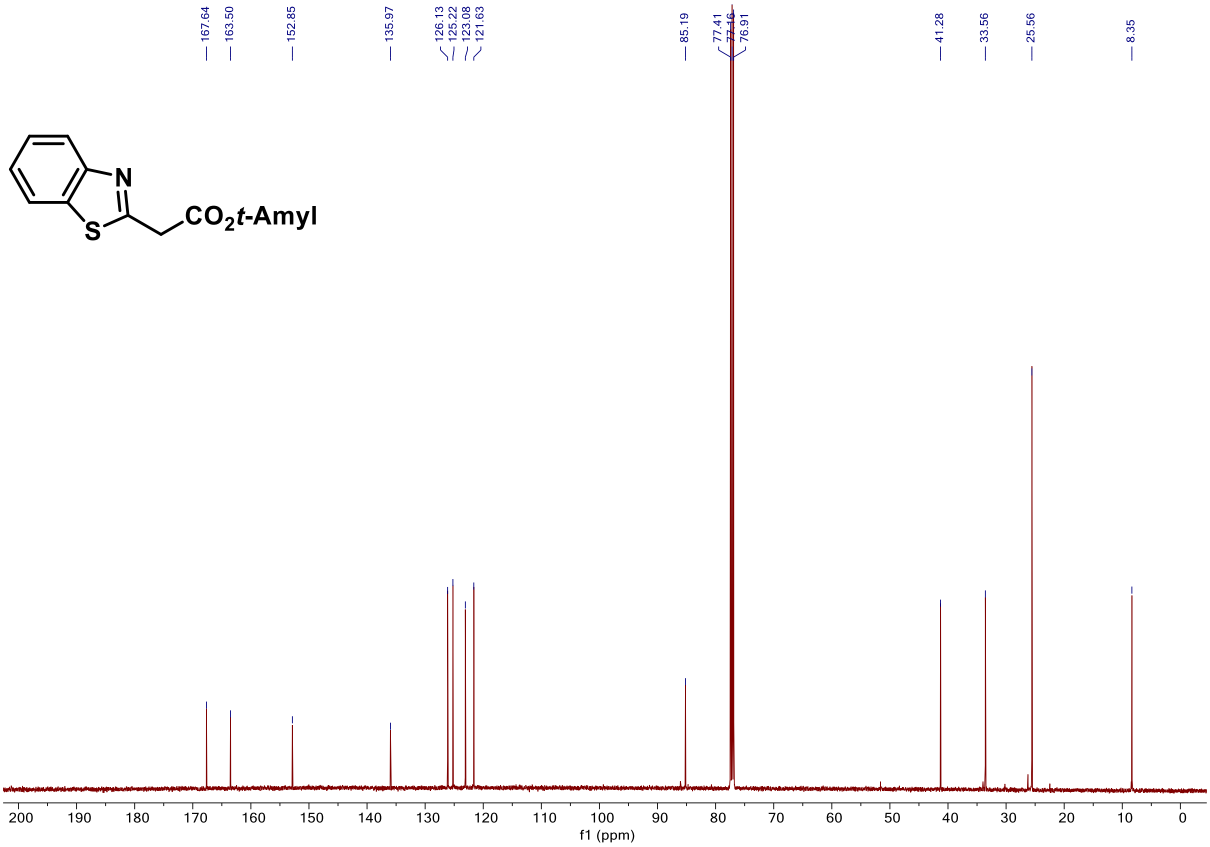

tert-Pentyl (3_S_)-2-(benzo[_d_]thiazol-2-yl)-3-phenylbutanoate (3ga'')

Diastereomer 1

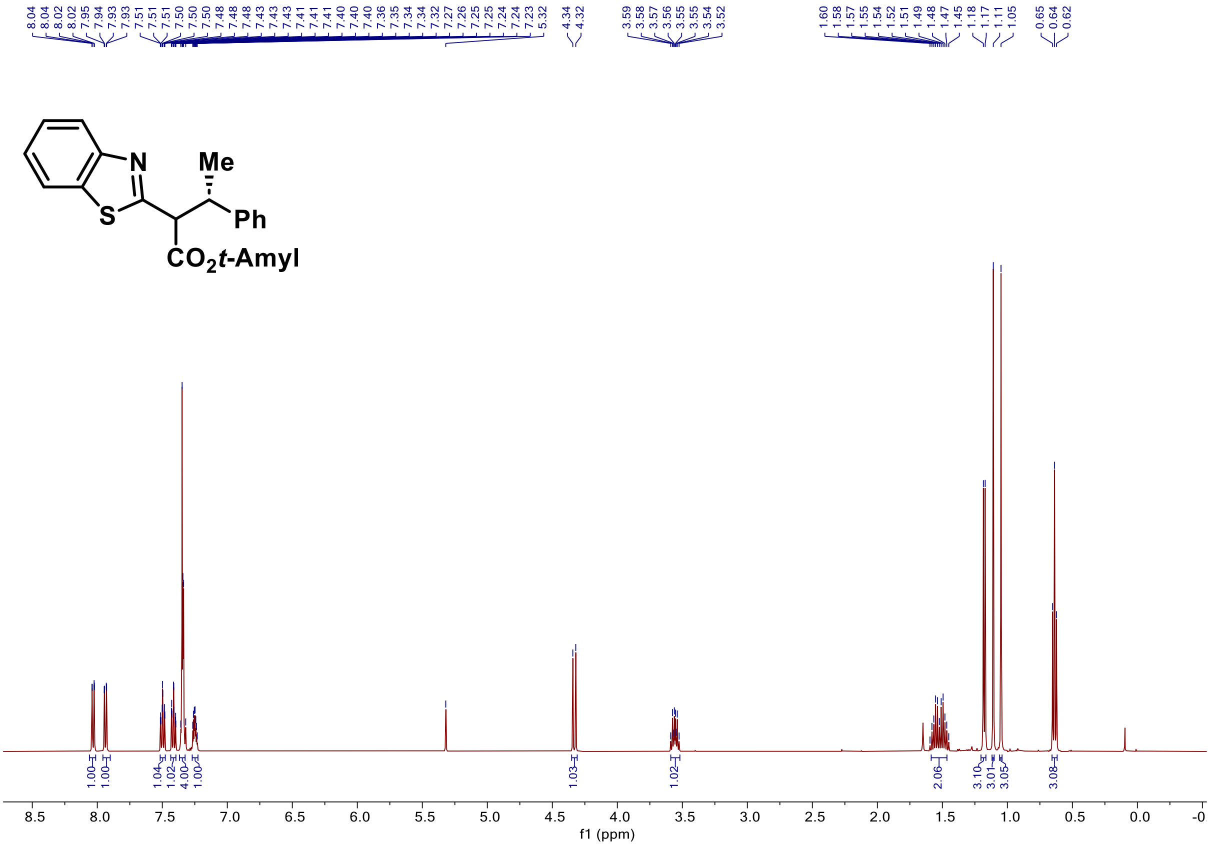

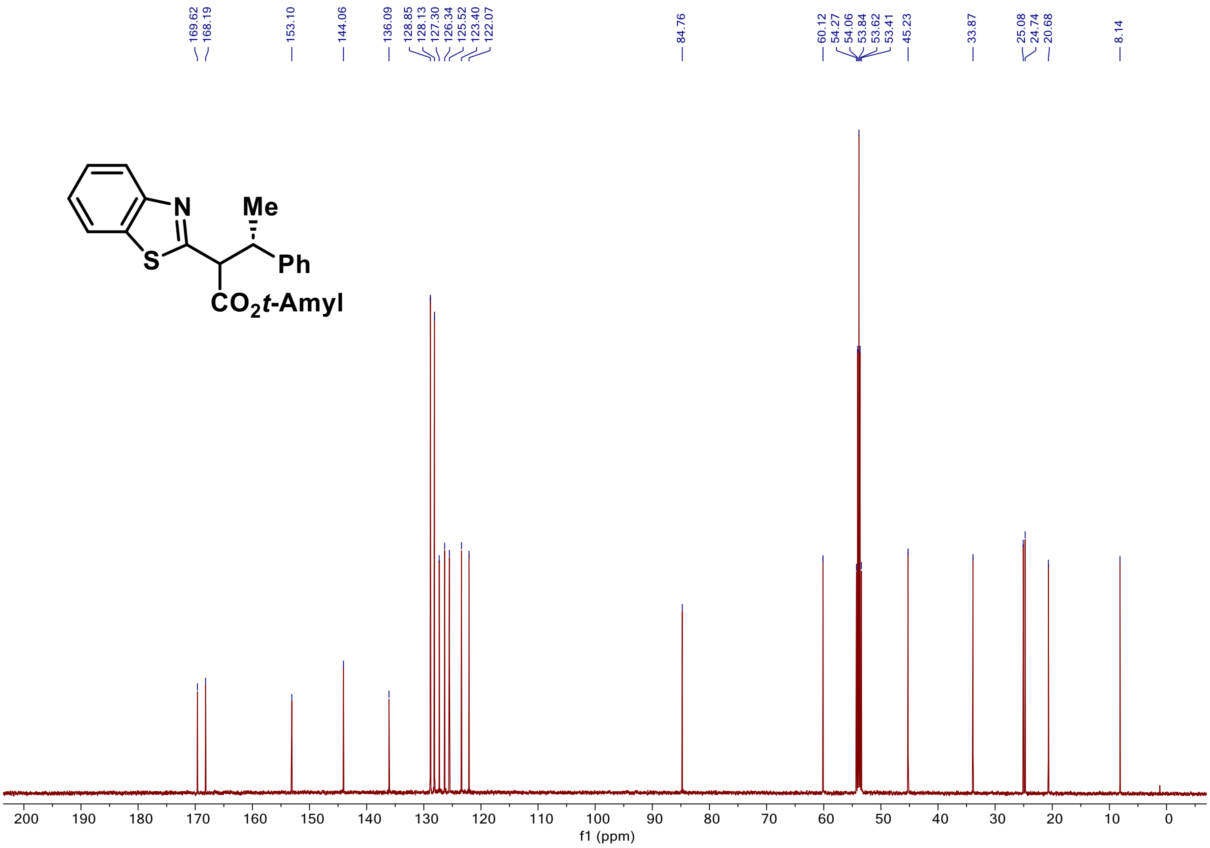

Diastereomer 2

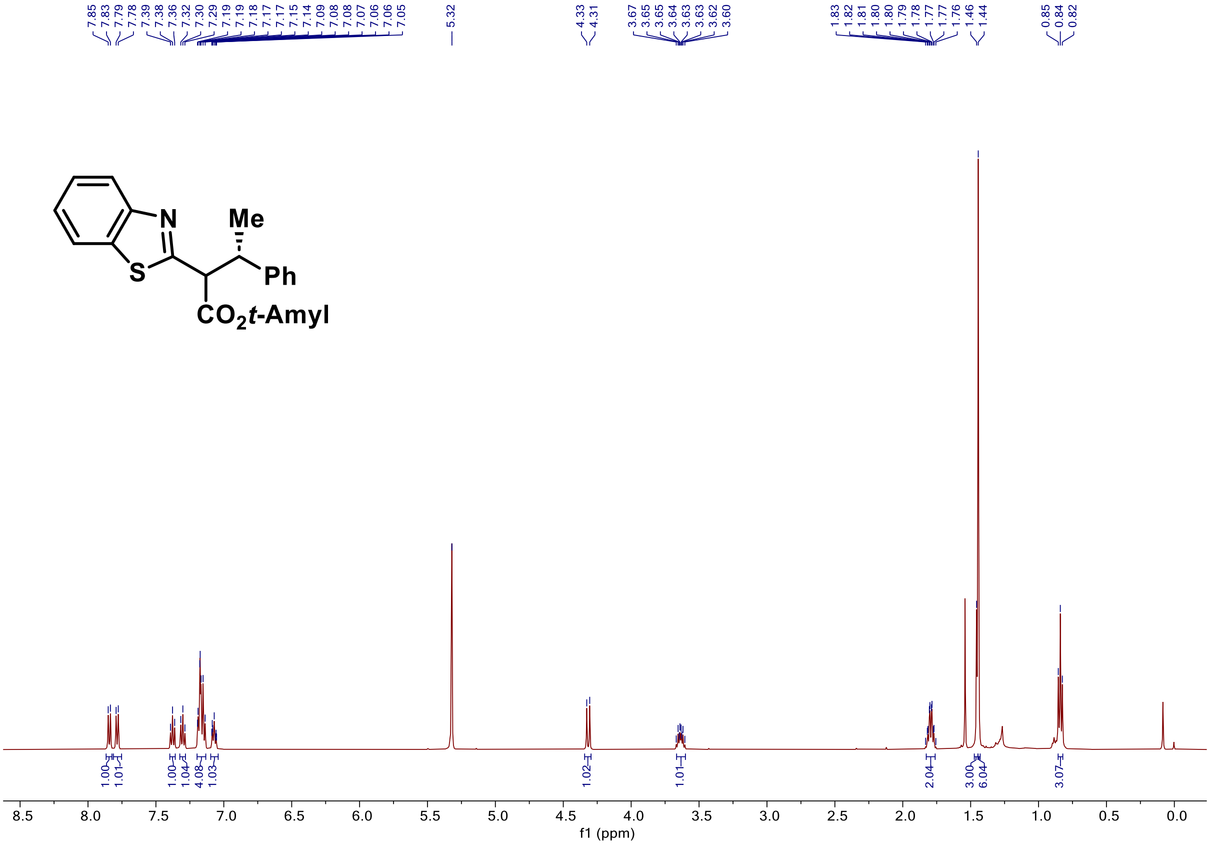

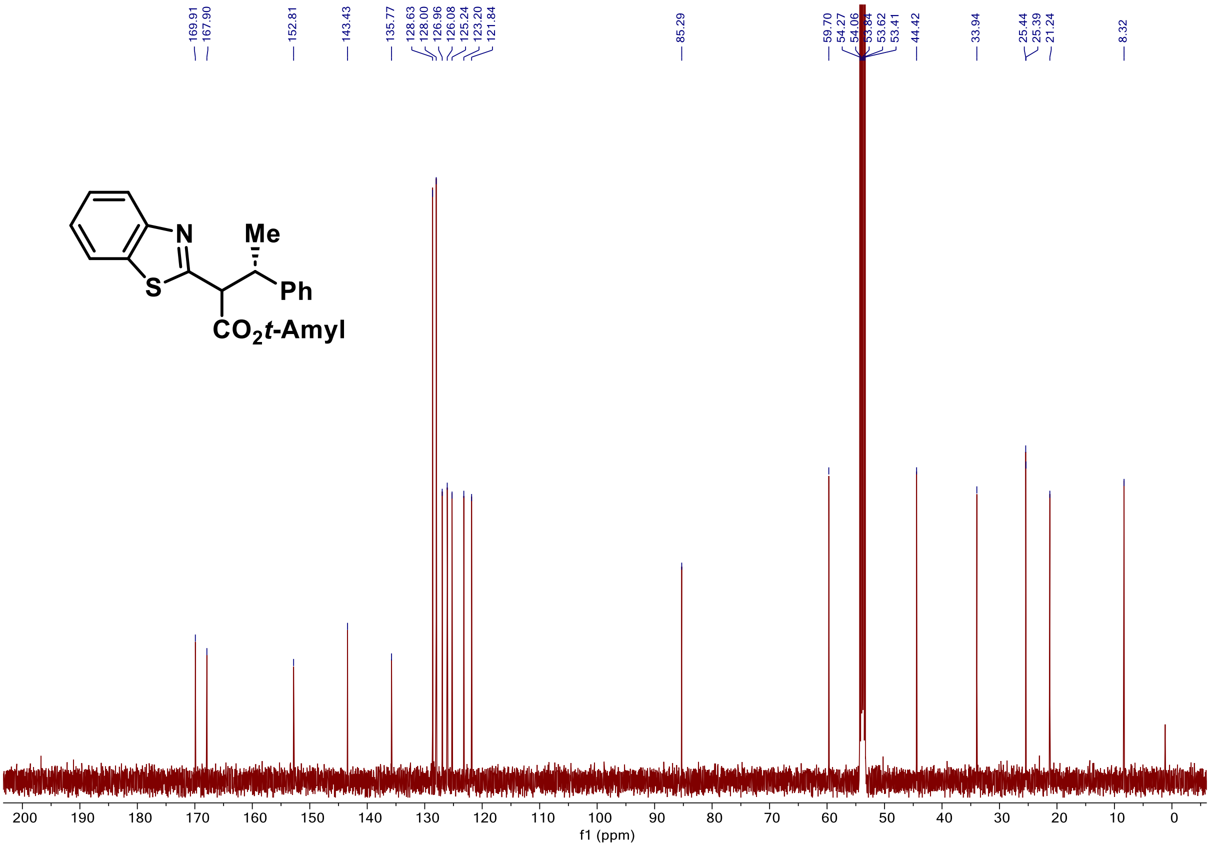

(3*S*)-2-(Benzo[*d*]thiazol-2-yl)-*N*,*N*-dimethyl-3-phenylbutanamide (3ga''')

Diastereomer 1

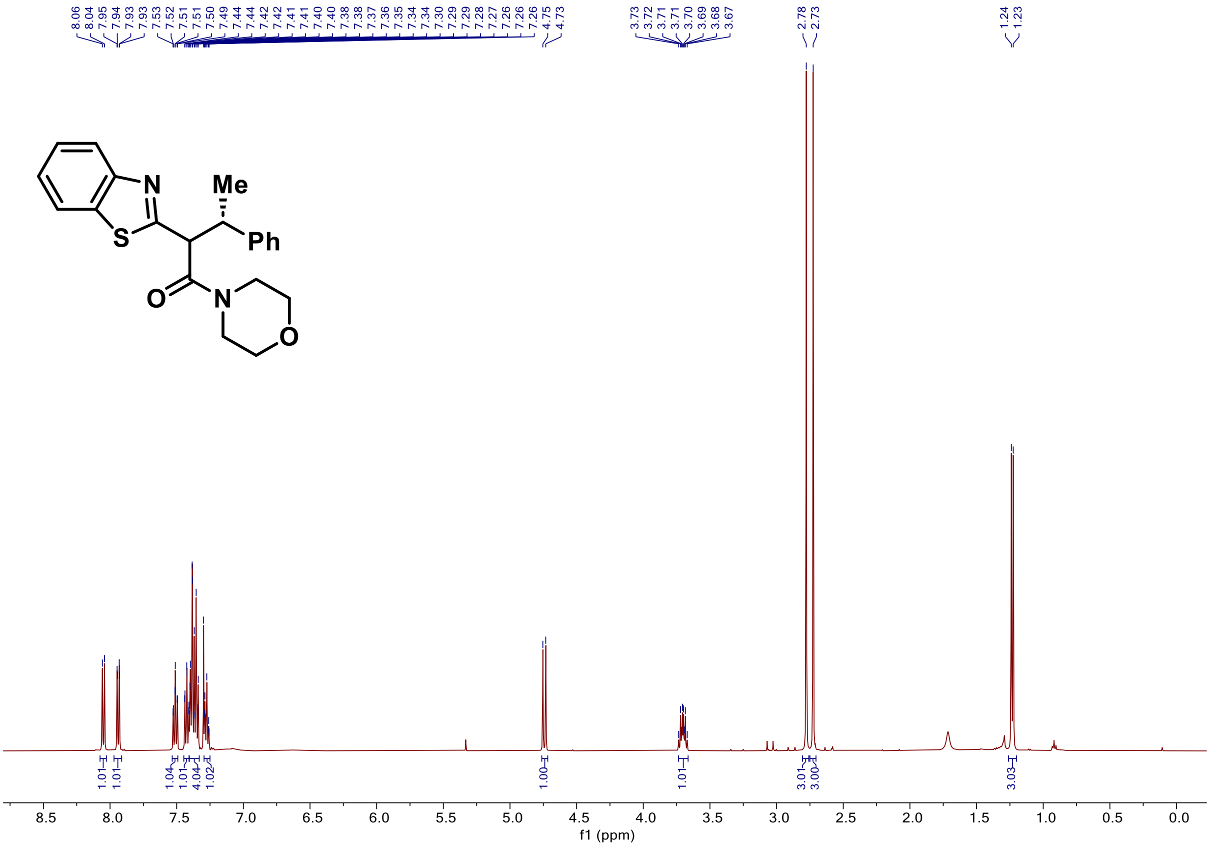

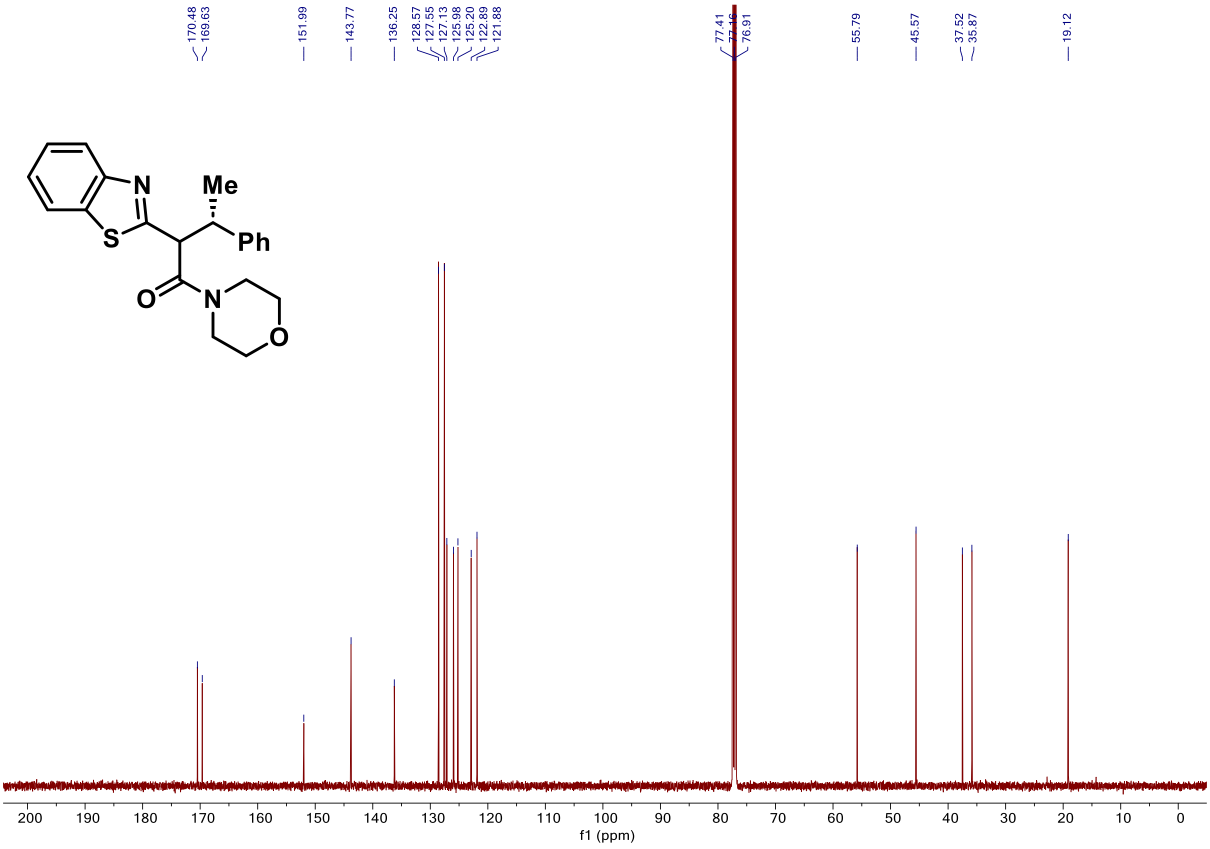

Diastereomer 2

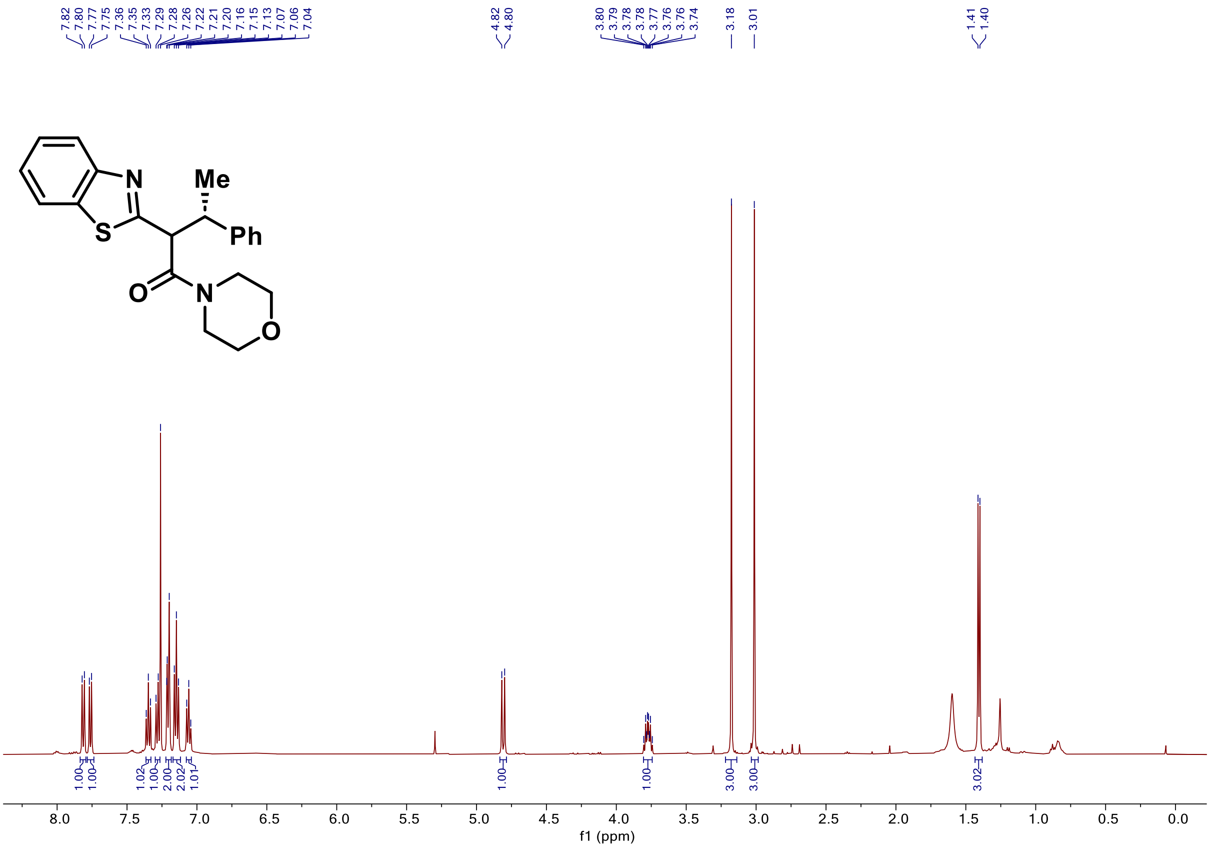

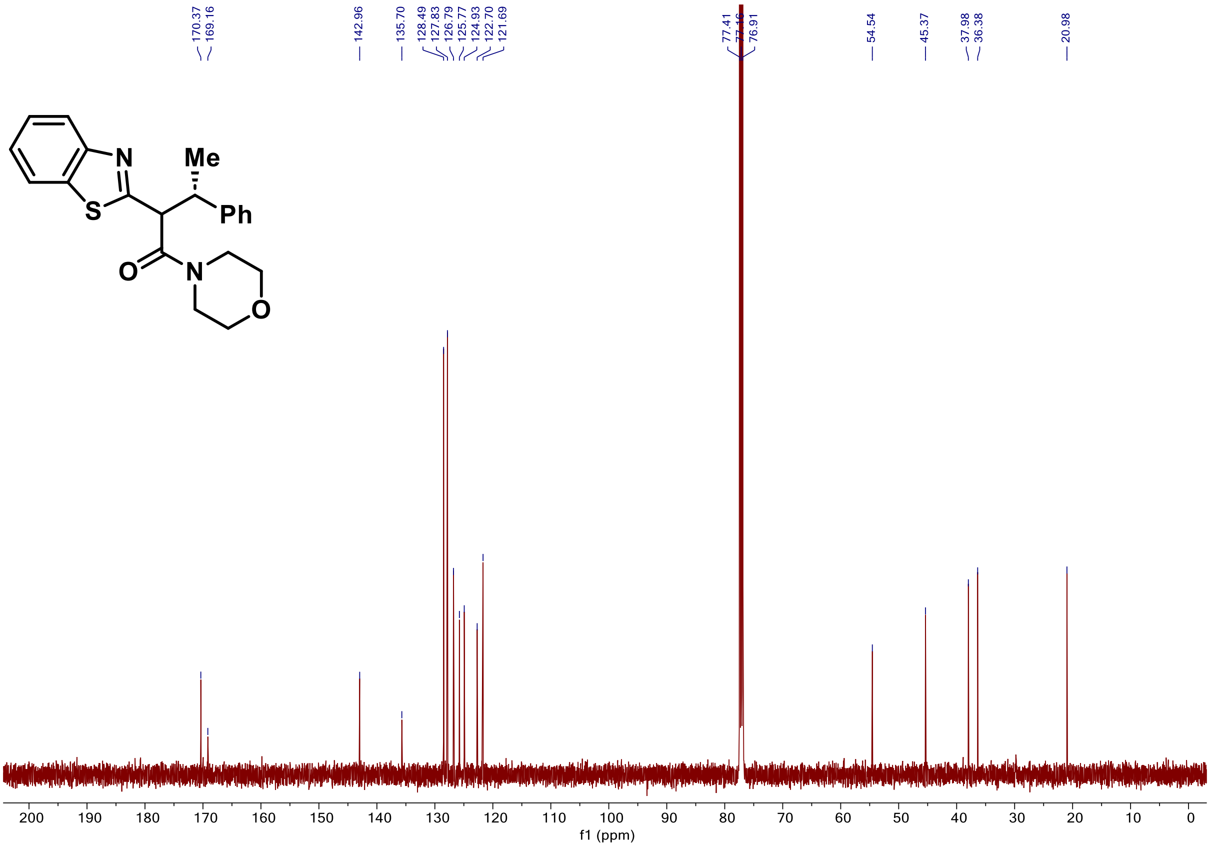

(3*S*)-2-(Benzo[*d*]thiazol-2-yl)-1-morpholino-3-phenylbutan-1-one (S3ga’’’’)

Diastereomer 1

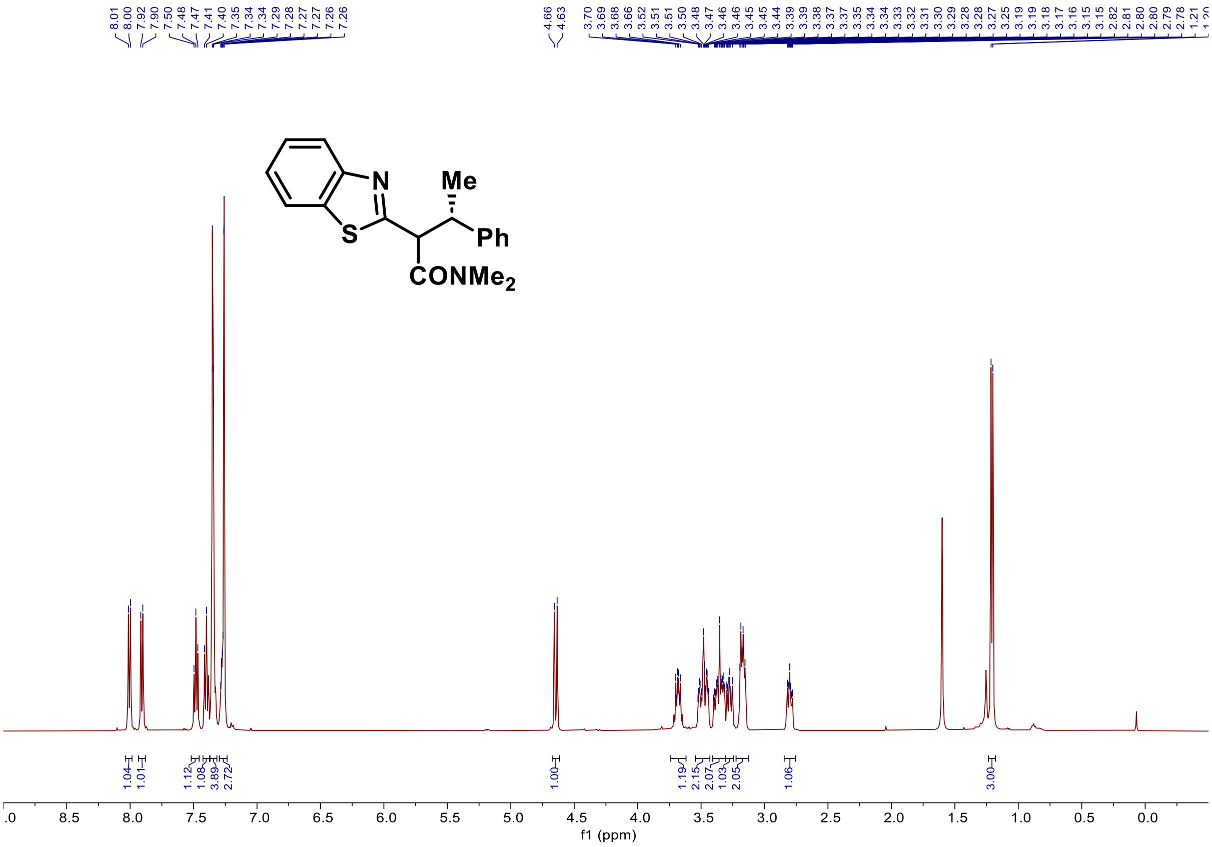

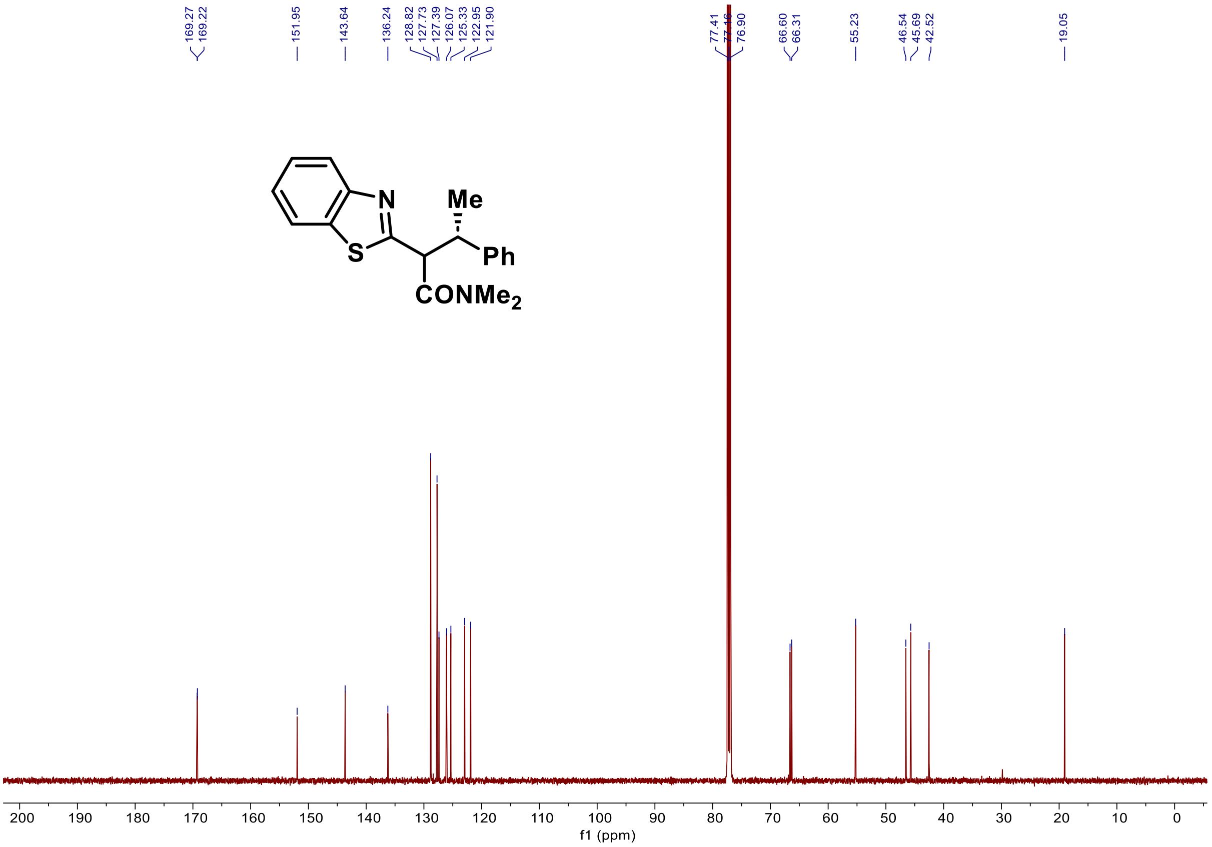

Diastereomer 2

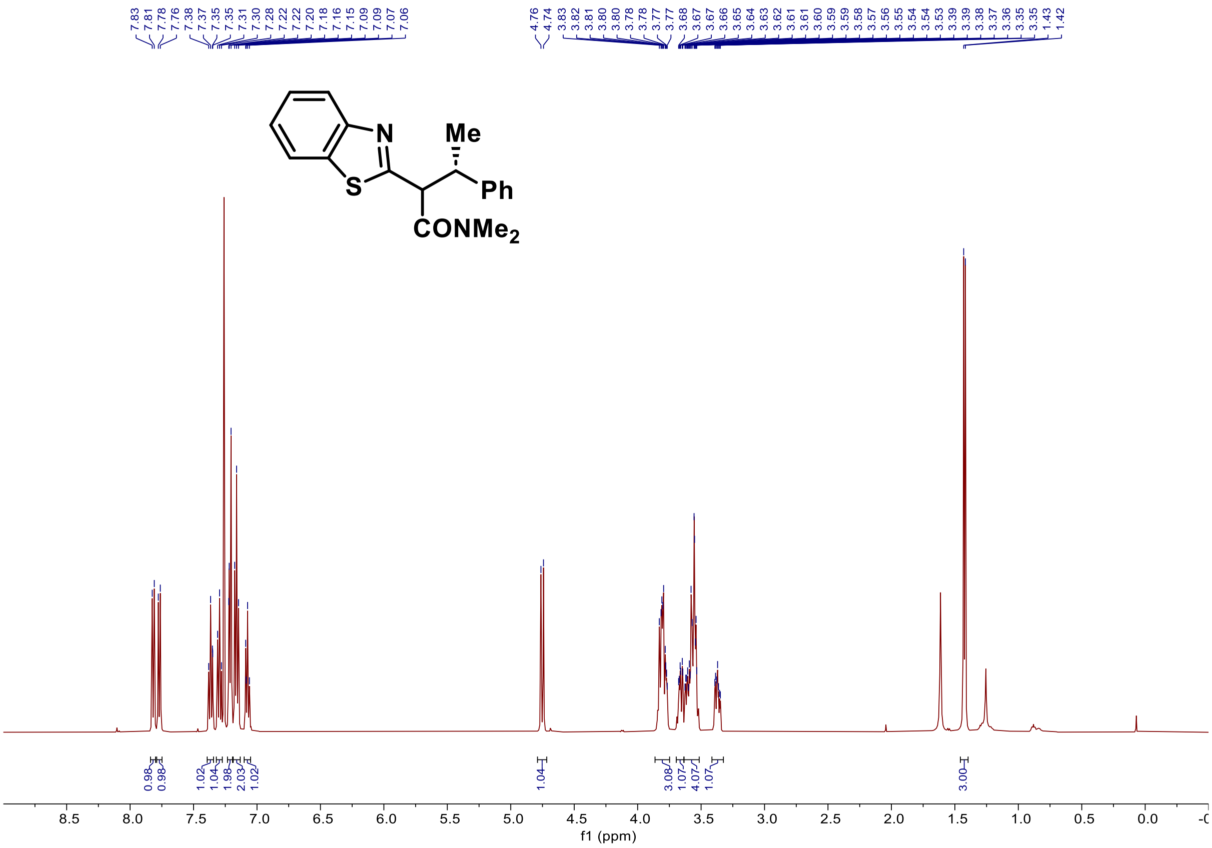

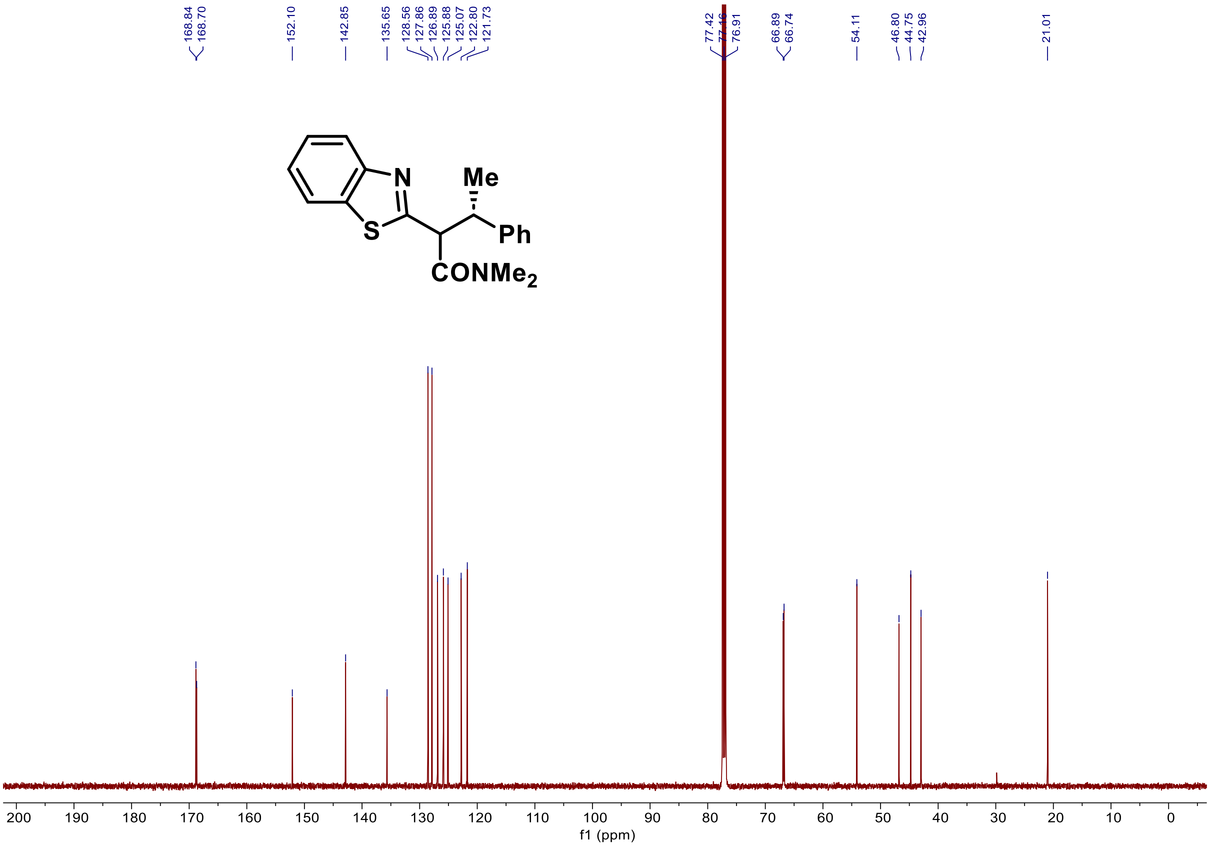

(3*S*)-2-(Benzo[*d*]thiazol-2-yl)-*N*-methoxy-*N*-methyl-3-phenylbutanamide (S3ga’’’’’)

Diastereomer 1

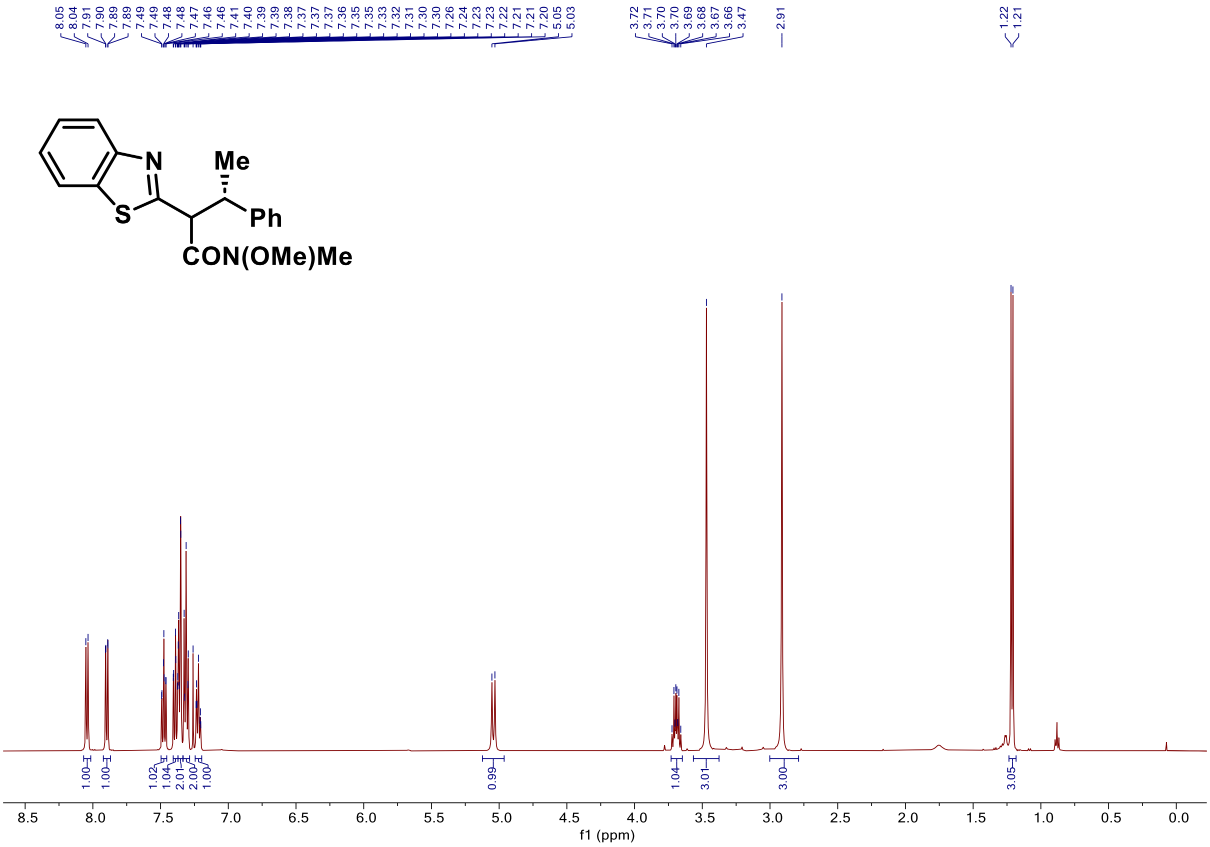

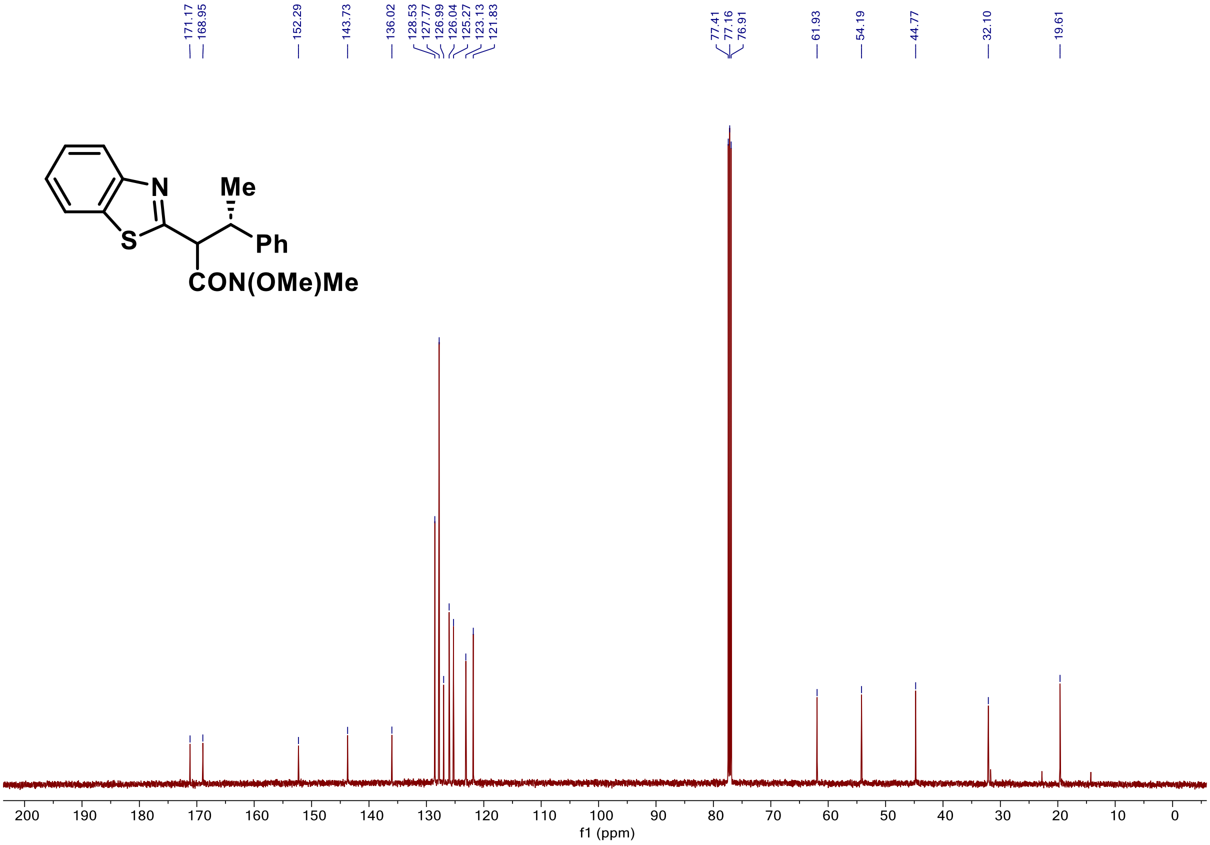

Diastereomer 2

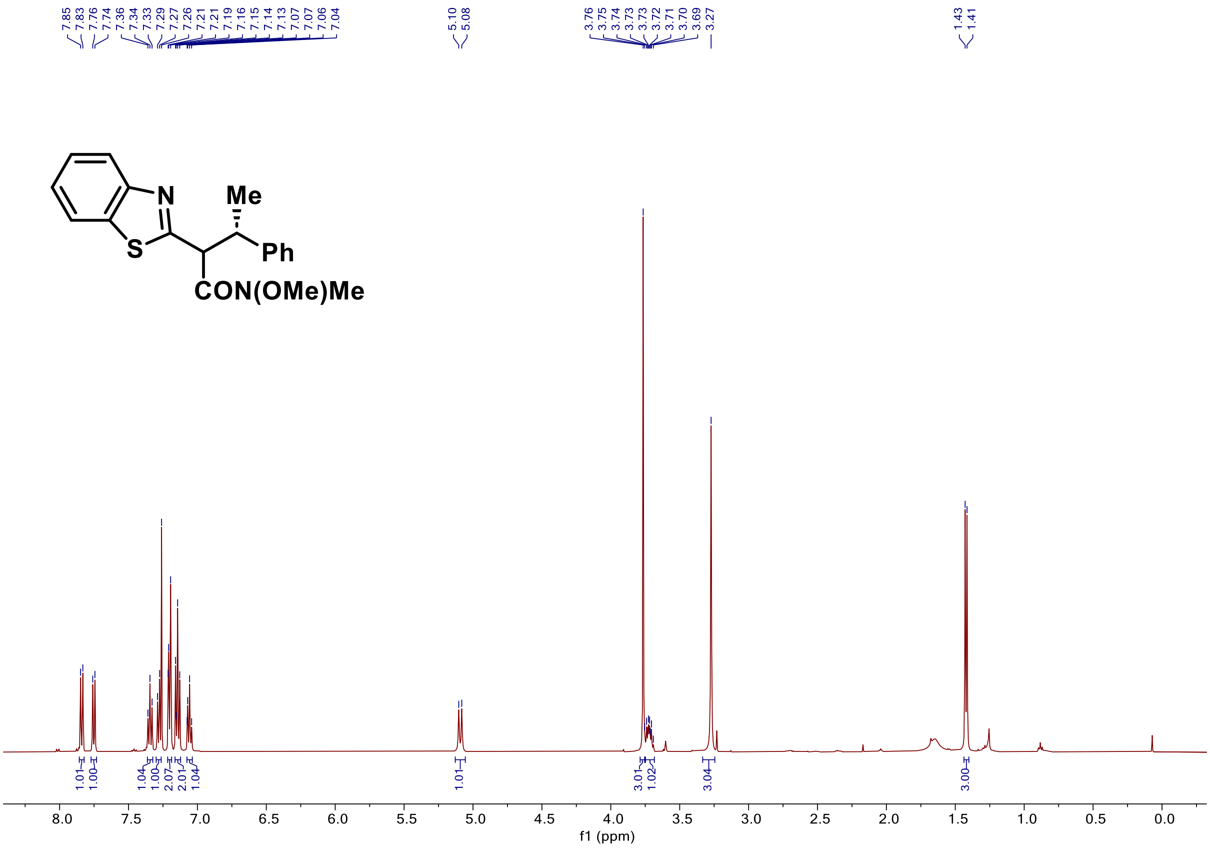

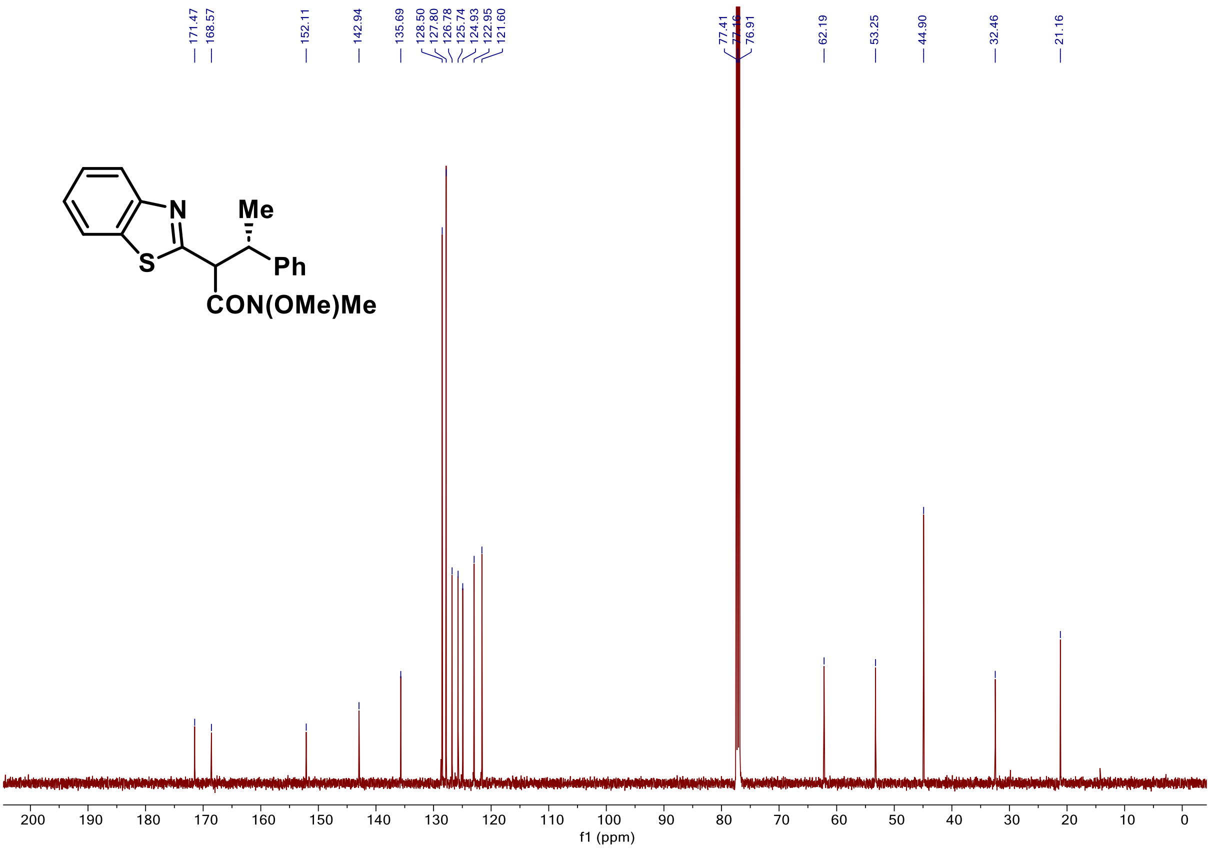

(*R*)-2-(2,5-Dimethylcyclopent-1-en-1-yl)benzo[*d*]thiazole (5)

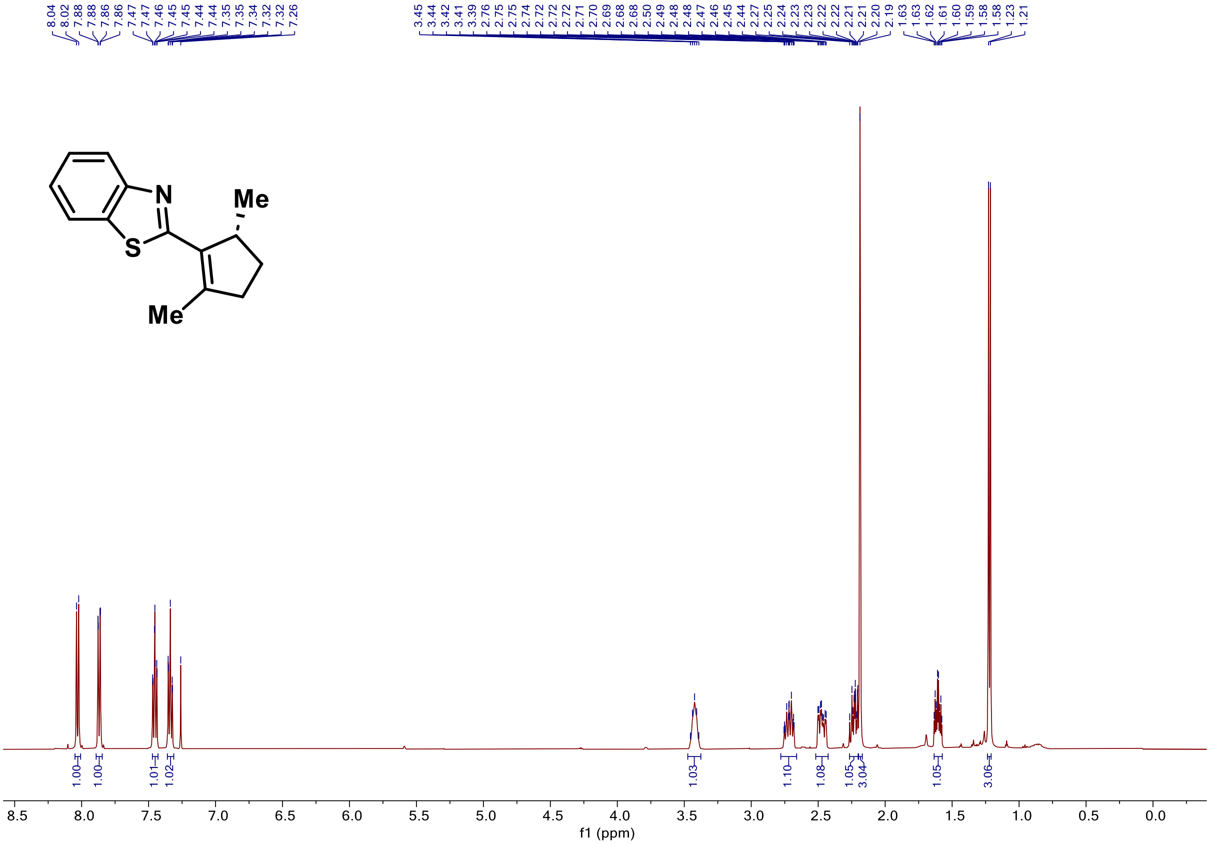

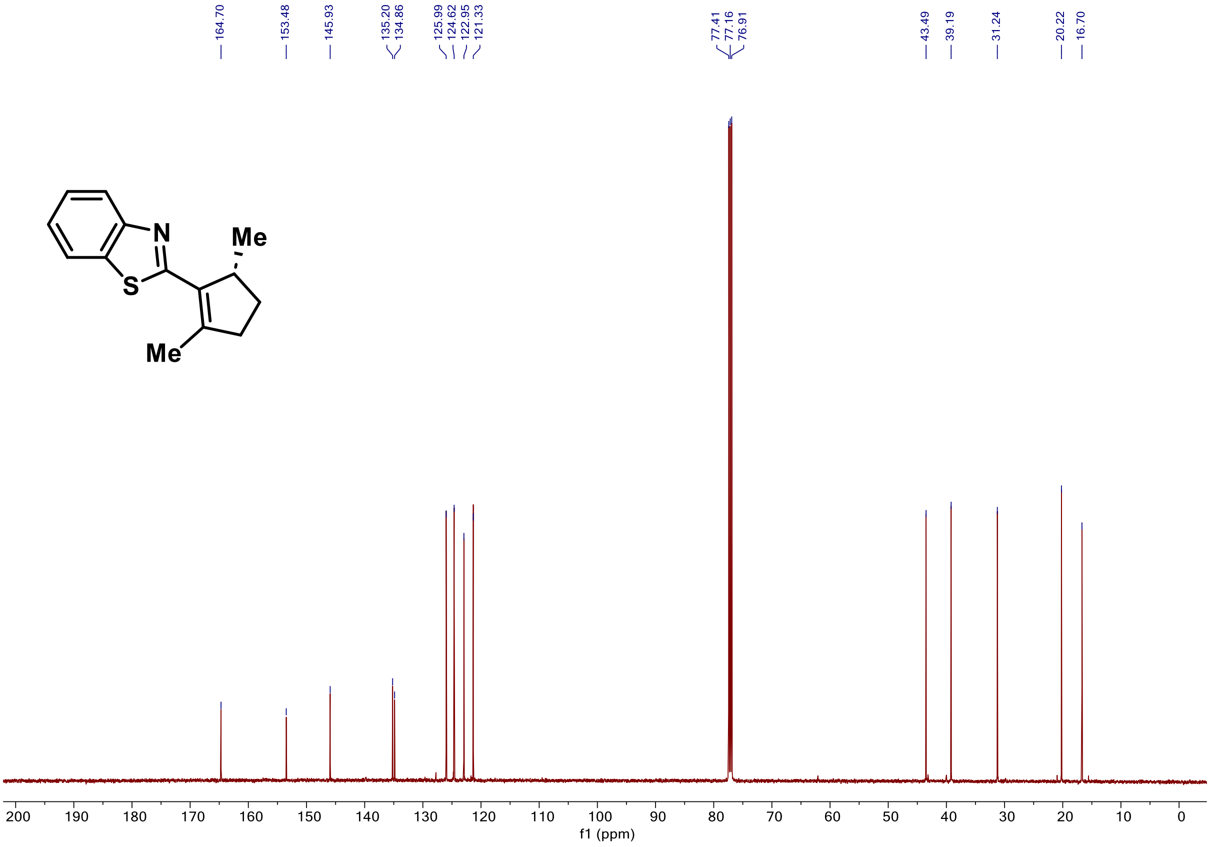

tert-Butyl (3R)-2-(benzo[d]thiazol-2-yl)-2-fluoro-3-phenylbutanoate (6)

Major diastereomer

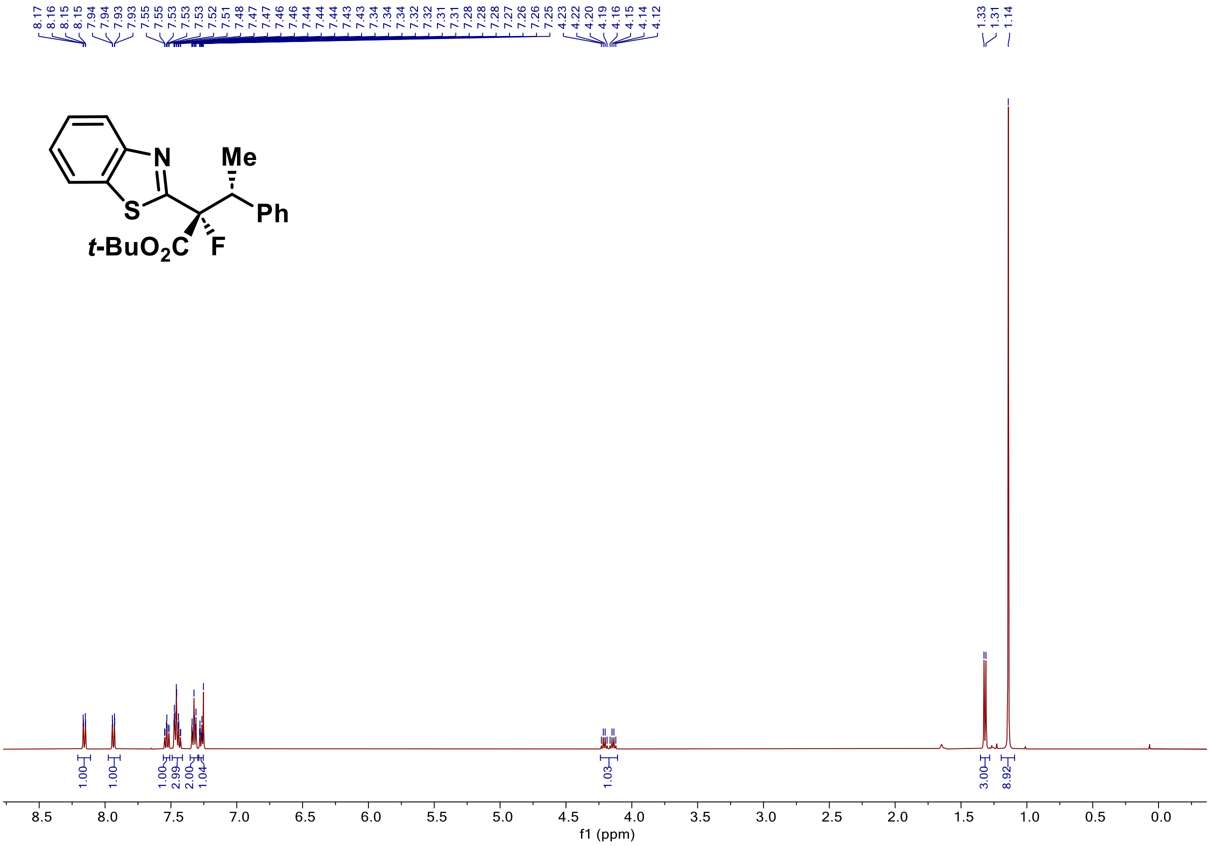

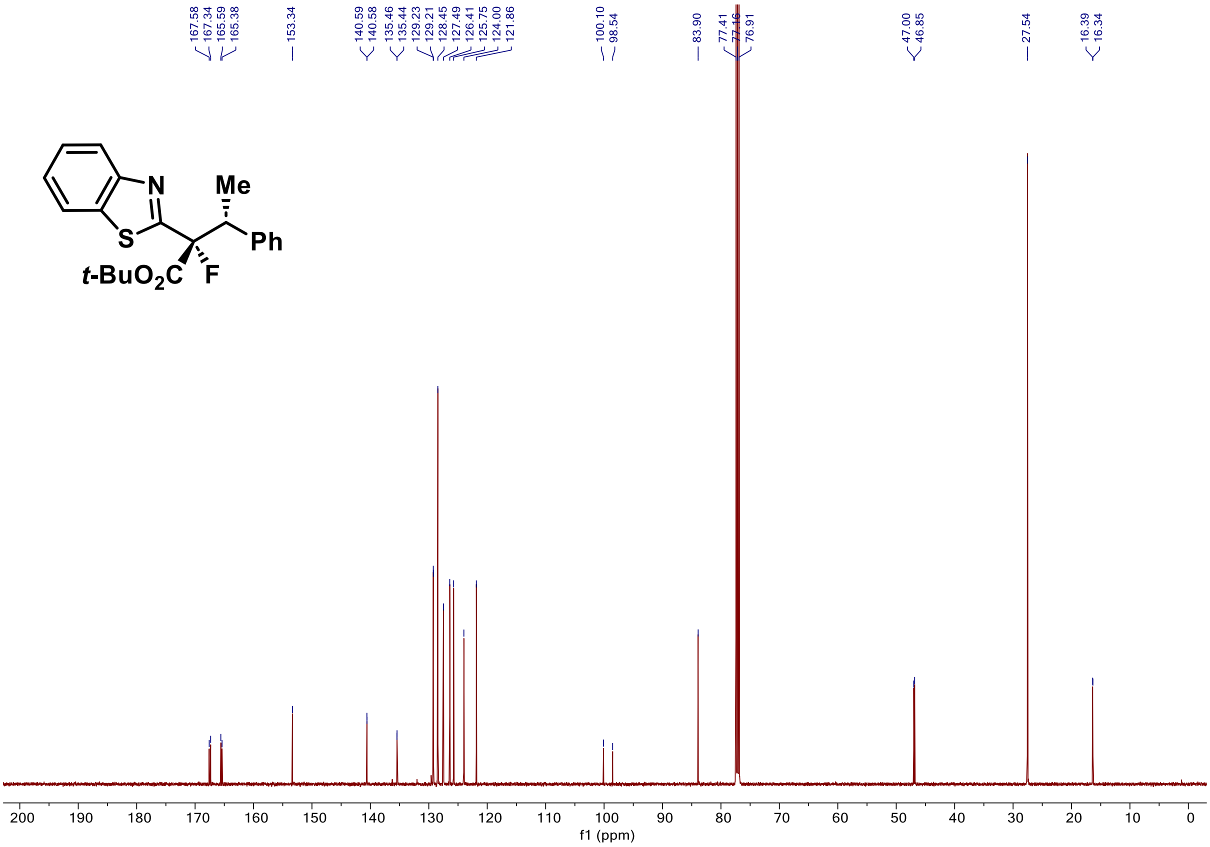

Minor diastereomer

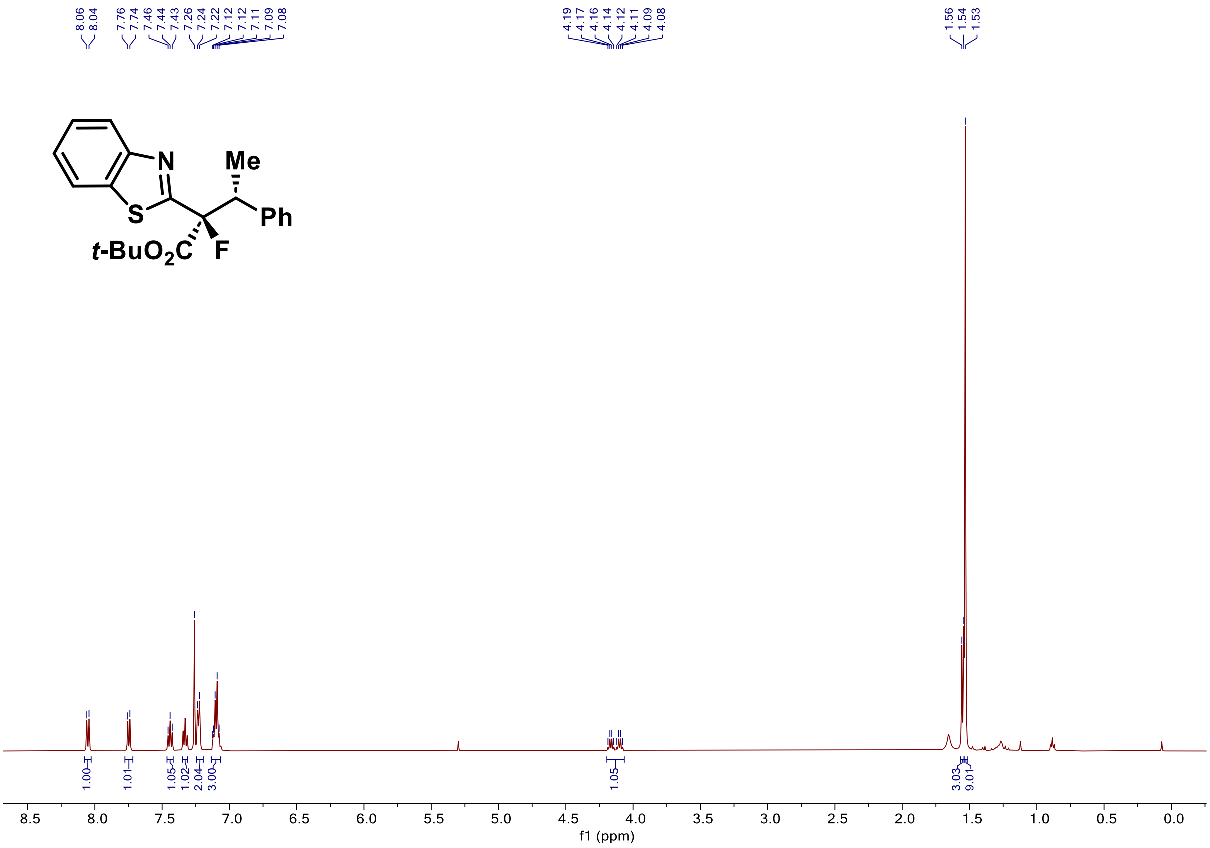

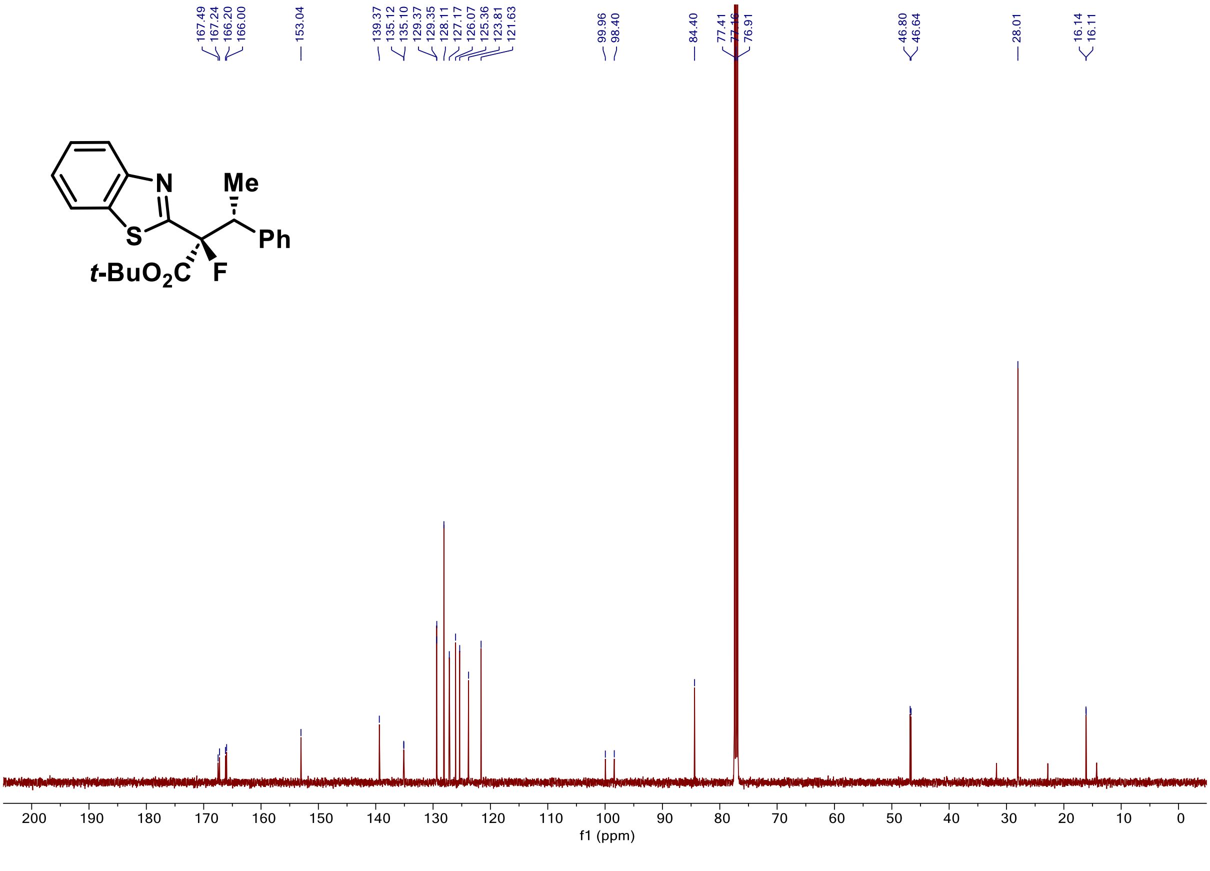

tert-Butyl (3*R*)-2-(benzo[*d*]thiazol-2-yl)-2-methyl-3-phenylbutanoate (7)

Major diastereomer

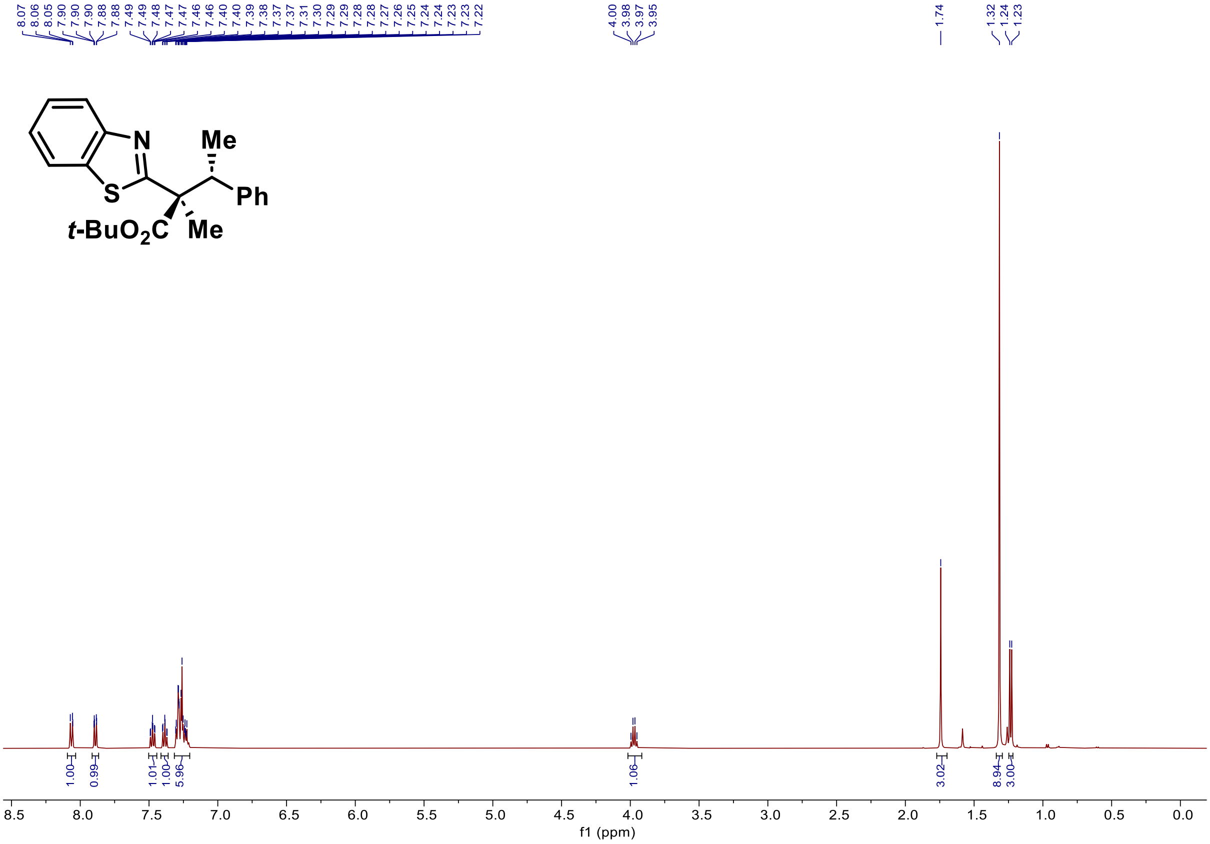

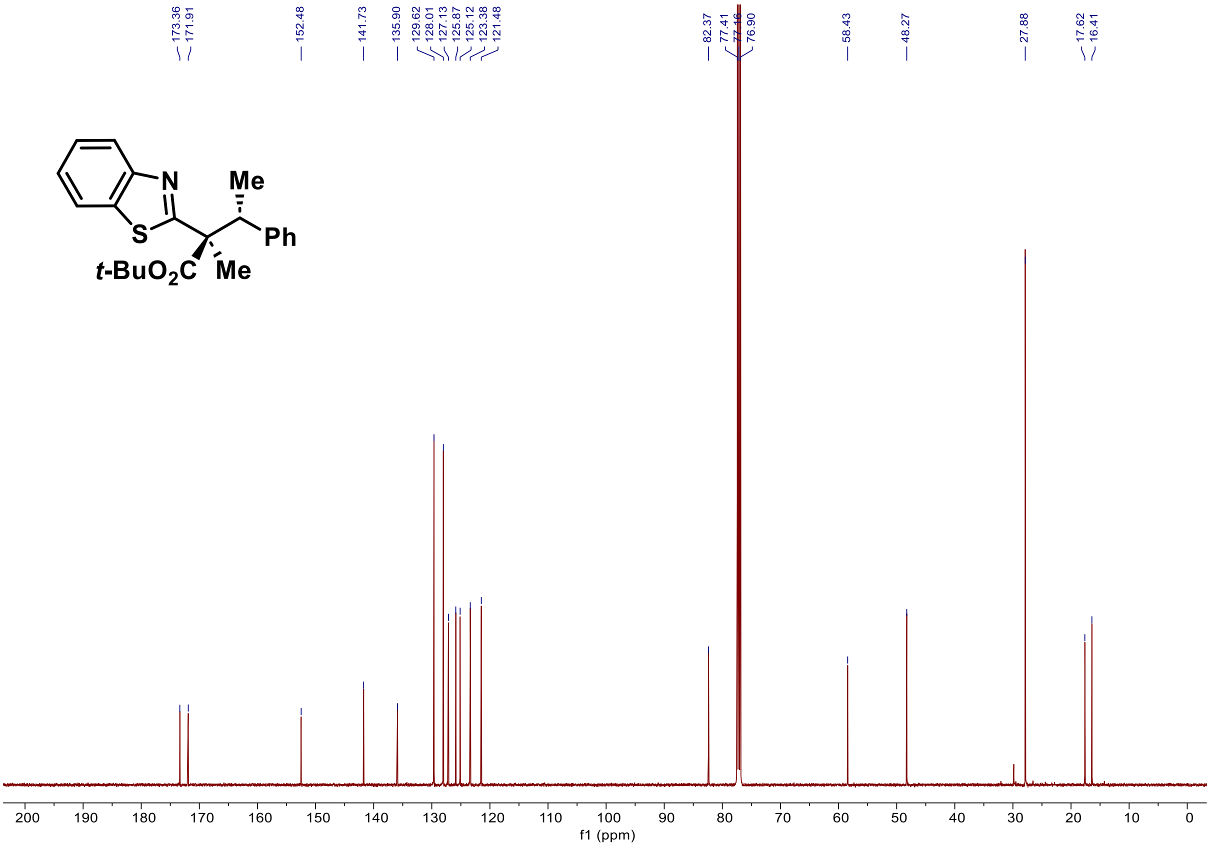

Minor diastereomer

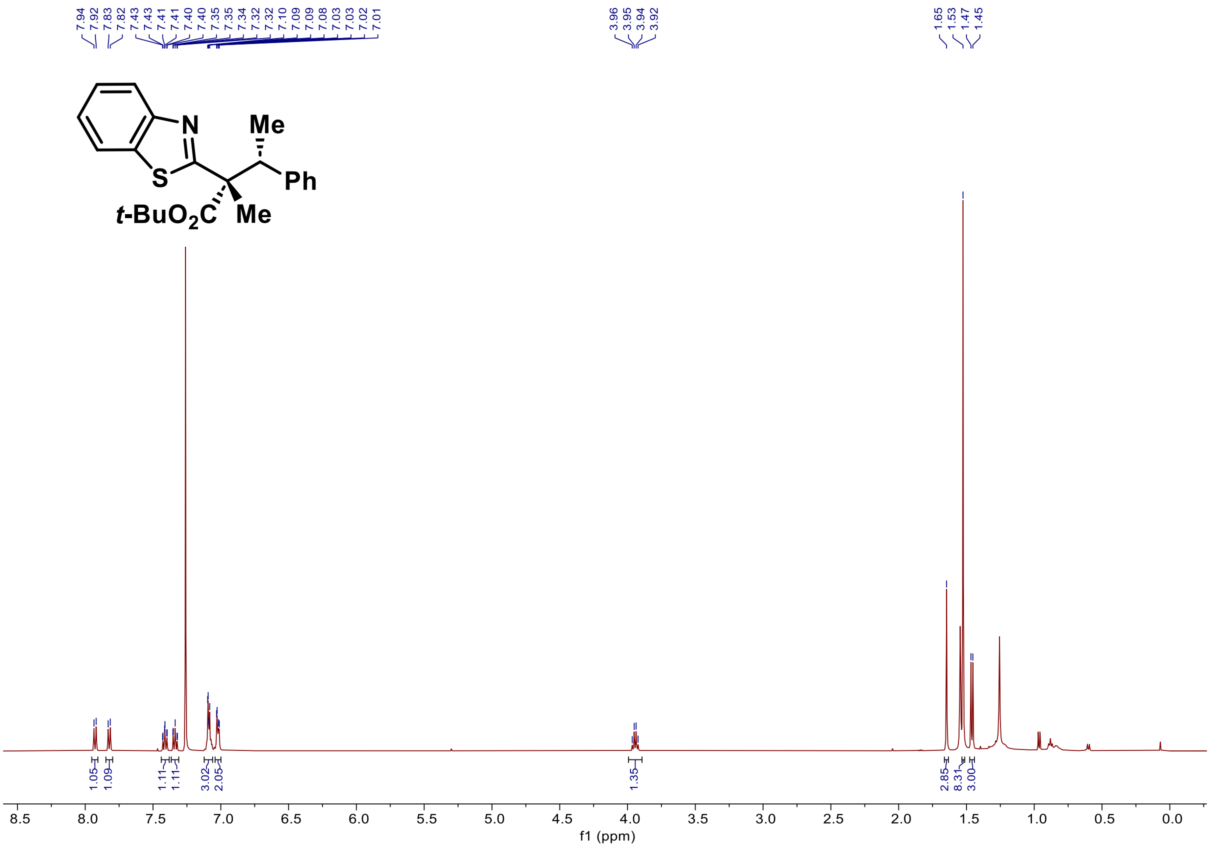

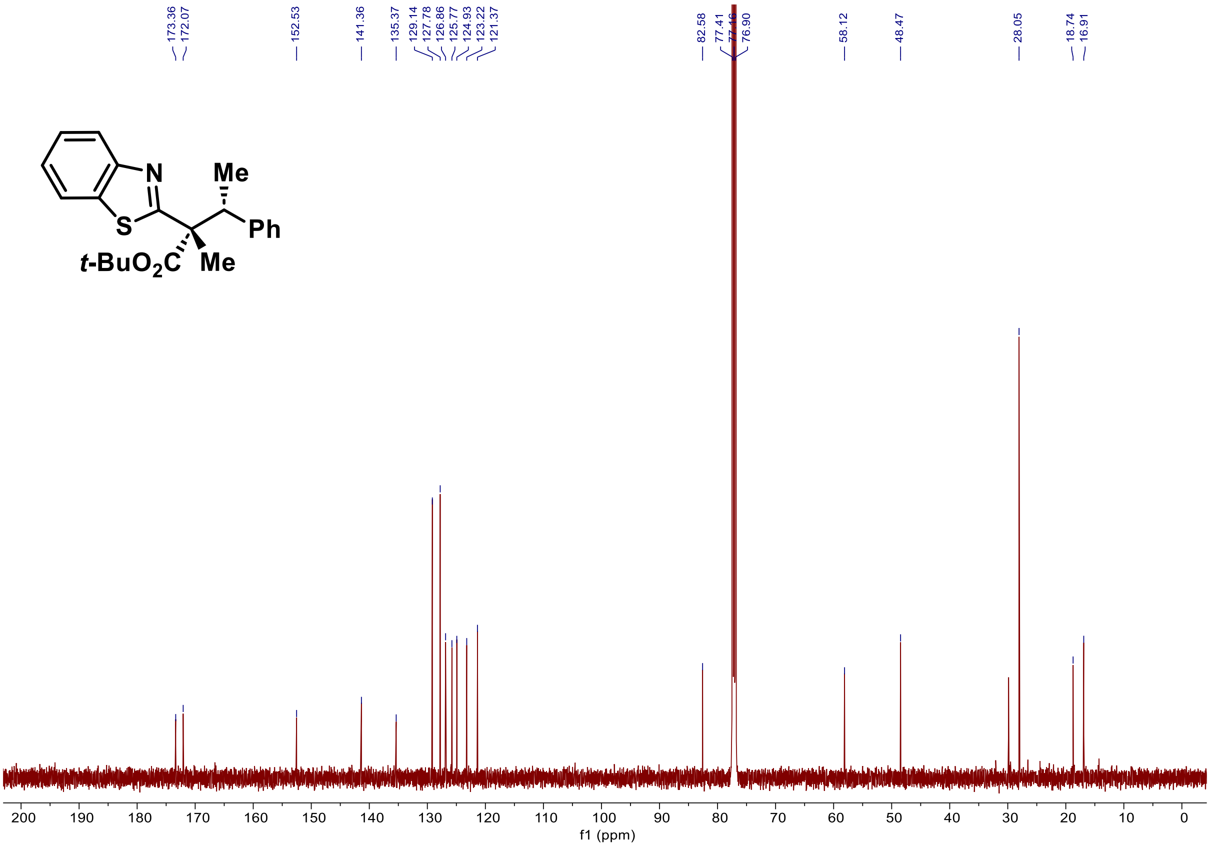

tert-Butyl (3S)-2-(benzo[d]thiazol-2-yl)-3-(2-chlorophenyl)butanoate

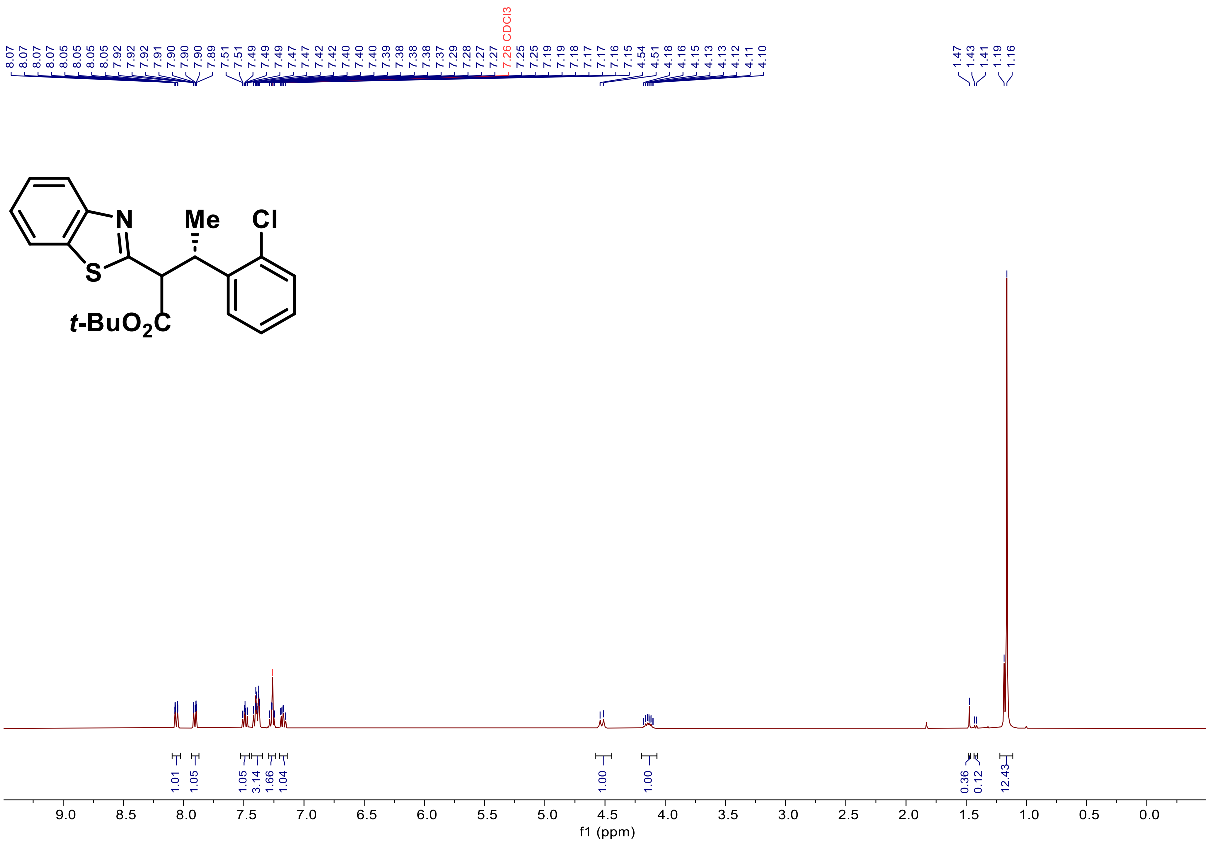

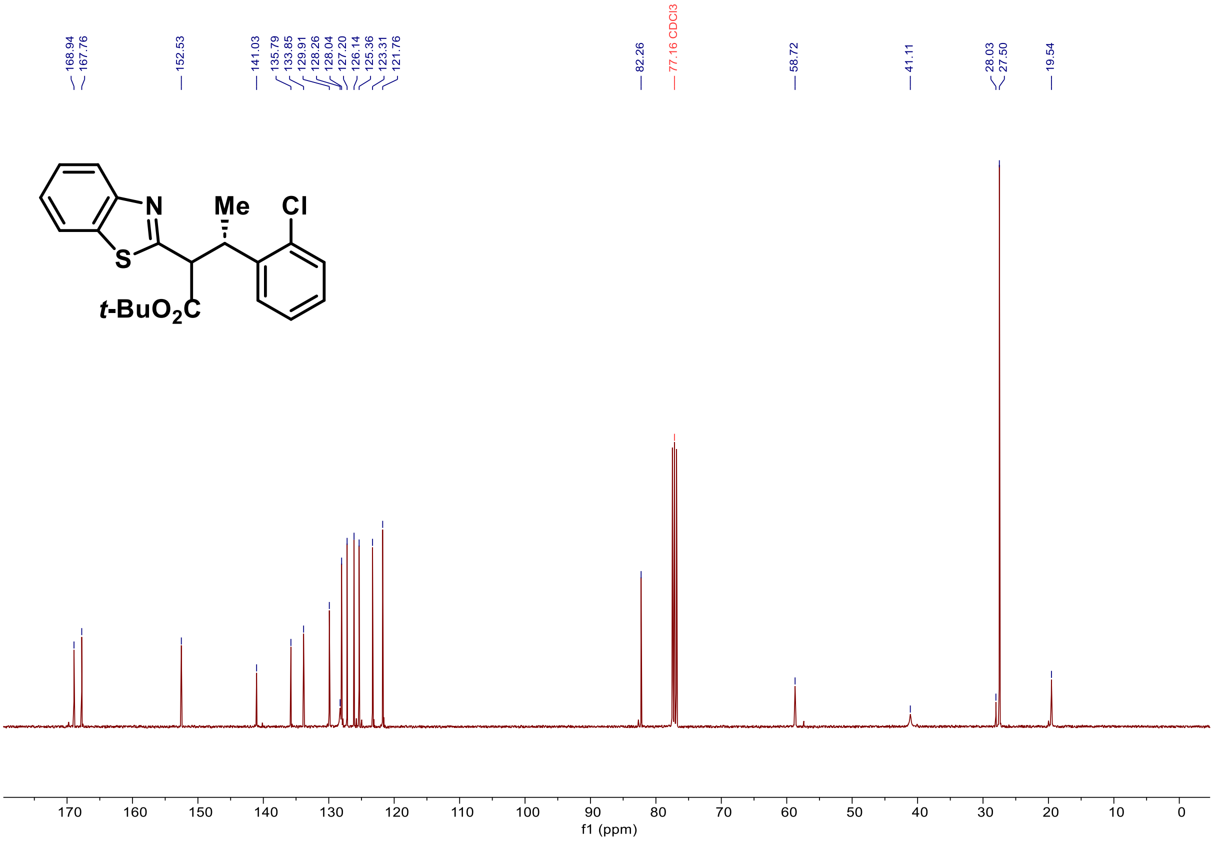

***tert*-Butyl (2*S*,3*S*)-2-(benzo[*d*]thiazol-2-yl)-3-(2-chlorophenyl)-2-methylbutanoate**

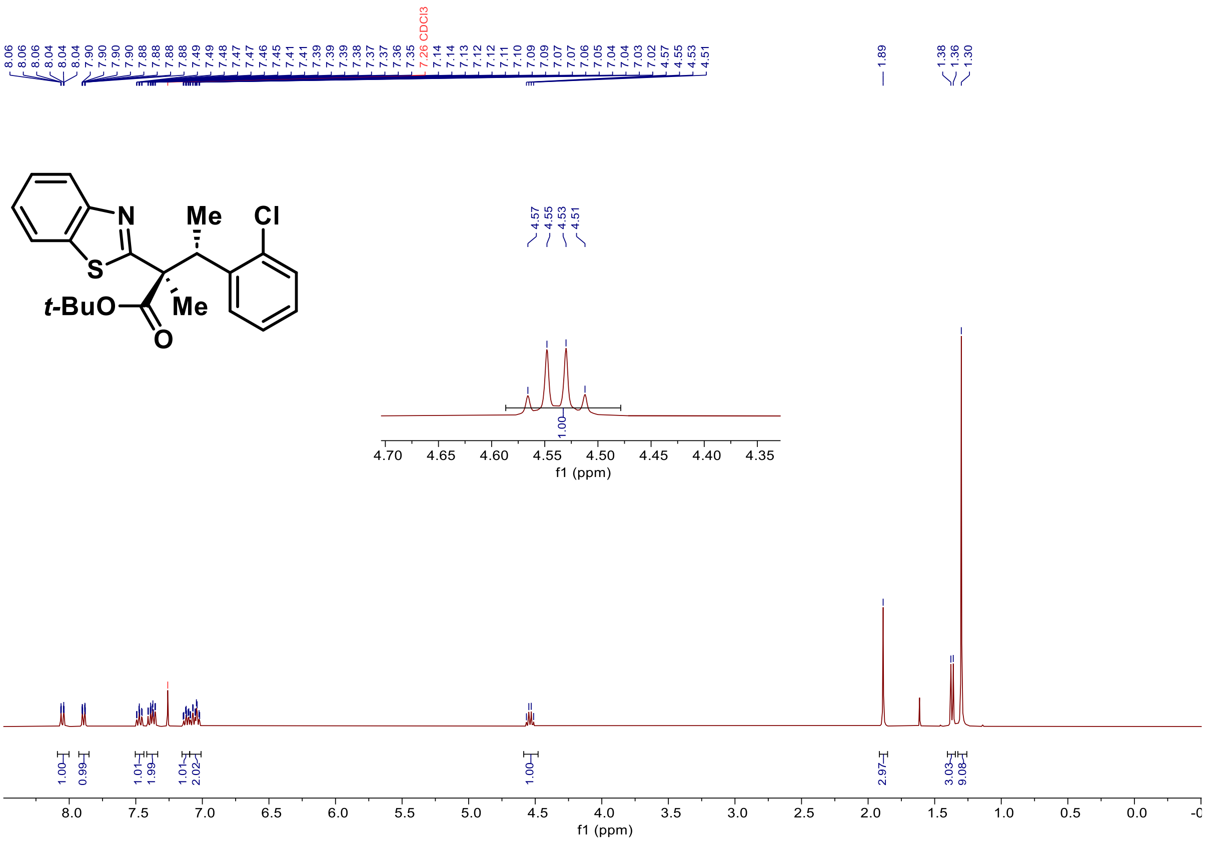

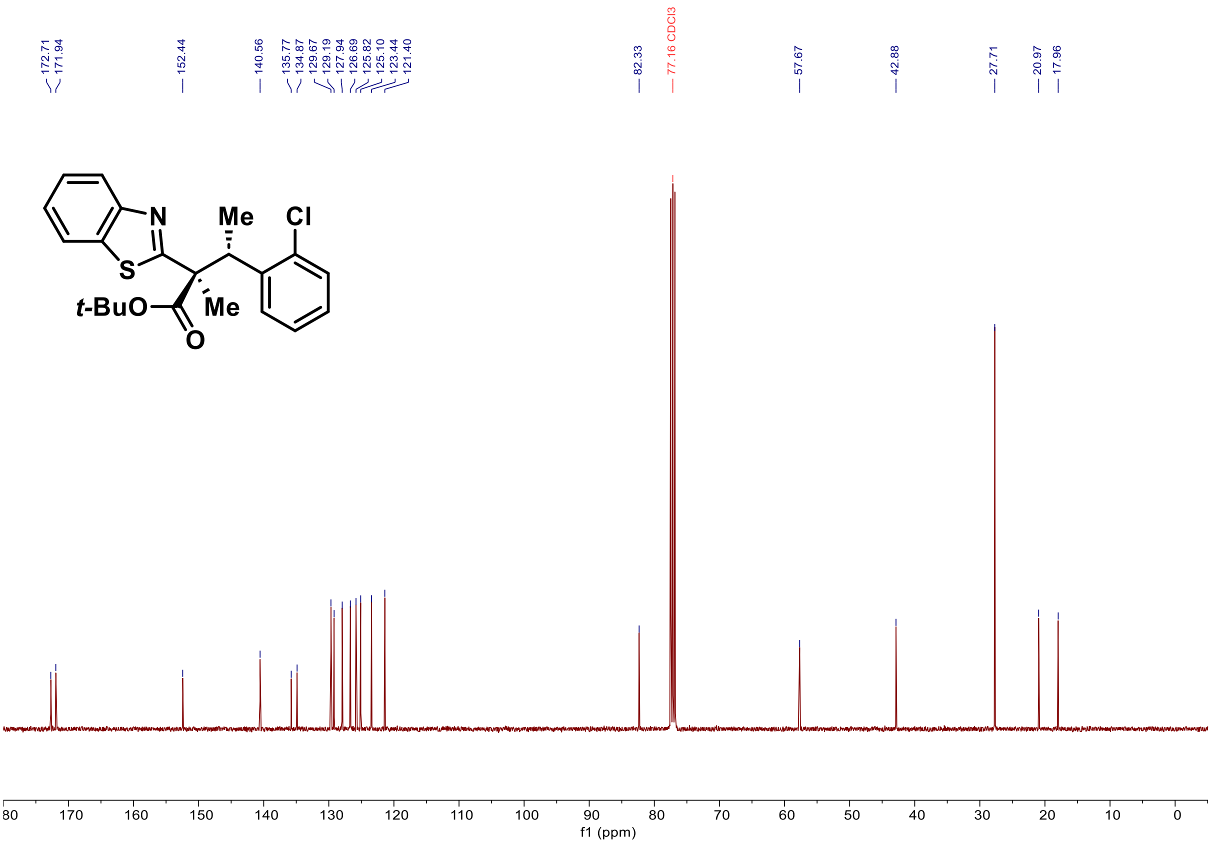

(2*S*,3*S*)-2-(Benzo[*d*]thiazol-2-yl)-3-(2-chlorophenyl)-2-methylbutan-1-ol

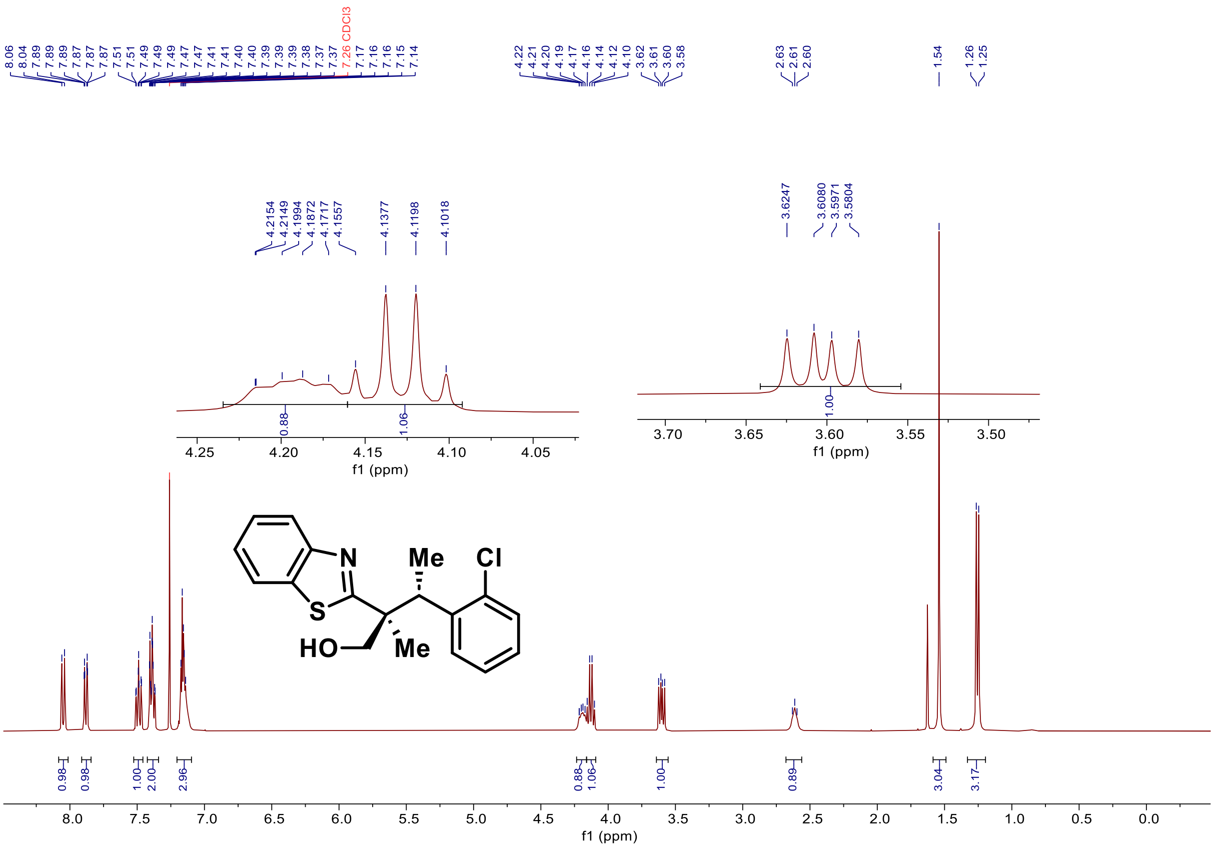

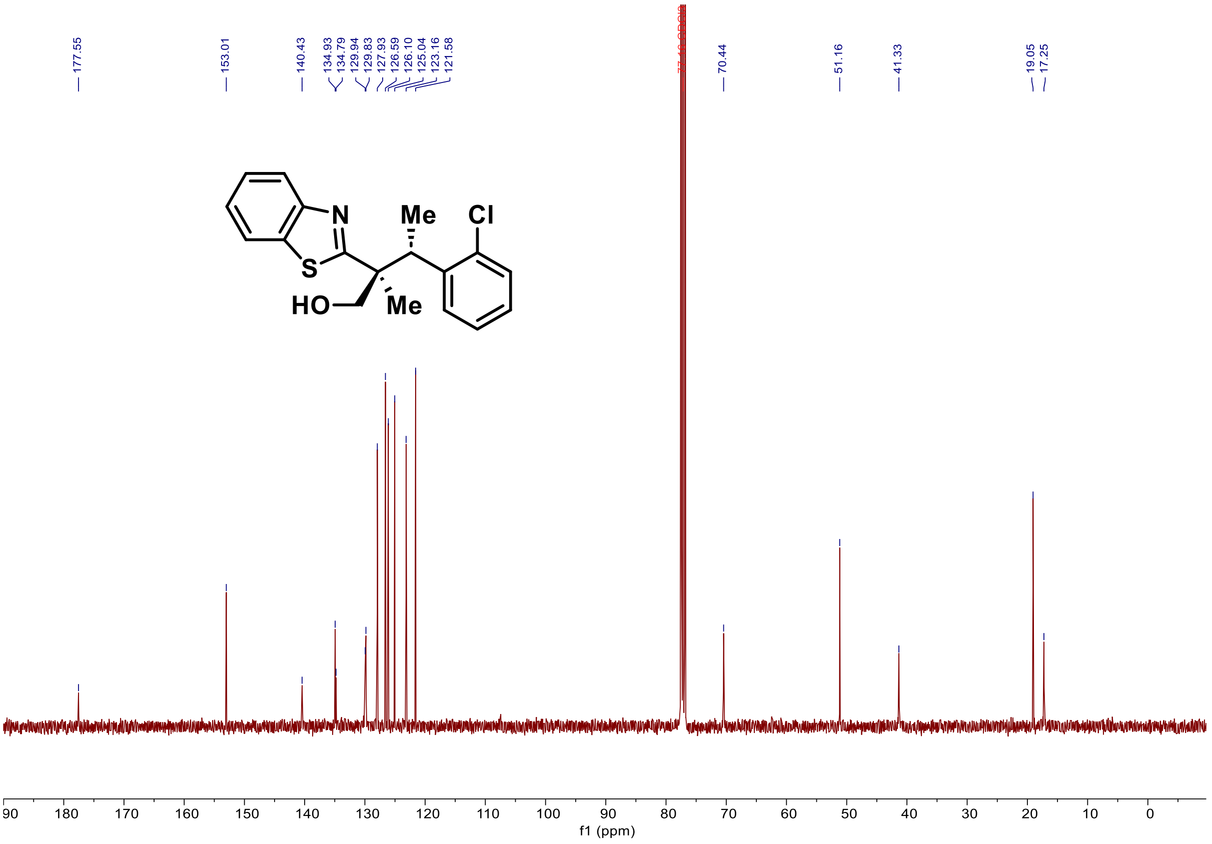

2-((3*S*,4*R*)-3,4-Dimethylchroman-3-yl)benzo[*d*]thiazole

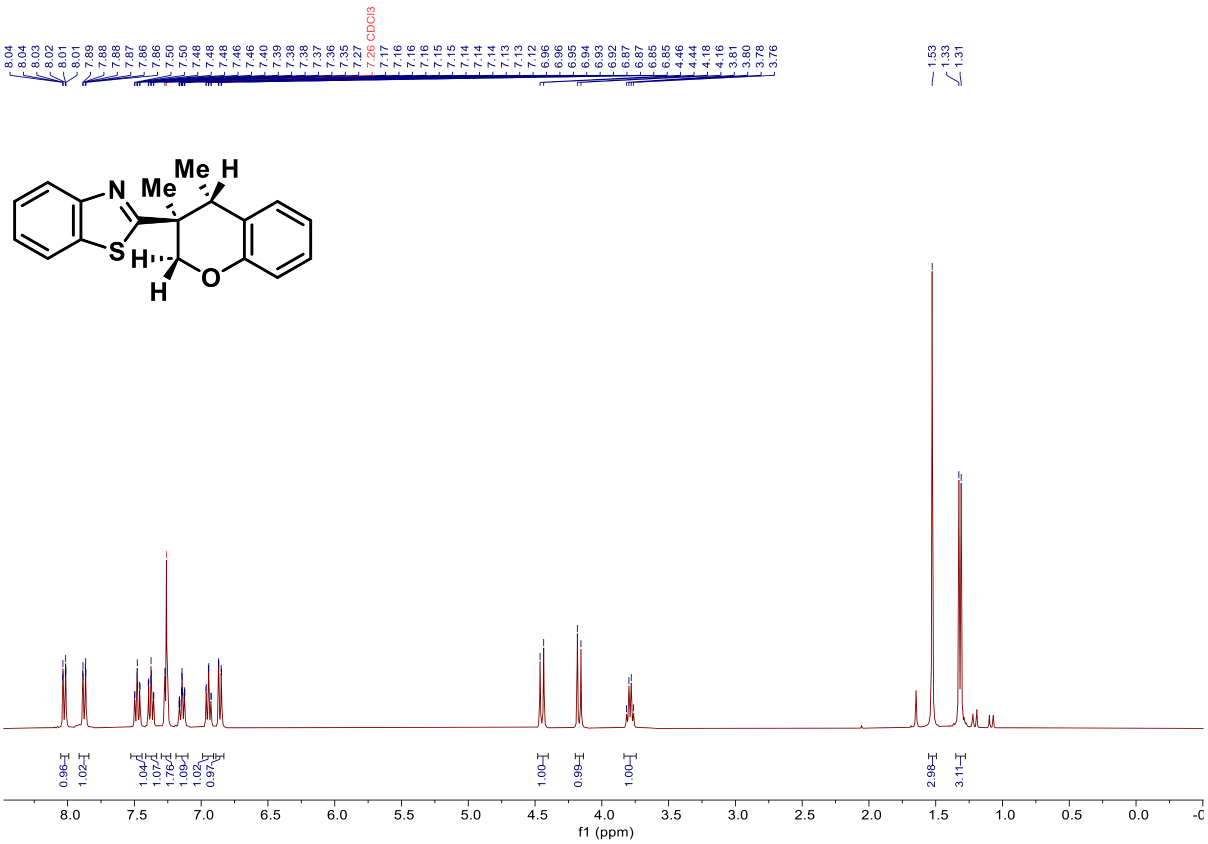

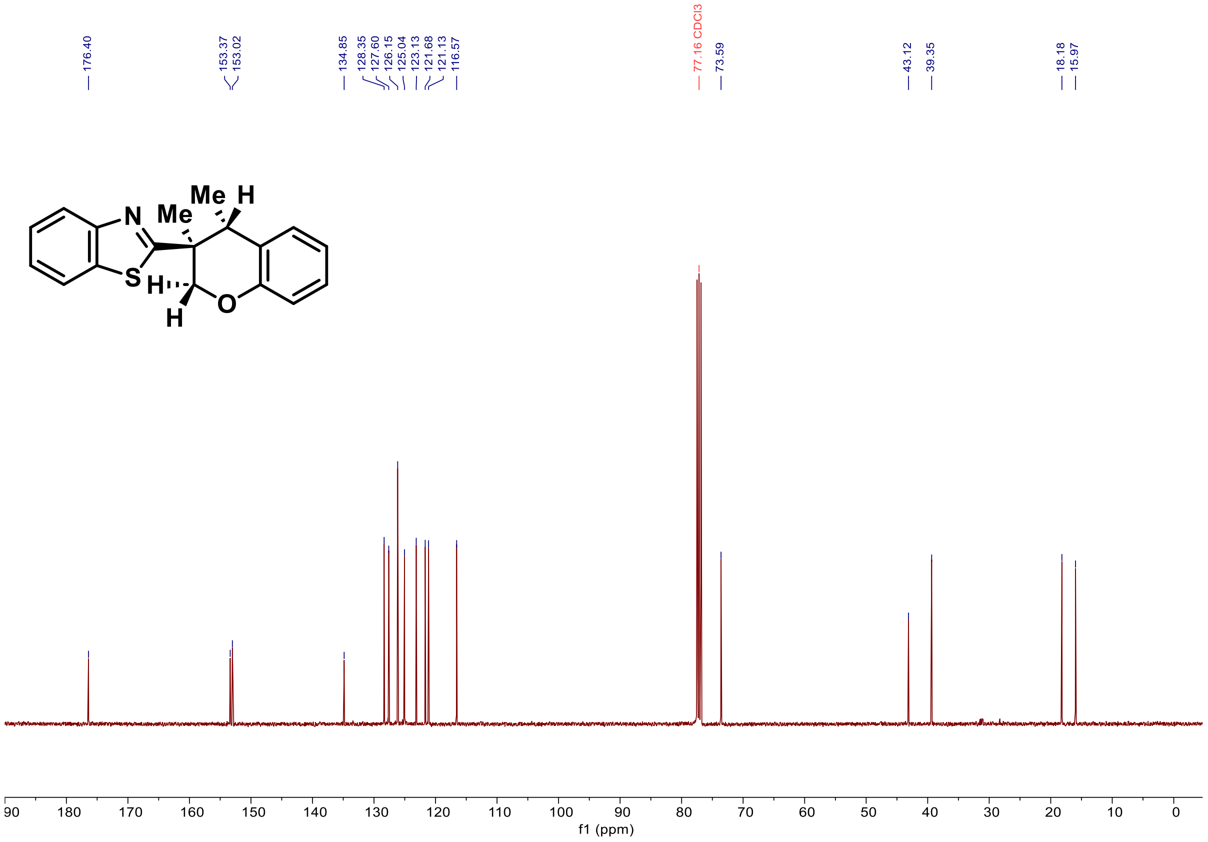

Ethyl 3-phenyl-2-(pyridin-2-yl)butanoate (3aa’’)

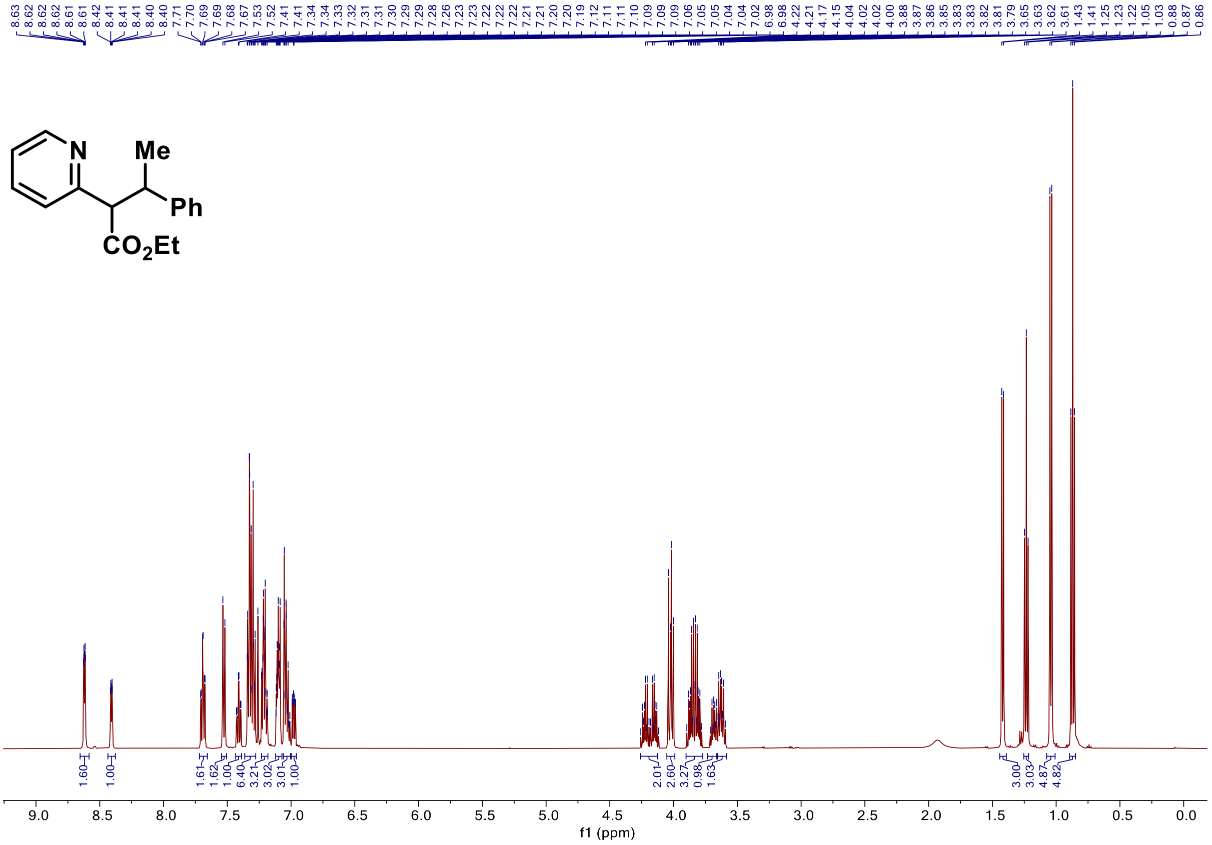

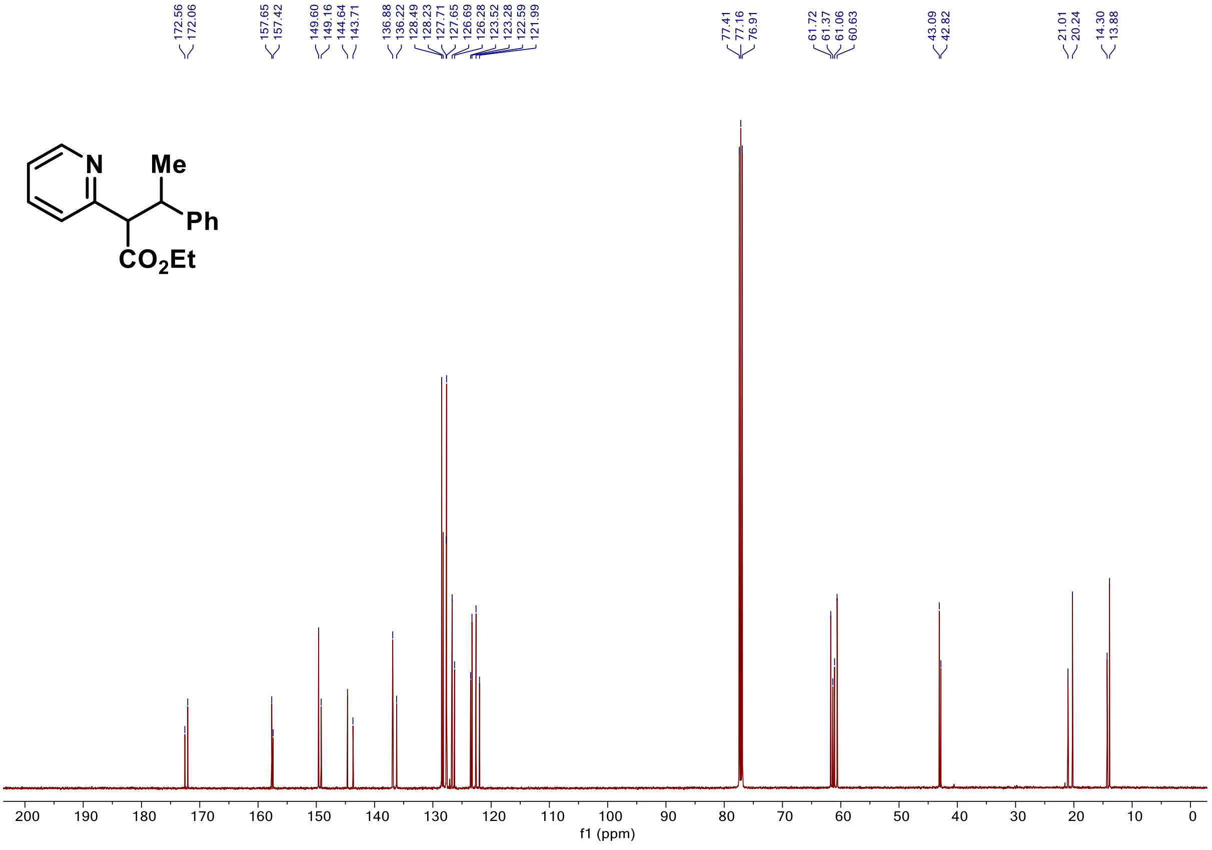

tert-Butyl 3-([1,1'-biphenyl]-4-yl)-2-(benzo[*d*]thiazol-2-yl)butanoate (3gc)

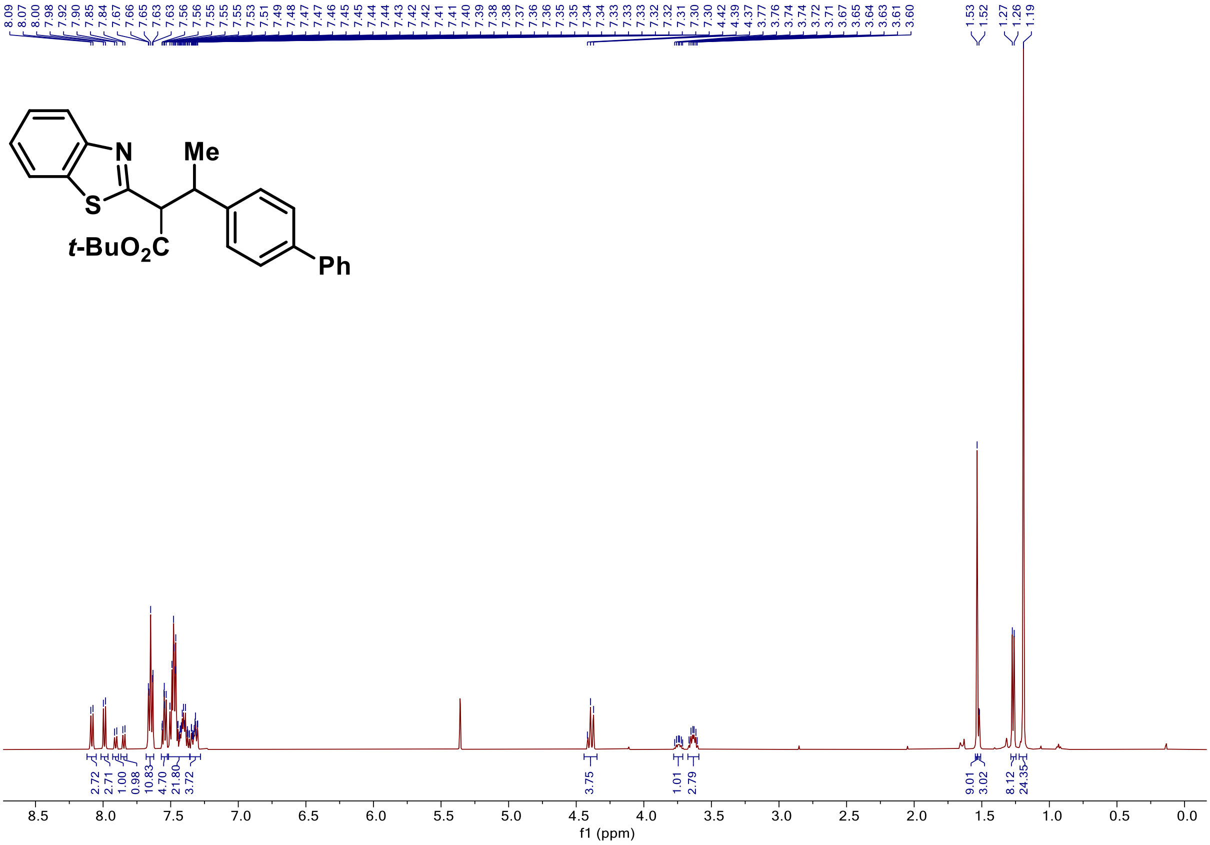

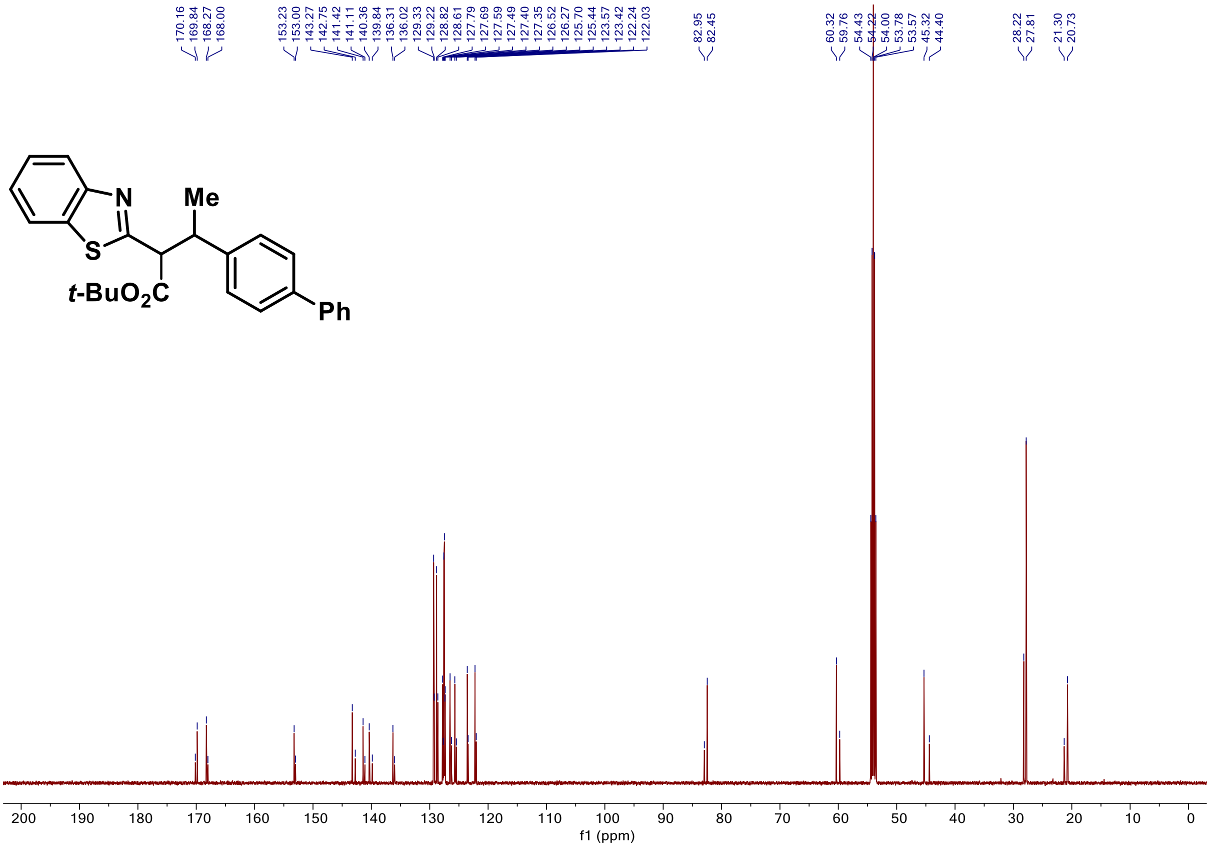

1,3-Diphenylbutan-1-one

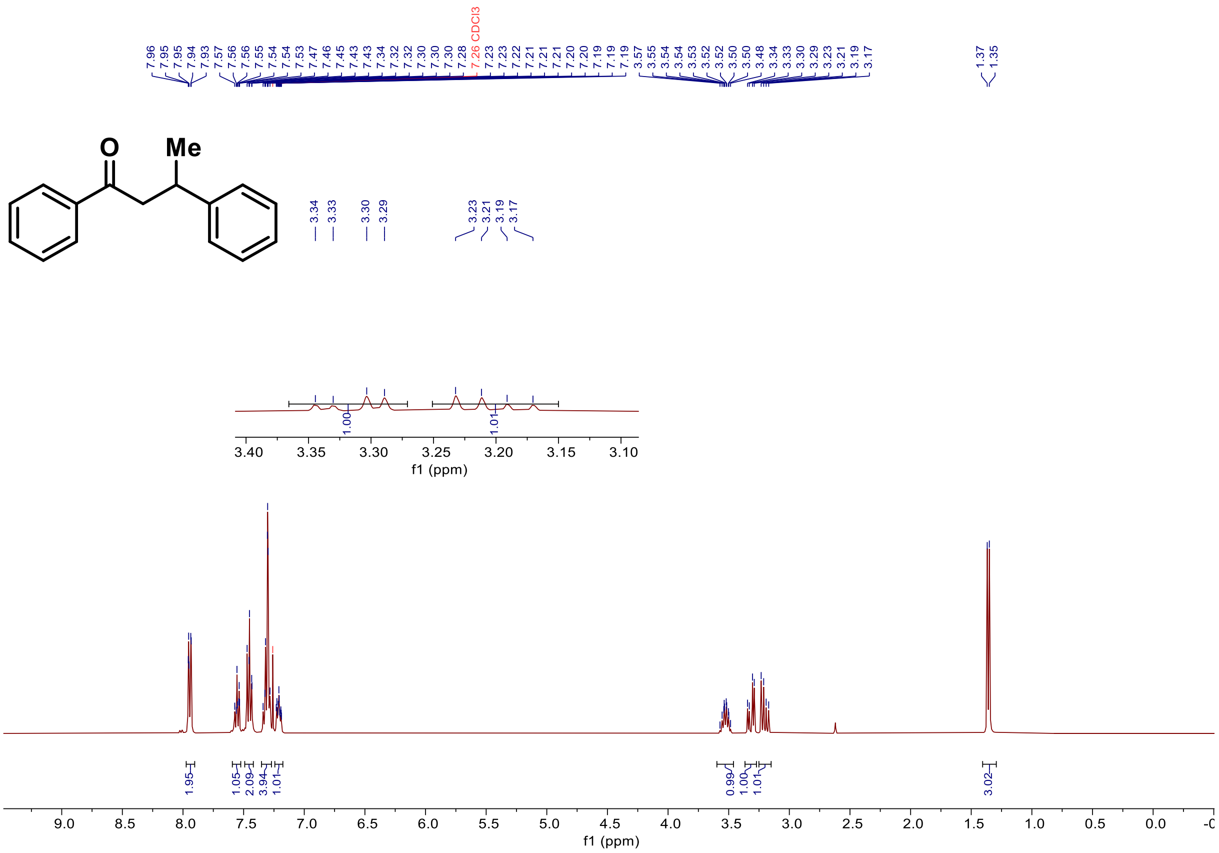

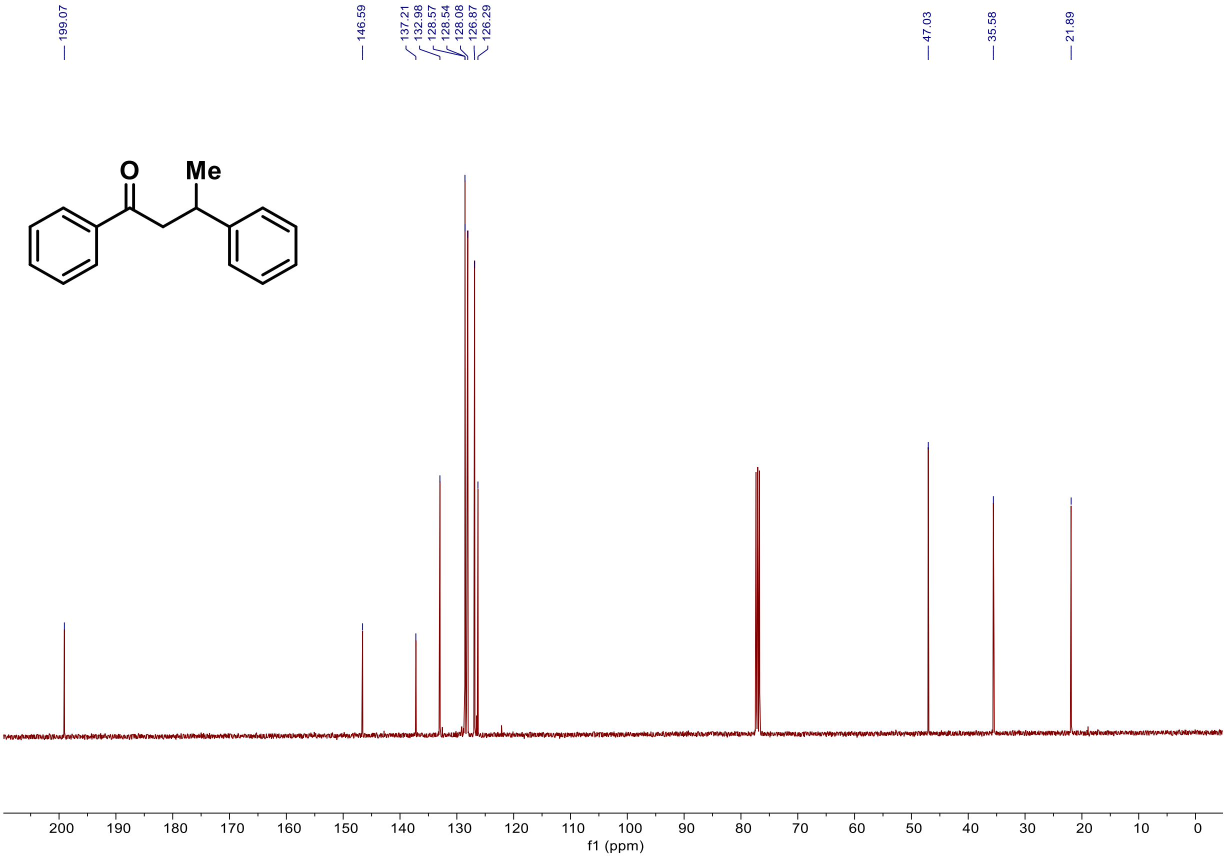

2,6-di-*tert*-Butyl-4-methylphenyl 2-(pyridin-2-yl)acetate (1a''')

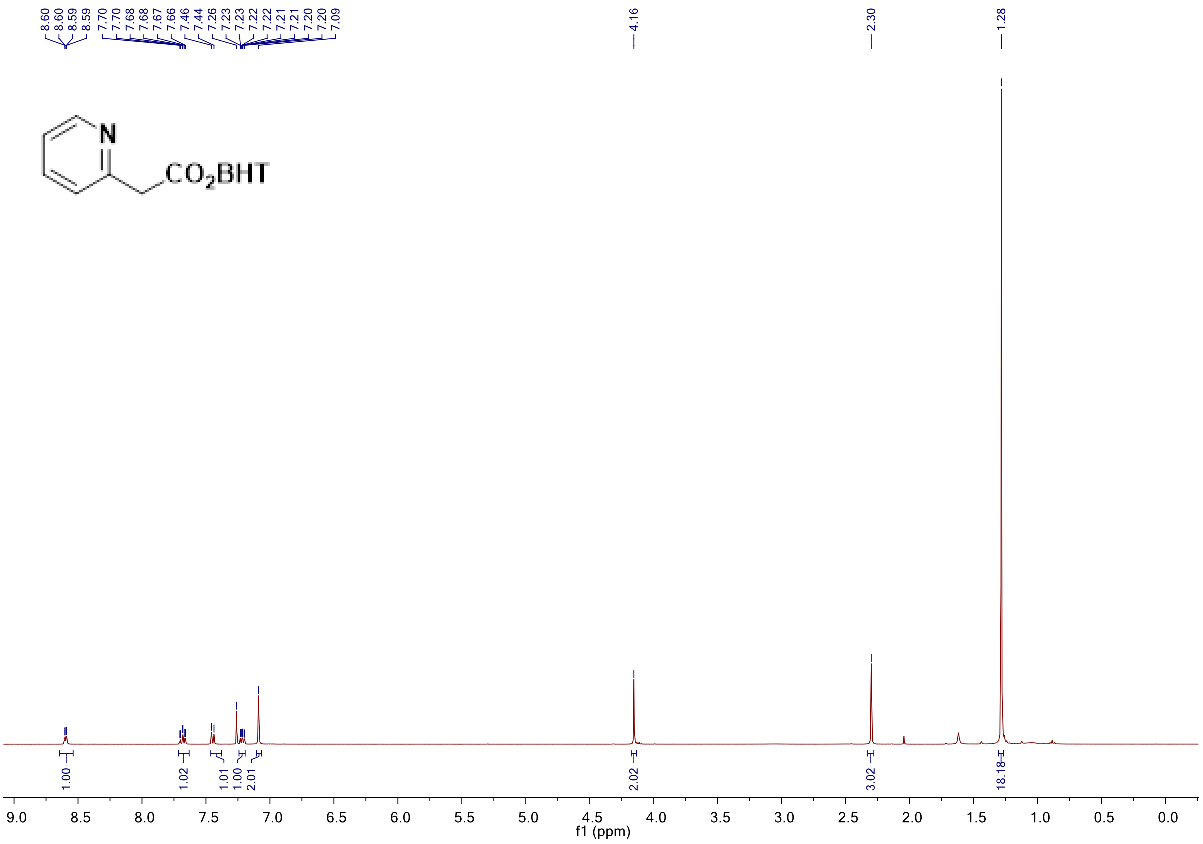

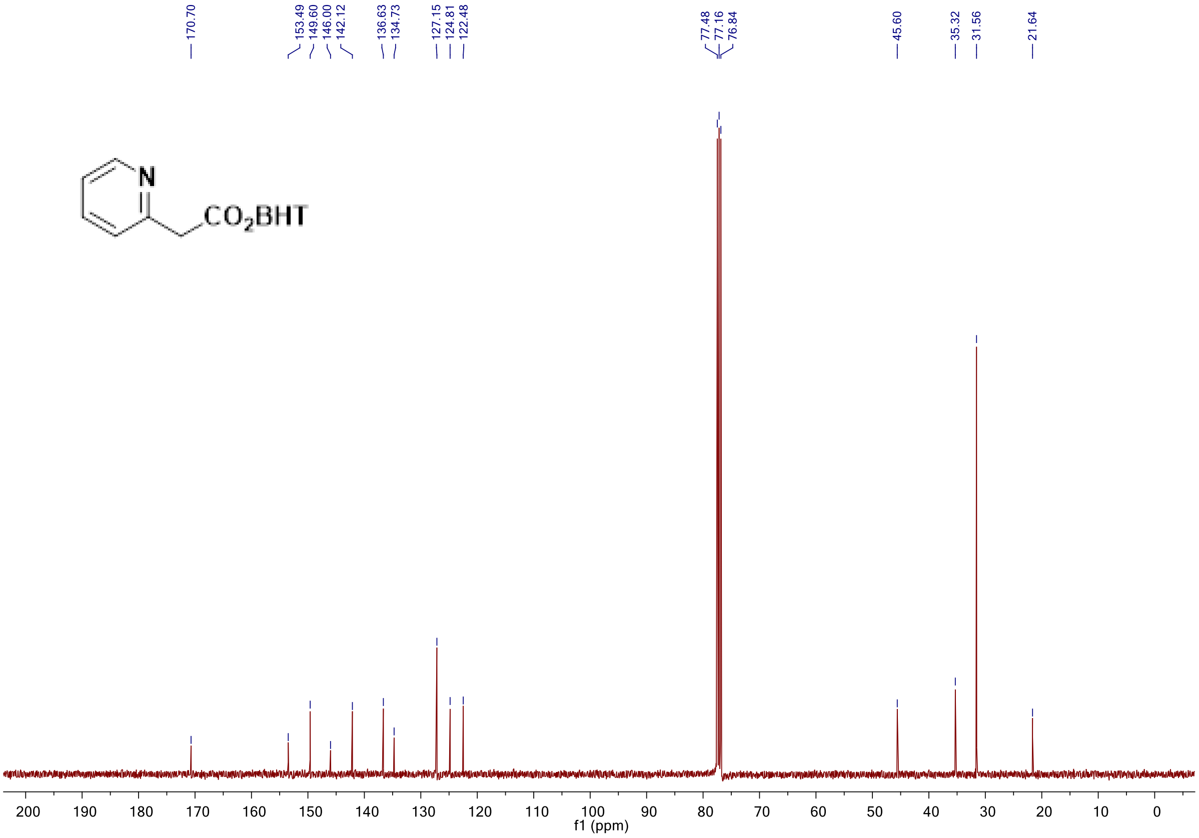

2,6-di-*tert*-Butyl-4-methylphenyl 3-phenyl-2-(pyridin-2-yl)butanoate (3aa''')

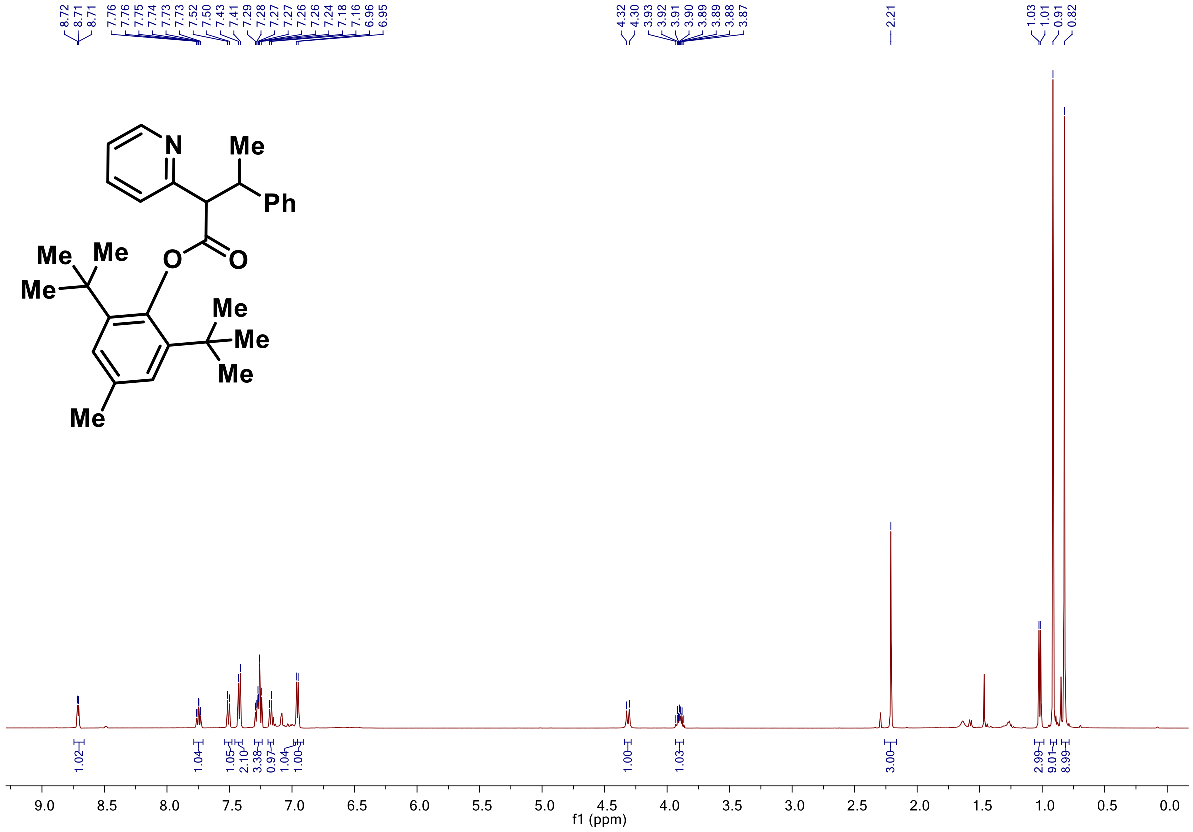

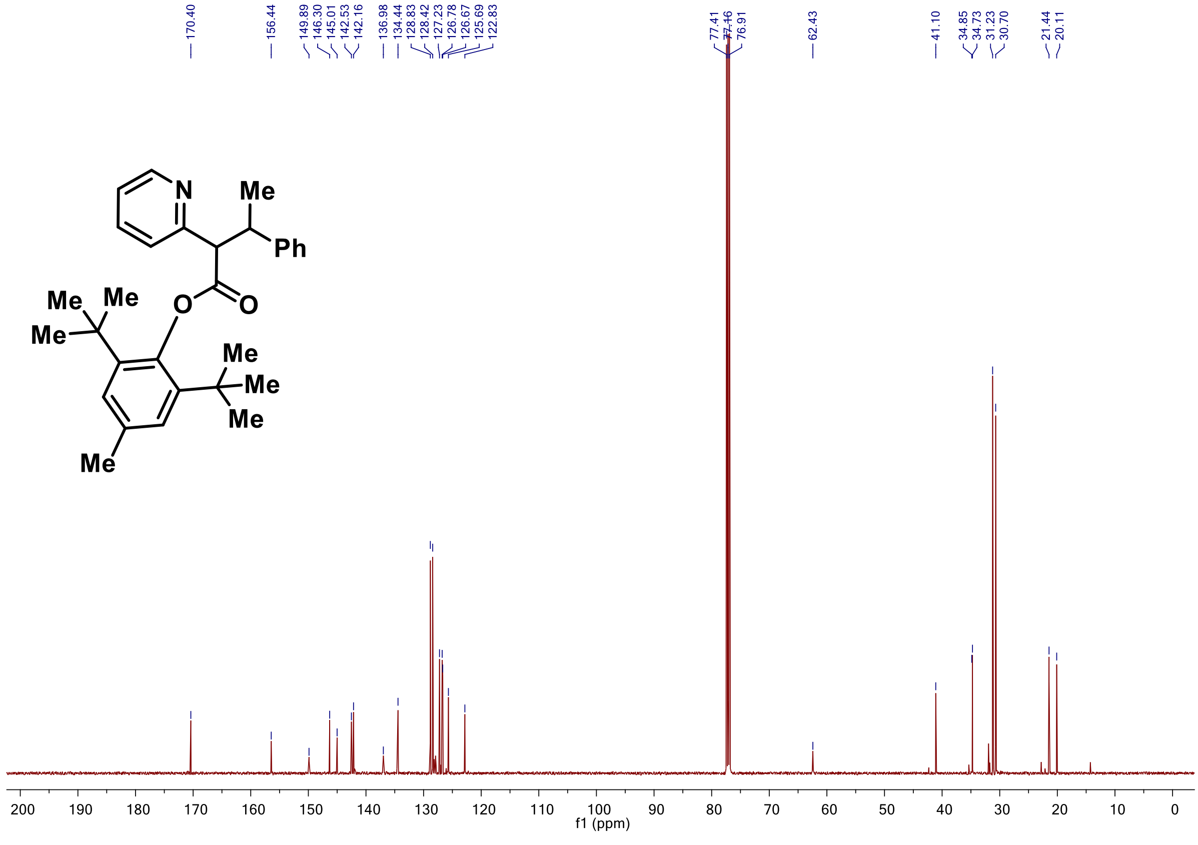

***tert*-Butyl 2-(4,6-dimethoxypyrimidin-2-yl)acetate (1t)**

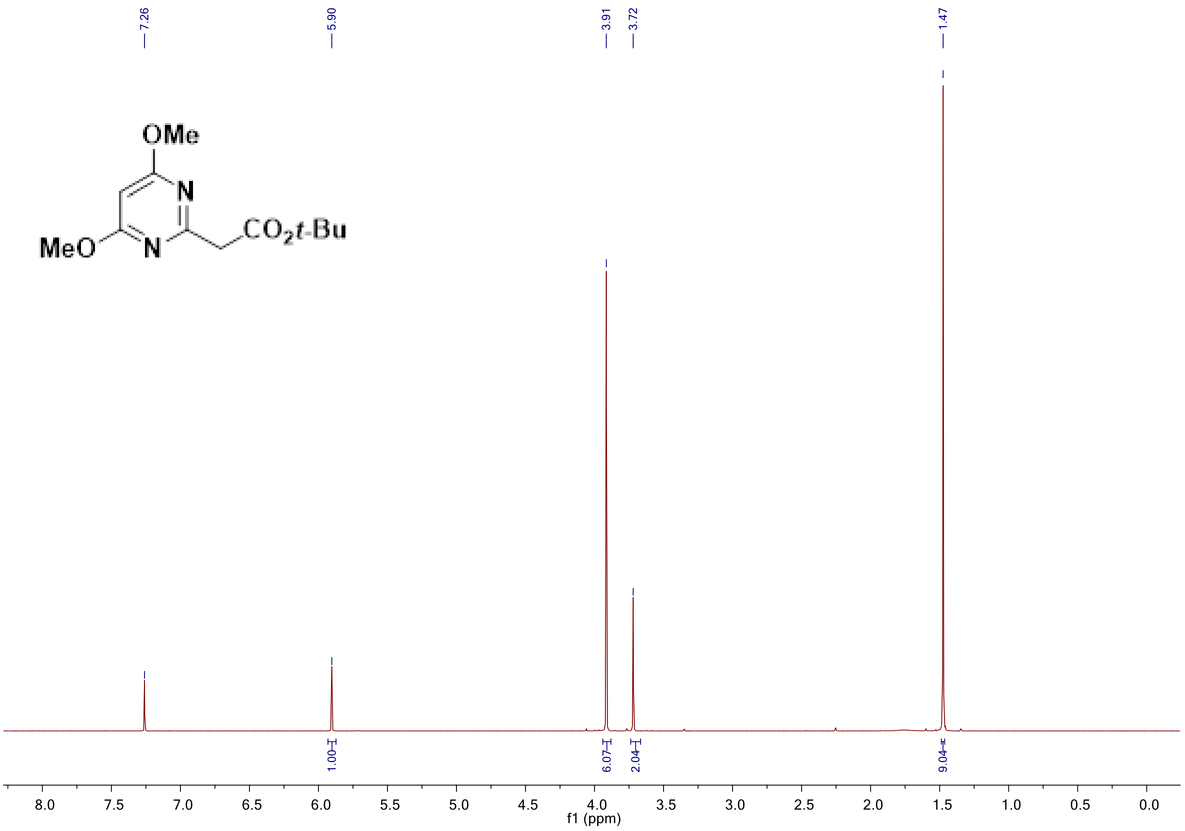

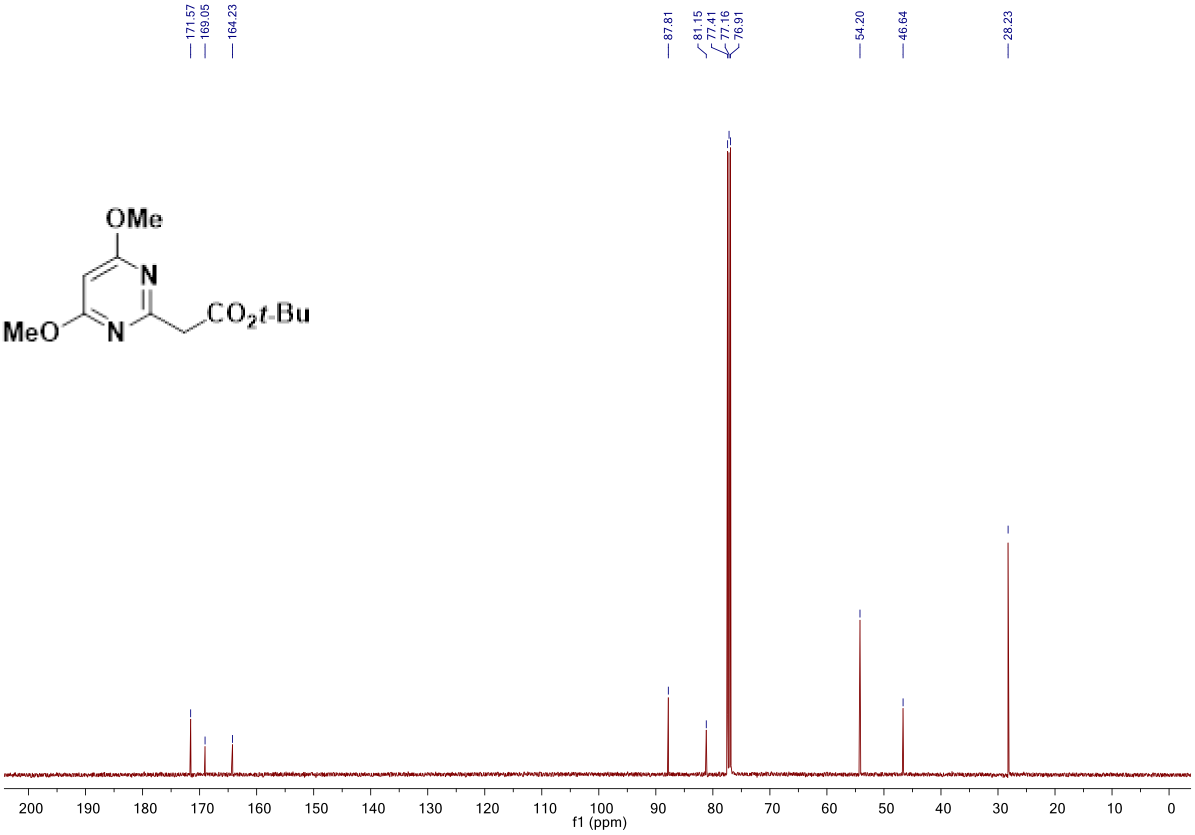

***tert*-Butyl 2-(3-chloroquinoxalin-2-yl)acetate (1u)**

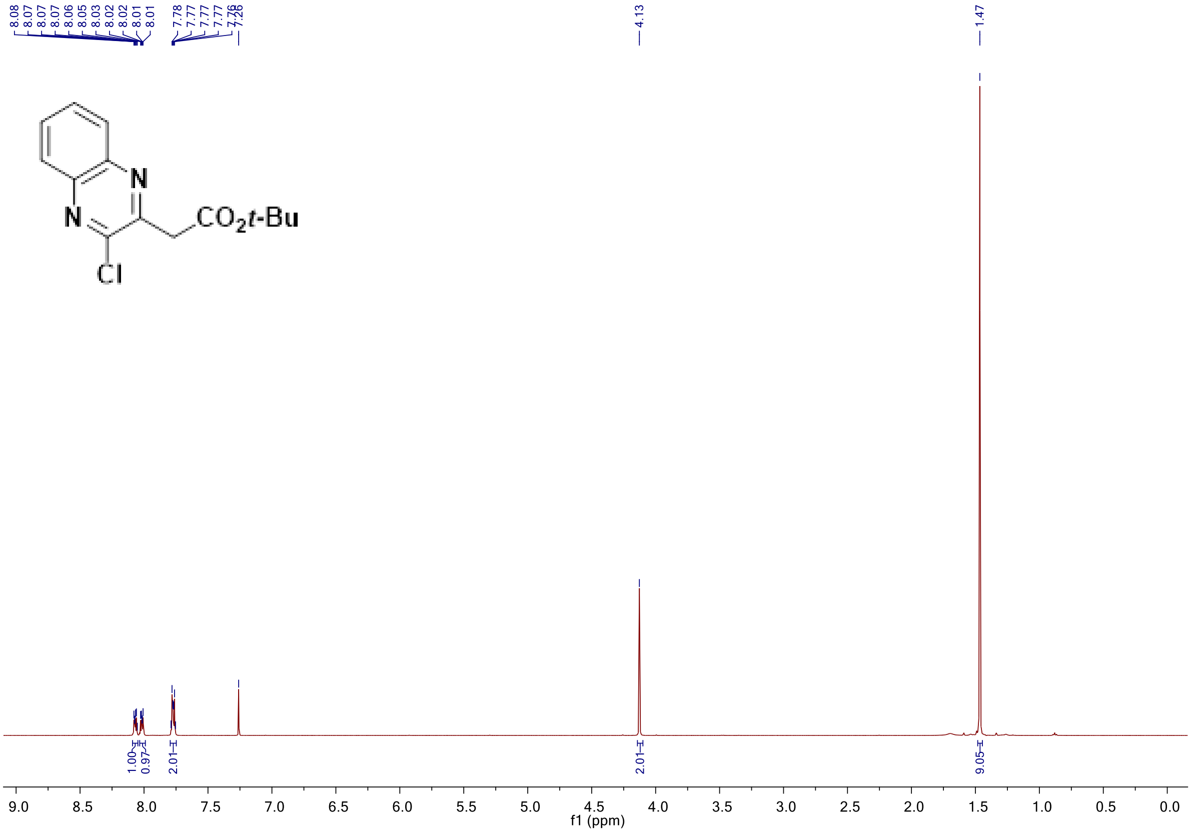

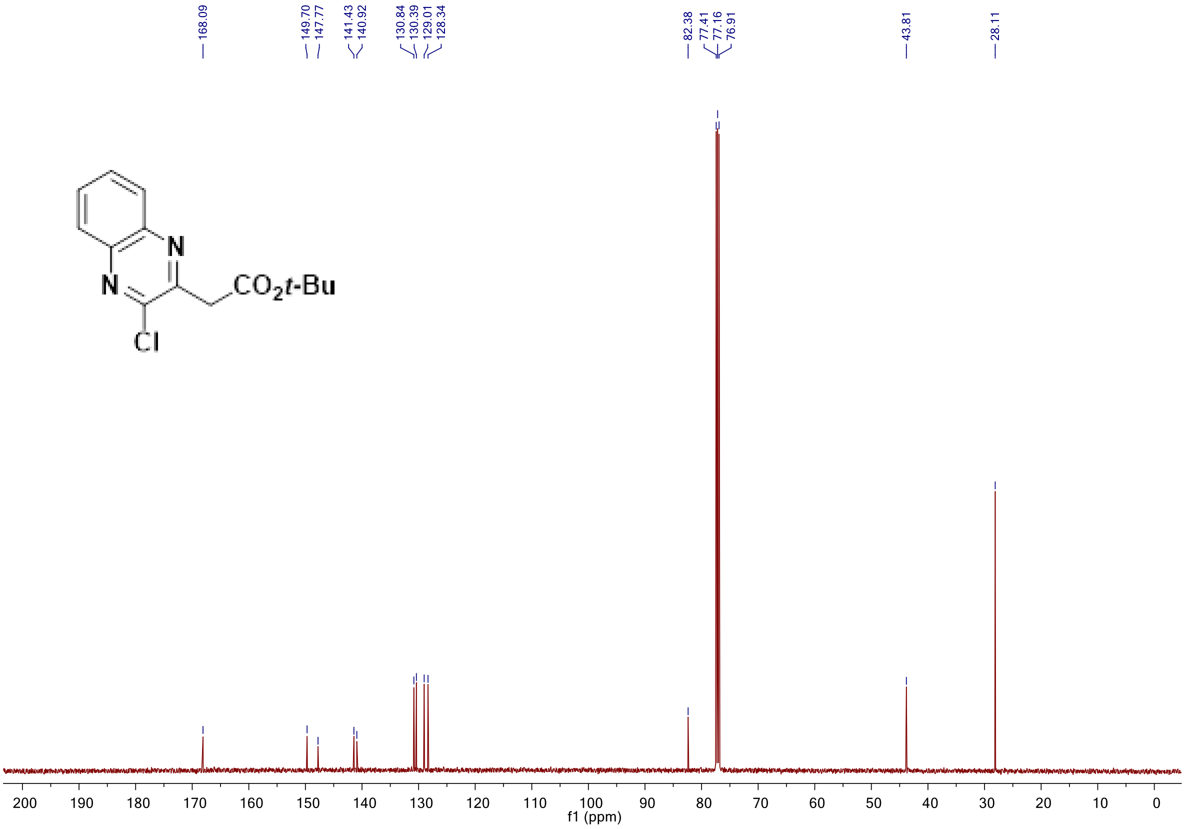

2-(2-Methyl-2-phenylbutyl)benzo[*d*]thiazole

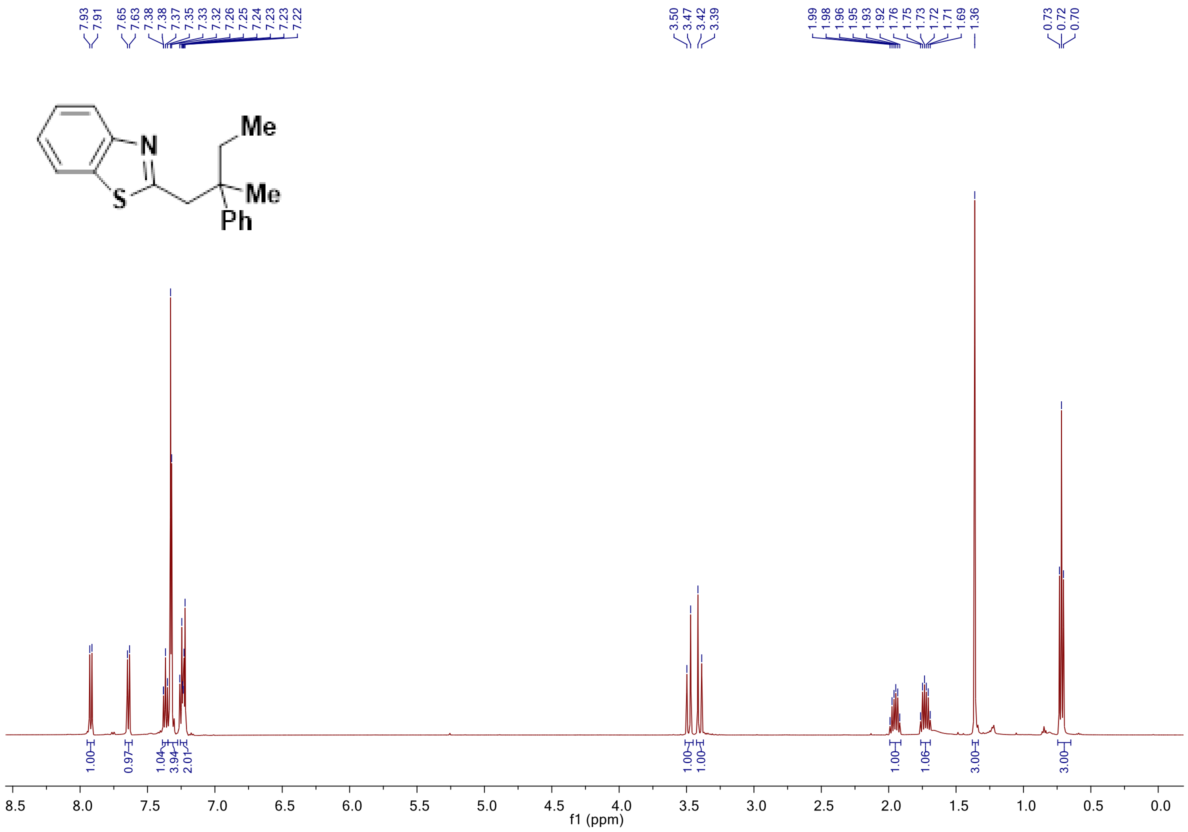

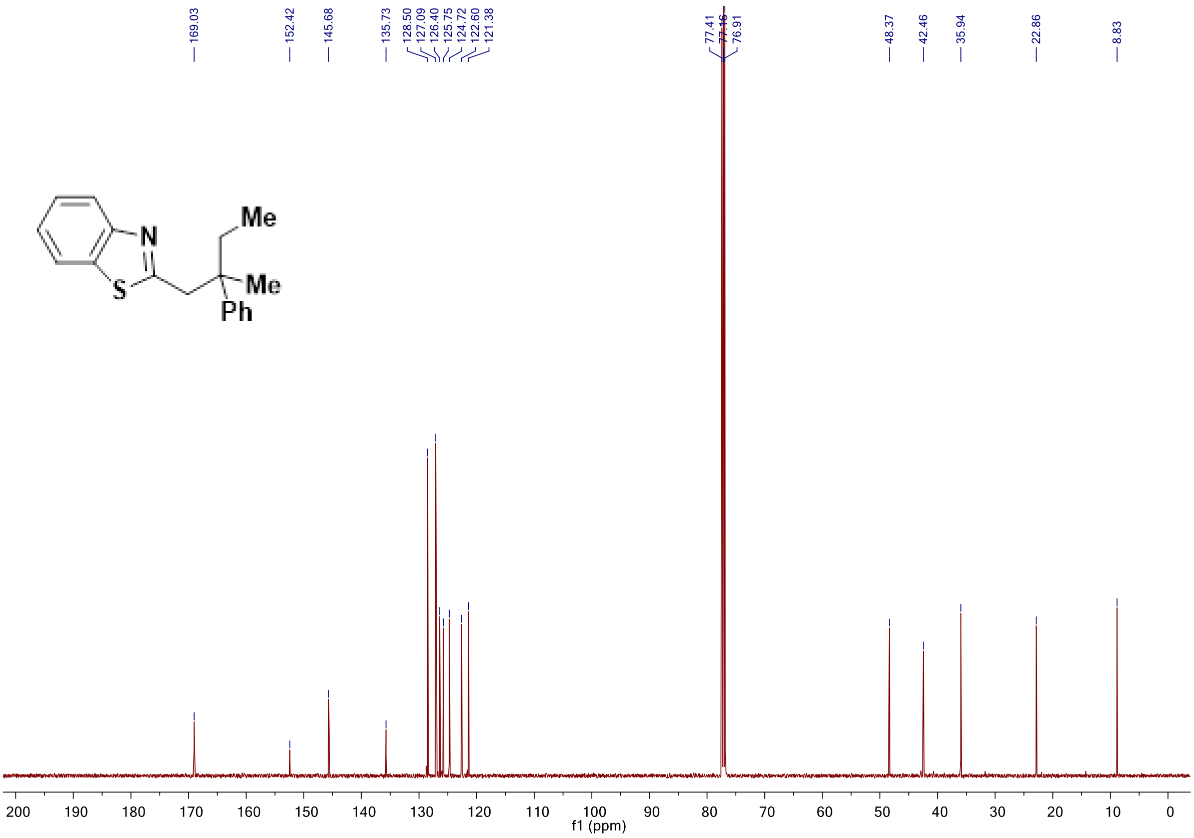

Notes and References

1. Heller, B., Sundermann, B.; Buschmann, H.; Drexler, H.-J.; You, J.; Holzgrabe, U.; Heller, E.; Oehme, G. Photocatalyzed [2 + 2 + 2]-Cycloaddition of Nitriles with Acetylene: An Effective Method for the Synthesis of 2-Pyridines under Mild Conditions. *J. Org. Chem.* **2002**, *67*, 4414.
2. Lv, X.; Yang, H.; Shi, T.; Xing, D.; Xu, X.; Hu, W. Rhodium(II)-Catalyzed Formal [4+1]-Cycloaddition of Pyridotriazoles and Propargyl Alcohols: Synthesis of 2,5-Dihydrofurans. *Adv. Synth. Catal.* **2019**, *361*, 1265.
3. Martín, R.; Buchwald, S. L. An Improved Protocol for the Pd-Catalyzed α-Arylation of Aldehydes with Aryl Halides. *Org. Lett.* **2008**, *10*, 4561.
4. Shen, H. C.; Ding, F.-X.; Colletti, S. L. α-Heteroarylation of Esters, Lactones, Amides, and Lactams by Nucleophilic Aromatic Substitution. *Org. Lett.* **2006**, *8*, 1447.
5. Dines, J. A.; Marson, C. M. A Direct Alkylation Route to Branched Derivatives of Suberoylanilide Hydroxamic Acid (SAHA), A Potent Non-selective Inhibitor of Histone Deacetylases. *Tetrahedron* **2016**, *72*, 8584.
6. Morgentin, R.; Jung, F.; Lamorlette, M.; Maudet, M.; Ménard, M.; Plé, P.; Pasquet, G.; Renaud, F. An Efficient Large-scale Synthesis of Alkyl 5-Hydroxy-pyridinand Pyrimidin-2-yl Acetate. *Tetrahedron* **2009**, *65*, 757.
7. Fallan, C.; Lam, H. W. Enantioselective Nickel-Catalyzed Michael Additions of Azaarylacetates and Acetamides to Nitroalkenes. *Chem. Eur. J.* **2012**, *18*, 11214.
8. Kazuhiro, K.; Takeshi, M.; Susumu, I.; Atsushi, T.; Miyuki, T.; Osamu, M.; Hisatoshi. K.; Direct Synthesis of *N*-Hydroxy β-Amino Acid Esters from Carboxylic Esters and Nitrones. *Bull. Chem. Soc. Jpn.* **2000**, *73*, 2805.
9. Bruno, N. C.; Tudge, M. T.; Buchwald, S. L. Design and Preparation of New Palladium Precatalysts for C–C and C–N Cross-coupling Reactions. *Chem. Sci.* **2013**, *4*, 916.
10. (a) Burés, J. A Simple Graphical Method to Determine the Order in Catalyst. *Angew. Chem. Int. Ed.* **2016**, *55*, 2028. (b) Burés, J. Variable Time Normalization Analysis: General Graphical Elucidation of Reaction Orders from Concentration Profiles. *Angew. Chem. Int. Ed.* **2016**, *55*, 16084.
11. Saga, Y.; Nakayama, Y.; Watanabe, T.; Kondo, M.; Masaoka, S. Visible-Light-Driven Hydroacylation of Unactivated Alkenes Using Readily Available Acyl Donors. *Org. Lett.* **2023**, *7*, 1136.
